# Development of highly efficient platinum catalysts for hydroalkoxylation and hydroamination of unactivated alkenes

**DOI:** 10.1038/s41467-021-22287-w

**Published:** 2021-03-29

**Authors:** Yali Zhou, Xingjun Xu, Hongwei Sun, Guanyu Tao, Xiao-Yong Chang, Xiangyou Xing, Bo Chen, Chen Xu

**Affiliations:** 1 Shenzhen Grubbs Institute and Department of Chemistry, Guangdong Provincial Key Laboratory of Catalysis, Southern University of Science and Technology, Shenzhen, Guangdong China; 2 Academy for Advanced Interdisciplinary Studies, Southern University of Science and Technology, Shenzhen, Guangdong China

**Keywords:** Catalysis, Catalyst synthesis, Synthetic chemistry methodology

## Abstract

Hydrofunctionalization, the direct addition of an X–H (e.g., X=O, N) bond across an alkene, is a desirable strategy to make heterocycles that are important structural components of naturally occurring molecules. Described here is the design and discovery of “donor–acceptor”-type platinum catalysts that are highly effective in both hydroalkoxylation and hydroamination of unactivated alkenes over a broad range of substrates under mild conditions. A number of alkene substitution patterns are accommodated, including tri-substituted, 1,1-disubstituted, (*E*)-disubstituted, (*Z*)-disubstituted and even mono-substituted double bonds. Detailed mechanistic investigations suggest a plausible pathway that includes an unexpected dissociation/re-association of the electron-deficient ligand to form an alkene-bound “donor–acceptor”-type intermediate. These mechanistic studies help understand the origins of the high reactivity exhibited by the catalytic system, and provide a foundation for the rational design of chiral catalysts towards asymmetric hydrofunctionalization reactions.

## Abstract (web summary)

The direct addition of an O–H (hydroalkoxylation) or an N–H (hydroamination) bond across an alkene is a useful strategy to access biologically relevant heterocycles. Here, the authors report “donor–acceptor”-type platinum catalysts that are effective for both hydroalkoxylation and hydroamination of unactivated alkenes, with broad substrate scope and use of mild conditions.

## Introduction

**Fig. 1:**
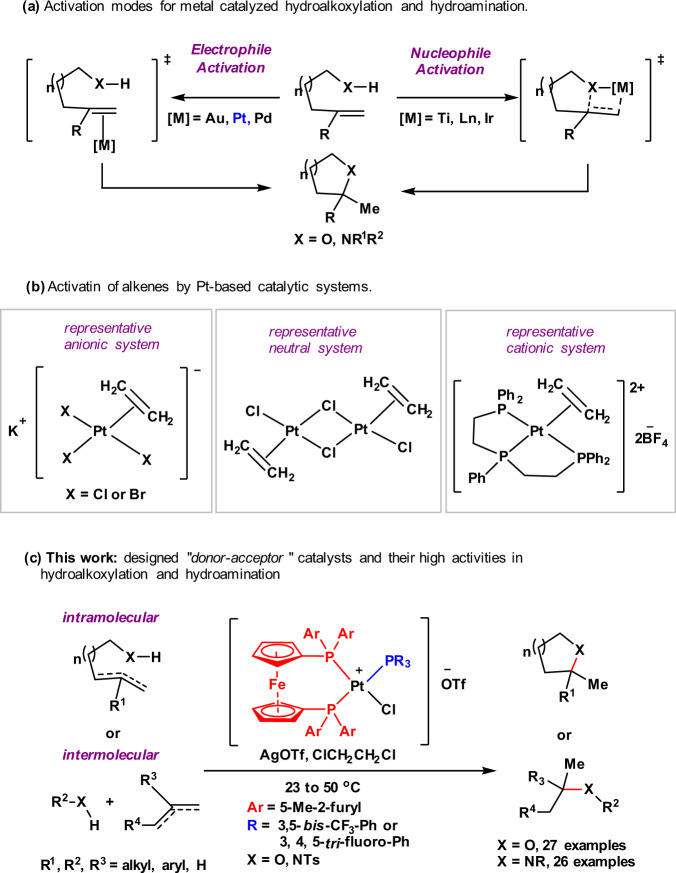
Pt-catalyzed hydroalkoxylation and hydroamination of unactivated alkenes. **a** Activation modes for metal-catalyzed hydroalkoxylation and hydroamination. **b** Activation of alkenes by Pt-based catalytic systems. **c** This work: designed “donor–acceptor” catalysts and their high activities in hydroalkoxylation and hydroamination.

Aliphatic or aromatic ethers and amines are often significant structural components of biologically active natural products^1,2^. Of the methods to generate these prevalent motifs, the most straightforward and atom-economical route would involve the direct addition of an O–H or an N–H bond across an alkene, commonly referred to as hydroalkoxylation and hydroamination, respectively. In the past decade, there have been a growing number of reports for catalysts that can facilitate hydroalkoxylation^3–14^ and hydroamination^15–29^. To the best of our knowledge, the most frequently used methods are metal-catalyzed conditions, which have been primarily enabled by the activation of either the nucleophile (OH or NH group)^6,8,9,14,16^ or the electrophile (C=C bond)^4,5,19,20,23^ with varying degrees of efficiency (Fig. 1a). Of the metals that are able to catalyze hydroalkoxylation and hydroamination, platinum catalysts attracted considerable attention for its unique Lewis acidity towards C=C double bonds^30,31^. Platinum-based complexes that can selectively activate simple C=C double bonds could fall into the following three categories (Fig. 1b)^32^. (1) Anionic systems, such as the famous Zeise’s salt (K[PtCl_3_(C_2_H_4_)]·H_2_O) and Chojnacki’s salt (K[PtBr_3_(C_2_H_4_)]). (2) Neutral systems^33,34^, such as [PtCl_2_(C_2_H_4_)_2_]_2_ that have been introduced by Widenhoefer^4,19,33,34^. (3) Cationic systems, with diamine^35–38^, bisphosphine^39,40^, carbene^41–43^ or pincer ligands^44–46^; in particular, Gagné discovered a series of elegant cationic Pt(II) complexes, such as (PPP)Pt^2+^ and (PP)PPt^2+^, which efficiently inhibit *β*-H elimination as a competing decomposition pathway^31,40,46^. Among the three activation modes, coordination to the cationic Pt derivatives renders the multiple bonds more electrophilic and thus susceptible to nucleophilic attack^47,48^.

Recently, Grubbs described the design and synthesis of novel “donor–acceptor”-type platinum catalysts by introducing electron-rich bisphosphine and electron-deficient dimethylphosphine oxide on the platinum center, and these catalysts have been demonstrated to be highly effective in the hydration of nitriles and cyanohydrins at ambient temperature^49^. We envisioned that this catalyst design concept may be suitable for the cationic, electrophilic activation of olefins: introducing both electron-rich “donor” ligand and electron-deficient “acceptor” ligand to the Pt center would make a soft and stable cationic system^50,51^, thus leading to a more effective activation of soft Lewis bases, such as C–C multiple bonds.

Herein, we disclose readily accessible “donor–acceptor”-type platinum catalysts that are highly active in both hydroalkoxylation and hydroamination, along with mechanistic studies that reveal the origins of the high reactivity of the catalytic system. The hydroalkoxylation and hydroamination reactions catalyzed by the Pt-complexes typically proceed at mild temperatures (23*–*50 °C), and encompass a remarkably broad substrate scope, including alkenes with various substitution patterns (Fig. 1c). Chiral platinum catalysts bearing (*R*)-BINAP or (*R*)-Difluorphos and an electron-deficient monodentate ligand with hydrogen-bonding site allows the asymmetric hydroalkoxylation and hydroamination with enantioselectivities, supporting that a more complex metal catalyst than triflic acid is involved^52–55^. Mechanistic data reveal an unusual dissociation/re-association of the electron-deficient monophosphine, and that a polarizable alkene-bound “donor–acceptor”-type Pt-intermediate could be formed, thus facilitating activation of simple alkenes.

## Results

### Screening of the optimal catalysts

**Fig. 2:**
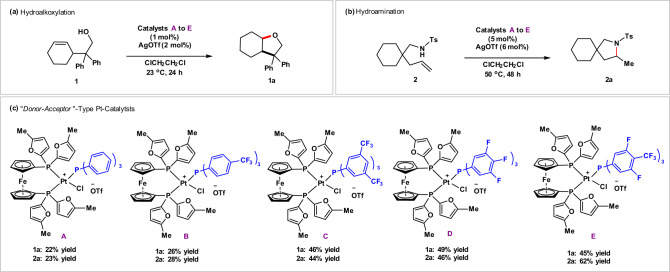
Development of “donor–acceptor” type Pt catalysts for hydroalkoxylation and hydroamination. **a** Hydroalkoxylation. **b** Hydroamination. **c** Screening of “donor–acceptor”-type Pt catalysts for hydroalkoxylation and hydroamination.

In Grubbs’ previous studies on the hydration of nitriles and cyanohydrins, more electron-rich groups in the “donor” moiety were discovered to increase the activity of the catalysts^49^. Therefore, we take the electron-rich 1.1′-[bis(5-methyl-2-furanyl)phosphine]ferrocene “donor” ligand and modulate the electronic nature of the monodentate “acceptor” ligands. As shown in Fig. 2, five Pt catalysts **A**–**E** were synthesized via the reaction of 1.1′-[bis(5-methyl-2-furanyl)phosphine]ferrocene platinum dichloride (dmfpfPtCl_2_) with the corresponding monodentate phosphine ligands (in blue) in the presence of silver triflate, respectively (Please see the details in Supplementary Information). Then the activity of the synthesized platinum catalysts was evaluated by performing hydroalkoxylation of **1** and hydroamination of **2** (Fig. 2). The results show that catalyst **A**, with triphenylphosphine as the monodentate ligand, has the worst reactivity for both hydroalkoxylation of **1** and hydroamination of **2**. Catalyst **B** that bears electron-deficient monodentate tris(4-(trifluoromethyl)phenyl)phosphine slightly improved the reactivity for both hydroalkoxylation and hydroamination. Catalyst **C** harboring the more electron-deficient monodentate tris(3,5-bis(trifluoromethyl)phenyl)phosphine displays noticeably increased catalytic activity, providing hydroalkoxylation product **1a** and hydroamination product **2a** in 46% and 44% yields, respectively. The observation that more electron-deficient tri-aryl phosphine ligand in the “acceptor” moiety resulted in higher catalytic activity as demonstrated by the improved performance of catalysts **A**, **B**, and **C** supports Grubbs’ “donor–acceptor” catalyst design^49^. Catalysts **D** and **E** bearing other two different electron-deficient monodentate ligands, tris(3,4,5-trifluorophenyl)phosphine and tris(3,5-difluoro-4-(trifluoromethyl)phenyl)phosphine, also show good reactivity for both hydroalkoxylation and hydroamination, and have their own advantages for different substrates. The trend that more electron-rich group in the “donor” part^49^ and/or the more electron-deficient group in the “acceptor” part increased the activity of the Pt catalysts could be explained: the cationic Pt(II) was stabilized by the strong σ donating from the electron-rich bidentate ligand, while the electron-deficient monodentate ligand which acts as weak σ donor but strong π acceptor makes the Pt(II) center more electrophilic toward multiple bonds^56^.

### Scope for hydroalkoxylation

**Fig. 3:**
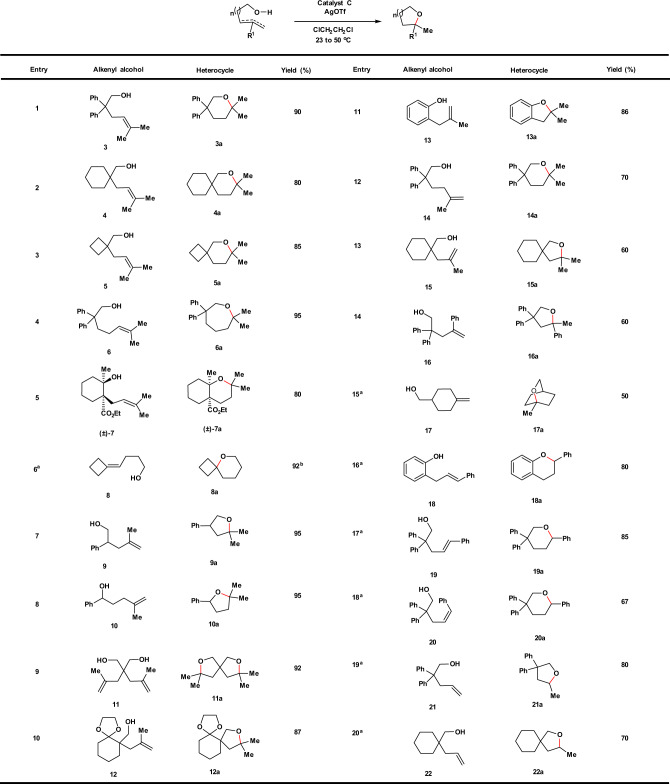
Substrate scope for intramolecular hydroalkoxylation. General conditions: Catalyst **C** (1 mol%), AgOTf (2 mol%), ClCH_2_CH_2_Cl, 23 °C, 24 h. Yields of isolated products are given. ^a^Catalyst **C** (2 mol%), AgOTf (4 mol%), ClCH_2_CH_2_Cl, 50 °C, 48 h. Yields of isolated products are given. ^b^Determined by ^1^H NMR analysis using 4,4'-di-*tert*-butyl-1,1'-biphenyl as an internal standard.

we next investigated the scope of intramolecular hydroalkoxylation with catalyst **C**, which contains the commercially available and inexpensive tris(3,5-bis(trifluoromethyl)phenyl)phosphine as the monodentate ligand (Fig. 3). All the reactions were conducted under mild conditions (either at 23 or 50 °C). The catalytic protocol displays excellent generality with Markovnikov regioselectivity, and is notably applicable to the synthesis of sterically hindered ethers with fused-, bridged-, and spiro-ring systems (Fig. 3, entries 5; 15; 2, 3, 6, 9, 10, 13, and 20). Various hydroxyl groups, such as primary, secondary, tertiary alcohols and phenols serve as good nucleophiles. Different olefins with various substitution patterns including trisubstituted (Fig. 3, entries 1–6), 1,1-disubstituted (Fig. 3, entries 7–15), 1,2-disubstituted (both *cis* and *trans*) (Fig. 3, entries 16–18), and even mono-substituted (Fig. 3, entries 19 and 20) double bonds work well in this reaction. Good to excellent yields were obtained in all cases reported.

**Fig. 4:**
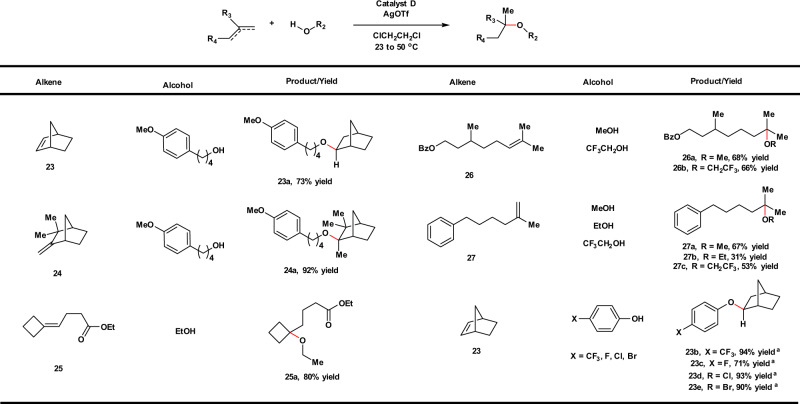
Substrate scope for intermolecular hydroalkoxylation. General conditions: catalyst **D** (5 mol%), AgOTf (6 mol%), 50 °C, 16–24 h. Yields of isolated products were given. ^a^Catalyst **D** (2 mol%), AgOTf (3 mol%), 23 °C, 24 h.

Expanding our investigation to intermolecular hydroalkoxylation, we discovered that catalyst **D** was the optimal catalyst whereas **C** gave slightly reduced yields over a range of different substrates (Fig. 4). Higher catalyst loadings (5 mol%) and slightly elevated temperatures are required for optimal conversion of the intermolecular reactions reported here. Alkenes with ring strain, such as norbornene (**23**), comphene (**24**), and four-membered carbocycles (**25**) all productively undergo intermolecular hydroalkoxylation reactions with alcohols. Acyclic alkenes, such as **26** and **27** that are less reactive substrates than their cyclic counterparts were coupled with alcohols in moderate yields. These reactions generally stalled at 30–70% conversions depending on the nature of the nucleophile used; longer reaction times or higher catalyst loadings do not help to convert the reactions further. Intermolecular hydroalkoxylation of norbornene (**23**) with *para*-substituted phenols was also investigated. Phenols have a potential to engage in hydroarylation reactivity^57^; however, we only observed hydroalkoxylation products (ethers) when *para*-substituted phenols (CF_3_, F, Cl, Br) and norbornene (**23**) were treated with **D**/AgOTf at either room temperature or 50 °C. In contrast, bis-*ortho*-hydroarylation products were exclusively generated when triflic acid (TfOH) alone was used as the catalyst (See Supplementary Information for further details). These results suggest a distinct mechanistic pathway for our catalytic system, different from that of a Brønsted acid-catalyzed hydroalkoxylation.

### Substrate scope for hydroamination

**Fig. 5:**
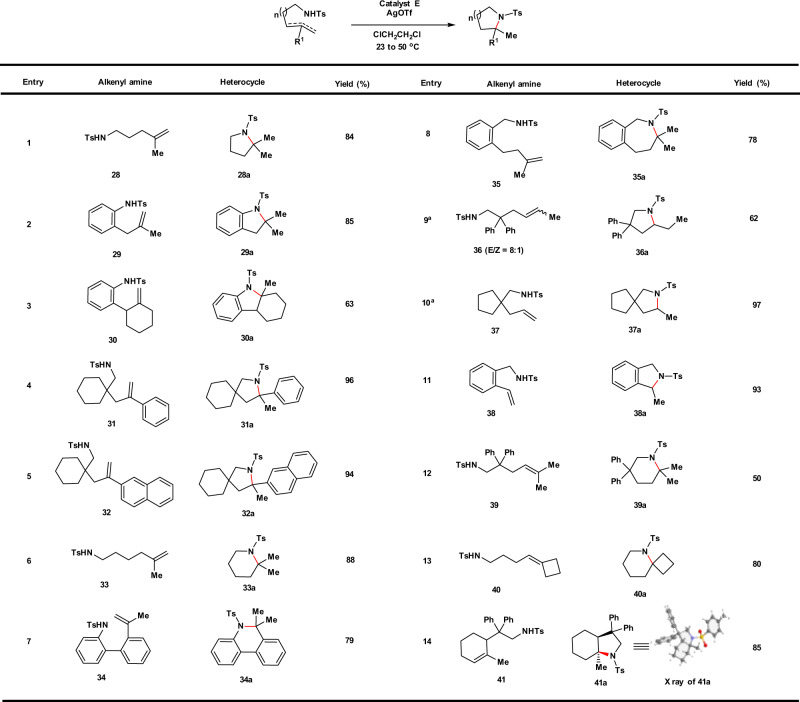
Substrate scope for intramolecular hydroamination. General conditions: catalyst **E** (1 mol%), AgOTf (2 mol%), 23 °C, 24 h. Yields of isolated products were given. ^a^Catalyst **E** (5 mol%), AgOTf (6 mol%), 50 °C, 48 h.

**Fig. 6:**
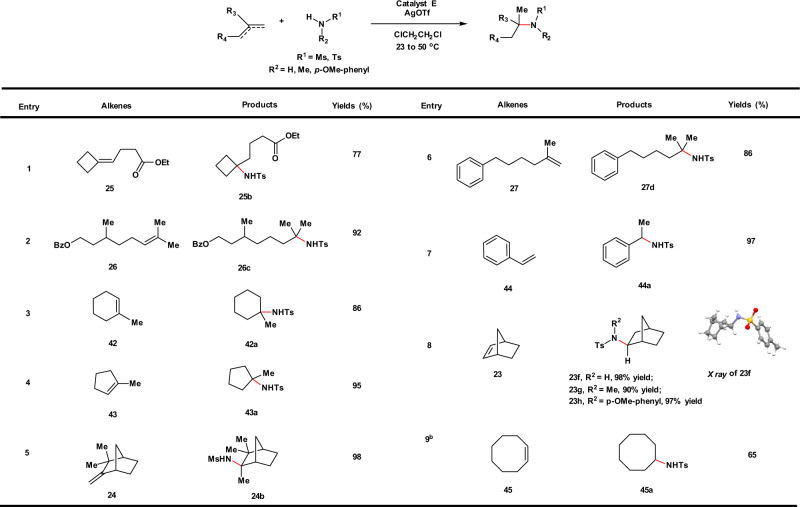
Substrate scope for intermolecular hydroamination. General conditions: catalyst **E** (2 mol%), AgOTf (4 mol%), 23 °C, 8–24 h. Yields of isolated products were given. ^a^Catalyst **E** (5 mol%), AgOTf (6 mol%), 50 °C, 48 h. Ts tosyl, Ms mesyl.

The “donor–acceptor”-type Pt catalysts not only are capable of catalyzing hydroalkoxylation but can also affect the hydroamination of unactivated alkenes in generally good yields. As compiled in Fig. 5, the catalyst **E**, even used at lower loading of 1 mol%, was shown to be capable of effecting intramolecular alkene hydroamination with sulfonamides at ambient temperatures, providing various nitrogen-heterocycles that are important structural motifs in naturally occuring and pharmaceuticals. A wide range of alkene substitution patterns is tolerated, including 1,1-disubstituted (Fig. 5, entries 1–8), 1,2-disubstituted (Fig. 5, entry 9), mono-substituted (Fig. 5, entries 10 and 11), and trisubstituted alkenes (Fig. 5, entries 12–14). The relative stereochemistry of **41a** was unambiguously confirmed by X-ray crystallography (Fig. 5, entry 14). Next, we examined the viability of more difficult intermolecular alkene aminations with *p*-toluenesulfonamide, (*p*-tolylsulfonyl)methylamine, *N*-tosyl-4-methoxyaniline, and methanesulfonamide (Fig. 6). A number of alkene substitution patterns were accommodated, including trisubstituted (Fig. 6, entries 1–4), terminal (Fig. 6, entries 5–7), and 1,2-disubstituted alkenes (Fig. 6, entries 8 and 9). Both cyclic and acyclic alkene partners can be aminated successfully. The structure of **23f** was verified by X-ray crystallography.

### Asymmetric hydroalkoxylation and hydroamination

**Fig. 7:**
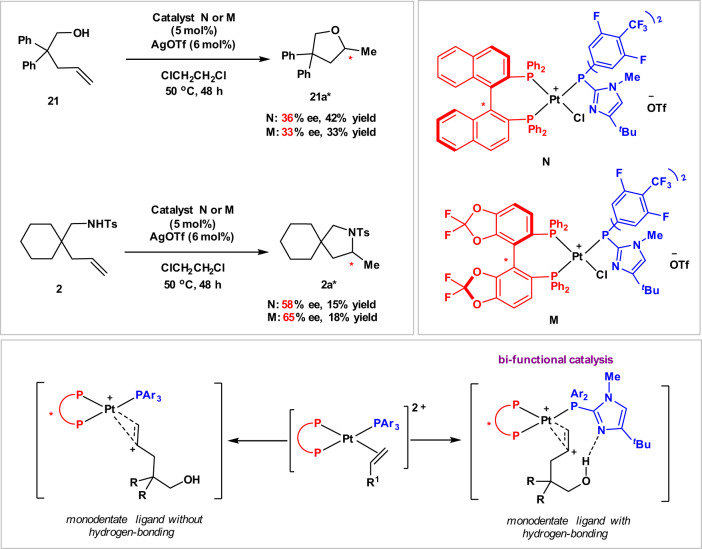
Development of bi-functional catalysis for catalytic asymmetric hydroalkoxylation and hydroamination. **a** Pt-catalyzed asymmetric intermolecular hydroalkoxylation and hydroamination of unactivated alkenes. **b** Catalysts **N** and **M**. **c** Bi-functional catalysis for asymmetric hydroalkoxylation and hydroamiantion.

We then explored the catalytic asymmetric hydroalkoxylation^58–61^ and hydroamination^62–66^ with the “donor–acceptor” catalytic system. Various chiral Pt-complexes generated in situ from chiral bisphosphine platinum dichlorides with electron-deficient tris(3,4,5-trifluorophenyl)phosphine were examined in the reaction of **21** and **2** under standard conditions. Disappointingly, none of the chiral Pt catalysts produced enantioselectivities (see further details in Supplementary Information). As shown in Fig. 7, we envisioned that a bi-functional catalysis where the monodentate ligand has a basic group that can form hydrogen bonding with the nucleophile would assist recognition of prochiral faces of the alkenes^59,67^. To our delight, catalysts **N** and **M** bearing (*R*)-BINAP and (*R*)-Difluorphos as “donor” ligands and the monodentate “acceptor” ligand with a hydrogen-bonding site induced moderate enantioselectivities in both hydroalkoxylation of **21** and hydroamination of **2**.

### Mechanistic studies

Recently, several groups have independently demonstrated that TfOH can catalyze the additions of oxygen- and nitrogen-based nucleophiles to simple alkenes with comparable efficiency/selectivity as some metal triflates^52–55^. The aforementioned reports raised the question of a competitive acid-catalyzed pathway when metal triflates are employed. Therefore, we elected to perform a detailed analysis of the possible mechanistic pathways for the “donor–acceptor” Pt-catalyzed hydroalkoxylation of alkenes.

**Fig. 8:**
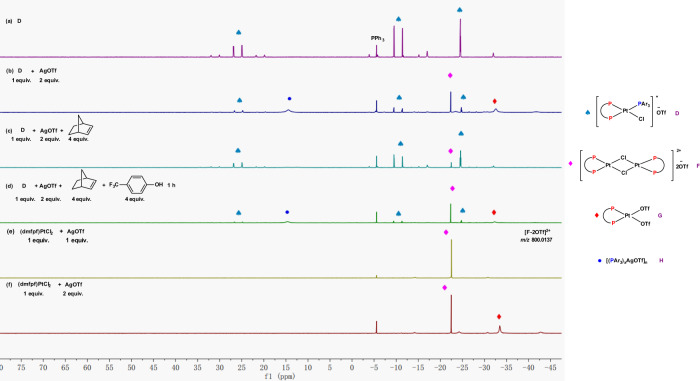
^31^P NMR spectra for monitoring the reaction of catalyst D + AgOTf + norbornene + 4-trifluromethylphenol (PPh_3_ was used as an external standard). **a** ^31^P NMR spectra of complex D. **b** ^31^P NMR spectra of a mixture of complex D (1 equiv.) and AgOTf (2 equiv.). **c** ^31^P NMR spectra of a mixture of complex D (1 equiv.), AgOTf (2 equiv.), and norbornene (4 equiv.). **d** ^31^P NMR spectra of a mixture of complex D (1 equiv.), AgOTf (2 equiv.), norbornene (4 equiv.), and 4-trifluoromethylphenol (4 equiv.). **e** ^31^P NMR spectra of a mixture of (dmfpf)PtCl_2_ (1 equiv.), and AgOTf (1 equiv.). **f** ^31^P NMR spectra of a mixture of (dmfpf)PtCl_2_ (1 equiv.), and AgOTf (2 equiv.). Specifically, (dmfpf)PtCl_2_:1.1′-[bis(5-methyl-2-furanyl)phosphine]ferrocene platinum dichloride; AgOTf: silver triflate.

The mechanistic study commenced with monitoring the reaction of norbornene and 4-trifluromethylphenol catalyzed by **D** and AgOTf in deuterated dichloromethane at 23 °C by ^31^P NMR (Fig. 8 and please also see Supplementary Fig. 6 for corresponding ^1^H NMRs). When AgOTf (2 equiv.) was added into catalyst **D** (1 equiv.), ^31^P NMR revealed the appearance of three new peaks at *δ* −33.5 (d, ^1^*J*_Pt-P_ = 4511 Hz), −22.5 (d, ^1^*J*_Pt-P_ = 4018 Hz), and 14.4 (s, broad) ppm, respectively (Fig. 8b). The peak at *δ* −22.5 ppm was proposed to be the dinuclear complex **F** that can be generated independently by mixing 1 equivalent of dmfpfPtCl_2_ with 1 equivalent of AgOTf (Fig. 8e), which was also confirmed by ESI-MS analysis ([F-2OTf]^2+^, m/z = 800.0137). We were able to grow the single crystal of the corresponding DPPF derived dinucleor Pt complex and confirmed its structure by X-ray. Please see Supplementary Information for details. The peak at *δ* −33.5 ppm was proposed to be complex **G** that can be prepared independently by mixing 1 equivalent of dmfpfPtCl_2_ with 2 equivalents of AgOTf (Fig. 8f). Complex **G** is moisture sensitive and when we attempted to grow single crystal of it, we only obtained [(P~P)Pt(H_2_O)_2_]^2+^(^−^OTf)_2_. Please see Supplementary Information for details. We also found that more AgOTf leads to more complex **G** appearing at *δ* −33.5 (d, ^1^*J*_Pt-P_ = 4511 Hz) in ^31^P NMR; however, too much AgOTf did not lead to more complex **G** (Supplementary Figs. 4, 7, 10, and 12 for adding 1, 2, 5, and 50 equiv. of AgOTf, respectively), indicating that there might be equilibrium between catalyst **D** and complex **G** in the presence of AgOTf. The third new peak at *δ* 14.4 ppm might belong to silver-phosphine complexes, such as [(Ar_3_P)_x_AgOTf]_n_ (**H**) with ^19^F signals at *δ* −78.9 (s), −131.6 (d, *J* = 21.3 Hz), and −154.8 (t, *J* = 20.8 Hz) ppm (Supplementary Fig. 5). Then 4 equivalents of norbornene were added to the above solution which led to the rapid disappearance of complexes **F**, **G**, and **H** (Fig. 8c). Finally, when 4-trifluoromethylphenol was added, ^1^H NMR confirmed that the hydroalkoxylation product **23b** was formed with the concurrent regeneration of the three complexes **F**, **G**, and **H** (Fig. 8d and please also see the ^1^H NMR in Supplementary Fig. 6). These results indicate that either one or both of complexes **F** and **G** might be the catalytic active species (Complex **H** has been excluded as a catalytic active specie; please see the control experiments in Supplementary Table 1, entry 24). Therefore, we would like to monitor the reaction pathways of the hydroalkoxylation catalyzed by complexes **F** and **G**, respectively.

As depicted in Supplementary Fig. 19a, b, the dinuclear complex **F** was immediately converted to the original catalyst **D** as soon as the monodentate ligand, tris(3,4,5-trifluorophenyl)phosphine, was added. Further treating the mixture with norbornene and 4-trifluromethylphenol did not lead to any change in ^31^P and ^1^H NMR (Supplementary Figs. 19c, d and 18). We have also conducted an experiment to monitor the reaction of complex **F** (1 equiv.) with norbornene (4 equiv.) and 4-trifluromethylphenol (4 equiv.) in the absence of the monodentate tris(3,4,5-trifluorophenyl)phosphine ligand by ^1^H and ^31^P NMRs, and we observed the formation of some hydroalkoxylation product **23b** (about 50% yield) after 1 h (Please see Supplementary Figs. 20 and  21). Another control experiment has been conducted to investigate the catalytic activity of complex **F** (Entry 1, Supplementary Table 7), where no intramolecular hydroalkoxylation of **1** proceeds in the presence of complex **F**. These results together suggest that complex **F** will more readily react with the monodentate ligand to form catalyst **D** than catalyze the hydroxylation reaction in the present system.

**Fig. 9:**
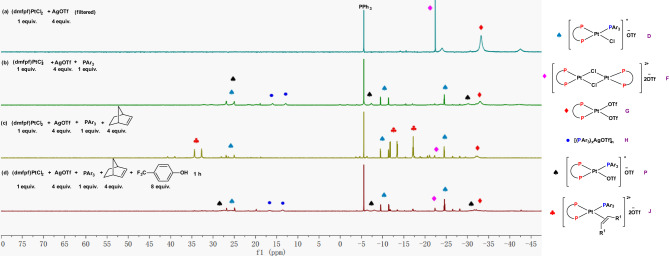
^31^P NMR spectra for the reaction of dmfpfPtCl_2_ + AgOTf (4 equiv.) + PAr_3_ + norbornene + 4-trifluromethylphenol (PPh_3_ was used as an external standard). **a** ^31^P NMR spectra of a mixture of (dmfpf)PtCl_2_ (1 equiv.) and AgOTf (4 equiv.) after filtration. **b** ^31^P NMR spectra of a mixture of (dmfpf)PtCl_2_ (1 equiv.), AgOTf (4 equiv), and tris(3,4,5-trifluorophenyl)phosphane (1 equiv.) after filtered. **c** ^31^P NMR spectra of a mixture of (dmfpf)PtCl_2_ (1 equiv.), AgOTf (4 equiv.), tris(3,4,5-trifluorophenyl)phosphane (1 equiv.), and norbornene (4 equiv.) after filtered. **d** ^31^P NMR spectra of a mixture of (dmfpf)PtCl_2_ (1 equiv.), AgOTf (4 equiv.), tris(3,4,5-trifluorophenyl)phosphane (1 equiv.), norbornene (4 equiv.), and 4-trifluoromethylphenol (8 equiv.). Specifically, (dmfpf)PtCl_2_:1.1′-[bis(5-methyl-2-furanyl)phosphine]ferrocene platinum dichloride; AgOTf: silver triflate; PAr_3_: tris(3,4,5-trifluorophenyl)phosphine.

We then turned our attention to monitoring the hydroalkoxylation catalyzed by the other potential catalytic active specie complex **G** (Fig. 9). However, we were unable to prepare pure complex **G** with 1.1′-[bis(5-methyl-2-furanyl)phosphine]ferrocene as the bidentate donor ligand^21^: the dinuclear complex **F** was always inevitable. As shown in Fig. 9a, treating (dmfpf)PtCl_2_ with 4 equivalents of AgOTf provides a mixture of **G** and **F** with a relatively high ratio (7:1), which was then filtered off AgCl and used for the following experiment. AgCl will re-enter into the solution and react with complex **G** to make catalyst **D** in the presence of monodentate ligand. When the monodentate ligand was added into the above mixture (Fig. 9b), except catalyst **D** that might be generated from the dinuclear complex **F**, a new complex appearing at *δ* 25.8 (d, ^2^*J*_P-P_ = 411 Hz, ^1^*J*_Pt-P_ = 2584 Hz), −6.2 (d, ^2^*J*_P-P_ = 411, ^1^*J*_Pt-P_ = 2745 Hz), −29.9 (broad, ^1^*J*_Pt-P_ = 4289 Hz) ppm was proposed to be **P** with both the electron-donating bidentate and electron-deficient monodetate ligands coordinating to the Pt center (Please also see the details in the Supplementary Information for the synthesis of complex **P**). Then 4 equivalents of norbornene were added, and a new group of peaks at *δ* 33.6 (dd, ^2^*J*_P-P_ = 410, 22 Hz, ^1^*J*_Pt-P_ = 3075), −12.6 (dd, ^2^*J*_P-P_ = 411, 23 Hz, ^1^*J*_Pt-P_ = 3463), −17.2 (t, ^2^*J*_P-P_ = 22 Hz, ^1^*J*_Pt-P_ = 1873) ppm were observed in ^31^P NMR (Fig. 9c). Because they all disappeared after adding 4-trifluoromethylphenol (Fig. 9d, and ^1^H NMR in Supplementary Fig. 25 showed the complete formation of hydroalkoxylation product **23b**), we proposed that this group of new peaks belongs to an alkene-bound “donor–acceptor”-type intermediate **J**. Notably, filtrating AgCl from the reaction media allowed us to identify the key intermediate **J**, whereas it is no clearly visible when the filtration is not carried out (Supplementary Fig. 23). AgCl will re-enter into the solution and react with complex **G** to make catalyst **D** in the presence of monodentate ligand.

**Fig. 10:**
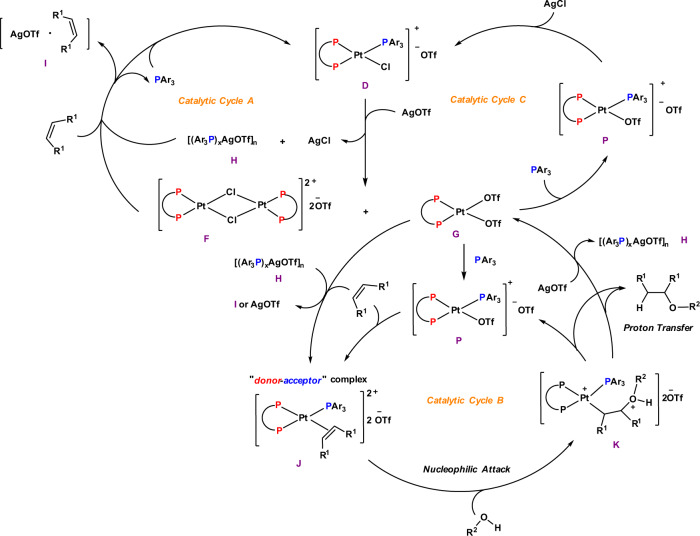
Proposed mechanism for “donor–acceptor” Pt complex catalyzed hydroalkoxylation of unactivated alkenes. Specifically, **D**: Catalyst; **F**: a dinuclear intermediate after Pt–P bond dissociation; **G**: an intermediate after Pt–Cl and Pt–P bonds dissociation; **H**: a silver-phosphine complexe; **I**: a silver-alkene complexe; **J**: an alkene-bound Pt (II) intermediate; **K**: transition state for nucleophilic attack of an alcohol to complex **J**; **P**: an intermediate after Pt–Cl bond dissociation.

Based on the NMR experiments, we propose a plausible mechanism for “donor–acceptor”-type Pt complex catalyzed hydroalkoxylation of unactivated alkenes (Fig. 10). When catalyst **D** was treated with AgOTf, both of the monophosphine ligand and chloride ion dissociate from the Pt center and react with AgOTf to generate complex **H** and AgCl, respectively. Therefore, two Pt-complexes **F** and **G** were formed simultaneously. The silver-phosphine complex **H** reacts with external alkenes to form silver-alkene adducts, such as complex **I**, releasing the monophosphine ligand that will react with the dinuclear complex **F** to regenerate the original catalyst **D** (Fig. 10, *catalytic cycle A*); when the hydroalkoxylation reaction was finished and all the alkenes were consumed, AgOTf was released from complex **I**, and therefore catalyst **D** was converted to dinuclear **F** again. Complex **G**, in the presence of alkenes, reacts with electron-deficient monophosphine to form a 16-electron, “donor–acceptor”-type Pt complex **J** either directly or through complex **P** (Fig. 10, *catalytic cycle B*). Nucleophilic attack on the bound alkene by a free alcohol provides complex **K**. Proton transfer thereafter provides the hydroalkoxylation product and regenerates complexes **G** (or **P**) and **H**. Another possible competitive pathway for the catalytic active species **G** and **P** includes regeneration of the original catalyst **D** in the presence of AgCl (Fig. 10, *catalytic cycle C*).

In summary, we have described herein the developed “donor–acceptor”-type platinum catalysts that are superior in effecting both hydroalkoxylation and hydroamination with respect to mild reaction conditions and generality in substrate scope. Mechanistic studies suggested a plausible pathway that includes an unusual dissociation/re-association of the electron-deficient monophosphine ligand to generate an alkene bound “donor–acceptor” type intermediate. Efforts to improve the reactivity and enantioselectivity of the chiral platinum catalysts based on bi-functional catalysis for asymmetric hydrofunctionalization will be the subject of future studies.

## Methods

### General procedure for preparing “donor–acceptor” Pt catalyst

In an argon-filled glovebox, to a 4 mL vial with a magnetic stir bar were added (dmfpf)PtCl_2_ (100 mg, 0.12 mmol), silver trifluoromethanesulfonate (31 mg, 0.12 mmol, 1.0 equiv.), monophosphine ligand (0.13 mmol, 1.05 equiv.), and CH_2_Cl_2_ (2 mL). Then the vial was taken outside of the glovebox and was stirred at 23 °C for 12 h. The orange solution was filtered and CH_2_Cl_2_ was evaporated to provide orange solid, which was recrystallized in CH_2_Cl_2_ and hexane to give yellow precipitate.

### General procedure for intramolecular hydroalkoxylation

In an argon-filled glovebox, to a 4 mL vial with a magnetic stir bar were added catalyst **C** (0.001 mmol, 1 mol%), silver trifluoromethanesulfonate (0.002 mmol, 2 mol%), the substrate (0.1 mmol), and ClCH_2_CH_2_Cl (1 mL). Then the vial was taken outside of the glovebox and stirred at 23 °C for 24 h. The mixture was diluted with CH_2_Cl_2_, filtered through a pad of celite and concentrated. The residue was purified by silica gel chromatography to give the desired product.

### General procedure for intermolecular hydroalkoxylation

In an argon-filled glovebox, to a 4 mL vial with a magnetic stir bar were added catalyst **D** (0.01 mmol, 5 mol%), silver trifluoromethanesulfonate (0.012 mmol, 6 mol%), alkenes (0.2 mmol), alcohols (0.3 mmol, 1.5 equiv.), and ClCH_2_CH_2_Cl (1 mL). Then the vial was taken outside of the glovebox and stirred at 50 °C for 16 h. The reaction mixture was cooled to room temperature (23 °C) and diluted with CH_2_Cl_2_, filtered through a pad of celite and concentrated in vacue. The residue was purified by silica gel chromatography to give the desired product.

### General procedure for intramolecular hydroamination

To a 4 mL vial equipped with a magnetic stir bar were added the catalyst **E** (0.001 mmol, 1 mol%), silver trifluoromethanesulfonate (0.002 mmol, 2 mol%), and ClCH_2_CH_2_Cl (1.0 mL) in an argon-filled glovebox. The mixture was stirred at 23 °C for 1 h. Then to the mixture was added a solution of **37** (30.7 mg, 0.1 mmol) in ClCH_2_CH_2_Cl (0.5 mL). The vial was taken outside of the glovebox and stirred at 23 °C for 24 h. The mixture was diluted with CH_2_Cl_2_ and concentrated under reduced pressure. The residue was purified by flash chromatography to afford the desired product.

### General procedure for intermolecular hydroamination

In an argon-filled glovebox, to a 4 mL vial with a magnetic stir bar were added catalyst **E** (0.002 mmol, 2 mol%), silver trifluoromethanesulfonate (1.0 mg, 0.004 mmol, 4 mol%), alkenes (0.1 mmol), sulfonamide (0.15 mmol, 1.5 equiv.), and ClCH_2_CH_2_Cl (0.7 mL). Then the vial was taken outside of the glovebox and stirred at 23 or 50 °C for 6–48 h. The reaction mixture was diluted with CH_2_Cl_2_, filtered through a pad of celite and concentrated. The residue was purified by silica gel chromatography to afford the desired product.

## Supplementary information

Supplementary Information

## Data Availability

The data that support the findings of this study are available within the paper and its Supplementary Information files. Raw data are available from the corresponding author on reasonable request. Materials and methods, experimental procedures, characterization data, ^1^H, ^13^C, ^19^F, ^31^P NMR spectra, and mass spectrometry data are available in the Supplementary Information. Crystallographic data are available free of charge from the Cambridge Crystallographic Data Centre (https://www.ccdc.cam.ac.uk/) under reference number CCDC 1942024 (catalyst **E**), CCDC 1945616 (**41a**), CCDC 1942471 (**23f**), CCDC 2021353 (catalyst **M**, in the Supplementary Information), CCDC 2053040 (complex **Q**, in the Supplementary Information) and CCDC 2053041 (complex **R**, in the Supplementary Information), respectively.
