# Supplementary material for: Development of highly efficient platinum catalysts for hydroalkoxylation and hydroamination of unactivated alkenes
Source: Nat Commun. 2021 Mar 29;12:1953. doi: 10.1038/s41467-021-22287-w (PMC8007598; doi:10.1038/s41467-021-22287-w)
Supplement: Supplementary file 1 — Supplementary Information [file 41467_2021_22287_MOESM1_ESM.pdf]

Supplementary Information

Development of Highly Efficient Platinum Catalysts for Hydroalkoxylation and Hydroamination of Unactivated Alkenes

Yali Zhou[1§], Xingjun Xu[1§], Hongwei Sun[1], Guanyu Tao[1], Xiao-Yong Chang[1], Xiangyou Xing[1*], Bo Chen[2*], and Chen Xu[1*]

[1]Shenzhen Grubbs Institute and Department of Chemistry, Guangdong Provincial Key Laboratory of Catalysis, Southern University of Science and Technology, Shenzhen 518055, Guangdong, China

[2]Academy for Advanced Interdisciplinary Studies, Southern University of Science and Technology, Shenzhen 518055, Guangdong, China

[§]These two authors contributed equally to this work.

*Correspondence: xuc@sustech.edu.cn, chenb@sustech.edu.cn, xingxy@sustech.edu.cn

Supplementary Information

Contents

1.General Information 2
2. Synthesis of Catalysts 3
3. Development of Reaction Condition 7
4. Synthesis of Substrates 11
5. Substrate Scope 26
6. Catalytic Enantioselective Hydrofunctionnization 53
7. NMR Monitoring of Hydroalkoxylation Reaction 69
8. Kinetic Experiments 95
9. X-Ray Structure Determination 99
10. References 107
11. NMR Spectra 108

1. General Information

All reactions were carried out under an argon atmosphere with dry solvents under anhydrous conditions, unless otherwise noted. Reaction progress was monitored by thin-layer chromatography (TLC) or Agilent 1260 LCMS analyses. HPLC analysis was conducted on Agilent 1260 or Shimadzu LC-20A instrument using chiral column described below in detail. TLC was performed using 0.25 mm Tsingdao silica gel plates (60F-254) and visualized by exposure to UV light (254 nm). Flash column chromatography was performed using Tsingdao silica gel (60, particle size 0.040–0.063 mm). ^{1}H NMR (400 MHz, 600 MHz), ^{13}C NMR (101 MHz, 151 MHz), ^{31}P NMR(162 MHz, 243 MHz) and ^{19}F NMR (376 MHz, 565 MHz) spectra were recorded on a Bruker AV III HD spectrometer, and were reported in terms of chemical shift relative to residual $CDCl_3$ (δ 7.26 and δ 77.16 ppm, respectively), CD_2Cl_2 (δ 5.32 ppm and δ 53.84 ppm, respectively). Data for ^{1}H NMR spectra is reported as follows: chemical shift (δ ppm) (multiplicity, coupling constant (Hz), integration). Abbreviations are used as follows: s = singlet, bs = broad singlet, d = doublet, t = triplet, q = quartet, m = complex multiplet. High-resolution mass spectra (HRMS) was obtained from the SUSTech Mass Spectral Facility using Thermo Scientific™ Q Exactive™ Quadrupole-Orbitrap Mass Spectrometer. Reagents were purchased from commercial vendors as follows: Dichloro(1,5-cyclooctadiene)Platinum, silver salts (silver trifluoromethanesulfonate) were purchased from Sigma-Aldrich and stored in an argon-filled glovebox. Alcohols (methanol, ethanol) were commercial available.

2. Synthesis of Catalysts

General Procedure

Step 1: In an argon filled glovebox, to a 20 mL scintillation vial with a magnetic stir bar were added 1,1'-bis[bis(5-methyl-2-furanyl)phosphino]ferrocene (182 mg, 0.32 mmol), (COD)$PtCl_2$ (120 mg, 0.32 mmol) and 3 mL CH_2Cl_2, then the vial was taken outside of the dry box and the mixture was stirred at room temperature (23 °C) for 10 h. The yellow solution was filtered and then evaporated to provide yellow solid, which was recrystallized through 1 mL CH_2Cl_2 and 1 mL hexane to give yellow precipitate. The solid was collected and dried in *vacuo* to yield (dmfpf)$PtCl_2$ (220 mg, 82% yield).

^{1}H NMR (600 MHz, $CDCl_3$): δ 7.16 (s, 4H), 6.12 (d, J = 2.6 Hz, 4H), 4.37 (d, J = 15.9 Hz, 8H), 2.35 (s, 12H) ppm

^{13}C NMR (151 MHz, $CDCl_3$): δ 158.7, 140.8(dd, J = 102.0, 2.1 Hz), 126.2 – 126.1 (m), 107.9 – 107.7 (m), 75.3 – 75.2 (m), 73.7 – 73.6 (m), 14.21 ppm

^{31}P NMR (243 MHz, $CDCl_3$): δ -24.7 (d, $^1J_{Pt-P}$ = 3764 Hz) ppm

Step 2: In an argon filled glovebox, to a 4 mL vial with a magnetic stir bar were added (dmfpf)$PtCl_2$ (100 mg, 0.12 mmol), silver trifluoromethanesulfonate (31 mg, 0.12 mmol, 1.0 equiv.), monophosphine ligand (0.13 mmol, 1.5 equiv.) and 2 mL CH_2Cl_2. Then the vial was taken outside of the glovebox and the mixture was stirred at room temperature (23 °C) for 12 h. The orange solution was filtered and CH_2Cl_2 was evaporated to provide orange solid, which was recrystallized through CH_2Cl_2 and hexane to give yellow precipitate.

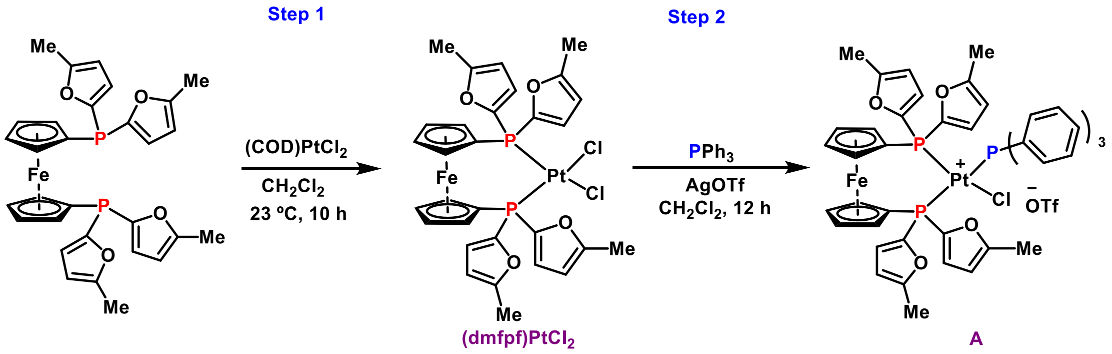

Yellow solid (130 mg, 89% yield).

^{1}H NMR (400 MHz, $CDCl_3$): δ 7.51 – 7.43 (m, 9H), 7.35 – 7.31 (m, 6H), 7.07 – 7.05 (m, 2H), 6.43 – 6.42 (m, 2H), 6.20 – 6.20 (m, 2H), 5.97 – 5.96 (m, 2H), 4.56 – 4.56 (m, 2H), 4.47 – 4.46 (m, 4H), 4.06 – 4.06 (m, 2H), 2.42 (s, 6H), 2.10 (s, 6H) ppm

^{13}C NMR (101 MHz, $CDCl_3$): δ 159.2 (dd, J = 46.7, 6.3 Hz), 139.4 (ddd, J = 105.3, 99.0, 3.0 Hz), 134.9 (dd, J = 10.2, 0.9 Hz), 134.0 (d, J = 16.1 Hz), 131.6 (d, J = 2.5 Hz), 129.2 (d, J = 10.0 Hz), 128.4 (d, J =

11.4 Hz), 126.1 (dd, J = 35.8, 17.9 Hz), 121.2 (q, J = 332.4 Hz), 108.8 (dd, J = 81.8, 7.7 Hz), 75.5 (d, J = 13.8 Hz), 75.3 – 74.8 (m), 14.3, 13.7 ppm

^{31}P NMR (243 MHz, CDCl$_3$): δ 23.3 (dd, $^2J_{P-P}$ = 445, 19 Hz, $^1J_{Pt-P}$ = 2555 Hz), -11.2 (dd, $^2J_{P-P}$ = 444, 19 Hz, $^1J_{Pt-P}$ = 2504 Hz), -23.7 (t, $^2J_{P-P}$ = 18 Hz, $^1J_{Pt-P}$ = 3701 Hz) ppm

^{19}F NMR (565 MHz, CDCl$_3$): δ -77.9 (s) ppm

HRMS (ESI+): calc'd for $C_{48}H_{43}ClFeO_4P_3Pt$ [M-OTf]$^+$: 1062.1054, found 1062.1045.

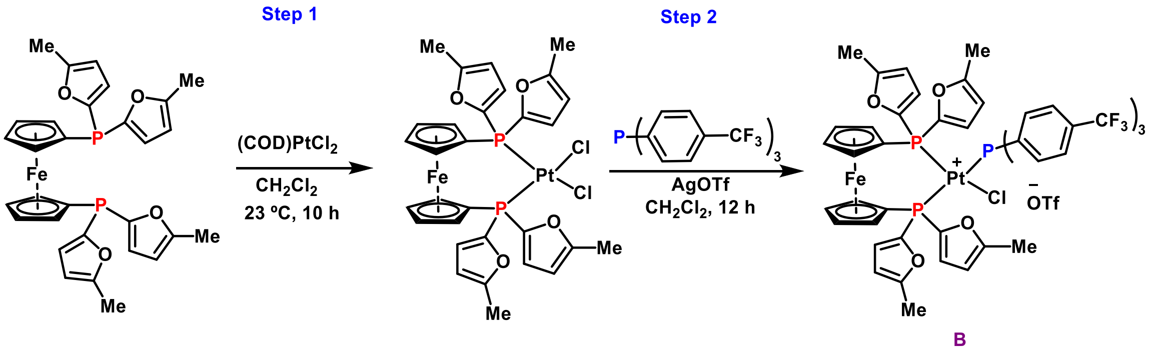

Yellow solid (143 mg, 85% yield).

^{1}H NMR (400 MHz, CDCl$_3$): δ 7.71 – 7.64 (m, 12H), 7.13 – 7.12 (m, 2H), 6.62 – 6.61 (m, 2H), 6.26 – 6.24 (m, 2H), 6.05 – 6.04 (m, 2H), 4.58 (d, J = 1.5 Hz, 1H), 4.51 (d, J = 1.5 Hz, 1H), 4.45 (dd, J = 4.0, 2.1 Hz, 1H), 4.21 (dd, J = 3.9, 2.0 Hz, 1H), 2.42 (s, 6H), 2.12 (s, 6H) ppm

^{13}C NMR (101 MHz, CDCl$_3$): δ 159.6 (dd, J = 55.6, 6.2 Hz), 139.0 (ddd, J = 75.1, 69.8, 3.1 Hz), 135.4 (d, J = 10.7 Hz), 133.7 (dq, J = 33.4, 3.0 Hz), 131.62 (d, J = 55.5 Hz), 126.7 (dd, J = 44.1, 18.2 Hz) 125.8 – 125.4 (m), 123.3 (q, J = 272.2 Hz), 121.2 (q, J = 322.3 Hz), 109.13 (dd, J = 83.4, 7.8 Hz), 75.6 – 75.1 (m), 14.3, 13.7 ppm

^{19}F NMR (565 MHz, CDCl$_3$): δ -63.3 (s), -78.0 (s) ppm

^{31}P NMR (162 MHz, CDCl$_3$): δ 22.8 (dd, $^2J_{P-P}$ = 452, 19 Hz, $^1J_{Pt-P}$ = 2508 Hz), -10.9 (dd, $^2J_{P-P}$ = 451, 17 Hz, $^1J_{Pt-P}$ = 2644 Hz), -24.1 (t, $^2J_{P-P}$ = 18 Hz, $^1J_{Pt-P}$ = 3656 Hz) ppm

HRMS (ESI+): calc'd for $C_{51}H_{40}ClF_9FeO_4FeP_3Pt$ [M-OTf]$^+$: 1266.0676, found 1266.0664.

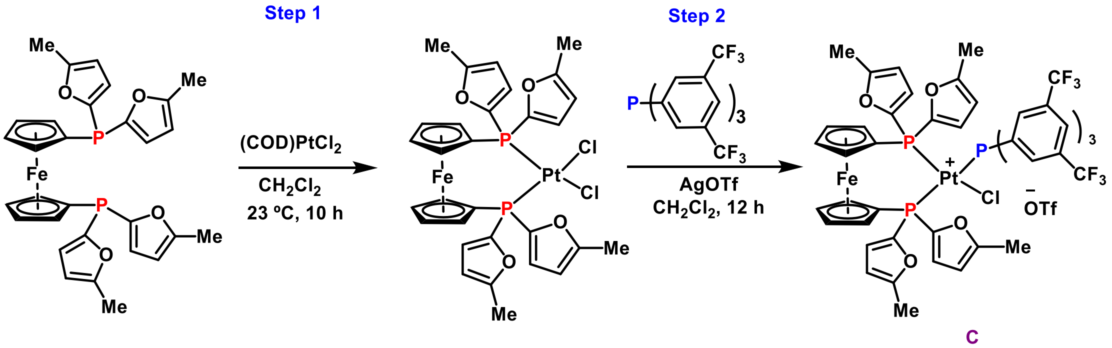

Yellow solid (192 mg, 99% yield).

^{1}H NMR (400 MHz, CDCl$_3$): δ 8.08 (s, 3H), 7.96 (s, 3H), 7.93 (s, 3H), 6.96 (s, 2H), 6.47 (s, 2H), 6.22 (s, 2H), 6.02 (s, 2H), 4.73 (s, 2H), 4.64 (s, 2H), 4.61 (s, 2H), 4.07 (s, 2H), 2.40 (s, 6H), 2.00 (s, 6H) ppm

^{13}C NMR (101 MHz, CDCl$_3$): δ 160.1 (dd, *J* = 64.8, 6.6 Hz), 138.6 (ddd, *J* = 65.9, 62.0, 3.0 Hz) 134.1 (d, *J* = 8.4 Hz), 132.9 (qd, *J* = 34.5, 11.3 Hz), 129.4 (dd, *J* = 56.5, 2.9 Hz), 126.9 – 126.4 (m), 122.3 (q, *J* = 273.6 Hz), 121.0 (q, *J* = 324.3 Hz), 109.5 (dd, *J* = 181.5, 7.4 Hz), 76.6 – 74.9 (m), 14.0, 13.4 ppm

^{31}P NMR (243 MHz, CDCl$_3$): δ 25.7 (dd, $^2J_{P-P}$ = 458, 19 Hz, $^1J_{Pt-P}$ = 2507 Hz), -10.2 (dd, $^2J_{P-P}$ = 459, 16 Hz, $^1J_{Pt-P}$ = 2760 Hz), -23.5 (t, $^2J_{P-P}$ = 18 Hz, $^1J_{Pt-P}$ = 3536 Hz) ppm

^{19}F NMR (565 MHz, CDCl$_3$): δ -63.2 (s), -78.2 (s) ppm

HRMS (ESI+): calc'd for $C_{54}H_{37}ClF_{18}FeO_4P_3Pt$ [M-OTf]$^+$: 1470.0297, found 1470.0295

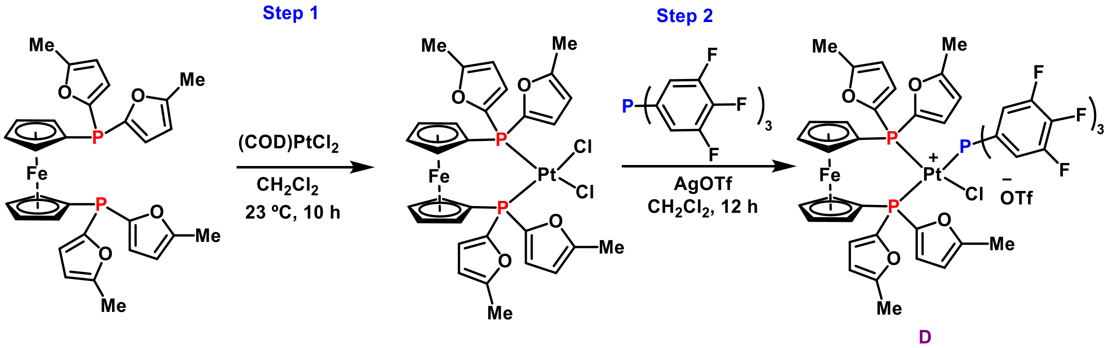

.

Yellow solid (140 mg, 84% yield).

Tris(3,4,5-trifluorophenyl)phosphane was prepared according to a literature procedure reported by Alexanian and coworkers.[1]

^{1}H NMR (400 MHz, CDCl$_3$): δ 7.09 – 7.08 (m, 2H), 7.05 – 7.01 (m, 6H), 6.95 – 6.94 (m, 2H), 6.28 – 6.28 (m, 2H), 6.24 – 6.24 (m, 2H), 4.60 (s, 2H), 4.55 (s, 2H), 4.46 – 4.45 (m, 2H), 4.22 (s, 2H), 2.47 (s, 6H), 2.39 (s, 6H) ppm

^{13}C NMR (101 MHz, CDCl$_3$): δ 160.2 (dd, *J* = 43.9, 6.5 Hz), 151.1 (dm, *J* =257.9 Hz), 142.3 (dt, *J* = 263.0, 14.8 Hz), 138.8 (ddd, *J* = 102.4, 13.8, 3.0 Hz), 126.3 (dd, *J* = 41.6, 17.6 Hz), 122.9 (d, *J* = 52.6 Hz), 120.5 – 118.2 (m), 109.2 (dd, *J* = 97.9, 7.7 Hz), 75.6 – 74.2 (m), 14.1, 13.8 ppm

^{31}P NMR (243 MHz, CDCl$_3$): δ 25.7 (dd, $^2J_{P-P}$ = 461, 17 Hz, $^1J_{Pt-P}$ = 2475 Hz), -10.3 (dd, $^2J_{P-P}$ = 461, 16 Hz, $^1J_{Pt-P}$ = 2747 Hz,), 24.4 (t, $^2J_{P-P}$ = 18 Hz, $^1J_{Pt-P}$ = 3642 Hz) ppm

^{19}F NMR (565 MHz, CDCl$_3$): δ -78.9, -129.9 (d, *J* = 19.3 Hz), -151.4 (t, *J* = 20.2 Hz) ppm

HRMS (ESI+): calc'd for $C_{48}H_{34}ClF_9FeO_4P_3Pt$ [M-OTf]$^+$: 1224.0206, found 1224.0201.

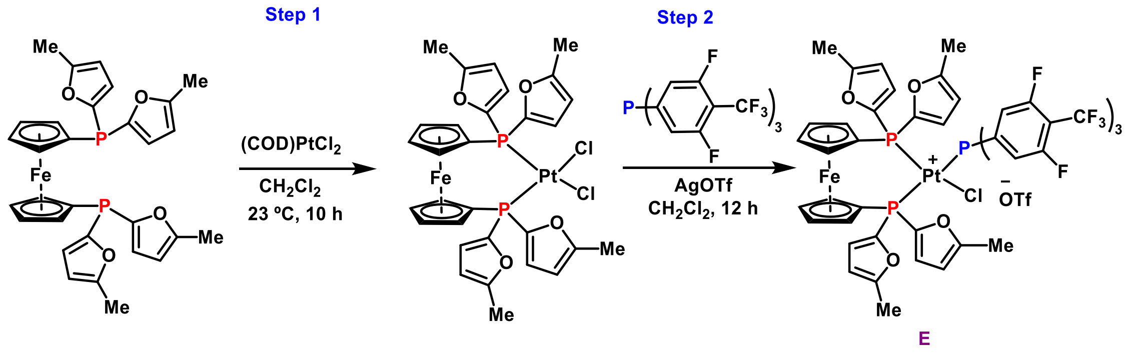

Yellow solid (150 mg, 82% yield).

^{1}H NMR (600 MHz, CDCl$_3$): ***δ*** 7.16 (dd, *J* = 11.8, 8.7 Hz, 6H), 7.13 – 7.09 (m, 2H), 6.93 – 6.86 (m, 2H), 6.29 (d, *J* = 3.1 Hz, 2H), 6.22 (d, *J* = 3.1 Hz, 2H), 4.58 (dd, *J* = 7.6, 1.3 Hz, 4H), 4.41 (dd, *J* = 3.8, 1.9 Hz, 2H), 4.32 (dd, *J* = 4.0, 2.1 Hz, 2H), 2.46 (s, 6H), 2.36 (s, 6H) ppm

^{13}C NMR (101 MHz, CDCl$_3$): *δ* 160.9 (d, *J* = 19.2 Hz), 160.6 (d, *J* = 6.1 Hz), 160.4 (d, *J* = 7.1 Hz), 158.3 (d, *J* = 20.2 Hz), 139.2 (dd, *J* = 37.8 Hz, *J* = 4.0 Hz), 138.1 (dd, *J* = 38.4 Hz, *J* = 3.0 Hz), 133.2 (d, *J* = 58.6 Hz), 126.9 (d, *J* = 17.2 Hz), 126.5 (d, *J* = 18.2 Hz), 120.8 (q, *J* = 275.7 Hz), 122.64, 119.5, 119.2 (d, *J* = 12.1 Hz), 119.0 (d, *J* = 11.1 Hz), 112.3-111.2 (m), 109.9 (d, *J* = 8.1 Hz), 108.9 (d, *J* = 7.1 Hz), 75.7 (d, *J* = 8.1 Hz), 75.6 (d, *J* = 13.1 Hz), 74.8 (d, *J* = 6.1 Hz), 71.6 (d, *J* = 90.9 Hz), 67.3 (d, *J* = 81.8 Hz), 14.2, 13.8 ppm

^{19}F NMR (376 MHz, CDCl$_3$): *δ* -56.8 – -56.9 (m), -57.0 (s), -78.2 – -78.3 (m), -105.4 (qd, *J* = 21.8, 4.0 Hz) ppm

^{31}P NMR (243 MHz, CDCl$_3$): *δ* 24.9 (ddd, $^{1}J_{Pt\text{-}P}$ = 3691 Hz, $^{2}J_{P\text{-}P}$ = 690 Hz, $^{2}J_{P\text{-}P}$ = 27 Hz), -10.3 (ddd, $^{1}J_{Pt\text{-}P}$ = 4238 Hz, $^{2}J_{P\text{-}P}$ = 690 Hz, $^{2}J_{P\text{-}P}$ = 24 Hz), -24.8 (dt, $^{1}J_{Pt\text{-}P}$ = 5448 Hz, $^{2}J_{P\text{-}P}$ = 24 Hz) ppm

HRMS (ESI+): calculated for $C_{51}H_{34}ClF_{15}FeO_4P_3Pt$ $[M\text{-}OTf]^+$: 1374.0111, found 1374.0126.

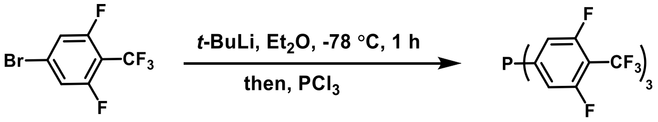

5-Bromo-1,3-difluoro-2-(trifluoromethyl)benzene (1.0 g, 3.8 mmol) was dissolved in 16 mL anhydrous ether, and cooled to -78 °C, *t*-BuLi (2.4 mL, 3.8 mmol) was added dropwise at this temperature. After stirred 1 h, freshly disted PCl_3 (83 μL, 0.95 mmol) was added dropwise, and then the mixture was stirred at 23 °C overnight. The solution was quenched with saturated NH_4Cl aqueous solution, and extracted with EtOAc (3 × 25 mL), dried over Na_2SO_4, filtered and concentrated under reduced pressure. The residue was purified by flash chromatography (EtOAc/hexane = 0/100 to 5/95) to afford the desired tris(3,5-difluoro-4-(trifluoromethyl)phenyl)phosphine (333 mg, 61% yield) as white solid.

^{1}H NMR (400 MHz, CDCl$_3$): *δ* 6.94 (t, *J* = 8.1 Hz, 6H) ppm

^{13}C NMR (151 MHz, CDCl$_3$): δ 160.4 (d, J = 263 Hz) , 141.6 – 141.4 (m), 121.24 (q, J = 274.8 Hz), 117.6 (td, J = 22.6 Hz, J = 4.5 Hz), 110.25 – 109.86 (m) ppm

^{19}F NMR (376 MHz, CDCl$_3$): δ -56.7 (t, J = 21.9 Hz), -107.2 (d, J = 22.1 Hz) ppm

^{31}P NMR (162 MHz, CDCl$_3$): δ -2.6 (s) ppm

HRMS (ESI-): calculated for $C_{21}H_6F_{15}PCl[M+Cl]^-$: 608.9651, found 608.9666.

3. Development of Reaction Condition

3.1 Evaluation of the Conditions for the Hydroalkoxylation of 1

General Procedure

In an argon filled glovebox, to a 4 mL vial with a magnetic stir bar were added the catalyst (0.0005mmol, 1 mol%), silver trifluoromethanesulfonate (0.3 mg, 0.001 mmol, 2 mol%), **1** (13.9 mg, 0.05 mmol, 1.0 equiv.), $ClCH_2CH_2Cl$ (1 mL) and 4,4'-di-*tert*-butyl-1,1'-biphenyl (0.5 equiv.) used as an internal standard. Then the vial was taken outside of the glovebox and the mixture was stirred at room temperature (23 ^oC) for 24 h. The mixture was diluted with CH_2Cl_2, filtered through a pad of celite and concentrated. The yield of **1a** was determined by crude ^{1}H NMR analysis.

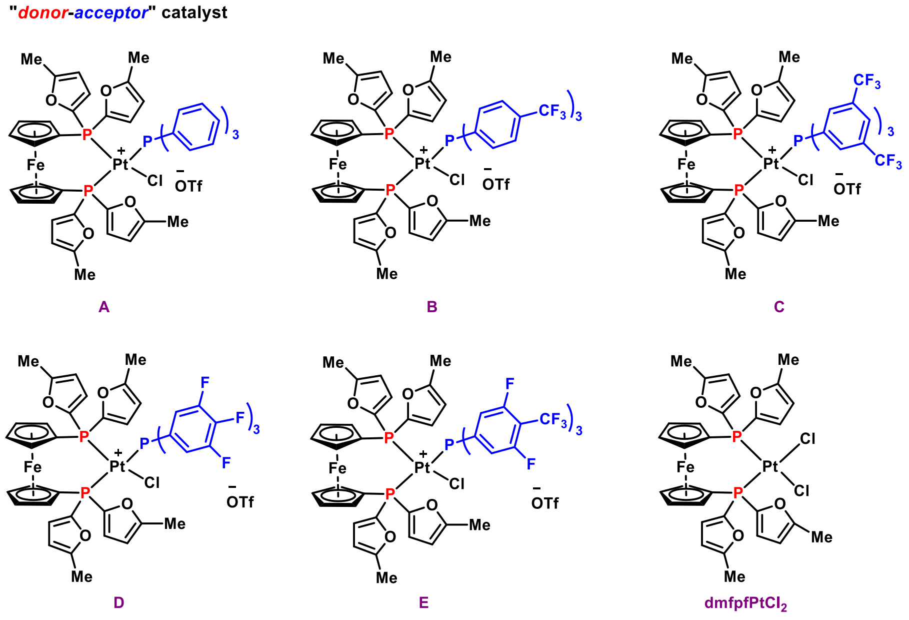

Supplementary Figure 1. Design of the Catalysts

Supplementary Table 1: Evaluation of the Conditions for the Hydroalkoxylation of **1**

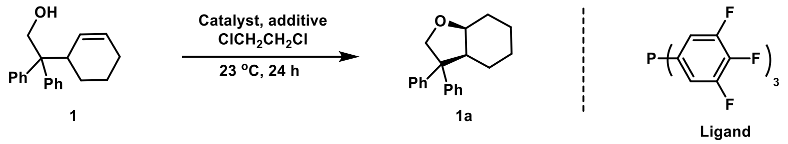

Entry	Catalyst	Additive	Yield (%)[a]	Entry	Catalyst	Additive	Yield (%)[a]
1	A (1 mol%)	AgOTf (1 mol%)	6	16	A (1 mol%)	AgOTf (2 mol%)	22
2	B (1 mol%)	AgOTf (1 mol%)	9	17	B (1 mol%)	AgOTf (2 mol%)	26
3	C (1 mol%)	AgOTf (1 mol%)	16	18	D (1 mol%)	AgOTf (2 mol%)	49
4	D (1 mol%)	AgOTf (1 mol%)	16	19	E (1 mol%)	AgOTf (2 mol%)	45
5	C (1 mol%)	AgOTf (2 mol%)	46	20	(dmfpf)$PtCl_2$ (2 mol%)	AgOTf (4 mol%)	37
7	C (1 mol%)	AgOTf (3 mol%)	48	21	(COD)$PtCl_2$ (2 mol%)	AgOTf (4 mol%)	17
6	C(1 mol%)	AgOTf (4 mol%)	50	22	D (2 mol%)	no additive	0
8	C (1 mol%)	AgOTf (5 mol%)	56	23	no catalyst	AgOTf (4 equiv.)	0
9	C (1 mol%)	AgOTf (6 mol%)	60	24[c]	no catalyst	AgOTf (4 mol%) Ligand (4 mol%)	0
10	C (1 mol%)	AgOTf (8 mol%)	63	25	(dmfpf)$PtCl_2$ (1 mol%)	$AgBF_4$ (2 mol%) Ligand (1mol%)	0
11	C (1 mol%)	AgOTf (10 mol%)	64	26	(dmfpf)$PtCl_2$ (1 mol%)	$AgPF_6$ (2 mol%) Ligand (1mol%)	0
12	C (1 mol%)	AgOTf (30 mol%)	60	27	C (2 mol%)	AgOTf (4 mol%)	90[b]
13	C (1 mol%)	AgOTf (50 mol%)	46	28	D (2 mol%)	AgOTf (4 mol%)	89[b]
14	C (1 mol%)	AgOTf (80 mol%)	32	30[d]	(dmfpf)$PtCl_2$ (1 mol%)	AgOTf (1 mol%)	0
15	C (1 mol%)	AgOTf (100 mol%)	25	31	(dmfpf)$PtCl_2$ (1 mol%)	AgOTf (1 mol%) Ligand (1mol%)	0

[a] Determined by ^{1}H NMR analysis using 4,4'-di-*tert*-butyl-1,1'biphenyl as an internal standard. [b] Catalyst (2 mol%), AgOTf (4 mol%), 24 h. [c] Complex **H** (in the proposed mechanism in the manuscript) was made, but no reactivity was observed, indicating that complex **H** is not the catalytic active specie. [d] Complex **F** (in the proposed mechanism in the manuscript) was made, but no reactivity was observed.

3.2 Evaluation of the Conditions for the Hydroamination of 2

General Procedure

In an argon filled glovebox, to a 4 mL vial with a magnetic stir bar were added the catalyst (0.005mmol, 5 mol%), silver trifluoromethanesulfonate (1.5 mg, 0.006 mmol, 6 mol%) and $ClCH_2CH_2Cl$ (0.5 mL). The mixture was stirred at 23 °C for 1 h. To the mixture was added a solution of **2** (30.7 mg, 0.1 mmol, 1.0 equiv) and 4,4'-di-*tert*-butyl-1,1'-biphenyl (13.3 mg, 0.05 mmol) in $ClCH_2CH_2Cl$ (0.5 mL). Then the vial was taken outside of the glovebox and the mixture was stirred at 50 °C for 48 h. The reaction mixture was diluted with CH_2Cl_2, filtered through a pad of celite and concentrated. The yield of **2a** was determined by crude ^{1}H NMR analysis.

Supplementary Table 2: Catalysts Screening by Intramolecular Hydroamination of **2**

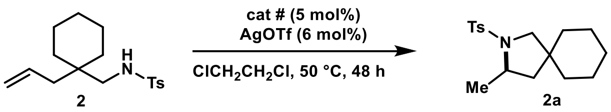

entry	cat #	additive	yield (%)[a]
1	E	AgOTf	62
2	A	AgOTf	23
3	B	AgOTf	28
4	C	AgOTf	44
5	D	AgOTf	46
6	none	AgOTf	n. r.
7	E	none	13

[a] Determined by ^{1}H NMR analysis using 4,4’-di-*tert*-butyl-1,1’biphenyl as an internal standard

3.3 The Effect of AgOTf on Catalytic Activity by Hydroalkoxylation of 1

Procedure: In an argon filled glovebox, to a 4 mL vial with a magnetic stir bar were added the catalyst (0.0005mmol, 1 mol%), silver trifluoromethanesulfonate, additive, $ClCH_2CH_2Cl$ (1 mL) and 4,4'-di-*tert*-butyl-1,1'-biphenyl (0.5 equiv.) used as an internal standard. The mixture was stirred for 1 h and **1** (13.9 mg, 0.05 mmol, 1.0 equiv.) was added to the above solution. Then the vial was taken outside of the glovebox and the mixture was stirred at room temperature (23 ^oC) for 24 h. The reaction mixture was diluted with CH_2Cl_2, filtered through a pad of celite and concentrated. The yield of **1a** was determined by crude ^{1}H NMR analysis.

Supplementary Table 3: The Effect of AgOTf on Catalytic Activity

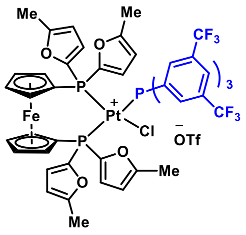

C

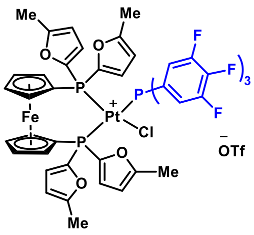

D

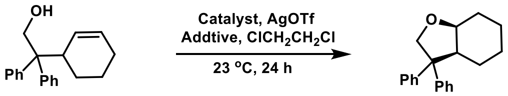

1 1a

Entry	Catalyst	AgOTf (mol%)	Addtive (mol%)	Yield (%)[a]	Entry	Catalyst	AgOTf (mol%)	Addtive (mol%)	Yield (%)[a]
1	C (1 mol%)	1	--	16	6	C (1 mol%)	100	--	25
2	C (1 mol%)	2	--	46	7	D (1 mol%)	2	--	49
3	C (1 mol%)	5	--	56	8	D (1 mol%)	2	KOTf(10 mol%)	13
4	C (1 mol%)	10	--	64	9	D (1 mol%)	2	KOTf(30 mol%)	7
5	C (1 mol%)	50	--	46	10	D (1 mol%)	2	KOTf(50 mol%)	4

[a] Determined by ^{1}H NMR analysis using 4,4'-di-*tert*-butyl-1,1'biphenyl as an internal standard.

3.4 The Effect of Monodentate Phosphine Ligand on the Catalytic Aactivity

Procedure: In an argon filled glovebox, to a 4 mL vial with a magnetic stir bar were added the catalyst **D** (0.0005mmol, 1 mol%), silver trifluoromethanesulfonate (0.004 mmol, 2 mol%), L, $ClCH_2CH_2Cl$ (1 mL) and 4,4'-di-*tert*-butyl-1,1'-biphenyl (0.5 equiv.) used as an internal standard. The mixture was stirred for 1 h and **1** (13.9 mg, 0.05 mmol, 1.0 equiv.) was added to the above solution. Then the vial was taken outside of the glovebox and the mixture was stirred at 23 °C for 24 h. The reaction mixture was diluted with CH_2Cl_2, filtered through a pad of celite and concentrated. The yield of **1a** was determined by crude ^{1}H NMR analysis.

Supplementary Table 4: The Effect of Monodentate Phosphine Ligand on the Catalytic Activity

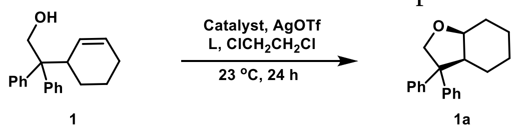

Entry	Catalyst	AgOTf (mol%)	L (mol%)	Yield (%)[a]
1	D (1 mol%)	2	--	49
2	D (1 mol%)	2	1	4
3	D (1 mol%)	2	2	0

[a] Determined by ^{1}H NMR analysis using 4,4'-di-*tert*-butyl-1,1'biphenyl as an internal standard.

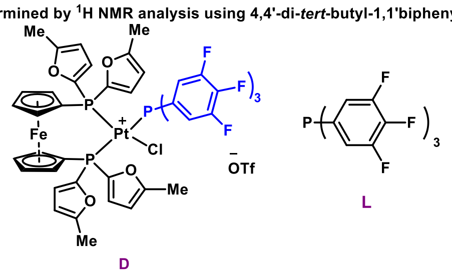

4. Synthesis of Substrates

4.1 Synthesis of Alcohols

Alcohols 2-(cyclohex-2-en-1-yl)-2,2-diphenylethan-1-ol(**1**), 5-methyl-2,2-diphenylhex-4-en-1-ol(**3**), 2,2-bis(2-methylallyl)propane-1,3-diol(**11**), 5-methyl-2,2-diphenylhex-5-en-1-ol(**14**), 2,2-diphenylpent-4-en-1-ol(**21**) were prepared according to a literature procedure reported by Widenhoefer and coworkers[2]. Alcohols (1-(3-methylbut-2-en-1-yl)cyclohexyl)methanol(**4**)[3], 4-methyl-2-phenylpent-4-en-1-ol(**9**)[27], 4-methyl-1-phenylpent-4-en-1-ol(**10**)[5], 2-(2-methylallyl)phenol(**13**)[6], (1-(2-methylallyl)cyclohexyl)methanol(**15**)[28], 2,2,4-triphenylpent-4-en-1-ol(**16**)[29], (4-methylenecyclohexyl)methanol(**17**)[9], 2-cinnamylphenol(**18**)[10], (E)-2,2,5-triphenylpent-4-en-1-ol(**19**)[11], (1-allylcyclohexyl)methanol(**22**)[4] were prepared according to literature procedures.

(1-(3-Methylbut-2-en-1-yl)cyclobutyl)methanol (5)

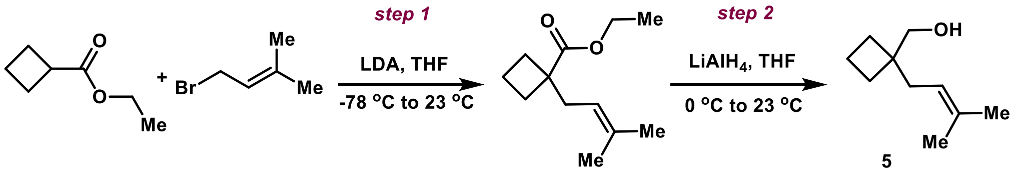

Step 1: A solution of ethyl cyclobutanecarboxylate (1.3 g, 10.0 mmol) in THF (5 mL) was added slowly to a solution of LDA [generated from diisopropylamine (1.2 g, 12.0 mmol) and *n*-BuLi (4.0 mL, 2.5 M in hexanes, 10.0 mmol) in THF (10 mL) at –78 °C] over 30 min and the resulting solution was stirred for additional 15 minutes. 1-Bromo-3-methylbut-2-ene (1.8 g, 12.0 mmol) was added over 10 minutes and the resulting mixture was warmed to room temperature (23 °C), stirred overnight, quenched with HCl (3 N, 10 mL), and extracted with ether (4 × 20 mL). The combined ether extracts were washed with water

(20 mL), dried over anhydrous Na_2SO_4 and concentrated under vacuum. Column chromatography of the residue (hexane/ethyl acetate = 40:1) gave ethyl 1-(3-methylbut-2-en-1-yl)-cyclobutane-1-carboxylate (1.4 g, 71%) as a viscous colorless oil.

Step 2: A solution of ethyl 1-(3-methylbut-2-en-1-yl)-cyclobutane-1-carboxylate (1.4 g, 7.0 mmol) in THF (15 mL) was added slowly to a suspension of $LiAlH_4$ (532 mg, 14.0 mmol) in THF (10 mL) at 0 ^oC. The resulting mixture was stirred at room temperature (23 ^oC) overnight, quenched by sequential addition of water (0.5 mL) and aqueous NaOH (15%, 0.5 mL) and additional water (1.5 mL) at 0 ^oC. The resulting suspension was filtered through celite and eluted with ether. The ether eluant was dried over anhydrous Na_2SO_4 and concentrated under vacuum. Chromatography of the residue (hexane/ethyl acetate = 20:1) gave 2,2-diphenyl-4-penten-1-ol **5** (864 mg, 80%) as a colorless oil.

^{1}H NMR (400 MHz, $CDCl_3$): δ 5.15 (tt, *J* = 7.4, 1.3 Hz, 1H), 3.50 (s, 2H), 2.18 (d, *J* = 7.4 Hz, 2H), 1.90 – 1.73 (m, 6H), 1.71 (s, 3H), 1.64 (s, 3H) ppm

^{13}C NMR (101 MHz, $CDCl_3$): δ 133.6, 120.6, 69.0, 43.5, 35.4, 28.0, 26.1, 18.1, 15.4 ppm

HRMS (ESI+): calc'd for $C_{10}H_{19}O$ $[M+H]^+$: 155.1430, found 155.1430.

6-Methyl-2,2-diphenylhept-5-en-1-ol (6)

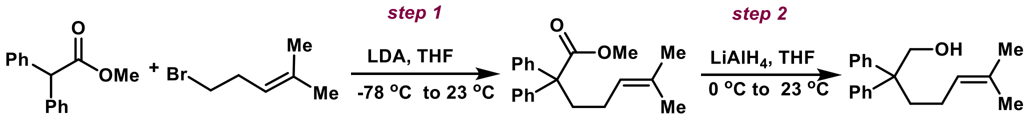

Step 1: A solution of ethyl 2,2-diphenylacetate (2.3 g, 10.0 mmol) in THF (5 mL) was added slowly to a solution of LDA [generated from diisopropylamine (1.5 g, 15.0 mmol) and *n*-BuLi (4.8 mL, 2.5 M in hexanes, 12.0 mmol) in THF (10 mL) at –78 ^oC] over 30 min and the resulting solution was stirred for additional 15 min. 5-Bromo-2-methyl-2-pentene (2.0 g, 12.0 mmol) was added over 10 min and the resulting mixture was warmed to room temperature (23 ^oC), stirred overnight, quenched with HCl (3 N, 10 mL), and extracted with ether (4 × 20 mL). The combined ether extracts were washed with water (20 mL), dried over anhydrous Na_2SO_4, and concentrated under vacuum. Column chromatography of the residue (hexane/ethyl acetate = 40:1) gave ethyl 6-methyl-2,2-diphenylhept-5-enoate (1.8 g, 59%) as a viscous colorless oil.

Step 2: A solution of ethyl 6-methyl-2,2-diphenylhept-5-enoate (1.8 g, 5.8 mmol) in THF (15 mL) was added slowly to a suspension of $LiAlH_4$ (440 mg, 11.6 mmol) in THF (10 mL) at 0 ^oC. The resulting mixture was stirred at room temperature (23 ^oC) overnight, quenched by sequential addition of water (0.5 mL) and aqueous NaOH (15%, 0.5 mL) and additional water (1.5 mL) at 0 ^oC. The resulting suspension was filtered through celite and eluted with ether. The ether eluant was dried over anhydrous Na_2SO_4, and

concentrated under vacuum. Chromatography of the residue (hexane/ethyl acetate = 20:1) gave 6-methyl-2,2-diphenylhept-5-en-1-ol **6** (1.2 g, 73%) as a colorless oil.

^{1}H NMR (400 MHz, CDCl$_3$): δ 7.32 – 7.29 (m, 4H), 7.24 – 7.19 (m, 6H), 5.10 (tt, *J* = 7.1, 1.2 Hz, 1H), 4.18 (d, *J* = 6.9 Hz, 2H), 2.19 – 2.15 (m, 2H), 1.72 (q, *J* = 7.6 Hz, 2H), 1.65 (s, 3H), 1.44 (s, 3H), 1.12 (t, *J* = 6.9 Hz, 1H) ppm

^{13}C NMR (151 MHz, CDCl$_3$): δ 145.7, 131.9, 128.4, 128.3, 126.4, 124.4, 68.2, 52.2, 36.6, 25.8, 23.1, 17.6 ppm

HRMS (ESI+): calc'd for $C_{20}H_{25}O$ $[M+H]^+$: 281.1900, found 281.1901.

Ethyl -2-hydroxy-2-methyl-1-(3-methylbut-2-en-1-yl)cyclohexane-1-carboxylate (7)

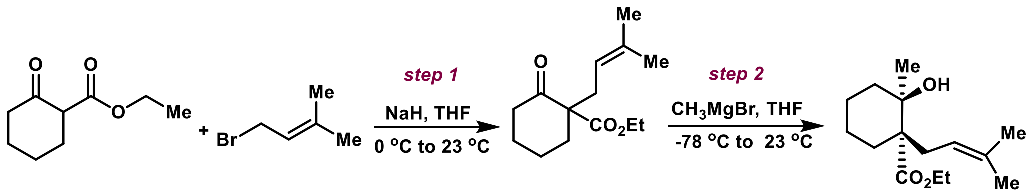

Step 1: To a suspension of NaH (880 mg, 22.0 mmol) in THF (30 mL) at 0 °C under argon was added dropwise a solution of ethyl 2-oxocyclohexane-1-carboxylate (3.4 g, 20.0 mmol) in THF (20 mL). After stirring 30 min, a solution of 1-bromo-3-methylbut-2-ene (3.3 g, 22.0 mmol) in THF (10 mL) was added. The resulting mixture was warmed to room temperature (23 °C) and stirred overnight. The mixture was quenched with aqueous NH_4Cl (50 mL) and extracted with ether (2 x 60 mL). The combined organic layers were dried over anhydrous Na_2SO_4, and concentrated under vacuum. The product was purified by column chromatography (hexane/ethyl acetate = 40:1) to yield 1.9 g of compound ethyl 1-(3-methylbut-2-en-1-yl)-2-oxocyclohexane-1-carboxylate (40% yield) as a colorless oil.

Step 2: To a suspension of ethyl 1-(3-methylbut-2-en-1-yl)-2-oxocyclohexane-1-carboxylate (263 mg, 1.1 mmol) in THF (5 mL) at -78 °C under argon was added dropwise a solution of CH_3MgBr in THF (1.2 mL, 1M, 1.2 mmol). The resulting mixture was warmed to room temperature (23 °C) and stirred for 2 h. The reaction was quenched with aqueous NH_4Cl (50 mL) and extracted with ether (2 x 60 mL). The combined organic layers were dried over anhydrous Na_2SO_4, and concentrated under vacuum. The product was purified by column chromatography (hexane/ethyl acetate = 20:1) yield 203 mg of compound ethyl -2-hydroxy-2-methyl-1-(3-methylbut-2-en-1-yl)cyclohexane-1-carboxylate **7** (73% yield) as a colorless oil.

^{1}H NMR (400 MHz, CDCl$_3$): δ 4.90 – 4.85 (m, 1H), 4.22 – 4.07 (m, 2H), 3.47 (bs, 1H), 2.75 (dd, *J* = 14.8, 7.1 Hz, 1H), 2.39 (dd, *J* = 14.8, 7.7 Hz, 1H), 1.83 – 1.50 (m, 12H), 1.34 – 1.19 (m, 8H) ppm

^{13}C NMR (101 MHz, CDCl$_3$): δ 177.3, 134.4, 119.5, 72.8, 60.7, 54.2, 34.8, 27.8, 27.0, 26.1, 25.4, 22.8, 20.8, 18.1, 14.4 ppm

HRMS (ESI+): calc'd for $C_{15}H_{27}O_3$ $[M+H]^+$: 255.1955, found 255.1953.

4-Cyclobutylidenebutan-1-ol (8)

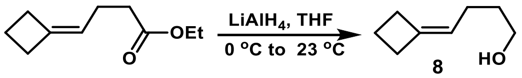

Ethyl 4-cyclobutylidenebutanoate was prepared according to a literature procedure reported by Shenvi and coworkers.[16] A solution of ethyl 4-cyclobutylidenebutanoate (2.0 mmol, 336 mg) in anhydrous THF (5 mL) was added dropwise to a suspension of $LiAlH_4$ (4.0 mmol, 152 mg) in anhydrous THF (5 mL) at 0 °C. The resulting mixture was warmed to room temperature (23 °C) and stirred for 2 h, the reaction mixture was cooled to 0 °C, diluted with Et_2O (10 mL), quenched by the sequential addition of water (0.15 mL), a 15% aqueous NaOH solution (0.15 mL) and additional water (0.45 mL). The resulting white precipitate was filtered through a pad of celite and washed with Et_2O. The filtrate was dried over anhydrous Na_2SO_4, filtered and concentrated under vacuum. Purification by flash chromatography (hexane/ethyl acetate = 20:1) yielded 4-Cyclobutylidenebutan-1-ol **8** (215 mg, 85 %) as a colorless oil.

^{1}H NMR (400 MHz, $CDCl_3$): δ 5.08 – 5.02 (m, 1H), 3.62 (t, *J* = 6.5 Hz, 2H), 2.64 – 2.60 (m, 4H), 1.97 – 1.88 (m, 4H), 1.62 – 1.55 (m, 2H) ppm

^{13}C NMR (101 MHz, $CDCl_3$): δ 140.8, 119.7, 62.8, 32.7, 31.0, 29.3, 24.5, 17.1 ppm

HRMS (ESI+): calc'd for $C_8H_{15}O$ $[M+H]^+$: 127.1117, found 127.118.

2-(Hydroxymethyl)-2-(2-methylallyl)cyclohexan-1-one (12)

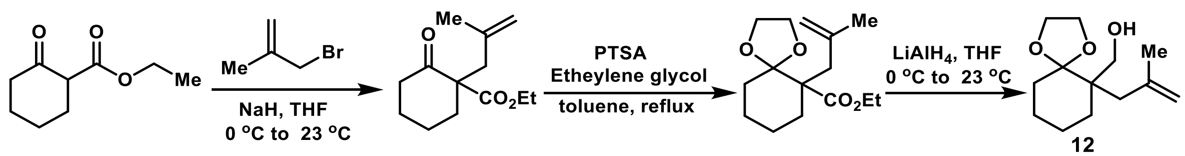

Step 1: To a suspension of NaH (230 mg, 9.6 mmol) in THF (15 mL) at 0 °C under argon was added dropwise a solution of ethyl 2-oxocyclohexane-1-carboxylate (1.4 g, 8.0 mmol) in THF (10 mL). After 30 min, a solution of 3-Bromo-2-methylpropene (1.3 g, 9.6 mmol) in THF (5 mL) was added. The resulting mixture was warmed to room temperature (23 °C) and stirred overnight. The reaction was quenched with aqueous NH_4Cl (25 mL) and extracted with ether (2 x 50 mL). The combined organic layers were dried with anhydrous Na_2SO_4, filtered, and concentrated under vacuum. The product was purified by column chromatography (hexane/ethyl acetate = 20:1) to yield 724 mg of compound ethyl 1-(2-methylallyl)-2-oxocyclohexane-1-carboxylate (40% yield) as a colorless oil.

Step 2: According to a procedure by Brimble and coworkers.[7] A mixture of ethyl 1-(2-methylallyl)-2-oxocyclohexane-1-carboxylate (519 mg, 2.3 mmol), ethylene glycol (2 mL, 35.0 mmol), and *p*-toluenesulfonic acid monohydrate (7.9 mg, 2 mol%) in dry benzene (15 mL) was refluxed under argon for 8 h with azeotropic removal of water. The reaction mixture was diluted with ethyl ether (20 mL), washed

with saturated aqueous $NaHCO_3$ and brine, dried over anhydrous Na_2SO_4, and concentrated in vacuo to afford a crude product as a colorless oil. The crude product was used in the next step without further purification.

Step 3: A solution of ethyl 6-(2-methylallyl)-1,4-dioxaspiro[4.5]decane-6-carboxylate (1.8 mmol, 481 mg) in anhydrous THF (5 mL) was added dropwise to a suspension of $LiAlH_4$ (3.6 mmol, 137 mg) in anhydrous THF (5 mL) at 0 °C. The resulting mixture was warmed to room temperature (23 °C) and stirred for 2 h, the reaction mixture was cooled to 0 °C, diluted with Et_2O (10 mL), quenched by the sequential addition of water (0.14 mL), a 15 % aqueous NaOH solution (0.14 mL) and additional water (0.42 mL). The resulting white precipitate was filtered through a pad of celite and washed with Et_2O. The filtrate was dried over anhydrous Na_2SO_4, filtered and concentrated under vacuum. Purification by flash chromatography (hexane/ethyl acetate = 20:1) yielded 2-(hydroxymethyl)-2-(2-methylallyl)cyclohexan-1-one **12** (220 mg, 54 %) as a colorless oil.

^{1}H NMR (400 MHz, $CDCl_3$): δ 4.90 (s, 1H), 4.78 (s, 1H), 4.01 – 3.92 (m, 4H), 3.85 (d, *J* = 11.4 Hz, 1H), 3.42 (dd, *J* = 11.4, 8.4 Hz, 1H), 3.06 (dd, *J* = 8.4, 2.4 Hz, 1H), 2.41 (d, *J* = 13.1 Hz, 1H), 2.18 (d, *J* = 13.1 Hz, 1H), 1.81 (s, 3H), 1.69 – 1.38 (m, 8H) ppm

^{13}C NMR (101 MHz, $CDCl_3$): δ 142.6, 115.4, 113.8, 65.0, 64.8, 64.2, 45.3, 36.2, 30.5, 30.2, 25.7, 23.4, 20.6 ppm

HRMS (ESI+): calc'd for $C_{13}H_{23}O_3$ $[M+H]^+$: 227.1642, found 227.1640.

(Z)-2,2,5-triphenylpent-4-en-1-ol [(*Z*)-20]

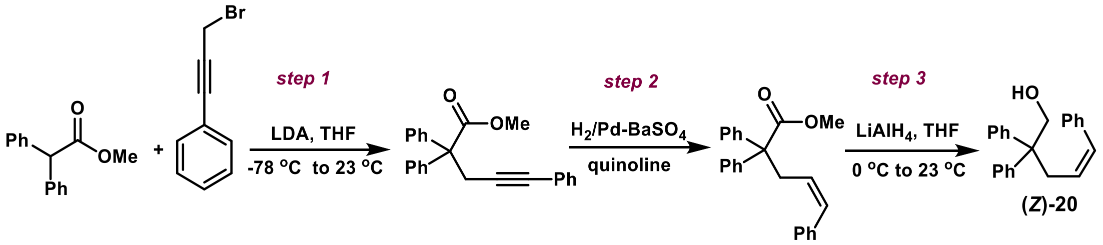

(3-bromoprop-1-yn-1-yl)benzene was synthesized from reported procedure and the analytical data was consistent with the literature.[8]

Step 1: A solution of methyl 2,2-diphenylacetate (2.3 g, 10.0 mmol) in THF (5 mL) was added slowly to a solution of LDA [generated from diisopropylamine (1.5 g, 15.0 mmol) and *n*-BuLi (4.8 mL, 2.5 M in hexanes, 12.0 mmol) in THF (10 mL) at –78 °C] over 30 min and the resulting solution was stirred for additional 15 min. (3-bromoprop-1-yn-1-yl)benzene (2.0 g, 10.0 mmol) was added over 10 min and the resulting mixture was warmed to room temperature (23 °C), stirred overnight, quenched with HCl (3 N, 10 mL), and extracted with ether (4 × 20 mL). The combined ether extracts were washed with water (20 mL), dried by anhydrous Na_2SO_4, and concentrated under vacuum. Column chromatography of the

residue (hexane/ethyl acetate = 40:1) gave methyl 2,2,5-triphenylpent-4-ynoate (3.2 g, 94%) as a yellow oil.

Step 2: To a stirred solution of methyl 2,2,5-triphenylpent-4-ynoate (681 mg, 2.0 mmol) in quinoline (2 mL) was added 5% Pd-$BaSO_4$ (424 mg, 0.2 mmol). The reaction mixture was stirred under H_2 balloon for 12 h at room temperature (23 ^oC). The resulting mixture was filtered through a celite pad and concentrated in vacuo. Methyl (Z)-2,2,5-triphenylpent-4-enoate (479 mg, 70 %) was obtained as a yellowish oil and used for next step without further purification.

Step 3: A solution of methyl (Z)-2,2,5-triphenylpent-4-enoate (213 g, 0.6 mmol) in THF (2 mL) was added slowly to a suspension of $LiAlH_4$ (45 mg, 1.2 mmol) in THF (2 mL) at 0 ^oC. The resulting mixture was stirred at room temperature (23 ^oC) overnight, quenched by sequential addition of water (0.05 mL) and aqueous NaOH (15%, 0.05 mL) and additional water (0.15 mL) at 0 ^oC. The resulting suspension was filtered through celite and eluted with ether. The ether eluant was dried by anhydrous Na_2SO_4 and concentrated under vacuum. Chromatography of the residue (hexane/ethyl acetate = 20:1) gave (*Z*)-2,2,5-triphenylpent-4-en-1-ol **(*Z*)-20** (131 mg, 70%) as a yellow oil.

^{1}H NMR (600 MHz, $CDCl_3$): δ 7.34 – 7.19 (m, 15H), 6.42 (d, *J* = 11.8 Hz, 1H), 5.43 (dt, *J* = 11.8, 6.9 Hz, 1H), 4.14 (d, *J* = 6.5 Hz, 2H), 3.24 (dd, *J* = 6.9, 2.0 Hz, 2H) 1.14 (t, *J* = 6.8 Hz, 1H) ppm

^{13}C NMR (151 MHz, $CDCl_3$): δ 145.1, 137.6, 131.3, 128.9, 128.5, 128.41, 128.4, 128.3, 126.9, 126.6, 68.8, 52.4, 35.4 ppm

4.2 Synthesis of Alkenes

Norbornylene(**23**), Comphene(**24**), 1-methylcyclohex-1-ene(**43**) and 1-methylcyclopent-1-ene (**44**), styrene(**48**), (Z)-cyclooctene(**49**) were purchased from commercial sources.

Alkenes ethyl 4-cyclobutylidenebutanoate(**25**)[16], 3,7-dimethyloct-6-en-1-yl benzoate(**26**)[14]**,** (5-methylhex-5-en-1-yl)benzene(**27**)[15] were prepared according to literature procedures.

4.3 Synthesis of Amines

_N_-((1-allylcyclohexyl)methyl)-4-methylbenzenesulfonamide (2)

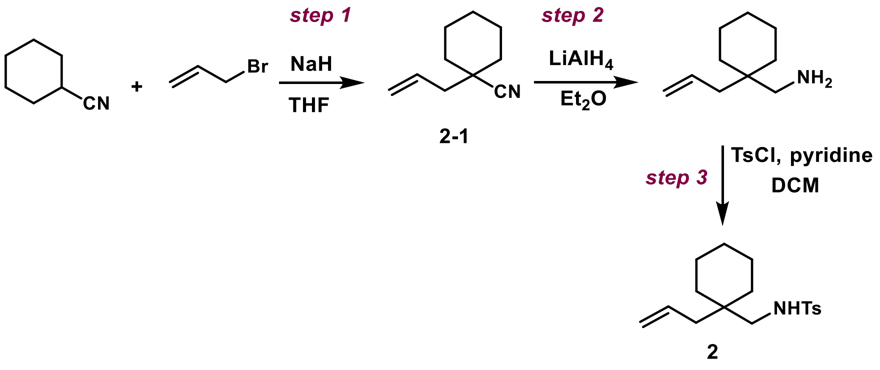

Step 1: The above alkene-substituted nitrile **2-1** was prepared by following a reported procedure.[18] To a

freshly prepared LDA (from 21.0 mmol *n*-BuLi and 24.0 mmol diisopropyl amine at 0 °C for 30 min) in THF (40 mL) was added nitrile (20.0 mmol, 1.0 equiv.) dropwise at -78 °C. The resulting mixture was stirred at the same temperature for 30 min. Allyl bromide (28.0 mmol, 1.4 equiv.) was added to the reaction mixture dropwise before allowing the reaction mixture to warm up to 23 °C. After stirring overnight, the reaction mixture was quenched with water at 0 °C and extracted with EtOAc. The combined extracts were washed with brine, dried over $MgSO_4$, and concentrated. The crude product was used in the next step directerly without further purification.

Step 2: The procedure analogous to that used to synthesize **31-2**

Step 3: The procedure analogous to that used to synthesize **29-1**.

^{1}H NMR (400 MHz, $CDCl_3$): δ 7.76 (d, J = 8.3 Hz, 2H), 7.30 (d, J = 8.0 Hz, 2H), 5.70 (ddt, J = 17.7, 10.2, 7.5 Hz, 1H), 5.07 – 4.94 (m, 2H), 4.90 (t, J = 6.8 Hz, 1H), 2.73 (d, J = 6.9 Hz, 2H), 2.42 (s, 3H), 2.04 (d, J = 7.5 Hz, 2H), 1.48 – 1.20 (m, 10H) ppm

^{13}C NMR (101 MHz, $CDCl_3$): δ 143.3, 137.1, 134.2, 129.7, 127.2, 117.9, 49.5, 40.4, 36.4, 33.3, 26.1, 21.6, 21.3 ppm

HRMS (ESI+): calculated for $C_{17}H_{26}NO_2S[M+H]^+$: 308.1679, found 308.1680.

4-Methyl-N-(4-methylpent-4-en-1-yl)benzenesulfonamide (28)

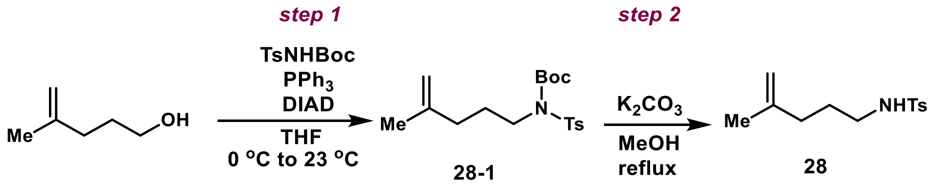

Step 1: The above compounds 4-methyl-N-(4-methylpent-4-en-1-yl)benzenesulfonamide was prepared by following a reported procedure.[12] A mixture of Ts-NH–Boc (1.63 g, 6.0 mmol, 1.2 equiv.), PPh_3 (1.70 g, 6.5 mmol, 1.3 equiv) and alcohol (4.52 g, 5 mmol, 1.0 equiv.) in THF (25 mL) was cooled to 0 °C and DIAD (1.28 mL, 6.5 mmol, 1.3 equiv.) was added dropwise. After stirring for 5 min at 0 °C, the mixture was allowed to warm up to 23 °C and stirred overnight. The solvent was removed and the residue was purified by flash column chromatography on silica gel (hexanes/EtOAc 20:1–10:1) to give **28-1** as white solid (1.59 g, 90%).

^{1}H NMR (600 MHz, $CDCl_3$): δ 7.77 (d, J = 8.2 Hz, 2H), 7.29 (d, J = 8.2 Hz, 2H), 4.74 (d, J = 6.7 Hz, 2H), 3.87 – 3.67 (m, 2H), 2.43 (s, 3H), 2.08 (t, J = 7.5 Hz, 2H), 1.97 – 1.81 (m, 2H), 1.75 (s, 3H), 1.33 (s, 9H) ppm

^{13}C NMR (151 MHz, $CDCl_3$): δ 151.1, 144.7, 144.2, 137.7, 129.4, 127.9, 110.6, 84.2, 47.1, 35.0, 28.0, 22.5, 21.7 ppm

HRMS (ESI+): calculated for $C_{18}H_{27}NO_4SNa[M+Na]^+$: 376.1553, found 376.1555.

Step 2: To a solution of **28-1** (1.41g, 4.0 mmol, 1.0 equiv.) in methanol (20 mL) was added K_2CO_3 (2.76g, 20.0 mmol, 5.0 equiv.). After refluxing for 5 h, water (50 mL) was introduced and the aqueous layer was extracted with CH_2Cl_2. The organic layer was washed with brine, dried over Na_2SO_4 and concentrated to give **28** (0.99 g, 98%) as a colorless oil.

^{1}H NMR (600 MHz, $CDCl_3$) δ 7.74 (d, J = 8.1 Hz, 2H), 7.31 (d, J = 7.9 Hz, 2H), 4.69 (s, 1H), 4.61 (s, 1H), 4.52 – 4.42 (m, 1H), 3.00 – 2.90 (m, 2H), 2.43 (s, 3H), 1.99 (t, J = 7.5 Hz, 2H), 1.65 (s, 3H), 1.64 – 1.56 (m, 3H) ppm

^{13}C NMR (151 MHz, $CDCl_3$) δ 144.6, 143.5, 137.2, 129.8, 127.2, 110.9, 43.0, 34.8, 27.6, 22.3, 21.7 ppm

HRMS (ESI+): calculated for $C_{13}H_{20}NO_2S[M+H]^+$: 254.1209, found 254.1210.

4-Methyl-*N*-(2-(2-methylallyl)phenyl)benzenesulfonamide (29)

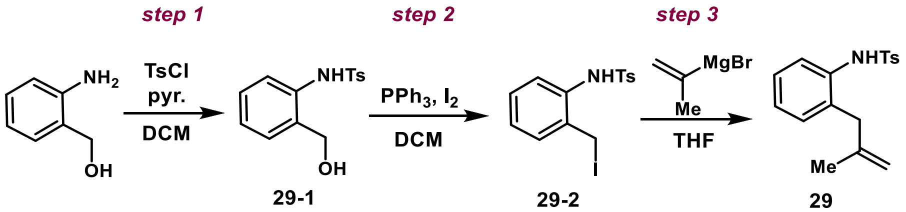

Step 1: The above compounds **29-1** was prepared by following reported procedures.[13] To a solution of anilines (15.0 mmol, 1.0 equiv.) in anhydrous DCM (30 mL) was added *p*-toluenesulfonyl chloride (16.5 mmol, 1.1 equiv.) and pyridine (45.0 mmol, 3.0 equiv.). Then the mixture was stirred at 23 °C overnight. After completion of the reaction (monitored by TLC), the reaction mixture was diluted with 40 mL H_2O and extracted with Et_2O (3 × 50 mL). The combined organic layers were washed with brine, dried over $MgSO_4$, and concentrated in vacuo. The residue was purified by flash chromatography (hexanes/EtOAc = 30:1-10:1) to afford the desired sulfonamide.

Step 2: To a solution of triphenylphosphine (10.3 mmol, 1.03 equiv.) in dry dichloromethane (30 mL) was added iodine (10.3 mmol, 1.03 equiv.). After stirring for 5 min, alcohol (10 mmol, 1.0 equiv.) was added to the above mixture. After complete conversion of the alcohol (monitored by TLC), the reaction was quenched with an aqueous solution of sodium thiosulfate (20 mL). The organic layers were removed and the aqueous solution extracted with EtOAc (50 mL). The combined organic layers were dried using sodium sulfate, filtered and concentrated. The residue was purified by column chromatography (hexanes/EtOAc = 10:1) to get the desired iodide product.

Step 3: To a solution of *N*-(2-(iodomethyl)phenyl)-4-methylbenzenesulfonamide (2.0 mmol, 1.0 equiv.) in anhydrious THF (10.0 mL) at 0 °C was added prop-1-en-2-ylmagnesium bromide (6.0 mmol, 3.0 equiv.) dropwise. Then the reaction mixture was allowed to warm up to 23 °C and stirred for 12 h. After completion of the reaction (monitored by TLC), the reaction was quenched with saturated NH_4Cl aqueous solution and diluted with 40 mL H_2O and extracted with EtOAc (3 × 50 mL). The combined organic layers

were washed with brine, dried over $MgSO_4$, and concentrated in vacuo. The residue was purified by flash chromatography (hexanes/EtOAc = 30:1-10:1) to afford the desired sulfonamide at high yield.

^{1}H NMR (400 MHz, $CDCl_3$): δ 7.66 – 7.54 (m, 2H), 7.46 (dd, *J* = 8.0, 0.9 Hz, 1H), 7.20 (dd, *J* = 9.6, 4.8 Hz, 3H), 7.09 (td, *J* = 7.4, 1.2 Hz, 1H), 7.03 (dd, *J* = 7.5, 1.4 Hz, 1H), 6.72 (s, 1H), 4.88 (s, 1H), 4.61 (s, 1H), 2.92 (s, 2H), 2.38 (s, 3H), 1.57 (s, 3H) ppm

^{13}C NMR (101 MHz, $CDCl_3$): δ 143.9, 143.7, 137.0, 135.6, 131.4, 131.1, 129.7, 127.9, 127.2, 126.1, 124.2, 113.0, 77.5, 77.2, 76.8, 41.0, 22.2, 21.7 ppm

HRMS (ESI+): calculated for $C_{17}H_{20}NO_2S$ $[M+H]^+$: 301.1209, found 302.1212.

4-Methyl-*N*-(2-(2-methylenecyclohexyl)phenyl)benzenesulfonamide (30)

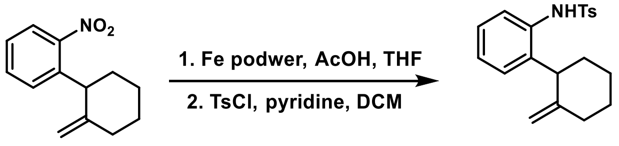

The compound **30a-1** was prepared by following reported procedures.[17] The spectroscopic data was in accordance with those reported in literature. And the procedure of sulfonation of amines was the same with the procedure of synthesis of **29-1**.

^{1}H NMR (400 MHz, $CDCl_3$): δ 7.55 (d, *J* = 8.3 Hz, 2H), 7.48 (dd, *J* = 7.7, 1.6 Hz, 1H), 7.28 – 7.15 (m, 4H), 7.09 (dd, *J* = 7.5, 1.7 Hz, 1H), 6.34 (s, 1H), 4.63 (d, *J* = 1.4 Hz, 1H), 3.86 (d, *J* = 1.3 Hz, 1H), 2.50 (dd, *J* = 12.0, 3.2 Hz, 1H), 2.39 (s, 4H), 2.00 (td, *J* = 13.1, 4.0 Hz, 1H), 1.90 – 1.75 (m, 2H), 1.58 (qd, *J* = 12.3, 3.4 Hz, 1H), 1.52 – 1.42 (m, 1H), 1.31 (qt, *J* = 13.1, 3.8 Hz, 1H), 1.21 – 1.10 (m, 1H) ppm

^{13}C NMR (101 MHz, $CDCl_3$): δ 151.2, 144.0, 137.3, 137.0, 134.7, 129.9, 127.7, 127.6, 127.3, 127.0, 126.3, 108.6, 44.7, 36.9, 33.0, 28.6, 26.8, 21.8 ppm

HRMS (ESI+): calculated for $C_{20}H_{24}NO_2S$ $[M+H]^+$: 342.1522, found 342.1525.

4-methyl-*N*-((1-(2-phenylallyl)cyclohexyl)methyl)benzenesulfonamide (31)

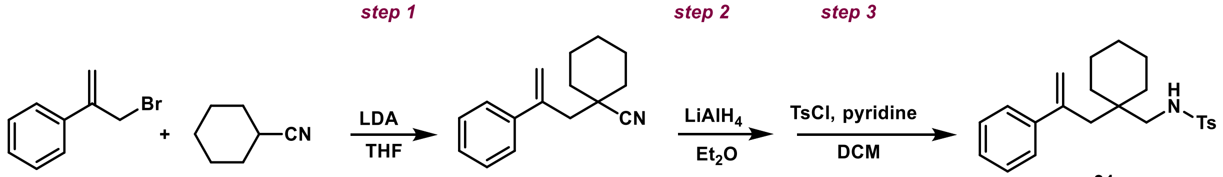

Step 1: Compound **31-1** was prepared following a reported procedure.[18] To a freshly prepared LDA (from 21.0 mmol *n*-BuLi and 24.0 mmol diisopropyl amine at 0 °C for 30 min) in THF (40 mL) was added nitrile (20.0 mmol, 1.0 equiv.) dropwise at -78 °C. The resulting mixture was stirred at the same temperature for 30 min. Allyl bromide (28.0 mmol, 1.4 equiv.) was added to the reaction mixture dropwise before allowing the reaction mixture to warm up to 23 °C. After stirring for 12 h, the reaction mixture was quenched with

water at 0 °C and extracted with EtOAc. The combined extracts were washed with brine, dried over $MgSO_4$, and concentrated. The crude product was used in the next step directerly without further purification.

Step 2: To a suspension of $LiAlH_4$ (36 mmol, 2.0 equiv.) in anhydrous Et_2O (30 mL) was added alkene-substituted acetonitrile (18 mmol, 1.0 equiv.) in anhydrous Et_2O (10 mL) dropwise at 0 °C. Then the reaction mixture was allowed to warm to 23 °C slowly and stired for 16 h. After completion of the reaction (monitored by TLC), the reaction was quenched by the sequential addition of H_2O (1.5 mL), 15 % NaOH aqueous solution (3.0 mL), and H_2O (1.5 mL) and stired for another 30 min. The resulting white precipitate was filtered through a pad of celite and washed with Et_2O. The filtrate was dried over $MgSO_4$, filtered and concentrated under reduced pressure. The crude product was used in the next step directerly without further purification.

Step 3: The above compound was prepared by following a reported procedure.[13] To a solution of amines (15.0 mmol, 1.0 equiv.) in anhydrous DCM (30 mL) was added *p*-toluenesulfonyl chloride (16.5 mmol, 1.1 equiv.) and pyridine (45.0 mmol, 3.0 equiv.). Then the mixture was stirred at 23 °C overnight. After completion of the reaction (monitored by TLC), the reaction mixture was diluted with 40 mL H_2O and extracted with Et_2O (3 × 50 mL). The combined organic layers were washed with brine, dried over $MgSO_4$, and concentrated in vacuo. The residue was purified by flash chromatography (hexanes:EtOAc = 30:1-10:1) to afford the desired sulfonamide at high yield.

^{1}H NMR (600 MHz, CDCl$_3$): δ 7.38 (d, *J* = 7.2 Hz, 2H), 7.29 (d, *J* = 5.5 Hz, 5H), 7.22 (d, *J* = 7.6 Hz, 2H), 5.17 (s, 1H), 5.03 (s, 1H), 3.95 (t, *J* = 7.0 Hz, 1H), 2.55 – 2.45 (m, 4H), 2.41 (s, 3H), 1.50 – 1.22 (m, 10H) ppm

^{13}C NMR (151 MHz, CDCl$_3$): δ 146.0, 143.8, 143.1, 136.9, 129.6, 128.9, 127.7, 127.0, 126.5, 118.0, 48.7, 37.6, 26.1, 21.7, 21.5 ppm

HRMS (ESI+): calculated for $C_{23}H_{29}NO_2S[M+H]^+$: 384.1992, found 384.1995.

4-Methyl-*N*-((1-(2-(naphthalen-2-yl)allyl)cyclohexyl)methyl)benzenesulfonamide (32)

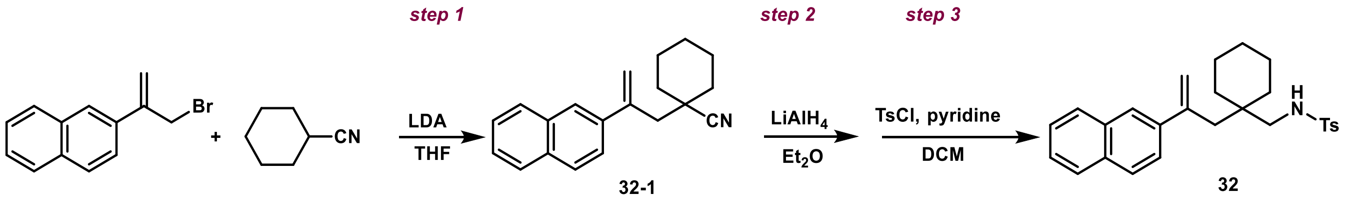

The procedure analogous to that used to synthesize **31**.

^{1}H NMR (400 MHz, CDCl$_3$): δ 7.86 (dd, J = 6.0, 3.4 Hz, 1H), 7.79 (dd, J = 9.5, 6.1 Hz, 2H), 7.69 (d, J = 1.1 Hz, 1H), 7.57 – 7.49 (m, 2H), 7.45 (dd, J = 8.5, 1.8 Hz, 1H), 7.03 (d, J = 8.3 Hz, 2H), 6.86 (d, J = 8.0

Hz, 2H), 5.31 (d, J = 1.7 Hz, 1H), 5.13 (d, J = 1.4 Hz, 1H), 3.91 (t, J = 7.3 Hz, 1H), 2.59 (s, 2H), 2.47 (d, J = 7.4 Hz, 2H), 2.30 (s, 3H), 1.47 – 1.26 (m, 10H) ppm

^{13}C NMR (101 MHz, CDCl$_3$): δ 145.8, 142.8, 141.1, 136.5, 133.6, 132.9, 129.4, 128.6, 128.2, 127.8, 126.7, 126.5, 126.3, 124.9, 124.7, 118.7, 48.7, 42.2, 37.6, 33.9, 26.1, 21.5 ppm

HRMS (ESI+): calculated for $C_{27}H_{32}NO_2S[M+H]^+$: 434.2148, found 434.2152.

4-Methyl-*N*-(5-methylhex-5-en-1-yl)benzenesulfonamide (33)

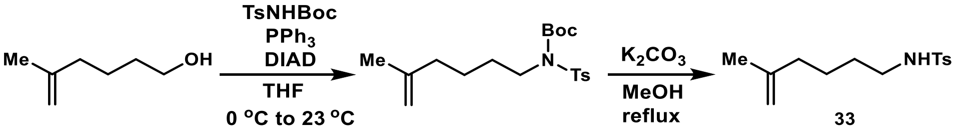

The procedure analogous to that used to synthesize **29**.

Compound **33** (1.02g, 95%) was obtained as a brown oil.

^{1}H NMR (600 MHz, CDCl$_3$): δ 7.75 (d, *J* = 8.0 Hz, 2H), 7.31 (d, *J* = 8.0 Hz, 2H), 4.67 (s, 1H), 4.59 (s, 1H), 4.54 (t, *J* = 5.8 Hz, 1H), 2.94 (q, *J* = 6.6 Hz, 2H), 2.43 (s, 3H), 1.94 (t, *J* = 7.3 Hz, 2H), 1.65 (s, 3H), 1.50 – 1.42 (m, 2H), 1.42 – 1.32 (m, 2H) ppm

^{13}C NMR (151 MHz, CDCl$_3$): δ 145.3, 143.5, 137.1, 129.8, 127.2, 110.3, 43.2, 37.2, 29.2, 24.5, 22.3, 21.6 ppm

HRMS (ESI+): calculated for $C_{14}H_{22}NO_2S$ $[M+H]^+$: 268.1366, found 268.1367.

4-Methyl-*N*-(2'-(prop-1-en-2-yl)-[1,1'-biphenyl]-2-yl)benzenesulfonamide (34)

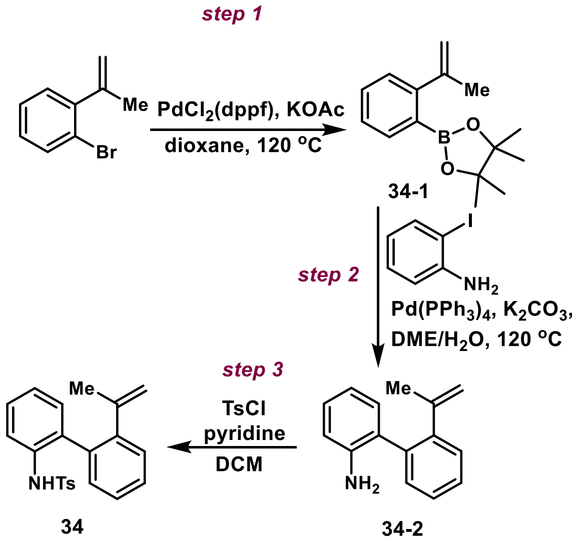

Step 1: The above compound **34-1** was prepared by following reported procedures.[23] To a mixture of 1-bromo-2-(prop-1-en-2-yl)benzene (12.0 mmol, 1 equiv.), bis(pinacolato)diboron (13.2 mmol, 1.1 equiv.), KOAc (24.0 mmol, 2 equiv.) in H_2O (3mL) and 1,4-dioxane (35 mL) under argon was added Pd(dppf)Cl_2 (0.6 mmol, 0.05 equiv.) and the suspension was degassed for 2 min while stiring vigorously. And the mixture was stirred at 120 ^{o}C for 18 h under reflux. After completion of the reaction (monitored by TLC),

the reaction mixture was diluted with 20 mL H_2O and extracted with EtOAc (3 × 30 mL). The combined organic layers were washed with brine, dried over $MgSO_4$, and concentrated in vacuo. The residue was purified by flash chromatography (hexanes:EtOAc = 30:1-10:1) to afford the desired product.

Step 2: To a solution of 2-iodoaniline (5 mmol, 1.0 equiv.) and 4,4,5,5-tetramethyl-2-(2-(prop-1-en-2-yl)phenyl)-1,3,2-dioxaborolane (5 mmol, 1.0 equiv.) in DME (25 mL) and distilled water (25 mL) under argon was added $Pd(PPh_3)_4$ (0.25 mmol, 0.05 equiv.) and K_2CO_3 (20 mmol, 4 equiv.). And then the mixture was warmed to reflux under 120 °C for 16 h. After completion of the reaction (monitored by TLC), the reaction mixture was diluted with 20 mL H_2O and extracted with EtOAc (3 × 30 mL). The combined organic layers were dried using sodium sulfate, filtered and concentrated. The residue was purified by column chromatography (hexanes:EtOAc = 10:1) to get the desired product.

Step 3: The procedure analogous to that used to synthesize **29-1**.

^{1}H NMR (600 MHz, CDCl$_3$): δ 7.66 (d, J = 8.2 Hz, 1H), 7.43 (d, J = 7.5 Hz, 2H), 7.32 (q, J = 7.8 Hz, 3H), 7.20 – 7.09 (m, 4H), 7.06 (d, J = 7.5 Hz, 1H), 6.51 (s, 1H), 6.44 (d, J = 7.5 Hz, 1H), 5.03 (s, 1H), 4.93 (s, 1H), 2.39 (s, 3H), 1.57 (s, 3H) ppm

^{13}C NMR (151 MHz, CDCl$_3$): δ 146.0, 144.0, 143.7, 136.7, 134.8, 134.2, 134.0, 130.9, 130.8, 129.9, 129.4, 128.9, 128.8, 127.8, 127.5, 125.1, 121.7, 117.4, 23.5, 21.9 ppm

HRMS (ESI+): calculated for $C_{22}H_{22}NO_2S[M+H]^+$: 364.1366, found 364.1368.

4-Methyl-*N*-(2-(3-methylbut-3-en-1-yl)benzyl)benzenesulfonamide (35)

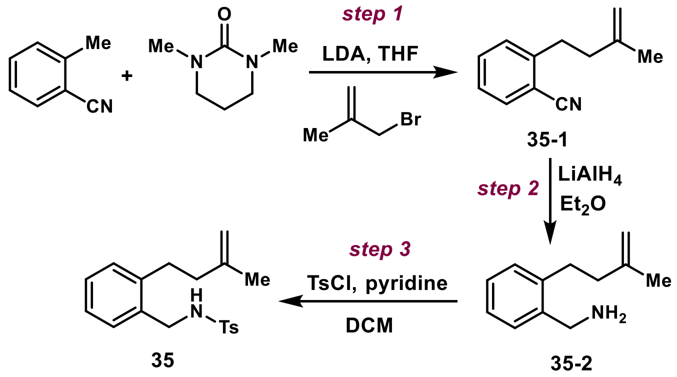

Step 1: The above nitrile intermediate **35-1** was prepared following reported procedures. The spectroscopic data was in accordance with those reported in literature.[23]

Step 2: The procedure analogous to that used to synthesize **31-2**.

Step 3: The procedure analogous to that used to synthesize **29-1**.

^{1}H NMR (400 MHz, CDCl$_3$): δ 7.82 – 7.73 (m, 2H), 7.32 (d, J = 8.0 Hz, 2H), 7.24 – 7.19 (m, 1H), 7.19 – 7.08 (m, 3H), 4.73 (s, 1H), 4.63 (d, J = 0.9 Hz, 1H), 4.41 (s, 1H), 4.11 (d, J = 5.9 Hz, 2H), 2.72 – 2.62 (m, 2H), 2.44 (s, 3H), 2.24 – 2.11 (m, 2H), 1.72 (s, 3H) ppm

^{13}C NMR (101 MHz, CDCl$_3$): δ 145.1, 143.8, 141.0, 136.6, 133.5, 130.0, 129.7, 129.6, 128.6, 127.4, 126.6, 110.7, 45.0, 39.4, 30.8, 22.7, 21.7 ppm

HRMS (ESI+): calculated for $C_{19}H_{24}NO_2S[M+H]^+$: 330.1522, found 330.1524.

***N*-(2,2-diphenylhex-4-en-1-yl)-4-methylbenzenesulfonamide (36)**

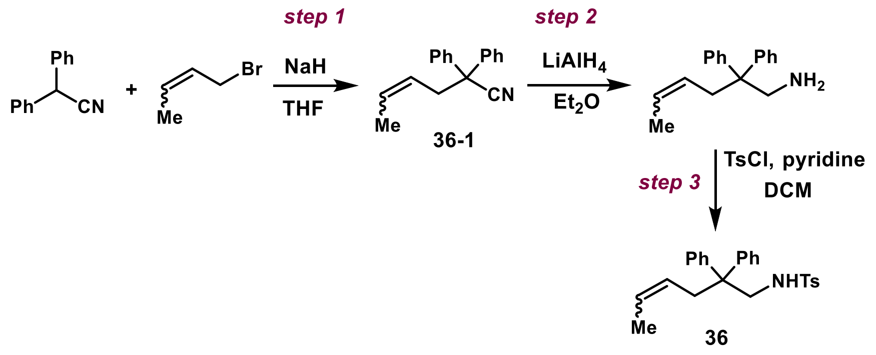

Step 1: The above alkene-substituted nitrile **36-1** was prepared by following a reported procedure.[12] To a suspension of NaH (26 mmol, 1.3 equiv.) in anhydrous THF (20 mL) was added nitrile (20 mmol, 1.0 equiv.) in anhydrous THF (10 mL) dropwise at 0 °C. The reaction mixture was allowed to stir for 1 h at 0 °C and then allyl bromide (26 mmol, 1.3 equiv.) was slowly added. The reaction mixture was warmed to 23 °C and allowed to stir at that temperature (monitored by TLC). After completion of the reaction, the reaction mixture was extracted with EtOAc (3 $\times$ 50 mL) and the combined organic phase was dired over Na_2SO_4 and concentrated under reduced pressure. The crude product was used in the next step directerly without further purification.

Step 2: The procedure analogous to that used to synthesize **31-2**.

Step 3: The procedure analogous to that used to synthesize **29-1**.

^{1}H NMR (400 MHz, CDCl$_3$): δ 7.58 (t, J = 10.0 Hz, 2H), 7.32 – 7.14 (m, 8H), 7.10 – 6.97 (m, 4H), 5.53 – 5.18 (m, 1H), 5.09 – 4.73 (m, 1H), 3.92 – 3.69 (m, 1H), 3.50 (d, J = 6.4 Hz, 2H), 2.84 (dd, J = 36.4, 7.1 Hz, 2H), 2.50 – 2.36 (m, 3H), 1.45 (ddd, J = 8.3, 6.6, 1.3 Hz, 3H) ppm

^{13}C NMR (101 MHz, CDCl$_3$): δ 144.9, 143.5, 136.3, 129.8, 129.8, 128.5, 127.9, 127.3, 126.7, 125.3, 49.6, 49.2, 40.1, 21.7, 18.2 ppm

HRMS (ESI+): calculated for $C_{25}H_{28}NO_2S[M+H]^+$: 406.1835, found 406.1838.

***N*-((1-allylcyclohexyl)methyl)-4-methylbenzenesulfonamide (37)**

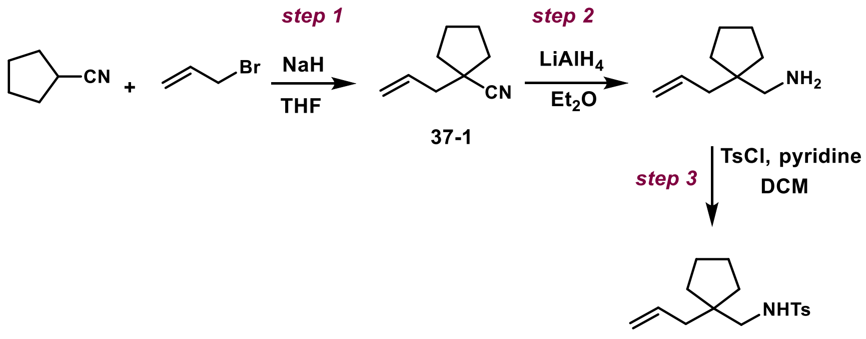

The procedure analogous to that used to synthesize **2-1**.

^{1}H NMR (600 MHz, CDCl$_3$): δ 7.73 (d, *J* = 8.2 Hz, 2H), 7.30 (d, *J* = 8.1 Hz, 2H), 5.69 (ddt, *J* = 17.3, 10.0, 7.4 Hz, 1H), 5.05 – 4.94 (m, 2H), 4.76 (t, *J* = 6.6 Hz, 1H), 2.73 (d, *J* = 6.7 Hz, 2H), 2.42 (s, 3H), 2.06 (d, *J* = 7.4 Hz, 2H), 1.63 – 1.50 (m, 4H), 1.43 – 1.30 (m, 4H) ppm

^{13}C NMR (151 MHz, CDCl$_3$): δ 143.4, 137.0, 135.1, 129.8, 127.2, 117.8, 50.2, 45.7, 42.1, 35.3, 24.8, 21.6 ppm

HRMS (ESI+): calculated for $C_{16}H_{24}NO_2S$[M+H]$^+$: 294.1520, found 294.1522.

The analytical data was consistent with the literature.[26]

4-Methyl-*N*-(2-vinylbenzyl)benzenesulfonamide (38)

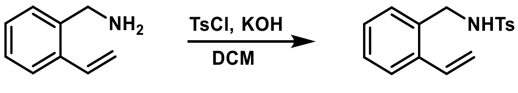

The precedure of literature about synthesis of substrate **38** was followed**.** [24]

^{1}H NMR (600 MHz, CDCl$_3$): *δ* 7.75 (d, J = 8.2 Hz, 2H), 7.45 (d, J = 7.7 Hz, 1H), 7.31 (d, J = 8.1 Hz, 2H), 7.25 (d, J = 8.0 Hz, 2H), 7.17 (dd, J = 7.4, 1.0 Hz, 1H), 7.12 (d, J = 7.0 Hz, 1H), 6.77 (dd, J = 17.3, 10.9 Hz, 1H), 5.61 (dd, J = 17.3, 1.1 Hz, 1H), 5.28 (dd, J = 10.9, 1.1 Hz, 1H), 4.48 (s, 1H), 4.16 (d, J = 5.9 Hz, 2H), 2.44 (s, 3H), 1.58 (s, 3H) ppm

^{13}C NMR (151 MHz, CDCl$_3$): *δ* 143.7, 137.2, 136.7, 133.4, 132.8, 129.9, 129.6, 128.7, 128.2, 127.4, 126.4, 117.4, 77.4, 77.2, 77.0, 45.4, 21.7 ppm

HRMS (ESI+): calculated for $C_{16}H_{18}NO_2S$[M+H]$^+$: 288.1054, found 288.1054.

4-Methyl-*N*-(5-methyl-2,2-diphenylhex-4-en-1-yl)benzenesulfonamide (39)

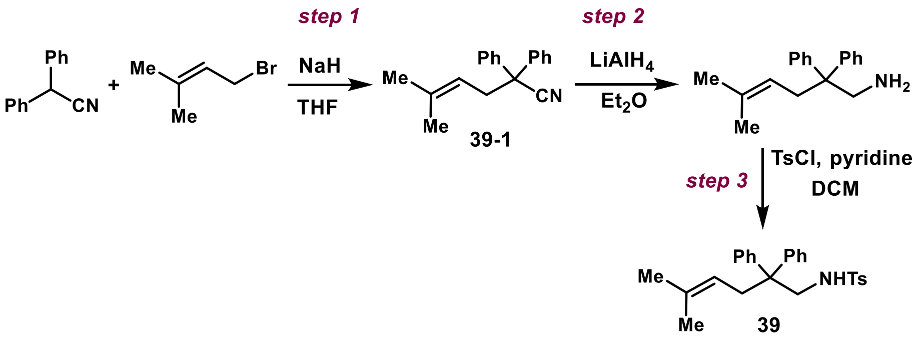

Step 1: The procedure analogous to that used to synthesize **36-1**.

Step 2: The procedure analogous to that used to synthesize **31-2**.

Step 3: The procedure analogous to that used to synthesize **29-1**.

^{1}H NMR (400 MHz, CDCl$_3$): δ 7.56 (d, J = 8.3 Hz, 2H), 7.23 (ddt, J = 11.8, 6.7, 4.0 Hz, 9H), 7.07 – 7.03 (m, 4H), 4.71 (ddd, J = 7.2, 5.9, 1.3 Hz, 1H), 3.86 (t, J = 6.2 Hz, 1H), 3.49 (d, J = 6.3 Hz, 2H), 2.80 (d, J = 7.2 Hz, 2H), 2.42 (s, 3H), 1.55 (d, J = 0.5 Hz, 3H), 1.37 (s, 3H) ppm

^{13}C NMR (101 MHz, CDCl$_3$): δ 144.9, 143.5, 136.2, 135.7, 129.8, 128.4, 128.0, 127.3, 126.8, 118.6, 50.3, 49.8, 35.7, 26.1, 21.7, 18.0 ppm

HRMS (ESI+): calculated for $C_{26}H_{30}NO_2S[M+H]^+$: 420.1992, found 420.1995.

***N*-(4-cyclobutylidenebutyl)-4-methylbenzenesulfonamide (40)**

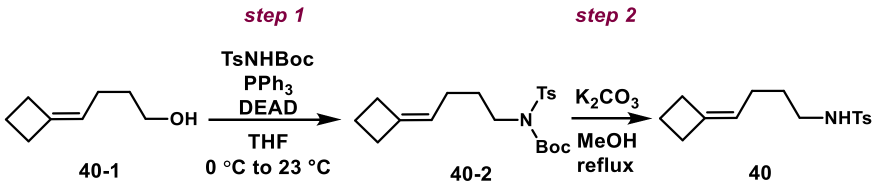

The procedure of the synthesis of **28** was followed, alcohol **40-1** was used as starting material.

^{1}H NMR (400 MHz, CDCl$_3$): δ 7.81 – 7.70 (m, 2H), 7.30 (d, J = 8.0 Hz, 2H), 4.99 – 4.83 (m, 1H), 4.73 (t, J = 6.1 Hz, 1H), 2.91 (dd, J = 13.3, 6.9 Hz, 2H), 2.66 – 2.47 (m, 4H), 2.42 (s, 3H), 2.00 – 1.73 (m, 4H), 1.47 (p, J = 7.1 Hz, 2H) ppm

^{13}C NMR (101 MHz, CDCl$_3$): δ 143.4, 141.4, 137.1, 129.8, 127.2, 118.8, 43.0, 31.0, 29.4, 29.3, 25.1, 21.6, 17.1 ppm

HRMS (ESI+): calculated for $C_{15}H_{22}NO_2S[M+H]^+$: 280.1366, found 280.1368.

4-Methyl-*N*-(2-(2-methylcyclohex-2-en-1-yl)-2,2-diphenylethyl)benzenesulfonamide (41)

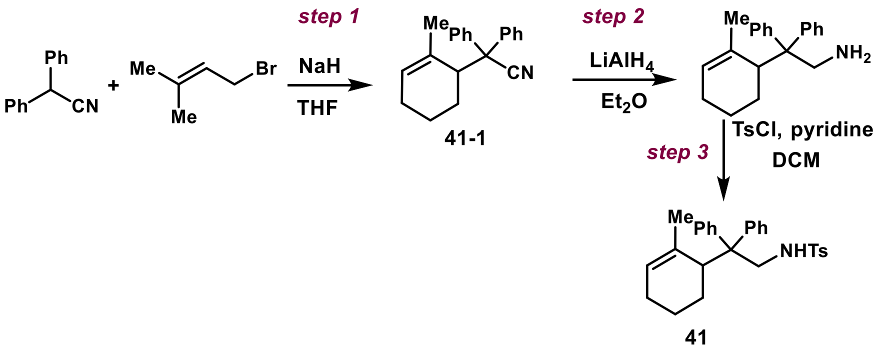

Step 1: The procedure analogous to that used to synthesize **36-1**.

Step 2: The procedure analogous to that used to synthesize **31-2**.

Step 3: The procedure analogous to that used to synthesize **29-1**.

^{1}H NMR (400 MHz, CDCl$_3$): δ 7.51 (d, J = 8.2 Hz, 2H), 7.32 – 7.26 (m, 7H), 7.23 – 7.09 (m, 5H), 5.47 (s, 1H), 4.03 (dd, J = 11.4, 8.5 Hz, 1H), 3.71 (dd, J = 8.4, 3.1 Hz, 1H), 3.16 (dd, J = 11.5, 3.3 Hz, 1H), 3.05 (s, 1H), 2.45 (s, 3H), 1.79 – 1.61 (m, 2H), 1.51 – 1.38 (m, 1H), 1.38 – 1.27 (m, 1H), 1.26 (d, J = 10.3 Hz, 3H), 1.17 – 1.03 (m, 1H), 0.90 (ddd, J = 16.8, 8.5, 4.6 Hz, 1H) ppm

^{13}C NMR (101 MHz, CDCl$_3$): δ 144.1, 143.5, 139.4, 135.9, 134.5, 130.2, 130.0, 129.8, 128.5, 127.7, 127.4, 127.2, 126.8, 54.5, 51.8, 47.2, 27.2, 25.9, 25.1, 21.7, 20.4 ppm

HRMS (ESI+): calculated for $C_{28}H_{31}NO_2S[M+H]^+$: 446.2070, found 446.2152.

5. Substrate Scope

5.1 Intramolecular Hydroalkoxylation Catalyzed by the Catalyst C

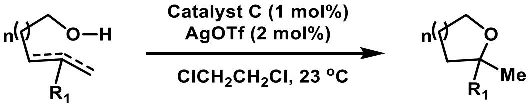

General Procedure I

In an argon filled glovebox, to a 4 mL vial with a magnetic stir bar were added the catalyst **C** (1.6 mg, 1 mol%), silver trifluoromethanesulfonate (0.5 mg, 2 mol%), the substrate (0.1 mmol, 1.0 equiv.), and $ClCH_2CH_2Cl$ (1 mL). Then the vial was taken outside of the glovebox and the resulting mixture was stirred at room temperature (23 ^{o}C) for 24 h. The reaction mixture was diluted with CH_2Cl_2, filtered through a pad of celite and concentrated. The residue was purified with silica gel chromatography (hexane/ethyl acetate = 40:1) to give the product.

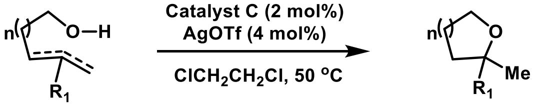

General Procedure II

In an argon filled glovebox, to a 4 mL vial with a magnetic stir bar were added the catalyst **C** (3.2 mg, 2 mol%), silver trifluoromethanesulfonate (1.0 mg, 4 mol%), the substrate (0.1 mmol, 1.0 equiv.), and $ClCH_2CH_2Cl$ (1 mL). Then the vial was taken outside of the glovebox and the resulting mixture was stirred at room temperature (23 ^oC) or 50 ^oC for 24 to 48 h. The reaction mixture was cooled to room temperature (23 ^oC), diluted with CH_2Cl_2, filtered through a pad of celite and concentrated. The residue was purified with silica gel chromatography (hexane/ethyl acetate = 40:1) to give the product.

(3a*S*,7a*S*)-3,3-Diphenyloctahydrobenzofuran (1a)

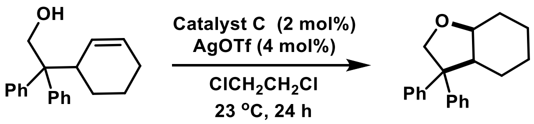

1 1a

The general procedure II was followed. The crude residue was purified by column chromatography (hexane/ethyl acetate = 40:1) to yield **1a** (25.1 mg, 90% yield) as a colorless oil.

^{1}H NMR (400 MHz, $CDCl_3$): δ 7.44 – 7.42 (m, 2H), 7.24 – 7.22 (m, 4H), 7.16 – 7.13 (m, 4H), 4.78 (d, *J* = 8.6 Hz, 1H), 4.65 (d, *J* = 8.6 Hz, 1H), 4.20 – 4.19 (m, 1H), 2.77 (dt, *J* = 10.1, 5.1 Hz, 1H), 1.97 (d, *J* = 13.5 Hz, 1H), 1.71 – 1.63 (m, 2H), 1.49 – 1.38 (m, 3H), 1.19 – 1.12 (m, 1H), 1.00 – 0.91 (m, 1H) ppm

^{13}C NMR (101 MHz, $CDCl_3$): δ 146.5, 143.6, 128.6, 128.3, 126.9, 126.2, 126.1, 76.4, 74.6, 59.7, 44.6, 29.0, 25.8, 24.6, 20.3 ppm

The analytical data was consistent with the literature.[2]

2,2-Dimethyl-5,5-diphenyltetrahydro-2*H*-pyran (3a)

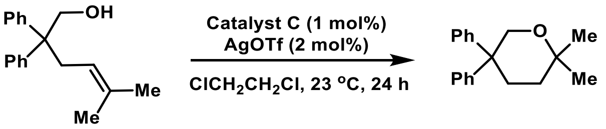

3 3a

The general procedure I was followed. The crude residue was purified by column chromatography (hexane/ethyl acetate = 40:1) to yield **3a** (24.0 mg, 90% yield) as a colorless oil.

^{1}H NMR (400 MHz, $CDCl_3$): δ 7.32 – 7.28 (m, 4H), 7.28 – 7.25 (m, 4H), 7.19 – 7.16 (m, 2H), 4.05 (s, 2H), 2.44 – 2.41 (m, 2H), 1.41 – 1.39 (m, 2H), 1.23 (s, 6H) ppm

^{13}C NMR (101 MHz, $CDCl_3$): δ 146.5, 128.2, 128.0, 126.1, 71.4, 69.1, 46.0, 32.6, 30.9, 26.5 ppm

The analytical data was consistent with the literature.[2]

3,3-Dimethyl-2-oxaspiro[5.5]undecane (4a)

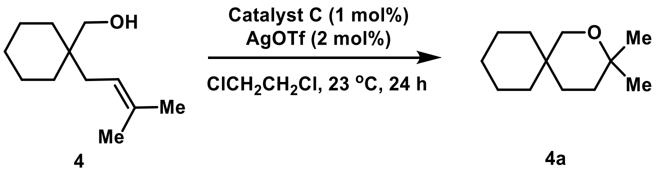

The general procedure I was followed. The crude residue was purified by column chromatography (hexane/ethyl acetate = 40:1) to yield **4a** (14.6 mg, 80% yield) as a colorless oil.

^{1}H NMR (400 MHz, CDCl$_3$): δ 3.35 (s, 2H), 1.41 – 1.25 (m, 4H), 1.15 (s, 6H) ppm

^{13}C NMR (101 MHz, CDCl$_3$): δ 71.5, 70.1, 34.0, 32.1, 31.9, 26.9, 26.4, 21.6 ppm

HRMS (ESI+): calc'd for $C_{12}H_{23}O$ [M+H]$^+$: 183.1743, found 183.1743.

7,7-Dimethyl-6-oxaspiro[3.5]nonane (5a)

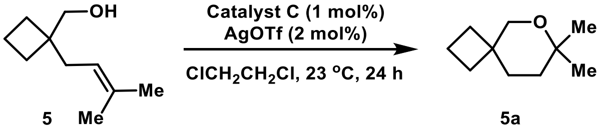

The general procedure I was followed. The crude residue was purified by column chromatography (hexane/ethyl acetate = 40:1) to yield **5a** (13.1 mg, 85% yield) as a colorless oil.

^{1}H NMR (400 MHz, CDCl$_3$): δ 3.48 (s, 2H), 1.87 – 1.73 (m, 4H), 1.69 – 1.60 (m, 2H), 1.58 (dd, *J* = 7.5, 4.9 Hz, 2H), 1.37 (dd, *J* = 7.5, 4.9 Hz, 2H), 1.14 (s, 6H) ppm

^{13}C NMR (101 MHz, CDCl$_3$): δ 70.9, 70.2, 37.9, 33.1, 32.3, 29.8, 26.2, 15.4 ppm

HRMS (ESI+): calc'd for $C_{10}H_{19}O$ [M+H]$^+$: 155.1430, found 155.1430.

2,2-Dimethyl-6,6-diphenyloxepane (6a)

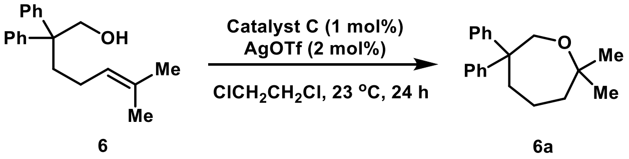

The general procedure I was followed. The crude residue was purified by column chromatography (hexane/ethyl acetate = 40:1) to yield **6a** (26.7 mg, 95% yield) as a colorless oil.

^{1}H NMR (400 MHz, CDCl$_3$): δ 7.28 – 7.21 (m, 8H), 7.15 (d, *J* = 6.9 Hz, 2H), 4.17 (s, 2H), 2.30 – 2.27 (m, 2H), 1.75 – 1.70 (m, 4H), 1.12 (s, 6H) ppm

^{13}C NMR (101 MHz, CDCl$_3$): δ 147.3, 128.2, 128.0, 125.8, 75.8, 69.6, 52.0, 42.3, 41.8, 28.0, 20.7 ppm

HRMS (ESI+): calc'd for $C_{20}H_{25}O$ [M+H]$^+$: 281.1900, found 281.1901.

Ethyl (4a*S*,8a*R*)-2,2,8a-trimethylhexahydro-2*H*-chromene-4a(5*H*)-carboxylate (7a)

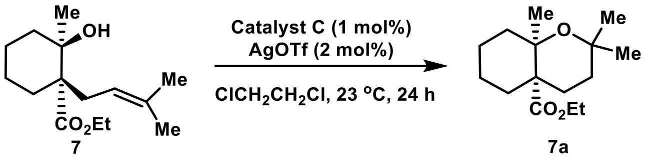

The general procedure I was followed. The crude residue was purified by column chromatography (hexane/ethyl acetate = 40:1) to yield **7a** (20.4 mg, 80% yield) as a colorless oil.

^{1}H NMR (400 MHz, CDCl$_3$): δ 4.12 (qd, *J* = 7.1, 1.0 Hz, 2H), 2.25 (td, *J* = 14.1, 4.7 Hz, 1H), 2.05 – 1.98 (m, 1H), 1.91 – 1.83 (m, 1H), 1.75 (td, *J* = 13.9, 4.8 Hz, 1H), 1.68 – 1.51 (m, 5H), 1.45 – 1.40 (m, 3H), 1.28 – 1.24 (m, 9H), 1.22 (s, 3H) ppm

^{13}C NMR (101 MHz, CDCl$_3$): δ 176.0, 73.3, 71.0, 60.3, 46.6, 38.5, 33.6, 31.9, 29.3, 28.9, 26.8, 25.3, 22.5, 21.5, 14.3 ppm

HRMS (ESI+): calc'd for $C_{15}H_{27}O_3$ $[M+H]^+$: 255.1955, found 255.1953.

5-Oxaspiro[3.5]nonane (8a)

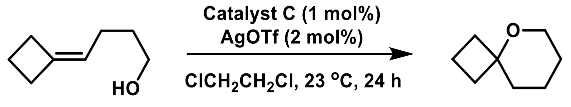

8 8a

The general procedure I was followed. The crude residue was purified by column chromatography (hexane/ethyl acetate = 40:1) to yield **8a** (6.3 mg, 50% yield) as a colorless oil. When the reaction was conducted with an internal standard, the yield was determined to be 92% yield by ^{1}H NMR.

^{1}H NMR (400 MHz, CDCl$_3$): δ 3.56 – 3.52 (m, 2H), 2.00 – 1.90 (m, 4H), 1.79 – 1.71 (m, 1H), 1.60 – 1.52 (m, 5H), 1.47 (dt, *J* = 8.2, 4.4 Hz, 2H) ppm

^{13}C NMR (101 MHz, CDCl$_3$): δ 76.7, 62.9, 34.0, 32.9, 25.9, 20.3, 12.7 ppm

HRMS (ESI+): calc'd for $C_8H_{15}O$ $[M+H]^+$: 127.1117, found 127.118.

2,2-Dimethyl-4-phenyltetrahydrofuran (9a)

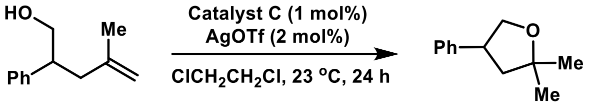

9 9a

The general procedure I was followed. The crude residue was purified by column chromatography (hexane/ethyl acetate = 40:1) to yield **9a** (16.7 mg, 95% yield) as a colorless oil.

^{1}H NMR (600 MHz, CDCl$_3$): δ 7.24 – 7.22 (m, 2H), 7.17 – 7.13 (m, 3H), 4.14 (t, *J* = 8.1 Hz, 1H), 3.71 (t, *J* = 8.9 Hz, 1H), 3.47 (dt, *J* = 17.6, 8.1 Hz, 1H), 2.14 (dd, *J* = 12.3, 8.0 Hz, 1H), 1.80 (dd, *J* = 12.1, 10.9 Hz, 1H), 1.31 (s, 3H), 1.24 (s, 3H) ppm

^{13}C NMR (151 MHz, CDCl$_3$): δ 141.9, 128.6, 127.4, 126.6, 81.5, 73.7, 47.1, 45.6, 28.9, 28.5 ppm

The analytical data was consistent with the literature[27].

2,2-Dimethyl-5-phenyltetrahydrofuran (10a)

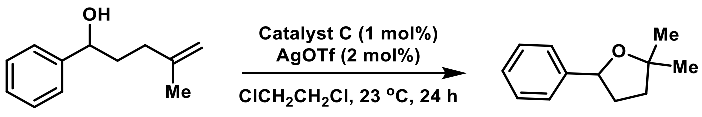

10 10a

The general procedure I was followed. The crude residue was purified by column chromatography (hexane/ethyl acetate = 40:1) to yield **10a** (16.7 mg, 95% yield) as a colorless oil.

^{1}H NMR (400 MHz, $CDCl_3$): δ 7.29 – 7.22 (m, 4H), 7.18 – 7.14 (m, 1H), 4.92 – 4.88 (m, 1H), 2.29 – 2.20 (m, 1H), 1.87 – 1.77 (m, 3H), 1.31 (s, 3H), 1.28 (s, 3H) ppm

^{13}C NMR (101 MHz, $CDCl_3$): δ 143.7, 128.4, 127.2, 125.9, 81.4, 80.6, 39.2, 35.8, 29.2, 28.5 ppm

The analytical data was consistent with the literature.[5]

3,3,8,8-Tetramethyl-2,7-dioxaspiro[4.4]nonane (11a)

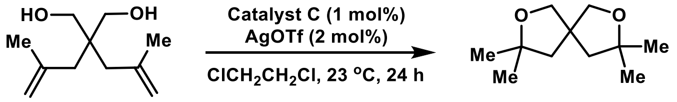

11 11a

The general procedure I was followed. The crude residue was purified by column chromatography (hexane/ethyl acetate = 40:1) to yield **11a** (16.9 mg, 92% yield) as a colorless oil.

^{1}H NMR (400 MHz, $CDCl_3$): δ 3.79 (d, J = 8.5 Hz, 2H), 3.60 (d, J = 8.5 Hz, 2H), 1.78 (s, 4H), 1.19 (d, J = 9.5 Hz, 12H) ppm

^{13}C NMR (101 MHz, $CDCl_3$): δ 80.3, 76.6, 52.9, 49.5, 29.0, 28.5 ppm

The analytical data was consistent with the literature[2].

9,9-Dimethyl-1,4,8-trioxadispiro[4.0.46.45]tetradecane (12a)

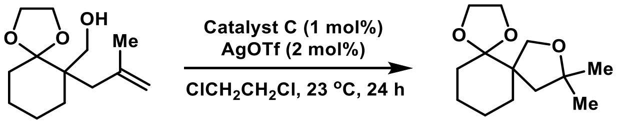

12 12a

The general procedure I was followed. The crude residue was purified by column chromatography (hexane/ethyl acetate = 40:1) to yield **12a** (19.6 mg, 87% yield) as a colorless oil.

^{1}H NMR (400 MHz, $CDCl_3$): δ 3.98 – 3.95 (m, 4H), 3.90 (d, J = 8.9 Hz, 1H), 3.57 (d, J = 8.9 Hz, 1H), 1.93 (d, J = 12.7 Hz, 1H), 1.78 – 1.68 (m 3H), 1.54 – 1.41 (m, 6H), 1.28 (s, 3H), 1.25 (s, 3H) ppm

^{13}C NMR (151 MHz, $CDCl_3$): δ 111.2, 81.1, 72.4, 65.0, 64.7, 53.1, 46.1, 36.2, 32.6, 29. 7, 27.8, 23.2, 22.2 ppm

HRMS (ESI+): calc'd for $C_{13}H_{23}O_3$ $[M+H]^+$: 227.1642, found 227.1640.

2,2-Dimethyl-2,3-dihydrobenzofuran (13a)

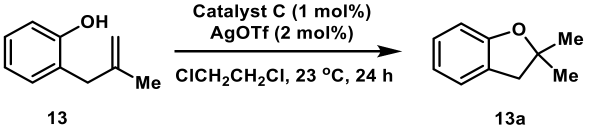

The general procedure I was followed. The crude residue was purified by column chromatography (hexane/ethyl acetate = 40:1) to yield **13a** (12.7 mg, 86% yield) as a colorless oil.

^{1}H NMR (400 MHz, CDCl$_3$): δ 7.14 – 7.09 (m, 2H), 6.84 – 6.80 (m, 1H), 6.73 (d, J = 8.0 Hz, 1H), 3.01 (s, 2H), 1.48 (s, 6H) ppm

^{13}C NMR (101 MHz, CDCl$_3$): δ 159.0, 128.1, 127.2, 125.3, 120.1, 109.7, 86.6, 43.0, 28.3 ppm

The analytical data was consistent with the literature.[6]

2,2-Dimethyl-5,5-diphenyltetrahydro-2*H*-pyran (14a)

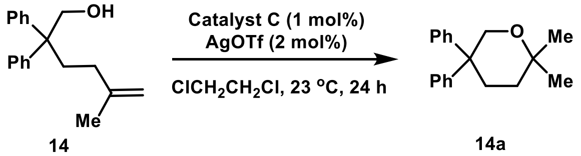

The general procedure I was followed. The crude residue was purified by column chromatography (hexane/ethyl acetate = 40:1) to yield **14a** (18.6 mg, 70% yield) as a white solid.

^{1}H NMR (400 MHz, CDCl$_3$): δ 7.33 – 7.23 (m, 8H), 7.19 – 7.14 (m, 2H), 4.05 (s, 2H), 2.44 – 2.41 (m, 2H), 1.41 – 1.38 (m, 2H), 1.22 (s, 6H) ppm

^{13}C NMR (101 MHz, CDCl$_3$): δ 146.5, 128.2, 128.0, 126.1, 71.3, 69.1, 46.0, 32.7, 30.9, 26.7 ppm

The analytical data was consistent with the literature.[2]

3,3-Dimethyl-2-oxaspiro[4.5]decane (15a)

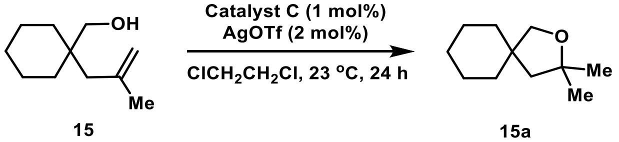

The general procedure I was followed. The crude residue was purified by column chromatography (hexane/ethyl acetate = 40:1) to yield **15a** (10.1 mg, 60% yield) as a colorless oil.

^{1}H NMR (400 MHz, CDCl$_3$): δ 3.57 (s, 2H), 1.55 (s, 2H), 1.47 – 1.36 (m, 10H), 1.22 (s, 6H) ppm

^{13}C NMR (101 MHz, CDCl$_3$): δ 80.5, 44.9, 37.0, 29.3, 26.1, 23.9 ppm

HRMS (ESI+): calc'd for $C_{11}H_{21}O$ $[M+H]^+$: 169.1587, found 169.1586.

2-Methyl-2,4,4-triphenyltetrahydrofuran (16a)

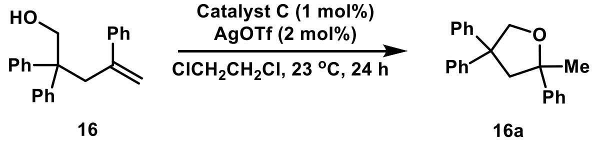

The general procedure I was followed. The crude residue was purified by column chromatography (hexane/ethyl acetate = 40:1) to yield **16a** (18.8 mg, 60% yield) as a colorless oil.

^{1}H NMR (400 MHz, CDCl$_3$): δ 7.35 – 7.30 (m, 4H), 7.24 – 7.16 (m, 4H), 7.13 – 7.07 (m, 4H), 7.04 – 6.99 (m, 3H), 4.77 (dd, *J* = 9.2, 1.0 Hz, 1H), 4.13 (d, *J* = 9.2 Hz, 1H), 3.04 (d, *J* = 12.7 Hz, 1H), 2.91 (dd, *J* = 12.6, 1.0 Hz, 1H), 1.28 (s, 3H) ppm

^{13}C NMR (101 MHz, CDCl$_3$): δ 149.6, 146.9, 146.0, 128.5, 128.4, 128.3, 127.3, 127.2, 126.4, 126.3, 124.4, 85.0, 76.1, 57.0, 52.7, 31.3 ppm

The analytical data was consistent with the literature[29].

(1*s*,4*s*)-1-methyl-2-oxabicyclo[2.2.2]octane (17a)

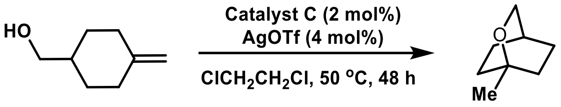

17 17a

The general procedure II was followed. The crude residue was purified by column chromatography (hexane/ethyl acetate = 40:1) to yield **17a** (6.3 mg, 50% yield) as a colorless oil.

^{1}H NMR (400 MHz, CDCl$_3$): δ 3.89 (q, *J* = 1.4 Hz, 2H), 1.82 – 1.75 (m, 4H), 1.64 – 1.55 (m, 5H), 1.04 (s, 3H) ppm

^{13}C NMR (101 MHz, CDCl$_3$): δ 70.2, 68.3, 33.0, 27.1, 25.8, 25.0 ppm

HRMS (ESI+): calc'd for $C_8H_{15}O$ $[M+H]^+$: 127.1117, found 127.1118.

2-Phenylchromane (18a)

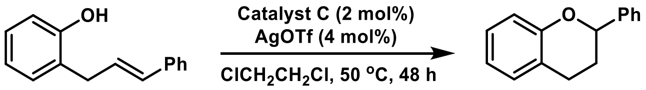

18 18a

The general procedure II was followed. The crude residue was purified by column chromatography (hexane/ethyl acetate = 40:1) to yield **18a** (16.8 mg, 80% yield) as a colorless oil.

^{1}H NMR (400 MHz, CDCl$_3$): δ 7.48 – 7.41 (m, 4H), 7.37 – 7.36 (m, 1H), 7.19 – 7.12 (m, 2H), 6.97 – 6.90 (m, 2H), 5.10 (dd, *J* = 10.1, 2.4 Hz, 1H), 3.03 (ddd, *J* = 17.0, 11.3, 6.0 Hz, 1H), 2.83 (dt, *J* = 16.5, 4.9 Hz, 1H), 2.25 (ddt, *J* = 13.6, 5.9, 3.0 Hz, 1H), 2.18 – 2.08 (m, 1H) ppm

^{13}C NMR (101 MHz, CDCl$_3$): δ 155.2, 141.9, 129.7, 128.6, 128.0, 127.5, 126.1, 122.0, 120.4, 117.1, 77.9, 30.1, 25.2 ppm

The analytical data was consistent with the literature.[10]

2,5,5-Triphenyltetrahydro-2*H*-pyran (19a)

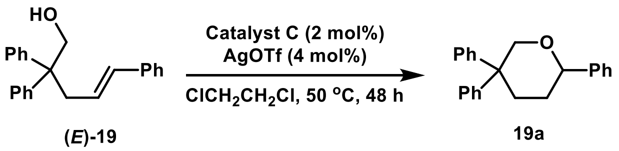

The general procedure II was followed. The crude residue was purified by column chromatography (hexane/ethyl acetate = 40:1) to yield **19a** (26.7 mg, 85% yield) as a colorless oil.

^{1}H NMR (400 MHz, $CDCl_3$): δ 7.46 – 7.44 (m, 2H), 7.27 – 7.11 (m, 13H), 4.72 (dd, J = 12.1, 2.5 Hz, 1H), 4.39 (dd, J = 11.4, 2.5 Hz, 1H), 3.63 (d, J = 12.1 Hz, 1H), 2.57 – 2.46 (m, 2H), 1.69 (dq, J = 13.7, 3.3 Hz, 1H), 1.53 – 1.43 (m, 1H) ppm

^{13}C NMR (101 MHz, $CDCl_3$): δ 146.7, 145.9, 142.8, 129.1, 128.5, 128.2, 127.6, 127.2, 126.5, 126.2, 125.9, 80.5, 75.5, 46.0, 35.2, 30.4 ppm

The analytical data was consistent with the literature.[10]

2,5,5-Triphenyltetrahydro-2*H*-pyran (20a)

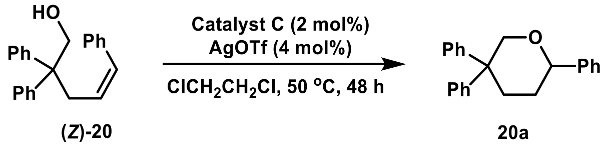

The general procedure II was followed. The crude residue was purified by column chromatography (hexane/ethyl acetate = 40:1) to yield **20a** (21.0 mg, 67% yield) as a colorless oil. The analytical data was consistent with **13a.**

2-Methyl-4,4-diphenyltetrahydrofuran (21a)

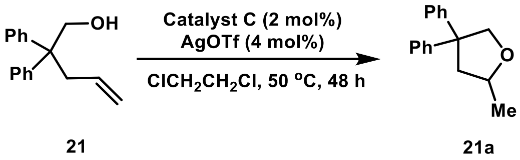

The general procedure II was followed. The crude residue was purified by column chromatography (hexane/ethyl acetate = 40:1) to yield **21a** (19.0 mg, 80% yield) as a colorless oil.

^{1}H NMR (400 MHz, $CDCl_3$): δ 7.32 – 7.28 (m, 6H), 7.23 – 7.17 (m, 4H), 4.61 – 4.58 (m, 1H), 4.22 – 4.13 (m, 2H), 2.64 (ddd, J = 12.1, 5.7, 0.9 Hz, 1H), 2.27 (dd, J = 12.1, 9.5 Hz, 1H), 1.59 (s, 1H), 1.30 (d, J = 6.1 Hz, 3H) ppm

^{13}C NMR (101 MHz, $CDCl_3$): δ 146.5, 146.3, 128.5, 128.4, 127.3, 126.5, 126.3, 77.0, 74.9, 56.5, 46.7, 21.5 ppm

The analytical data was consistent with the literature.[11]

3-Methyl-2-oxaspiro[4.5]decane (22a)

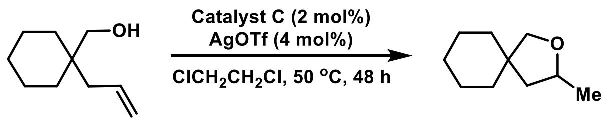

22 22a

The general procedure II was followed. The crude residue was purified by column chromatography (hexane/ethyl acetate = 40:1) to yield **22a** (10.8 mg, 70% yield) as a colorless oil.

^{1}H NMR (400 MHz, CDCl$_3$): δ 4.06 – 3.98 (m, 1H), 3.63 (d, *J* = 8.5 Hz, 1H), 3.47 (d, *J* = 8.5 Hz, 1H), 1.86 (dd, *J* = 12.3, 6.3 Hz, 1H), 1.44 –1.40 (m, 10H), 1.23 (d, *J* = 6.1 Hz, 3H), 1.20 (d, *J* = 9.1 Hz, 1H) ppm

^{13}C NMR (101 MHz, CDCl$_3$): δ 74.9, 44.3, 37.5, 36.0, 26.1, 24.1, 23.8, 21.58 ppm

The analytical data was consistent with the literature.[2]

5.2 Intermolecular Hydroalkoxylation Catalyzed by the Catalyst D

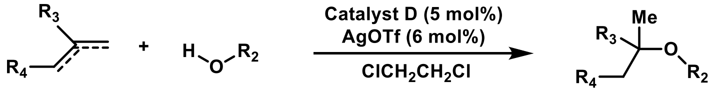

General Procedure I

In an argon filled glovebox, to a 4 mL vial with a magnetic stir bar were added the catalyst **D** (13.7 mg, 5 mol%), silver trifluoromethanesulfonate (3.1 mg, 6 mol%), alkenes (0.2 mmol, 1.0 equiv.), alcohols (0.3 mmol, 1.5 equiv.) and $ClCH_2CH_2Cl$ (1 mL). Then the vial was taken outside of the glovebox and the resulting mixture was stirred at 50 °C for 16 h. Then the reaction mixture was cooled to room temperature (23 °C) and diluted with CH_2Cl_2, filtered through a pad of celite and concentrated. The residue was purified with silica gel chromatography (hexane/ethyl acetate = 40:1) to give the product.

General Procedure II

In an argon filled glovebox, to a 4 mL vial with a magnetic stir bar were added catalyst **D** (13.7 mg, 5 mol%), silver trifluoromethanesulfonate (3.1 mg, 6 mol%), alkenes (0.2 mmol, 1.0 equiv.), alcohols (1.4 mmol, 7.0 equiv.) and $ClCH_2CH_2Cl$ (1 mL). Then the vial was taken outside of the glovebox and the resulting mixture was stirred at room temperature (23 °C) or 50 °C for 24 h. The reaction mixture was diluted with CH_2Cl_2, filtered through a pad of celite and concentrated. The residue was purified with silica gel chromatography (hexane/ethyl acetate = 40:1) to give the product.

General Procedure III

In an argon filled glovebox, to a 4 mL vial with a magnetic stir bar were added catalyst **D** (5.5 mg, 2 mol%), silver trifluoromethanesulfonate (1.5 mg, 3 mol%), alkenes (0.2 mmol, 1.0 equiv.), alcohols (0.3 mmol, 1.5 equiv.) and $ClCH_2CH_2Cl$ (1 mL), Then the vial was taken outside of the glovebox and the resulting mixture was stirred at room temperature (23 °C). The reaction mixture diluted with CH_2Cl_2,

filtered through a pad of celite and concentrated. The residue was purified with silica gel chromatography (hexane/ethyl acetate = 40:1) to give the product.

(1*R*,2*R*,4*S*)-2-(4-(4-methoxyphenyl)butoxy)bicyclo[2.2.1]heptane (23a)

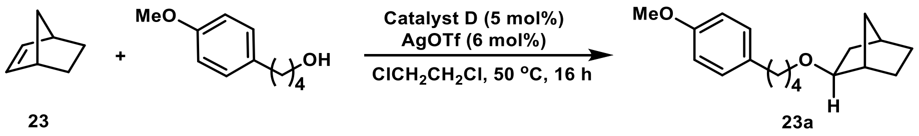

The general procedure I was followed and the reaction mixture was stirred at 50 °C for 16 h. The crude residue was purified by column chromatography (hexane/ethyl acetate = 40:1) to yield **23a** (40.0 mg, 73% yield) as a colorless oil.

^{1}H NMR (400 MHz, CDCl$_3$): δ 7.09 (d, *J* = 7.3 Hz, 2H), 6.82 (d, *J* = 7.1 Hz, 2H), 3.78 (d, *J* = 1.6 Hz, 3H), 3.43 – 3.29 (m, 3H), 2.57 (t, *J* = 7.1 Hz, 2H), 2.25 (d, *J* = 30.0 Hz, 2H), 1.62 – 1.26 (m, 9H), 1.08 – 0.96 (m, 3H) ppm

^{13}C NMR (101 MHz, CDCl$_3$): δ 157.8, 134.8, 129.4, 113.8, 82.6, 68.2, 55.4, 40.5, 39.7, 35.3, 34.9, 29.8, 28.7, 28.6, 24.8 ppm

HRMS (ESI+): calc'd for $C_{18}H_{27}O_2$ $[M+H]^+$: 275.2006, found 275.2005.

(1*S*,2*S*,4*R*)-2-(4-(trifluoromethyl)phenoxy)bicyclo[2.2.1]heptane (23b)

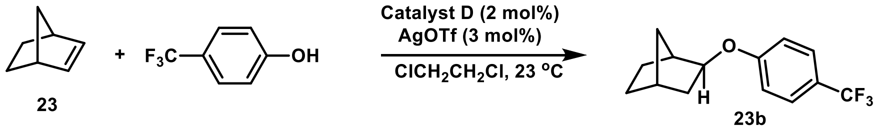

The general procedure III was followed and the reaction mixture was stirred at 23 °C for 24 h. The crude residue was purified by column chromatography (hexane) to yield **23b** (48 mg, 94% yield) as a colorless oil.

^{1}H NMR (400 MHz, CDCl$_3$): δ 7.51 (d, *J* = 8.6 Hz, 2H), 6.90 (d, *J* = 8.6 Hz, 2H), 4.20 (d, *J* = 6.6 Hz, 1H), 2.47 (*d*, *J* = 4.4 Hz, 1H), 2.33 – 2.34 (m, 1H), 1.78 (ddd, J = 13.2, 6.7, 2.4 Hz, 1H), 1.70 – 1.65 (m, 1H), 1.61 – 1.50 (m, 3H),) 1.21 (ddd, *J* = 9.9, 2.4, 1.3 Hz, 1H)., 1.18 – 1.15 (m, 2H) ppm

^{13}C NMR (101 MHz, CDCl$_3$): δ 160.5, 126.9 (q, *J* = 3.9 Hz), 124.7 (q, *J* = 271.2 Hz), 122.4 (q, *J* = 32.5 Hz), 115.5, 80.6, 41.3, 40.1, 35.6, 35.4, 28.5, 24.4 ppm

^{19}F NMR (376 MHz, CDCl$_3$): δ – 61.4 ppm

(1*S*,2*S*,4*R*)-2-(4-fluorophenoxy)bicyclo[2.2.1]heptane (23c)

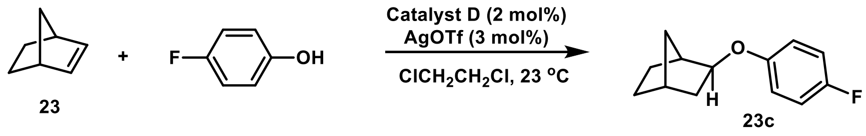

The general procedure III was followed and the reaction mixture was stirred at 23 °C for 24 h. The crude residue was purified by column chromatography (hexane) to yield **23c** (29 mg, 71% yield) as a colorless oil.

^{1}H NMR (400 MHz, CDCl$_3$): δ 6.98 – 6.90 (m, 2H), 6.82 – 6.73 (m, 2H), 4.10 (d, *J* = 6.7 Hz, 1H), 2.43 (d, *J* = 4.4 Hz, 1H), 2.31 – 2.32 (m, 1H), 1.73 (ddd, *J* = 13.1, 6.7, 2.4 Hz, 1H), 1.69 – 1.63 (m, 1H), 1.57 – 1.45 (m, 3H), 1.18 (ddd, *J* = 9.8, 2.4, 1.4 Hz, 1H), 1.14 – 1.12 (m, 2H) ppm

^{13}C NMR (101 MHz, CDCl$_3$): δ 157.1 (d, *J* = 237.5 Hz), 154.0 (d, *J* = 1.8 Hz), 116.5 (d, *J* = 7.9 Hz), 115.8 (d, *J* = 23.0 Hz), 80.8, 41.2, 40.1, 35.6, 35.28, 28.6, 24.4 ppm

^{19}F NMR (376 MHz, CDCl$_3$): δ -124.8 ppm

(1*S*,2*S*,4*R*)-2-(4-chlorophenoxy)bicyclo[2.2.1]heptane (23d)

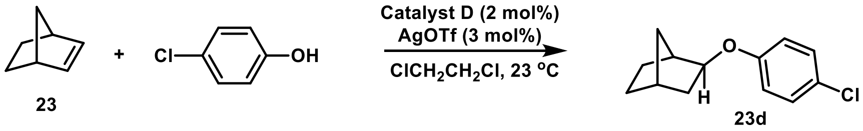

The general procedure III was followed and the reaction mixture was stirred at 23 °C for 24 h. The crude residue was purified by column chromatography (hexane) to yield **23d** (41 mg, 93% yield) as a colorless oil.

^{1}H NMR (400 MHz, CDCl$_3$): δ 7.20 (d, *J* = 9.0 Hz, 2H), 6.77 (d, *J* = 9.0 Hz, 2H), 4.11 (d, *J* = 6.6 Hz, 1H), 2.43 (d, *J* = 4.4 Hz, 1H), 2.31 – 2.32 (m, 1H), 1.74 (ddd, *J* = 13.1, 6.7, 2.4 Hz, 1H), 1.68 – 1.63 (m, 1H), 1.58 – 1.46 (m, 3H), 1.19 (ddd, *J* = 9.8, 2.4, 1.3 Hz, 1H), 1.15 – 1.13 (m, 2H) ppm

^{13}C NMR (101 MHz, CDCl$_3$): δ156.5, 129.3, 125.1, 116.8, 80.5, 41.2, 40.0, 35.6, 35.3, 28.6, 24.4 ppm

(1S,2S,4R)-2-(4-bromophenoxy)bicyclo[2.2.1]heptane (23e)

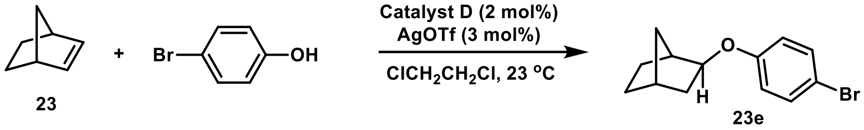

The general procedure III was followed and the reaction mixture was stirred at 23 °C for 24 h. The crude residue was purified by column chromatography (hexane) to yield **23e** (48 mg, 90% yield) as a colorless oil.

^{1}H NMR (400 MHz, CDCl$_3$): δ 7.34 (d, *J* = 9.0 Hz, 2H), 6.72 (d, *J* = 9.0 Hz, 2H), 4.11 (d, *J* = 6.6 Hz, 1H), 2.43 (d, *J* = 4.4 Hz, 1H), 2.31 – 2.32 (m, 1H), 1.74 (ddd, *J* = 13.1, 6.7, 2.4 Hz, 1H), 1.68 – 1.63 (m, 1H), 1.60 – 1.49 (m, 3H), 1.19 (ddd, *J* = 9.9, 2.4, 1.3 Hz, 1H), 1.15 – 1.12 (m, 2H) ppm

^{13}C NMR (101 MHz, CDCl$_3$): δ 157.0, 132.3, 117.4, 112.4, 80.5, 77.5, 77.2, 76.8, 41.2, 40.2, 35.6, 35.3, 28.5, 24.4 ppm

(1*S*,4*S*,5*R*)-5-(4-(4-methoxyphenyl)butoxy)-2,2-dimethylbicyclo[2.2.1]heptane (24a)

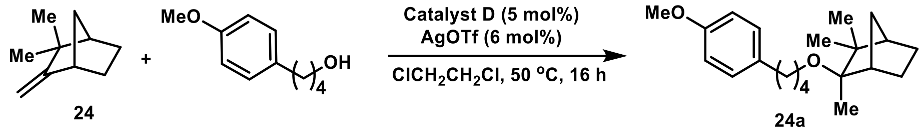

The general procedure I was followed and the reaction mixture was stirred at 50 °C for 16 h. The crude residue was purified by column chromatography (hexane/ethyl acetate = 40:1) to yield **21a** (58.2 mg, 92% yield) as a colorless oil.

^{1}H NMR (400 MHz, CDCl$_3$): δ 7.10 (d, *J* = 8.6 Hz, 2H), 6.82 (d, *J* = 8.6 Hz, 2H), 3.79 (s, 3H), 3.41 (dt, *J* = 9.1, 6.2 Hz, 1H), 3.26 (dt, *J* = 9.1, 6.1 Hz, 1H), 3.15 (dd, *J* = 7.5, 3.4 Hz, 1H), 2.56 (t, *J* = 7.6 Hz, 2H), 1.75 – 1.45 (m, 1H), 1.00 – 0.98 (m, 4H), 0.88 (s, 3H), 0.80 (s, 3H) ppm

^{13}C NMR (101 MHz, CDCl$_3$): δ 157.7, 135.0, 129.4, 113.8, 87.1, 69.0, 55.4, 49.3, 46.5, 45.2, 38.8, 34.9, 34.7, 29.8, 28.6, 27.5, 20.4, 20.4, 12.4 ppm

HRMS (ESI+): calc'd for $C_{21}H_{33}O_2$ $[M+H]^+$: 339.2295, found 339.2296.

Ethyl 4-(1-ethoxycyclobutyl)butanoate (25a)

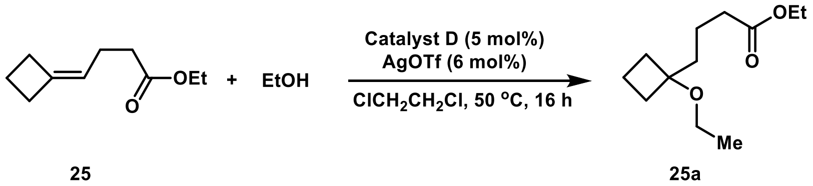

The general procedure I was followed and the reaction mixture was stirred at 50 °C for 16 h. The crude residue was purified by column chromatography (hexane/ethyl acetate = 40:1) to yield **25a** (34.2 mg, 80% yield) as a colorless oil.

^{1}H NMR (400 MHz, CDCl$_3$): δ 4.12 (q, *J* = 7.1 Hz, 2H), 3.27 (q, *J* = 7.0 Hz, 2H), 2.33 – 2.30 (m, 2H), 2.09– 2.01 (m, 2H), 1.88 – 1.82 (m, 2H), 1.77 – 1.63 (m, 5H), 1.56 – 1.49 (m, 1H), 1.24 (t, *J* = 7.2 Hz, 3H), 1.16 (t, *J* = 7.0 Hz, 3H) ppm

^{13}C NMR (101 MHz, CDCl$_3$): δ 173.8, 78.7, 60.3, 56.7, 34.6, 34.5, 32.2, 18.8, 16.0, 14.4, 12.8 ppm

HRMS (ESI+): calc'd for $C_{12}H_{23}O_3$ $[M+H]^+$: 215.1642, found 215.1641.

7-Methoxy-3,7-dimethyloctyl benzoate (26a)

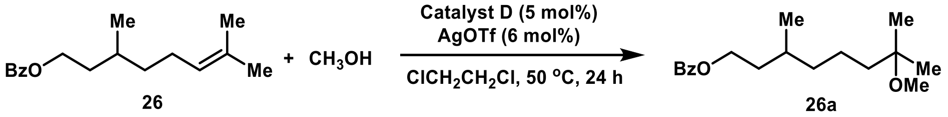

The general procedure II was followed and the reaction mixture was stirred at 50 °C for 24 h. The crude residue was purified by column chromatography (hexane/ethyl acetate = 40:1) to yield **26a** (39.7 mg, 68% yield) as a colorless oil.

^{1}H NMR (400 MHz, $CDCl_3$): δ 8.05 – 8.02 (m, 2H), 7.57 – 7.53 (m, 1H), 7.45 – 7.41 (m, 2H), 4.41 – 4.31 (m, 2H), 3.17 (s, 3H), 1.86 – 1.78 (m, 1H), 1.63 – 1.53 (m, 1H), 1.46 – 1.18 (m, 7H), 1.13 (s, 6H), 0.97 (d, *J* = 6.5 Hz, 3H) ppm

^{13}C NMR (101 MHz, $CDCl_3$): δ 166.8, 132.9, 130.6, 129.7, 128.5, 74.7, 63.7, 49.2, 40.2, 37.6, 35.7, 30.1, 25.1, 21.3, 19.7 ppm

HRMS (ESI+): calc'd for $C_{18}H_{29}O_3$ $[M+H]^+$: 293.2111, found 293.2110.

3,7-Dimethyl-7-(2,2,2-trifluoroethoxy)octyl benzoate (26b)

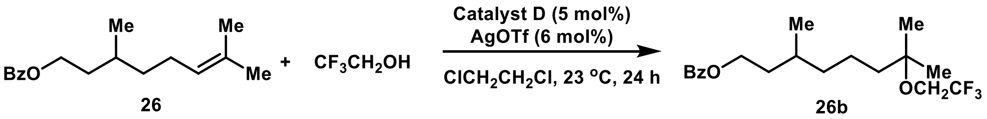

The general procedure II was followed and the reaction mixture was stirred at 23 °C for 24 h. The crude residue was purified by column chromatography (hexane/ethyl acetate = 40:1) to yield **26b** (47.5 mg, 66% yield) as a colorless oil.

^{1}H NMR (400 MHz, $CDCl_3$): δ 8.05 – 8.03 (m, 2H), 7.58 – 7.53 (m, 1H), 7.46 – 7.42 (m, 2H), 4.41 – 4.31 (m, 2H), 3.69 (q, *J* = 8.7 Hz, 2H), 1.84 – 1.77 (m, 1H), 1.62 – 1.54 (m, 1H), 1.47 – 1.21 (m, 7H), 1.18 (s, 6H), 0.97 (d, *J* = 6.5 Hz, 3H) ppm

^{13}C NMR (101 MHz, $CDCl_3$): δ 166.8, 132.9, 130.6, 129.7, 128.5, 124.3 (q, *J* = 281.1 Hz), 76.9, 63.6, 60.5 (q, *J* = 34.2 Hz), 40.5, 37.4, 35.7, 30.0, 25.2, 21.1, 19.6 ppm

^{19}F NMR (565 MHz, $CDCl_3$): δ -74.5 (t, *J* = 8.5 Hz) ppm

HRMS (ESI+): calc'd for $C_{19}H_{28}F_3O_3$ $[M+H]^+$: 383.1805, found 383.1802.

(5-Methoxy-5-methylhexyl)benzene (27a)

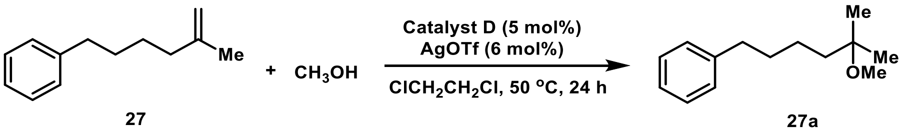

The general procedure II was followed and the reaction mixture was stirred at 50 °C for 24 h. The crude residue was purified by column chromatography (hexane/ethyl acetate = 40:1) to yield **27a** (27.6 mg, 67% yield) as a colorless oil.

^{1}H NMR (400 MHz, $CDCl_3$): δ 7.29 – 7.25 (m, 2H), 7.19 – 7.17 (m, 3H), 3.17 (s, 3H), 2.62 (t, *J* = 8.0 Hz, 2H), 1.65 – 1.58 (m, 2H), 1.51 – 1.47 (m, 2H), 1.40 – 1.32 (m, 2H), 1.13 (s, 6H) ppm

^{13}C NMR (101 MHz, $CDCl_3$): δ 142.9, 128.5, 128.4, 125.7, 74.7, 49.2, 39.9, 36.1, 32.3, 25.1, 23.8 ppm

HRMS (ESI+): calc'd for $C_{14}H_{22}ONa$ $[M+Na]^+$: 229.1563, found 229.1568.

(5-Ethoxy-5-methylhexyl)benzene (27b)

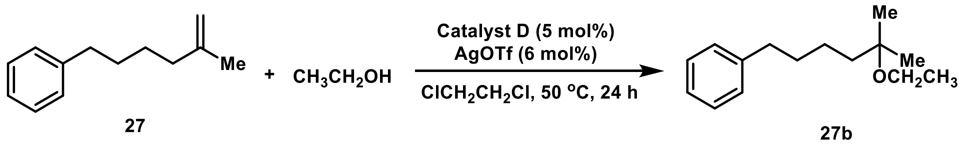

The general procedure II was followed and the reaction mixture was stirred at 50 °C for 24 h. The crude residue was purified by column chromatography (hexane/ethyl acetate = 40:1) to yield **27b** (13.6 mg, 31% yield) as a colorless oil.

^{1}H NMR (400 MHz, $CDCl_3$): δ 7.29 – 7.25 (m, 2H), 7.19 – 7.15 (m, 3H), 3.35 (q, *J* = 7.0 Hz, 2H), 2.62 (t, *J* = 8.0 Hz, 2H), 1.65 – 1.57 (m, 2H), 1.51 – 1.47 (m, 2H), 1.41-1.33 (m, 2H), 1.16 – 1.12 (m, 9H) ppm

^{13}C NMR (101 MHz, $CDCl_3$): δ 142.9, 128.5, 128.4, 125.7, 74.5, 56.4, 40.1, 36.1, 32.3, 25.8, 23.8, 20.2, 16.3 ppm

HRMS (ESI+): calc'd for $C_{15}H_{24}ONa$ $[M+Na]^+$: 243.1719, found 243.1716.

(5-Methyl-5-(2,2,2-trifluoroethoxy)hexyl)benzene (27c)

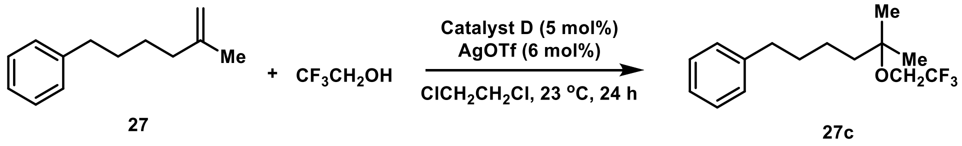

The general procedure II was followed and the reaction mixture was stirred at 23 °C for 24 h. The crude residue was purified by column chromatography (hexane/ethyl acetate = 40:1) to yield **27c** (29.0 mg, 53% yield) as a colorless oil.

^{1}H NMR (600 MHz, $CDCl_3$): δ 7.30 – 7.26 (m, 2H), 7.19 – 7.18 (m, 3H), 3.70 (q, *J* = 8.7 Hz, 2H), 2.63 (t, *J* = 5.2 Hz, 2H), 1.66 – 1.60 (m, 2H), 1.53 – 1.51 (m, 2H), 1.43 – 1.38 (m, 2H), 1.19 (s, 6H) ppm

^{13}C NMR (151 MHz, $CDCl_3$): δ 142.7, 128.5, 128.4, 125.8, 124.5 (q, *J* = 277.8 Hz), 76.9, 60.5 (q, *J* = 34.2 Hz), 40.3, 36.0, 32.0, 25.2, 23.6 ppm

^{19}F NMR (565 MHz, CDCl3): δ -74.5 (t, *J* = 8.1 Hz) ppm

HRMS (ESI+): calc'd for $C_{15}H_{21}F_3ONa$ $[M+Na]^+$: 297.1437, found 297.1437.

4-(1-((4-(trifluoromethyl)benzyl)oxy)cyclobutyl)butyl benzoate (46a)

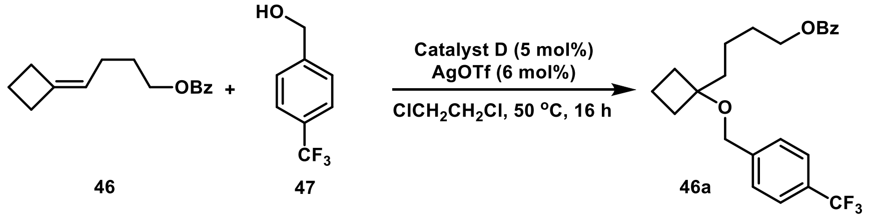

The general procedure I was followed and the reaction mixture was stirred at 50 °C for 16 h. The crude residue was purified by column chromatography (hexane/ethyl acetate = 40:1) to yield **46a** (73 mg, 90% yield) as a pale yellow oil.

^{1}H NMR (400 MHz, CDCl$_3$): δ 8.03 – 8.02 (m, 2H), 7.59 – 7.51 (m, 3H), 7.46 – 7.38 (m, 4H), 4.41 – 4.35 (m, 4H), 2.23 – 2.15 (m, 2H), 2.01 – 1.91 (m, 2H), 1.87 – 1.79 (m, 5H), 1.65 – 1.51 (m, 3H) ppm

^{13}C NMR (101 MHz, CDCl$_3$): δ 166.7, 143.5, 133.0, 130.5, 129.6, 129.5 (q, J = 32.2 Hz), 128.5, 127.4, 125.32 (q, J = 3.7 Hz), 124.4 (q, J = 272.9 Hz), 79.0, 64.9, 63.4, 35.1, 32.1, 29.1, 19.7, 12.9 ppm

^{19}F NMR (376 MHz, CDCl$_3$): δ -62.4 ppm

HRMS (ESI+): calc'd for $C_{23}H_{25}F_3O_3Na$ $[M+Na]^+$: 429.1648, found 429.1648.

1-methoxy-4-(5-methoxy-5-methylhexyl)benzene (48a)

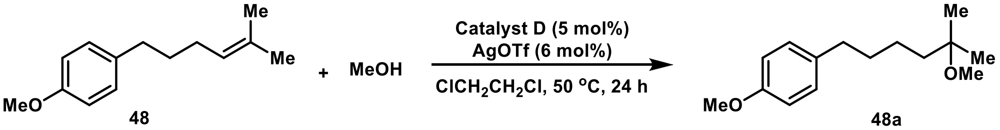

The general procedure II was followed and the reaction mixture was stirred at 50 °C for 24 hours. The crude residue was purified by column chromatography (hexane/ethyl acetate = 40:1) to yield **48a** (26.4 mg, 56% yield) as a colorless oil.

^{1}H NMR (400 MHz, CDCl$_3$): δ 7.09 (d, J = 8.4 Hz, 2H), 6.82 (d, J = 8.4 Hz, 2H), 3.79 (s, 3H), 3.17 (s, 3H), 2.56 (t, J = 8.0 Hz, 2H), 1.62 – 1.54 (m, 2H), 1.50 – 1.46 (m, 2H), 1.39 – 1.33 (m, 2H), 1.13 (s, 6H) ppm

^{13}C NMR (101 MHz, CDCl$_3$): δ 157.7, 135.0, 129.4, 113.8, 74.8, 55.4, 49.2, 39.9, 35.2, 32.5, 25.1, 23.7 ppm

HRMS (ESI+): calc'd for $C_{15}H_{24}O_2Na$ $[M+Na]^+$: 259.1669, found 259.1663.

5.3 HOTf-catalyzed Hydroarylation of Norbornene with Phenol

General Procedure

In an argon filled glovebox, to a 4 mL vial with a magnetic stir bar were added HOTf (0.6 mg, 2 mol%), alkenes (0.2 mmol, 1.0 equiv.), alcohols (0.3 mmol, 1.5 equiv.) and $ClCH_2CH_2Cl$ (1 mL), Then the vial was taken outside of the glovebox and the resulting mixture was stirred at room temperature (23 °C) for 24 h. The reaction mixture diluted with CH_2Cl_2, filtered through a pad of celite and concentrated. The residue was purified with silica gel chromatography (hexane/ethyl acetate = 20:1) to give the product.

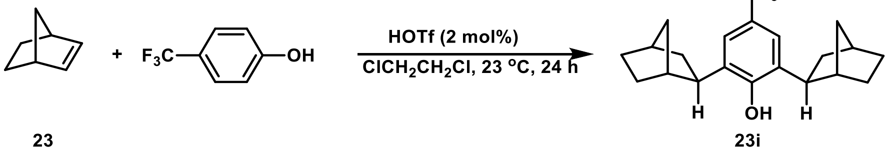

The crude residue was purified by column chromatography (hexane/ethyl acetate = 20:1) to yield **23i** (19 mg, 54% yield, 1:1 dr) as a white solid.

^{1}H NMR (600 MHz, $CDCl_3$, 2 isomers): δ 7.29 (s, 2H), 5.10 (s, 1H), 2.84 – 2.81 (m, 2H), 2.45 (s, 2H), 2.39 (s, 2H), 1.87 – 1.83 (m, 2H), 1.70 – 1.65 (m, 2H), 1.64 – 1.59 (m, 2H), 1.54 – 1.49 (m, 4H), 1.42 – 1.38 (m, 2H) , 1.35 – 1.30 (m, 2H), 1.28 – 1.27 (m, 2H) ppm

^{13}C NMR (150 MHz, $CDCl_3$, 2 isomers): δ [154.05, 154.03], [133.00, 132.98], 125.08 (d, *J* = 271.4 Hz), [121.85 (d, *J* = 31.6 Hz), 121.83 (d, *J* = 23.8 Hz)],125.56 – 125.51 (m), [41.02, 41.96], [40.86, 40.85], [38.32, 38.23], [37.05, 37.03], [36.37, 36.32], [30.36, 30.34], [29.17, 29.16] ppm

^{19}F NMR (564 MHz, $CDCl_3$, 2 isomers): δ -61.03 ppm

HRMS (ESI): calc'd for $C_{21}H_{24}F_3O$ $[M-H]^-$: 349.1779, found 349.1783.

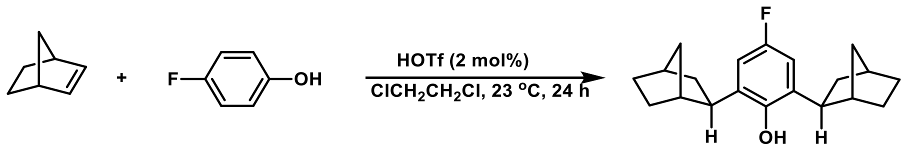

23 23j

The crude residue was purified by column chromatography (hexane/ethyl acetate = 20:1) to yield **23j** (21 mg, 67% yield, 1:1 dr) as a white solid.

^{1}H NMR (600 MHz, $CDCl_3$, 2 isomers): δ 6.76 (s, 1H), 6.74 (s, 1H), 4.55 (s, 1H), 2.81 – 2.79 (m, 2H), 2.38 – 2.37 (m, 4H), 1.85 – 1.80 (m, 2H), 1.68 – 1.56 (m, 4H), 1.52 – 1.47 (m, 4H), 1.40 – 1.36 (m, 2H), 1.33 – 1.28 (m, 2H) , 1.26 – 1.23 (m, 2H) ppm

^{13}C NMR (150 MHz, $CDCl_3$, 2 isomers): δ 157.00 (d, *J* = 235.7 Hz), [147.01, 146.99], [134.38, 134.34], [109.81 (d, *J* = 23.8 Hz), 109.78 (d, *J* = 23.8 Hz)], [41.23, 41.15], 41.06, [38.50, 38.40], [36.98, 36.96], [36.34, 36.29], [30.37, 30.25], [29.16, 29.14] ppm

^{19}F NMR (564 MHz, $CDCl_3$, 2 isomers): δ -123.69, -123.72 ppm

HRMS (ESI): calc'd for $C_{20}H_{24}FO$ $[M-H]^-$: 299.1811, found 299.1818.

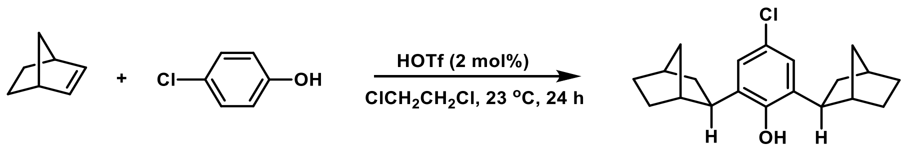

23 23 k

The crude residue was purified by column chromatography (hexane/ethyl acetate = 20:1) to yield **23k** (22 mg, 71% yield, 1:1 dr) as a white solid.

^{1}H NMR (400 MHz, $CDCl_3$, 2 isomers): δ 6.99 (s, 2H), 4.71 (s, 1H), 2.80 – 2.76 (m, 2H), 2.43 – 2.33 (m, 4H), 1.86 – 1.76 (m, 2H), 1.65 – 1.58 (m, 4H), 1.53 – 1.47 (m, 4H), 1.41 – 1.20 (m, 8H) ppm

^{13}C NMR (101 MHz, $CDCl_3$, 2 isomers): δ [149.85, 149.83], [134.47, 134.45], 125.0, [123.31, 123.28], [41.11, 41.04], [40.98, 40.96], [38.42, 38.32], [37.01, 36.99], [36.37, 36.32], [30.38, 30.36], [29.17, 29.15] ppm

HRMS (ESI): calc'd for $C_{20}H_{24}ClO$ $[M-H]^-$: 315.1516, found 315.1522.

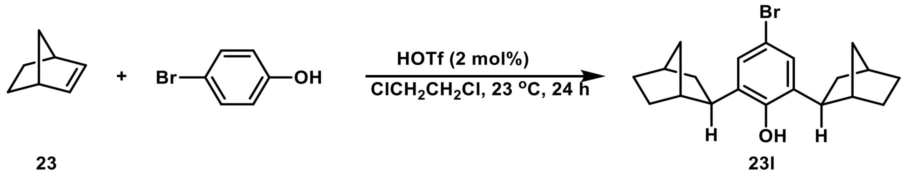

The crude residue was purified by column chromatography (hexane/ethyl acetate = 20:1) to yield **23l** (52 mg, 75% yield, 1:1 dr) as a white solid.

^{1}H NMR (600 MHz, CDCl$_3$, 2 isomers): δ 7.13 (s, 2H), 4.73 (s, 1H), 2.79 – 2.76 (m, 2H), 2.39 – 2.37 (m, 4H), 1.85 – 1.78 (m, 2H), 1.65 – 1.55 (m, 4H), 1.51 – 1.48 (m, 4H), 1.37 – 1.23 (m, 8H) ppm

^{13}C NMR (150 MHz, CDCl$_3$, 2 isomers): δ [150.39, 150.37], [134.95, 134.93], [126.22, 126.19], 112.70, [41.08, 41.02], [40.95, 40.93], [38.41, 38.31], [37.01, 36.98], [36.36, 36.32], [30.37, 30.35], [29.15, 29.14] ppm

HRMS (ESI): calc'd for $C_{20}H_{24}BrO$ [M-H]$^-$: 359.1011, found 359.1016.

Norbornene and para-substituted phenols (CF_3, F, Cl, Br) underwent hydroalkoxylation catalyzed by complex **D**/AgOTf at either room temperature (23 ^{o}C) and 50 ^{o}C. However, hydroarylation products were formed when triflic acid replaced catalyst **D**. These results indicated our catalytic systerm was different from trifilic acid.

5.4 Intramolecular Hydroamination by the Catalyst E

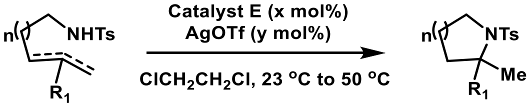

General Procedure I: To a 4 mL vial equipped with a magnetic stir bar were added the catalyst **E** (0.001mmol, 1 mol%), silver trifluoromethanesulfonate (0.6 mg, 0.002 mmol, 2 mol%) and $ClCH_2CH_2Cl$ (1.0 mL) in a nitrogen filled glovebox. The mixture was stirred at 23 ^{o}C for 1 h. Then to the mixture was added a solution of **37** (30.7 mg, 0.1 mmol, 1.0 equiv) in $ClCH_2CH_2Cl$ (0.5 mL). The vial was taken outside of the glovebox and the resulting mixture was stirred at 23 ^{o}C for 24 h. When the reaction was finished (monitered by TLC), the mixture was diluted with CH_2Cl_2 and concentrated under reduced pressure. The residue was purified by flash chromatography (EtOAc/hexane = 1/20) to afford the desired product.

General Procedure II: To a 4 mL vial equipped with a magnetic stir bar were added the catalyst **E** (0.005mmol, 5 mol%), silver trifluoromethanesulfonate (1.5 mg, 0.006 mmol, 6 mol%) and $ClCH_2CH_2Cl$ (1.0 mL) in a nitrogen filled glovebox. The mixture was stirred at 23 ^{o}C for 1 h. Then to the mixture was added a solution of **37** (30.7 mg, 0.1 mmol, 1.0 equiv) in $ClCH_2CH_2Cl$ (0.5 mL). The vial was taken outside of the glovebox and the resulting mixture was stirred at 50 ^{o}C for 48 h. When the reaction was finished (monitered by TLC), the mixture was diluted with CH_2Cl_2 and concentrated under reduced

pressure. The residue was purified by flash chromatography (EtOAc/hexane = 1/20) to afford the desired product.

3-Methyl-2-tosyl-2-azaspiro[4.5]decane (2a)

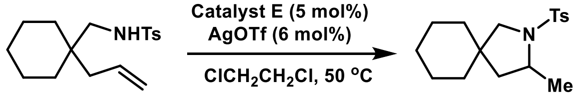

The general procedure II was followed. White solid (19.0 mg, 62% yield).

^{1}H NMR (400 MHz, CDCl$_3$): δ 7.72 (d, J = 8.2 Hz, 2H), 7.30 (d, J = 8.0 Hz, 2H), 3.56 (dp, J = 8.6, 6.2 Hz, 1H), 3.24 (d, J = 10.7 Hz, 1H), 3.14 (d, J = 10.7 Hz, 1H), 2.42 (s, 3H), 1.80 (dd, J = 12.6, 7.2 Hz, 1H), 1.49 – 1.06 (m, 13H), 0.89 – 0.62 (m, 2H) ppm

^{13}C NMR (101 MHz, CDCl$_3$): δ 143.2, 135.2, 129.6, 127.6, 58.9, 55.3, 47.2, 41.1, 36.7, 34.4, 26.0, 23.8, 23.0, 22.9, 21.6 ppm

HRMS (ESI+): calculated for $C_{17}H_{26}NO_2S[M+H]^+$: 308.1679, found 308.1678.

2,2-Dimethyl-1-tosylpyrrolidine (28a)

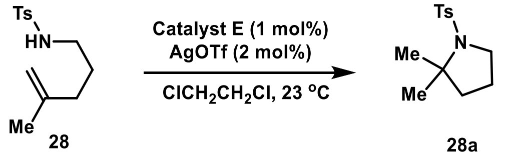

The general procedure I was followed. White solid (21.2 mg, 84% yield).

1H NMR (400 MHz, CDCl3): δ 7.73 (d, J = 8.2 Hz, 2H), 7.27 (d, J = 8.1 Hz, 2H), 3.39 (t, J = 6.4 Hz, 2H), 2.41 (s, 3H), 1.87 – 1.71 (m, 4H), 1.44 (s, 6H) ppm

13C NMR (101 MHz, CDCl3): δ 142.6, 138.8, 129.5, 127.2, 65.2, 49.5, 43.0, 28.4, 22.6, 21.6 ppm

HRMS (ESI+): calculated for $C_{13}H_{20}NO_2S[M+H]^+$: 254.1209, found 254.1206.

2,2-Dimethyl-1-tosylindoline (29a)

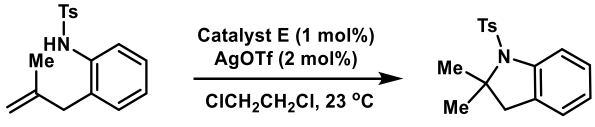

The general procedure I was followed. White solid (25.6 mg, 85% yield).

^{1}H NMR (400 MHz, CDCl$_3$): *δ* 7.84 – 7.75 (m, 2H), 7.47 (d, J = 8.2 Hz, 1H), 7.24 (t, J = 6.9 Hz, 2H), 7.16 – 7.11 (m, 1H), 7.10 (dd, J = 5.9, 5.3 Hz, 1H), 6.93 (td, J = 7.4, 0.8 Hz, 1H), 2.97 (s, 2H), 2.37 (s, 3H), 1.66 (s, 7H) ppm

^{13}C NMR (101 MHz, CDCl$_3$): *δ* 143.5, 142.1, 139.4, 129.7, 128.4, 127.8, 126.8, 125.1, 122.8, 114.2, 77.5, 76.9, 70.1, 46.2, 28.7, 21.6 ppm

HRMS (ESI+): calculated for $C_{17}H_{20}NO_2S[M+H]^+$: 302.1209, found 302.1205.

9a-Methyl-9-tosyl-2,3,4,4a,9,9a-hexahydro-1*H*-carbazole (30a)

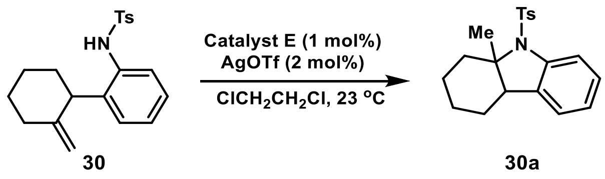

The general procedure I was followed. White solid (21.5 mg, 63% yield).

^{1}H NMR (600 MHz, CDCl$_3$): δ 7.82 (d, *J* = 8.1 Hz, 2H), 7.51 (d, *J* = 8.1 Hz, 1H), 7.24 (d, *J* = 8.0 Hz, 2H), 7.15 (t, *J* = 7.8 Hz, 1H), 7.10 (d, *J* = 7.3 Hz, 1H), 6.99 (t, *J* = 7.4 Hz, 1H), 2.95 (s, 1H), 2.38 (s, 3H), 2.11 – 1.98 (m, 2H), 1.82 (t, *J* = 12.6 Hz, 1H), 1.74 (s, 4H), 1.52 – 1.43 (m, 2H), 1.37 – 1.18 (m, 2H) ppm

^{13}C NMR (151 MHz, CDCl$_3$): δ 143.4, 142.2, 139.6, 133.0, 129.6, 127.6, 126.7, 122.9, 122.78, 114.8, 73.8, 48.1, 34.9, 24.3, 23.9, 21.7, 21.5, 20.8 ppm

HRMS (ESI+): calculated for $C_{20}H_{24}NO_2S$[M+H]$^+$: 342.1522, found 342.1520.

3-Methyl-3-phenyl-2-tosyl-2-azaspiro[4.5]decane (31a)

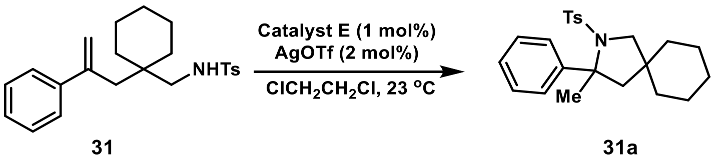

The general procedure I was followed. White solid (36.9 mg, 96% yield).

^{1}H NMR (400 MHz, CDCl3): δ 7.54 (d, *J* = 8.3 Hz, 2H), 7.40 (dd, *J* = 8.2, 0.9 Hz, 2H), 7.26 (t, *J* = 7.5 Hz, 2H), 7.22 – 7.17 (m, 3H), 3.48 (d, *J* = 9.8 Hz, 1H), 3.40 (d, *J* = 9.8 Hz, 1H), 2.40 (s, 3H), 2.19 (d, *J* = 13.3 Hz, 1H), 1.97 (d, *J* = 13.3 Hz, 1H), 1.90 (s, 3H), 1.44 – 1.24 (m, 10H) ppm

^{13}C NMR (101 MHz, CDCl$_3$): *δ* 144.7, 142.6, 138.0, 133.1, 132.3, 129.2, 128.5, 127.9, 127.3, 126.0, 124.7, 124.5, 77.5, 76.8, 69.8, 40.7, 37.2, 28.2, 25.8, 23.5, 21.6 ppm

HRMS (ESI+): calculated for $C_{23}H_{30}NO_2S$[M+H]$^+$: 384.1992, found 384.1987.

3-Methyl-3-(naphthalen-2-yl)-2-tosyl-2-azaspiro[4.5]decane 32a

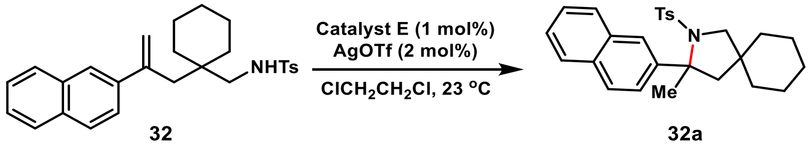

The general procedure I was followed. White solid (40.8 mg, 94% yield).

1H NMR (400 MHz, CDCl3): *δ* 7.85 (d, *J* = 1.7 Hz, 1H), 7.83 – 7.74 (m, 2H), 7.67 (d, *J* = 8.7 Hz, 1H), 7.57 – 7.37 (m, 5H), 7.08 (d, *J* = 8.0 Hz, 2H), 3.60 (d, *J* = 9.8 Hz, 1H), 3.48 (d, *J* = 9.8 Hz, 1H), 2.36 (s, 3H), 2.28 (d, *J* = 13.4 Hz, 1H), 2.12 – 1.95 (m, 4H), 1.52 – 1.24 (m, 10H) ppm

^{13}C NMR (101 MHz, CDCl$_3$): *δ* 144.7, 142.6, 138.0, 133.1, 132.3, 129.2, 128.5, 127.9, 127.3, 126.0, 124.7, 124.5, 77.5, 76.8, 69.8, 40.7, 37.2, 28.2, 25.8, 23.5, 21.6 ppm

HRMS (ESI+): calculated for $C_{27}H_{32}NO_2S[M+H]^+$: 434.2148, found 434.2145.

2,2-Dimethyl-1-tosylpiperidine (33a)

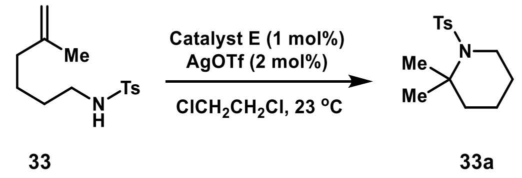

The general procedure I was followed. White solid (23.5 mg, 88% yield).

^{1}H NMR (400 MHz, CDCl$_3$): *δ* 7.62 (d, *J* = 8.3 Hz, 2H), 7.26 – 7.11 (m, 2H), 3.52 – 3.32 (m, 2H), 2.34 (s, 3H), 1.64 – 1.45 (m, 5H), 1.45 – 1.35 (m, 2H), 1.21 (s, 7H) ppm

^{13}C NMR (101 MHz, CDCl$_3$): *δ* 142.7, 140.5, 129.5, 127.1, 77.5, 76.8, 58.0, 43.8, 41.5, 26.3, 21.6, 20.6 ppm

HRMS (ESI+): calculated for $C_{14}H_{23}NO_2S[M+H]^+$: 268.1366, found 268.1362.

6,6-Dimethyl-5-tosyl-5,6-dihydrophenanthridine (34a)

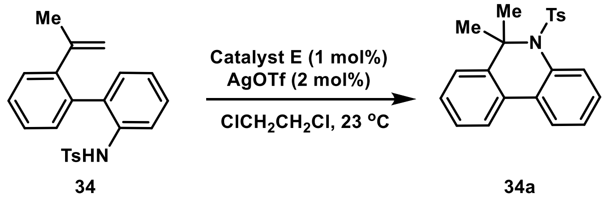

The general procedure I was followed. White solid (28.6 mg, 79% yield).

^{1}H NMR (600 MHz, CDCl$_3$): δ 7.73 (dd, *J* = 7.7, 1.1 Hz, 1H), 7.55 (dd, *J* = 7.5, 1.4 Hz, 1H), 7.38 (dtd, *J* = 19.5, 7.4, 1.4 Hz, 2H), 7.20 (d, *J* = 7.6 Hz, 1H), 7.08 (m, 3H), 6.84 (d, *J* = 8.2 Hz, 2H), 6.67 (d, *J* = 8.0 Hz, 2H), 2.17 (s, 6H), 1.25 (s, 3H) ppm

^{13}C NMR (151 MHz, CDCl$_3$): *δ* 142.6, 140.2, 136.9, 135.2, 131.8, 131.1, 130.7, 128.3, 128.2, 127.7, 127.4, 127.0, 123.7, 123.3, 77.4, 77.0, 60.5, 29.8, 21.4 ppm

HRMS (ESI+): calculated for $C_{22}H_{23}NO_2S[M+H]^+$: 364.1366, found 364.1360.

3,3-Dimethyl-2-tosyl-2,3,4,5-tetrahydro-1*H*-benzo[*c*]azepine (35a)

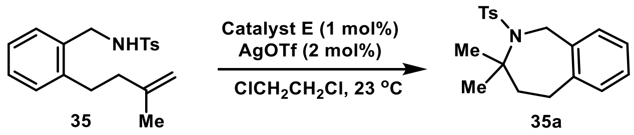

The general procedure I was followed. White solid (25.6 mg, 78% yield).

^{1}H NM (600 MHz, CDCl$_3$): *δ* 7.29 – 7.23 (m, 2H), 7.18 (dd, *J* = 15.1, 6.3 Hz, 3H), 7.08 (d, *J* = 7.1 Hz, 1H), 7.04 (d, *J* = 8.1 Hz, 2H), 4.84 (s, 2H), 2.80 (d, *J* = 10.8 Hz, 2H), 2.32 (s, 3H), 1.94 – 1.86 (m, 2H), 1.41 (s, 6H) ppm

^{13}C NMR (151 MHz, CDCl$_3$): *δ* 142.4, 141.3, 139.4, 137.3, 129.7, 129.2, 128.9, 127.5, 126.8, 126.2, 77.4, 77.0, 61.2, 48.0, 42.1, 30.0, 26.6, 21.5 ppm

HRMS (ESI+): calculated for $C_{19}H_{24}NO_2S[M+H]^+$: 330.1552, found 330.1518.

2-Ethyl-4,4-diphenyl-1-tosylpyrrolidine (36a)

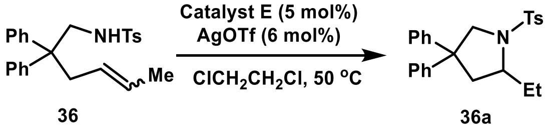

The general procedure II was followed. White solid (25.1 mg, 62% yield).

^{1}H NMR (400 MHz, CDCl3): δ 7.56 (d, J = 8.2 Hz, 2H), 7.32 – 7.17 (m, 5H), 7.17 – 7.04 (m, 7H), 4.14 (d, J = 10.4 Hz, 1H), 4.04 (dd, J = 10.5, 0.9 Hz, 1H), 3.63 (d, J = 6.8 Hz, 1H), 2.84 – 2.69 (m, 1H), 2.38 (d, J = 6.6 Hz, 3H), 2.30 (dd, J = 12.8, 7.3 Hz, 1H), 2.04 (ddd, J = 11.9, 7.6, 3.3 Hz, 1H), 1.37 – 1.23 (m, 2H), 0.82 (t, J = 7.5 Hz, 3H) ppm

^{13}C NMR (101 MHz, CDCl3): δ 145.9, 144.9, 143.0, 135.8, 129.6, 128.6, 127.2, 126.7, 126.6, 126.6, 126.3, 77.48 (s), 76.8, 61.3, 58.8, 52.5, 42.6, 28.3, 21.6, 10.2 ppm

HRMS (ESI+): calculated for $C_{25}H_{28}NO_2S[M+H]^+$: 406.1835, found 406.1829.

3-Methyl-2-tosyl-2-azaspiro[4.4]nonane (37a)

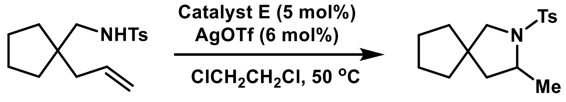

The general procedure II was followed. Colorless oil (28.4 mg, 97% yield)

^{1}H NMR (400 MHz, CDCl3): δ 7.71 (d, J = 8.2 Hz, 2H), 7.30 (d, J = 8.1 Hz, 2H), 3.71 – 3.51 (m, 1H), 3.25 (d, J = 10.2 Hz, 1H), 3.07 (d, J = 10.2 Hz, 1H), 2.42 (s, 3H), 1.81 (dd, J = 12.1, 7.7 Hz, 1H), 1.65 – 1.42 (m, 7H), 1.40 (d, J = 6.2 Hz, 3H), 1.01 (dt, J = 14.7, 7.4 Hz, 1H), 0.96 – 0.83 (m, 1H) ppm

^{13}C NMR (101 MHz, CDCl3): δ 143.2, 135.3, 129.6, 127.6, 60.1, 56.3, 48.6, 46.9, 36.8, 36.7, 24.6, 24.4, 22.9, 21.6 ppm

HRMS (ESI+): calculated for $C_{16}H_{24}NO_2S[M+H]^+$: 294.1520, found 294.1522.

1-Methyl-2-tosylisoindoline (38a)

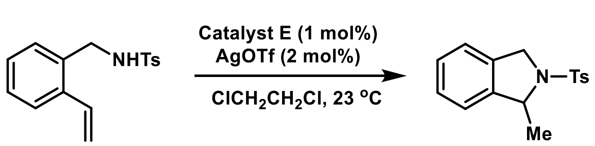

The general procedure I was followed. White solid (26.7 mg, 93% yield).

^{1}H NMR (600 MHz, CDCl3): δ 7.75 (d, J = 8.2 Hz, 2H), 7.27 (d, J = 8.1 Hz, 2H), 7.22 (dd, J = 13.1, 6.9 Hz, 2H), 7.14 (d, J = 7.0 Hz, 1H), 7.10 (d, J = 7.3 Hz, 1H), 4.92 (d, J = 6.3 Hz, 1H), 4.73 (d, J = 13.8 Hz, 1H), 4.56 (d, J = 13.7 Hz, 1H), 2.38 (s, 3H), 1.67 (d, J = 6.4 Hz, 3H) ppm

^{13}C NMR (151 MHz, CDCl$_3$): δ 143.6, 141.8, 135.1, 134.8, 129.9, 127.9, 127.67 (s), 122.5, 77.4, 77.0, 62.0, 53.9, 23.9, 21.6 ppm

HRMS (ESI+): calculated for $C_{16}H_{18}NO_2S[M+H]^+$: 288.1053, found 288.1049.

2,2-Dimethyl-5,5-diphenyl-1-tosylpiperidine (39a)

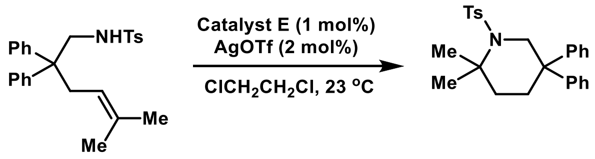

39 39a

The general procedure I was followed. White solid (39.0 mg, 93% yield).

^{1}H NMR (600 MHz, CDCl$_3$): δ 7.40 (d, J = 7.9 Hz, 2H), 7.24 (d, J = 7.9 Hz, 4H), 7.20 (t, J = 7.6 Hz, 4H), 7.10 (dd, J = 17.8, 7.6 Hz, 4H), 4.03 (s, 2H), 2.36 – 2.28 (m, 5H), 1.50 – 1.36 (m, 2H), 1.17 (s, 6H) ppm

^{13}C NMR (151 MHz, CDCl$_3$): δ 145.9, 143.0, 139.6, 129.5, 128.6, 127.6, 127.4, 126.3, 77.37 (s), 77.0, 57.5, 51.2, 46.6, 38.3, 31.1, 25.9, 21.6 ppm

HRMS (ESI+): calculated for $C_{26}H_{30}NO_2S[M+H]^+$: 420.1992, found 420.1987.

5-Tosyl-5-azaspiro[3.5]nonane (40a)

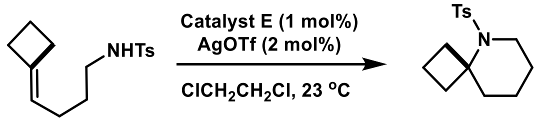

40 40a

The general procedure I was followed. White solid (26.7 mg, 96% yield).

1H NMR (400 MHz, CDCl3): δ 7.69 (d, J = 8.3 Hz, 2H), 7.36 – 7.13 (m, 2H), 3.47 – 3.31 (m, 2H), 2.42 (s, 3H), 2.32 – 2.16 (m, 2H), 2.05 – 1.90 (m, 2H), 1.83 – 1.74 (m, 2H), 1.72 – 1.63 (m, 2H), 1.63 – 1.51 (m, 4H) ppm

^{13}C NMR (101 MHz, CDCl$_3$): δ 142.6, 141.3, 129.6, 126.7, 77.5, 76.8, 61.0, 44.5, 34.1, 31.8, 25.5, 21.6, 21.1, 14.6 ppm

HRMS (ESI+): calculated for $C_{15}H_{22}NO_2S[M+H]^+$: 280.1366, found 280.1363.

7a-Methyl-3,3-diphenyl-1-tosyloctahydro-1*H*-indole (41a)

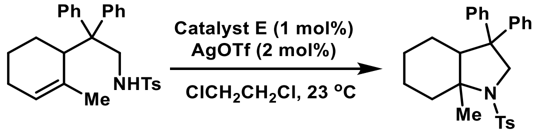

41 41a

The general procedure II was followed. White solid (40.6 mg, 91% yield).

^{1}H NMR (600 MHz, CDCl$_3$): δ 7.59 (d, J = 8.1 Hz, 2H), 7.19 – 6.96 (m, 12H), 6.89 (d, J = 7.6 Hz, 2H), 4.29 (t, J = 19.9 Hz, 1H), 3.02 (d, J = 10.0 Hz, 1H), 2.56 (d, J = 6.5 Hz, 1H), 2.23 (d, J = 10.1 Hz, 3H),

2.19 (d, J = 18.8 Hz, 1H), 2.10 (t, J = 17.7 Hz, 1H), 1.90 (t, J = 25.2 Hz, 1H), 1.67 – 1.57 (m, 1H), 1.55 – 1.39 (m, 2H), 1.30 (s, 3H), 1.12 (dd, J = 25.9, 21.1 Hz, 2H), 0.89 (ddd, J = 20.7, 7.4, 3.8 Hz, 2H), 0.19 – -0.10 (m, 1H) ppm

^{13}C NMR (101 MHz, $CDCl_3$): δ 147.4, 144.8, 142.9, 137.8, 129.6, 128.5, 127.7, 127.2, 126.6, 77.5, 76.8, 67.2, 59.3, 53.8, 53.6, 49.7, 36.3, 26.7, 23.4, 21.6, 21.3, 20.6 ppm

HRMS (ESI+): calculated for $C_{28}H_{32}NO_2S[M+H]^+$: 446.2148, found 446.2143.

5.5 Intermolecular Hydroamination Catalyzed by the Catalyst E

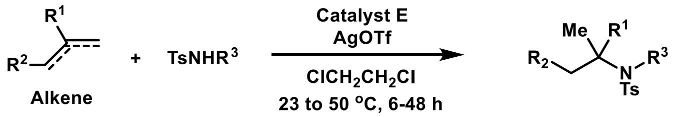

General Procedure I

In a nitrogen filled glovebox, to a 4 mL vial with a magnetic stir bar were added catalyst **E** (3.0 mg, 0.002 mmol, 2 mol%), silver trifluoromethanesulfonate (1.0 mg, 0.004 mmol, 4 mol%), alkenes (0.1 mmol, 1.0 equiv.), sulfonamide (0.15 mmol, 1.5 equiv.) and $ClCH_2CH_2Cl$ (0.7 mL). Then the vial was taken outside of the glovebox and the resulting mixture was stirred at 23 °C or 50 °C for 8 to 24 h (unless otherwise stated: see experimental details). The reaction mixture was diluted with CH_2Cl_2, filtered through a pad of celite and concentrated. The residue was purified with silica gel chromatography (EtOAc/hexane= 1/20 to 1/3) to give the product.

General Procedure II

In a nitrogen filled glovebox, to a 5 mL vial with a magnetic stir bar were added catalyst **E** (3.0 mg, 0.002 mmol, 2 mol%), silver trifluoromethanesulfonate (1.0 mg,0.004 mmol, 4 mol%), alkenes (0.5 mmol, 5.0 equiv.), sulfonamide (0.1 mmol, 1.0 equiv.) and $ClCH_2CH_2Cl$ (0.7 mL). Then the vial was taken outside of the glovebox and the resulting mixture was stirred at 23 °C or 50 °C for 6 to 8 h (unless otherwise stated: see experimental details). The reaction mixture was diluted with CH_2Cl_2, filtered through a pad of celite and concentrated. The residue was purified with silica gel chromatography (EtOAc/hexane= 1/20 to 1/3) to give the product.

General procedure III

In a nitrogen filled glovebox, to a 4 mL vial with a magnetic stir bar were added catalyst **E** (7.6 mg, 0.005 mmol, 5 mol%), silver trifluoromethanesulfonate (1.5 mg, 0.006 mmol, 6 mol%), alkenes (0.1 mmol, 1.0 equiv.), sulfonamide (0.15 mmol, 1.5 equiv.) and $ClCH_2CH_2Cl$ (0.7 mL). Then the vial was taken outside of the glovebox and the resulting mixture was stirred at 50 °C for 48 h (unless otherwise stated: see experimental details). The reaction mixture was diluted with CH_2Cl_2, filtered through a pad of celite and concentrated. The residue was purified with silica gel chromatography (EtOAc/hexane= 1/20 to 1/3) to

give the product.

Ehyl 4-(1-((4-methylphenyl)sulfonamido)cyclobutyl)butanoate (25b)

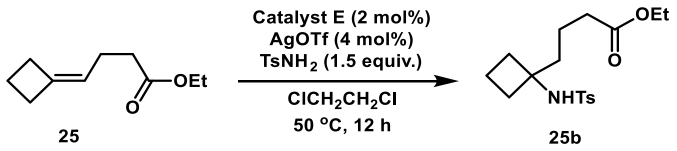

The general procedure I was followed and the reaction mixture was stirred at 50 °C for 12 h. The crude residue was purified by column chromatography (EtOAc/hexane= 1/20 to 1/3) to yield **25b** (26.1 mg, 77% yield) as a white solid.

^{1}H NMR (400 MHz, $CDCl_3$): δ 7.76 (d, J = 8.0 Hz, 2H), 7.27 (d, J = 8.0 Hz, 2H), 4.94 (s, 1H), 4.11 (q, J = 7.1 Hz, 2H), 2.41 (s, 3H), 2.19 – 2.04 (m, 4H), 1.94 – 1.81 (m, 2H), 1.79 – 1.59 (m, 4H), 1.58 – 1.42 (m, 2H), 1.24 (t, J = 7.1 Hz, 3H) ppm

^{13}C NMR (101 MHz, $CDCl_3$): δ 173.6, 143.2, 140.1, 129.7, 127.1, 60.4, 59.6, 37.2, 34.0, 33.6, 21.6, 19.1, 14.9, 14.4 ppm

HRMS (ESI+): calc'd for $C_{17}H_{26}NO_4S$ $[M+H]^+$: 340.1583, found 340.1573.

3,7-Dimethyl-7-((4-methylphenyl)sulfonamido)octyl benzoate (26c)

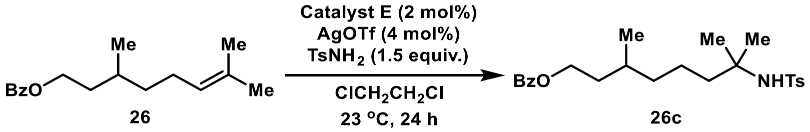

The general procedure I was followed and the reaction mixture was stirred at 23 °C for 24 h. The crude residue was purified by column chromatography (EtOAc/hexane= 1/20 to 1/3) to yield **26c** (39.7 mg, 92% yield) as a white solid.

^{1}H NMR (400 MHz, $CDCl_3$): δ 8.04 (dd, J = 8.3, 1.3 Hz, 2H), 7.76 (d, J = 8.3 Hz, 2H), 7.63 – 7.49 (m, 1H), 7.49 – 7.40 (m, 2H), 7.26 (d, J = 8.3 Hz, 2H), 4.60 (s, 1H), 4.46 – 4.22 (m, 2H), 2.40 (s, 3H), 1.83 – 1.68 (m, 1H), 1.54 (dd, J = 11.5, 5.4 Hz, 2H), 1.47 – 1.39 (m, 2H), 1.33 – 1.19 (m, 4H), 1.16 (d, J = 2.5 Hz, 6H), 1.13 – 1.02 (m, 1H), 0.91 (d, J = 6.4 Hz, 3H) ppm

^{13}C NMR (101 MHz, $CDCl_3$): δ 166.8, 142.9, 140.7, 133.0, 130.6, 129.7, 129.6, 128.5, 127.1, 63.5, 57.2, 43.2, 37.2, 35.6, 30.0, 27.9, 27.8, 21.6, 21.3, 19.6 ppm

HRMS (ESI+): calc'd for $C_{24}H_{34}NO_4S$ $[M+H]^+$: 432.2209, found 432.2201.

4-Methyl-N-(1-methylcyclohexyl)benzenesulfonamide (42a)

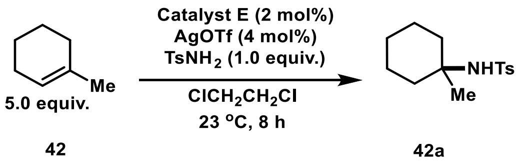

The general procedure II was followed and the reaction mixture was stirred at 23 °C for 8 h. The crude

residue was purified by column chromatography (hexane: EtOAc = 20:1 to 3:1) to yield **42a** (23.0 mg, 86% yield) as a white solid.

^{1}H NMR (400 MHz, CDCl$_3$): δ 7.78 (d, J = 8.1 Hz, 2H), 7.26 (d, J = 8.1 Hz, 2H), 4.70 (s, 1H), 2.41 (s, 3H), 1.76 – 1.63 (m, 2H), 1.38 (d, J = 8.9 Hz, 8H), 1.17 (s, 3H) ppm

^{13}C NMR (101 MHz, CDCl$_3$): δ 142.8, 141.0, 129.6, 127.0, 57.0, 38.5, 26.9, 25.3, 21.9, 21.6 ppm

HRMS (ESI+): calc'd for $C_{14}H_{21}NO_2SNa$ $[M+Na]^+$: 290.1191, found 290.1182.

4-Methyl-N-(1-methylcyclopentyl)benzenesulfonamide (43a)

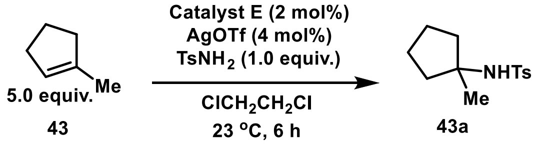

The general procedure II was followed and the reaction mixture was stirred at 23 °C for 6 h. The crude residue was purified by column chromatography (hexane: EtOAc = 20:1 to 3:1) to yield **43a** (24.0 mg, 95% yield) as a white solid.

^{1}H NMR (600 MHz, CDCl$_3$): δ 7.78 (d, J = 8.2 Hz, 2H), 7.28 (d, J = 8.2 Hz, 2H), 4.79 (s, 1H), 2.42 (s, 3H), 1.86 – 1.74 (m, 2H), 1.61 – 1.51 (m, 4H), 1.50 – 1.46 (m, 2H), 1.28 (s, 3H) ppm

^{13}C NMR (151 MHz, CDCl$_3$): δ 142.9, 140.5, 129.6, 127.0, 64.8, 40.2, 26.7, 23.0, 21.6 ppm

HRMS (ESI+): calc'd for $C_{13}H_{19}NO_2SNa$ $[M+Na]^+$: 276.1034, found 276.1025.

***N*-((1*S*,2*R*,4*R*)-2,3,3-trimethylbicyclo[2.2.1]heptan-2-yl)methanesulfonamide (24b)**

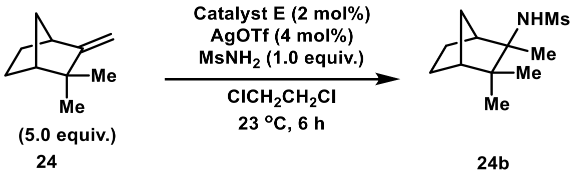

The general procedure II was followed and the reaction mixture was stirred at 23 °C for 6 h. The crude residue was purified by column chromatography (EtOAc/hexane= 1/20 to 1/3) to yield **24b** (22.6 mg, 98% yield) as a white solid.

^{1}H NMR (600 MHz, CDCl$_3$): δ 4.50 (d, J = 8.9 Hz, 1H), 3.28 (td, J = 8.9, 4.2 Hz, 1H), 2.92 (s, 3H), 1.93 – 1.85 (m, 1H), 1.76 – 1.66 (m, 3H), 1.60 (td, J = 12.1, 4.2 Hz, 1H), 1.21 – 1.15 (m, 1H), 1.14 – 1.08 (m, 1H), 0.92 (s, 3H), 0.88 (s, 3H), 0.83 (s, 3H) ppm

^{13}C NMR (151 MHz, CDCl$_3$): δ 61.6, 48.7, 47.1, 45.1, 41.3, 40.6, 36.4, 27.0, 20.4, 20.3, 12.5 ppm

HRMS (ESI+): calc'd for $C_{11}H_{21}NO_2SNa$ $[M+Na]^+$: 254.1191, found 254.1181.

4-Methyl-*N*-(2-methyl-6-phenylhexan-2-yl)benzenesulfonamide (27d)

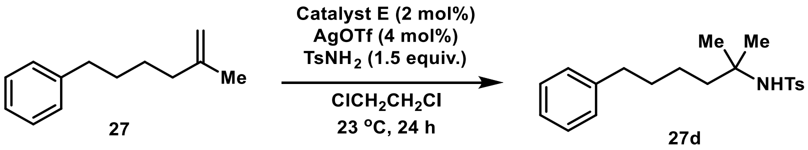

The general procedure I was followed and the reaction mixture was stirred at 23 °C for 24 h. The crude residue was purified by column chromatography (EtOAc/hexane= 1/20 to 1/3) to yield **27d** (29.7 mg, 86% yield) as a white solid.

^{1}H NMR (400 MHz, CDCl$_3$): δ 7.69 (d, J = 8.3 Hz, 1H), 7.22 – 7.18 (m, 4H), 7.14 – 7.07 (m, 1H), 7.06 (d, J = 8.3 Hz, 2H), 4.61 (s, 1H), 2.46 (t, J = 8.1 Hz, 1H), 2.33 (s, 3H), 1.48 – 1.33 (m, 4H), 1.27 – 1.14 (m, 2H), 1.08 (s, 6H) ppm

^{13}C NMR (101 MHz, CDCl$_3$): δ 142.9, 142.6, 140.7, 129.6, 128.45, 128.4, 127.1, 125.8, 57.2, 42.8, 35.9, 31.7, 27.8, 23.7, 21.6 ppm

HRMS (ESI+): calc'd for $C_{20}H_{27}NO_2SNa$ [M+Na]$^+$: 368.1660, found 368.1650.

4-Methyl-N-(1-phenylethyl)benzenesulfonamide (44a)

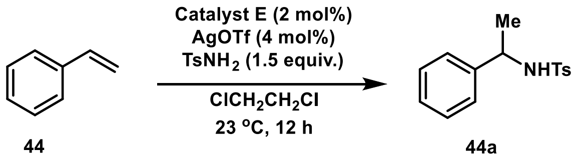

The general procedure I was followed and the reaction mixture was stirred at 23 °C for 12 h. The crude residue was purified by column chromatography (EtOAc/hexane= 1/20 to 1/3) to yield **44a** (26.7 mg, 97% yield) as a white solid.

^{1}H NMR (600 MHz, CDCl$_3$): δ 7.62 (d, J = 7.7 Hz, 2H), 7.18 (t, J = 7.3 Hz, 5H), 7.10 (d, J = 7.2 Hz, 2H), 5.00 (d, J = 6.8 Hz, 1H), 4.46 (p, J = 6.8 Hz, 1H), 2.38 (s, 3H), 1.42 (d, J = 6.8 Hz, 3H) ppm

^{13}C NMR (151 MHz, CDCl$_3$): δ 143.2, 142.2, 137.8, 129.6, 128.7, 127.6, 127.2, 126.2, 53.8, 23.7, 21.6 ppm

HRMS (ESI+): calc'd for $C_{15}H_{17}NO_2SNa$ [M+Na]$^+$: 298.0878, found 298.0870.

***N*-((1*S*,2*S*,4*R*)-bicyclo[2.2.1]heptan-2-yl)-4-methylbenzenesulfonamide (23f)**

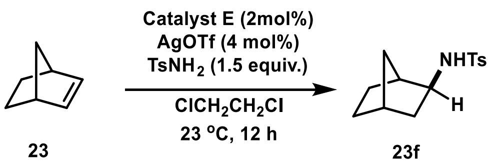

The general procedure I was followed and the reaction mixture was stirred at 23 °C for 12 h. The crude residue was purified by column chromatography (EtOAc/hexane= 1/20 to 1/5) to yield **23f** (26.1 mg, 98% yield) as a white solid.

^{1}H NMR (600 MHz, CDCl$_3$): δ 7.75 (d, J = 7.7 Hz, 2H), 7.30 (d, J = 7.7 Hz, 2H), 4.51 (d, J = 6.2 Hz,

1H), 3.12 (s, 1H), 2.43 (s, 3H), 2.19 (s, 1H), 2.10 (s, 1H), 1.58 (d, *J* = 9.4 Hz, 1H), 1.48 – 1.34 (m, 2H), 1.31 (d, *J* = 10.1 Hz, 1H), 1.15 (d, *J* = 13.2 Hz, 1H), 1.11 (d, *J* = 10.1 Hz, 1H), 1.08 – 0.96 (m, 2H) ppm

^{13}C NMR (151 MHz, $CDCl_3$): δ 143.4, 138.1, 129.8, 127.2, 56.8, 42.6, 41.0, 35.7, 35.3, 28.1, 26.5, 21.7 ppm

HRMS (ESI+): calc'd for $C_{14}H_{20}NO_2S$ $[M+H]^+$: 266.1215, found 266.1205.

***N*-((1*S*,2*S*,4*R*)-bicyclo[2.2.1]heptan-2-yl)-*N*,4-dimethylbenzenesulfonamide (23g)**

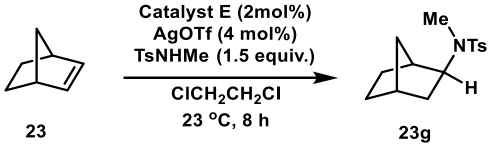

23 23g

The general procedure I was followed and the reaction mixture was stirred at 23 °C for 8 h. The crude residue was purified by column chromatography (EtOAc/hexane= 1/20 to 1/5) to yield **23g** (25.1 mg, 90% yield) as a white solid.

^{1}H NMR (400 MHz, $CDCl_3$): δ 7.66 (d, *J* = 8.3 Hz, 2H), 7.29 (d, *J* = 8.3 Hz, 2H), 3.83 (dd, *J* = 8.5, 5.9 Hz, 1H), 2.71 (s, 3H), 2.42 (s, 3H), 2.20 (s, 1H), 1.87 (s, 1H), 1.55 (ddd, *J* = 12.8, 8.5, 2.2 Hz, 1H), 1.45 – 1.32 (m, 4H), 1.21 – 1.11 (m, 1H), 1.12 – 1.00 (m, 2H) ppm

^{13}C NMR (101 MHz, $CDCl_3$): δ 143.1, 136.4, 129.7, 127.4, 60.0, 39.24, 36.9, 36.8, 35.9, 30.0, 29.3, 27.4, 21.6 ppm

HRMS (ESI+): calc'd for $C_{15}H_{22}NO_2S$ $[M+H]^+$: 280.1371, found 280.1362.

***N*-((1*S*,2*S*,4*R*)-bicyclo[2.2.1]heptan-2-yl)-*N*-(4-methoxyphenyl)-4-methylbenzenesulfonamide (23h)**

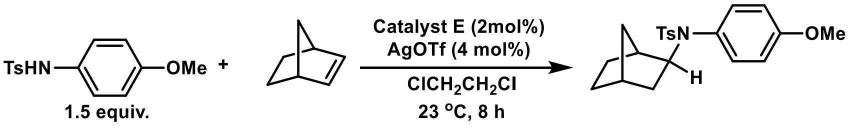

23 23h

The general procedure I was followed and the reaction mixture was stirred at 23 °C for 8 h. The crude residue was purified by column chromatography (EtOAc/hexane= 1/25 to 1/7) to yield **23h** (35.9 mg, 97% yield) as a white solid.

^{1}H NMR (400 MHz, $CDCl_3$): δ 7.58 (d, *J* = 8.3 Hz, 2H), 7.25 (d, *J* = 8.3 Hz, 2H), 6.92 – 6.85 (m, 2H), 6.83 – 6.75 (m, 2H), 4.11 (dd, *J* = 8.1, 5.3 Hz, 1H), 3.79 (s, 3H), 2.42 (s, 3H), 2.21 (d, *J* = 3.0 Hz, 1H), 2.03 (s, 1H), 1.70 (ddd, *J* = 12.8, 8.4, 2.1 Hz, 1H), 1.53 (ddd, *J* = 8.0, 4.6, 2.5 Hz, 1H), 1.40 (ddd, *J* = 10.1, 8.0, 3.8 Hz, 1H), 1.29 – 1.17 (m, 1H), 1.17 – 1.02 (m, 1H), 0.79 (dd, *J* = 9.9, 0.8 Hz, 1H), 0.73 – 0.59 (m, 1H) ppm

^{13}C NMR (101 MHz, $CDCl_3$): δ 159.5, 143.1, 137.8, 133.5, 129.5, 127.8, 114.0, 62.3, 55.5, 42.0, 38.8, 35.7, 28.8, 28.1, 21.7 ppm

HRMS (ESI+): calc'd for $C_{21}H_{26}NO_3S$ $[M+H]^+$: 372.1628, found 372.1627.

N-cyclooctyl-4-methylbenzenesulfonamide (45a)

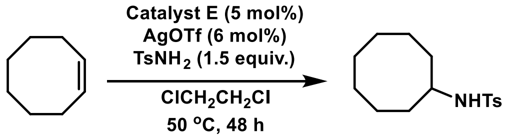

The general procedure III was followed and the reaction mixture was stirred at 50 °C for 48 h. The crude residue was purified by column chromatography (EtOAc/hexane= 1/20 to 1/5) to yield **45a** (18.3 mg, 65% yield) as a white solid.

^{1}H NMR (400 MHz, $CDCl_3$): δ 7.75 (d, J = 8.3 Hz, 2H), 7.29 (d, J = 8.3 Hz, 2H), 4.31 (d, J = 7.7 Hz, 1H), 3.37 (dq, J = 12.5, 4.3 Hz, 1H), 2.43 (s, 3H), 1.80 – 1.63 (m, 2H), 1.63 – 1.33 (m, 12H) ppm

^{13}C NMR (151 MHz, $CDCl_3$): δ 143.3, 138.4, 129.8, 127.1, 54.0, 32.9, 27.3, 25.4, 23.3, 21.7 ppm

The spectroscopic data were in accordance with those reported in literature.[25]

6. Catalytic Enantioselective Hydrofunctionnization

6.1 General Procedure for Chiral Catalyst Screening by Hydroalkoxylation of 21

2,2-Diphenylpent-4-en-1-ol **21** was selected as model substrate and the screening was performed in an argon filled-glovebox. 9 Chiral precatalysts and 4 monophosphine ligands were investigated.

In an argon filled glovebox, to a 4 mL vial with a magnetic stir bar were added the precatalyst (0.002mmol, 2 mol%), silver trifluoromethanesulfonate (1.5 mg, 0.006 mmol, 6 mol%), $ClCH_2CH_2Cl$ (200 μL). The mixture was stirred at room temperature (23 °C) for 1 h. And monophosphine ligand (0.002mmol, 2 mol%) dissolved in $ClCH_2CH_2Cl$ (100 μL) was added to the above mixture. The resulting solution was stirred for 5 min and **21** (24 mg, 0.1 mmol, 1.0 equiv) was added. The mixture was stirred at 50 °C for 48 h. The reaction mixture was concentrated under reduced pressure and the crude product was purified by silica gel column chromatography using hexane/ ethyl acetate (20/1) to provide the product **21a**.

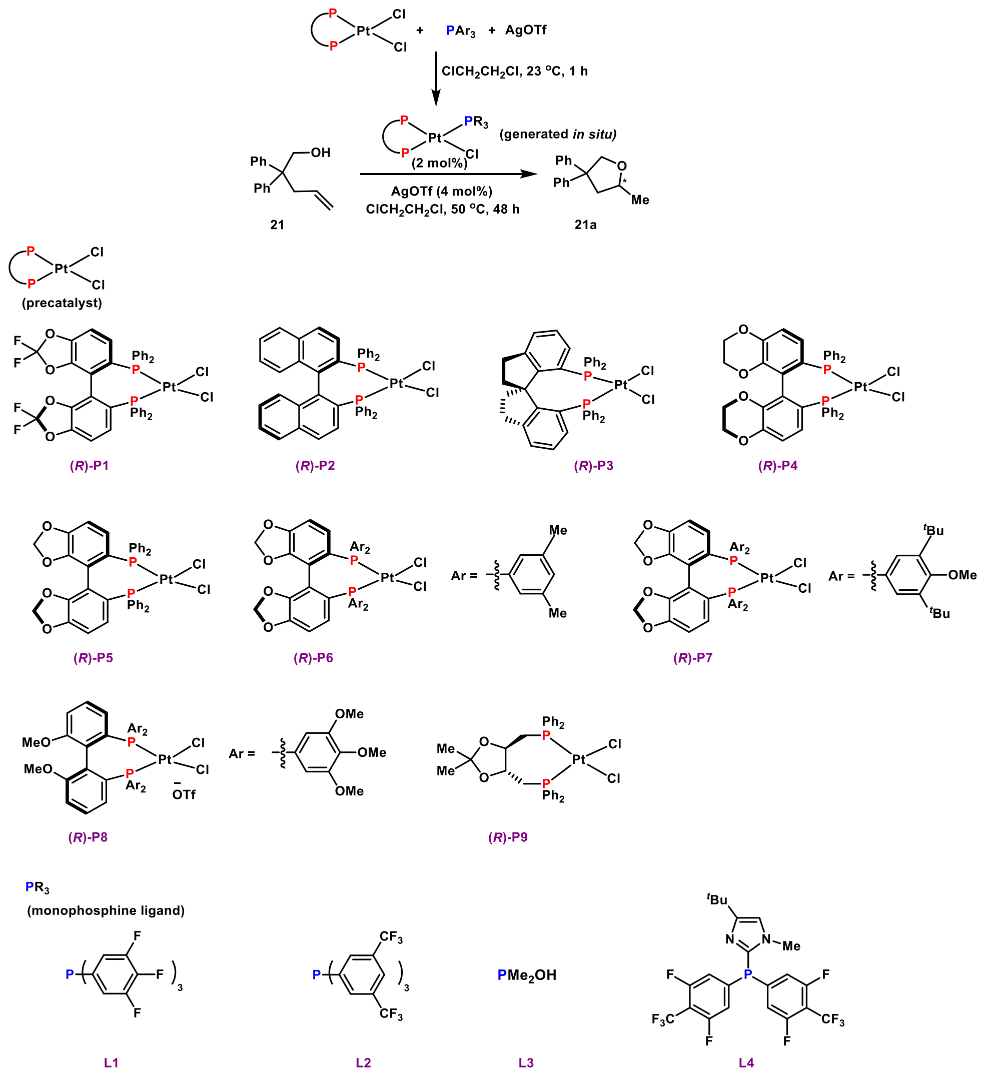

Supplementary Figure 2. Chiral Pre-catalyst and Monodentate Phosphine Ligand

Supplementary Table 5: Chiral Catalysts Screening by Hydroalkoxylation of **21**

Entry	Precatalyst	Monophosphine Ligand	Yield (%)	ee (%)
1	P1	L1	20	0
2	P2	L1	36	0
3	P3	L1	40	0
4	P4	L1	trace (21)[b]	-- (0)[b]
5	P5	L1	38	0
6	P6	L1	15	0
7	P7	L1	trace (20)[b]	-- (0)[b]
8	P8	L1	25	0
9	P9	L1	N.R.	--
10	P1	L2	18	0
11	P2	L2	24	0
12	P9	L3	N.R.	--
13	P1	L4	35[b]	35[b]
14	P2	L4	44[b]	35[b]
15	P2	--	trace	--

[a] Yields of isolated products are given. [b]5 mol% catalyst was generated in situ (Precatalyst (5 mol%), L (5 mol%), AgOTf (12 mol%)), $ClCH_2CH_2Cl$, 50 °C, 48 h.

HPLC (AD-H, 0.46*25 cm, 5μm, hexane / isopropylalcohol = 99/1, flow 0.7 mL/min, detection at 254 nm)

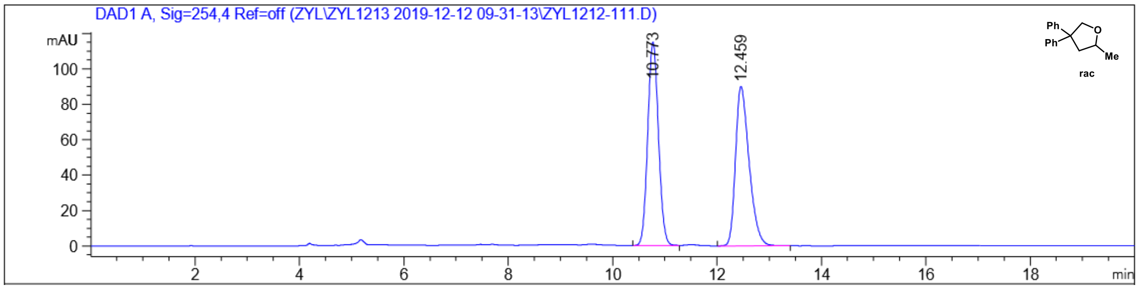


```
Signal 1: DAD1 A, Sig=254,4 Ref=off

Peak RetTime Type  Width     Area      Height     Area
  #    [min]        [min]   [mAU*s]     [mAU]       %
----|-------|----|-------|----------|----------|--------|
   1  10.773 BB     0.2221 1634.30518  114.82312  50.1018
   2  12.459 BB     0.2750 1627.66626   90.00819  49.8982

Totals :                   3261.97144  204.83131
```

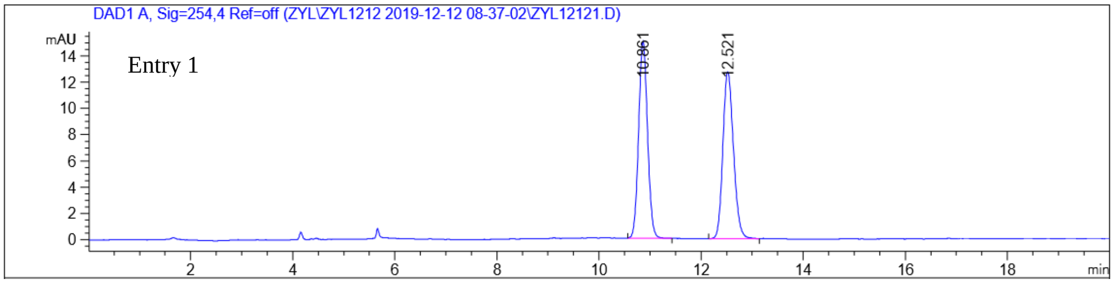

Signal 1: DAD1 A, Sig=254,4 Ref=off

Peak #	RetTime [min]	Type	Width [min]	Area [mAU*s]	Height [mAU]	Area %
1	10.861	BB	0.1875	181.87561	14.99074	49.7890
2	12.521	BB	0.2242	183.41747	12.71885	50.2110
Totals :				365.29308	27.70959	

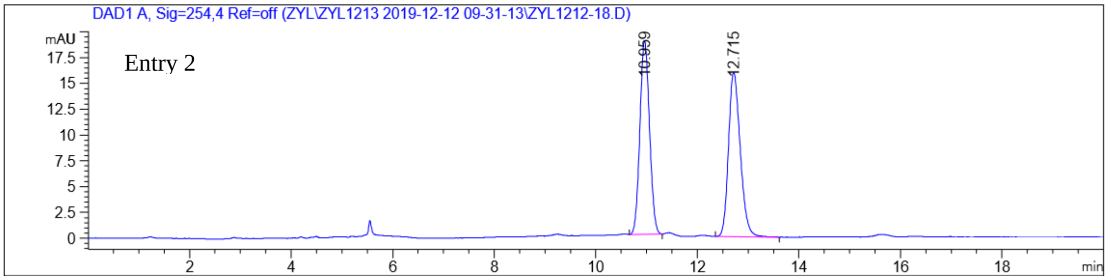

Signal 1: DAD1 A, Sig=254,4 Ref=off

Peak #	RetTime [min]	Type	Width [min]	Area [mAU*s]	Height [mAU]	Area %
1	10.959	BB	0.2053	246.17366	18.73603	49.0227
2	12.715	BB	0.2475	255.98851	15.92771	50.9773
Totals :				502.16217	34.66375	

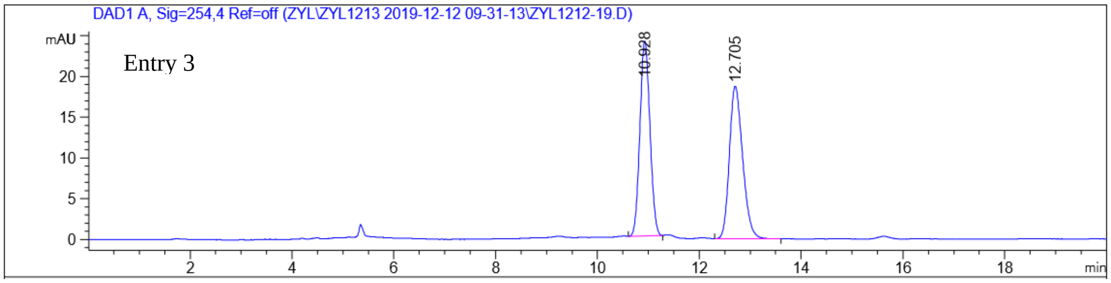

Signal 1: DAD1 A, Sig=254,4 Ref=off

Peak #	RetTime [min]	Type	Width [min]	Area [mAU*s]	Height [mAU]	Area %
1	10.928	BB	0.2141	327.93478	23.89274	49.2610
2	12.705	BB	0.2807	337.77332	18.70465	50.7390
Totals :				665.70810	42.59739	

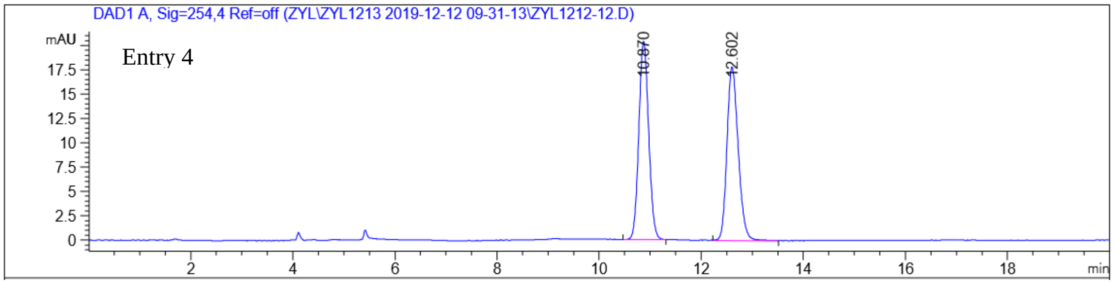

Signal 1: DAD1 A, Sig=254,4 Ref=off

Peak #	RetTime [min]	Type	Width [min]	Area [mAU*s]	Height [mAU]	Area %
1	10.870	BB	0.2103	273.16055	20.38731	50.0295
2	12.602	BB	0.2364	272.83859	17.84177	49.9705
Totals :				545.99915	38.22907	

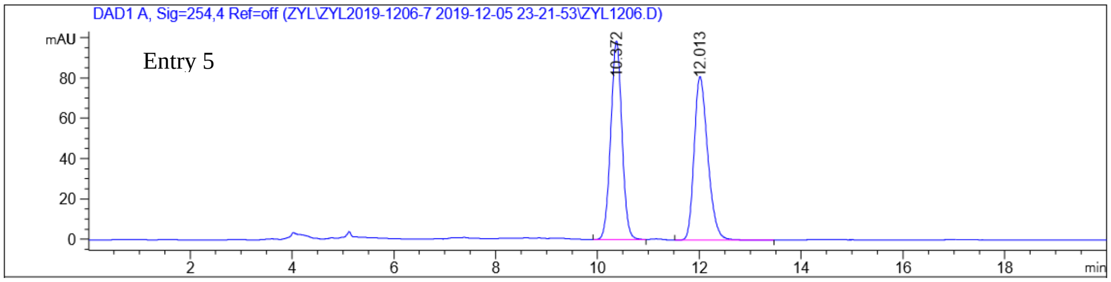

Signal 1: DAD1 A, Sig=254,4 Ref=off

Peak #	RetTime [min]	Type	Width [min]	Area [mAU*s]	Height [mAU]	Area %
1	10.372	BB	0.2371	1509.30530	98.34425	50.0029
2	12.013	BB	0.2852	1509.12976	81.04923	49.9971
Totals :				3018.43506	179.39349	

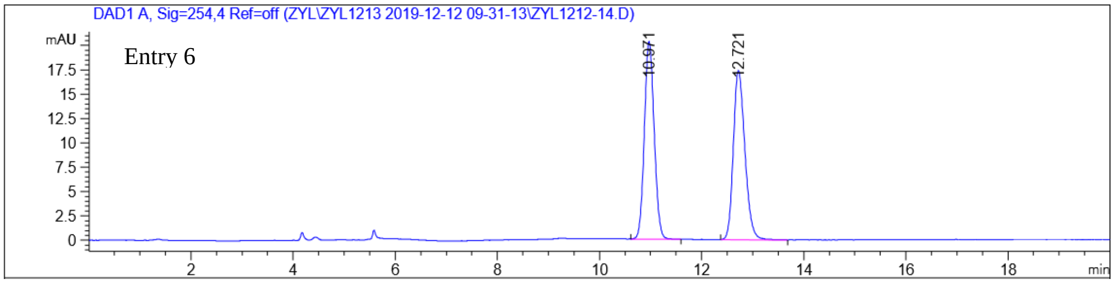

Signal 1: DAD1 A, Sig=254,4 Ref=off

Peak #	RetTime [min]	Type	Width [min]	Area [mAU*s]	Height [mAU]	Area %
1	10.971	BB	0.2079	271.35812	20.30522	49.8231
2	12.721	BB	0.2436	273.28510	17.36303	50.1769
Totals :				544.64322	37.66825	

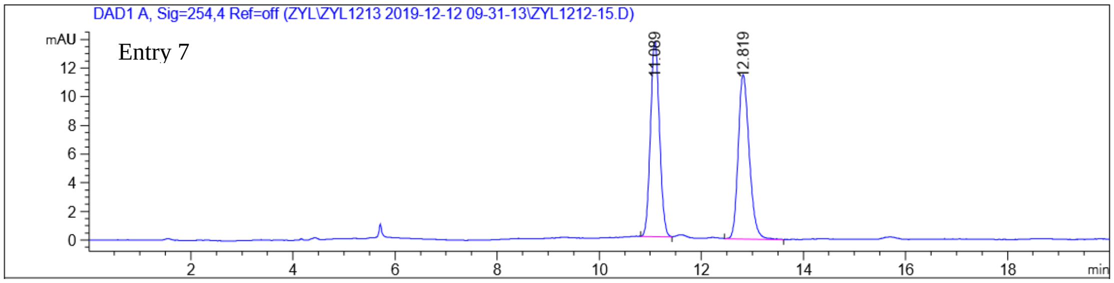

Signal 1: DAD1 A, Sig=254,4 Ref=off

Peak #	RetTime [min]	Type	Width [min]	Area [mAU*s]	Height [mAU]	Area %
1	11.089	BB	0.1908	166.52278	13.60602	49.2429
2	12.819	BB	0.2308	171.64363	11.45737	50.7571
Totals :				338.16641	25.06338	

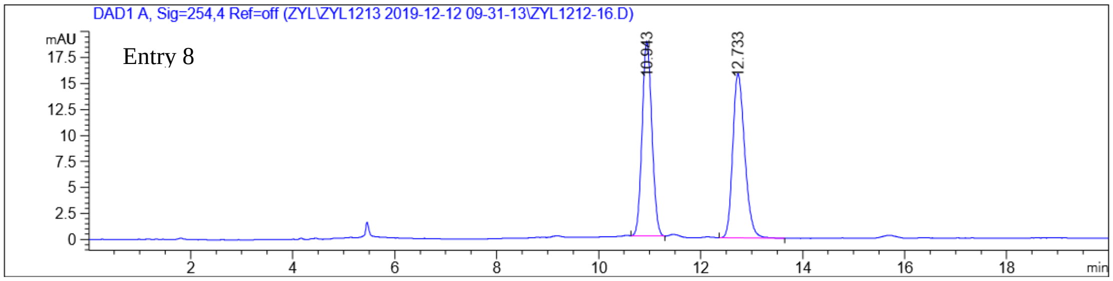

Signal 1: DAD1 A, Sig=254,4 Ref=off

Peak #	RetTime [min]	Type	Width [min]	Area [mAU*s]	Height [mAU]	Area %
1	10.943	BB	0.2073	249.58208	18.74781	49.1462
2	12.733	BB	0.2525	258.25430	15.81542	50.8538
Totals :				507.83638	34.56324	

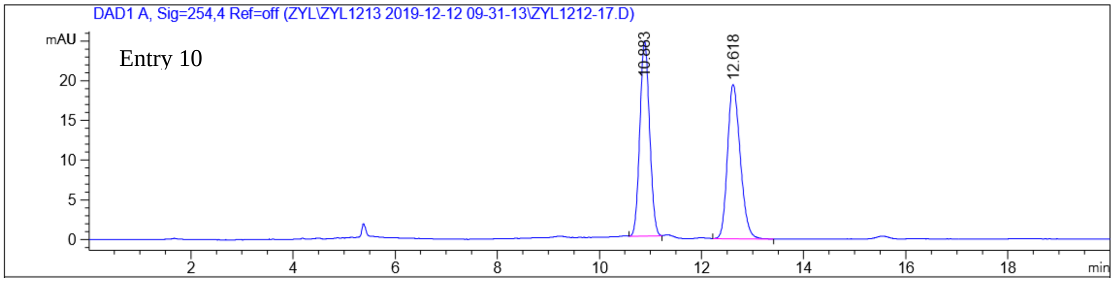

Signal 1: DAD1 A, Sig=254,4 Ref=off

Peak #	RetTime [min]	Type	Width [min]	Area [mAU*s]	Height [mAU]	Area %
1	10.883	BB	0.2082	324.20340	24.52976	49.0294
2	12.618	BB	0.2685	337.03900	19.42263	50.9706
Totals :				661.24240	43.95240	

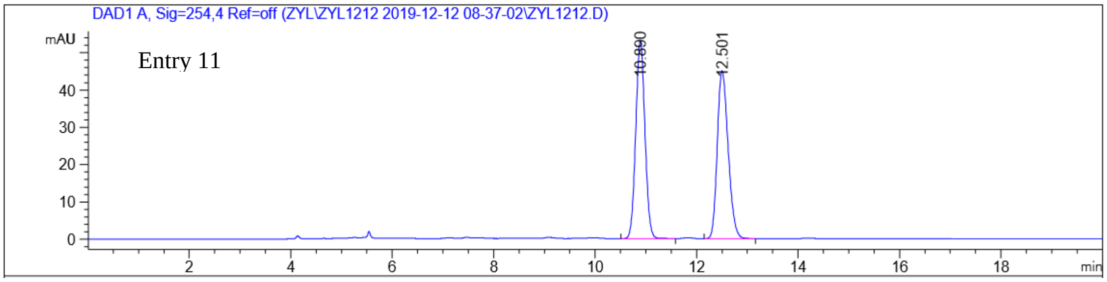

Signal 1: DAD1 A, Sig=254,4 Ref=off

Peak #	RetTime [min]	Type	Width [min]	Area [mAU*s]	Height [mAU]	Area %
1	10.890	BB	0.1954	670.22284	53.04930	49.9197
2	12.501	BB	0.2319	672.37891	45.11346	50.0803
Totals :				1342.60175	98.16276	

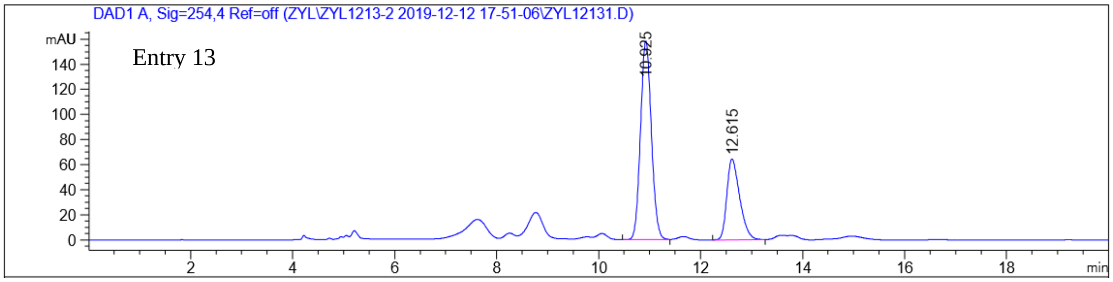

Signal 1: DAD1 A, Sig=254,4 Ref=off

Peak #	RetTime [min]	Type	Width [min]	Area [mAU*s]	Height [mAU]	Area %
1	10.925	BB	0.2304	2338.62427	158.22594	67.5249
2	12.615	BB	0.2654	1124.72827	64.50167	32.4751
Totals :				3463.35254	222.72761	

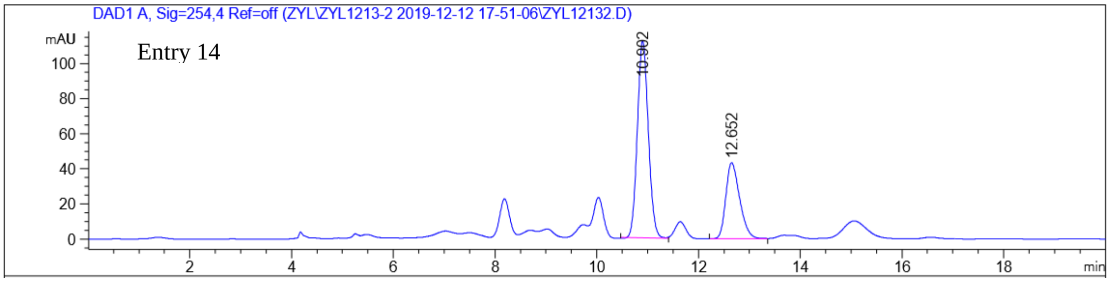

Signal 1: DAD1 A, Sig=254,4 Ref=off

Peak #	RetTime [min]	Type	Width [min]	Area [mAU*s]	Height [mAU]	Area %
1	10.902	MM	0.2505	1686.37671	112.20116	67.3360
2	12.652	BB	0.2882	818.04419	43.35111	32.6640
Totals :				2504.42090	155.55228	

6.2 Control Experiments

Procedure: In an argon filled glovebox, to a 4 mL vial with a magnetic stir bar were added the precatalyst (0.002mmol, 5 mol%), silver trifluoromethanesulfonate (0.01 mmol, 10 mol%), $ClCH_2CH_2Cl$ (200 μL). The resulting mixture was stirred at room temperature (23 ^{o}C) for 1 h. And monophosphine ligand **L4**,

HOTf, **1** (24 mg, 0.1 mmol, 1.0 equiv) and 4,4'-di-*tert*-butyl-1,1'-biphenyl (0.5 equiv.) used as an internal standard were added into the above mixture. The mixture was stirred at 50 °C for 48 h. The reaction mixture was concentrated under reduced pressure and the crude product was purified by silica gel column chromatography using hexane/ ethyl acetate (20/1) to provide the product **21a**. The yield of **1a** was determined by crude ^{1}H NMR analysis.

Supplementary Table 6: The Effect of HOTf on Catalytic Activity and Enantioselectivity

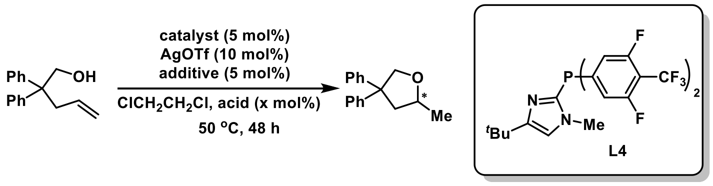

Entry	catalyst	additive	HOTf(x mol%)	Yield (%)[a]	ee (%)
1	(BINAP)PtCl$_2$	L4	0	27	30
2	(BINAP)PtCl$_2$	L4	5	50	13
3	BINAP	L4	0	0	--
4	BINAP	L4	5	91	0
5	(BINAP)PtCl$_2$	--	0	<5	--

HPLC (AD-H, 0.46*25 cm, 5μm, hexane / isopropylalcohol = 99/1, flow 0.7 mL/min, detection at 254 nm)

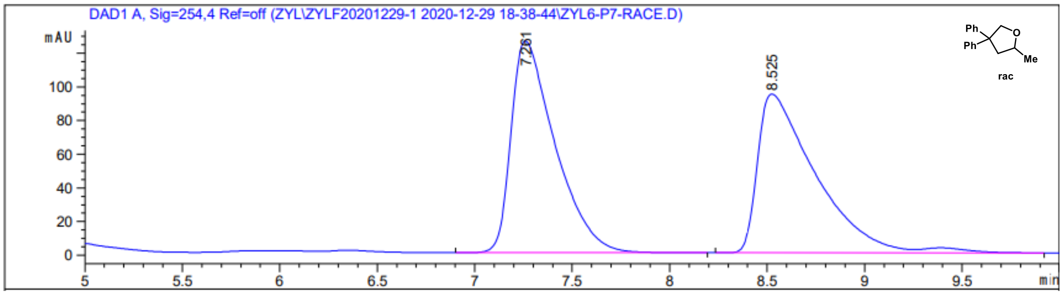


```
Signal 1: DAD1 A, Sig=254,4 Ref=off

Peak RetTime Type  Width     Area      Height     Area
  #    [min]        [min]   [mAU*s]     [mAU]       %
----|-------|----|-------|----------|----------|--------|
   1   7.261 BB     0.2310 1972.47388  125.76474  50.1004
   2   8.525 BV R   0.2924 1964.56946   94.24731  49.8996
```

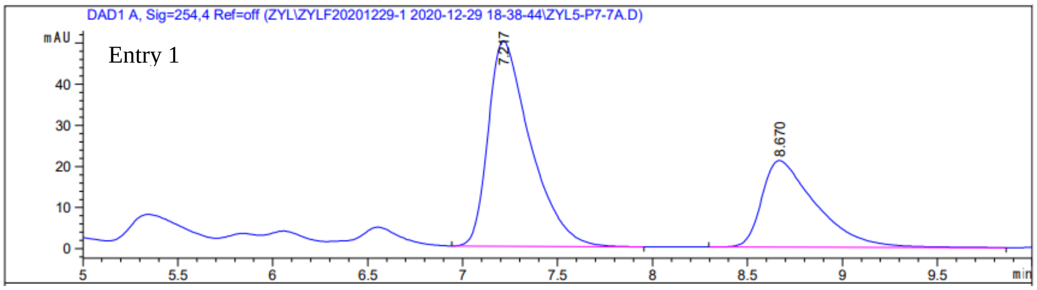

Signal 1: DAD1 A, Sig=254,4 Ref=off

Peak #	RetTime [min]	Type	Width [min]	Area [mAU*s]	Height [mAU]	Area %
1	7.217	BB	0.2283	770.54462	49.86490	64.8991
2	8.670	BB	0.2849	416.75110	21.05275	35.1009

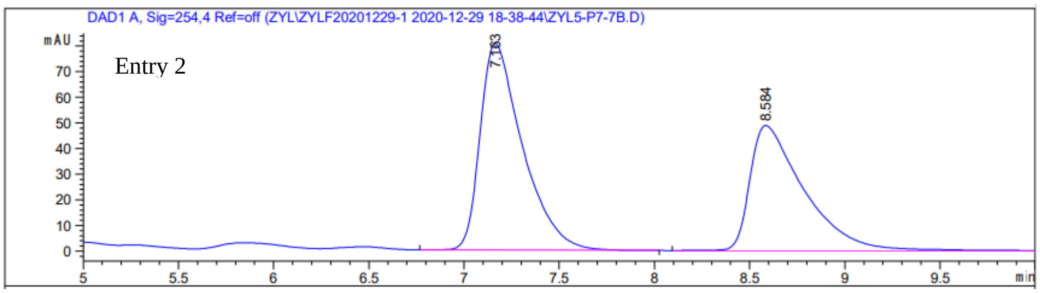

Signal 1: DAD1 A, Sig=254,4 Ref=off

Peak #	RetTime [min]	Type	Width [min]	Area [mAU*s]	Height [mAU]	Area %
1	7.163	BB	0.2286	1248.29028	80.65449	56.6351
2	8.584	BB	0.2825	955.80389	48.79872	43.3649

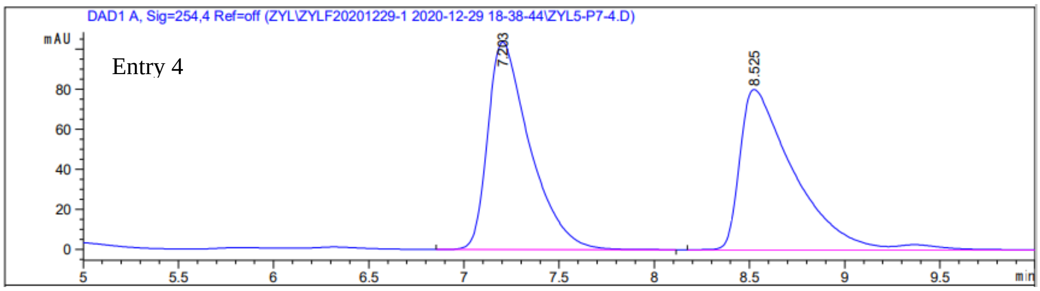

Signal 1: DAD1 A, Sig=254,4 Ref=off

Peak #	RetTime [min]	Type	Width [min]	Area [mAU*s]	Height [mAU]	Area %
1	7.203	BB	0.2268	1584.15149	103.35302	50.0544
2	8.525	BV R	0.2803	1580.70898	79.84180	49.9456

6.3 Synthesis of the Chiral Catalysts M and N

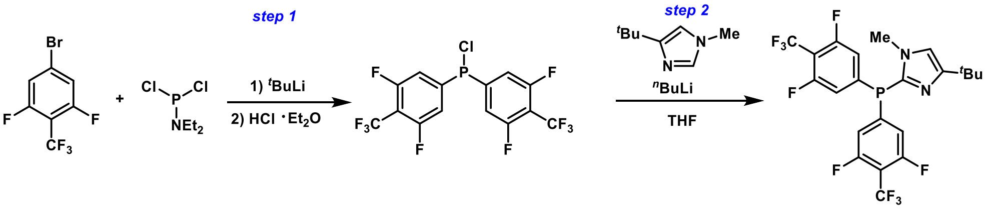

L4

Step 1: Chlorobis(3,5-difluoro-4-(trifluoromethyl)phenyl)phosphane was prepared according to literature[21]. To a suspension of 5-bromo-1,3-difluoro-2-(trifluoromethyl)benzene (6.26 g, 24 mmol) in Et_2O (20 mL) at -78 °C under argon was added dropwise *t*-BuLi (18.5 mL, 1.3 M in hexanes, 24 mmol).

The reaction was stirred at -78 °C for 1 h. The solution of 1,1-dichloro-*N*,*N*-diethylphosphanamine (1.91 g, 11 mmol) dissolved in Et_2O was added dropwise. The mixture was stirred at room temperature (23 °C) for 12 h. The reaction was quenched with aqueous NH_4Cl (50 mL) and extracted with ether (2 x 60 mL). The combined organic layers were dried over anhydrous Na_2SO_4, and concentrated under vacuum. The crude product was dissolved in Hexane at -10 °C. A solution of HCl in Et_2O (3 equiv., 1.45 M in Et_2O) was added. The mixture was stirred -10 °C for 2 h. The solution was filtered through a pad of celite and concentrated. The crude product used in the next step without further purification.

Step 2: Monophosphine ligand **L4** was prepared according to literature[22]. To a suspension of 4-(*tert*-butyl)-1-methyl-1*H*-imidazole in Et_2O (20 mL) at -78 °C under argon was added dropwise *n*-BuLi (4.2 mL, 2.4 M in hexanes, 10 mmol). The reaction was stirred at -78 °C for 1 h. The solution of chlorobis(3,5-difluoro-4-(trifluoromethyl)phenyl)phosphane (2.57 g, 6 mmol) dissolved in THF was added dropwise. The mixture was stirred at 23 °C for 2 h. The reaction was quenched with aqueous NH_4Cl (50 mL) and extracted with ether (2 x 60 mL). The combined organic layers were dried over anhydrous Na_2SO_4, and concentrated under vacuum. The crude product was purified by silica gel column chromatography using hexane/ ethyl acetate (20/1) to provide the product **L4** 2-(bis(3,5-difluoro-4-(trifluoromethyl)phenyl)phosphanyl)-4-(*tert*-butyl)-1-methyl-1*H*-imidazole (2.50 g, 79%) as a pale yellow solid.

^{1}H NMR (400 MHz, CDCl$_3$): δ 7.37 – 7.32 (m, 4H), 6.84 (d, *J* = 3.6 Hz, 1H), 3.84 (s, 3H), 1.30 (s, 9H) ppm

^{13}C NMR (151 MHz, CDCl$_3$): δ 159.60 (dd, *J* = 263.7, 9.1 Hz), 156.1 (d, *J* = 3.0 Hz), 143.9 (dd, *J* = 18.3, 8.0 Hz), 139.2 (d, *J* = 6.8 Hz), 121.6 (q, *J* = 274.3 Hz), 119.1 (d, *J* = 1.6 Hz), 117.8 – 117.5 (m), 108.9 – 108.2 (m), 34.0, 32.3, 30.2 ppm

^{31}P NMR (162 MHz, CDCl$_3$): δ -37.5 ppm

^{19}F NMR (376 MHz, CDCl$_3$): δ -56.5 (t, *J* = 22.1 Hz), -109.7 (q, *J* = 22.0 Hz) ppm

HRMS (ESI+): calc'd for $C_{22}H_{18}F_{10}N_2P$ $[M+H]^+$: 531.1042, found 531.1037.

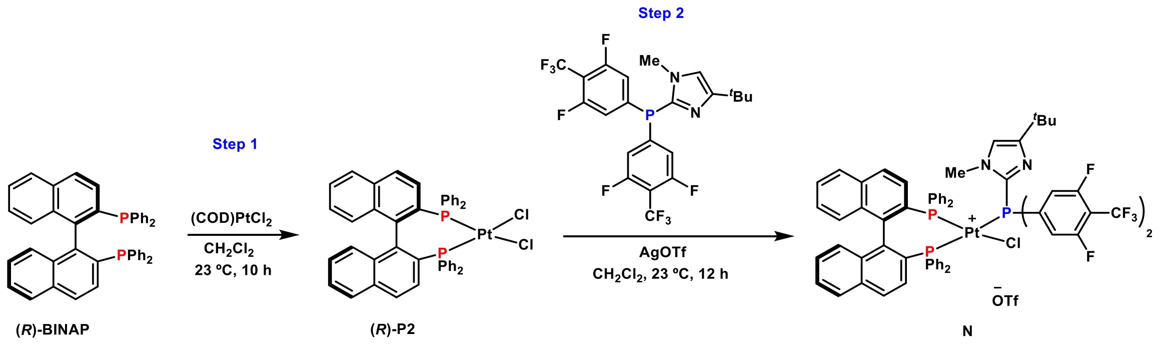

Step 1: In an argonfilled glovebox, to a 20 mL scintillation vial with a magnetic stir bar were added (R)-BINAP(200 mg, 0.32 mmol), (COD)$PtCl_2$ (120 mg, 0.32 mmol) and 3 mL CH_2Cl_2, then the vial was taken outside of the dry box and the resulting mixture was stirred at room temperature (23 °C) for 10 h. The yellow solution was filtered and then evaporated to provide yellow solid, which was recrystallized through 1 mL CH_2Cl_2 and 1 mL hexane to give yellow precipitate. The solid was collected and dried in *vacuo* to yield **(*R*)-P2** (257 mg, 91% yield).

Step 2: In an argon filled glovebox, to a 4 mL vial with a magnetic stir bar were added **(*R*)-P2** (100 mg, 0.11 mmol), silver trifluoromethanesulfonate (29 mg, 0.11 mmol), 2-(bis(3,5-difluoro-4-(trifluoromethyl)phenyl)phosphanyl)-4-(*tert*-butyl)-1-methyl-1H-imidazole (65 mg, 0.12 mmol) and 2 mL CH_2Cl_2. Then the vial was taken outside of the glovebox and the resulting mixture was stirred at room 23 °C for 12 h. The orange solution was filtered and CH_2Cl_2 was evaporated to provide pale yellow solid, which was recrystallized through CH_2Cl_2 and hexane to give white precipitate. The solid was collected and dried in vacuo to yield catalyst **N** (168 mg, 98% yield).

^{1}H NMR (600 MHz, $CDCl_3$): δ 8.30 (s, 1H), 7.76 (d, *J* = 8.0 Hz, 1H), 7.68 – 7.63 (m, 2H), 7.62 (d, *J* = 8.3 Hz, 1H), 7.59 – 7.54 (m, 2H), 7.54 – 7.49 (m, 2H), 7.47 – 7.28 (m, 17H), 7.13 – 7.09 (m, 1H), 7.09 – 7.05 (m, 1H), 6.98 – 6.90 (m, 3H), 6.85 – 6.84 (m, 3H), 6.57 (d, *J* = 8.6 Hz, 1H), 6.50 (d, *J* = 8.5 Hz, 1H), 6.44 (s, 1H), 3.83 (s, 3H), 1.51 (s, 9H) ppm

^{31}C NMR (150 MHz, $CDCl_3$): δ 160.1 –157.4 (m), 155.7 (d, *J* = 11.7 Hz), 140.6 – 140.5 (m), 138.7 – 138.6 (m), 136.2 – 136.0 (m), 135.2 (d, *J* = 10.1 Hz), 134.5 (d, *J* = 20.5 Hz), 134.1 – 134.0 (m), 133.2 – 132.6 (m), 132.1, 131.6 (d, *J* = 9.3 Hz), 130.9 (d, *J* = 10.6 Hz), 130.0 (d, *J* = 9.4 Hz), 129.5 (d, *J* = 11.1 Hz), 128.9 (d, *J* = 34.8 Hz), 128.5 (d, *J* = 34.1 Hz), 128.2 (d, *J* = 11.3 Hz), 127.8 (d, *J* = 10.5 Hz), 127.5 (d, *J* = 14.1 Hz), 127.3 (d, *J* = 7.2 Hz), 127.0 (d, *J* = 6.9 Hz), 126.6 (d, *J* = 5.6 Hz), 123.0, 121.0 (q, *J* = 319.5 Hz), 120.8 (q, *J* = 272.7 Hz), 120.0 – 119.5 (m), 118.7, 118.4 – 118.2 (m), 37.7, 32.4, 30.2 ppm

^{31}P NMR (243 MHz, $CDCl_3$): δ 16.8 (d, $^2J_{P-P}$ = 21 Hz, $^1J_{Pt-P}$ = 2760 Hz), 15.0 (d, $^2J_{P-P}$ = 21 Hz, $^1J_{Pt-P}$ = 2762 Hz), 7.6 (t, $^2J_{P-P}$ = 18 Hz, $^1J_{Pt-P}$ = 3554 Hz) ppm

^{19}F NMR (565 MHz, $CDCl_3$): δ -56.7 – 57.0 (m), -77.9, -108.1 (d, *J* = 20.0 Hz) ppm

HRMS (ESI+): calc'd for $C_{66}H_{49}ClF_{10}N_2P_3Pt$ [M-OTf]$^+$: 1382.2280, found 1382.2275.

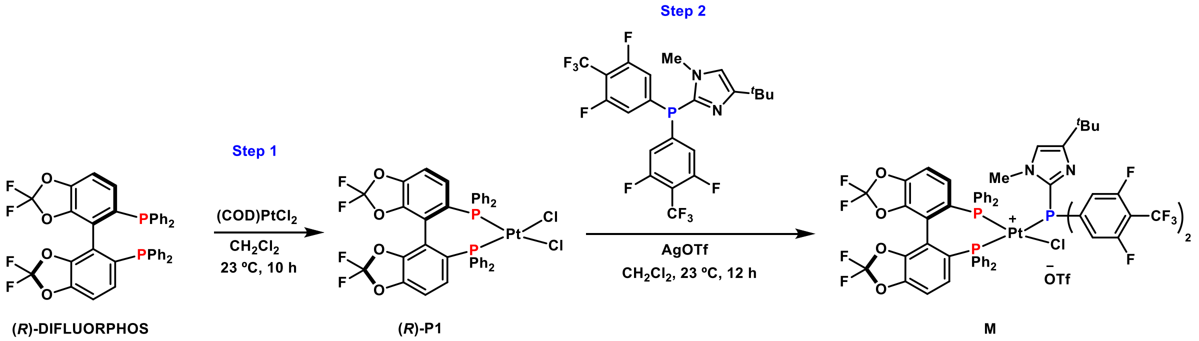

Step 1: In an argonfilled glovebox, to a 20 mL scintillation vial with a magnetic stir bar were added (R)-DIFLUORPHOS (100 mg, 0.15 mmol), (COD)$PtCl_2$ (55 mg, 0.15 mmol) and 1 mL CH_2Cl_2, then the vial was taken outside of the dry box and the resulting mixture was stirred at 23 °C for 10 h. The yellow solution was filtered and then evaporated to provide yellow solid, which was recrystallized through 1 mL CH_2Cl_2 and 1 mL hexane to give yellow precipitate. The solid was collected and dried in *vacuo* to yield **(*R*)-P1** (137 mg, 99% yield).

Step 2: In an argon filled glovebox, to a 4 mL vial with a magnetic stir bar were added **(*R*)-P1** (50 mg, 0.05 mmol), silver trifluoromethanesulfonate (14 mg, 0.05 mmol), 2-(bis(3,5-difluoro-4-(trifluoromethyl)phenyl)phosphanyl)-4-(*tert*-butyl)-1-methyl-1H-imidazole (31 mg, 0.06 mmol) and 1 mL CH_2Cl_2. Then the vial was taken outside of the glovebox and the resulting mixture was stirred at room 23 °C for 12 h. The orange solution was filtered and CH_2Cl_2 was evaporated to provide pale yellow solid, which was recrystallized through CH_2Cl_2 and Hexane to give white precipitate. The solid was collected and dried in vacuo to yield catalyst **M** (82 mg, 99% yield).

^{1}H NMR (400 MHz, CDCl$_3$): δ 8.61 (s, 1H), 7.66 – 7.35 (m, 21H), 7.22 (s, 2H), 7.09 – 7.02 (m, 4H), 6.85 (d, *J* = 8.3 Hz, 1H), 4.08 (s, 3H), 1.45 (s, 9H) ppm

^{13}C NMR (151 MHz, CDCl$_3$): δ 160.5 – 157.2 (m), 155.8 (d, *J* = 3.4 Hz), 146.2 (d, *J* = 12.5 Hz), 142.3 (d, *J* = 14.4 Hz), 142.0 (d, *J* = 12.3 Hz), 137.4 (d, *J* = 20.5 Hz), 134.7, 133.5, 133.2, 132.6, 132.3 (d, *J* = 9.5 Hz), 131.3 – 130.9 (m), 130.4 – 129.6 (m), 129.3 (d, *J* = 8.9 Hz), 128.8 (d, *J* = 10.3 Hz), 125.80 (d, *J* = 70.1 Hz), 123.9 – 123.1 (m), 122.1, 121.8, 121.5, 121.3 (q, *J* = 321.0 Hz), 121.1, 120.9 (q, *J* = 274.8 Hz), 120.8 (q, *J* = 274.8 Hz), 118.6 – 118.4 (m), 117.6 (dd, *J* = 9.9, 2.6 Hz), 115.5 (d, *J* = 12.0 Hz), 111.8 (d, *J* = 12.1 Hz), 110.5 (d, *J* = 10.4 Hz), 38.2, 32.5, 30.2 ppm

^{31}P NMR (243 MHz, CDCl$_3$): δ 16.3 (d, $^2J_{P\text{-}P}$ = 19 Hz, $^1J_{Pt\text{-}P}$ = 2714 Hz), 14.5 (d, $^2J_{P\text{-}P}$ = 19 Hz, $^1J_{Pt\text{-}P}$ = 2701 Hz), 6.27 (d, $^2J_{P\text{-}P}$ = 19 Hz, $^1J_{Pt\text{-}P}$ = 3052 Hz) ppm

^{19}F NMR (376 MHz, CDCl$_3$): δ -46.8 (dd, *J* = 185.0, 89.2 Hz), -49.9 (dd, *J* = 247.2, 89.3 Hz), -56. 8 – -57.0 (m), -78.0, -107.8 ppm

HRMS (ESI+): calc'd for $C_{60}H_{41}ClF_{14}N_2O_4P_3Pt$ [M-OTf]$^+$: 1442.1386, found 1442.1376.

6.4 General Procedure for Catalytic Enantioselective Hydroalkoxylation

In an argon filled glovebox, to a 4 mL vial with a magnetic stir bar were added the chiral catalyst **M** or **N** (0.005mmol, 5 mol%), silver trifluoromethanesulfonate (0.006 mmol, 6 mol%), $ClCH_2CH_2Cl$. The mixture was stirred at room temperature (23 °C) for 1 h. And 2,2-diphenylpent-4-en-1-ol **21** (24 mg, 0.1 mmol, 1.0 equiv) was added. The resulting mixture was stirred at 50 °C for 48 h. The reaction mixture was concentrated under reduced pressure and the crude product was purified by silica gel column chromatography using hexane/ ethyl acetate (20/1) to provide the product **21a**.

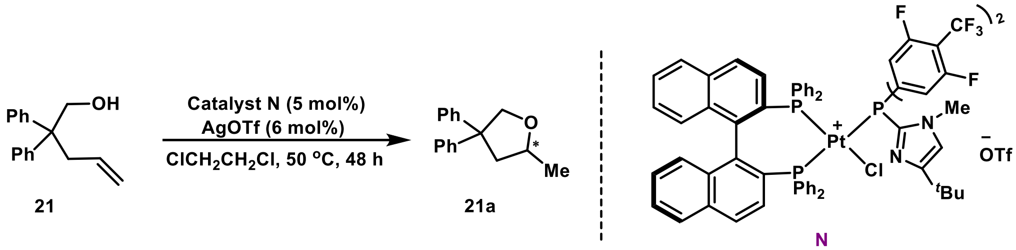

The desired product **21a** (10.0 mg, 42% yield, 36% ee) was obtained.

^{1}H NMR (400 MHz, $CDCl_3$): δ 7.32 – 7.28 (m, 6H), 7.23 – 7.17 (m, 4H), 4.61 – 4.58 (m, 1H), 4.22 – 4.13 (m, 2H), 2.64 (ddd, J = 12.1, 5.7, 0.9 Hz, 1H), 2.27 (dd, J = 12.1, 9.5 Hz, 1H), 1.59 (s, 1H), 1.30 (d, J = 6.1 Hz, 3H) ppm

^{13}C NMR (101 MHz, $CDCl_3$): δ 146.5, 146.3, 128.5, 128.4, 127.3, 126.5, 126.3, 77.0, 74.9, 56.5, 46.7, 21.5 ppm

The analytical data was consistent with the literature.[11]

HPLC (AD-H, 0.46*25 cm, 5μm, hexane / isopropyl alcohol = 99/1, flow 0.7 mL/min, detection at 254 nm) retention time = 10.900 min (major) and 12.681 min (minor).

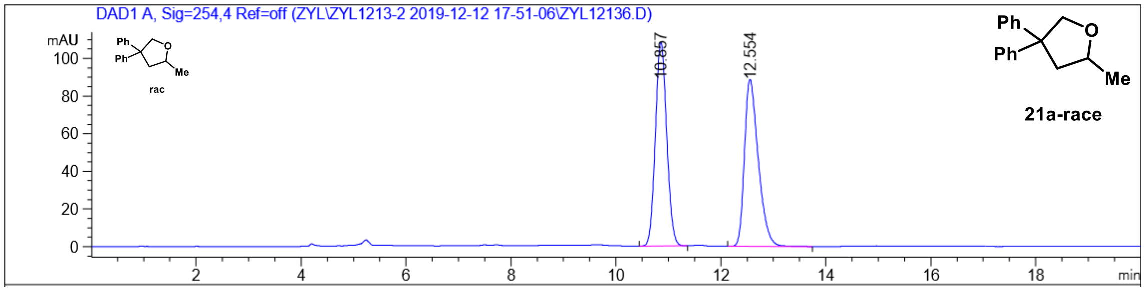

Signal 1: DAD1 A, Sig=254,4 Ref=off

Peak #	RetTime [min]	Type	Width [min]	Area [mAU*s]	Height [mAU]	Area %
1	10.857	BB	0.2351	1627.99878	108.46142	50.0790
2	12.554	BB	0.2772	1622.86401	88.81694	49.9210
Totals :				3250.86279	197.27836	

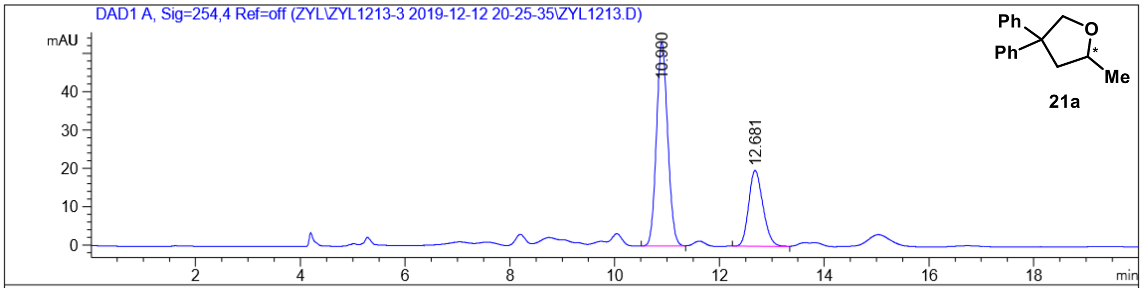

Signal 1: DAD1 A, Sig=254,4 Ref=off

Peak #	RetTime [min]	Type	Width [min]	Area [mAU*s]	Height [mAU]	Area %
1	10.900	BB	0.2323	794.20441	53.15425	68.0628
2	12.681	BB	0.2919	372.66541	19.76850	31.9372
Totals :				1166.86981	72.92275	

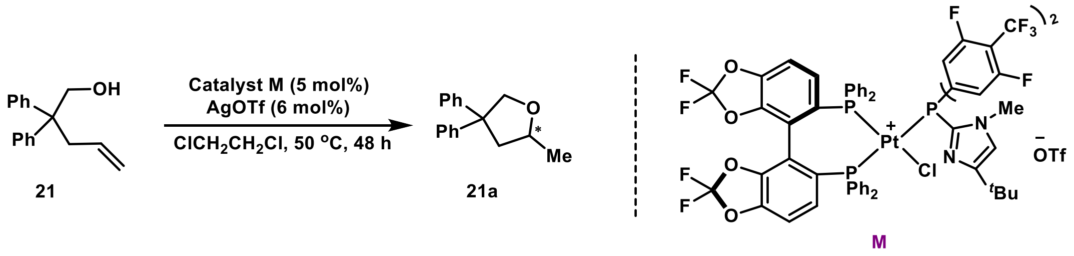

The desired product **21a** (7.9 mg, 33% yield, 33% ee) was obtained. HPLC (AD-H, 0.46*25 cm, 5μm, hexane / isopropyl alcohol = 99/1, flow 0.7 mL/min, detection at 254 nm) retention time = 10.930 min (major) and 12.777 min (minor)

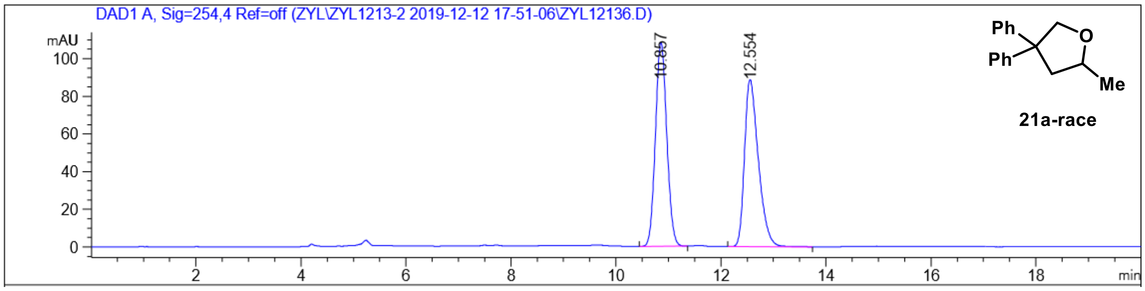

Signal 1: DAD1 A, Sig=254,4 Ref=off

Peak #	RetTime [min]	Type	Width [min]	Area [mAU*s]	Height [mAU]	Area %
1	10.857	BB	0.2351	1627.99878	108.46142	50.0790
2	12.554	BB	0.2772	1622.86401	88.81694	49.9210
Totals :				3250.86279	197.27836	

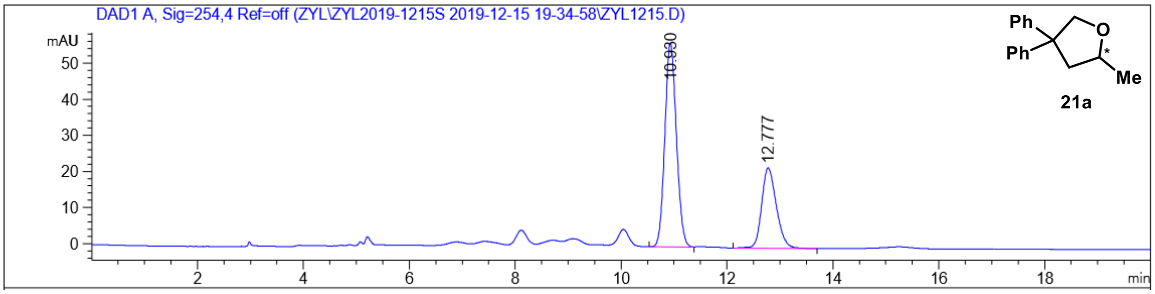

.

Signal 1: DAD1 A, Sig=254,4 Ref=off

Peak #	RetTime [min]	Type	Width [min]	Area [mAU*s]	Height [mAU]	Area %
1	10.930	BB	0.2343	854.58081	56.55159	66.6114
2	12.777	BB	0.2946	428.35492	22.24724	33.3886
Totals :				1282.93573	78.79883	

6.5 General Procedure for Catalytic Enantioselective Hydroamination

Catalyst **M** and catalyst **N** were generated *in situ.*

In an argon filled glovebox, to a 4 mL vial with a magnetic stir bar were added the (***R***)-**P2** or (***R***)-**P1** (0.005mmol, 5 mol%), silver trifluoromethanesulfonate (0.012 mmol), $ClCH_2CH_2Cl$ (200 μL). The mixture was stirred at room temperature (23 °C) for 1 h. And monophosphine ligand **L4** (0.002mmol, 5 mol%) dissolved in $ClCH_2CH_2Cl$ (100 μL) was added into the above mixture. The resulting solution was stirred for 5 min and *N*-((1-allylcyclohexyl)methyl)-4-methylbenzenesulfonamide **2** (31 mg, 0.1 mmol, 1.0 equiv) was added. The mixture was stirred at 50 °C for 48 h. The reaction mixture was concentrated under reduced pressure and the crude product was purified by silica gel column chromatography using hexane/ ethyl acetate (20/1) to provide the product **2a**.

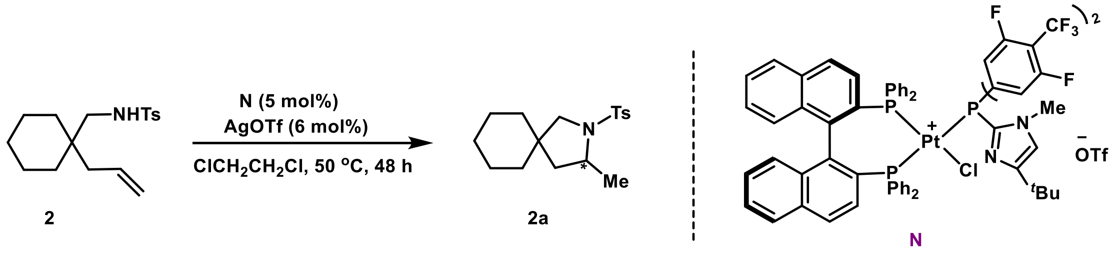

The desired product **2a** (15% yield, 58% ee) was obtained. HPLC (OJ-H, 0.46*25 cm, 5μm, hexane / ethanol = 90/10, flow 1.0 mL/min, detection at 254 nm)retention time = 6.390 min (major) and 7.789 min (minor)

<Chromatogram>

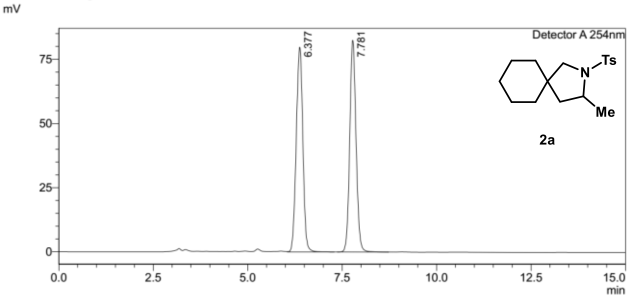

<Peak Table>

Detector A 254nm

Peak#	Ret. Time	Area	Height	Conc.	Unit	Mark	Name
1	6.377	970636	79803	50.224		S	
2	7.781	961978	82513	49.776			
Total		1932614	162315				

<Chromatogram>

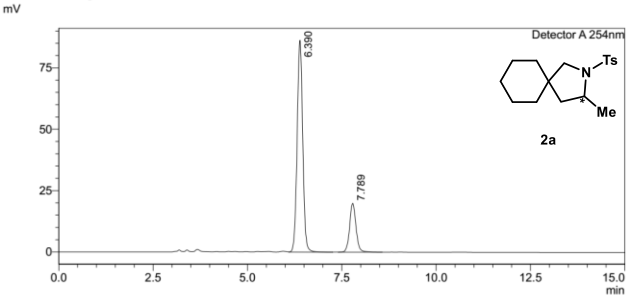

<Peak Table>

Detector A 254nm

Peak#	Ret. Time	Area	Height	Conc.	Unit	Mark	Name
1	6.390	885557	86312	79.105		S	
2	7.789	233919	19856	20.895			
Total		1119476	106168				

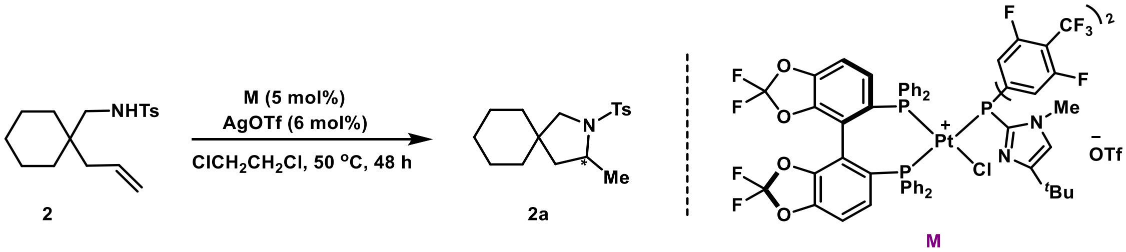

The desired product **2a** (18% yield, 65% ee) was obtained. HPLC (OJ-H, 0.46*25 cm, 5μm, hexane / ethanol = 90/10, flow 1.0 mL/min, detection at 254 nm) retention time = 6.375 min (major) and 7.787 min (minor)

<Chromatogram>

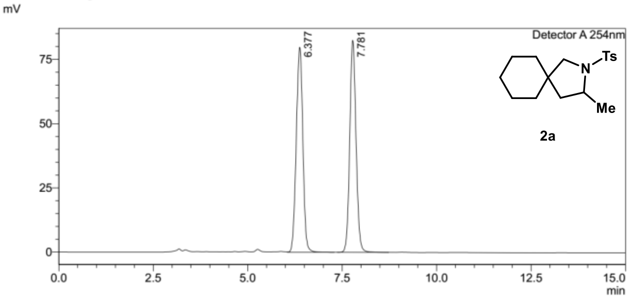

<Peak Table>

Detector A 254nm

Peak#	Ret. Time	Area	Height	Conc.	Unit	Mark	Name
1	6.377	970636	79803	50.224		S	
2	7.781	961978	82513	49.776			
Total		1932614	162315				

<Chromatogram>

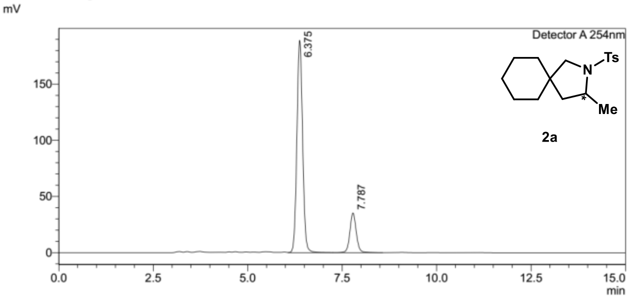

<Peak Table>

Detector A 254nm

Peak#	Ret. Time	Area	Height	Conc.	Unit	Mark	Name
1	6.375	1989577	189133	82.700			
2	7.787	416191	35288	17.300		V	
Total		2405768	224421				

7. NMR Monitoring of Hydroalkoxylation Reaction

7.1 NMR Monitoring of Hydroalkoxylation of Norbornene and 4-Trifluromethylphenol Catalyzed by Catalyst D and Different Amounts of AgOTf

7.1.1 Hydroalkoxylation of Norbornene and 4-Trifluromethylphenol Catalyzed by Complex D and 1 equiv. of AgOTf

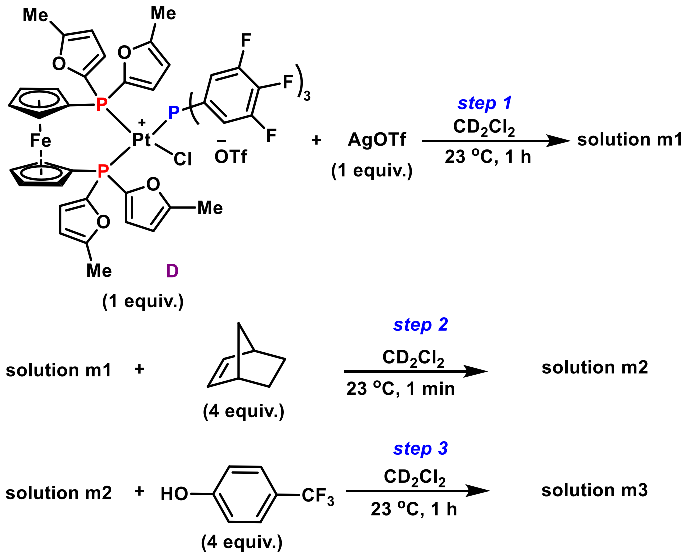

Procedure

Step 1: In an argon filled glovebox, to a 4 mL vial with a magnetic stir bar were added **D** (28 mg, 0.02 mmol), AgOTf (5 mg, 0.02 mmol) and CD_2Cl_2 (500 μL). The mixture was stirred at room temperature (23 °C) for 1 h to give solution m1. Solution m1 was recorded by ^{1}H NMR, ^{19}F NMR and ^{31}P NMR respectively. ^{31}P NMR chemical shifts are referenced to an external PPh_3 standard in CD_2Cl_2 sealed in a capillary tube. Analysis of the ^{31}P NMR showed that four new peaks at δ -33.5 ppm, -22.5 ppm, 12.5 ppm and 15.7 ppm were formed (Supplementary Figure 4b). Analysis of the ^{19}F NMR showed new two peaks at -131.6 (d, J = 21.3 Hz) and -154.8 (t, J = 20.8 Hz) ppm were formed (Supplementary Figure 5b).

Step 2: Norbornene (7.5 mg, 0.08 mmol) dissolved in CD_2Cl_2 (100 μL) was added into tube containing solution m1. The mixture was stirred for 30 min and the resulting-solution m2 was monitored by NMR techniques.

Step 3: 4-Trifluromethylphenol (13 mg, 0.08 mmol) dissolved in CD_2Cl_2 (100 μL) was added into tube containing solution m2. The mixture was stirred for 1 h and the resulting-solution was monitored by NMR techniques.

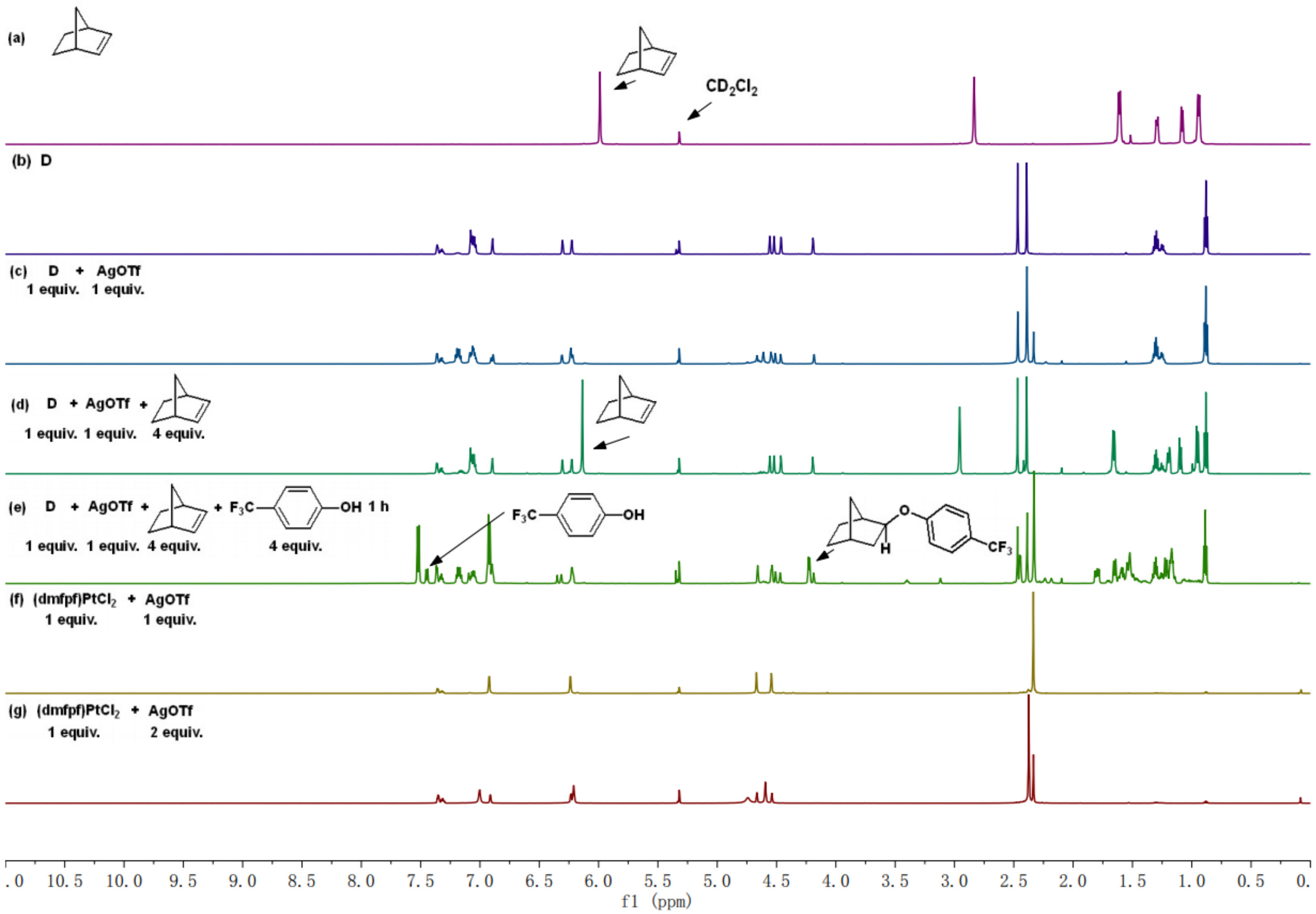

Supplementary Figure 3. ^{1}H NMR (600 MHz, CD_2Cl_2) Monitoring of Hydroalkoxylation of Norbornene and 4-Trifluromethylphenol Catalyzed by Catalyst **D** and 1 equiv. of AgOTf

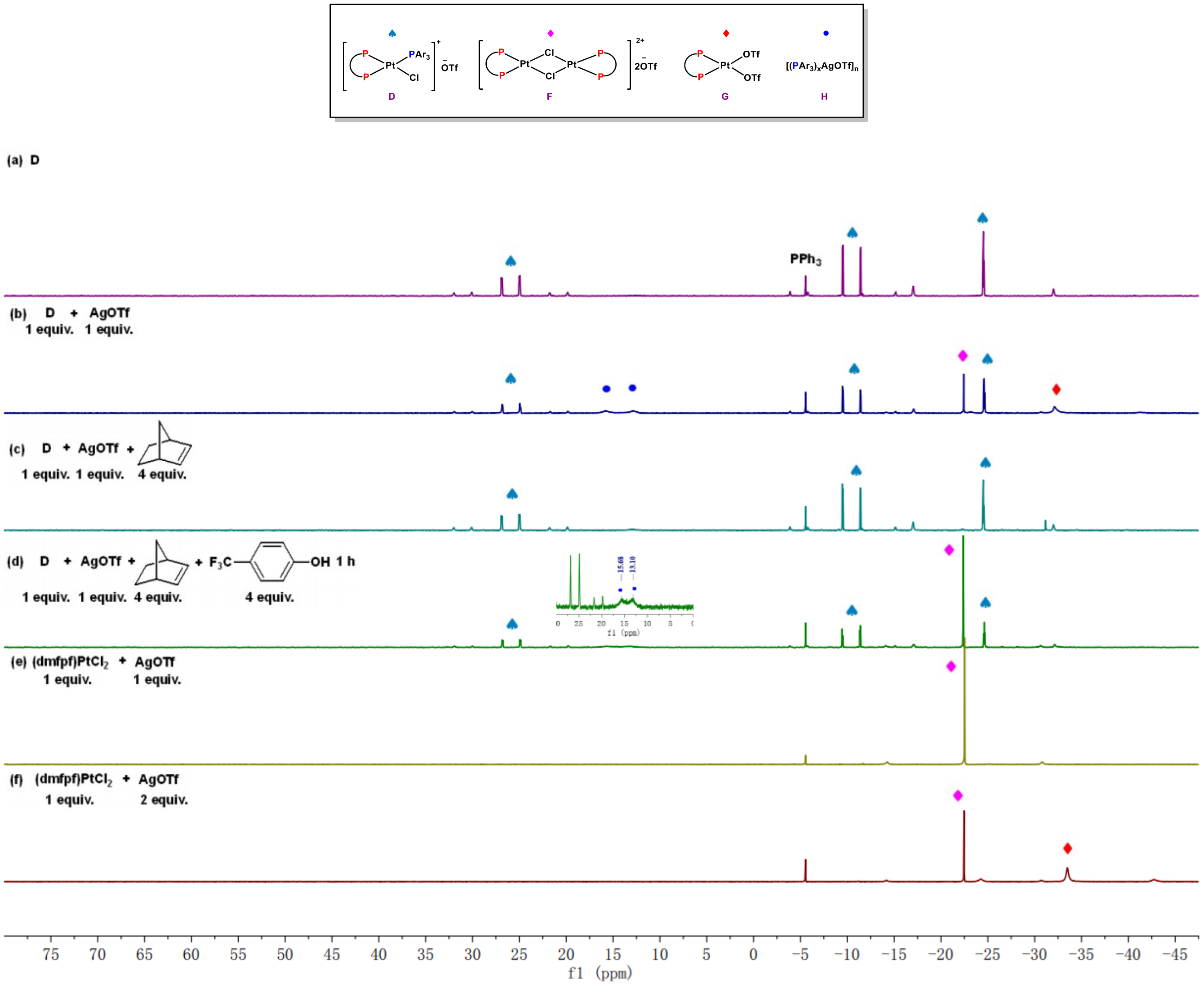

Supplementary Figure 4. ^{31}P NMR (243 MHz, CD_2Cl_2) Monitoring of Hydroalkoxylation of Norbornene and 4-Trifluromethylphenol Catalyzed by Catalyst **D** and 1 equiv. of AgOTf

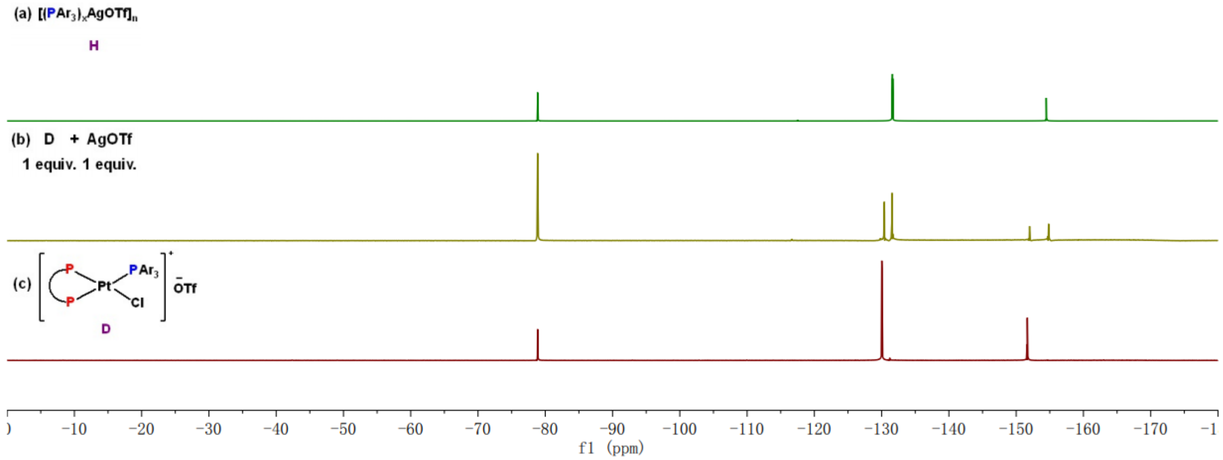

Supplementary Figure 5. ^{19}F NMR (565 MHz, CD_2Cl_2) Monitoring of Hydroalkoxylation of Norbornene and 4-Trifluromethylphenol Catalyzed by Catalyst **D** and 1 equiv. of AgOTf

7.1.2 Hydroalkoxylation of Norbornene and 4-Trifluromethylphenol Catalyzed by Complex D and 2 equiv. of AgOTf

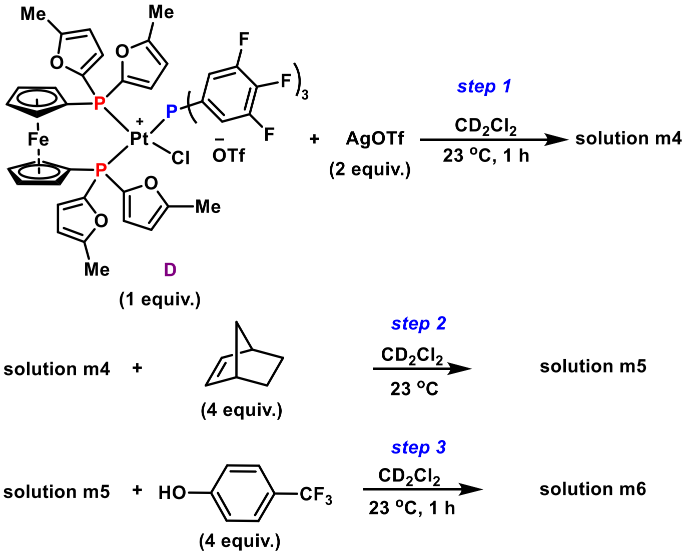

Procedure

Step 1: In an argon filled glovebox, to a 4 mL vial with a magnetic stir bar were added **D** (28 mg, 0.02 mmol), AgOTf (10mg, 0.04 mmol) and CD_2Cl_2 (500 μL). The mixture was stirred at room temperature (23 °C) for 1 h. The resulted solution was recorded by ^{1}H NMR and ^{31}P NMR respectively. ^{31}P NMR chemical shifts are referenced to an external PPh_3 standard in CD_2Cl_2 sealed in a capillary tube. Analysis of the ^{31}P NMR showed that three new peaks at δ -33.5 ppm, -22.5 ppm, 14.4 ppm were formed (Supplementary Figure 7b).

Step 2: Norbornene (7.5 mg, 0.08 mmol) dissolved in CD_2Cl_2 (100 μL) was added into tube containing solution m4. The mixture was stirred for 30 min and the resulting-solution m5 was monitored by NMR techniques. Analysis of the ^{31}P NMR showed that the new three groups of peaks [δ 33.6 (dd, $^2J_{P\text{-}P}$ = 410, 22 Hz, $^1J_{Pt\text{-}P}$ = 3075), -12.6 (dd, $^2J_{P\text{-}P}$ = 411, 23 Hz, $^1J_{Pt\text{-}P}$ = 3463), -17.2 (t, $^2J_{P\text{-}P}$ = 22 Hz, $^1J_{Pt\text{-}P}$ = 1873)] were formed (Supplementary Figure 8c).

Step 3: 4-Trifluromethylphenol (13 mg, 0.08 mmol) dissolved in CD_2Cl_2 (100 μL) was added into tube containing solution m5. The mixture was stirred for 1 h and the resulting-solution was monitored by NMR techniques.

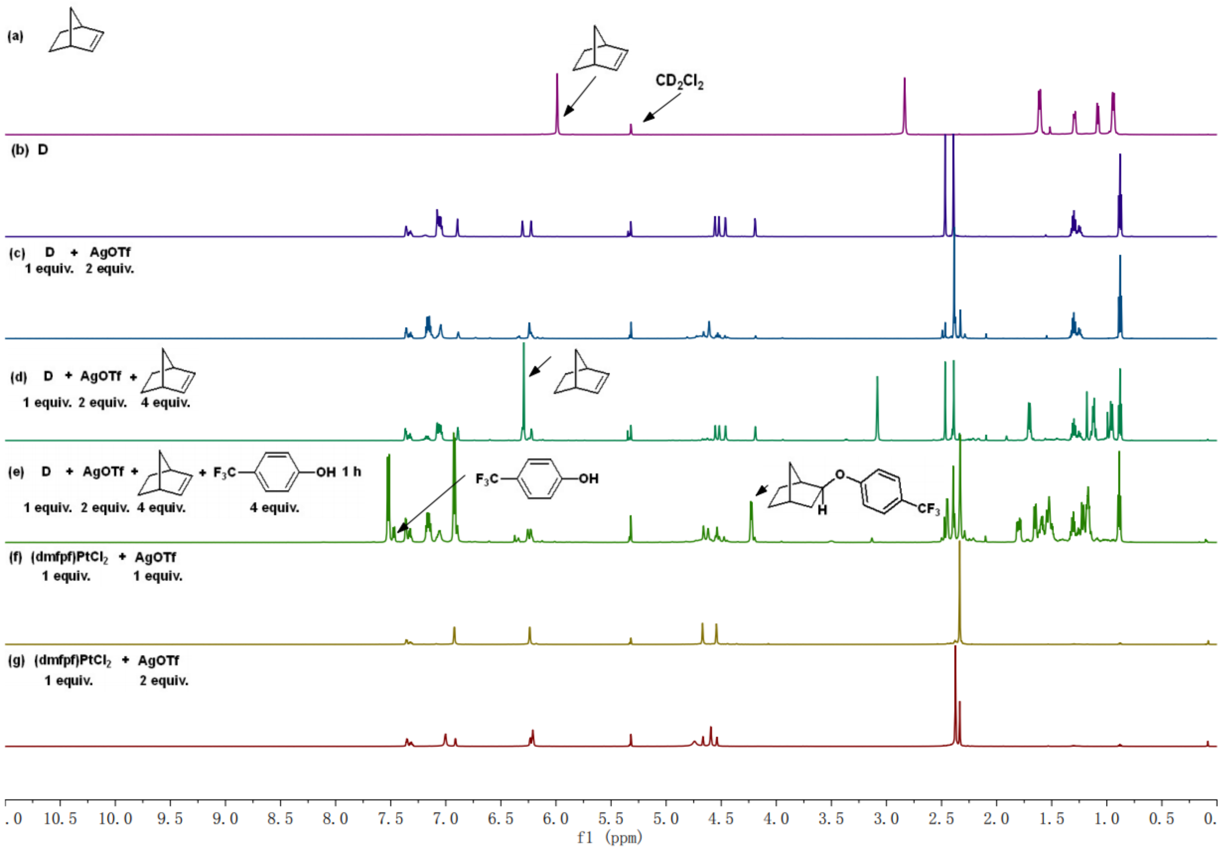

Supplementary Figure 6. ^{1}H NMR (600 MHz, CD_2Cl_2) Monitoring of Hydroalkoxylation of Norbornene and 4-Trifluromethylphenol Catalyzed by Catalyst **D** and 2 equiv. of AgOTf

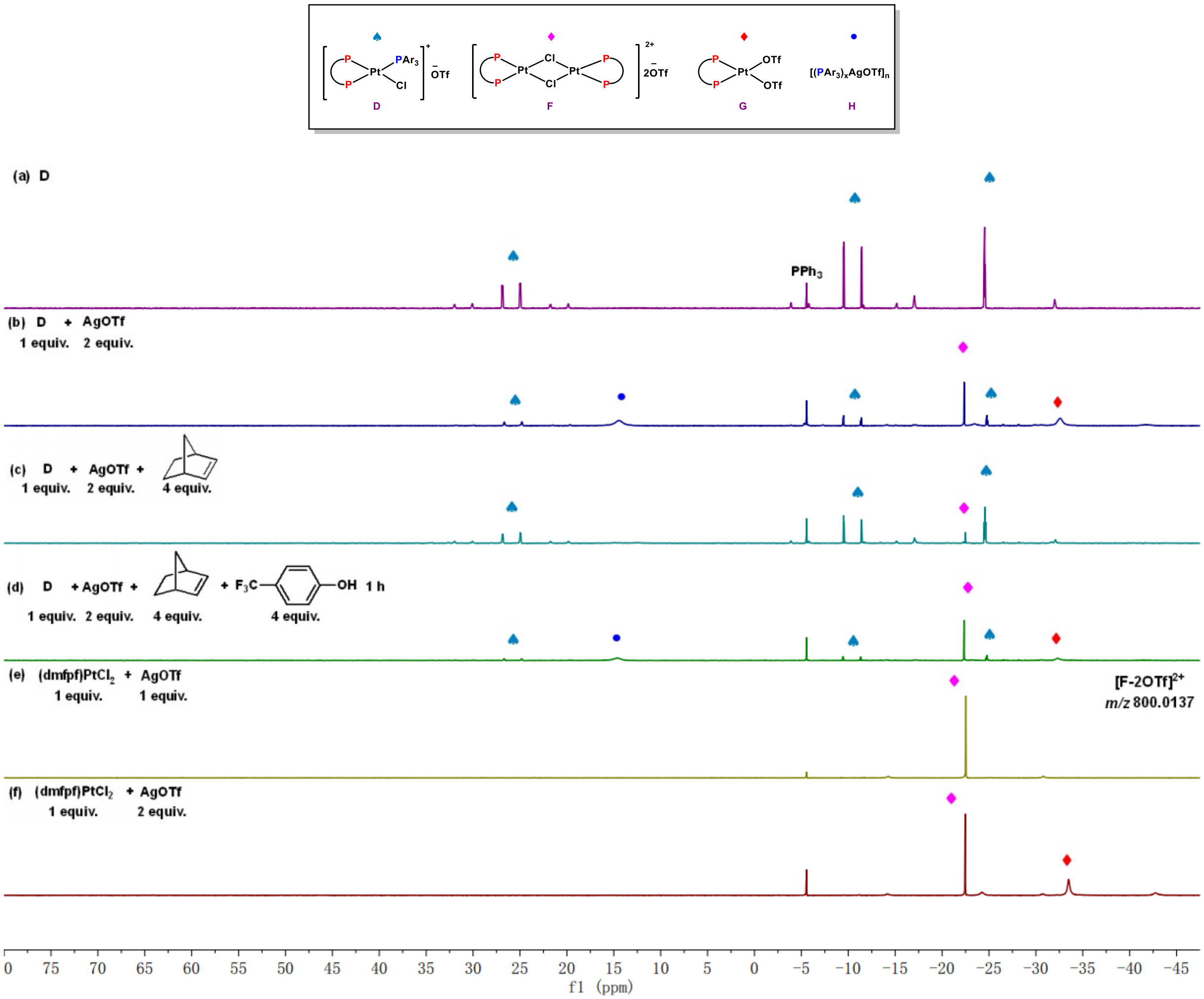

Supplementary Figure 7. ^{31}P NMR (243 MHz, CD_2Cl_2) Monitoring of Hydroalkoxylation of Norbornene and 4-Trifluromethylphenol Catalyzed by Catalyst **D** and 2 equiv. of AgOTf

As shown below, the three new groups of peaks [δ 33.6 (dd, $^{2}J_{P\text{-}P}$ = 410, 22 Hz, $^{1}J_{Pt\text{-}P}$ = 3075), -12.6 (dd, $^{2}J_{P\text{-}P}$ = 411, 23 Hz, $^{1}J_{Pt\text{-}P}$ = 3463), -17.2 (t, $^{2}J_{P\text{-}P}$ = 22 Hz, $^{1}J_{Pt\text{-}P}$ = 1873)] were formed when adding norbornene to a mixture of complex **D** and AgOTf (Supplementary Figure 8e). To the filtrate of a mixture of complex **D** and 2 equiv. of AgOTf was added norbornene, the concentration of intermediate **J** increased (Supplementary Figure 8c).

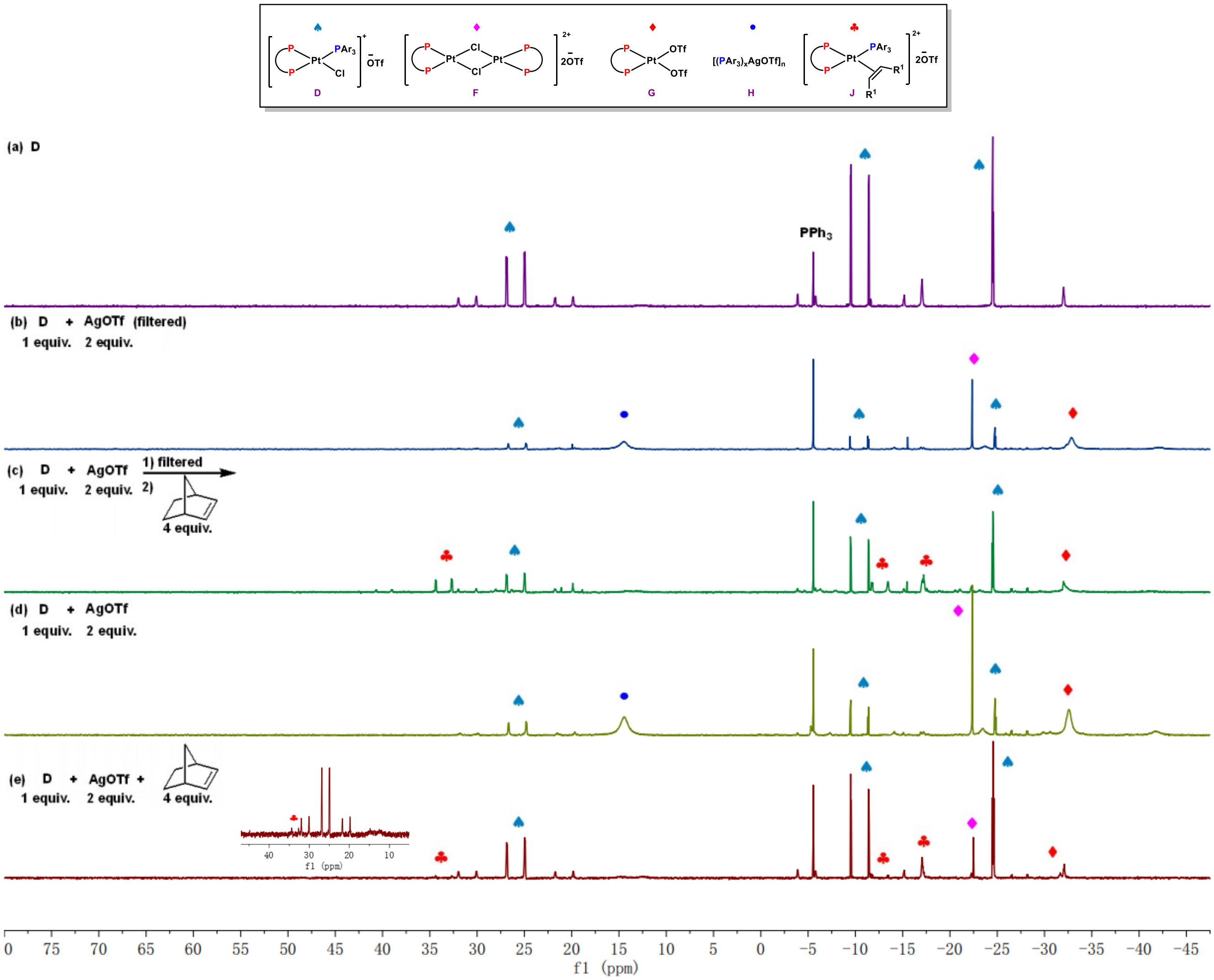

Supplementary Figure 8. Comparison of ^{31}P NMR (243 MHz, CD_2Cl_2) of the Reaction of D + AgOTf + Norbornene and Reaction of the Filtrate of D and AgOTf + Norbornene (^{31}P NMR Chemical Shifts Are Referenced to An External PPh_3 Standard in CD_2Cl_2 Sealed in A Capillary Tube.)

7.1.3 Hydroalkoxylation of Norbornene and 4-Trifluromethylphenol Catalyzed by Complex D and 5 equiv. of AgOTf

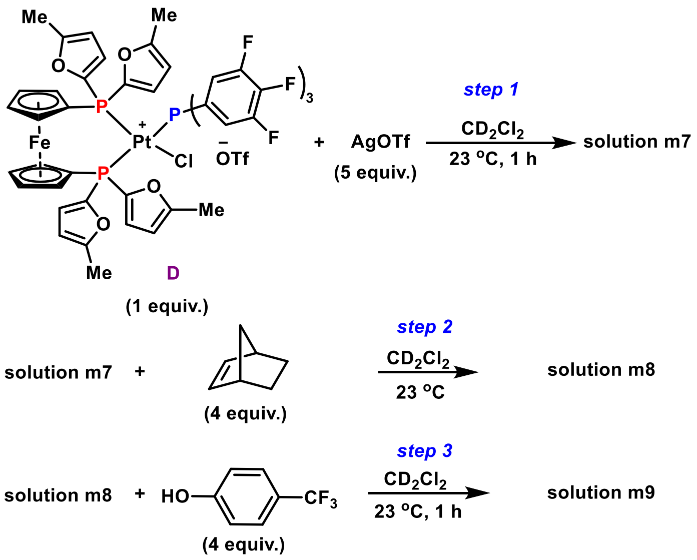

Procedure

Step 1: In an argon filled glovebox, to a 4 mL vial with a magnetic stir bar were added **D** (28 mg, 0.02 mmol), AgOTf (26 mg, 0.1 mmol) and CD_2Cl_2 (500 μL). The mixture was stirred at room temperature (23 ^{o}C) for 1 h. The resulted solution was recorded by ^{1}H NMR and ^{31}P NMR respectively. ^{31}P NMR chemical shifts are referenced to an external PPh_3 standard in CD_2Cl_2 sealed in a capillary tube. Analysis of the ^{31}P NMR showed that three new peaks at δ -33.5 ppm, -22.4 ppm, 14.6 ppm were formed (Supplementary Figure10b).

Step 2: Norbornene (7.5 mg, 0.08 mmol) dissolved in CD_2Cl_2 (100 μL) was added into tube containing solution m7. The tube was stirred for 30 min and the resulting-solution was monitored by NMR techniques.

Step 3: 4-Trifluromethylphenol (13 mg, 0.08 mmol) dissolved in CD_2Cl_2 (100 μL) was added into tube containing solution m8. The mixture was stirred for 1 h and the resulting-solution was monitored by NMR techniques.

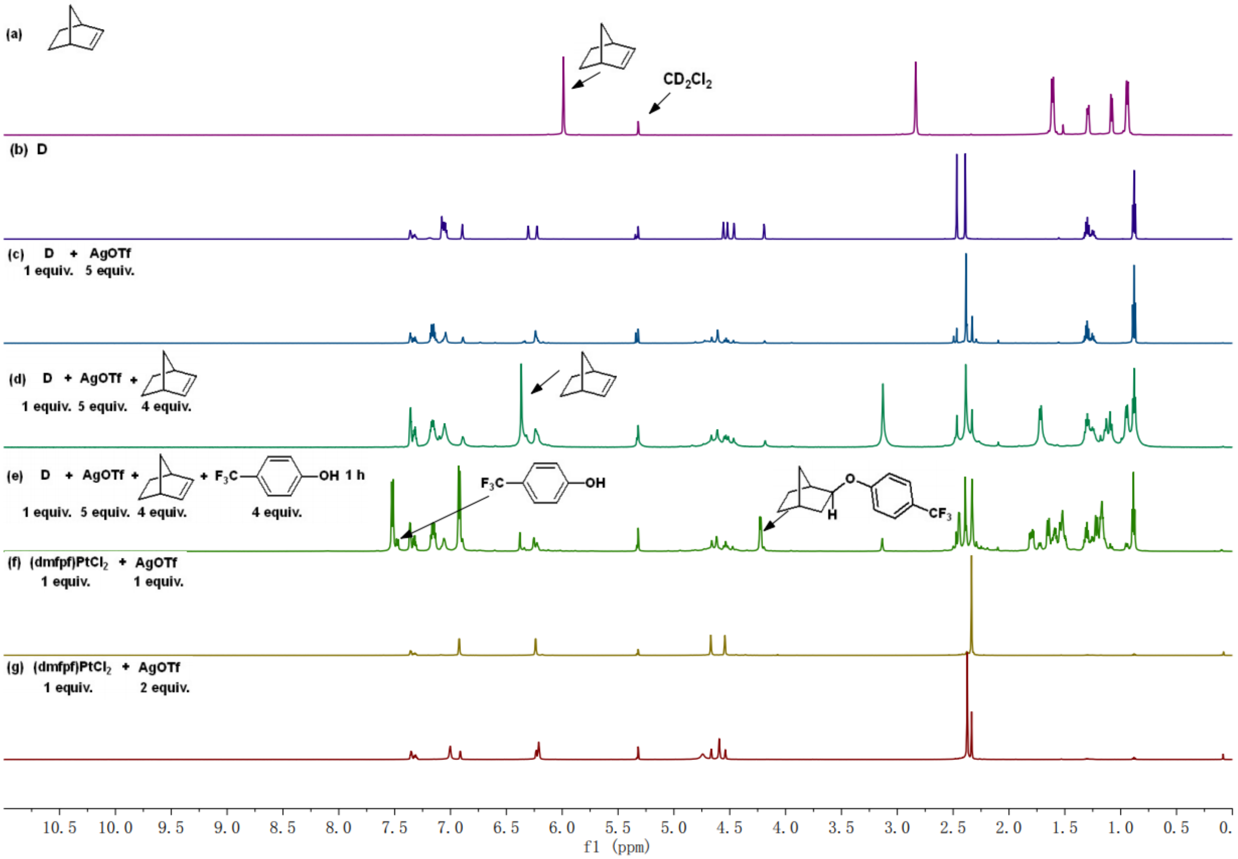

Supplementary Figure 9. ^{1}H NMR (600 MHz, CD_2Cl_2) Monitoring of Hydroalkoxylation of Norbornene and 4-Trifluromethylphenol Catalyzed by Catalyst **D** and 5 equiv. of AgOTf

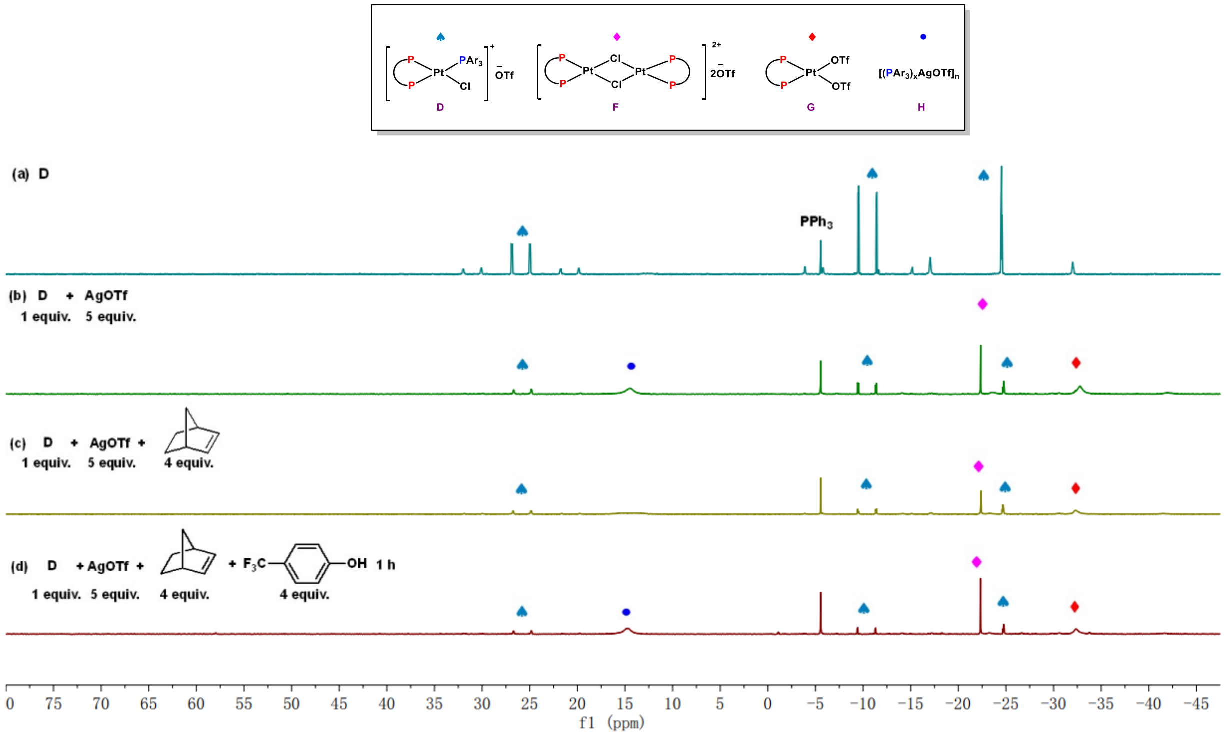

Supplementary Figure 10. ^{31}P NMR (243 MHz, CD_2Cl_2) Monitoring of Hydroalkoxylation of Norbornene and 4-Trifluromethylphenol Catalyzed by Catalyst **D** and 5 equiv. of AgOTf

7.1.4 Hydroalkoxylation of Norbornene and 4-Trifluromethylphenol Catalyzed by Complex D and 50 equiv. of AgOTf

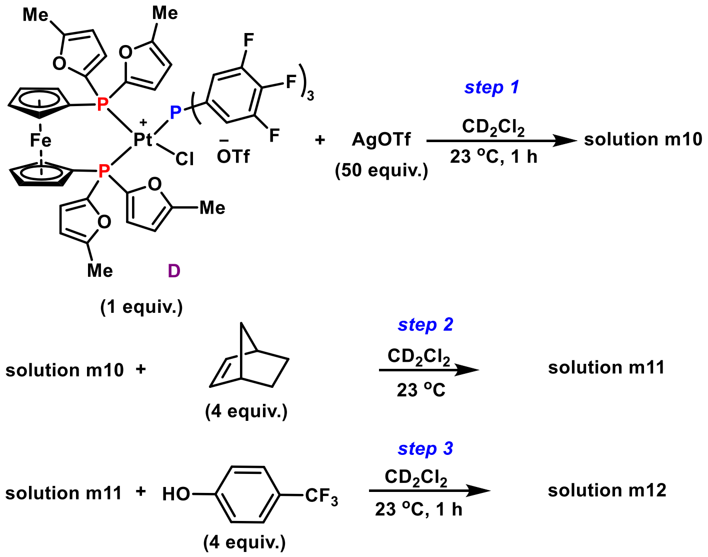

Procedure

Step 1: In an argon filled glovebox, to a 4 mL vial with a magnetic stir bar were added **D** (28 mg, 0.02 mmol), AgOTf (257mg, 1.0 mmol) and CD_2Cl_2 (500 μL). The mixture was stirred at room temperature (23 ^{o}C) for 1 h. The resulted solution was recorded by ^{1}H NMR and ^{31}P NMR respectively. ^{31}P NMR chemical shifts are referenced to an external PPh_3 standard in CD_2Cl_2 sealed in a capillary tube. Analysis

of the ^{31}P NMR showed that three new peaks at δ -33.5 ppm, -22.4 ppm, 14.6 ppm were formed (Supplementary Figure 12b).

Step 2: Norbornene (7.5 mg, 0.08 mmol) dissolved in CD_2Cl_2 (100 μL) was added into tube containing solution m10. The resulting mixture was stirred for 30 min and the resulting-solution was monitored by NMR techniques.

Step 3: 4-Trifluromethylphenol (13 mg, 0.08 mmol) dissolved in CD_2Cl_2 (100 μL) was added into tube containing solution m11. The resulting mixture was stirred for 1 h and the resulting-solution was monitored by NMR techniques.

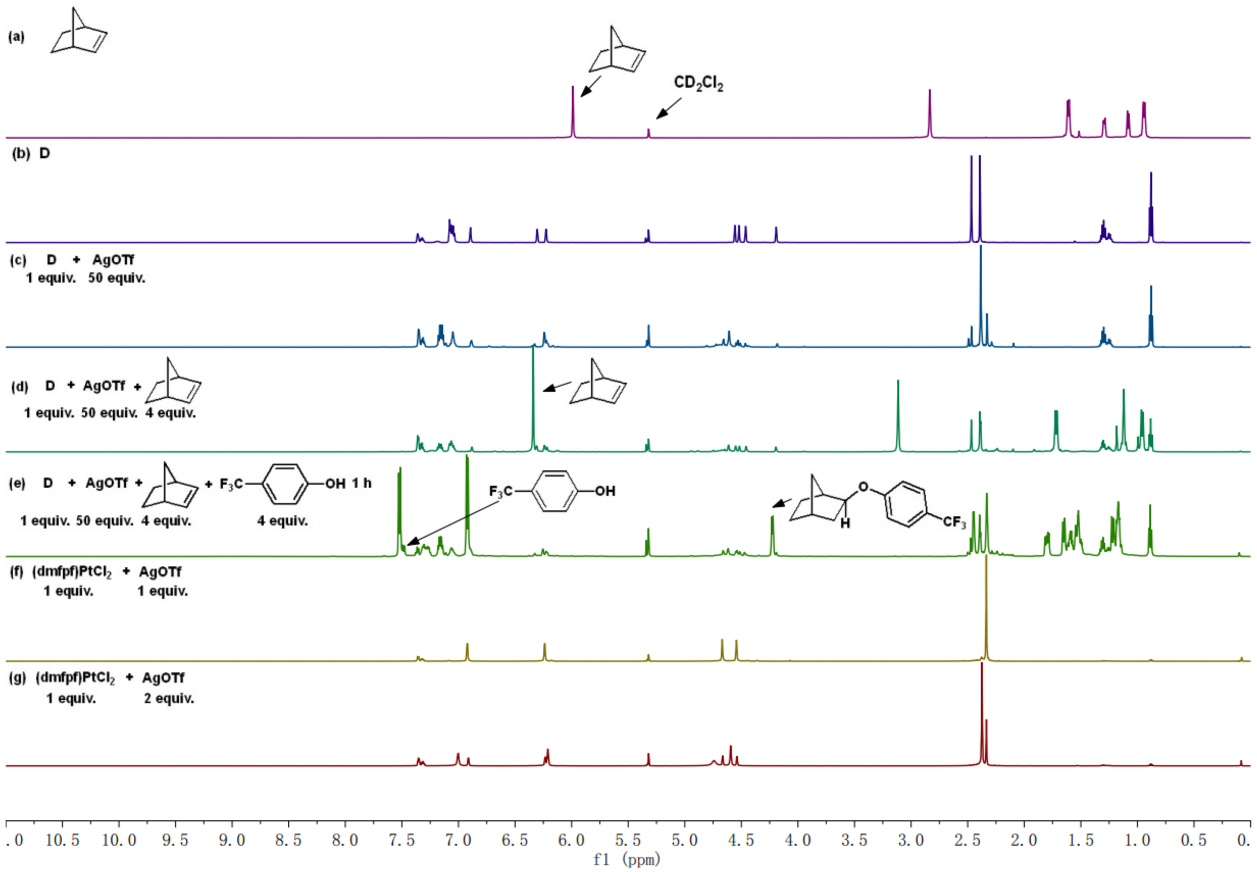

Supplementary Figure 11. ^{1}H NMR (600 MHz, CD_2Cl_2) Monitoring of Hydroalkoxylation of Norbornene and 4-Trifluromethylphenol Catalyzed by Catalyst D and 50 equiv. of AgOTf

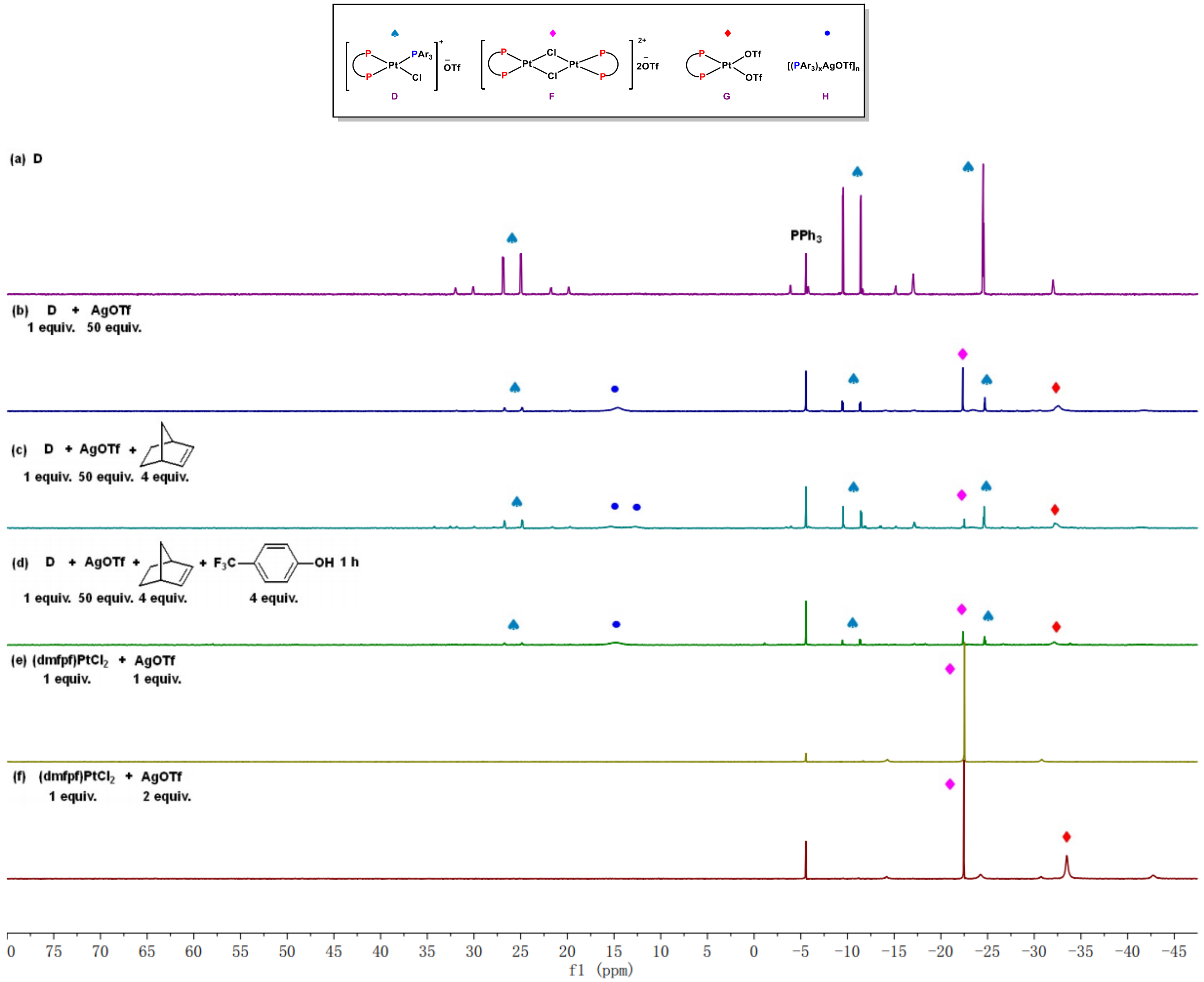

Supplementary Figure 12. ^{31}P NMR (243 MHz, CD_2Cl_2) Monitoring of Hydroalkoxylation of Norbornene and 4-Trifluromethylphenol Catalyzed by Catalyst **D** and 50 equiv. of AgOTf

7.2 Synthesis of Complexes F, G, and H

7.2.1 Synthesis and Characterization of Complex F

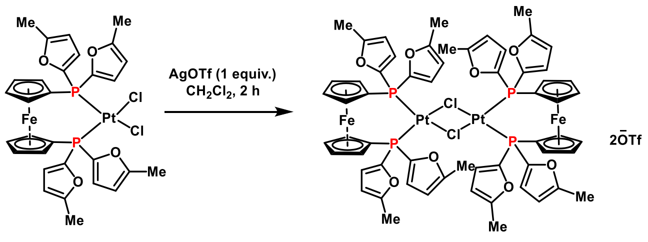

F

Procedure: In an argon filled glovebox, to a 4 mL vial with a magnetic stir bar were added (dmfpf)$PtCl_2$ (100 mg, 0.12 mmol), silver trifluoromethanesulfonate (31 mg, 0.12 mmol, 1.0 equiv.) and 2 mL CH_2Cl_2. The mixture was stirred at room temperature (23 ^oC) for 2 h. The purple solution was filtered and CH_2Cl_2 was evaporated to provide purple solid, which was recrystallized through CH_2Cl_2 and hexane to give purple precipitate.

^{1}H NMR (600 MHz, CD_2Cl_2): δ 6.91 (d, J = 3.3 Hz, 8H), 6.23(d, J = 3.3 Hz, 8H), 4.66 (s, 8H), 4.53 (s, 8H), 2.32 (s, 24H) ppm

^{13}C NMR (151 MHz, CD_2Cl_2): δ 161.9, 136.7 (d, J = 111 Hz), 127.8 – 127.6 (m), 109.2 (d, J = 3.7 Hz), 76.8 – 76.6 (m), 76.3 – 76.1 (m), 66.8 (d, J = 91.8 Hz), 14.3 ppm

^{31}P NMR (243 MHz, CD_2Cl_2): δ -22.5 (d, $^1J_{Pt\text{-}P}$ = 4018 Hz) ppm

^{19}F NMR (376 MHz, CD_2Cl_2): δ -78.5 (s) ppm

HRMS (ESI-): calculated for $C_{60}H_{56}Cl_2Fe_2O_8P_4Pt_2$ $[M\text{-}2OTf]^{2+}$: m/z = 800.0149, found 800.0137.

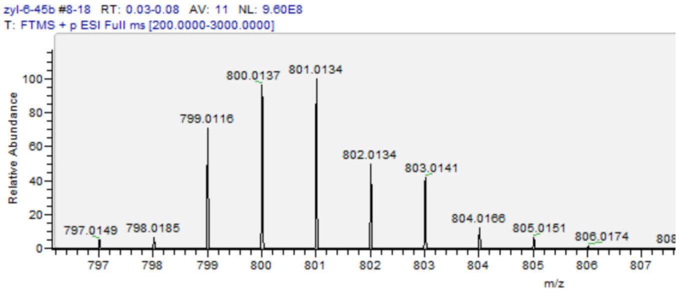

Supplementary Figure 13. HRMS of Complex **F**

Attempts to form X-ray quality crystals of complex **F** failed. To check if complex **F** is a dinuclear species with a bridging Cl ligand, a test experiment used (dppf)$PtCl_2$ with 1 equiv. of AgOTf to generated a new complex **Q**. Complex **Q** was identified by X-ray crystallography as a dinuclear species with a bridging Cl ligand. So it assumed that complex **F** was like a dinuclear species with a bridging Cl ligand. Therefor the peak at δ -22.5 ppm was proposed to be the dinuclear complex **F**.

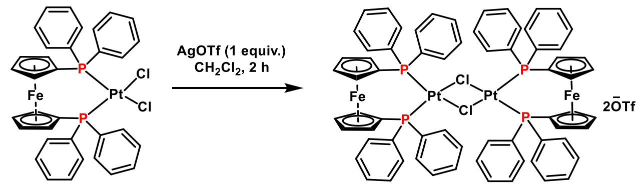

Q

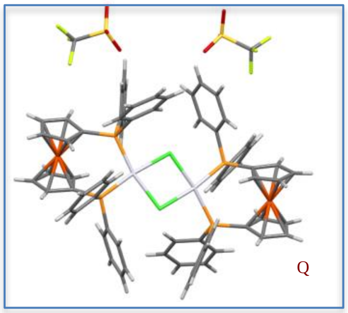

^{1}H NMR (400 MHz, CD_2Cl_2): δ 7.58 – 7.50 (m, 8H), 7.41 – 7.37(d, J = 3.3 Hz, 8H), 4.65 (s, 8H), 4.41 (s, 8H) ppm

^{13}C NMR (101 MHz, CD_2Cl_2): δ 134.4 – 134.3 (m), 133.7, 129.6 – 129.4 (m), 127.2 (d, J = 70.1 Hz), 77.7 – 77.5 (m), 76.4 – 76.3 (m), 68.11 (d, J = 79.7 Hz) ppm

^{31}P NMR (162 MHz, CD_2Cl_2): δ 18.3 (d, $^1J_{Pt\text{-}P}$ = 3983 Hz) ppm

^{19}F NMR (376 MHz, CD_2Cl_2): δ -78.5 (s) ppm

7.2.2 Synthesis and Characterization of Complex G

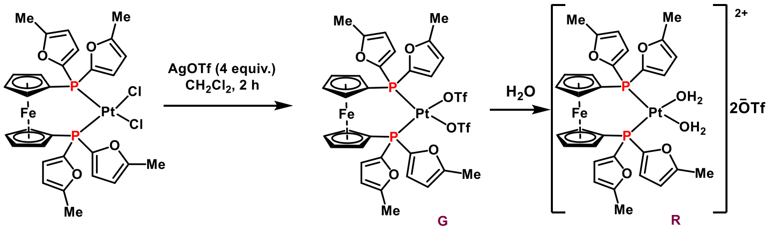

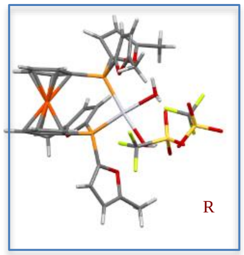

Procedure: In an argon filled glovebox, to a 4 mL vial with a magnetic stir bar were added (dmfpf)$PtCl_2$ (17 mg, 0.02 mmol), silver trifluoromethanesulfonate (20 mg, 0.08mmol, 4.0 equiv.) and 2 mL CH_2Cl_2. The mixture was stirred at room temperature (23 ^{o}C) for 2 h. We obtained a mixture of complex **F** and complex **G**. The major product was complex **G**. Complexes **G** was moisture-sensitive and readily formed complex **R**. Complex **G** was confirmed by HRMS at $C_{31}H_{28}F_3FeO_7P_2PtS$ $[M\text{-}OTf]^+$: 913.9975, found 913.9969. Therefor the peak at δ -33.5 ppm belonged to the complex **G**

^{31}P NMR (162 MHz, CD_2Cl_2): δ -33.5 (d, $^1J_{Pt\text{-}P}$ = 4511 Hz) ppm.

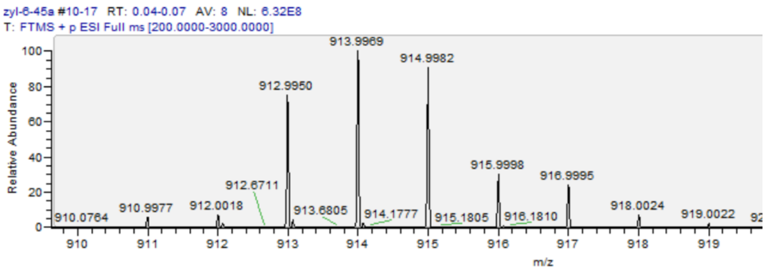

Supplementary Figure 14. HRMS of Complex **G**

7.2.3 Synthesis of Complex H

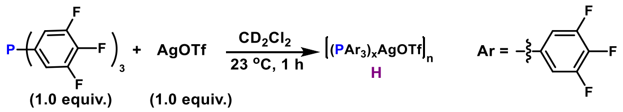

In an argon filled glovebox, to a 4 mL vial with a magnetic stir bar were added tris(3,4,5-trifluorophenyl)phosphine (21 mg, 0.05 mmol), AgOTf (13 mg, 0.05 mmol) and CD_2Cl_2 (500 μL). The mixture was stirred at room temperature (23 °C) for 1 h. The resulting solution was recorded by ^{1}H NMR, ^{19}F NMR and ^{31}P NMR. Analysis of ^{31}P NMR showed two peaks at δ 15.7 ppm and 12.5 ppm were formed (Supplementary Figure 15 to 17)[19-20].

^{1}H NMR (600 MHz, CD_2Cl_2): δ 7.15 – 7.11(m, 6H) ppm

^{31}P NMR (243 MHz, CD_2Cl_2): δ 15.7 (s), 12.5 (s) ppm

^{19}F NMR (565 MHz, CD_2Cl_2): δ -78.9 (s), -131.6 (d, J = 21.3 Hz), -154.5 (t, J = 20.8 Hz) ppm

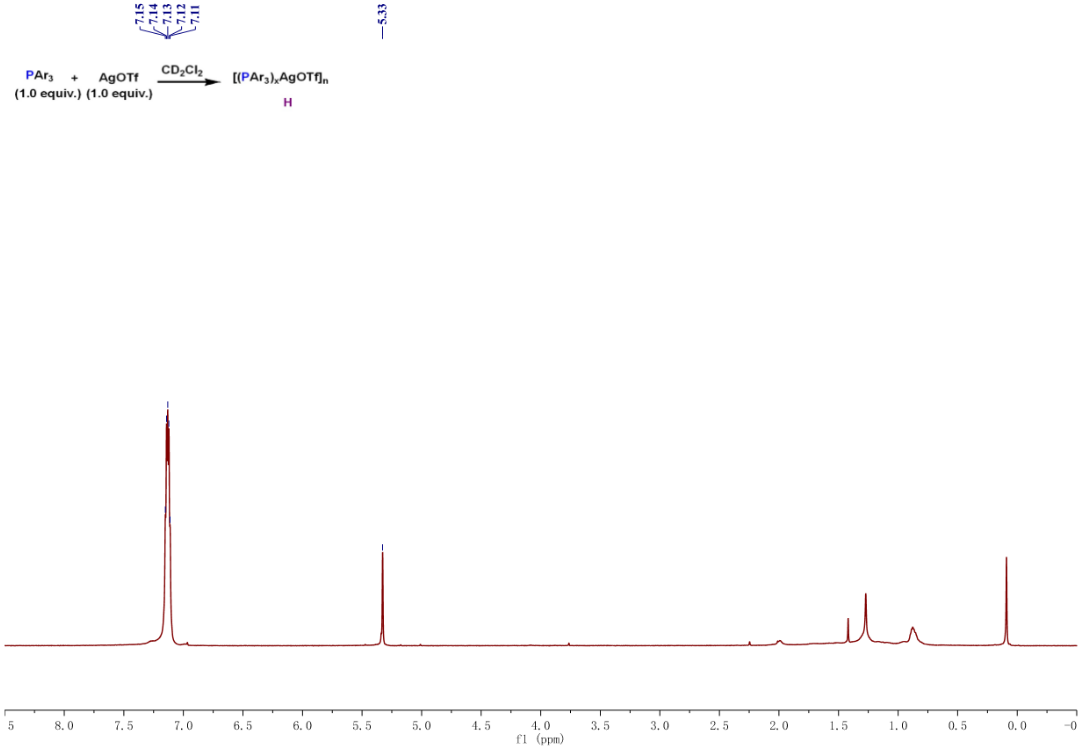

Supplementary Figure 15. ^{1}H NMR (600 MHz, CD_2Cl_2) of Complex **H**

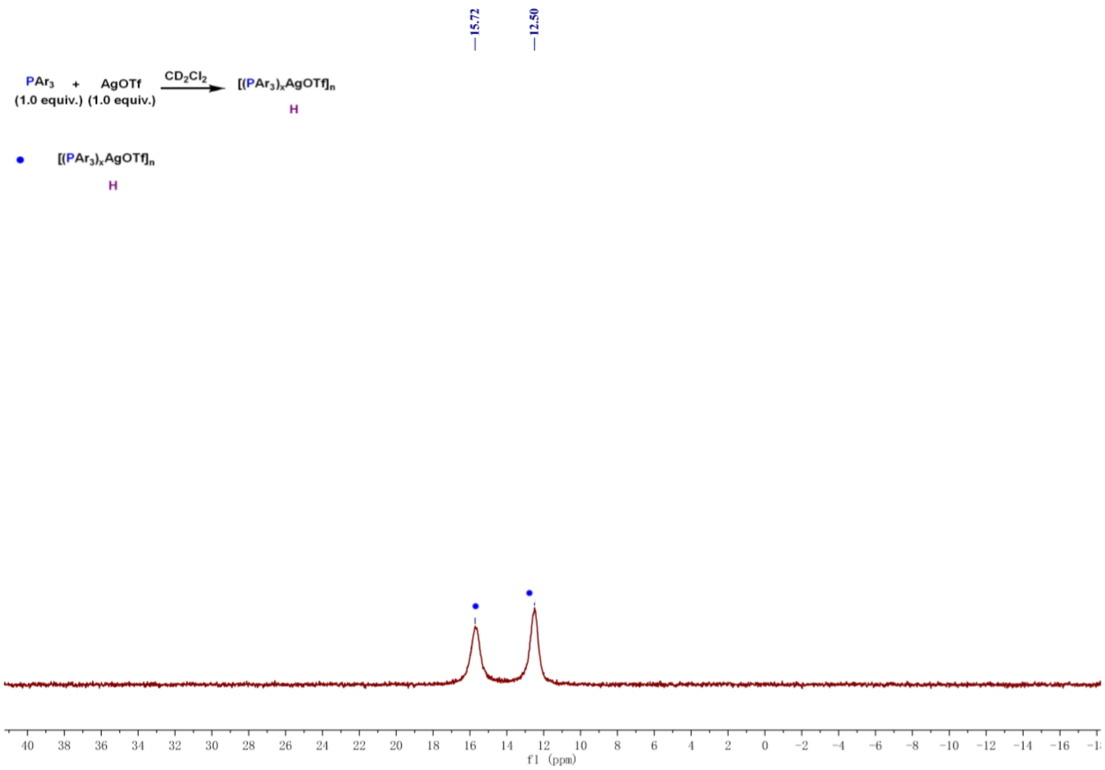

Supplementary Figure 16. ^{31}P NMR (243 MHz, CD_2Cl_2) of Complex **H**

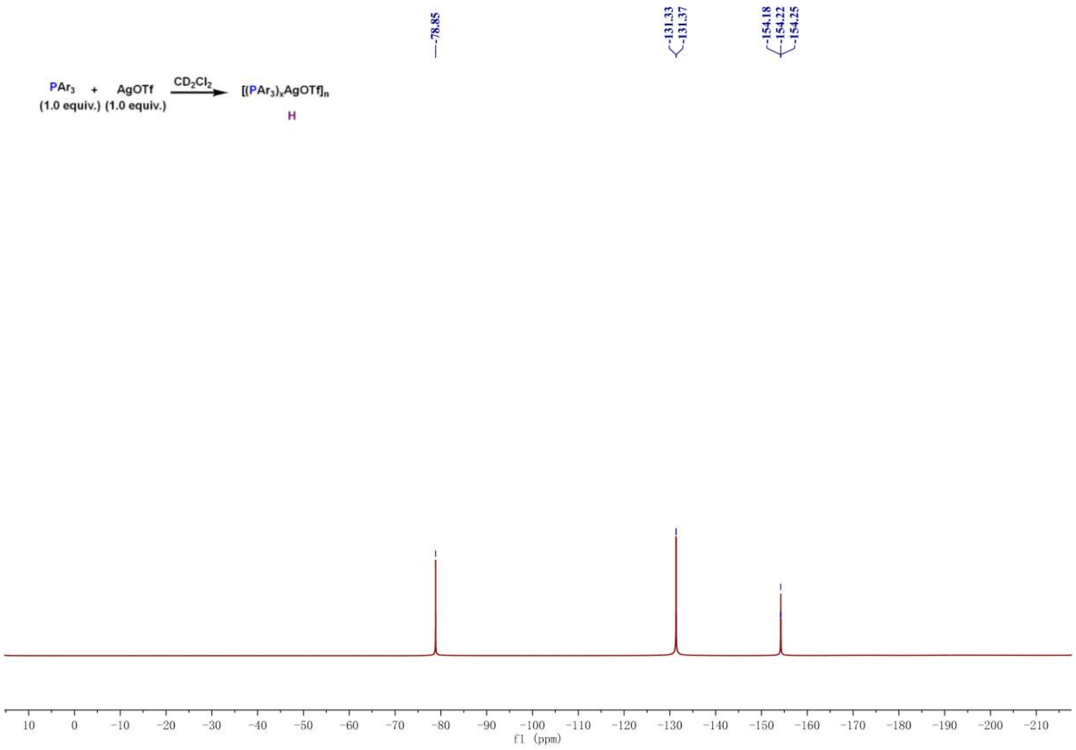

Supplementary Figure 17. ^{19}F NMR (565 MHz, CD_2Cl_2) of Complex **H**

7.3 Investigation of the Catalytic Activity of Complex F, G, and H

7.3.1 Comparison of the Catalytic Activity of Complex F, G and H

Complex **F** was generated from dmfpf$PtCl_2$ (1 mol%) and AgOTf (1 mol%) in $ClCH_2CH_2Cl$ (Supplementary Table 7, entry 1).

Complex **G** was generated from dmfpf$PtCl_2$ (1 mol%) and AgOTf (4 mol%) in $ClCH_2CH_2Cl$ (Supplementary Table 7, entry 2).

Complex **H** was generated from tris(3,4,5-trifluorophenyl)phosphine (1 mol%) and AgOTf (1 mol%) in $ClCH_2CH_2Cl$(Supplementary Table 7, entry 3).

Complex **P** was generated from dmfpf$PtCl_2$ (1 mol%), AgOTf (4 mol%) and tris(3,4,5-trifluorophenyl)phosphine (1 mol%) in $ClCH_2CH_2Cl$ (Supplementary Table 7, entry 4).

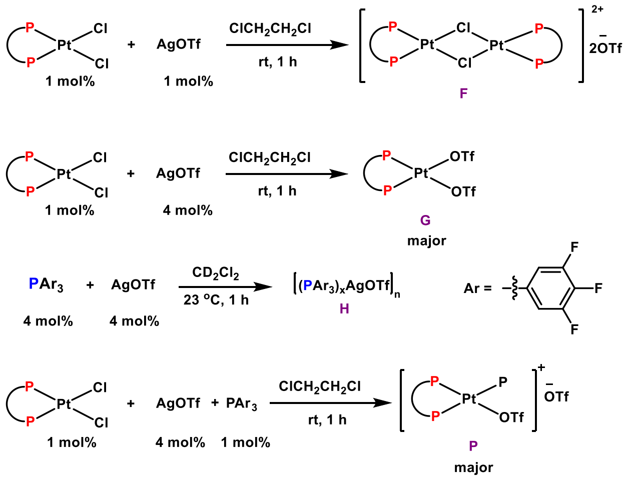

Supplementary Table 7: The Catalytic Activity of Complex **F**, **G,** and **H**

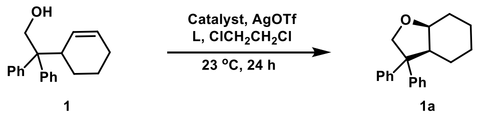

Entry	Catalyst	AgOTf (mol%)	L (mol%)	Yield (%)[a]
1	(dmfpf)$PtCl_2$ (1 mol%)	1	--	0
2	(dmfpf)$PtCl_2$ (1 mol%)	4	--	21
3	--	4	4	0
4	(dmfpf)$PtCl_2$ (1 mol%)	4	1	49

[a] Determined by ^{1}H NMR analysis using 4,4'-di-*tert*-butyl-1,1'biphenyl as an internal standard

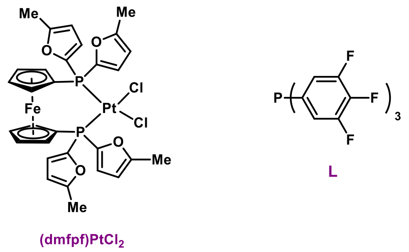

7.3.2 Investigation of the Catalytic Activity of Complex F

7.3.2.1 NMR Monitoring of Hydroalkoxylation of Norbornene and 4-Trifluromethylphenol Catalyzed by Complex F and Tris(3,4,5-trifluorophenyl)phosphane

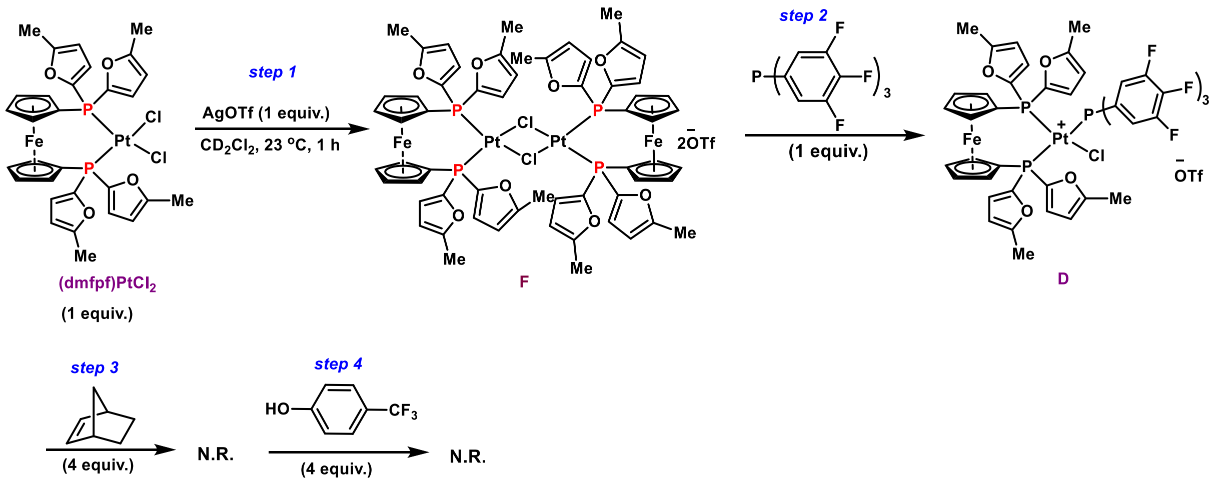

Step 1: In an argon filled glovebox, to a 4 mL vial with a magnetic stir bar were added (dmfpf)$PtCl_2$ (17 mg, 0.02 mmol), AgOTf (5 mg, 0.02 mmol) in CD_2Cl_2 (500 μL). The mixture was stirred at room temperature (23 °C) for 1 h. The mixture was recorded by ^{1}H NMR and ^{31}P NMR respectively. ^{31}P NMR chemical shifts are referenced to an external PPh_3 standard in CD_2Cl_2 sealed in a capillary tube. Analysis of the ^{31}P NMR showed that the new peaks at δ -22.5 ppm belonged to complex **F** (Supplementary Figure 19a).

Step 2: To the above solution was added tris(3,4,5-trifluorophenyl)phosphane (8.5 mg, 0.02 mol, 1.0 equiv.). The resulted solution was recorded by ^{1}H NMR and ^{31}P NMR respectively. The new three groups of peaks [δ 25.9 (dd, $^2J_{P\text{-}P}$ = 461, 18 Hz, $^1J_{Pt\text{-}P}$ = 2484 Hz), -10.5 (dd, $^2J_{P\text{-}P}$ = 461, 17 Hz, $^1J_{Pt\text{-}P}$ = 2735 Hz), -24.5 (t, $^2J_{P\text{-}P}$ = 18 Hz, $^1J_{Pt\text{-}P}$ = 3641 Hz) ppm] were formed, which belonged to complex **D** (Supplementary Figure 19b).

Step 3: To the above solution was added norbornene (7.5 mg, 0.08 mmol, 4 equiv.), the resulted solution was recorded by ^{1}H NMR and ^{31}P NMR respectively. Analysis of the ^{31}P NMR showed there was no change (Supplementary Figure 18d and 19c).

Step 4: To the above solution was added 4-trifluromethylphenol (13 mg, 0.08 mmol, 4 equiv.). No reaction occurred (Supplementary Figure 18e and 19d).

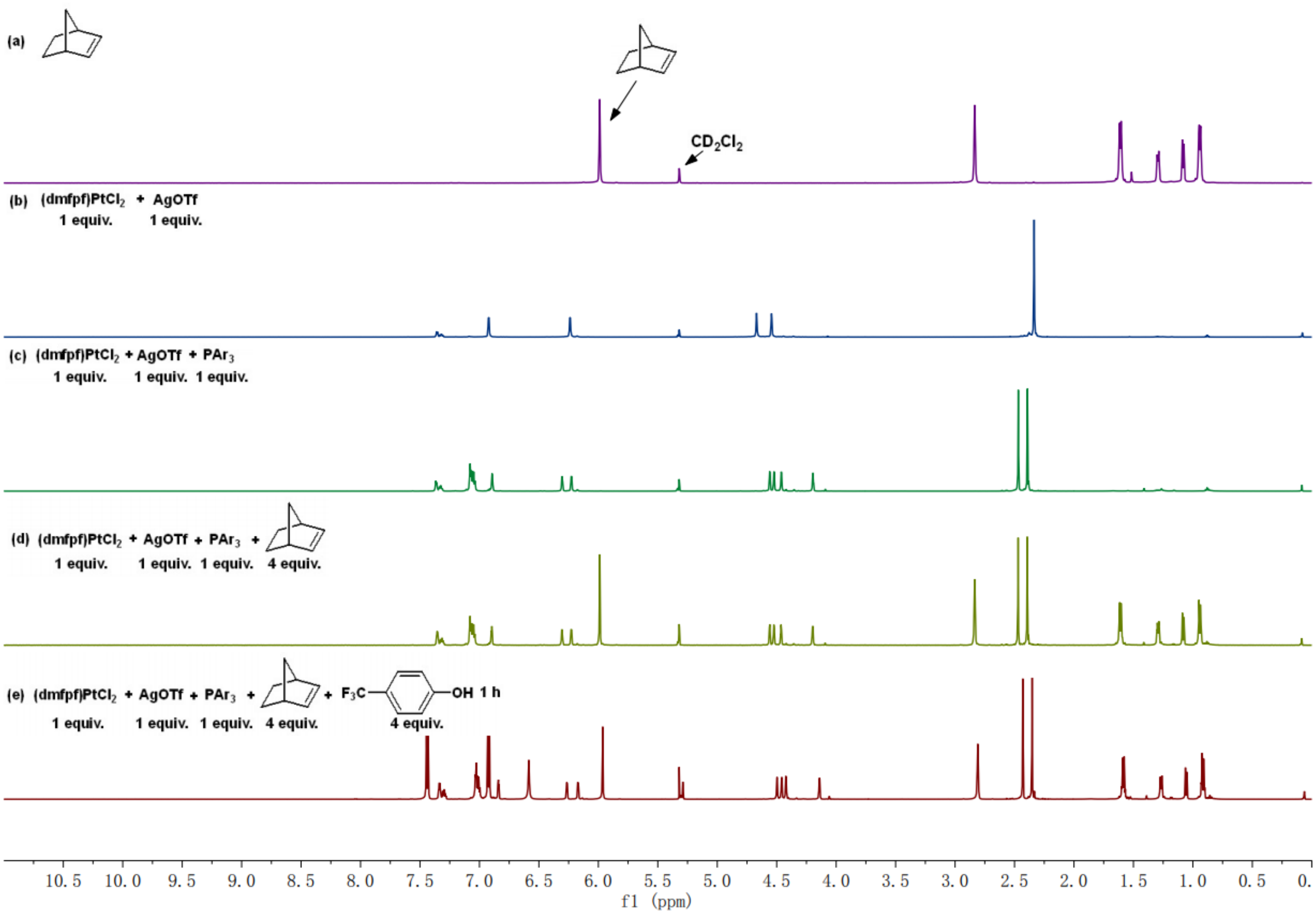

Supplementary Figure 18. ^{1}H NMR (600 MHz, CD_2Cl_2) Monitoring of Hydroalkoxylation of Norbornene and 4-Trifluromethylphenol Catalyzed by Complex **F** and Tris(3,4,5-trifluorophenyl)phosphane

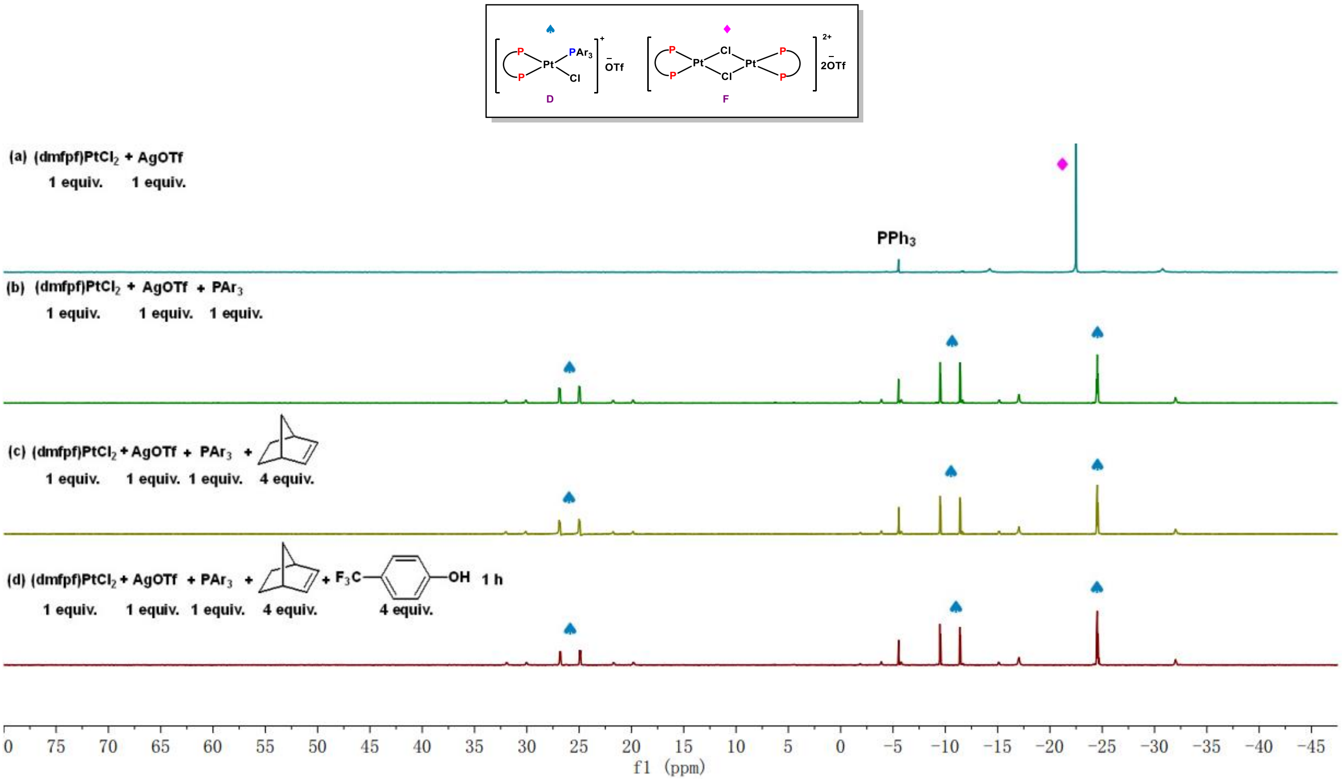

Supplementary Figure 19. ^{31}P NMR (243 MHz, CD_2Cl_2) Monitoring of Hydroalkoxylation of Norbornene and 4-Trifluromethylphenol Catalyzed by Complex **F** and Tris(3,4,5-trifluorophenyl)phosphane

7.3.2.2 NMR Monitoring of Hydroalkoxylation of Norbornene and 4-Trifluromethylphenol Catalyzed by Complex F

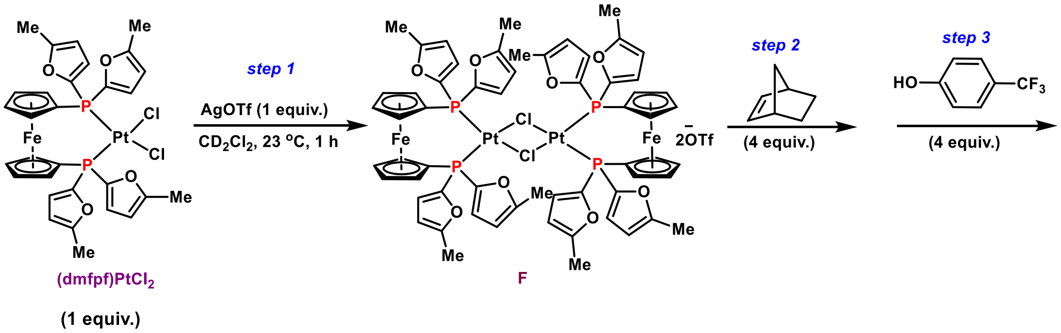

Step 1: In an argon filled glovebox, to a 4 mL vial with a magnetic stir bar were added (dmfpf)$PtCl_2$ (17 mg, 0.02 mmol), AgOTf (5 mg, 0.02 mmol) in CD_2Cl_2 (500 μL). The mixture was stirred at room temperature (23 °C) for 1 h. The mixture was recorded by ^{1}H NMR and ^{31}P NMR respectively. ^{31}P NMR chemical shifts are referenced to an external PPh_3 standard in CD_2Cl_2 sealed in a capillary tube.

Step 2: To the above solution was added norbornene (7.5 mg, 0.08 mmol, 4 equiv.), the resulted solution was recorded by ^{1}H NMR and ^{31}P NMR respectively. Analysis of the ^{31}P NMR showed there was no change (Supplementary Figure 20c and 21b).

Step 3: To the above solution was added 4-trifluromethylphenol (13 mg, 0.08 mmol, 4 equiv.). hydroalkoxylation product **23b** was obtained in 50% yield after 1 h (Supplementary Figure 20d).

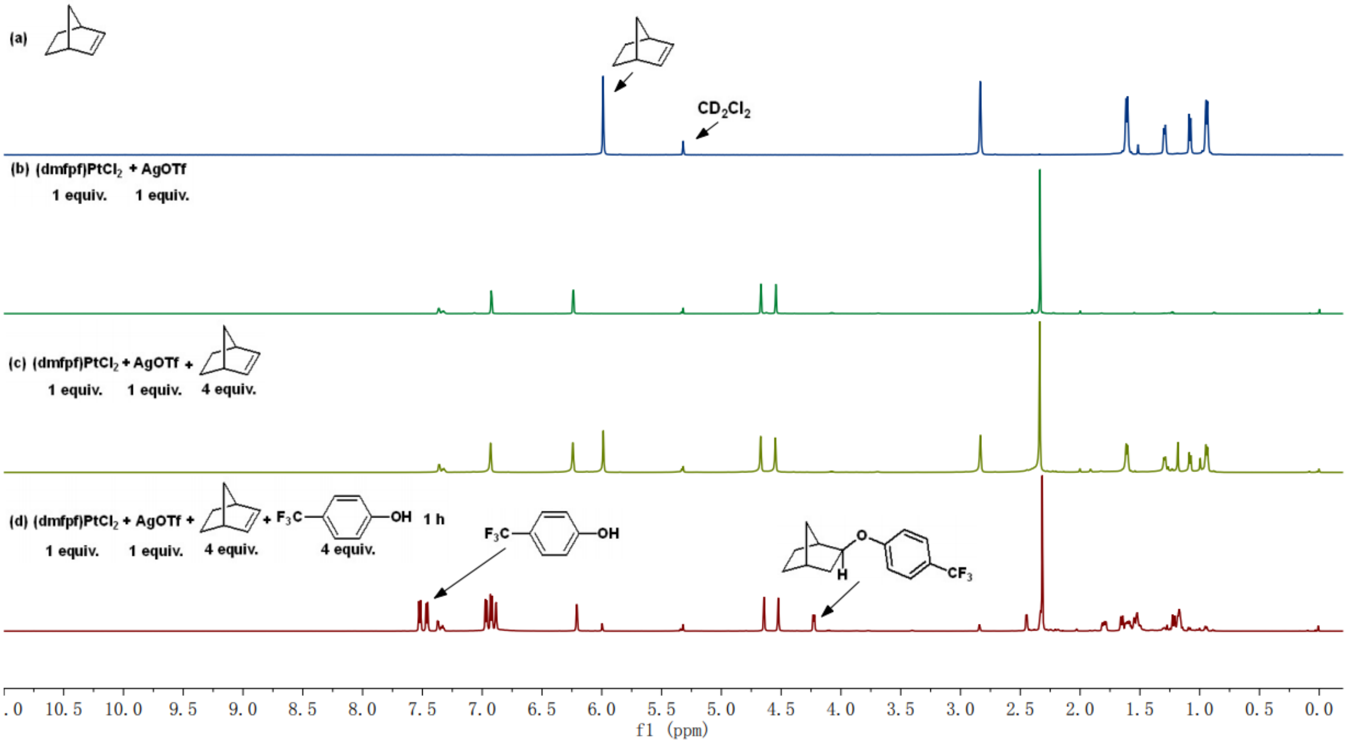

Supplementary Figure 20. ^{1}H NMR (600 MHz, CD_2Cl_2) Monitoring of Hydroalkoxylation of Norbornene and 4-Trifluromethylphenol Catalyzed by Complex **F**

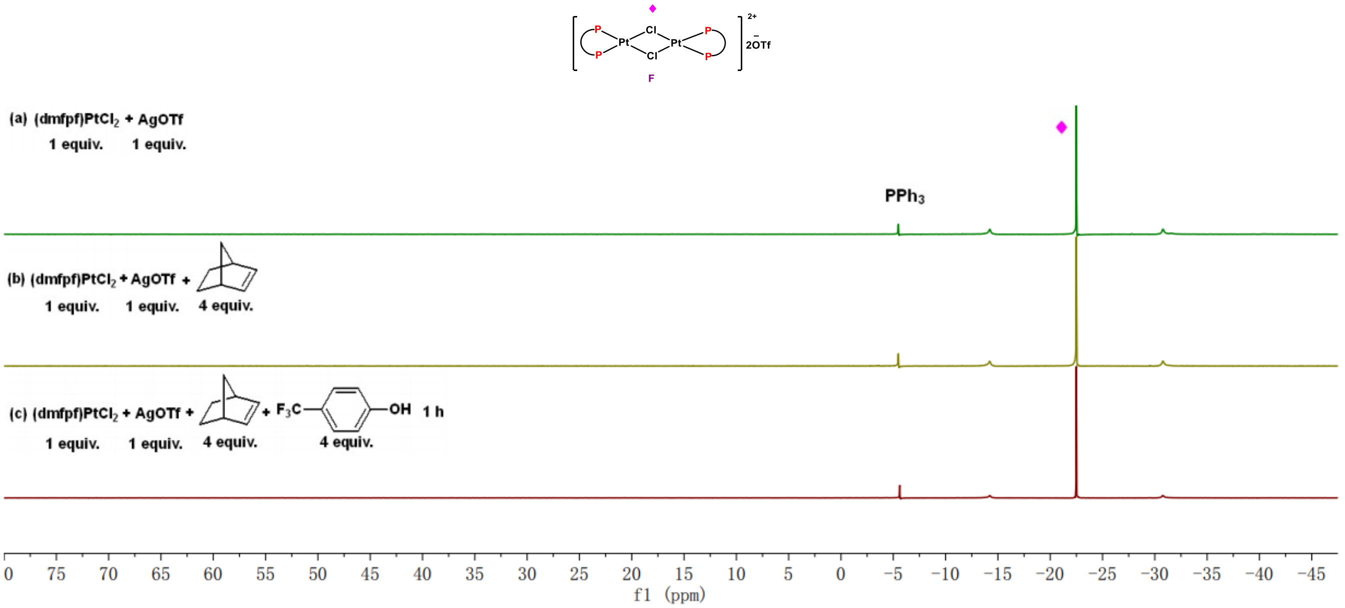

Supplementary Figure 21. ^{31}P NMR (243 MHz, CD_2Cl_2) Monitoring of Hydroalkoxylation of Norbornene and 4-Trifluromethylphenol Catalyzed by Complex **F**

7.3.3 Investigation of the Catalytic Activity of Complex G

7.3.3.1 NMR Monitoring of Hydroalkoxylation of Norbornene and 4-Trifluromethylphenol Catalyzed by Unfiltered Complex G

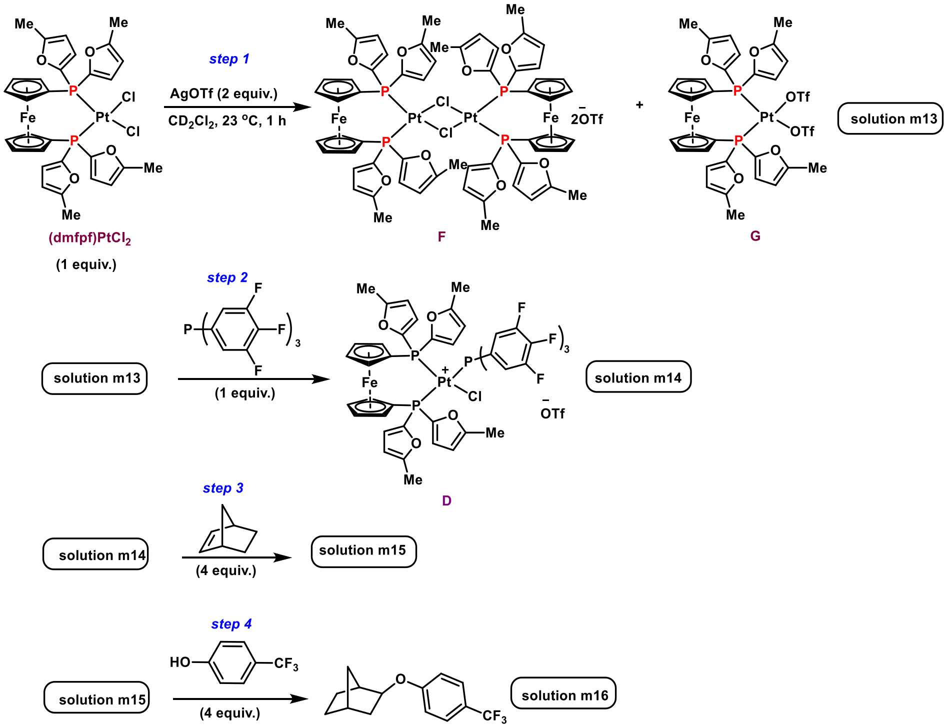

Step 1: In an argon filled glovebox, to a 4 mL vial with a magnetic stir bar were added (dmfpf)$PtCl_2$ (17 mg, 0.02 mmol), AgOTf (10 mg, 0.04 mmol) and CD_2Cl_2 (500 μL). The mixture was stirred at room temperature (23 °C) for 1 h. The mixture was recorded by ^{1}H NMR and ^{31}P NMR respectively. ^{31}P NMR chemical shifts are referenced to an external PPh_3 standard in CD_2Cl_2 sealed in a capillary tube. Analysis of the ^{31}P NMR showed that the new peaks at δ -22.5 ppm belonged to complex **F** and δ -33.5 ppm belonged to complex **G** (Supplementary Figure 23a).

Step 2: To the above solution was added tris(3,4,5-trifluorophenyl)phosphane (8.5 mg, 0.02 mol, 1.0 equiv.). The resulted solution was recorded by ^{1}H NMR and ^{31}P NMR respectively. The new three groups of peaks [δ 25.9 (dd, $^2J_{P\text{-}P}$ = 461, 18 Hz, $^1J_{Pt\text{-}P}$ = 2484 Hz), -10.5 (dd, $^2J_{P\text{-}P}$ = 461, 17 Hz, $^1J_{Pt\text{-}P}$ = 2735 Hz), -24.5 (t, $^2J_{P\text{-}P}$ = 18 Hz, $^1J_{Pt\text{-}P}$ = 3641 Hz) ppm] were formed, which belonged to complex **D**. And the new peaks at δ 15.8 ppm and 12.8 ppm were formed, which belonged complex **H** (Supplementary Figure 23b).

Step 3: To the above solution m18 was added norbornene (7.5 mg, 0.08 mmol, 4 equiv.). The resulted solution was recorded by ^{1}H NMR and ^{31}P NMR respectively. Analysis of the ^{31}P NMR showed complex **G** and complex **F** disappeared. Analysis of the ^{1}H NMR showed the ^{1}H signal of the double bond moved downfield from 5.99 ppm to 6.14 ppm (Supplementary Figure 22d and S24).

Step 4: To the above solution was added 4-trifluromethylphenol (13 mg, 0.08 mmol, 4 equiv.). Analysis of the ^{1}H NMR showed that hydroalkoxylation product **23b** was obtained after 1 h (Supplementary Figure 23e).

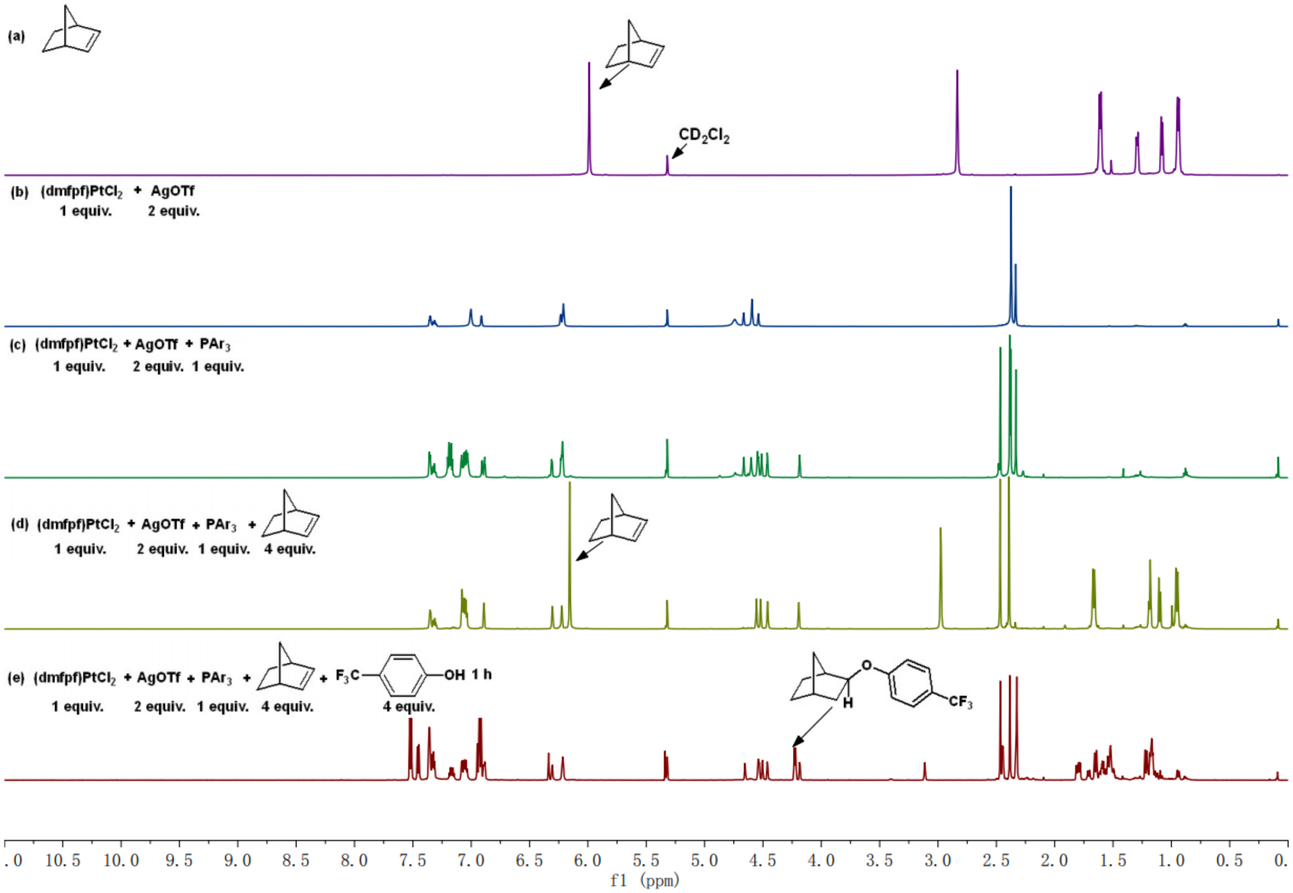

Supplementary Figure 22. ^{1}H NMR (600 MHz, CD_2Cl_2) Monitoring of Hydroalkoxylation of Norbornene and 4-Trifluromethylphenol Catalyzed by Complex **G**

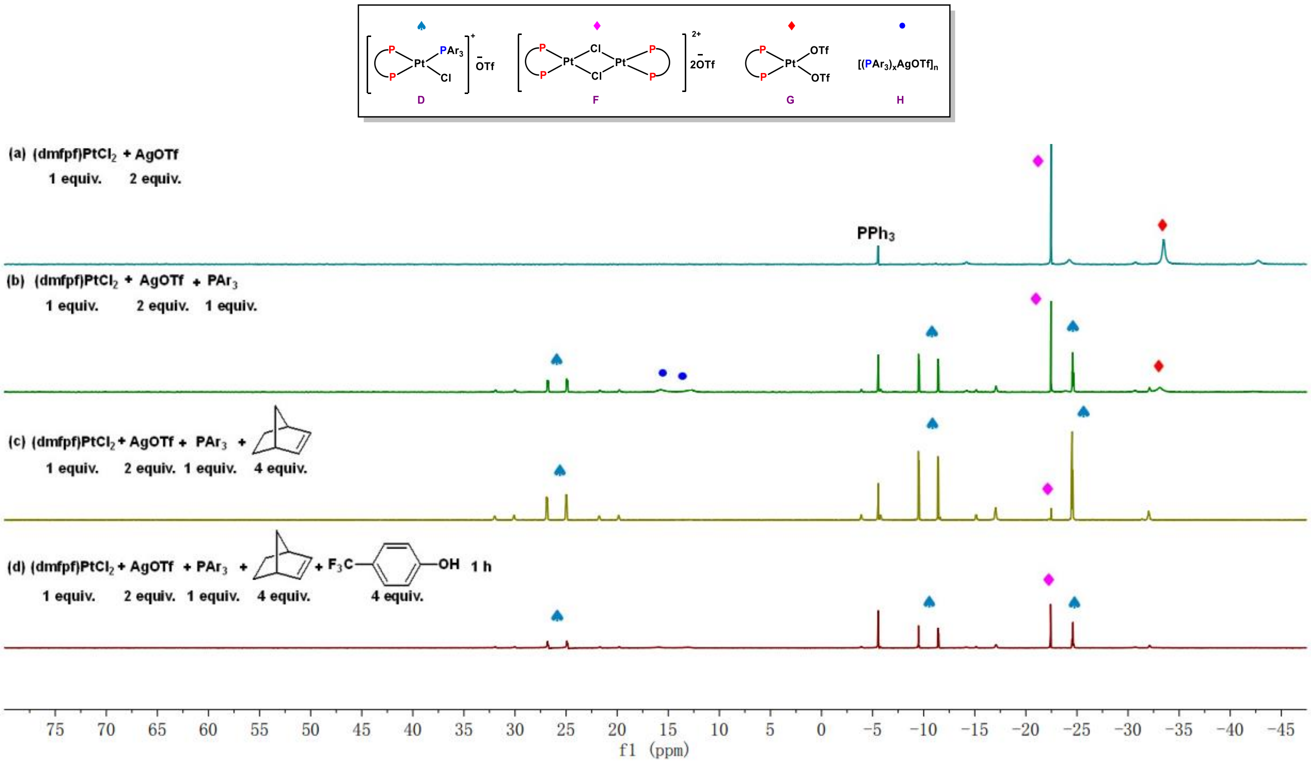

Supplementary Figure 23. ^{31}P NMR (243 MHz, CD_2Cl_2) Monitoring of Hydroalkoxylation of Norbornene and 4-TriflurometHylphenol Catalyzed by Complex **G**

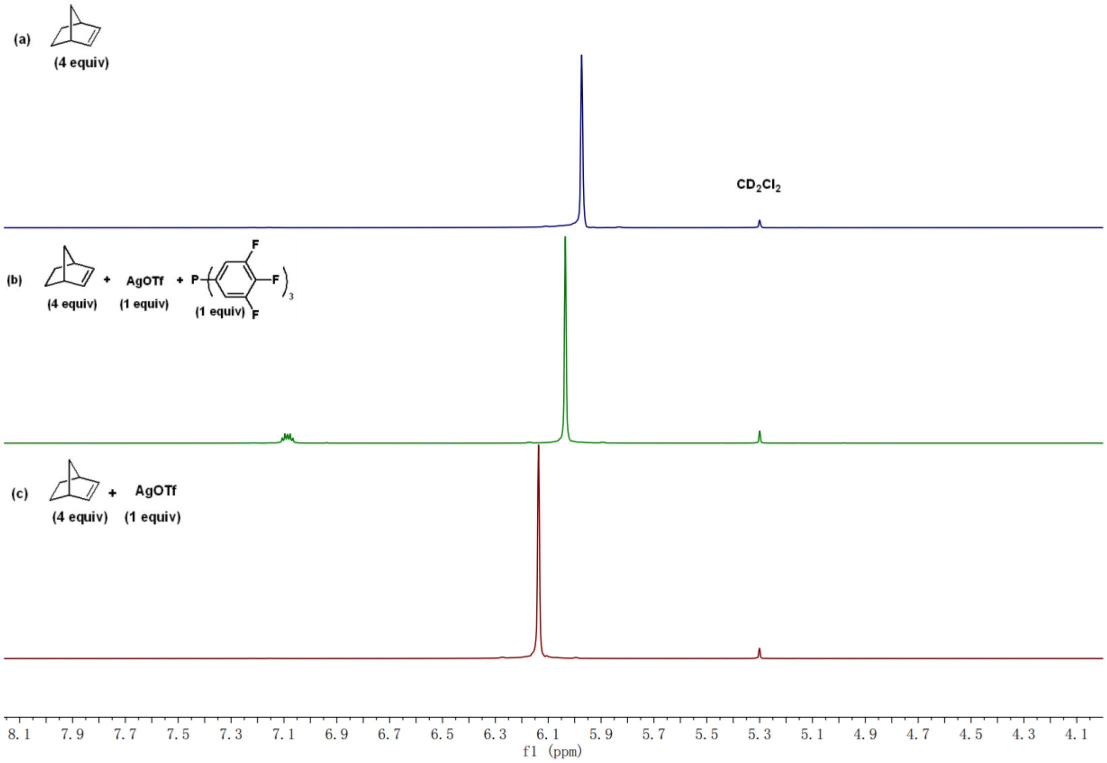

Supplementary Figure 24. Comparison of ^{1}H NMRs of Norbornene, AgOTf + Monodentate Phosphine + Norbornene and Norbornene + AgOTf

7.3.3.2 NMR Monitoring of Hydroalkoxylation of Norbornene and 4-Trifluromethylphenol Catalyzed by Filtered Complex G

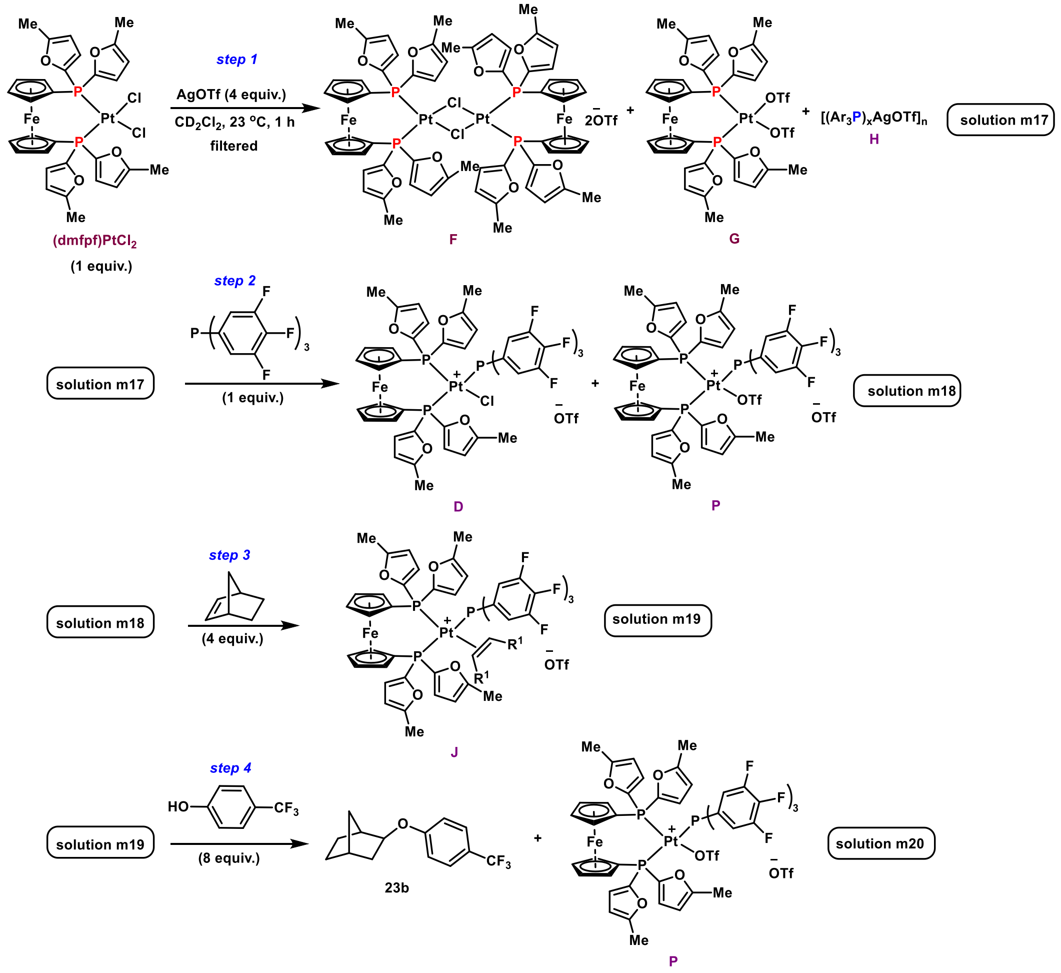

Step 1: In an argon filled glovebox, to a 4 mL vial with a magnetic stir bar were added (dmfpf)$PtCl_2$ (17 mg, 0.02 mmol), AgOTf (20 mg, 0.08 mmol) and CD_2Cl_2 (500 μL). The mixture was stirred at room temperature (23 °C) for 1 h. The mixture was filtered and filtrate was recorded by ^{1}H NMR and ^{31}P NMR respectively. ^{31}P NMR chemical shifts are referenced to an external PPh_3 standard in CD_2Cl_2 sealed in a capillary tube. Analysis of the ^{31}P NMR showed that the new peaks at δ -22.4 ppm belonged to complex **F** and δ -33.2 ppm belonged to complex **G** (Supplementary Figure 26a).

Step 2: To the above solution was added monophosphine ligand tris(3,4,5-trifluorophenyl)phosphane (8.5 mg, 0.02 mol, 1.0 equiv.). The resulted solution was recorded by ^{1}H NMR and ^{31}P NMR respectively. The new three groups of peaks [δ 25.9 (dd, $^2J_{P\text{-}P}$ = 461, 18 Hz, $^1J_{Pt\text{-}P}$ = 2484 Hz), -10.5 (dd, $^2J_{P\text{-}P}$ = 461, 17 Hz, $^1J_{Pt\text{-}P}$ = 2735 Hz), -24.5 (t, $^2J_{P\text{-}P}$ = 18 Hz, $^1J_{Pt\text{-}P}$ = 3641 Hz) ppm] were formed, which belonged to complex **D**. And the new peaks at δ 16.0 ppm,12.9 ppm were formed, which belonged complex **H**. Another new three groups of peaks [δ 25.8 (d, $^2J_{P\text{-}P}$ = 411 Hz, $^1J_{Pt\text{-}P}$ = 2584 Hz), -6.2 (d, $^2J_{P\text{-}P}$ = 411, $^1J_{Pt\text{-}P}$ = 2745 Hz),

-29.9 (s, broad, $^{1}J_{\text{Pt-P}}$ = 4289 Hz) ppm] were formed, which might belong to complex **P** (Supplementary Figure 26b).

Step 3: To the above solution was added norbornene (7.5 mg, 0.08 mmol, 4 equiv.), and the resulted solution was recorded by ^{1}H NMR and ^{31}P NMR respectively. The three new groups of peaks [δ 33.6 (dd, $^{2}J_{\text{P-P}}$ = 410, 22 Hz, $^{1}J_{\text{Pt-P}}$ = 3075), -12.6 (dd, $^{2}J_{\text{P-P}}$ = 411, 23 Hz, $^{1}J_{\text{Pt-P}}$ = 3463), -17.2 (t, $^{2}J_{\text{P-P}}$ = 22 Hz, $^{1}J_{\text{Pt-P}}$ = 1873)] were formed, which could belong to intermediate **J** (Supplementary Figure 26c).

Step 4: To the above solution was added 4-trifluromethylphenol (26 mg, 0.16 mmol, 8 equiv.). Analysis of the ^{1}H NMR showed that the product **23b** was obtained after 1 h (Supplementary Figure 25e).

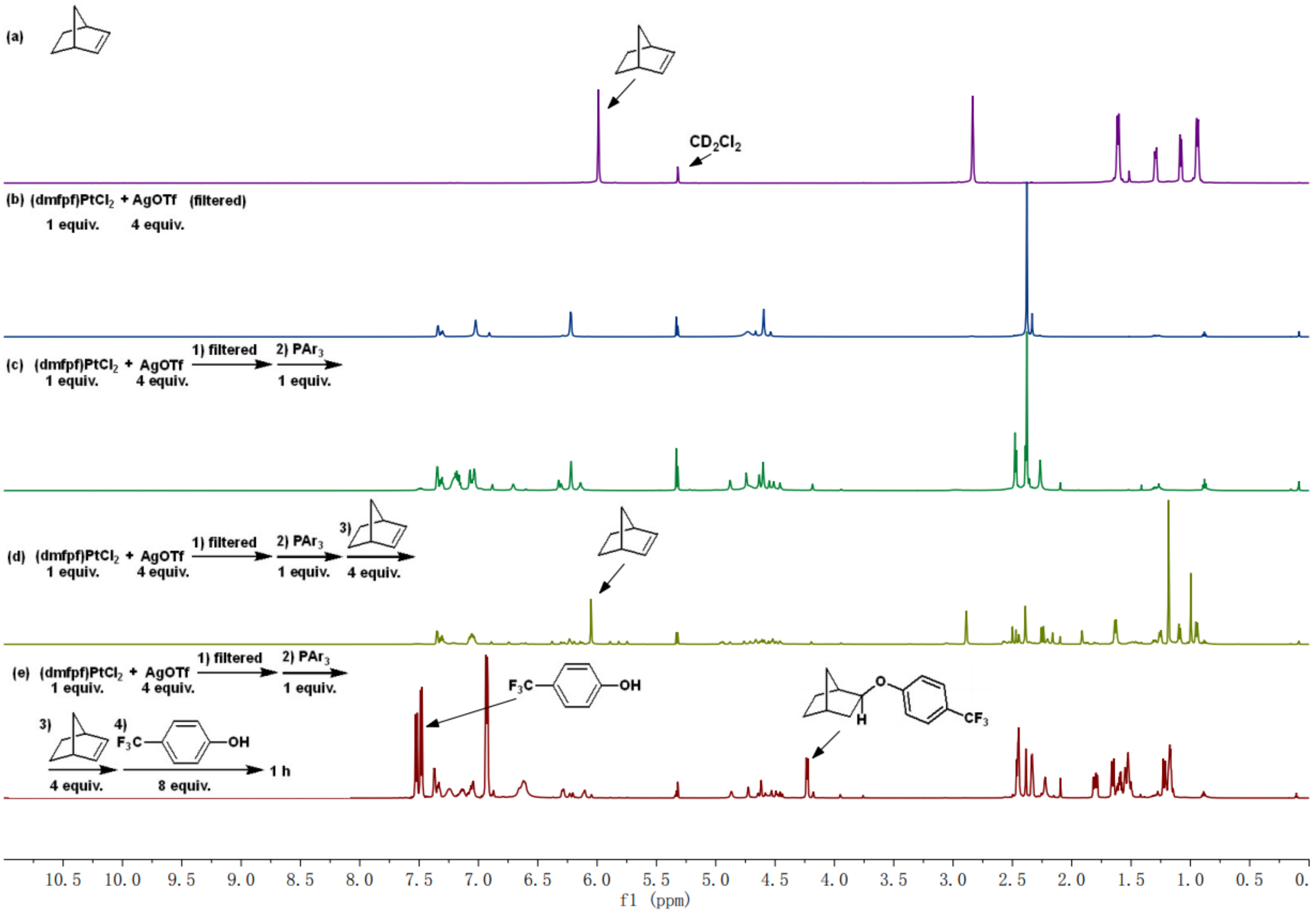

Supplementary Figure 25. ^{1}H NMR (600 MHz, CD_2Cl_2) Monitoring of Hydroalkoxylation of Norbornene and 4-Trifluromethylphenol Catalyzed by Filtered Complex **G**

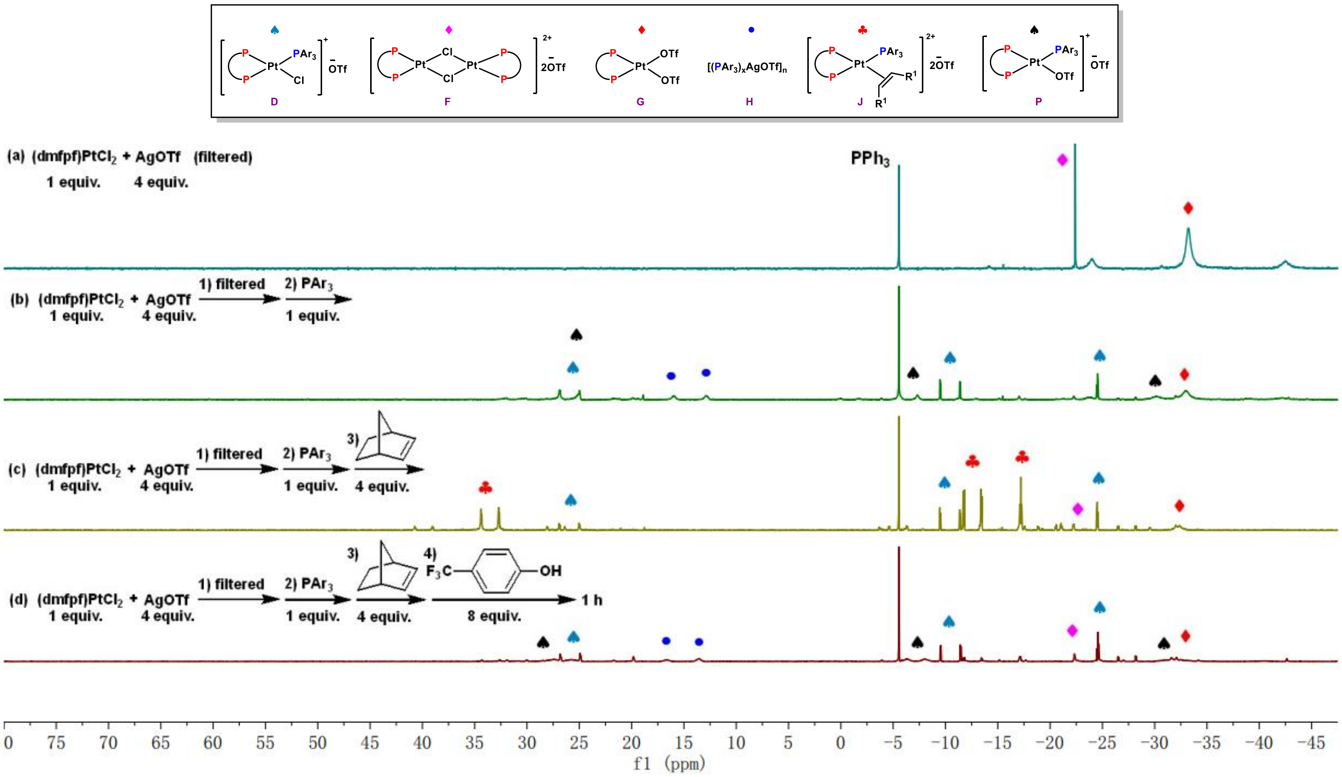

Supplementary Figure 26. ^{31}P NMR (243 MHz, CD_2Cl_2) Monitoring of Hydroalkoxylation of Norbornene and 4-Trifluromethylphenol Catalyzed by Filtered Complex **G**

8. Kinetic Experiments

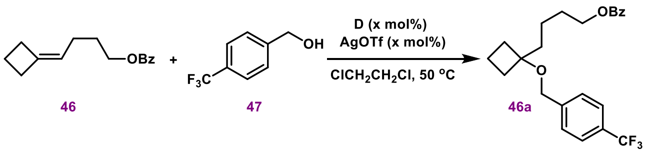

For catalyst concentration

In an argon filled glovebox, to a 4 mL vial with a magnetic stir bar were added the catalyst **D** (1 μmol, 2 μmol, 4 μmol, 8 μmol, 16 μmol), AgOTf (1.0 equiv. relative to Pt), $ClCH_2CH_2Cl$ (1.0 mL) and the mixture was stirred at room temperature (23 °C) for 1 hour. A solution of 4-cyclobutylidenebutyl benzoate **46**(0.2 mmol, 46 mg), (4-(trifluoromethyl)phenyl)methanol **47** (0.2 mmol, 35 mg) and 4,4'-di-*tert*-butyl-1,1'-biphenyl (4 μmol) used as an internal standard in $ClCH_2CH_2Cl$ (1 mL) was added. The reaction was stirred at 50 °C. Periodically, an aliquot was removed from the tube and analyzed by LCMS. The experimental results show that the reaction rates for the catalytic Hydroalkoxylation of olefin **46** is first-order in the Pt catalyst **D** (Supplementary Figure 27).

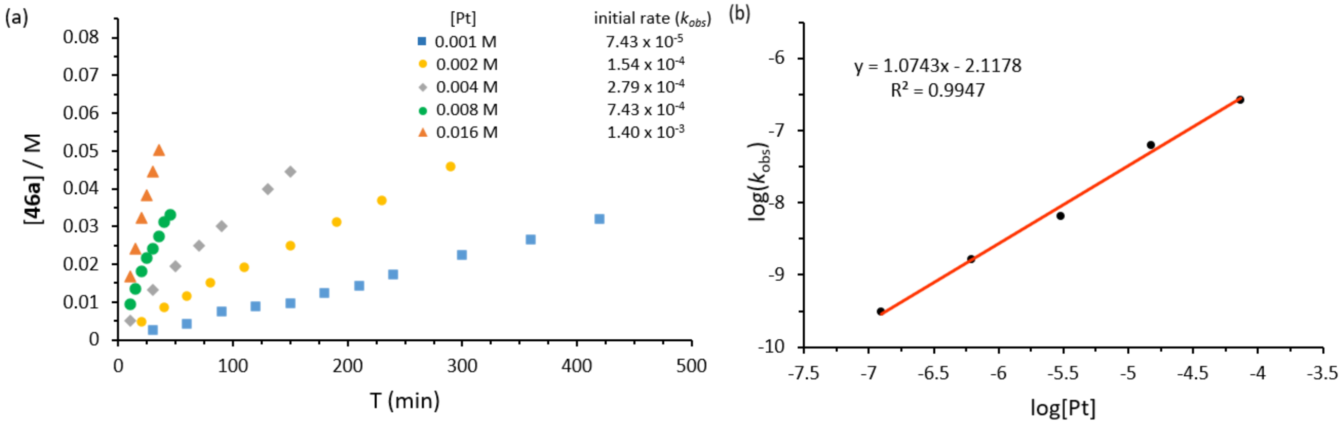

Supplementary Figure 27. Kinetic Analysis. **a** Plot of Initial Reaction Rates at Various Concentrations of Catalyst **D**. **b** Log-Log Plot of Initial Reaction Rates as A Function of Catalyst **D** Concentration.

For 4-cyclobutylidenebutyl benzoate concentration

In an argon filled glovebox, to a 4 mL vial with a magnetic stir bar were added the catalyst **D** (8 μmol, 11.0 mg), AgOTf (9.6 μmol, 2.5 mg), $ClCH_2CH_2Cl$ (1.0 mL) in a 4 mL tube and the mixture was stirred at room temperature (23 °C) for 1 hour. And then a solution of 4-cyclobutylidenebutyl benzoate **46** (0.02 mmol, 0.05 mmol, 0.1 mmol, 0.15 mmol, 0.2 mmol), (4-(trifluoromethyl)phenyl)methanol **47** (0.4 mmol, 70.5 mg) and 4,4'-di-*tert*-butyl-1,1'-biphenyl (2 mol% relative to **46**) in $ClCH_2CH_2Cl$ (1 mL) was added. The reaction was stirred at 50 °C. Periodically, an aliquot was removed from the tube and analyzed by LCMS. Under low [**46**] concentration (0.01-0.05 M), a positive linear dependence on [**46**] (slope: 0.72) was observed. In the concentration range of 0.05-0.1 M, a positive linear with a lower slope (0.34) dependence on [**46**] was also obtained (Supplementary Figure 28).

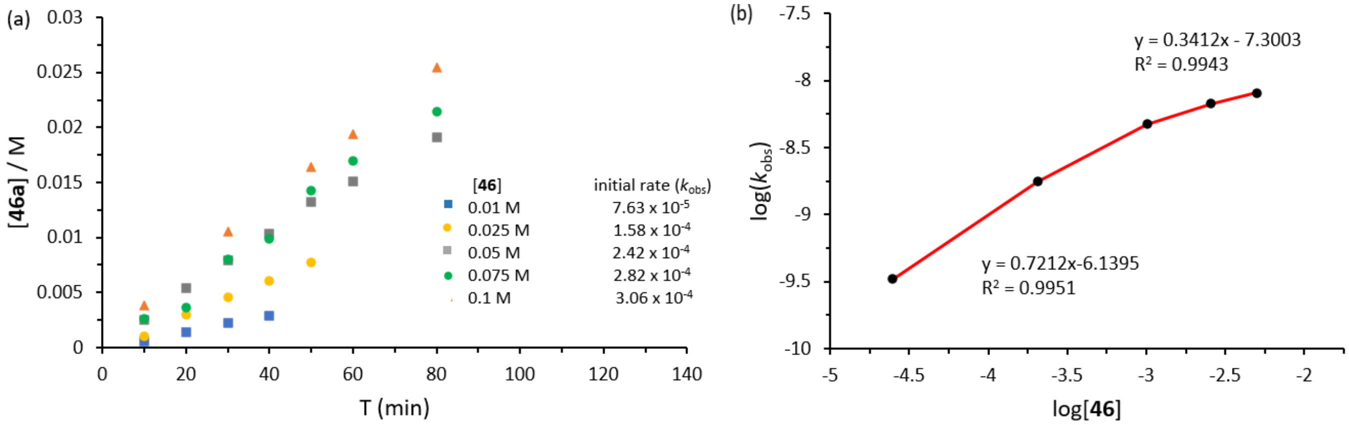

Supplementary Figure 28. Kinetic Analysis. **a** Plot of Initial Reaction Rates at Various Concentrations of 4-Cyclobutylidenebutyl Benzoate. **b** Log-Log Plot of Initial Reaction Rates as A Function of 4-Cyclobutylidenebutyl Benzoate Concentration.

For (4-(trifluoromethyl)phenyl)methanol concentration

In an argon filled glovebox, to a 4 mL vial with a magnetic stir bar were added the catalyst **D** (8 μmol, 11.0 mg), AgOTf (9.6 μmol, 2.5 mg), $ClCH_2CH_2Cl$ (1.0 mL) in a 4 mL tube and the mixture was stirred at room temperature (23 °C) for 1 hour. Then 4-cyclobutylidenebutyl benzoate **46** (0.4 mmol, 92.0 mg), (4-(trifluoromethyl)phenyl)methanol **47** (0.02 mmol, 0.05 mmol, 0.1 mmol, 0.15 mmol, 0.2 mmol), and 4,4'-di-*tert*-butyl-1,1'-biphenyl (2 mol% relative to **47**) used as an internal standard in $ClCH_2CH_2Cl$ (1 mL) was added. The reaction was stirred at 50 °C. Periodically, an aliquot was removed from the tube and analyzed by LCMS. The experimental results show that the reaction rates for the catalytic Hydroalkoxylation of olefin **46** is first-order dependence on [**47**] (Supplementary Figure 29).

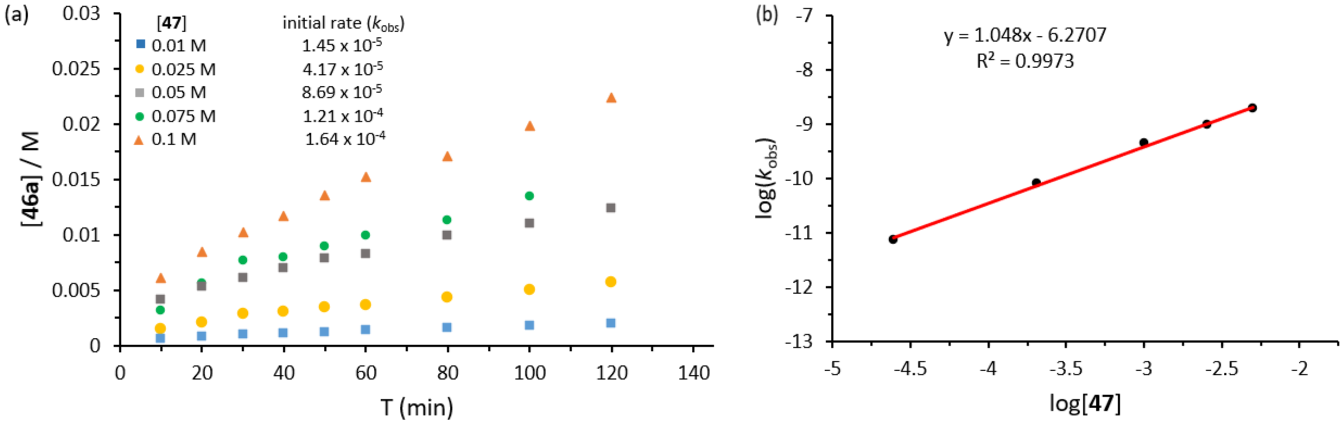

Supplementary Figure 29. Kinetic Analysis. **a** Plot of Initial Reaction Rates at Various Concentrations of (4-(Trifluoromethyl)Phenyl)Methanol. **b** Log-log Plot of Initial Reaction Rates as A Function of (4-(Trifluoromethyl)Phenyl)Methanol Concentration.

Kinetic Isotope Effects

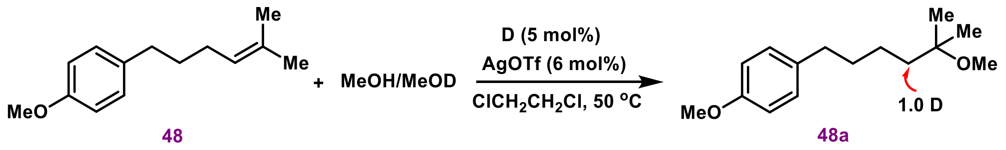

In an argon filled glovebox, to a 4 mL vial with a magnetic stir bar were added the catalyst **D** (10 μmol, 13.7 mg), AgOTf (12 μmol, 3.1 mg), $ClCH_2CH_2Cl$ (1.0 mL) in a 4 mL tube and the mixture was stirred at room temperature (23 °C) for 1 hour. A solution of 1-methoxy-4-(5-methylhex-4-en-1-yl)benzene **48** (0.2 mmol, 41.0 mg), MeOH or MeOD (1.4 mmol) and 4,4'-di-*tert*-butyl-1,1'-biphenyl (0.5 equiv. relative to **48**) used as an internal standard in $ClCH_2CH_2Cl$ (1 mL) was added. The reaction was stirred at 50 °C. Periodically, an aliquot was removed from the tube and analyzed by ^{1}H NMR (Supplementary Figure 30).

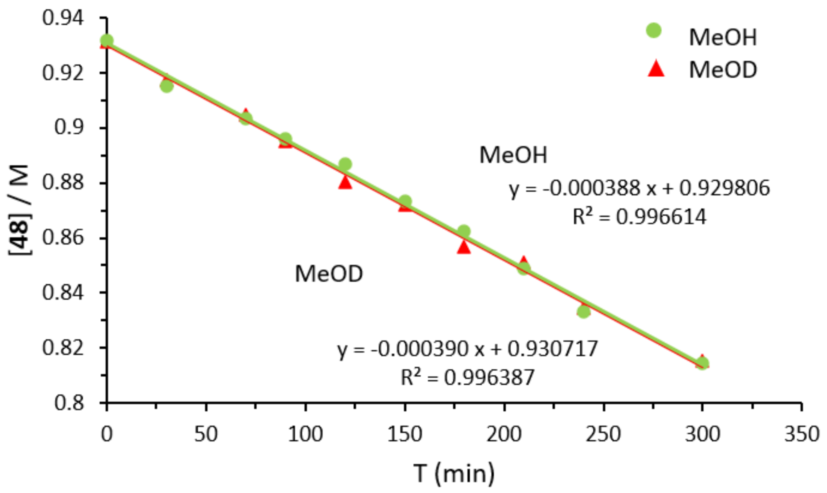

Supplementary Figure 30. KIE Measurements for Intermolecular Hydroalkoxylation KIE = k_{MeOH}/k_{MeOD} = 0.99

9. X-Ray Structure Determination

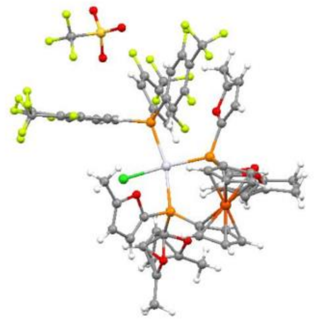

Supplementary Figure 31. Structure of **Catalyst E** with 50% Probability Anisotropic Displacement Ellipsoids

Supplementary Table 8: Crystal Data and Structure Refinement for Catalyst **E**

CCDC Number	1942024
Empirical formula	$C_{52}H_{34}ClF_{18}FeO_7P_3PtS$
Formula weight	1524.15
Temperature/K	100
Crystal system	triclinic
Space group	P-1
a/Å	14.2361(9)
b/Å	15.0250(10)
c/Å	16.8881(11)
α/°	65.753(2)
β/°	88.283(2)
γ/°	66.531(2)
Volume/Å^3	2982.4(3)
Z	2
ρ_{calc}g/cm^3	1.697
μ/mm^{-1}	2.850
F(000)	1492.0
Crystal size/mm^3	0.45 × 0.36 × 0.28
Radiation	MoKα (λ = 0.71073)
2Θ range for data collection/°	4.458 to 55.136

Index ranges	-18 ⩽ h ⩽ 18, -19 ⩽ k ⩽ 19, -21 ⩽ l ⩽ 21
Reflections collected	41881
Independent reflections	13722 [R_{int} = 0.0441, R_{sigma} = 0.0480]
Data/restraints/parameters	13722/63/806
Goodness-of-fit on F^2	1.088
Final R indexes [I>=2σ (I)]	R_1 = 0.0435, wR_2 = 0.0909
Final R indexes [all data]	R_1 = 0.0569, wR_2 = 0.0993
Largest diff. peak/hole / e $Å^{-3}$	2.14/-2.36

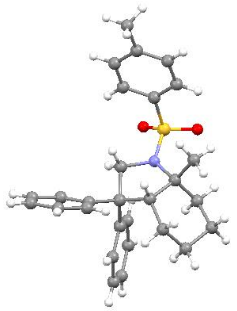

Supplementary Figure 32. Structure of **41a** with 50% Probability Anisotropic Displacement Ellipsoids

Supplementary Table 9: Crystal Data and Structure Refinement for **41a**

CCDC Number	1945616
Empirical formula	$C_{28.5}H_{32}ClNO_2S$
Formula weight	488.06
Temperature/K	100.03
Crystal system	orthorhombic
Space group	$Pca2_1$
a/Å	17.9334(10)
b/Å	14.9360(8)
c/Å	18.5885(9)
α/°	90
β/°	90
γ/°	90
Volume/$Å^3$	4979.0(5)

Z	8
ρ_{calc}g/cm^3	1.302
μ/mm^{-1}	0.264
F(000)	2072.0
Crystal size/mm^3	0.51 × 0.48 × 0.08
Radiation	MoKα (λ = 0.71073)
2Θ range for data collection/°	4.17 to 55.154
Index ranges	-23 ≤ h ≤ 23, -19 ≤ k ≤ 19, -24 ≤ l ≤ 24
Reflections collected	74178
Independent reflections	11299 [R_{int} = 0.0927, R_{sigma} = 0.0605]
Data/restraints/parameters	11299/1/608
Goodness-of-fit on F^2	0.997
Final R indexes [I>=2σ (I)]	R_1 = 0.0354, wR_2 = 0.0804
Final R indexes [all data]	R_1 = 0.0542, wR_2 = 0.0860
Largest diff. peak/hole / e Å^{-3}	0.28/-0.35
Flack parameter	0.001(16)

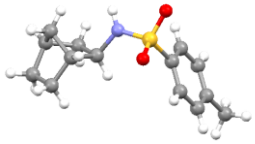

Supplementary Figure 33. Structure of **23f** with 50% Probability Anisotropic Displacement Ellipsoids

Supplementary Table 10: Crystal Data and Structure Refinement for **23f**

CCDC Number	1942471
Empirical formula	$C_{14.01}H_{19.01}NO_2S$
Formula weight	265.45
Temperature/K	100
Crystal system	monoclinic
Space group	C2/c
a/Å	19.0935(12)
b/Å	8.2000(5)

c/Å	18.4861(11)
α/°	90
β/°	112.539(2)
γ/°	90
Volume/Å^3	2673.2(3)
Z	8
ρ_{calc}g/cm^3	1.319
μ/mm^{-1}	0.236
F(000)	1136.0
Crystal size/mm^3	0.34 × 0.32 × 0.29
Radiation	MoKα (λ = 0.71073)
2Θ range for data collection/°	4.62 to 55.012
Index ranges	-22 ⩽ h ⩽ 24, -10 ⩽ k ⩽ 10, -24 ⩽ l ⩽ 21
Reflections collected	12215
Independent reflections	3073 [R_{int} = 0.0453, R_{sigma} = 0.0376]
Data/restraints/parameters	3073/734/294
Goodness-of-fit on F^2	1.044
Final R indexes [I>=2σ (I)]	R_1 = 0.0483, wR_2 = 0.1244
Final R indexes [all data]	R_1 = 0.0649, wR_2 = 0.1374
Largest diff. peak/hole / e Å^{-3}	0.40/-0.56

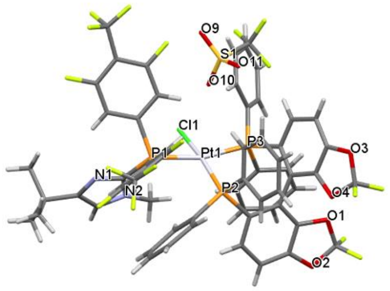

Supplementary Figure 34. Structure of Catalyst **M** with 50% Probability Anisotropic Displacement Ellipsoids

Supplementary Table 11: Crystal Data and Structure Refinement for Catalyst **M**

CCDC Number	2021353
Empirical formula	$C_{61}H_{41}ClF_{17}N_2O_7P_3PtS$
Formula weight	1591.09
Temperature/K	100
Crystal system	triclinic
Space group	P1
a/Å	13.6752(9)
b/Å	16.8453(11)
c/Å	18.9222(12)
α/°	70.156(2)
β/°	89.890(2)
γ/°	86.615(2)
Volume/Å^3	4092.2(5)
Z	2
ρ_{calc}g/cm^3	1.479
μ/mm^{-1}	1.922
F(000)	1822.0
Crystal size/mm^3	0.32 × 0.29 × 0.16
Radiation	MoKα (λ = 0.71073)
2Θ range for data collection/°	4.578 to 59.264
Index ranges	-18 ≤ h ≤ 19, -23 ≤ k ≤ 23, -26 ≤ l ≤ 26
Reflections collected	111371
Independent reflections	45537 [Rint = 0.0415, Rsigma = 0.0568]
Data/restraints/parameters	45537/308/1990
Goodness-of-fit on F^2	1.033
Final R indexes [I>=2σ (I)]	R_1 = 0.0429, wR_2 = 0.1010
Final R indexes [all data]	R_1 = 0.0505, wR_2 = 0.1052
Largest diff. peak/hole / e Å^{-3}	1.71/-1.38
Flack parameter	-0.009(2)

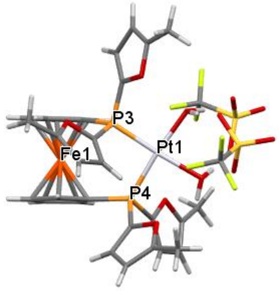

Supplementary Figure 35. Structure of Complex **R** with 50% Probability Anisotropic Displacement Ellipsoids

Supplementary Table 12: Crystal Data and Structure Refinement for Complex **R**

CCDC Number	2053041
Empirical formula	$C_{30}H_{32}FeO_6P_2Pt$
Formula weight	801.0671
Temperature/K	100
Crystal system	triclinic
Space group	P1
a/Å	10.3493(5)
b/Å	10.4314(5)
c/Å	20.0476(10)
α/°	91.063(2)
β/°	99.470(2)
γ/°	112.8780(10)
Volume/Å^3	1958.74(17)
Z	2
ρ_{calc}g/cm^3	1.864
μ/mm^{-1}	4.213
F(000)	1080.0
Crystal size/mm^3	0.32 × 0.29 × 0.05
Radiation	MoKα (λ = 0.71073)
2Θ range for data collection/°	4.136 to 56.752
Index ranges	-13 ⩽ h ⩽ 13, -13 ⩽ k ⩽ 13, -26 ⩽ l ⩽ 26
Reflections collected	38311

Independent reflections	9779 [R_{int} = 0.0554, R_{sigma} = 0.0439]
Data/restraints/parameters	9779/0/512
Goodness-of-fit on F^2	1.044
Final R indexes [I>=2σ (I)]	R_1 = 0.0254, wR2 = 0.0558
Final R indexes [all data]	R_1 = 0.0304, wR2 = 0.0577
Largest diff. peak/hole / e $Å^{-3}$	1.42/-1.45

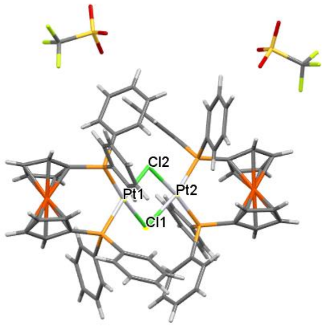

Supplementary Figure 36. Structure of Catalyst **Q** with 50% Probability Anisotropic Displacement Ellipsoids

Supplementary Table 13: Crystal Data and Structure Refinement for Catalyst **Q**

CCDC Number	2053040
Empirical formula	C70H56Cl2F6Fe2O6P4Pt2S2
Formula weight	1865.9744
Temperature/K	100
Crystal system	triclinic
Space group	P-1
a/Å	13.0484(3)
b/Å	16.9540(4)
c/Å	19.2928(5)
α/°	80.1960(10)
β/°	76.9990(10)
γ/°	76.8210(10)
Volume/$Å^3$	4017.10(17)
Z	2

ρ_{calc}g/cm^3	1.808
μ/mm^{-1}	13.093
F(000)	2153.0
Crystal size/mm^3	0.31 × 0.24 × 0.22
Radiation	MoKα (λ = 1.54178)
2Θ range for data collection/°	4.738 to 136.848
Index ranges	-14 ⩽ h ⩽ 15, -20 ⩽ k ⩽ 20, -23 ⩽ l ⩽ 23
Reflections collected	58377
Independent reflections	14714 [R_{int} = 0.0617, R_{sigma} = 0.0502]
Data/restraints/parameters	14714/199/1052
Goodness-of-fit on F^2	1.055
Final R indexes [I>=2σ (I)]	R_1 = 0.0459, wR2 = 0.1226
Final R indexes [all data]	R_1 = 0.0500, wR2 = 0.1259
Largest diff. peak/hole / e Å^{-3}	2.83/-1.85

10. Supplementary References

1. Clarke, M. L. et al. The electron-poor phosphines $P\{C_6H_3(CF_3)_2\text{-}3,5\}_3$ and $P(C_6F_5)_3$ do not mimic phosphites as ligands for hydroformylation. A comparison of the coordination chemistry of $P\{C_6H_3(CF_3)_2\text{-}3,5\}_3$ and $P(C_6F_5)_3$ and the unexpectedly low hydroformylation activity of their rhodium complexes. *Dalton Trans.*, 1294-1300 (2005).
2. Qian, H., Han, X. & Widenhoefer, R. A. Platinum-catalyzed intramolecular hydroalkoxylation of γ- and δ-hydroxy olefins to form cyclic ethers. *J. Am. Chem. Soc.* **126**, 9536-9537 (2004).
3. Bloome, K. S. & Alexanian, E. J. Palladium-catalyzed carbonylative heck-type reactions of alkyl iodides. *J. Am. Chem. Soc.* **132**, 12823-12825 (2010).
4. Fujita, S., Abe, M., Shibuya, M. & Yamamoto, Y. Intramolecular hydroalkoxylation of unactivated alkenes using silane–iodine catalytic system. *Org. Lett.* **17**, 3822-3825 (2015).
5. Hartung, J. et al. (Schiff-base)vanadium(V) complex-catalyzed oxidations of substituted bis(homoallylic) alcohols-stereoselective synthesis of functionalized tetrahydrofurans. *Eur. J. Org. Chem.* 2388-2408 (2003).
6. Hoang, G. T., Walsh, D. J., McGarry, K. A., Anderson, C. B. & Douglas, C. J. Development and mechanistic study of quinoline-directed acyl C–O bond activation and alkene oxyacylation reactions. *J. Org. Chem.* **82**, 2972-2983 (2017).
7. Sparrow, K. J., Carley, S., Sohnel, T., Barker, D. & Brimble, M. A. Studies towards development of asymmetric double-Mannich reactions of chiral 2-oxocyclohexanecarboxylate derivatives with bis(aminol)ethers. *Tetrahedron* **71**, 2210-2221 (2015).
8. Chen, X., Merrett, J. T. & Chan, P. W. H. Gold-catalyzed formal [4 + 2] cycloaddition of 5-(ethynylamino)pent-2-yn-1-yl esters to 1,2,3,5-tetrahydrobenzo[*g*]quinolines. *Org. Lett.* **20**, 1542-1545 (2018).
9. Mihara, K., Okada, I., Chiba, K. & Kitano, Y. Facile synthesis of *N*-substituted amides from alkenes and amides by a brønsted acid mediated electrophilic addition reaction. *Synthesis* **46**, 1455-1462 (2014).
10. White, D. R., Hinds, E. M., Bornowski, E. C. & Wolfe, J. P. Pd-catalyzed alkene difunctionalization reactions of malonate nucleophiles: synthesis of substituted cyclopentanes via alkene aryl-alkylation and akenyl-alkylation. *Org. Lett.* **21**, 3813-3816 (2019).
11. Ferrand, L. et al. Niobium-catalyzed intramolecular addition of O–H and N–H bonds to alkenes: a tool for hydrofunctionalization. *Org. Lett.* **19**, 2062-2065 (2017).
12. Marion, F., Coulomb, J., Courillon, C., Fensterbank, L. & Malacria, M. Platinum dichloride-catalyzed cycloisomerization of ene-ynamides. *Org. Lett.* **6**, 1509-1511 (2004).
13. Kuznetsov, A., Makarov, A., Rubtsov, A. E., Butin, A. V. & Gevorgyan, V. Brönsted acid-catalyzed one-pot synthesis of indoles from *o*-aminobenzyl alcohols and furans. *J. Org. Chem.* **78**, 12144-12153 (2013).
14. Kapat, A., König, A., Montermini, F. & Renaud, P. A radical procedure for the anti-markovnikov hydroazidation of alkenes. *J. Am. Chem. Soc.* **133**, 13890-13893 (2011).
15. Ohmiya, H., Tsuji, T., Yorimitsu, H. & Oshima, K. Cobalt-catalyzed cross-coupling reactions of alkyl halides with allylic and benzylic grignard reagents and their application to tandem radical cyclization/cross-coupling reactions. *Chem. Eur. J.* **10**, 5640-5648 (2004).
16. Green, S. A., Vásquez-Céspedes, S. & Shenvi, R. A. Iron–nickel dual-catalysis: a new engine for olefin

functionalization and the formation of quaternary centers. *J. Am. Chem. Soc.* **140**, 11317-11324 (2018).

17. Ye, L., Lo, K.-Y., Gu, Q. & Yang, D. Pd-catalyzed intramolecular aminoalkylation of unactivated alkenes: access to diverse *N*-heterocycles. *Org. Lett.* **19**, 308-311 (2017).
18. Zhu, X. & Chiba, S. TEMPO-mediated allylic C–H amination with hydrazones. *Org. Biomol. Chem.* **12**, 4567-4570 (2014).
19. Driver, T. G. & Woerpel, K. A. Mechanism of silver-mediated di-*tert*-butylsilylene transfer from a silacyclopropane to an alkene. *J. Am. Chem. Soc.* **126**, 9993-10002 (2004).
20. Lettko, L., Wood, J. S. & Rausch, M. D. Synthesis of (phosphine)silver(I) trifluoromethanesulfonate complexes and the molecular structure of di-μ-trifluoromethylsulfonate-(tetrakis-triphenylphosphine)disilver(I). I*norganica Chimica Acta* **308**, 37-44 (2000).
21. Clark, T. J. et al. Rhodium-catalyzed dehydrocoupling of fluorinated phosphine-borane adducts: synthesis, characterization, and properties of cyclic and polymeric phosphinoboranes with electron-withdrawing substituents at phosphorus. *Chem. Eur. J.* **11**, 4526-4534 (2005).
22. Grotjahn, D. B. et al. Finding the proton in a key intermediate of anti-markovnikov alkyne hydration by a bifunctional catalyst. *J. Am. Chem. Soc.* **130**, 10860-10861 (2008).
23. Watson, M. P. & Jacobsen, E. N. Asymmetric intramolecular arylcyanation of unactivated olefins via C−CN bond activation. *J. Am. Chem. Soc.* **130**, 12594-12595 (2008).
24. Fustero, S. et al. Organocatalytic approach to benzofused nitrogen-containing heterocycles: enantioselective total synthesis of (+)-angustureine. *Chem.* **-** *Eur. J.* **14**, 9868-9872 (2008).
25. Rosenfeld, D. C., Shekhar, S., Takemiya, A., Utsunomiya, M. & Hartwig, J. F. Hydroamination and hydroalkoxylation catalyzed by triflic acid. Parallels to reactions initiated with metal triflates. *Org. Lett.* **8**, 4179-4182 (2006).
26. Zeng, W. & Chemler, S. R. Copper(II)-catalyzed enantioselective intramolecular carboamination of alkenes. *J. Am. Chem. Soc.* **129**, 12948-12949 (2007).
27. Hazelden, I. R., Carmona, R. C., Langer, T., Pringle, P. G. & Bower, J. F. Pyrrolidines and piperidines by ligand-enabled aza-heck cyclizations and cascades of *N*-(pentafluorobenzoyloxy)carbamates. *Angew. Chem. Int. Ed.* **57**, 5124-5128 (**2018**).
28. Escudero, J., Bellosta, V. & Cossy, J. Rhodium-catalyzed cyclization of *O*,w-unsaturated alkoxyamines: formation of oxygen-containing heterocycles. *Angew. Chem. Int. Ed.* **57**, 574-578 (2018).
29. Rösner, C. & Hennecke, U. Homohalocyclization: electrophilic bromine-induced cyclizations of cyclopropanes. *Org. Lett.* **17**, 3226-3229 (2015).

11. NMR Spectra

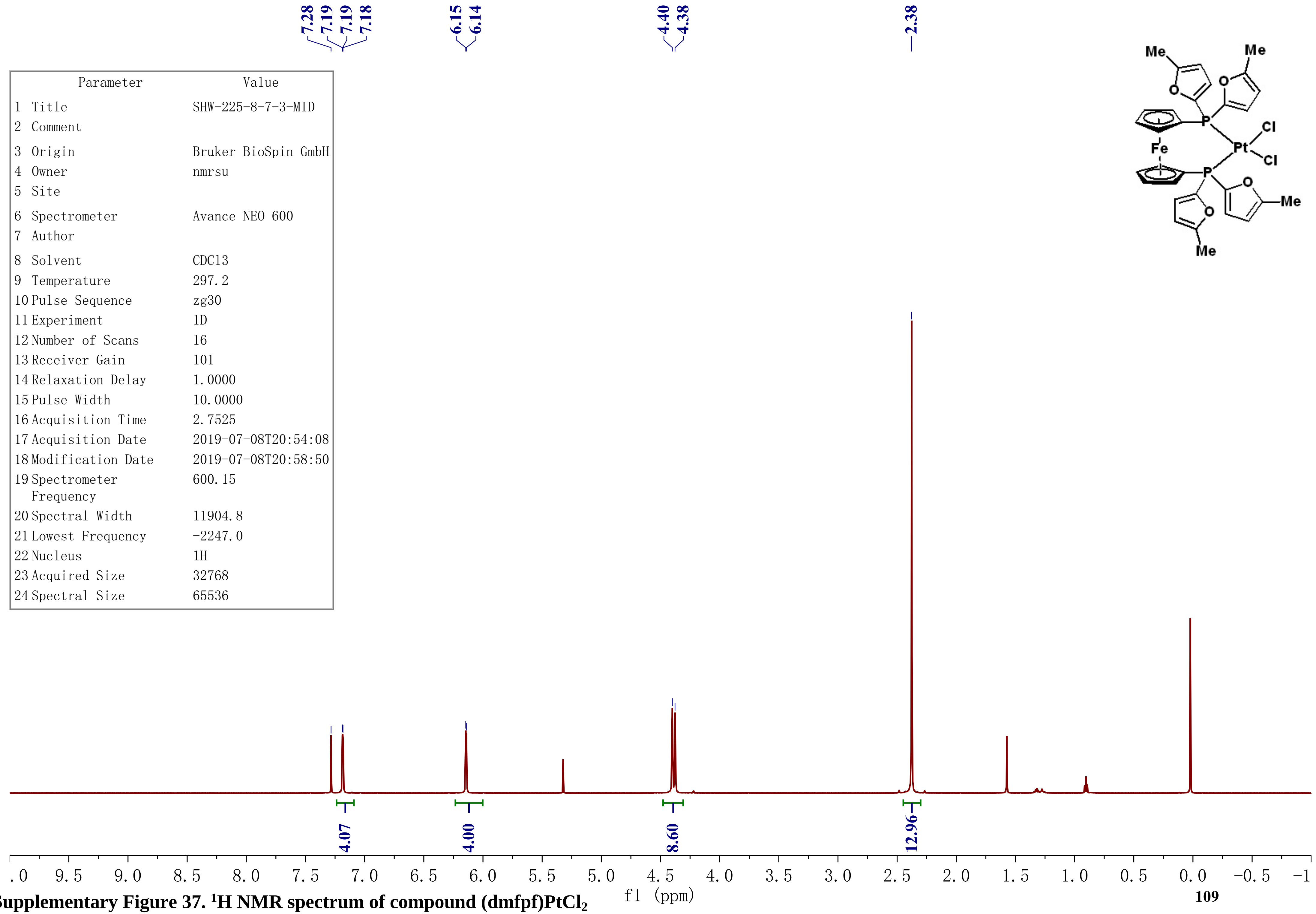

Supplementary Figure 37. ^{1}**H NMR spectrum of compound (dmfpf)PtCl$_2$**

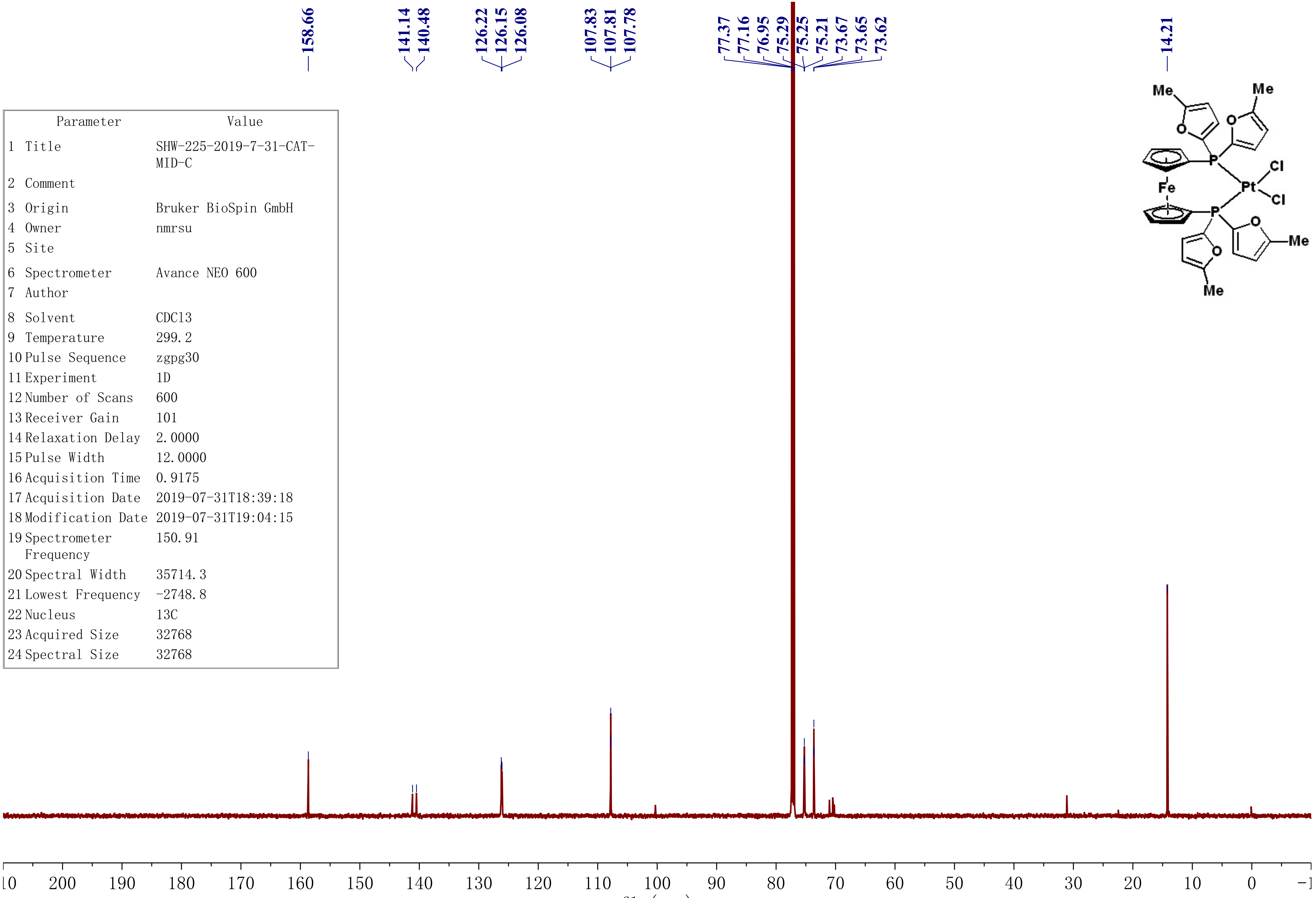

Parameter	Value
1 Title	SHW-225-2019-7-31-CAT-MID-C
2 Comment	
3 Origin	Bruker BioSpin GmbH
4 Owner	nmrsu
5 Site	
6 Spectrometer	Avance NEO 600
7 Author	
8 Solvent	CDCl3
9 Temperature	299.2
10 Pulse Sequence	zgpg30
11 Experiment	1D
12 Number of Scans	600
13 Receiver Gain	101
14 Relaxation Delay	2.0000
15 Pulse Width	12.0000
16 Acquisition Time	0.9175
17 Acquisition Date	2019-07-31T18:39:18
18 Modification Date	2019-07-31T19:04:15
19 Spectrometer Frequency	150.91
20 Spectral Width	35714.3
21 Lowest Frequency	-2748.8
22 Nucleus	13C
23 Acquired Size	32768
24 Spectral Size	32768

Supplementary Figure 38. ^{13}C NMR spectrum of compound (dmfpf)$PtCl_2$

Parameter		Value
1	Title	SHW-225-8-7-3-MID
2	Comment	
3	Origin	Bruker BioSpin GmbH
4	Owner	nmrsu
5	Site	
6	Spectrometer	Avance NEO 600
7	Author	
8	Solvent	CDCl3
9	Temperature	298.2
10	Pulse Sequence	zgpg30
11	Experiment	1D
12	Number of Scans	38
13	Receiver Gain	101
14	Relaxation Delay	2.0000
15	Pulse Width	12.0000
16	Acquisition Time	0.3408
17	Acquisition Date	2019-07-08T20:56:25
18	Modification Date	2019-07-08T20:58:50
19	Spectrometer Frequency	242.95
20	Spectral Width	96153.8
21	Lowest Frequency	-60224.2
22	Nucleus	31P
23	Acquired Size	32768
24	Spectral Size	32768

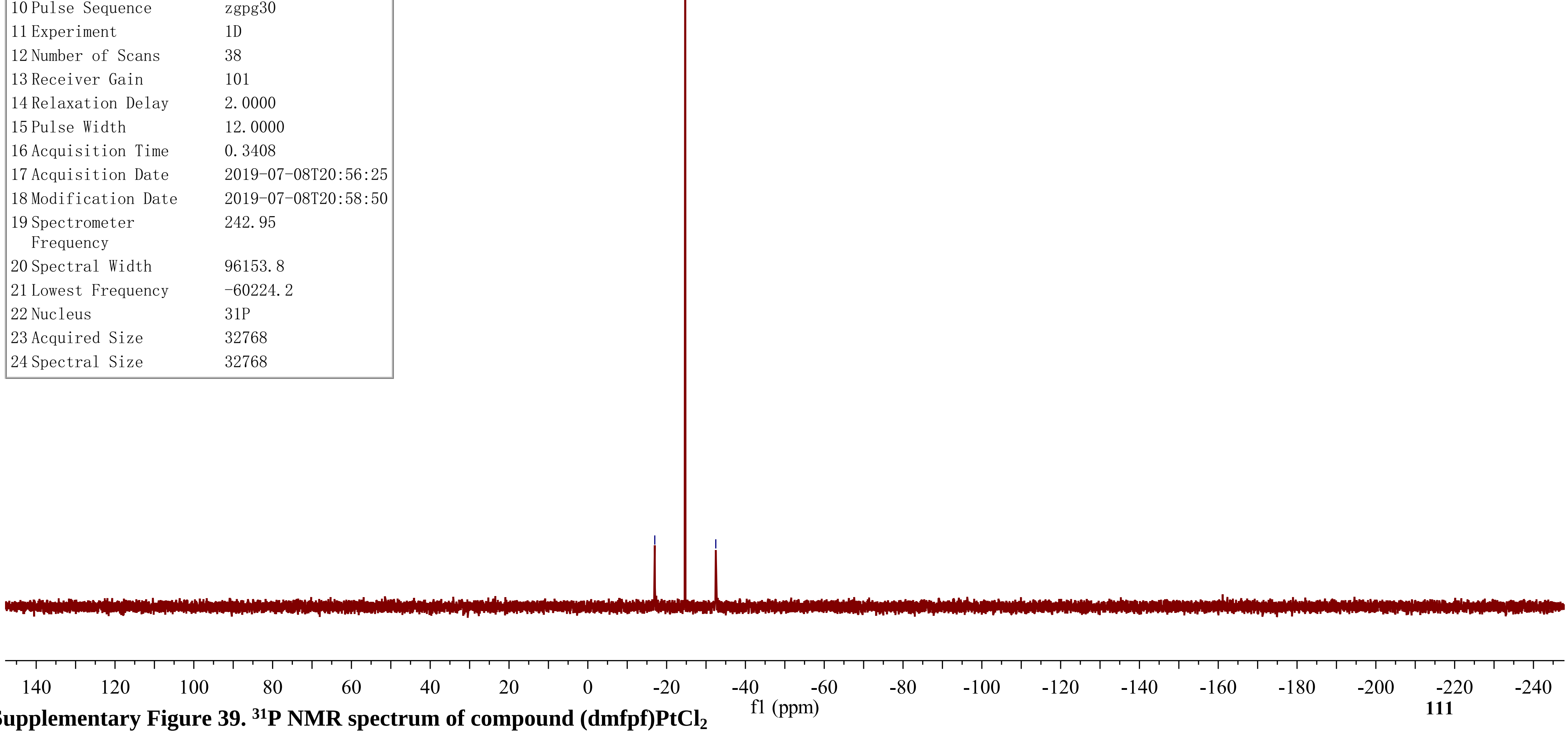

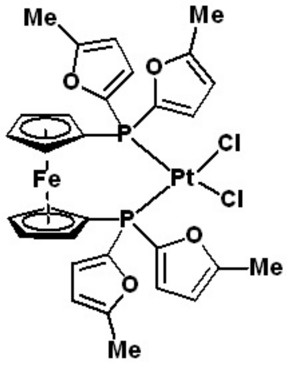

Supplementary Figure 39. ^{31}P NMR spectrum of compound (dmfpf)$PtCl_2$

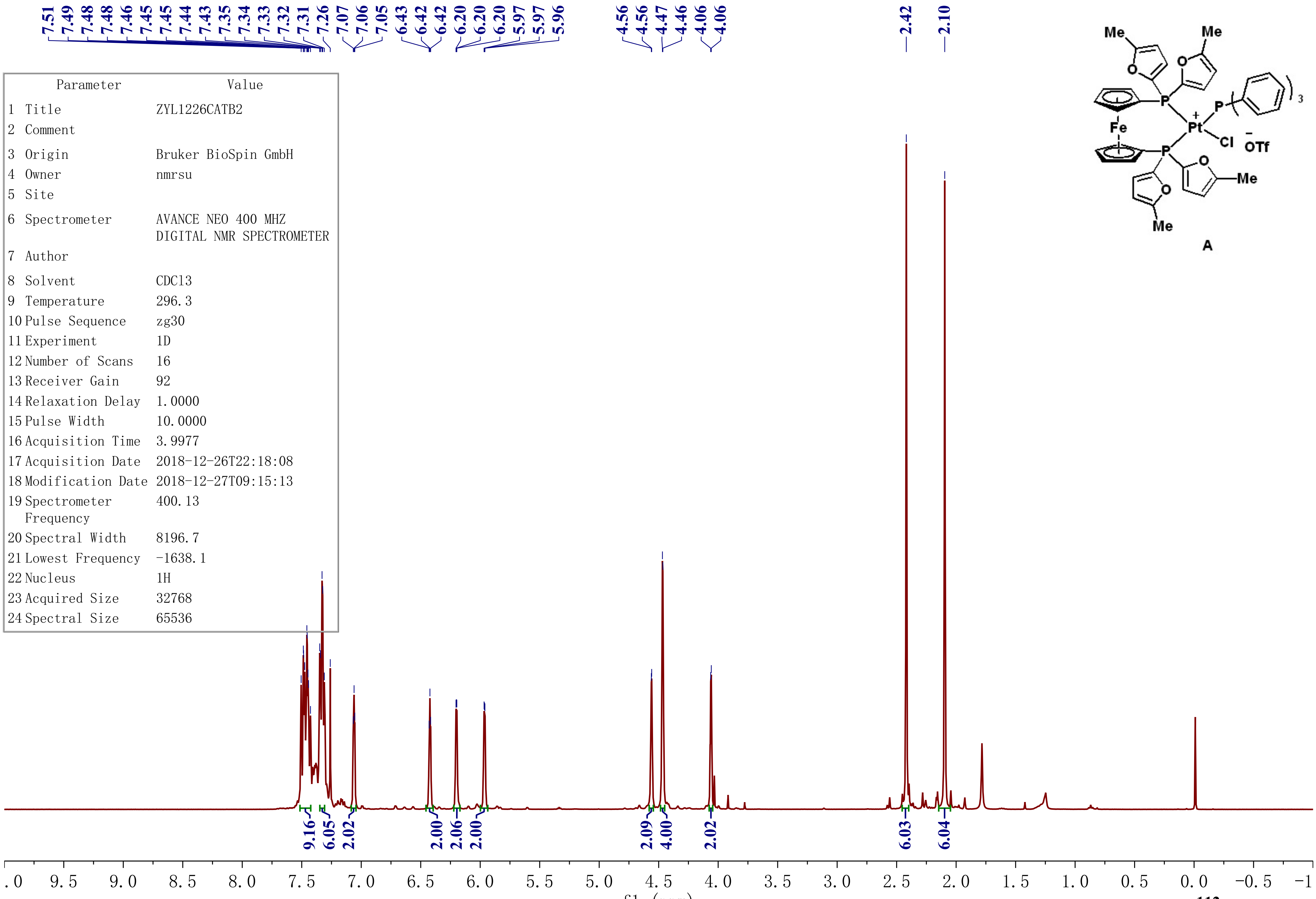

Supplementary Figure 40. ^{1}**H NMR spectrum of compound A**

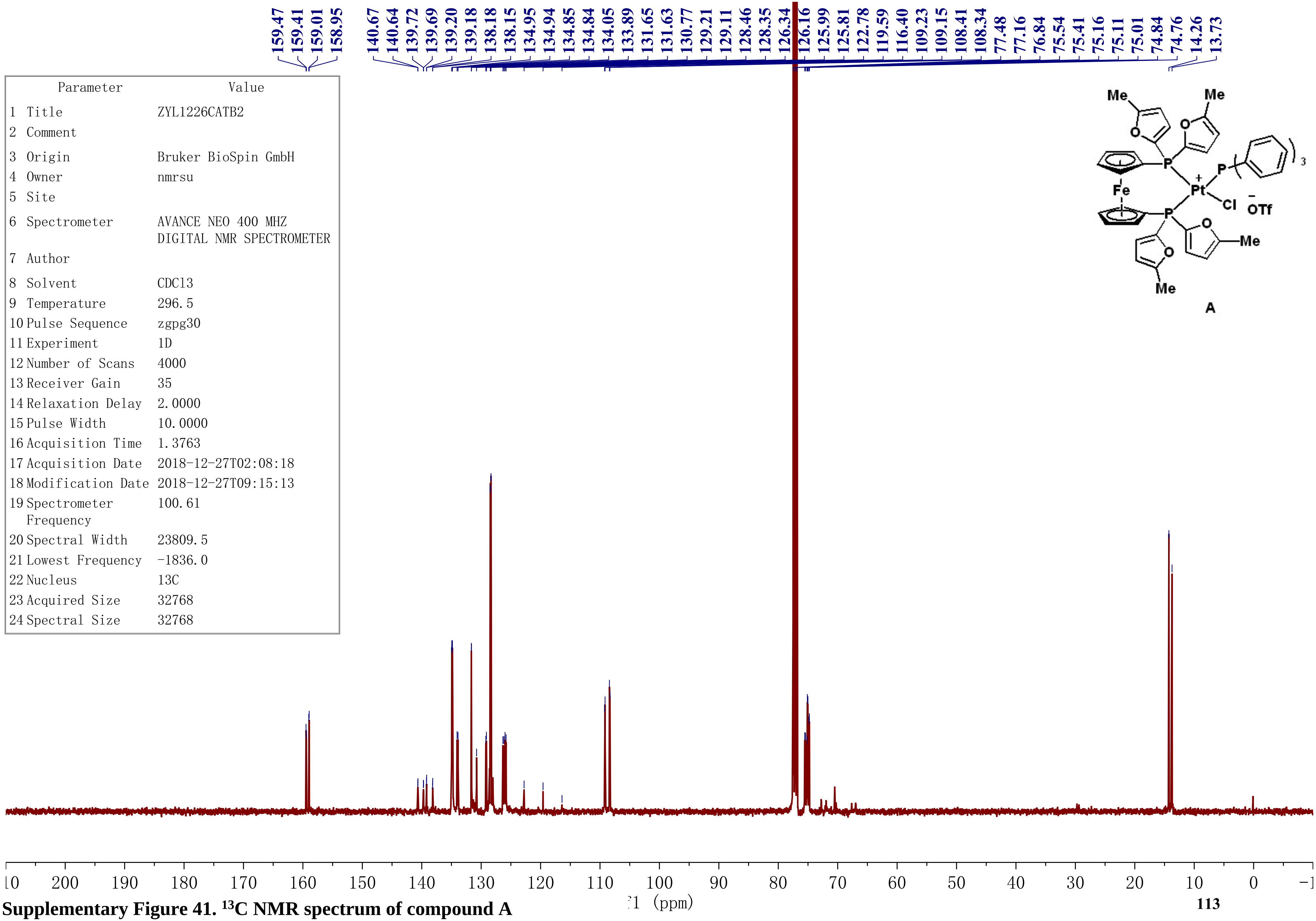

Parameter		Value
1	Title	ZYL1226CATB2
2	Comment	
3	Origin	Bruker BioSpin GmbH
4	Owner	nmrsu
5	Site	
6	Spectrometer	AVANCE NEO 400 MHZ DIGITAL NMR SPECTROMETER
7	Author	
8	Solvent	CDCl3
9	Temperature	296.5
10	Pulse Sequence	zgpg30
11	Experiment	1D
12	Number of Scans	4000
13	Receiver Gain	35
14	Relaxation Delay	2.0000
15	Pulse Width	10.0000
16	Acquisition Time	1.3763
17	Acquisition Date	2018-12-27T02:08:18
18	Modification Date	2018-12-27T09:15:13
19	Spectrometer Frequency	100.61
20	Spectral Width	23809.5
21	Lowest Frequency	-1836.0
22	Nucleus	13C
23	Acquired Size	32768
24	Spectral Size	32768

Supplementary Figure 41. ^{13}C NMR spectrum of compound A

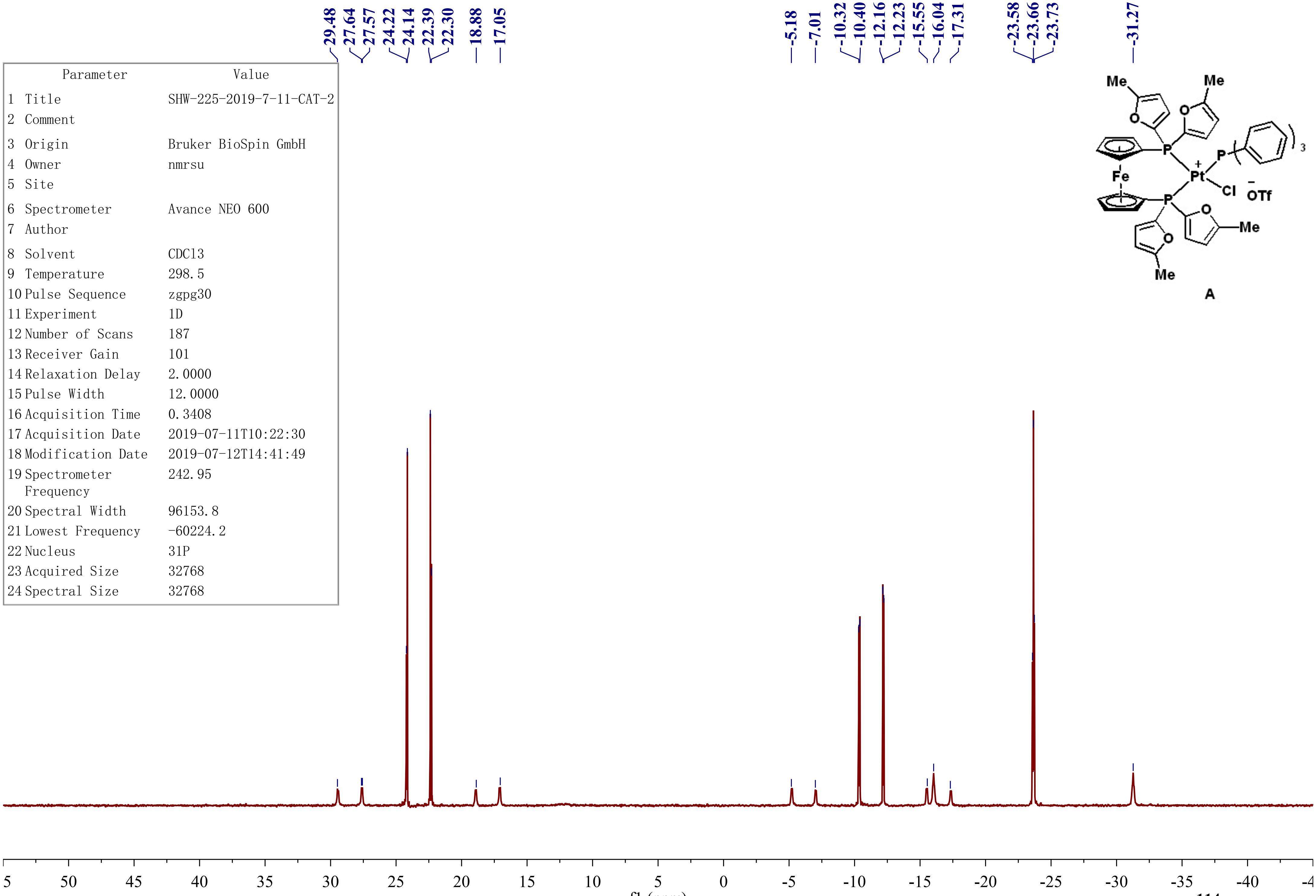

Parameter	Value
1 Title	SHW-225-2019-7-11-CAT-2
2 Comment	
3 Origin	Bruker BioSpin GmbH
4 Owner	nmrsu
5 Site	
6 Spectrometer	Avance NEO 600
7 Author	
8 Solvent	CDCl3
9 Temperature	298.5
10 Pulse Sequence	zgpg30
11 Experiment	1D
12 Number of Scans	187
13 Receiver Gain	101
14 Relaxation Delay	2.0000
15 Pulse Width	12.0000
16 Acquisition Time	0.3408
17 Acquisition Date	2019-07-11T10:22:30
18 Modification Date	2019-07-12T14:41:49
19 Spectrometer Frequency	242.95
20 Spectral Width	96153.8
21 Lowest Frequency	-60224.2
22 Nucleus	31P
23 Acquired Size	32768
24 Spectral Size	32768

Supplementary Figure 42. ^{31}P NMR spectrum of compound A

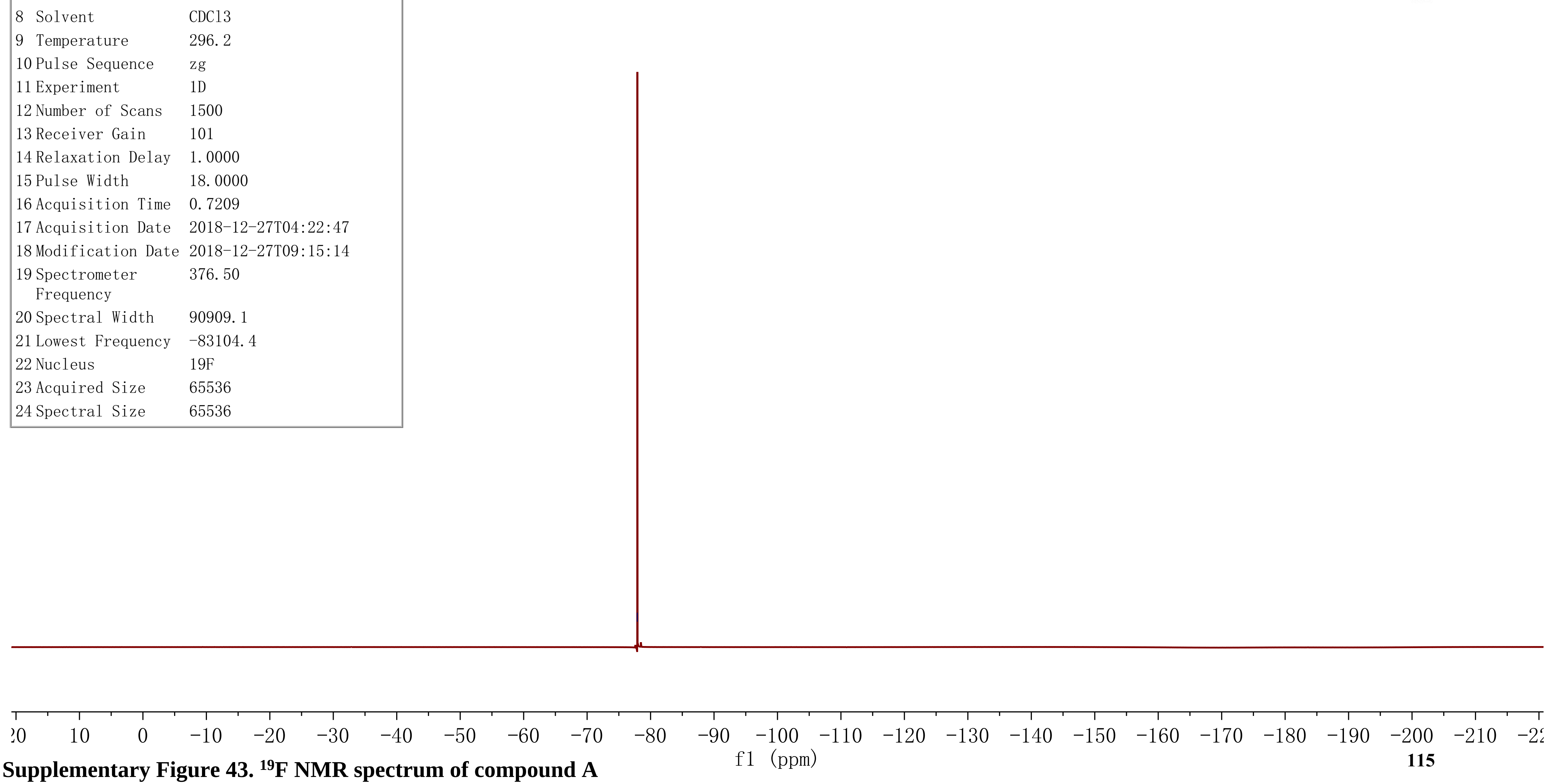

	Parameter	Value
1	Title	ZYL1226CATB2
2	Comment	
3	Origin	Bruker BioSpin GmbH
4	Owner	nmrsu
5	Site	
6	Spectrometer	AVANCE NEO 400 MHZ DIGITAL NMR SPECTROMETER
7	Author	
8	Solvent	CDCl3
9	Temperature	296.2
10	Pulse Sequence	zg
11	Experiment	1D
12	Number of Scans	1500
13	Receiver Gain	101
14	Relaxation Delay	1.0000
15	Pulse Width	18.0000
16	Acquisition Time	0.7209
17	Acquisition Date	2018-12-27T04:22:47
18	Modification Date	2018-12-27T09:15:14
19	Spectrometer Frequency	376.50
20	Spectral Width	90909.1
21	Lowest Frequency	-83104.4
22	Nucleus	19F
23	Acquired Size	65536
24	Spectral Size	65536

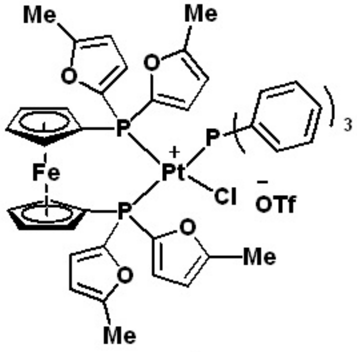

A

Supplementary Figure 43. ^{19}**F NMR spectrum of compound A**

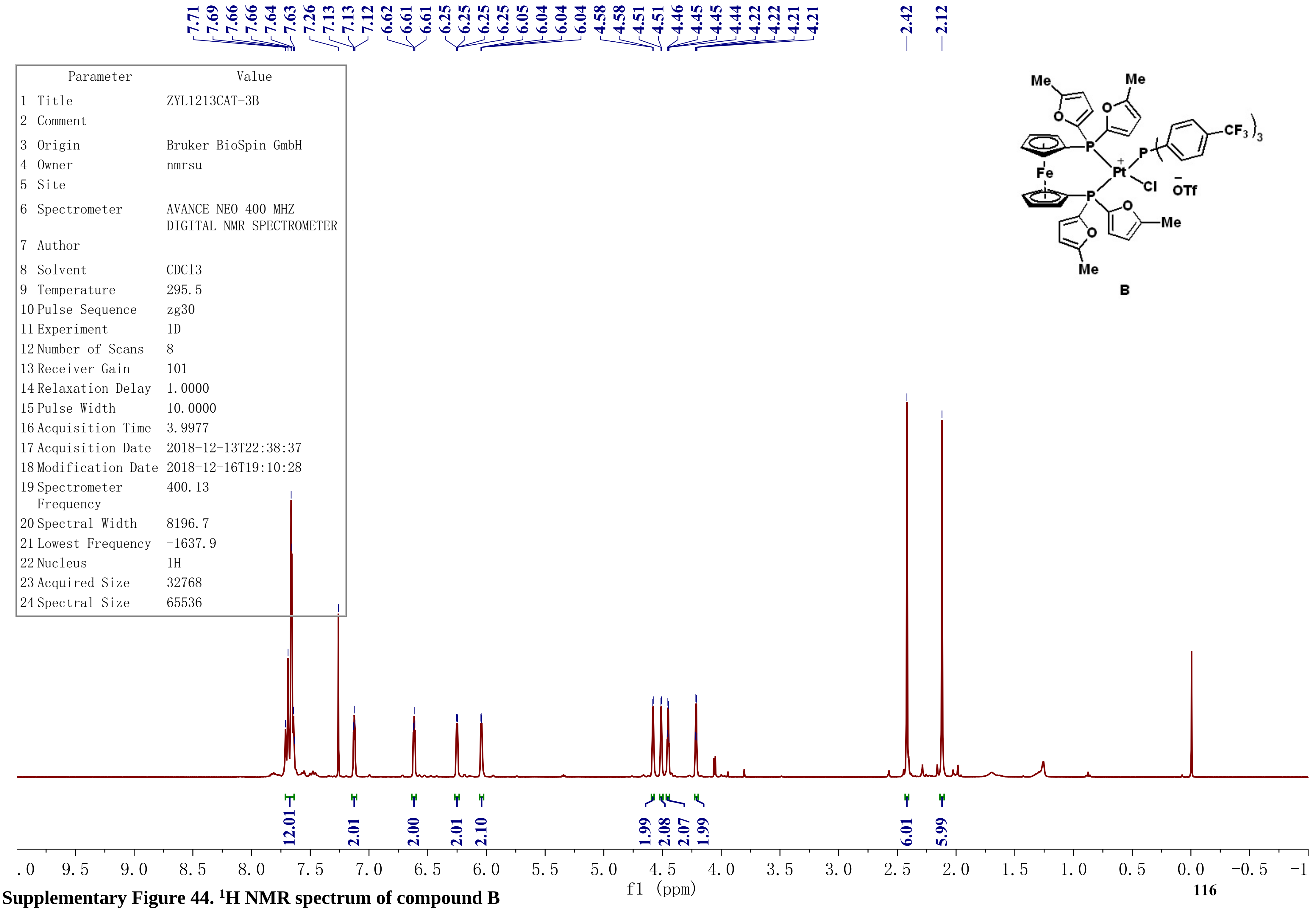

Supplementary Figure 44. ^{1}H NMR spectrum of compound B

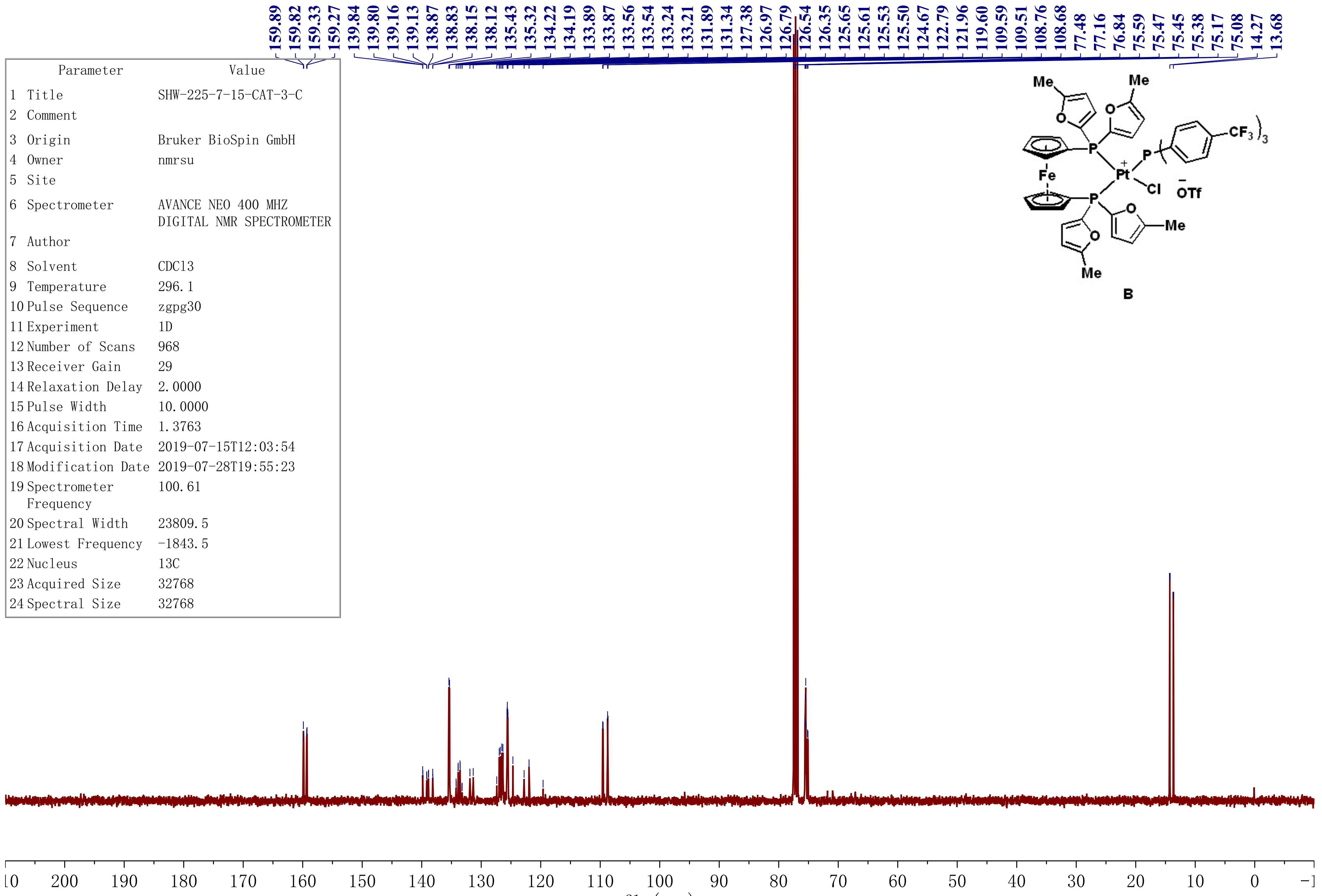

Parameter	Value
1 Title	SHW-225-7-15-CAT-3-C
2 Comment	
3 Origin	Bruker BioSpin GmbH
4 Owner	nmrsu
5 Site	
6 Spectrometer	AVANCE NEO 400 MHZ DIGITAL NMR SPECTROMETER
7 Author	
8 Solvent	CDCl3
9 Temperature	296.1
10 Pulse Sequence	zgpg30
11 Experiment	1D
12 Number of Scans	968
13 Receiver Gain	29
14 Relaxation Delay	2.0000
15 Pulse Width	10.0000
16 Acquisition Time	1.3763
17 Acquisition Date	2019-07-15T12:03:54
18 Modification Date	2019-07-28T19:55:23
19 Spectrometer Frequency	100.61
20 Spectral Width	23809.5
21 Lowest Frequency	-1843.5
22 Nucleus	13C
23 Acquired Size	32768
24 Spectral Size	32768

Supplementary Figure 45. ^{13}C NMR spectrum of compound B

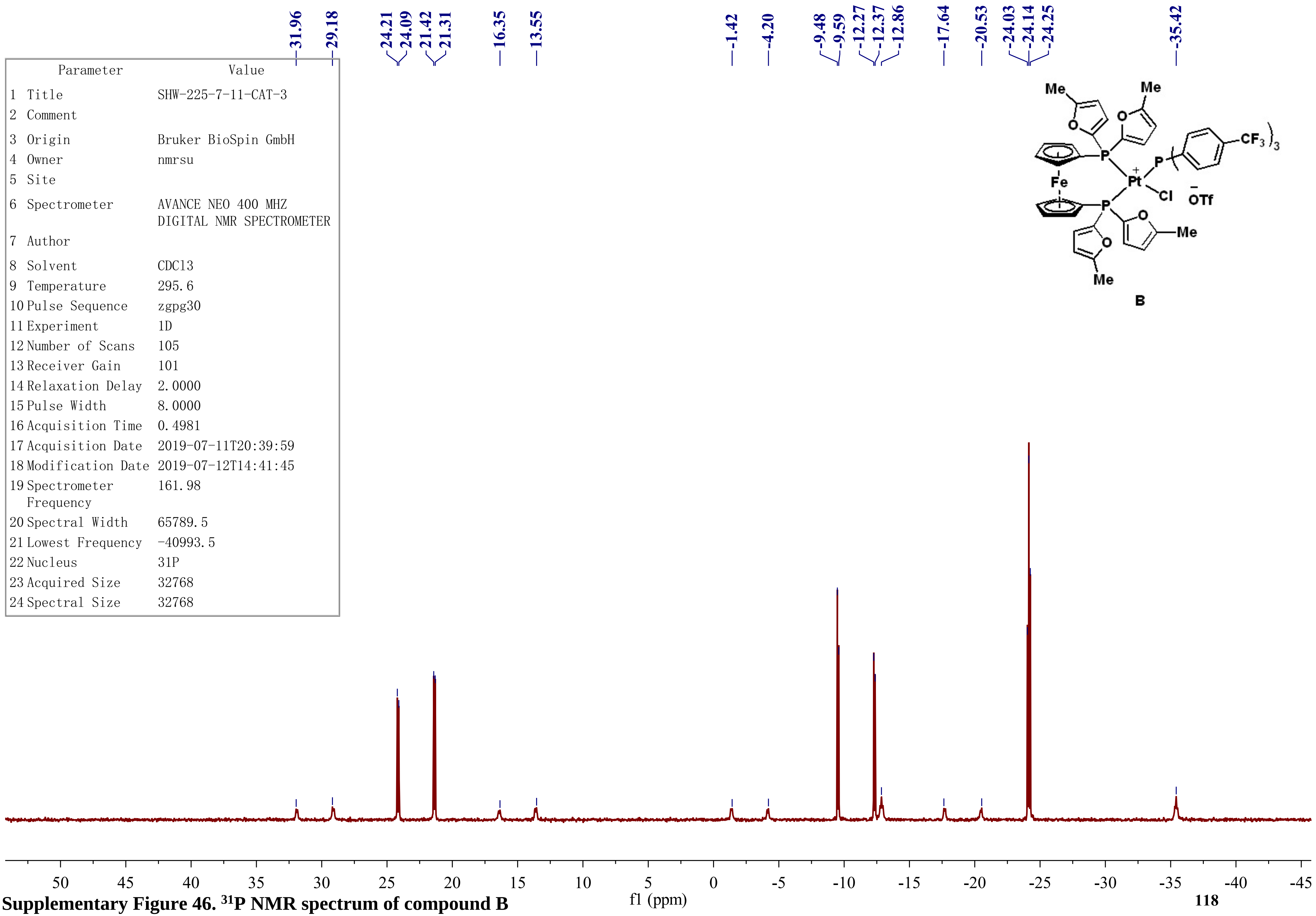

	Parameter	Value
1	Title	SHW-225-7-11-CAT-3
2	Comment	
3	Origin	Bruker BioSpin GmbH
4	Owner	nmrsu
5	Site	
6	Spectrometer	AVANCE NEO 400 MHZ DIGITAL NMR SPECTROMETER
7	Author	
8	Solvent	CDC13
9	Temperature	295.6
10	Pulse Sequence	zgpg30
11	Experiment	1D
12	Number of Scans	105
13	Receiver Gain	101
14	Relaxation Delay	2.0000
15	Pulse Width	8.0000
16	Acquisition Time	0.4981
17	Acquisition Date	2019-07-11T20:39:59
18	Modification Date	2019-07-12T14:41:45
19	Spectrometer Frequency	161.98
20	Spectral Width	65789.5
21	Lowest Frequency	-40993.5
22	Nucleus	31P
23	Acquired Size	32768
24	Spectral Size	32768

Supplementary Figure 46. ^{31}P **NMR spectrum of compound B**

Parameter		Value
1	Title	ZYL1213CAT-3B
2	Comment	
3	Origin	Bruker BioSpin GmbH
4	Owner	nmrsu
5	Site	
6	Spectrometer	AVANCE NEO 400 MHZ DIGITAL NMR SPECTROMETER
7	Author	
8	Solvent	CDCl3
9	Temperature	295.3
10	Pulse Sequence	zg
11	Experiment	1D
12	Number of Scans	1453
13	Receiver Gain	101
14	Relaxation Delay	1.0000
15	Pulse Width	18.0000
16	Acquisition Time	0.7209
17	Acquisition Date	2018-12-14T09:03:49
18	Modification Date	2018-12-16T19:10:29
19	Spectrometer Frequency	376.50
20	Spectral Width	90909.1
21	Lowest Frequency	-83104.4
22	Nucleus	19F
23	Acquired Size	65536
24	Spectral Size	65536

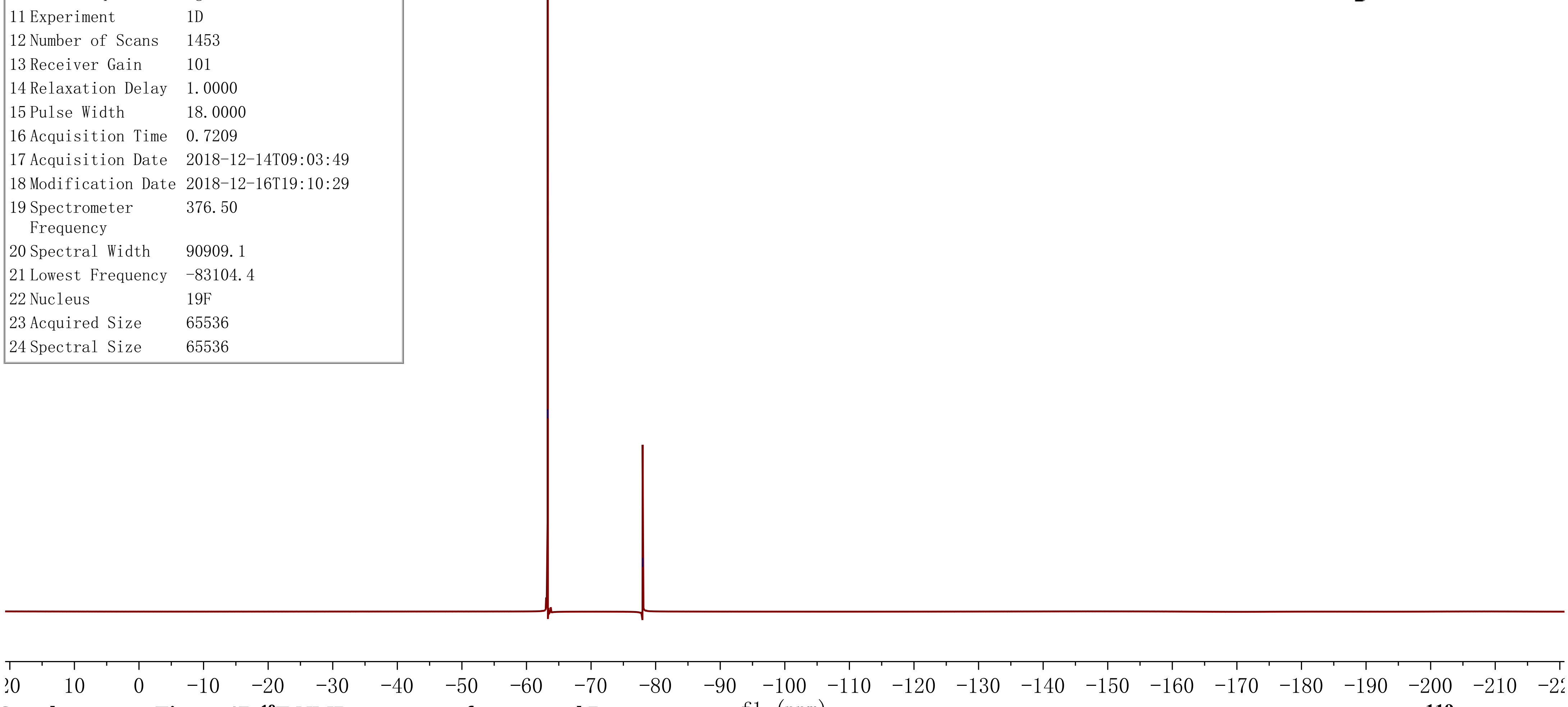

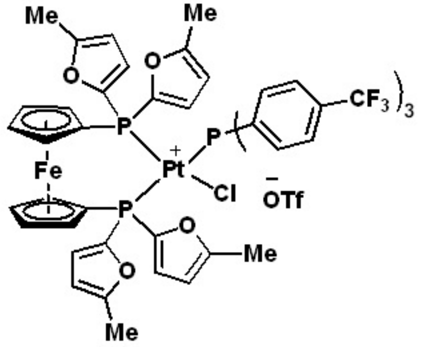

B

Supplementary Figure 47. ^{19}F NMR spectrum of compound B

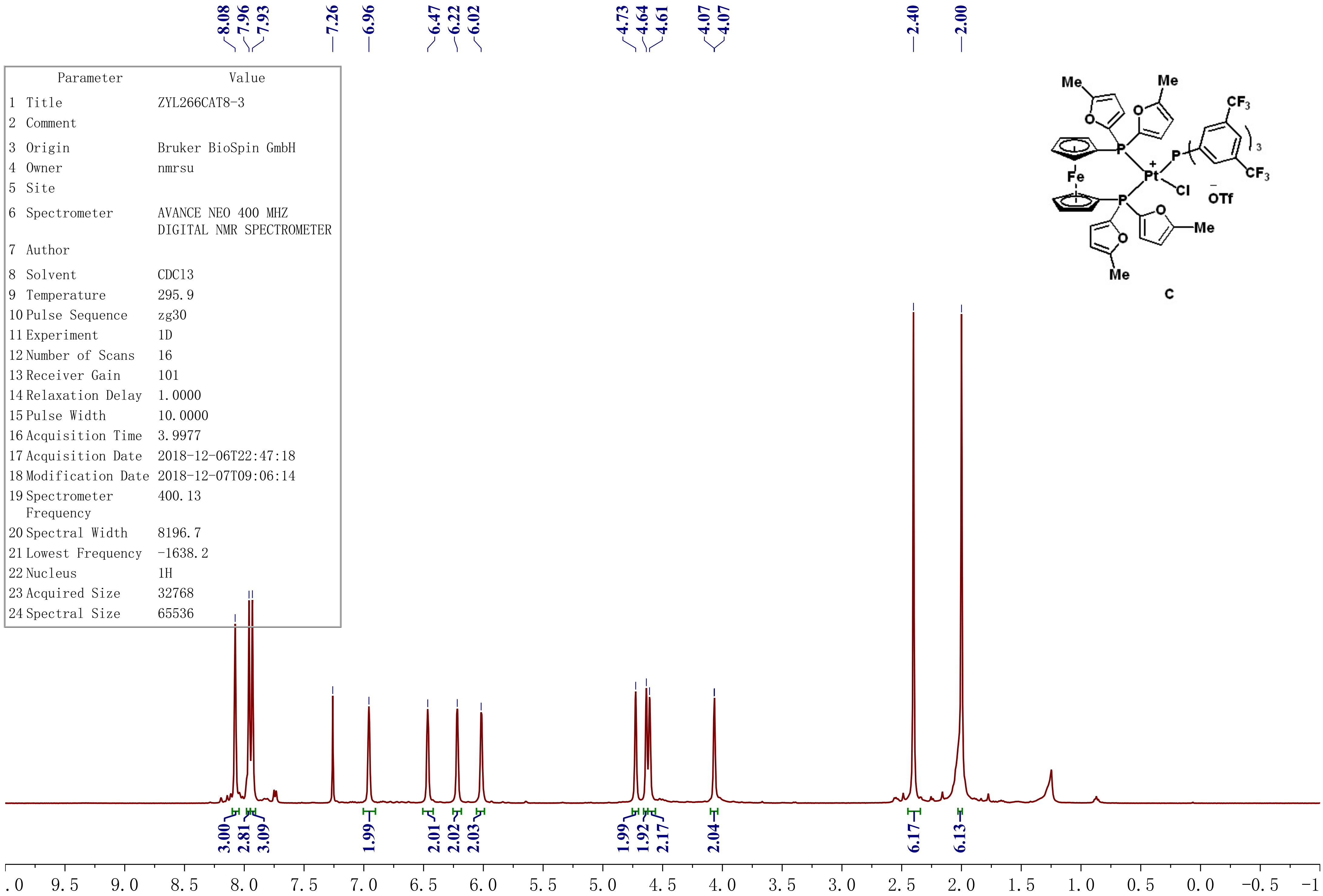

Supplementary Figure 48. ^{1}H **NMR spectrum of compound C**

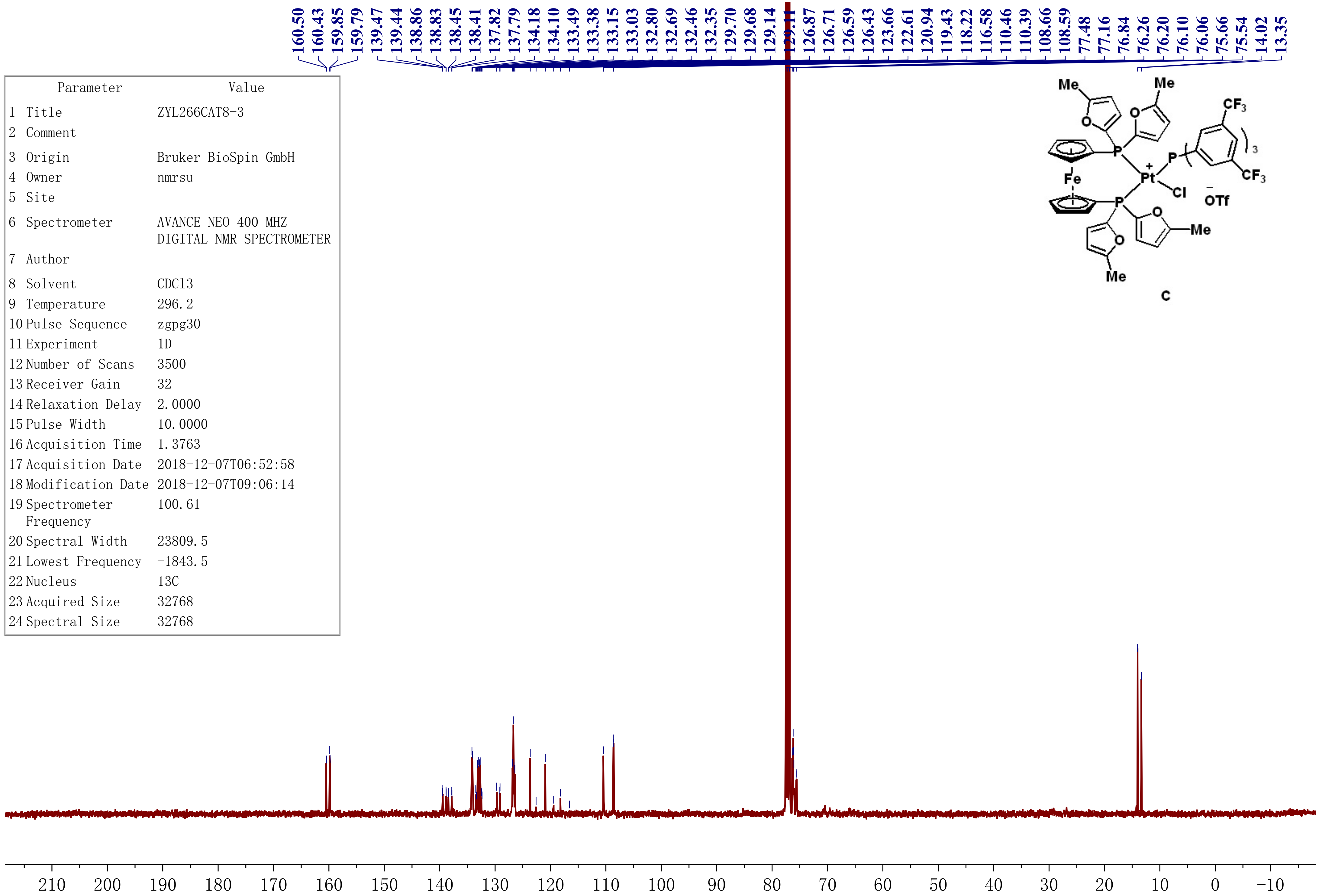

Supplementary Figure 49. ^{13}**C NMR spectrum of compound C**

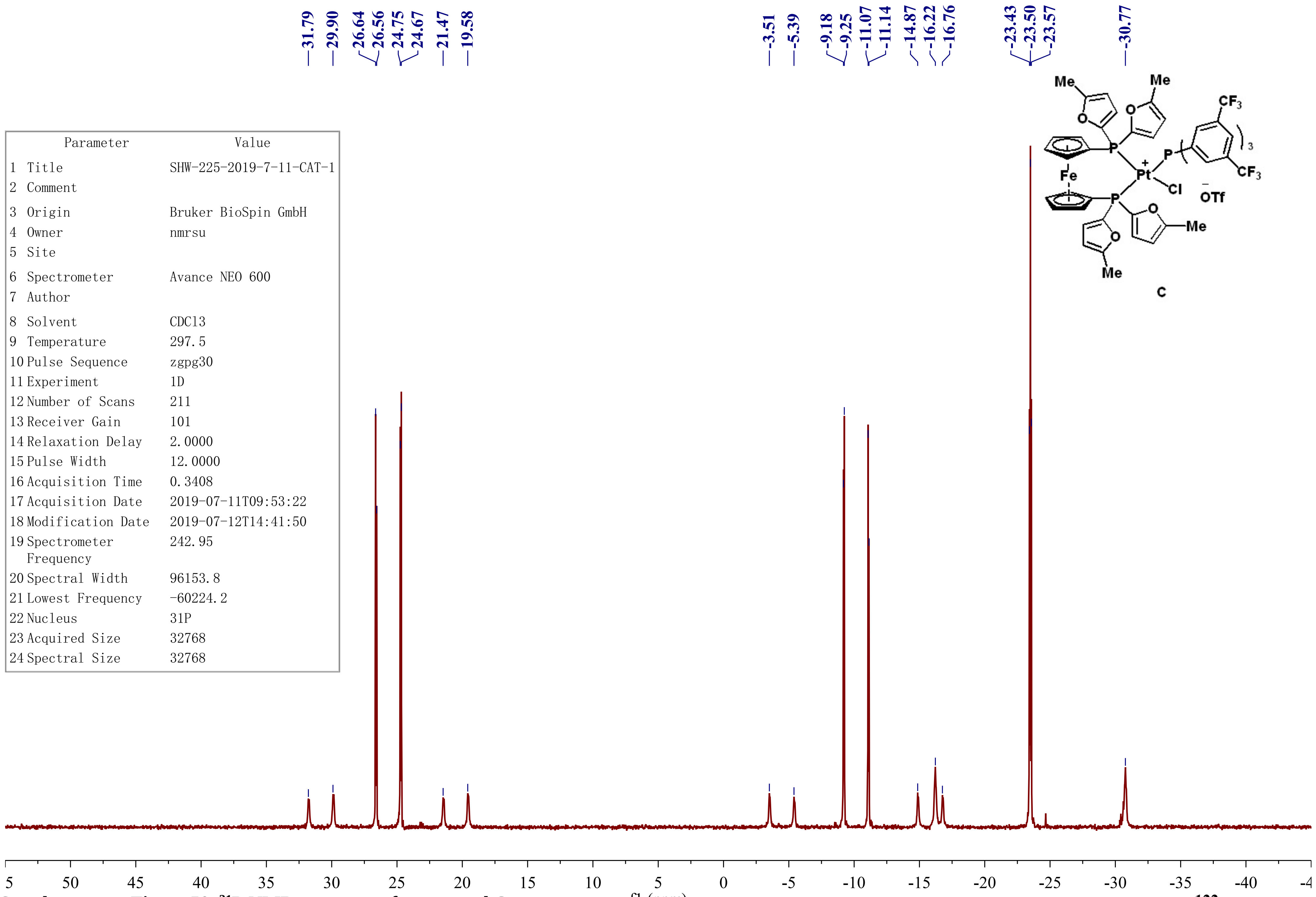

Parameter	Value
1 Title	SHW-225-2019-7-11-CAT-1
2 Comment	
3 Origin	Bruker BioSpin GmbH
4 Owner	nmrsu
5 Site	
6 Spectrometer	Avance NEO 600
7 Author	
8 Solvent	CDCl3
9 Temperature	297.5
10 Pulse Sequence	zgpg30
11 Experiment	1D
12 Number of Scans	211
13 Receiver Gain	101
14 Relaxation Delay	2.0000
15 Pulse Width	12.0000
16 Acquisition Time	0.3408
17 Acquisition Date	2019-07-11T09:53:22
18 Modification Date	2019-07-12T14:41:50
19 Spectrometer Frequency	242.95
20 Spectral Width	96153.8
21 Lowest Frequency	-60224.2
22 Nucleus	31P
23 Acquired Size	32768
24 Spectral Size	32768

Supplementary Figure 50. ^{31}P NMR spectrum of compound C

	Parameter	Value
1	Title	ZYL266CAT8-3
2	Comment	
3	Origin	Bruker BioSpin GmbH
4	Owner	nmrsu
5	Site	
6	Spectrometer	AVANCE NEO 400 MHZ DIGITAL NMR SPECTROMETER
7	Author	
8	Solvent	CDCl3
9	Temperature	295.6
10	Pulse Sequence	zg
11	Experiment	1D
12	Number of Scans	2400
13	Receiver Gain	101
14	Relaxation Delay	1.0000
15	Pulse Width	18.0000
16	Acquisition Time	0.7209
17	Acquisition Date	2018-12-07T08:03:52
18	Modification Date	2018-12-07T09:06:15
19	Spectrometer Frequency	376.50
20	Spectral Width	90909.1
21	Lowest Frequency	-83104.4
22	Nucleus	19F
23	Acquired Size	65536
24	Spectral Size	65536

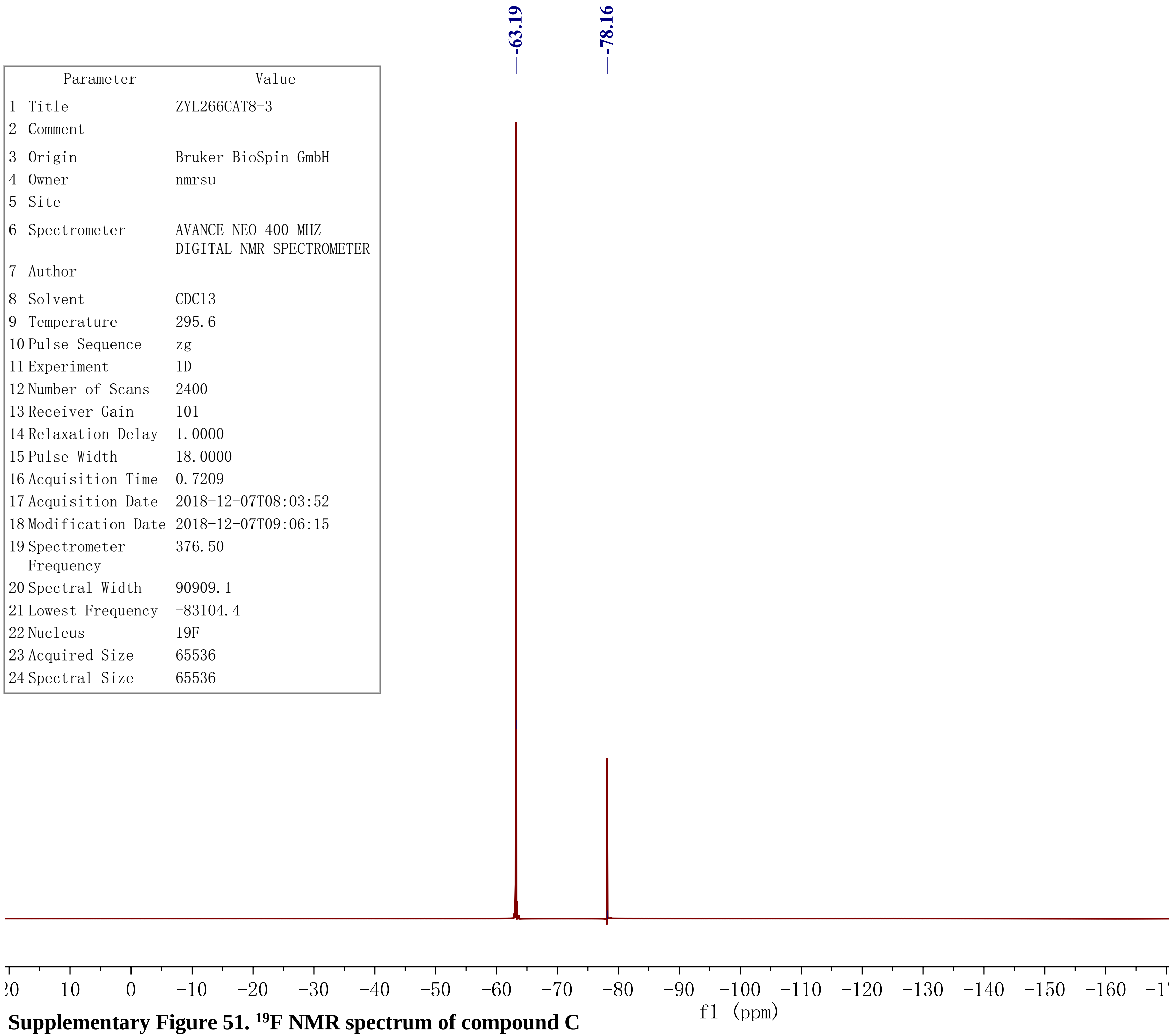

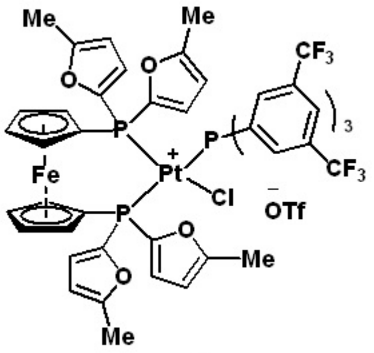

C

Supplementary Figure 51. ^{19}F NMR spectrum of compound C

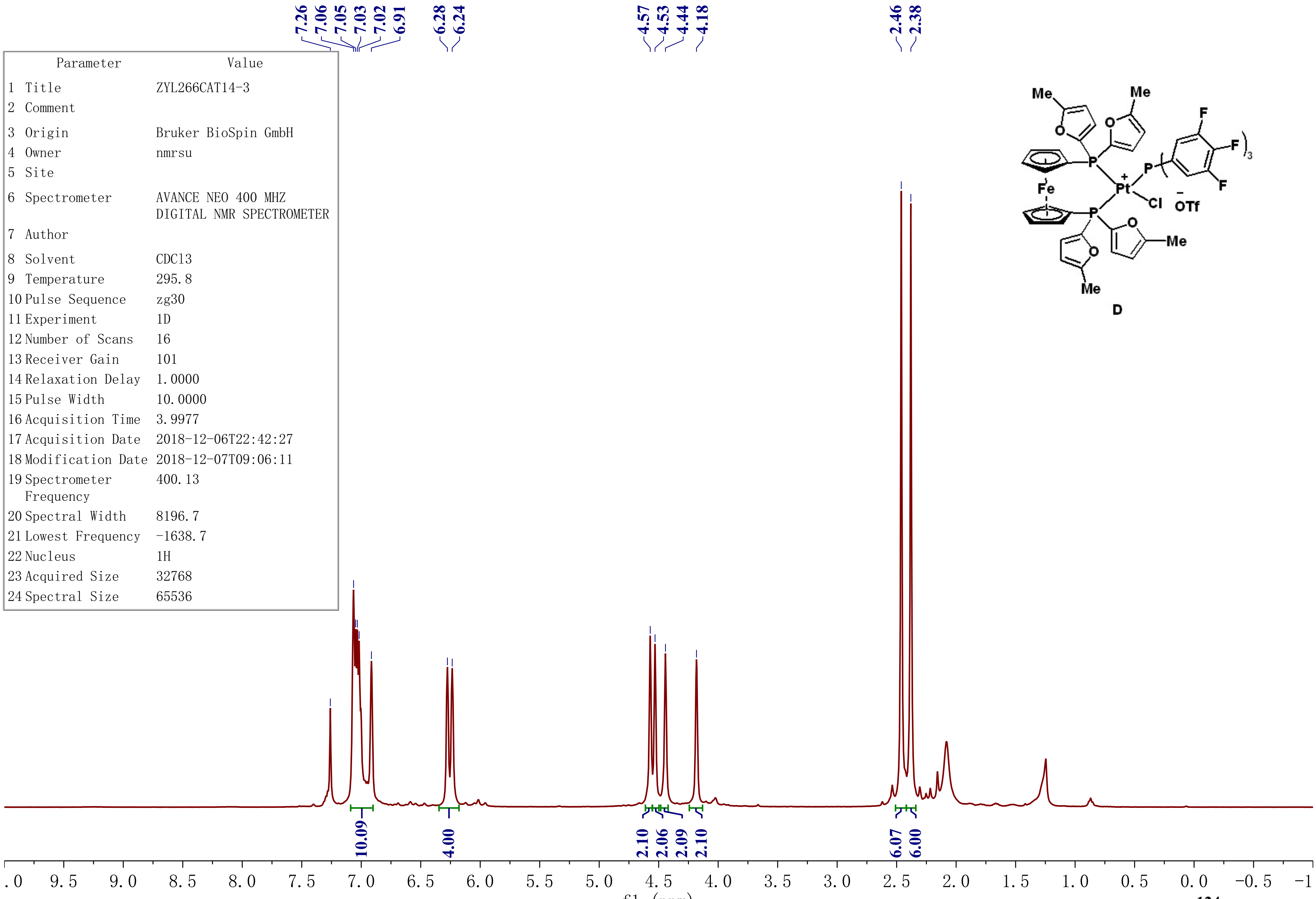

	Parameter	Value
1	Title	ZYL266CAT14-3
2	Comment	
3	Origin	Bruker BioSpin GmbH
4	Owner	nmrsu
5	Site	
6	Spectrometer	AVANCE NEO 400 MHZ DIGITAL NMR SPECTROMETER
7	Author	
8	Solvent	CDCl3
9	Temperature	295.8
10	Pulse Sequence	zg30
11	Experiment	1D
12	Number of Scans	16
13	Receiver Gain	101
14	Relaxation Delay	1.0000
15	Pulse Width	10.0000
16	Acquisition Time	3.9977
17	Acquisition Date	2018-12-06T22:42:27
18	Modification Date	2018-12-07T09:06:11
19	Spectrometer Frequency	400.13
20	Spectral Width	8196.7
21	Lowest Frequency	-1638.7
22	Nucleus	1H
23	Acquired Size	32768
24	Spectral Size	65536

Supplementary Figure 52. ^{1}H NMR spectrum of compound D

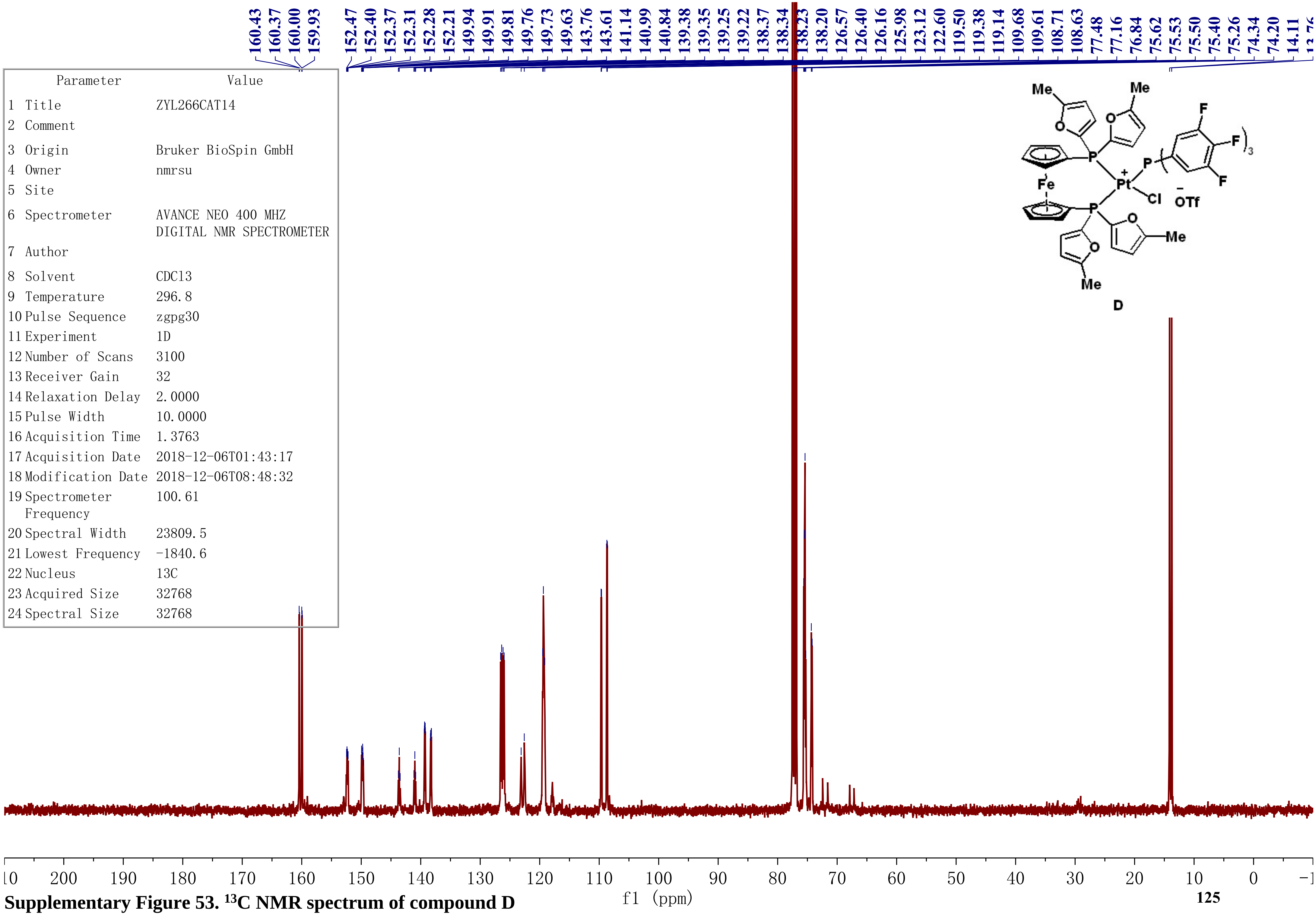

Supplementary Figure 53. ^{13}C **NMR spectrum of compound D**

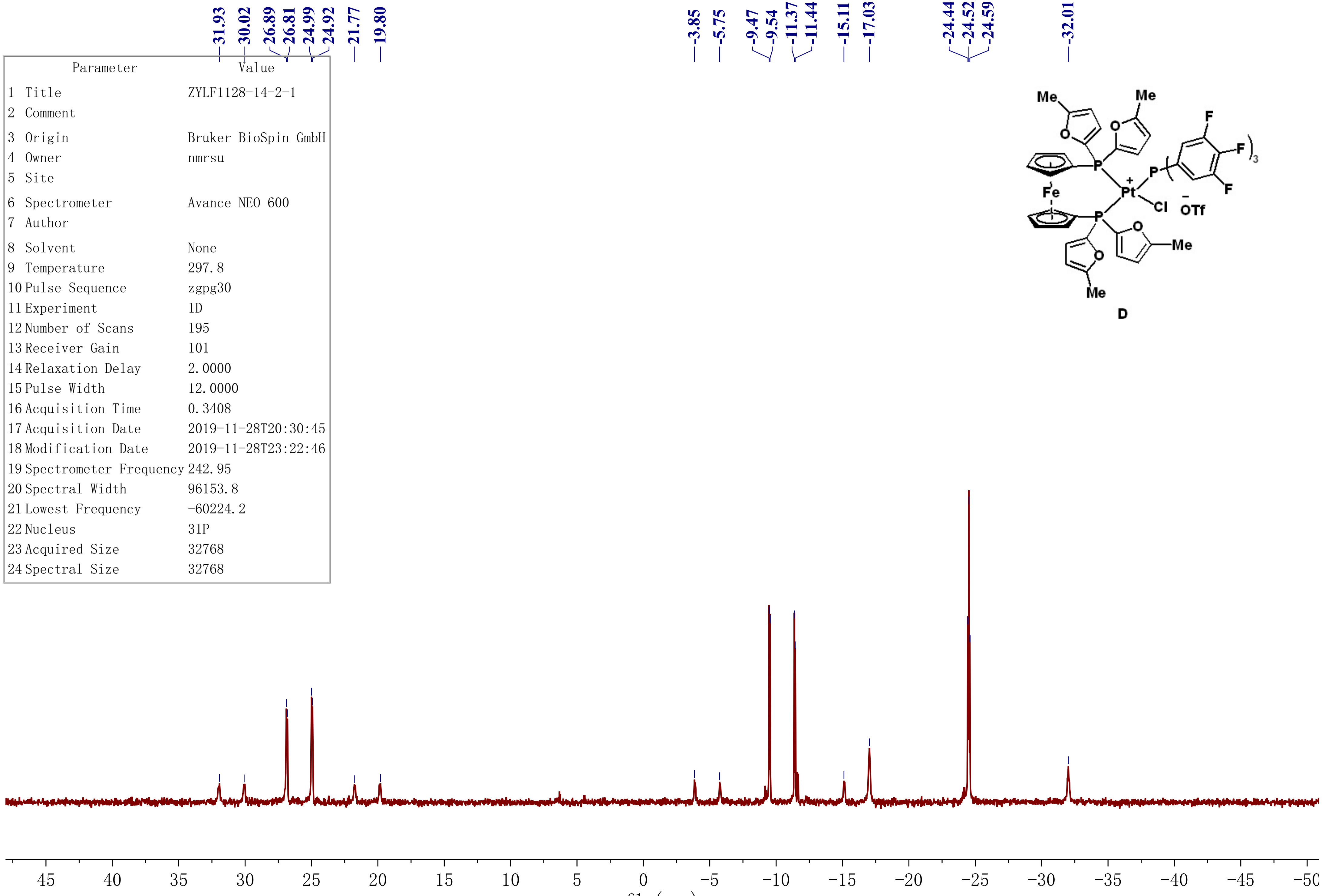

Supplementary Figure 54. ^{31}**P NMR spectrum of compound D**

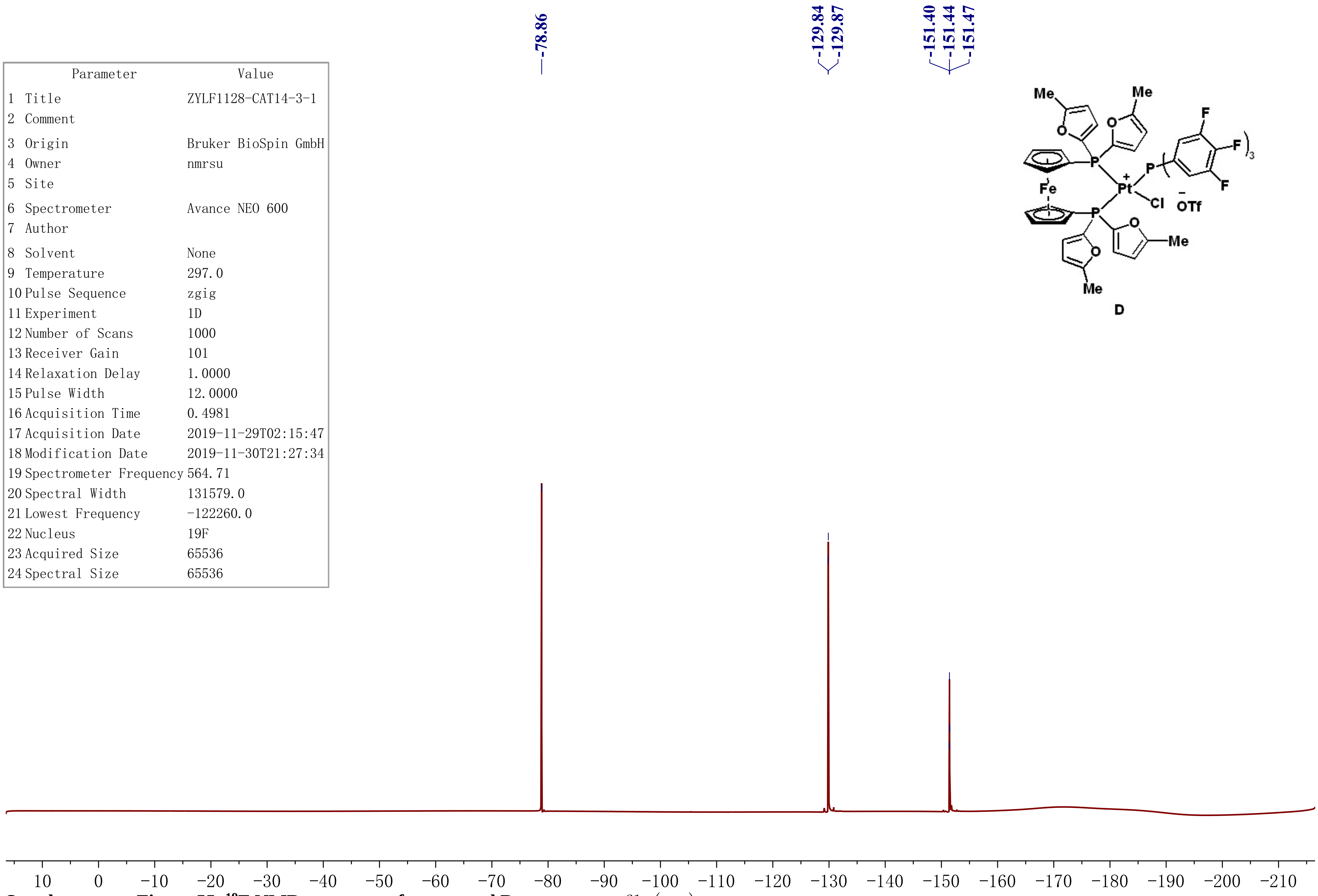

	Parameter	Value
1	Title	ZYLF1128-CAT14-3-1
2	Comment	
3	Origin	Bruker BioSpin GmbH
4	Owner	nmrsu
5	Site	
6	Spectrometer	Avance NEO 600
7	Author	
8	Solvent	None
9	Temperature	297.0
10	Pulse Sequence	zgig
11	Experiment	1D
12	Number of Scans	1000
13	Receiver Gain	101
14	Relaxation Delay	1.0000
15	Pulse Width	12.0000
16	Acquisition Time	0.4981
17	Acquisition Date	2019-11-29T02:15:47
18	Modification Date	2019-11-30T21:27:34
19	Spectrometer Frequency	564.71
20	Spectral Width	131579.0
21	Lowest Frequency	-122260.0
22	Nucleus	19F
23	Acquired Size	65536
24	Spectral Size	65536

Supplementary Figure 55. ^{19}F NMR spectrum of compound D

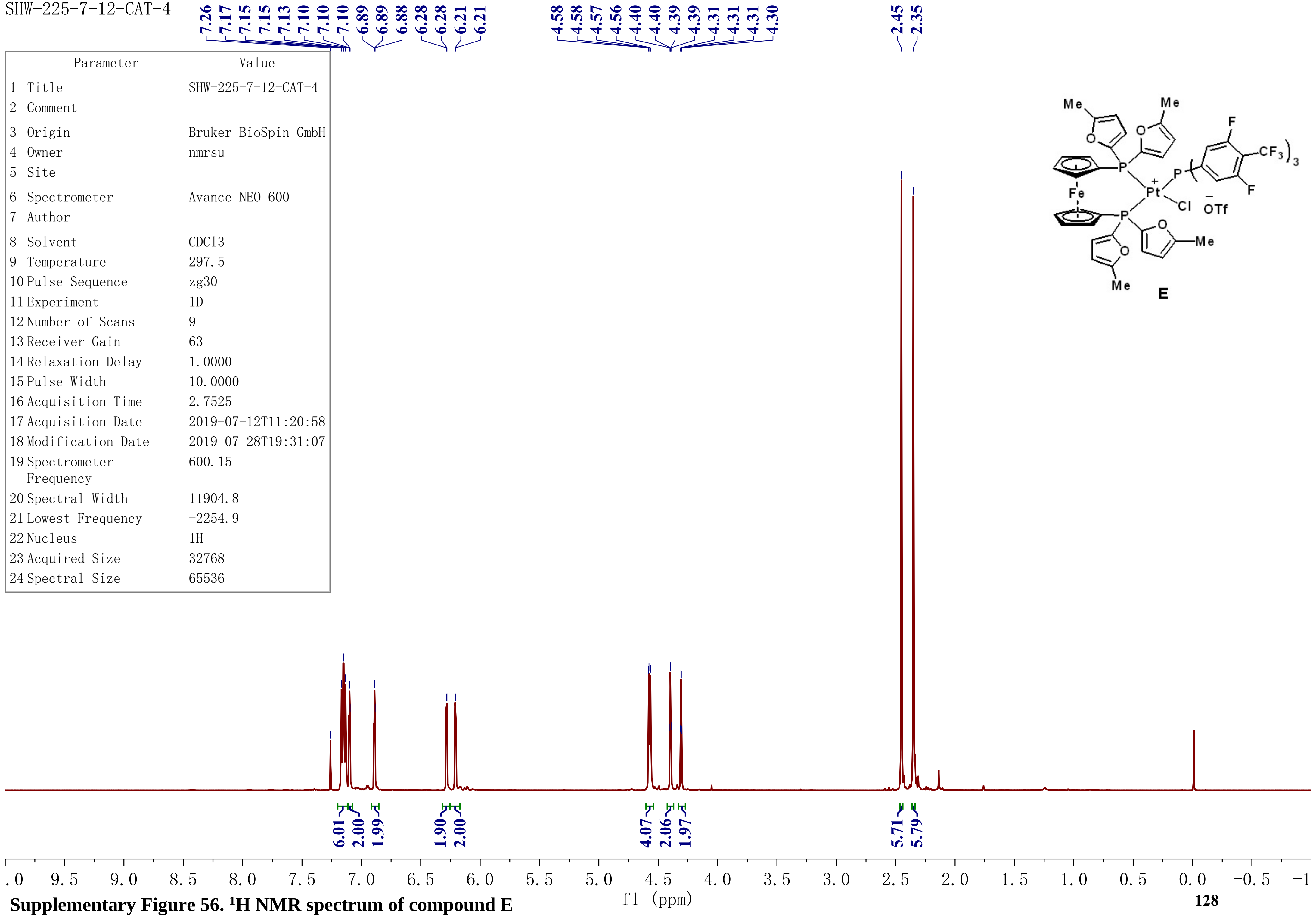

Parameter	Value
1 Title	SHW-225-7-12-CAT-4
2 Comment	
3 Origin	Bruker BioSpin GmbH
4 Owner	nmrsu
5 Site	
6 Spectrometer	Avance NEO 600
7 Author	
8 Solvent	CDCl3
9 Temperature	297.5
10 Pulse Sequence	zg30
11 Experiment	1D
12 Number of Scans	9
13 Receiver Gain	63
14 Relaxation Delay	1.0000
15 Pulse Width	10.0000
16 Acquisition Time	2.7525
17 Acquisition Date	2019-07-12T11:20:58
18 Modification Date	2019-07-28T19:31:07
19 Spectrometer Frequency	600.15
20 Spectral Width	11904.8
21 Lowest Frequency	-2254.9
22 Nucleus	1H
23 Acquired Size	32768
24 Spectral Size	65536

Supplementary Figure 56. ^{1}H NMR spectrum of compound E

Parameter		Value
1	Title	SHW-225-7-16-CAT-4-C
2	Comment	
3	Origin	Bruker BioSpin GmbH
4	Owner	nmrsu
5	Site	
6	Spectrometer	AVANCE NEO 400 MHZ DIGITAL NMR SPECTROMETER
7	Author	
8	Solvent	CDCl3
9	Temperature	300.8
10	Pulse Sequence	zgpg30
11	Experiment	1D
12	Number of Scans	4000
13	Receiver Gain	32
14	Relaxation Delay	2.0000
15	Pulse Width	10.0000
16	Acquisition Time	1.3763
17	Acquisition Date	2019-07-17T08:50:52
18	Modification Date	2019-07-17T09:18:28
19	Spectrometer Frequency	100.61
20	Spectral Width	23809.5
21	Lowest Frequency	-1832.3
22	Nucleus	13C
23	Acquired Size	32768
24	Spectral Size	32768

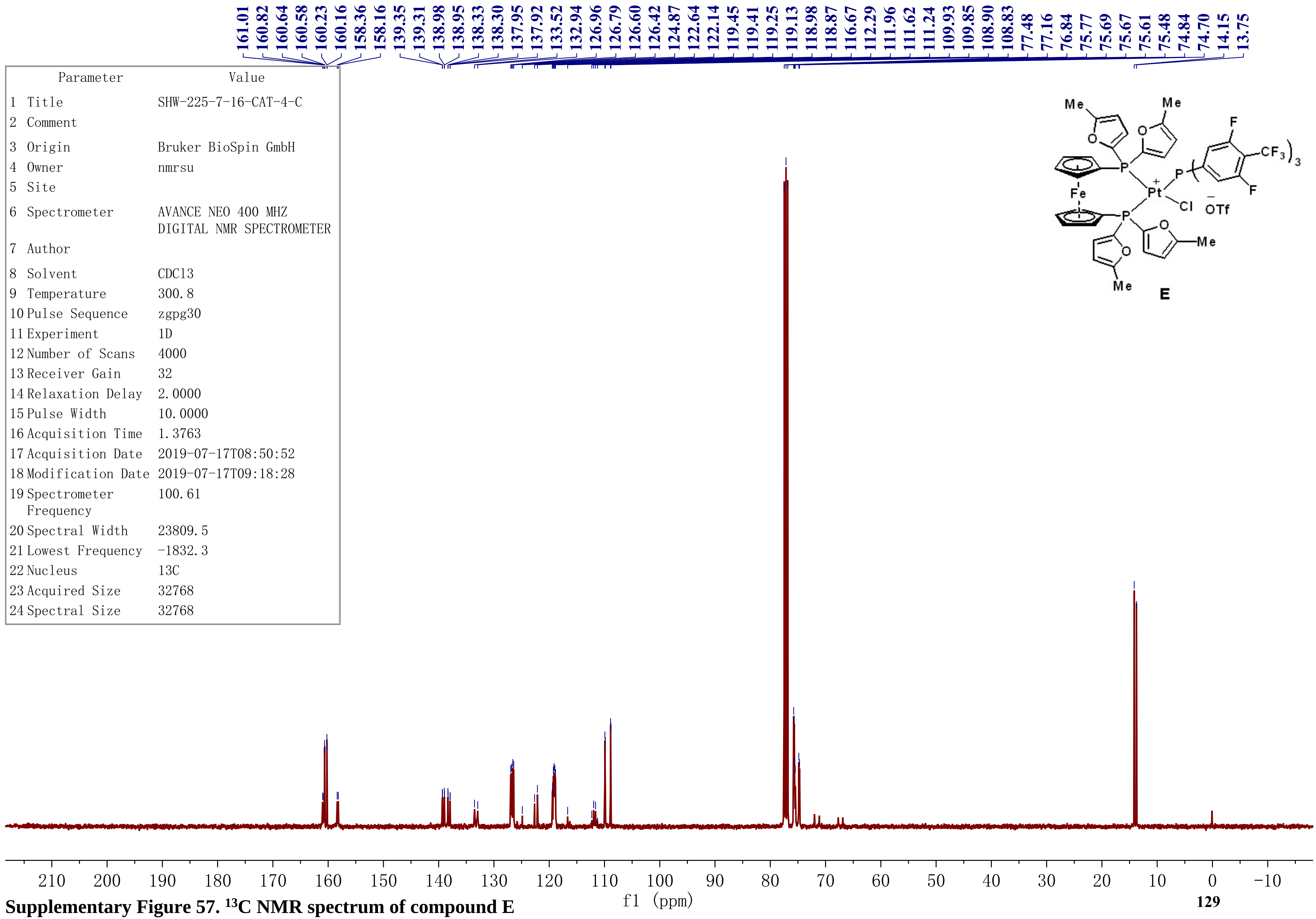

Supplementary Figure 57. ^{13}**C NMR spectrum of compound E**

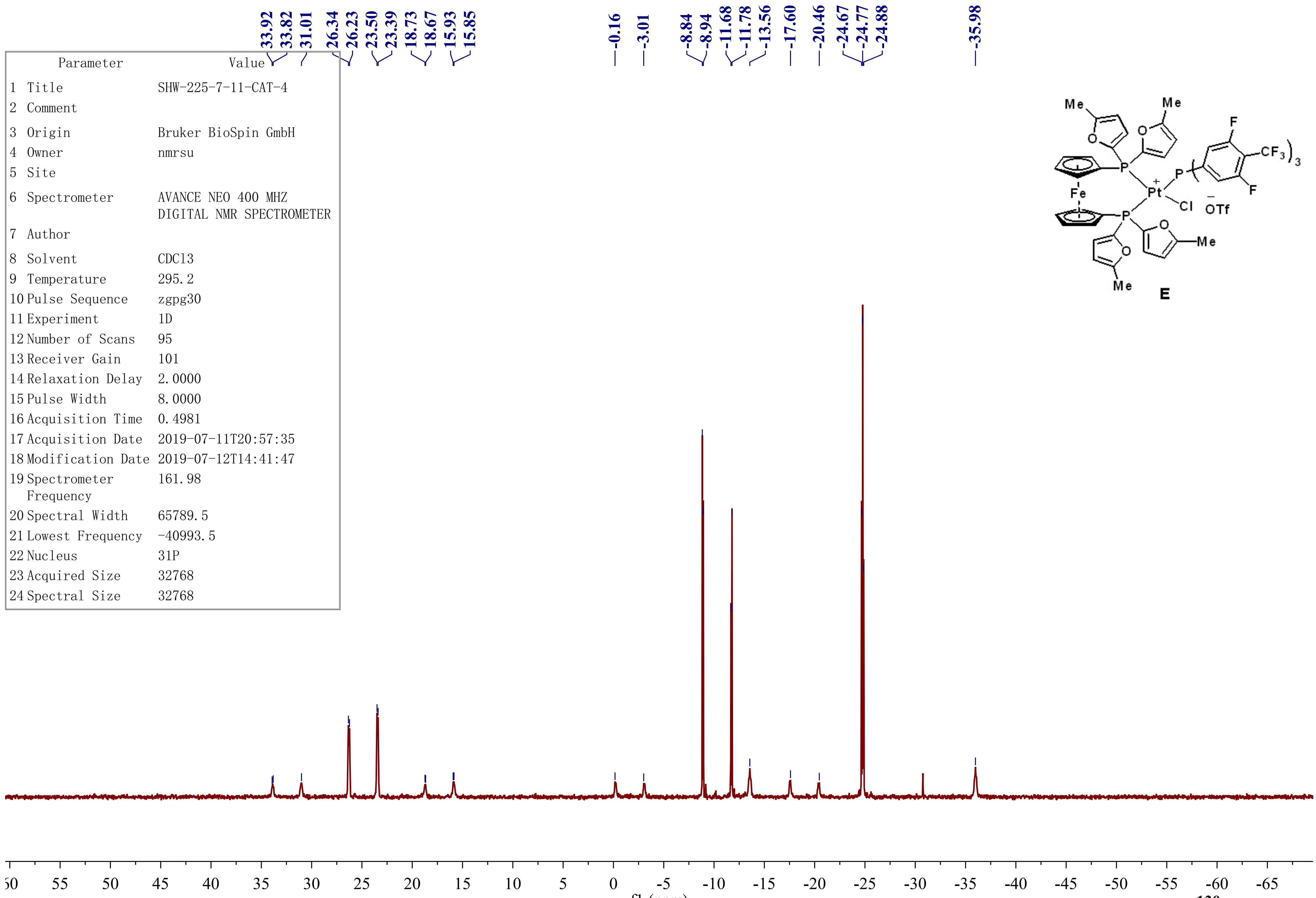

Supplementary Figure 58. ^{31}P NMR spectrum of compound E

	Parameter	Value
1	Title	SHW-225-7-11-CAT-4
2	Comment	
3	Origin	Bruker BioSpin GmbH
4	Owner	nmrsu
5	Site	
6	Spectrometer	AVANCE NEO 400 MHZ DIGITAL NMR SPECTROMETER
7	Author	
8	Solvent	CDCl3
9	Temperature	295.0
10	Pulse Sequence	zgig
11	Experiment	1D
12	Number of Scans	12
13	Receiver Gain	101
14	Relaxation Delay	1.0000
15	Pulse Width	18.0000
16	Acquisition Time	0.7209
17	Acquisition Date	2019-07-11T20:52:24
18	Modification Date	2019-07-12T14:41:47
19	Spectrometer Frequency	376.50
20	Spectral Width	90909.1
21	Lowest Frequency	-83104.4
22	Nucleus	19F
23	Acquired Size	65536
24	Spectral Size	65536

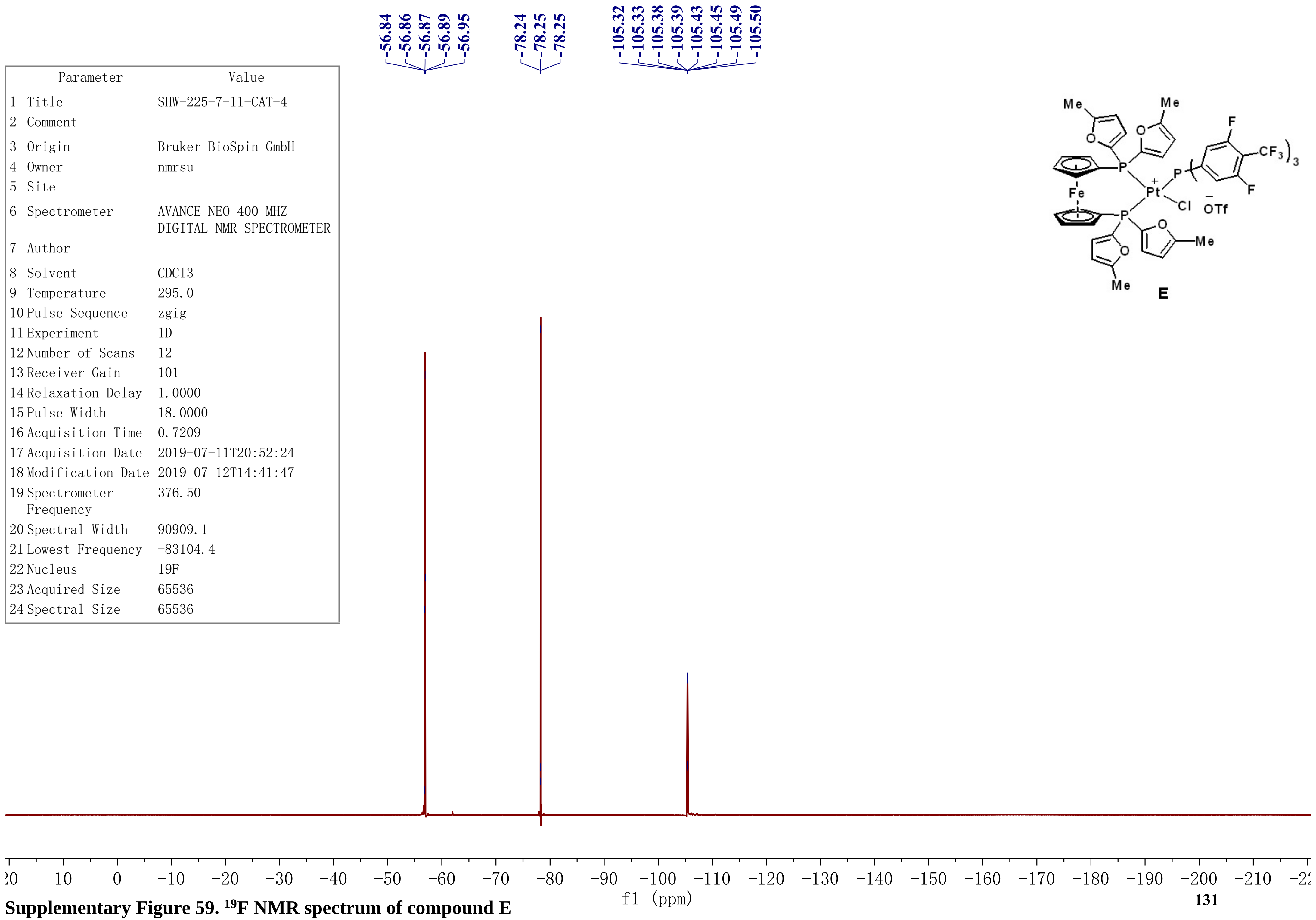

Supplementary Figure 59. ^{19}F NMR spectrum of compound E

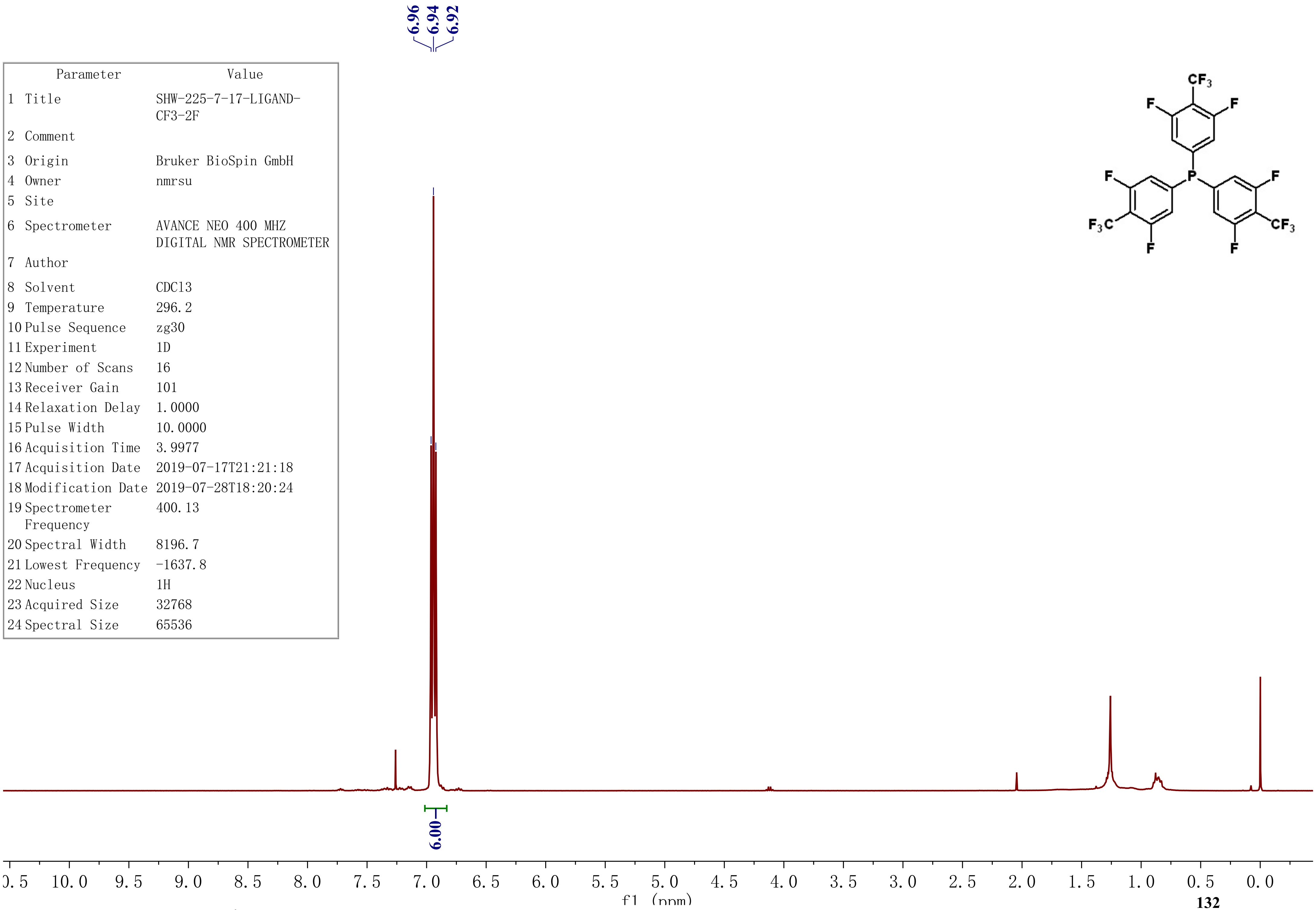

	Parameter	Value
1	Title	SHW-225-7-17-LIGAND-CF3-2F
2	Comment	
3	Origin	Bruker BioSpin GmbH
4	Owner	nmrsu
5	Site	
6	Spectrometer	AVANCE NEO 400 MHZ DIGITAL NMR SPECTROMETER
7	Author	
8	Solvent	CDCl3
9	Temperature	296.2
10	Pulse Sequence	zg30
11	Experiment	1D
12	Number of Scans	16
13	Receiver Gain	101
14	Relaxation Delay	1.0000
15	Pulse Width	10.0000
16	Acquisition Time	3.9977
17	Acquisition Date	2019-07-17T21:21:18
18	Modification Date	2019-07-28T18:20:24
19	Spectrometer Frequency	400.13
20	Spectral Width	8196.7
21	Lowest Frequency	-1637.8
22	Nucleus	1H
23	Acquired Size	32768
24	Spectral Size	65536

Supplementary Figure 60. ^{1}H NMR spectrum of tris(3,5-difluoro-4-(trifluoromethyl)phenyl)phosphine

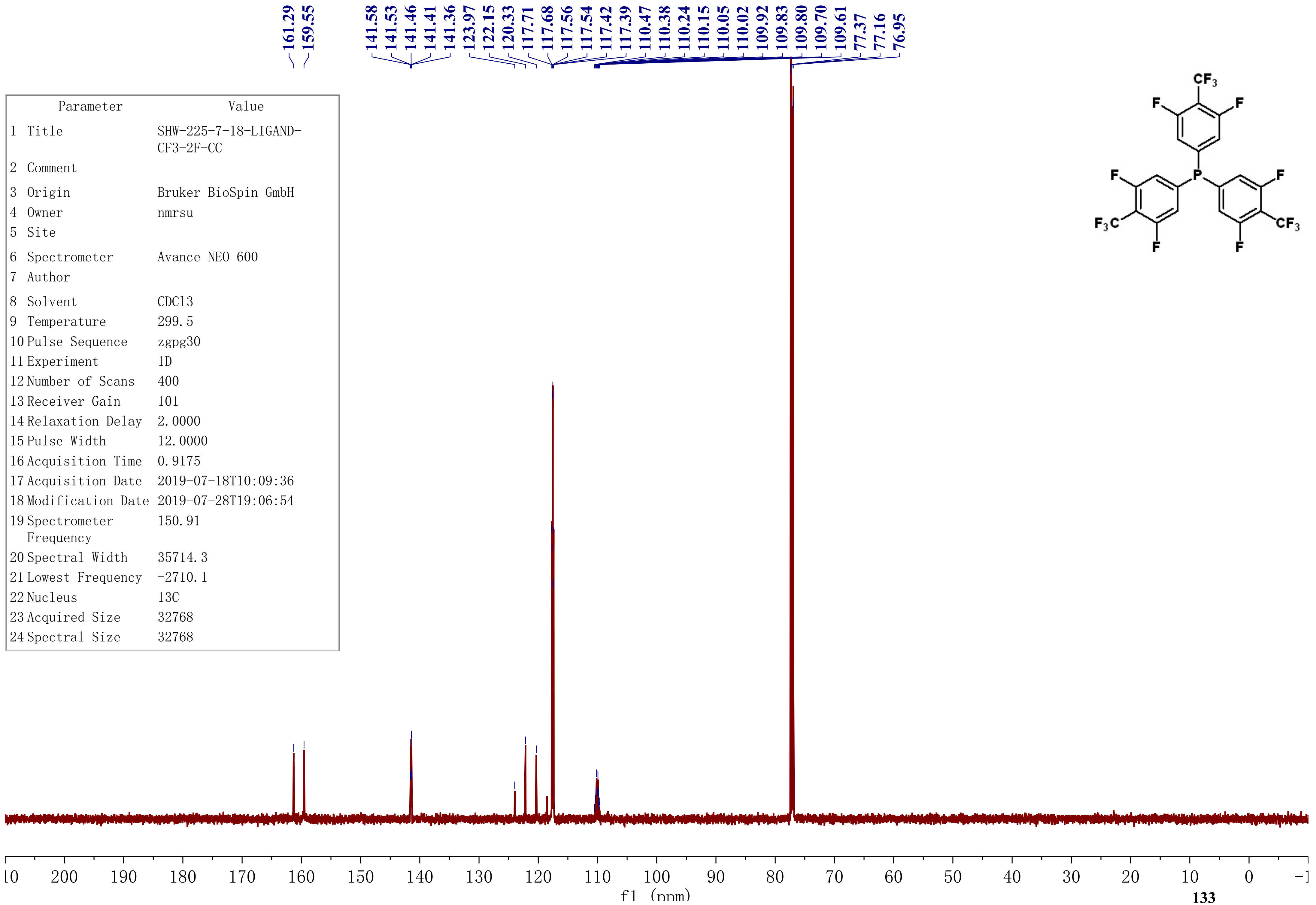

	Parameter	Value
1	Title	SHW-225-7-18-LIGAND-CF3-2F-CC
2	Comment	
3	Origin	Bruker BioSpin GmbH
4	Owner	nmrsu
5	Site	
6	Spectrometer	Avance NEO 600
7	Author	
8	Solvent	CDCl3
9	Temperature	299.5
10	Pulse Sequence	zgpg30
11	Experiment	1D
12	Number of Scans	400
13	Receiver Gain	101
14	Relaxation Delay	2.0000
15	Pulse Width	12.0000
16	Acquisition Time	0.9175
17	Acquisition Date	2019-07-18T10:09:36
18	Modification Date	2019-07-28T19:06:54
19	Spectrometer Frequency	150.91
20	Spectral Width	35714.3
21	Lowest Frequency	-2710.1
22	Nucleus	13C
23	Acquired Size	32768
24	Spectral Size	32768

Supplementary Figure 61. ^{13}C **NMR spectrum of tris(3,5-difluoro-4-(trifluoromethyl)phenyl)phosphine**

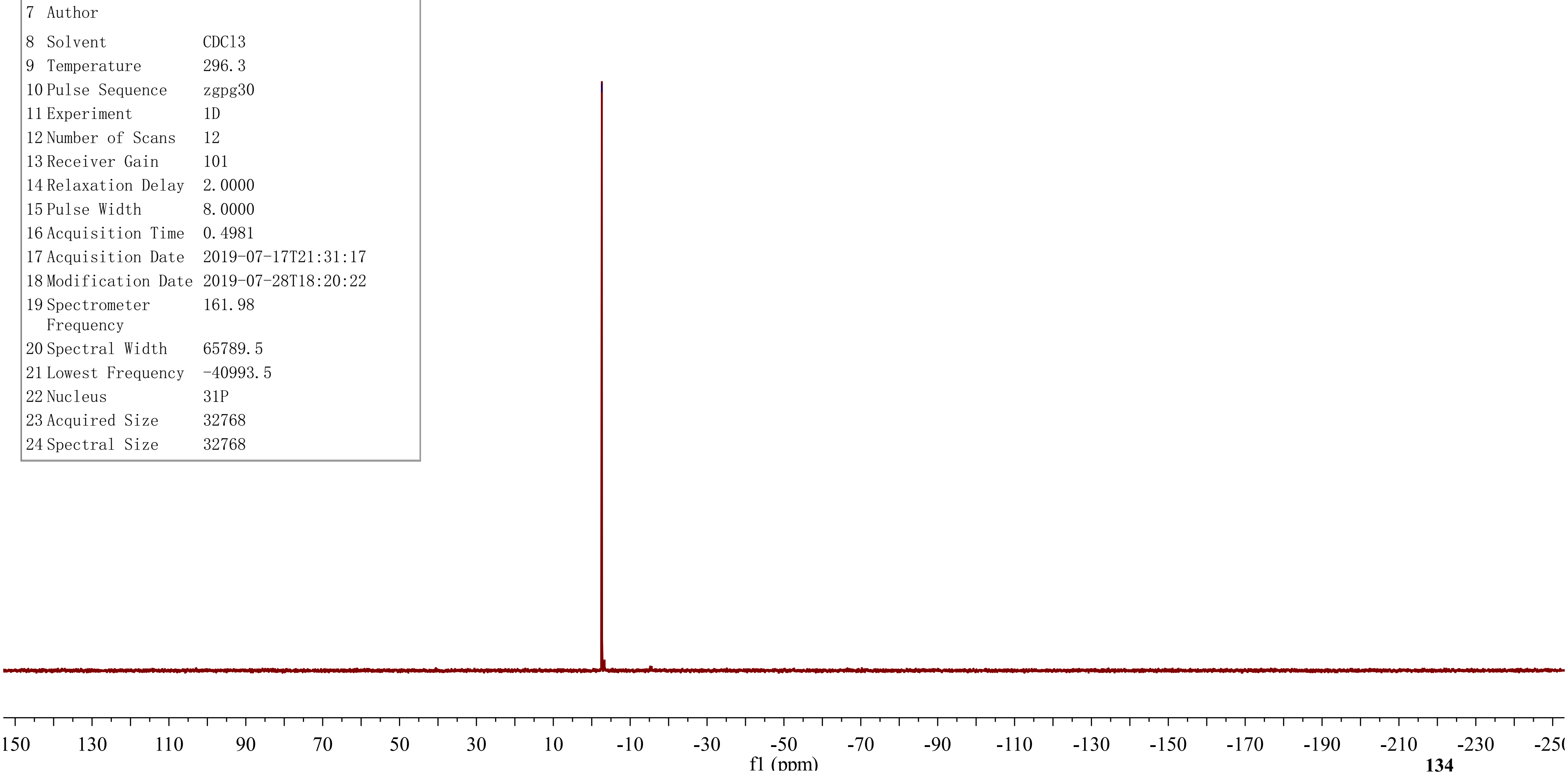

Parameter	Value
1 Title	SHW-225-7-17-LIGAND-CF3-2F
2 Comment	
3 Origin	Bruker BioSpin GmbH
4 Owner	nmrsu
5 Site	
6 Spectrometer	AVANCE NEO 400 MHZ DIGITAL NMR SPECTROMETER
7 Author	
8 Solvent	CDCl3
9 Temperature	296.3
10 Pulse Sequence	zgpg30
11 Experiment	1D
12 Number of Scans	12
13 Receiver Gain	101
14 Relaxation Delay	2.0000
15 Pulse Width	8.0000
16 Acquisition Time	0.4981
17 Acquisition Date	2019-07-17T21:31:17
18 Modification Date	2019-07-28T18:20:22
19 Spectrometer Frequency	161.98
20 Spectral Width	65789.5
21 Lowest Frequency	-40993.5
22 Nucleus	31P
23 Acquired Size	32768
24 Spectral Size	32768

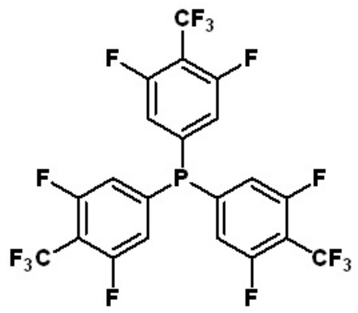

Supplementary Figure 62. ^{31}P NMR spectrum of tris(3,5-difluoro-4-(trifluoromethyl)phenyl)phosphine

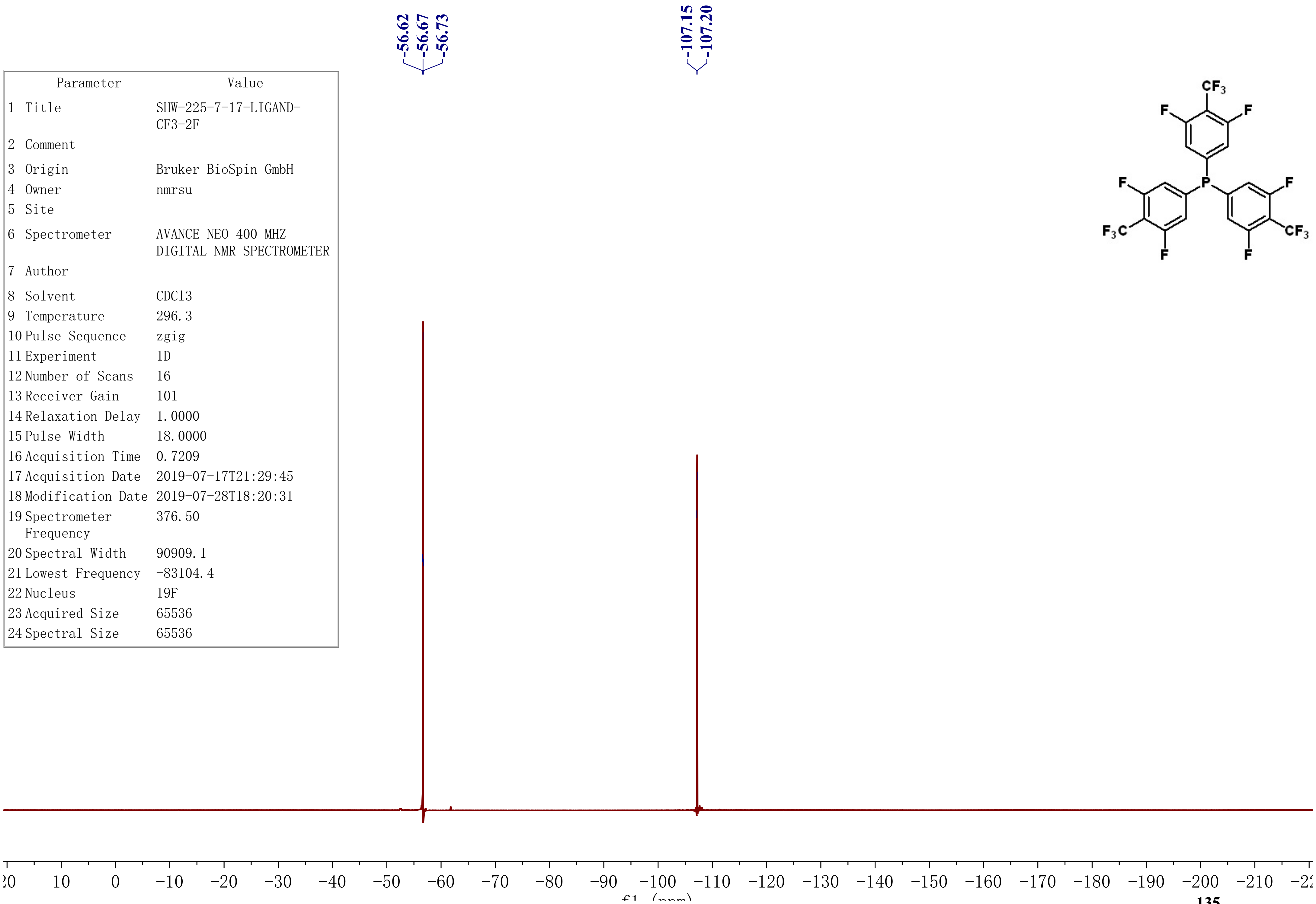

	Parameter	Value
1	Title	SHW-225-7-17-LIGAND-CF3-2F
2	Comment	
3	Origin	Bruker BioSpin GmbH
4	Owner	nmrsu
5	Site	
6	Spectrometer	AVANCE NEO 400 MHZ DIGITAL NMR SPECTROMETER
7	Author	
8	Solvent	CDCl3
9	Temperature	296.3
10	Pulse Sequence	zgig
11	Experiment	1D
12	Number of Scans	16
13	Receiver Gain	101
14	Relaxation Delay	1.0000
15	Pulse Width	18.0000
16	Acquisition Time	0.7209
17	Acquisition Date	2019-07-17T21:29:45
18	Modification Date	2019-07-28T18:20:31
19	Spectrometer Frequency	376.50
20	Spectral Width	90909.1
21	Lowest Frequency	-83104.4
22	Nucleus	19F
23	Acquired Size	65536
24	Spectral Size	65536

Supplementary Figure 63. ^{19}F NMR spectrum of tris(3,5-difluoro-4-(trifluoromethyl)phenyl)phosphine

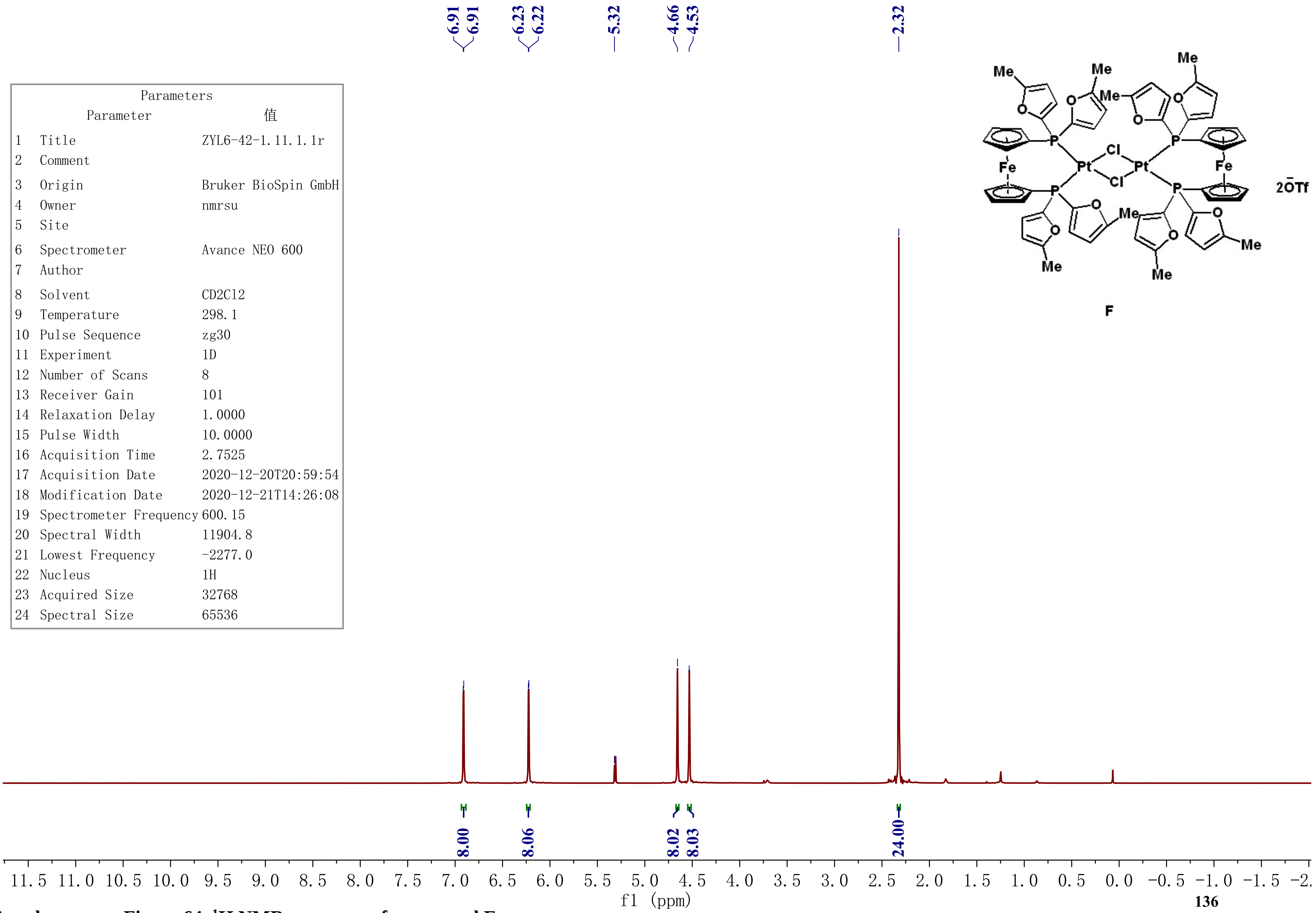

Supplementary Figure 64. ^{1}H NMR spectrum of compound F

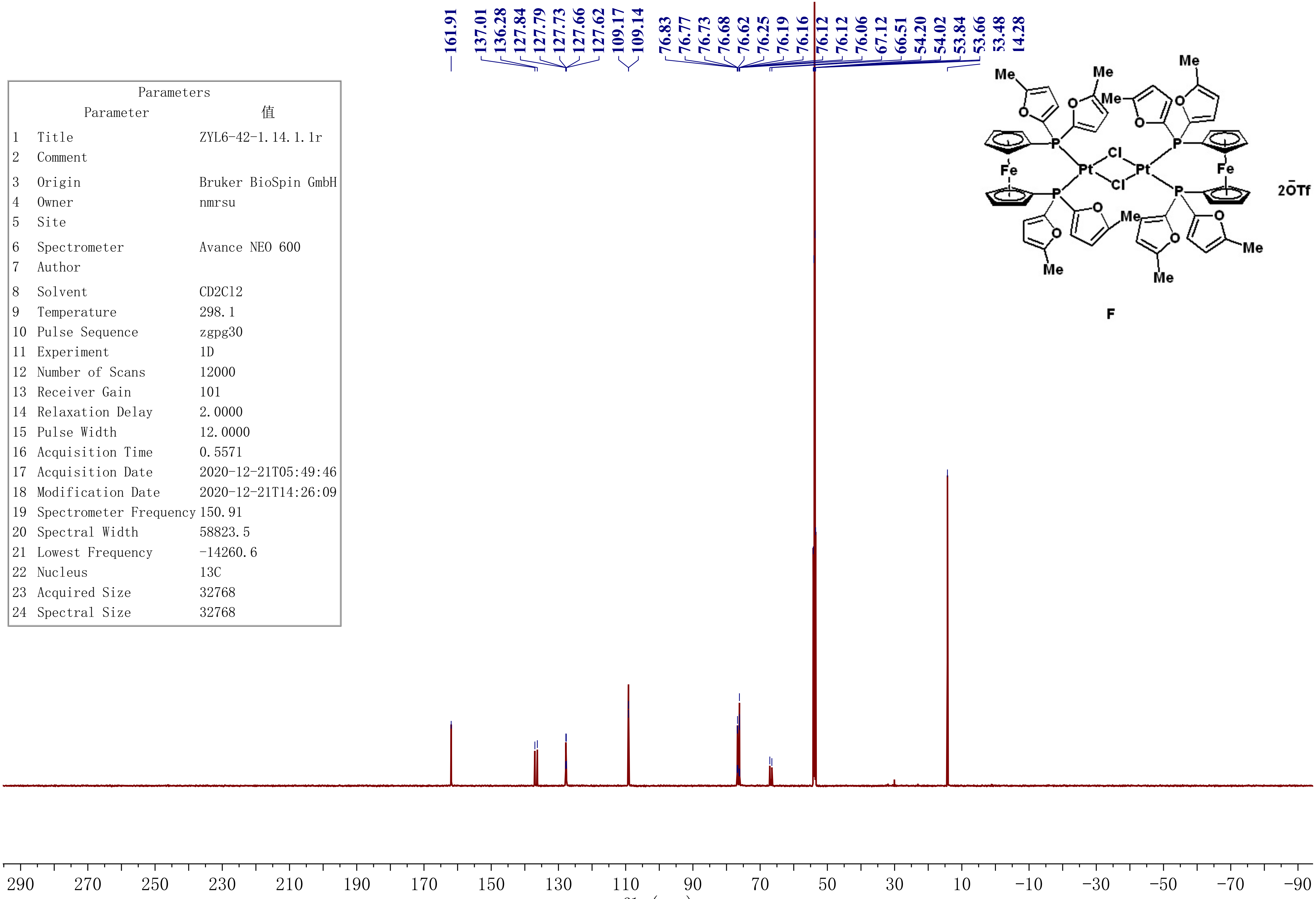

Supplementary Figure 65. ^{13}C NMR spectrum of compound F

Parameters

	Parameter	值
1	Title	ZYL6-42-1.15.1.1r
2	Comment	
3	Origin	Bruker BioSpin GmbH
4	Owner	nmrsu
5	Site	
6	Spectrometer	Avance NEO 600
7	Author	
8	Solvent	CD2Cl2
9	Temperature	298.1
10	Pulse Sequence	zgpg30
11	Experiment	1D
12	Number of Scans	100
13	Receiver Gain	101
14	Relaxation Delay	2.0000
15	Pulse Width	12.0000
16	Acquisition Time	0.3408
17	Acquisition Date	2020-12-21T05:55:35
18	Modification Date	2020-12-21T14:26:10
19	Spectrometer Frequency	242.95
20	Spectral Width	96153.8
21	Lowest Frequency	-60224.2
22	Nucleus	31P
23	Acquired Size	32768
24	Spectral Size	32768

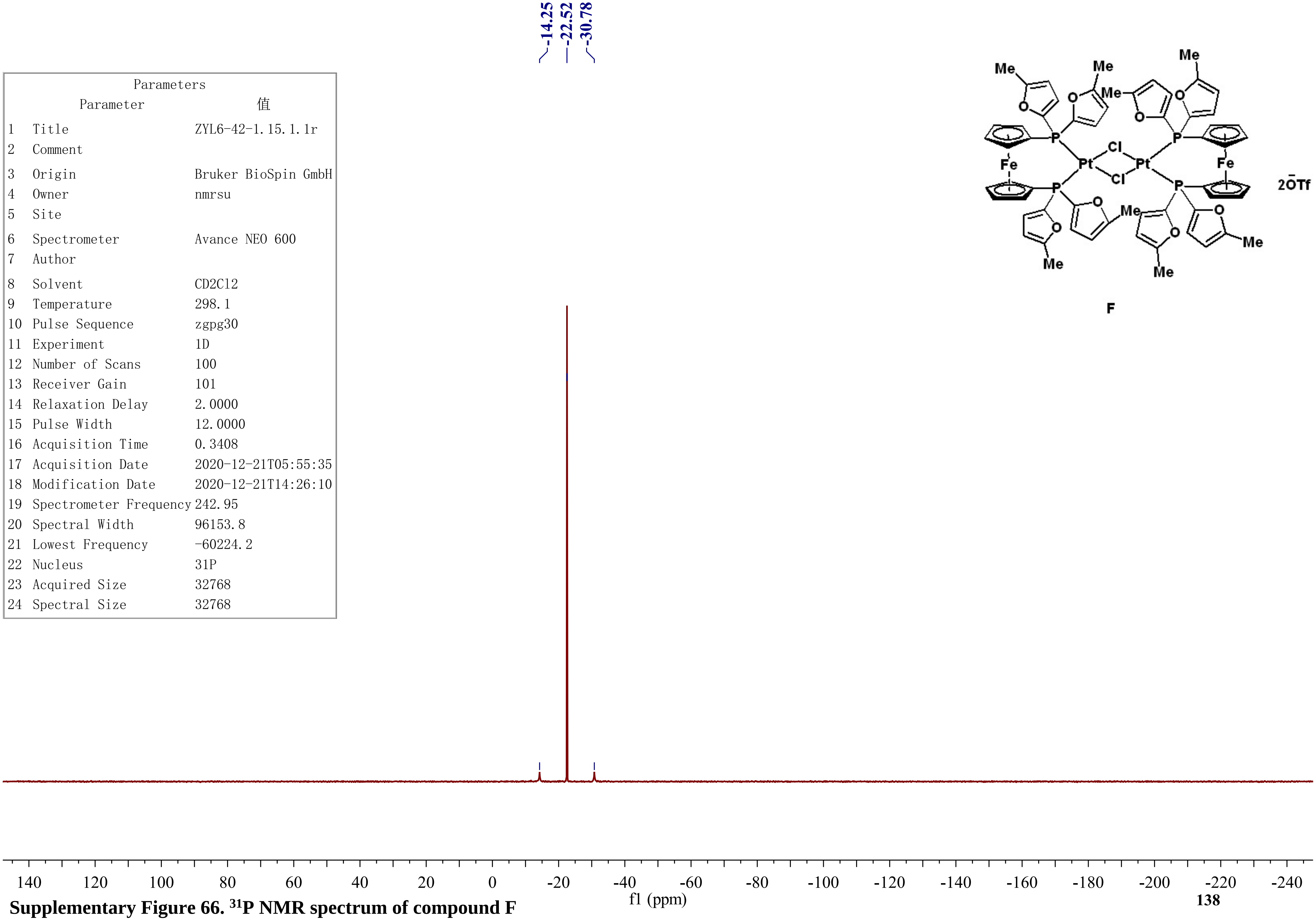

F

Supplementary Figure 66. ^{31}P NMR spectrum of compound F

	Parameter	值
1	Title	ZYL6-42-1.16.1.1r
2	Comment	
3	Origin	Bruker BioSpin GmbH
4	Owner	nmrsu
5	Site	
6	Spectrometer	Avance NEO 600
7	Author	
8	Solvent	CD2Cl2
9	Temperature	298.1
10	Pulse Sequence	zgig
11	Experiment	1D
12	Number of Scans	16
13	Receiver Gain	101
14	Relaxation Delay	1.0000
15	Pulse Width	12.0000
16	Acquisition Time	0.4981
17	Acquisition Date	2020-12-21T05:57:30
18	Modification Date	2020-12-21T14:26:10
19	Spectrometer Frequency	564.71
20	Spectral Width	131578.9
21	Lowest Frequency	-122260.0
22	Nucleus	19F
23	Acquired Size	65536
24	Spectral Size	65536

Parameters

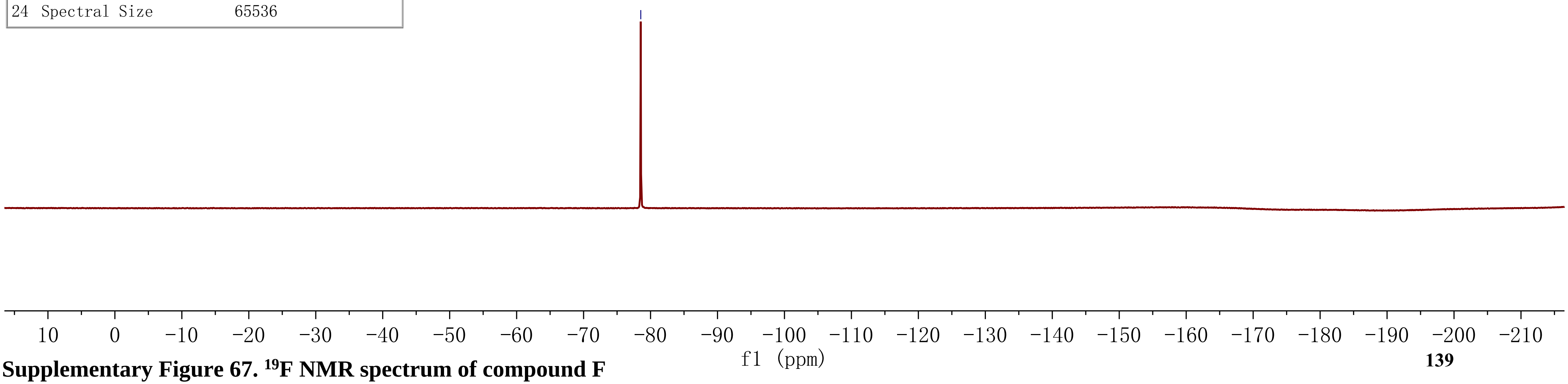

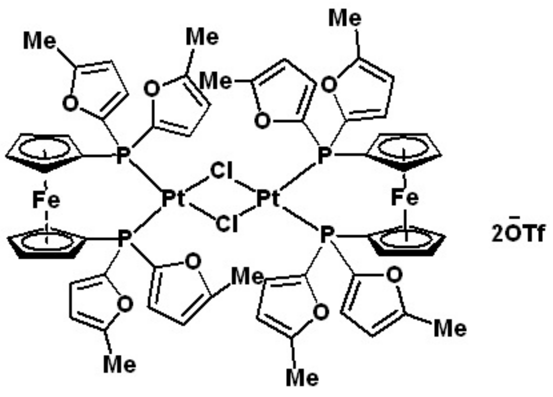

F

Supplementary Figure 67. ^{19}F NMR spectrum of compound F

Parameters		
	Parameter	値
1	Title	ZYL6-DPPF.20.1.1r
2	Comment	
3	Origin	Bruker BioSpin GmbH
4	Owner	nmrsu
5	Site	
6	Spectrometer	Avance Neo 400M
7	Author	
8	Solvent	CD2Cl2
9	Temperature	298.1
10	Pulse Sequence	zg30
11	Experiment	1D
12	Number of Scans	6
13	Receiver Gain	101
14	Relaxation Delay	1.0000
15	Pulse Width	8.0000
16	Acquisition Time	3.9977
17	Acquisition Date	2020-12-24T23:50:01
18	Modification Date	2020-12-25T09:57:01
19	Spectrometer Frequency	400.18
20	Spectral Width	8196.7
21	Lowest Frequency	-1643.2
22	Nucleus	1H
23	Acquired Size	32768
24	Spectral Size	65536

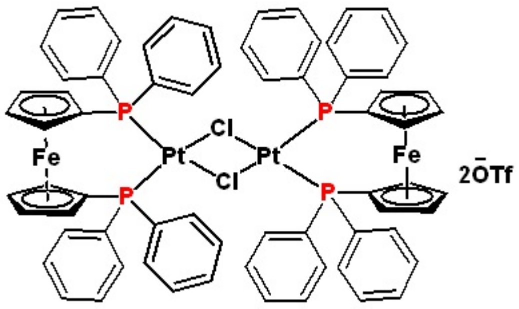

Q

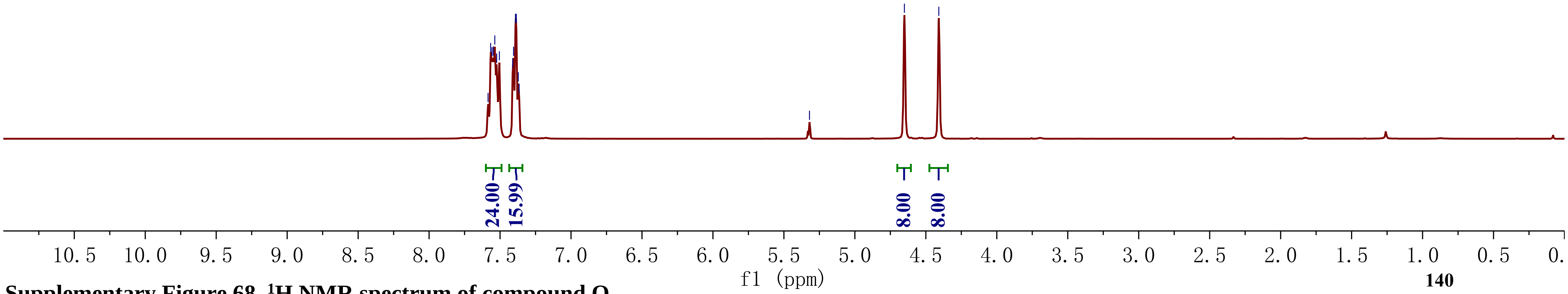

Supplementary Figure 68. ^{1}H NMR spectrum of compound Q

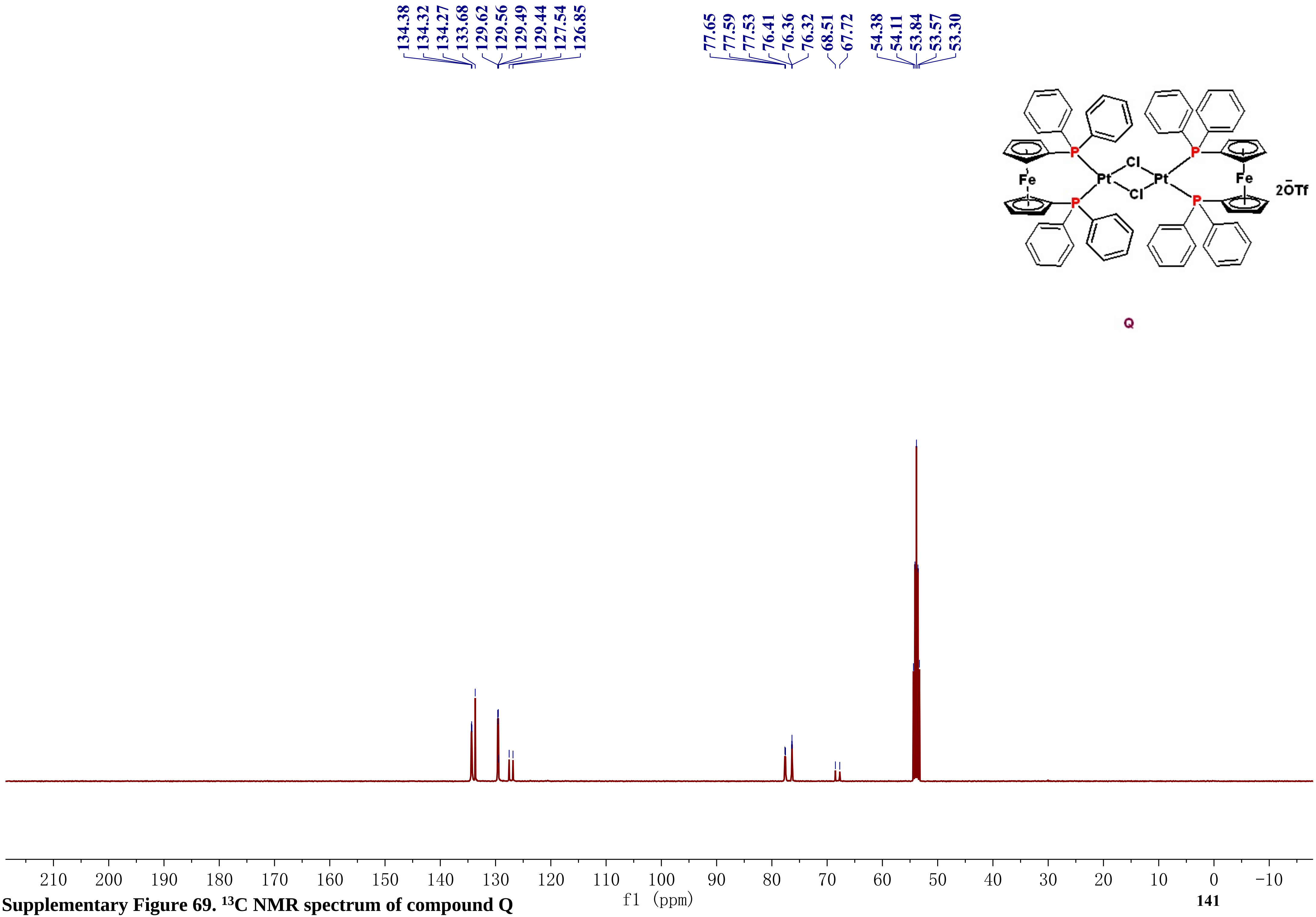

Supplementary Figure 69. ^{13}C NMR spectrum of compound Q

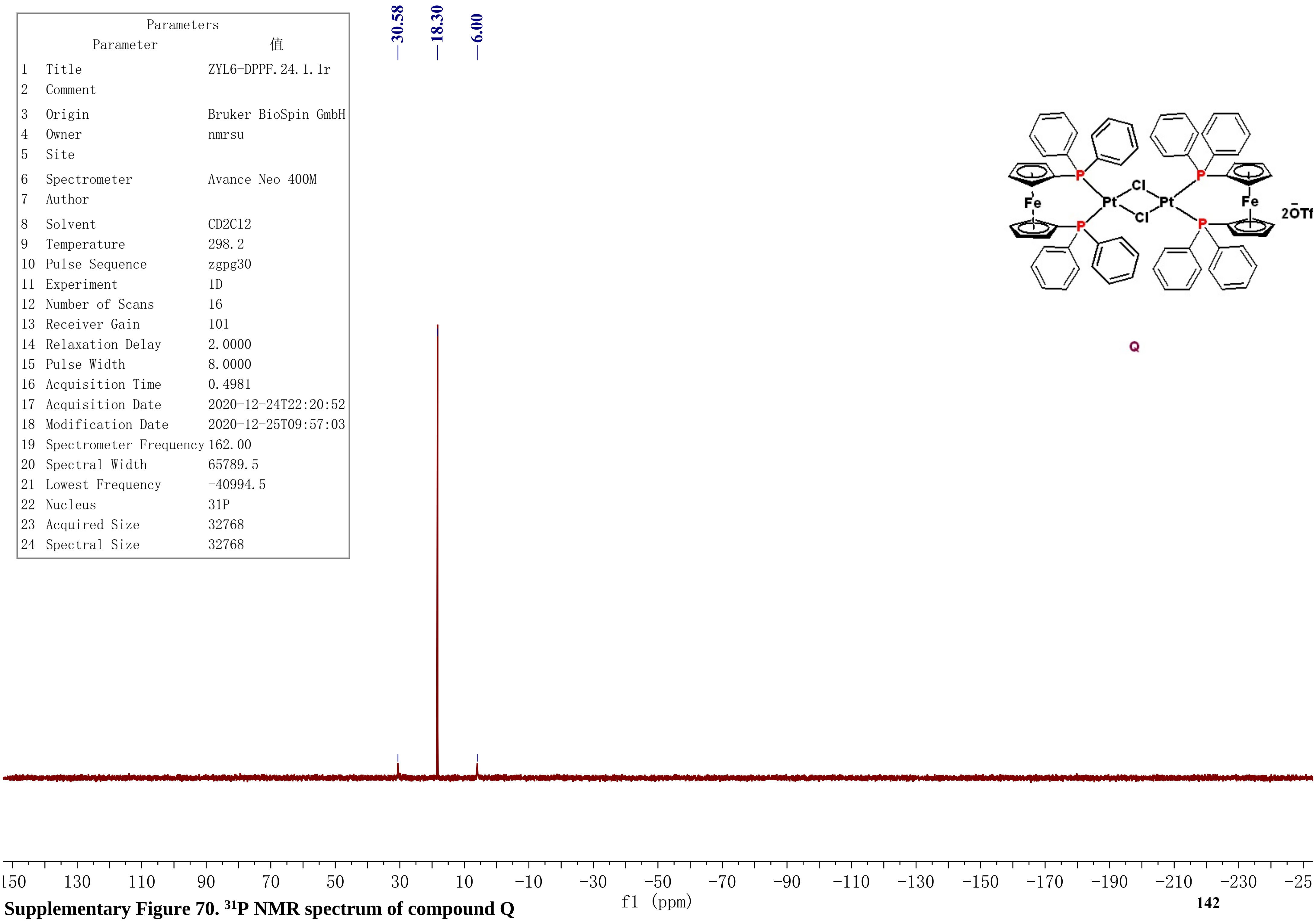

Supplementary Figure 70. ^{31}**P NMR spectrum of compound Q**

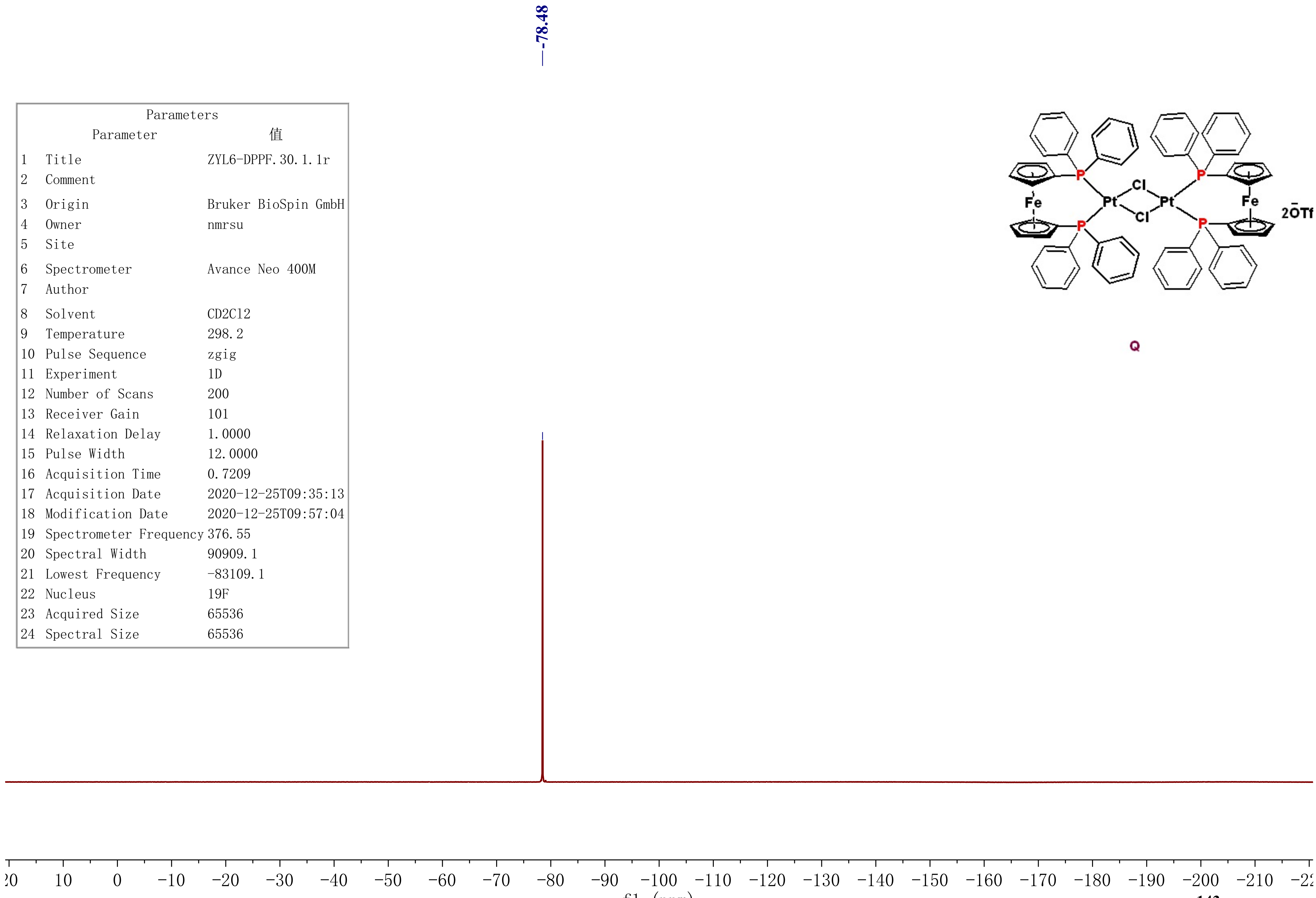

Supplementary Figure 71. ^{19}F **NMR spectrum of compound Q**

Parameter		Value
1	Title	ZYLS5
2	Comment	
3	Origin	Bruker BioSpin GmbH
4	Owner	nmrsu
5	Site	
6	Spectrometer	AVANCE NEO 400 MHZ DIGITAL NMR SPECTROMETER
7	Author	
8	Solvent	CDCl3
9	Temperature	295.5
10	Pulse Sequence	zg30
11	Experiment	1D
12	Number of Scans	7
13	Receiver Gain	32
14	Relaxation Delay	1.0000
15	Pulse Width	10.0000
16	Acquisition Time	3.9977
17	Acquisition Date	2019-01-03T11:02:21
18	Modification Date	2019-01-03T12:31:20
19	Spectrometer Frequency	400.13
20	Spectral Width	8196.7
21	Lowest Frequency	-1637.7
22	Nucleus	1H
23	Acquired Size	32768
24	Spectral Size	65536

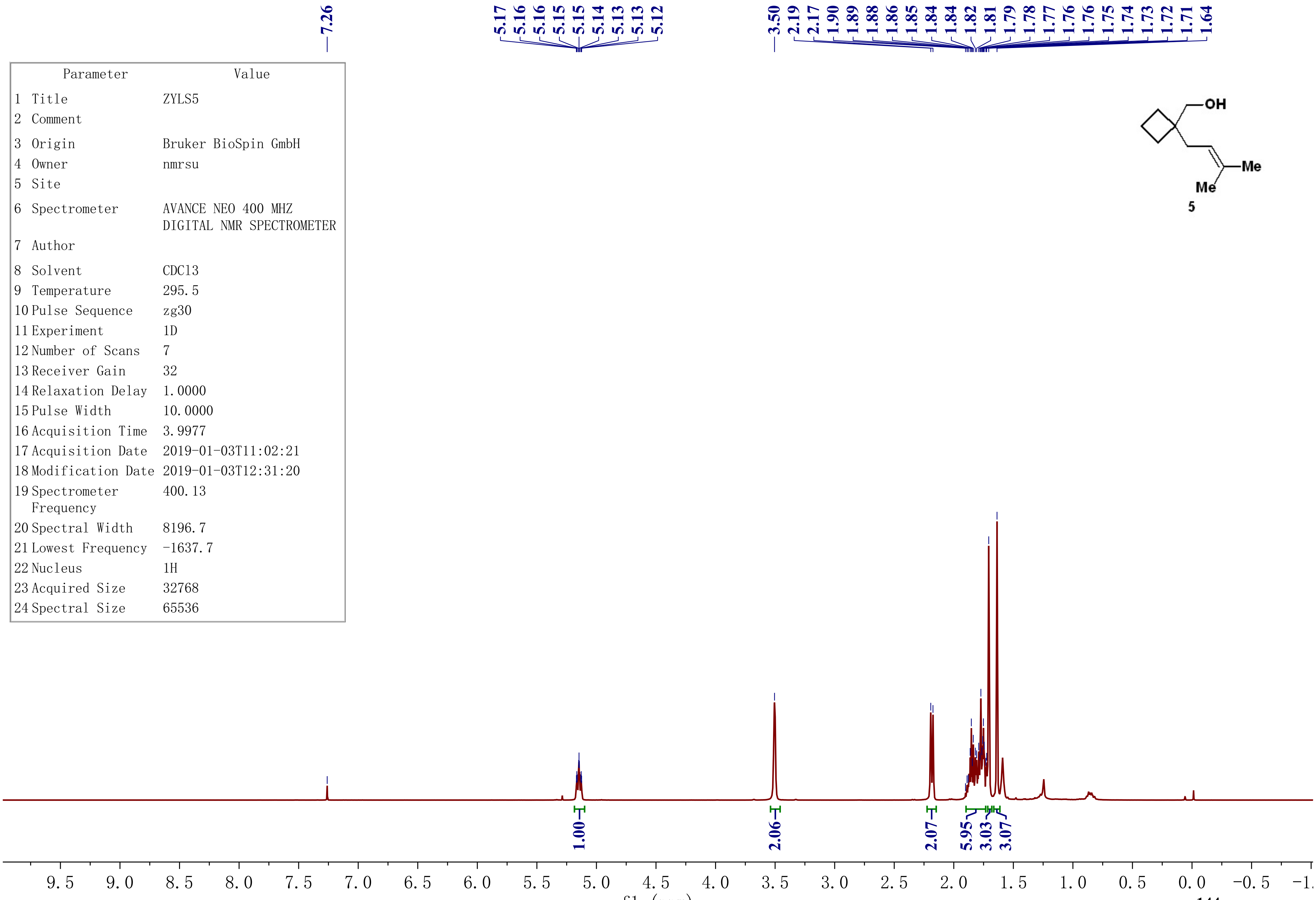

Supplementary Figure 72. ^{1}H NMR spectrum of compound 5

Parameter		Value
1	Title	ZYLS5
2	Comment	
3	Origin	Bruker BioSpin GmbH
4	Owner	nmrsu
5	Site	
6	Spectrometer	AVANCE NEO 400 MHZ DIGITAL NMR SPECTROMETER
7	Author	
8	Solvent	CDCl3
9	Temperature	295.8
10	Pulse Sequence	zgpg30
11	Experiment	1D
12	Number of Scans	11
13	Receiver Gain	32
14	Relaxation Delay	2.0000
15	Pulse Width	10.0000
16	Acquisition Time	1.3763
17	Acquisition Date	2019-01-03T11:03:57
18	Modification Date	2019-01-03T12:31:20
19	Spectrometer Frequency	100.61
20	Spectral Width	23809.5
21	Lowest Frequency	-1833.0
22	Nucleus	13C
23	Acquired Size	32768
24	Spectral Size	32768

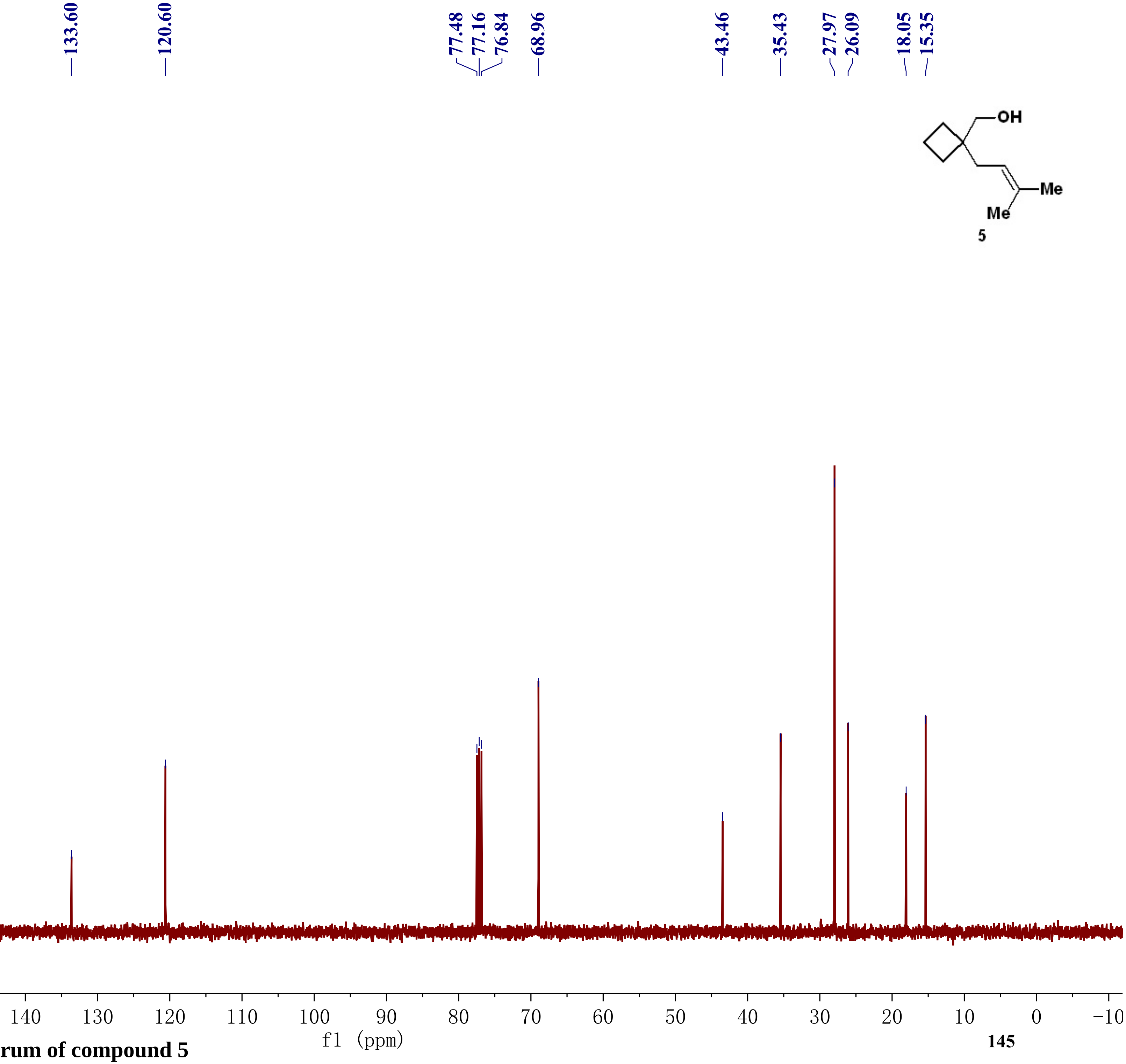

Supplementary Figure 72. ^{13}C NMR spectrum of compound 5

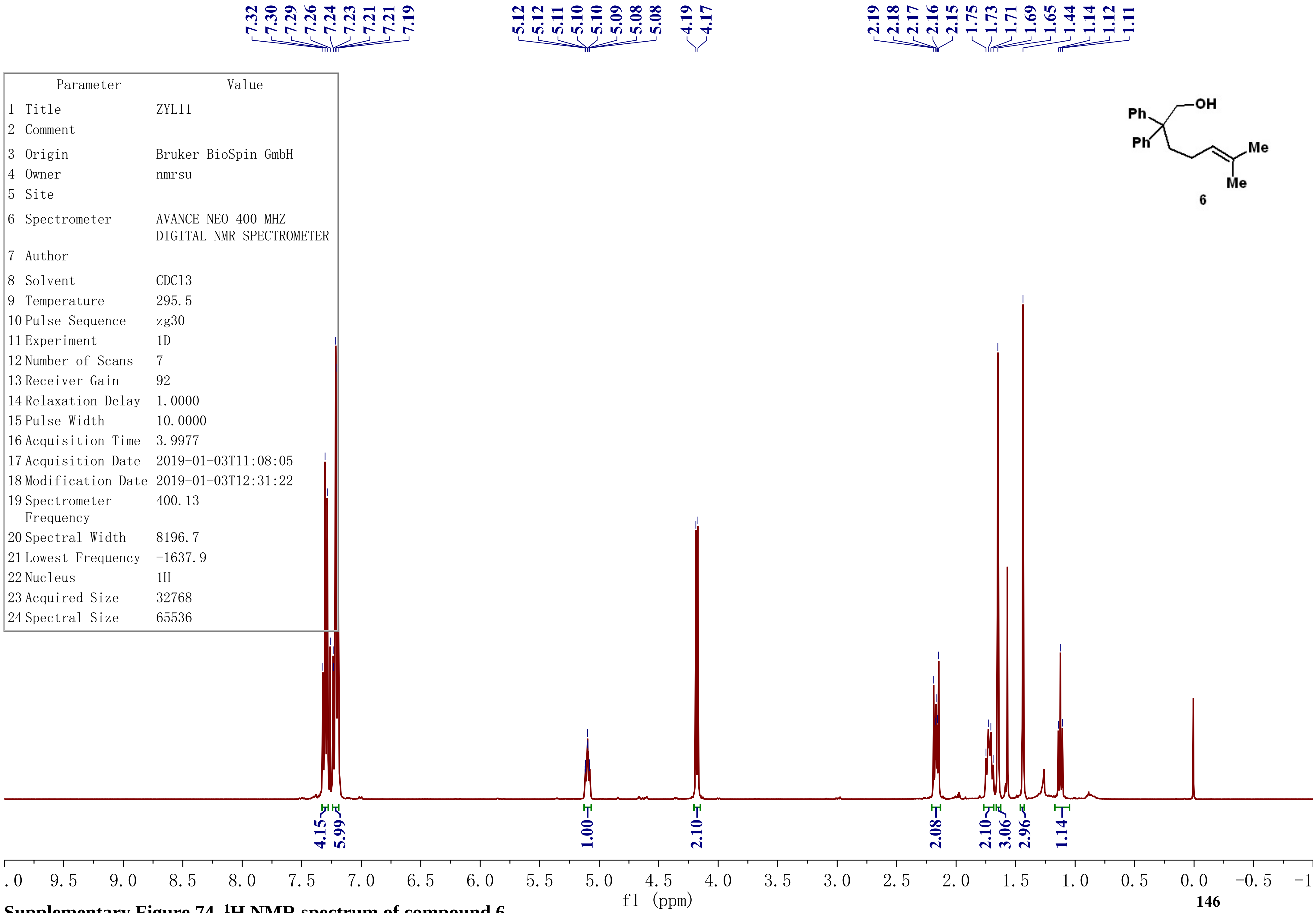

Supplementary Figure 74. ^{1}H NMR spectrum of compound 6

Parameter	Value
1 Title	ZYL11
2 Comment	
3 Origin	Bruker BioSpin GmbH
4 Owner	nmrsu
5 Site	
6 Spectrometer	AVANCE NEO 400 MHZ DIGITAL NMR SPECTROMETER
7 Author	
8 Solvent	CDCl3
9 Temperature	296.0
10 Pulse Sequence	zgpg30
11 Experiment	1D
12 Number of Scans	30
13 Receiver Gain	32
14 Relaxation Delay	2.0000
15 Pulse Width	10.0000
16 Acquisition Time	1.3763
17 Acquisition Date	2019-01-03T11:21:35
18 Modification Date	2019-01-03T12:31:22
19 Spectrometer Frequency	100.61
20 Spectral Width	23809.5
21 Lowest Frequency	-1832.1
22 Nucleus	13C
23 Acquired Size	32768
24 Spectral Size	32768

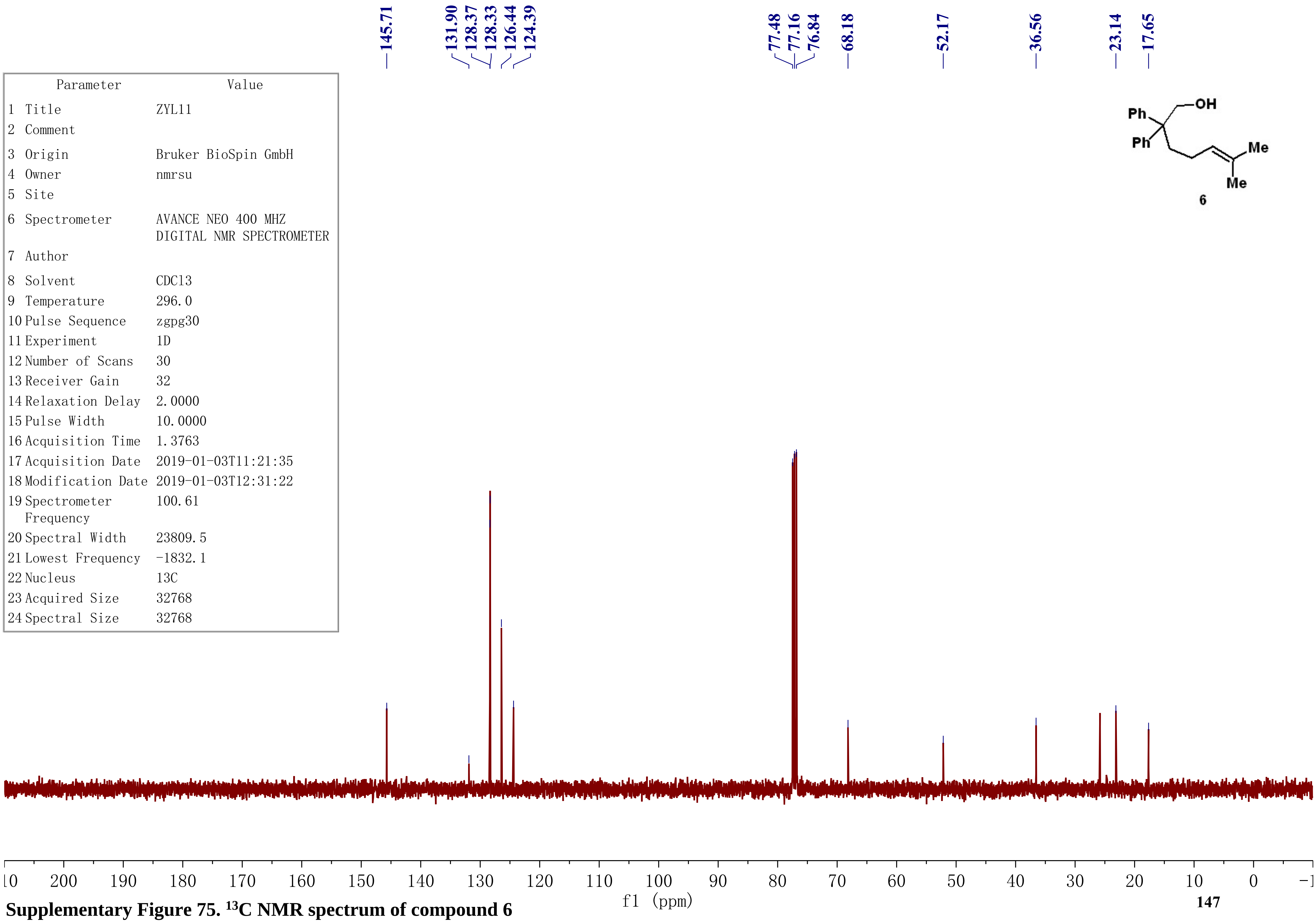

Supplementary Figure 75. ^{13}C NMR spectrum of compound 6

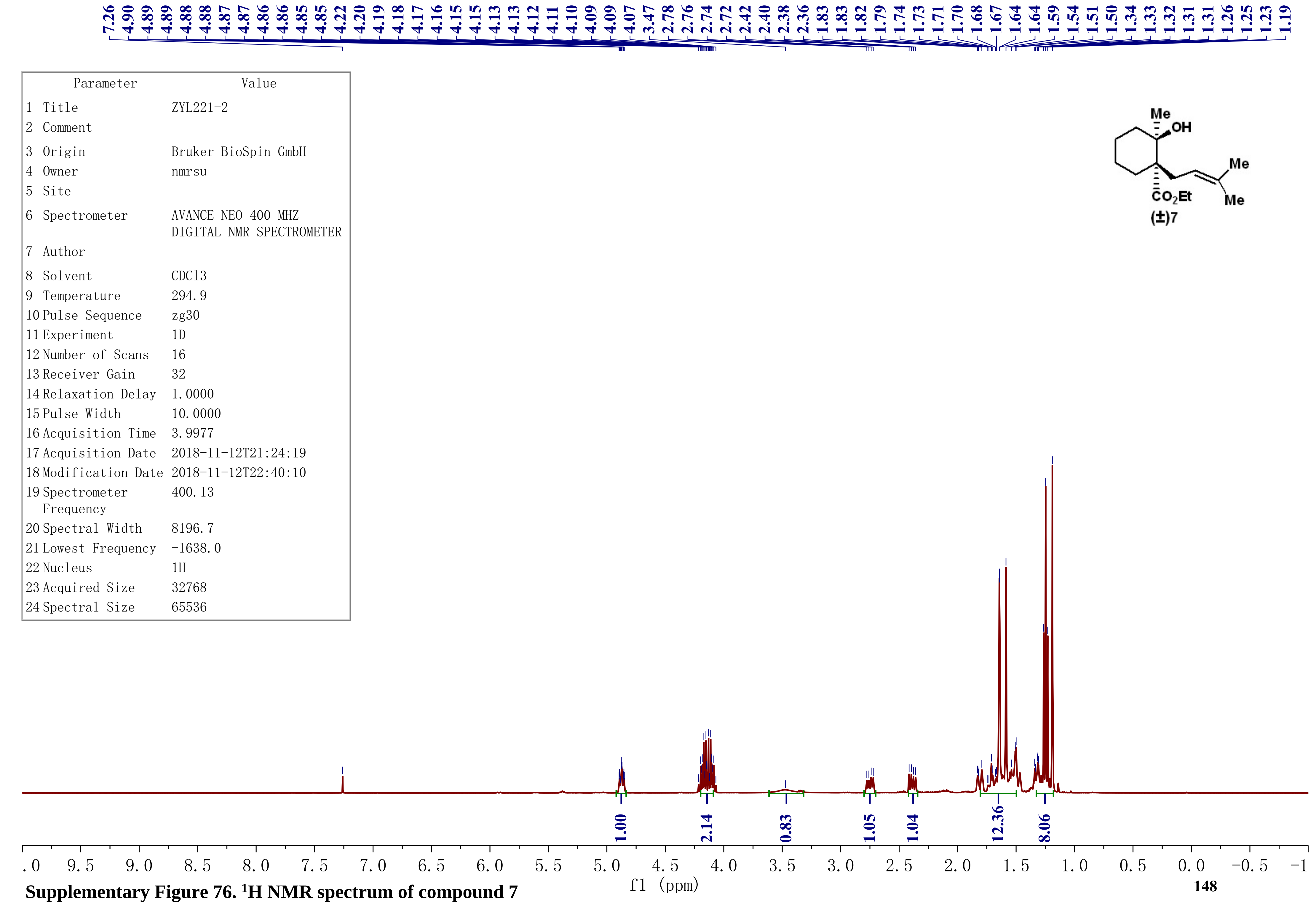

	Parameter	Value
1	Title	ZYL221-2
2	Comment	
3	Origin	Bruker BioSpin GmbH
4	Owner	nmrsu
5	Site	
6	Spectrometer	AVANCE NEO 400 MHZ DIGITAL NMR SPECTROMETER
7	Author	
8	Solvent	CDCl3
9	Temperature	294.9
10	Pulse Sequence	zg30
11	Experiment	1D
12	Number of Scans	16
13	Receiver Gain	32
14	Relaxation Delay	1.0000
15	Pulse Width	10.0000
16	Acquisition Time	3.9977
17	Acquisition Date	2018-11-12T21:24:19
18	Modification Date	2018-11-12T22:40:10
19	Spectrometer Frequency	400.13
20	Spectral Width	8196.7
21	Lowest Frequency	-1638.0
22	Nucleus	1H
23	Acquired Size	32768
24	Spectral Size	65536

Supplementary Figure 76. ^{1}H **NMR spectrum of compound 7**

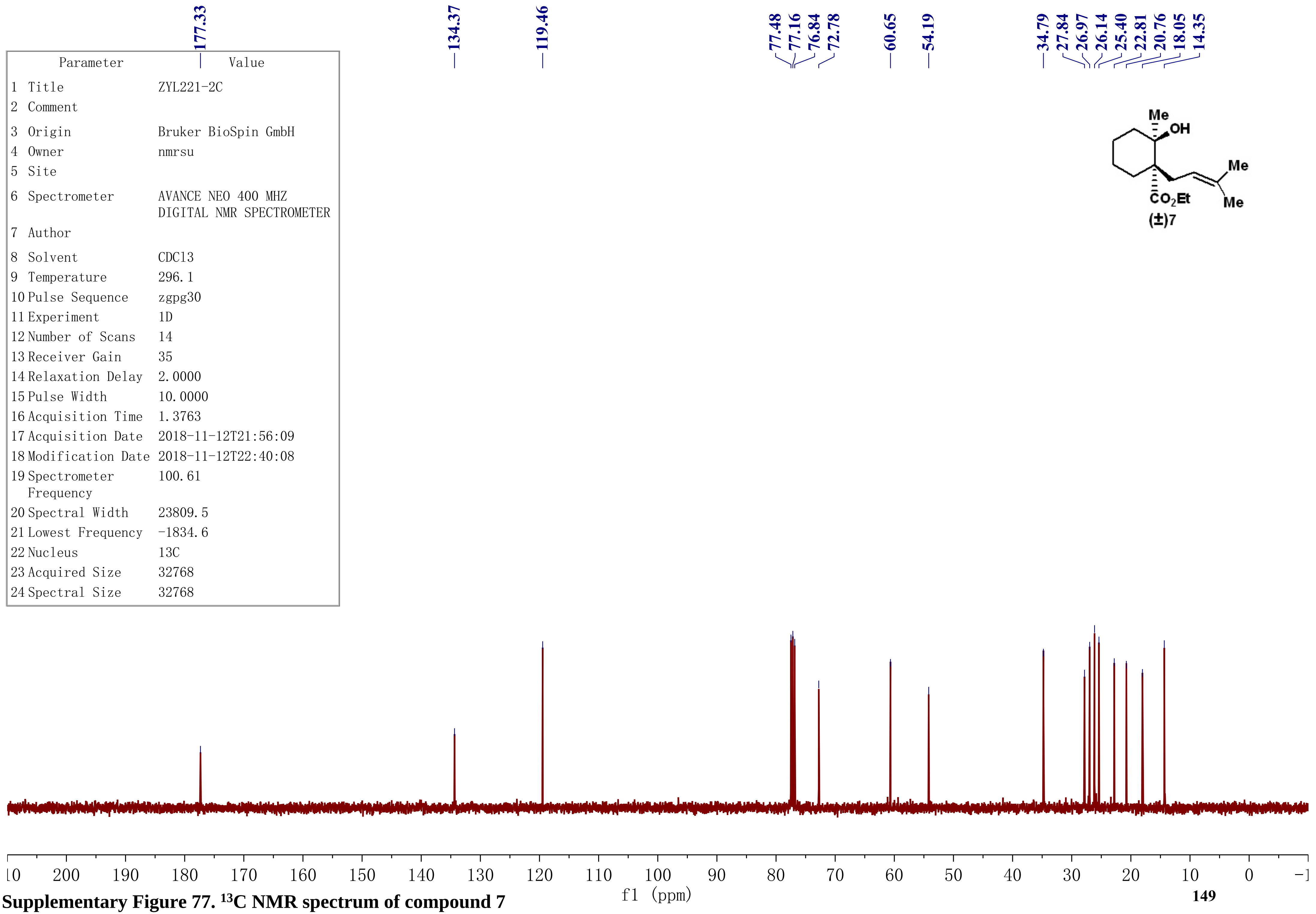

Parameter	Value
1 Title	ZYL221-2C
2 Comment	
3 Origin	Bruker BioSpin GmbH
4 Owner	nmrsu
5 Site	
6 Spectrometer	AVANCE NEO 400 MHZ DIGITAL NMR SPECTROMETER
7 Author	
8 Solvent	CDCl3
9 Temperature	296.1
10 Pulse Sequence	zgpg30
11 Experiment	1D
12 Number of Scans	14
13 Receiver Gain	35
14 Relaxation Delay	2.0000
15 Pulse Width	10.0000
16 Acquisition Time	1.3763
17 Acquisition Date	2018-11-12T21:56:09
18 Modification Date	2018-11-12T22:40:08
19 Spectrometer Frequency	100.61
20 Spectral Width	23809.5
21 Lowest Frequency	-1834.6
22 Nucleus	13C
23 Acquired Size	32768
24 Spectral Size	32768

Supplementary Figure 77. ^{13}C NMR spectrum of compound 7

Parameter	Value
1 Data File Name	C:/ ZYL251/ 1/ pdata/ 1/ 1r
2 Title	ZYL251
3 Comment	
4 Origin	Bruker BioSpin GmbH
5 Owner	nmr
6 Site	
7 Spectrometer	AVANCE NEO 400 MHZ DIGITAL NMR SPECTROMETER
8 Author	
9 Solvent	CDCl3
10 Temperature	295.3
11 Pulse Sequence	zg30
12 Experiment	1D
13 Number of Scans	16
14 Receiver Gain	32
15 Relaxation Delay	1.0000
16 Pulse Width	10.0000
17 Acquisition Time	3.9977
18 Acquisition Date	2018-11-24T16:04:48
19 Modification Date	2019-01-03T12:19:53
20 Spectrometer Frequency	400.13
21 Spectral Width	8196.7
22 Lowest Frequency	-1637.8
23 Nucleus	1H
24 Acquired Size	32768
25 Spectral Size	65536

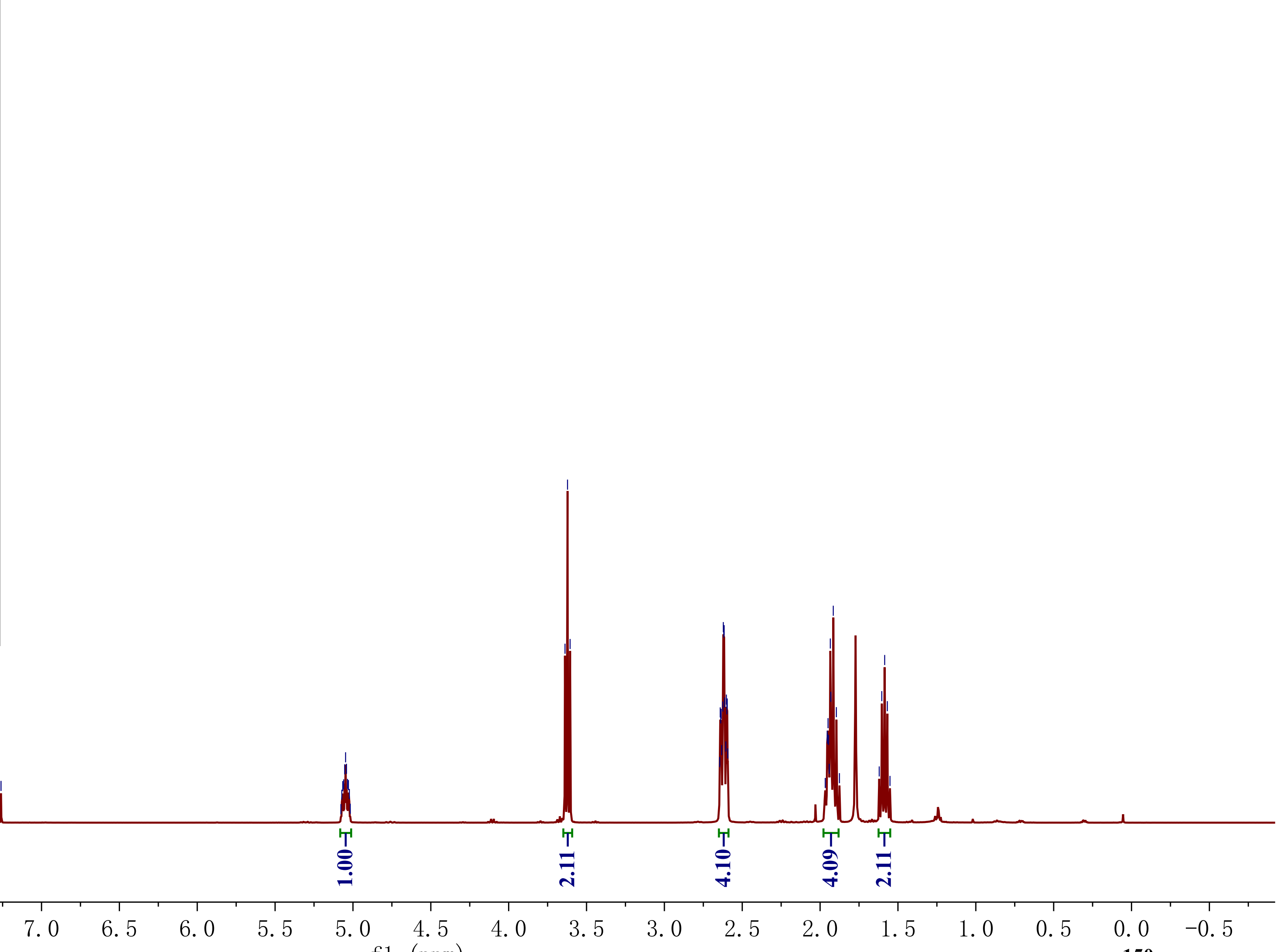

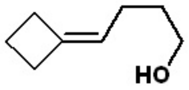

8

Supplementary Figure 78. ^{1}H NMR spectrum of compound 8

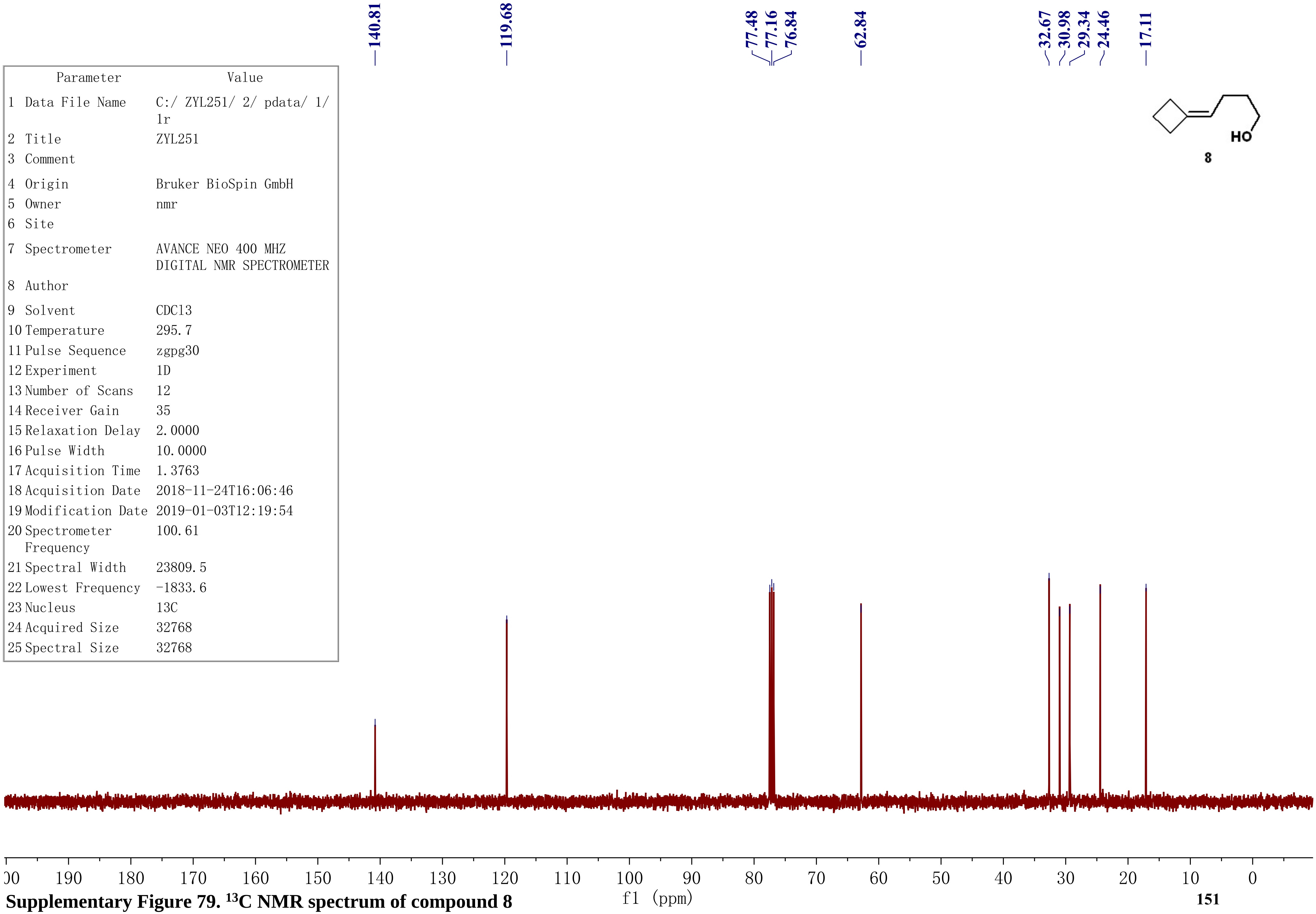

Parameter	Value
1 Data File Name	C:/ ZYL251/ 2/ pdata/ 1/ 1r
2 Title	ZYL251
3 Comment	
4 Origin	Bruker BioSpin GmbH
5 Owner	nmr
6 Site	
7 Spectrometer	AVANCE NEO 400 MHZ DIGITAL NMR SPECTROMETER
8 Author	
9 Solvent	CDCl3
10 Temperature	295.7
11 Pulse Sequence	zgpg30
12 Experiment	1D
13 Number of Scans	12
14 Receiver Gain	35
15 Relaxation Delay	2.0000
16 Pulse Width	10.0000
17 Acquisition Time	1.3763
18 Acquisition Date	2018-11-24T16:06:46
19 Modification Date	2019-01-03T12:19:54
20 Spectrometer Frequency	100.61
21 Spectral Width	23809.5
22 Lowest Frequency	-1833.6
23 Nucleus	13C
24 Acquired Size	32768
25 Spectral Size	32768

Supplementary Figure 79. ^{13}C NMR spectrum of compound 8

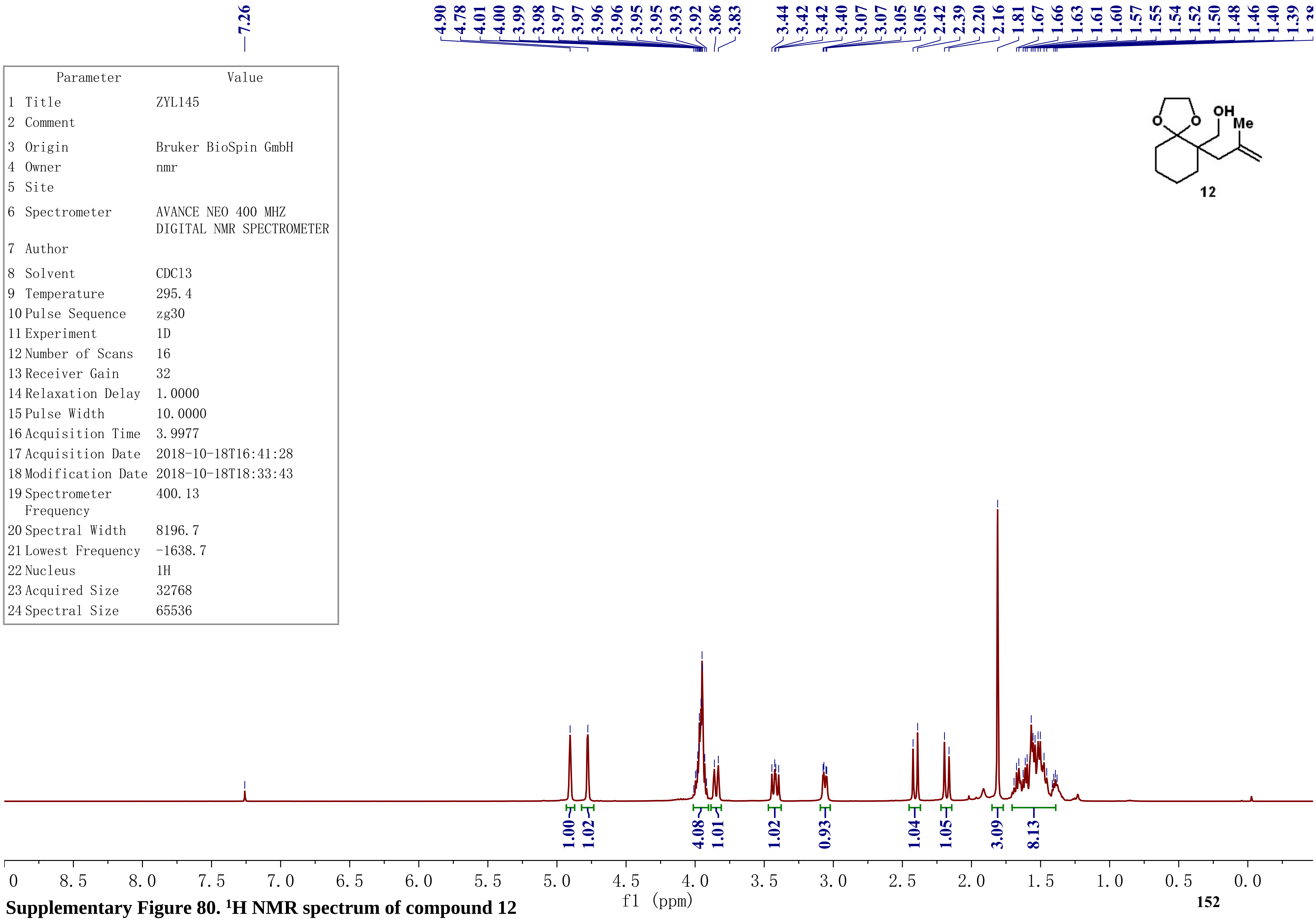

	Parameter	Value
1	Title	ZYL145
2	Comment	
3	Origin	Bruker BioSpin GmbH
4	Owner	nmr
5	Site	
6	Spectrometer	AVANCE NEO 400 MHZ DIGITAL NMR SPECTROMETER
7	Author	
8	Solvent	CDCl3
9	Temperature	295.4
10	Pulse Sequence	zg30
11	Experiment	1D
12	Number of Scans	16
13	Receiver Gain	32
14	Relaxation Delay	1.0000
15	Pulse Width	10.0000
16	Acquisition Time	3.9977
17	Acquisition Date	2018-10-18T16:41:28
18	Modification Date	2018-10-18T18:33:43
19	Spectrometer Frequency	400.13
20	Spectral Width	8196.7
21	Lowest Frequency	-1638.7
22	Nucleus	1H
23	Acquired Size	32768
24	Spectral Size	65536

Supplementary Figure 80. ^{1}H NMR spectrum of compound 12

Parameter	Value
1 Title	ZYL145C
2 Comment	
3 Origin	Bruker BioSpin GmbH
4 Owner	nmr
5 Site	
6 Spectrometer	AVANCE NEO 400 MHZ DIGITAL NMR SPECTROMETER
7 Author	
8 Solvent	CDCl3
9 Temperature	295.8
10 Pulse Sequence	zgpg30
11 Experiment	1D
12 Number of Scans	16
13 Receiver Gain	32
14 Relaxation Delay	2.0000
15 Pulse Width	10.0000
16 Acquisition Time	1.3763
17 Acquisition Date	2018-10-18T18:29:26
18 Modification Date	2018-10-18T18:33:47
19 Spectrometer Frequency	100.61
20 Spectral Width	23809.5
21 Lowest Frequency	-1834.6
22 Nucleus	13C
23 Acquired Size	32768
24 Spectral Size	32768

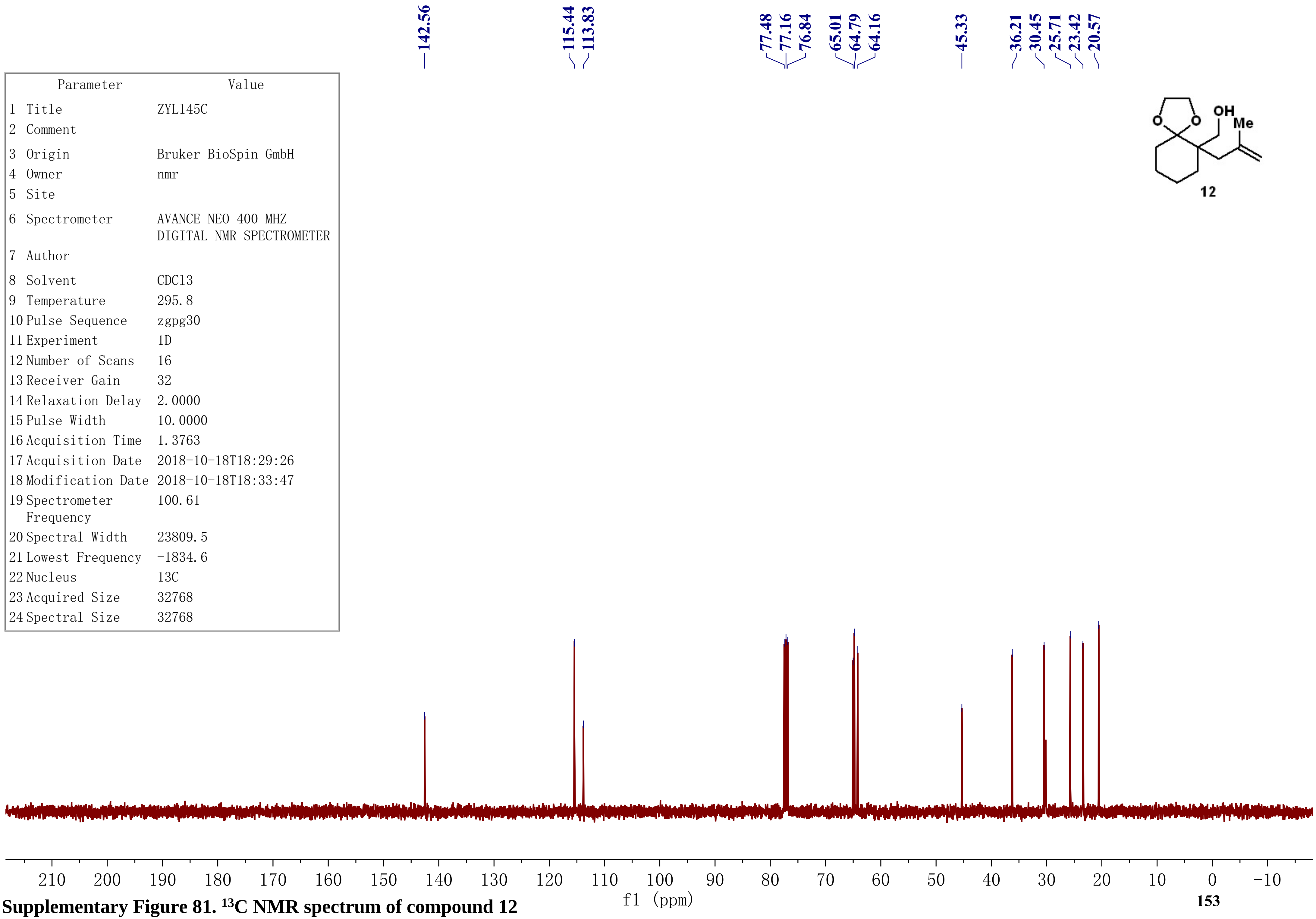

12

Supplementary Figure 81. ^{13}C **NMR spectrum of compound 12**

Parameter	Value
1 Title	ZTL320-4
2 Comment	
3 Origin	Bruker BioSpin GmbH
4 Owner	nmrsu
5 Site	
6 Spectrometer	Avance NEO 600
7 Author	
8 Solvent	CDCl3
9 Temperature	297.4
10 Pulse Sequence	zg30
11 Experiment	1D
12 Number of Scans	16
13 Receiver Gain	32
14 Relaxation Delay	1.0000
15 Pulse Width	10.0000
16 Acquisition Time	2.7525
17 Acquisition Date	2019-01-24T20:22:48
18 Modification Date	2019-01-26T14:06:15
19 Spectrometer Frequency	600.15
20 Spectral Width	11904.8
21 Lowest Frequency	-2274.0
22 Nucleus	1H
23 Acquired Size	32768
24 Spectral Size	65536

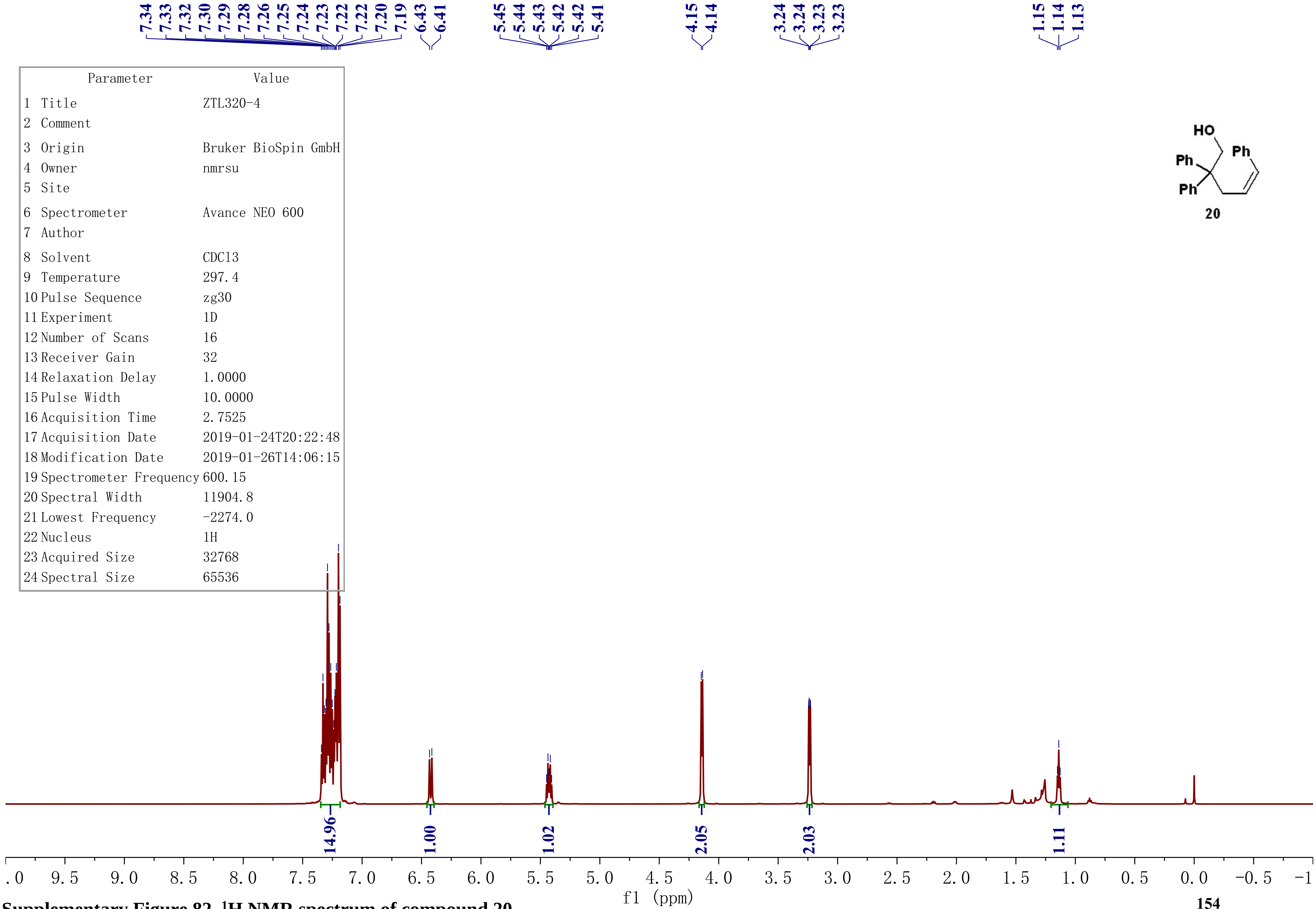

20

Supplementary Figure 82. ¹H NMR spectrum of compound 20

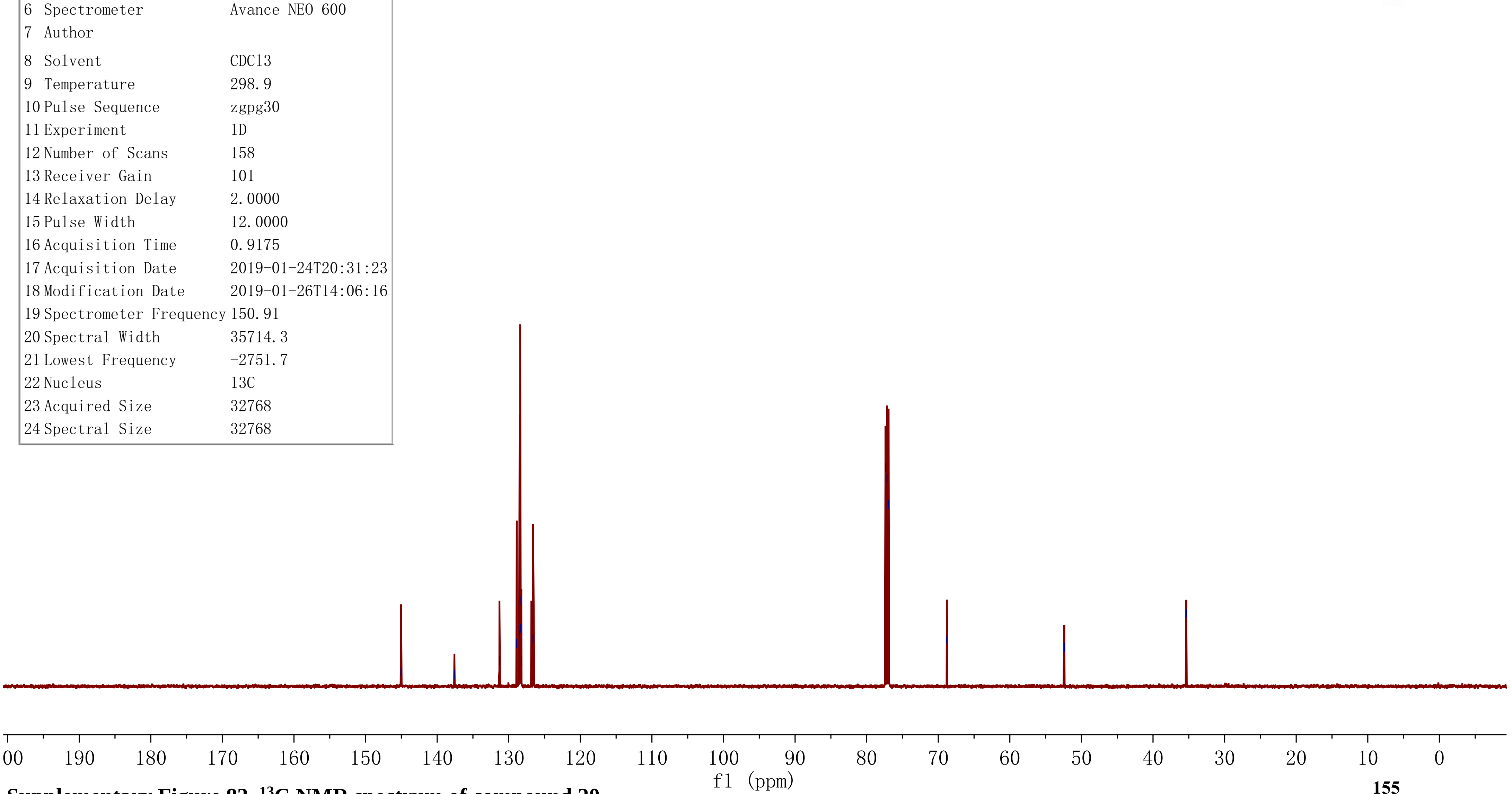

	Parameter	Value
1	Title	ZTL320-4
2	Comment	
3	Origin	Bruker BioSpin GmbH
4	Owner	nmrsu
5	Site	
6	Spectrometer	Avance NEO 600
7	Author	
8	Solvent	CDCl3
9	Temperature	298.9
10	Pulse Sequence	zgpg30
11	Experiment	1D
12	Number of Scans	158
13	Receiver Gain	101
14	Relaxation Delay	2.0000
15	Pulse Width	12.0000
16	Acquisition Time	0.9175
17	Acquisition Date	2019-01-24T20:31:23
18	Modification Date	2019-01-26T14:06:16
19	Spectrometer Frequency	150.91
20	Spectral Width	35714.3
21	Lowest Frequency	-2751.7
22	Nucleus	13C
23	Acquired Size	32768
24	Spectral Size	32768

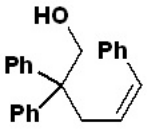

20

Supplementary Figure 83. ^{13}C NMR spectrum of compound 20

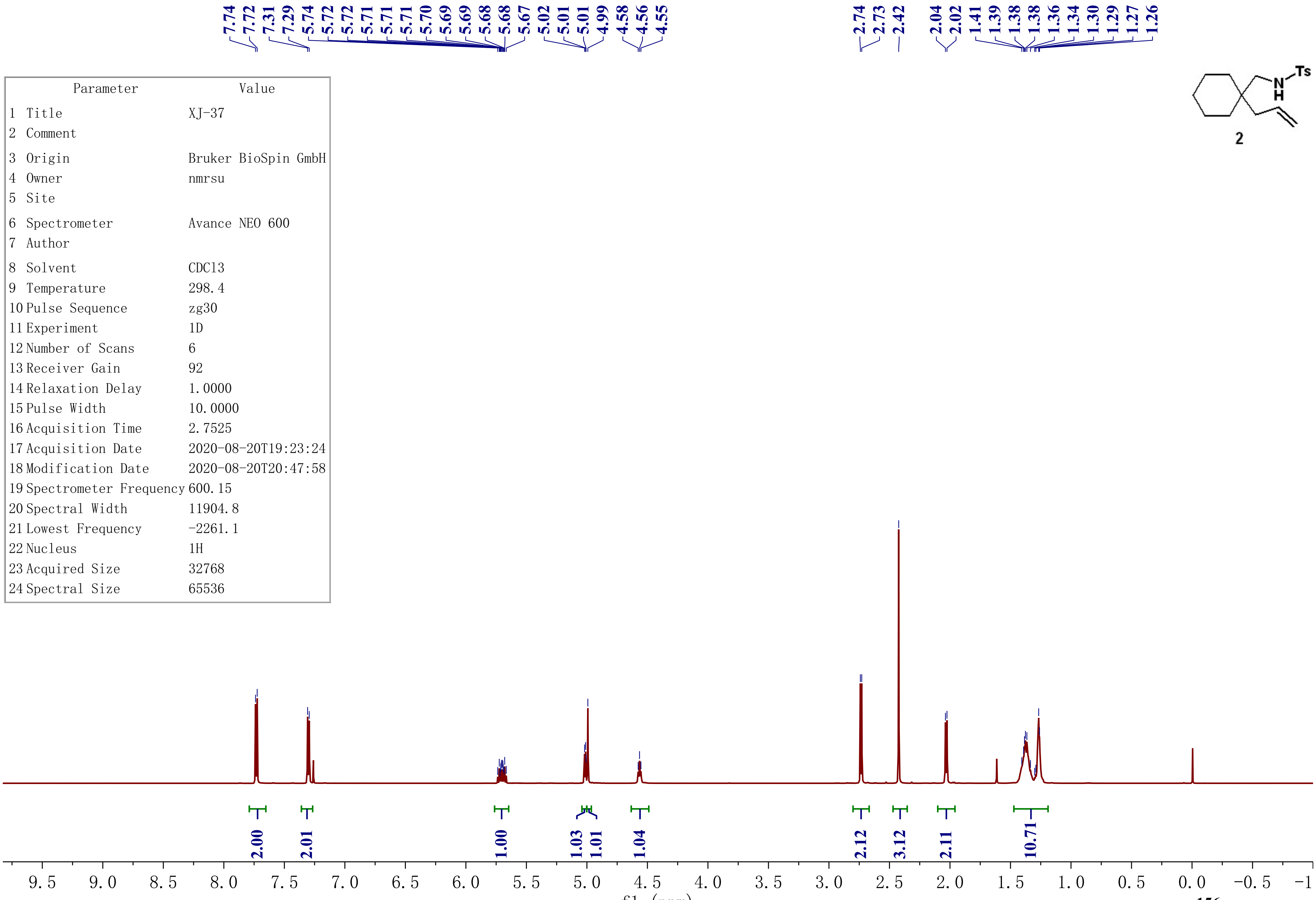

	Parameter	Value
1	Title	XJ-37
2	Comment	
3	Origin	Bruker BioSpin GmbH
4	Owner	nmrsu
5	Site	
6	Spectrometer	Avance NEO 600
7	Author	
8	Solvent	CDCl3
9	Temperature	298.4
10	Pulse Sequence	zg30
11	Experiment	1D
12	Number of Scans	6
13	Receiver Gain	92
14	Relaxation Delay	1.0000
15	Pulse Width	10.0000
16	Acquisition Time	2.7525
17	Acquisition Date	2020-08-20T19:23:24
18	Modification Date	2020-08-20T20:47:58
19	Spectrometer Frequency	600.15
20	Spectral Width	11904.8
21	Lowest Frequency	-2261.1
22	Nucleus	1H
23	Acquired Size	32768
24	Spectral Size	65536

Supplementary Figure 84. ^{1}H NMR spectrum of compound 2

Parameter	Value
1 Title	XJ-37
2 Comment	
3 Origin	Bruker BioSpin GmbH
4 Owner	nmrsu
5 Site	
6 Spectrometer	Avance NEO 600
7 Author	
8 Solvent	CDCl3
9 Temperature	299.1
10 Pulse Sequence	zgpg30
11 Experiment	1D
12 Number of Scans	107
13 Receiver Gain	101
14 Relaxation Delay	2.0000
15 Pulse Width	12.0000
16 Acquisition Time	0.9175
17 Acquisition Date	2020-08-20T19:29:58
18 Modification Date	2020-08-20T20:48:00
19 Spectrometer Frequency	150.91
20 Spectral Width	35714.3
21 Lowest Frequency	-2747.6
22 Nucleus	13C
23 Acquired Size	32768
24 Spectral Size	32768

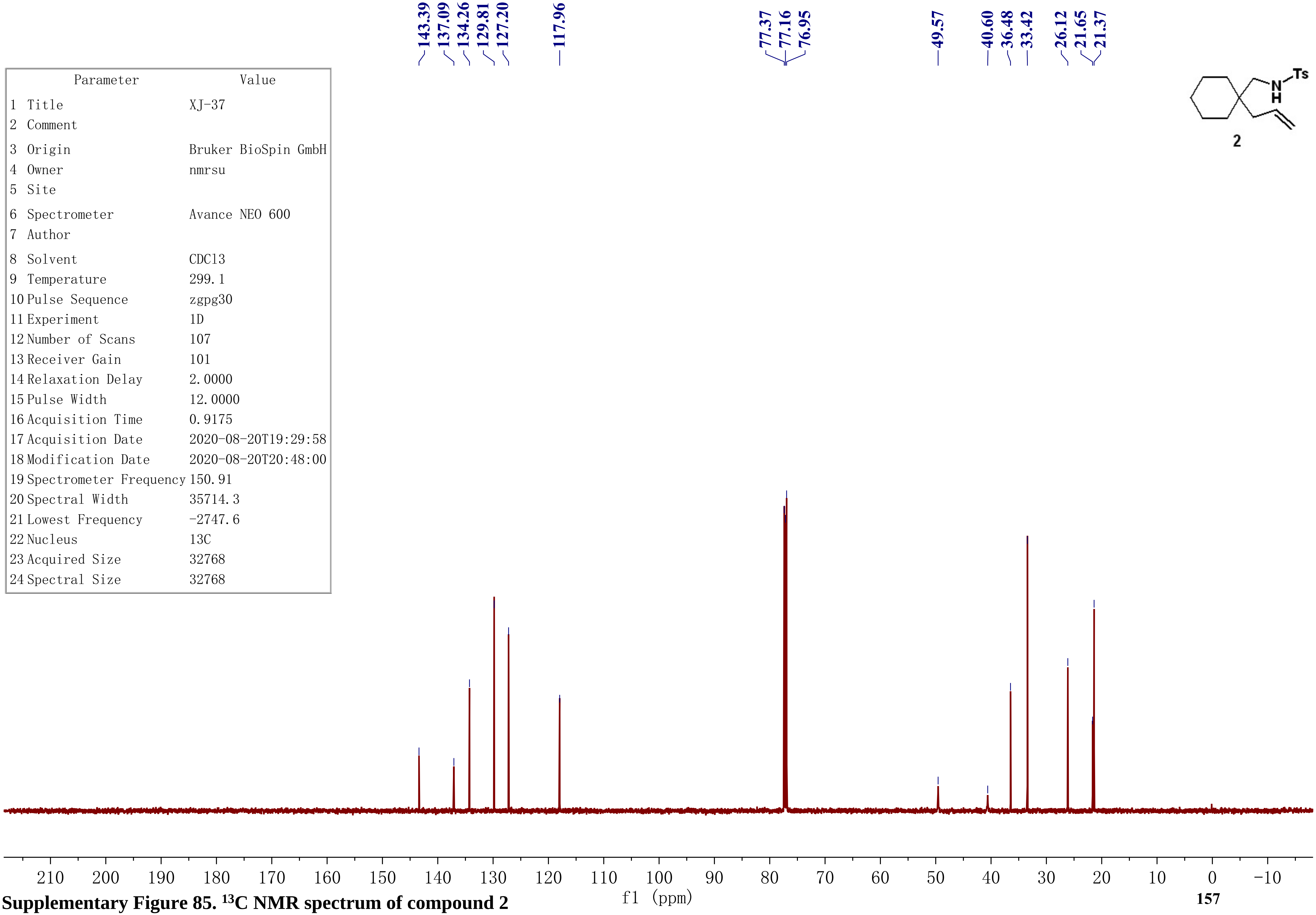

2

Supplementary Figure 85. ^{13}**C NMR spectrum of compound 2**

Parameter	Value
1 Title	XJ5-S21-1
2 Comment	
3 Origin	Bruker BioSpin GmbH
4 Owner	nmrsu
5 Site	
6 Spectrometer	Avance NEO 600
7 Author	
8 Solvent	CDCl3
9 Temperature	297.8
10 Pulse Sequence	zg30
11 Experiment	1D
12 Number of Scans	8
13 Receiver Gain	78
14 Relaxation Delay	1.0000
15 Pulse Width	10.0000
16 Acquisition Time	2.7525
17 Acquisition Date	2020-08-02T12:22:20
18 Modification Date	2020-08-02T20:41:00
19 Spectrometer Frequency	600.15
20 Spectral Width	11904.8
21 Lowest Frequency	-2260.9
22 Nucleus	1H
23 Acquired Size	32768
24 Spectral Size	65536

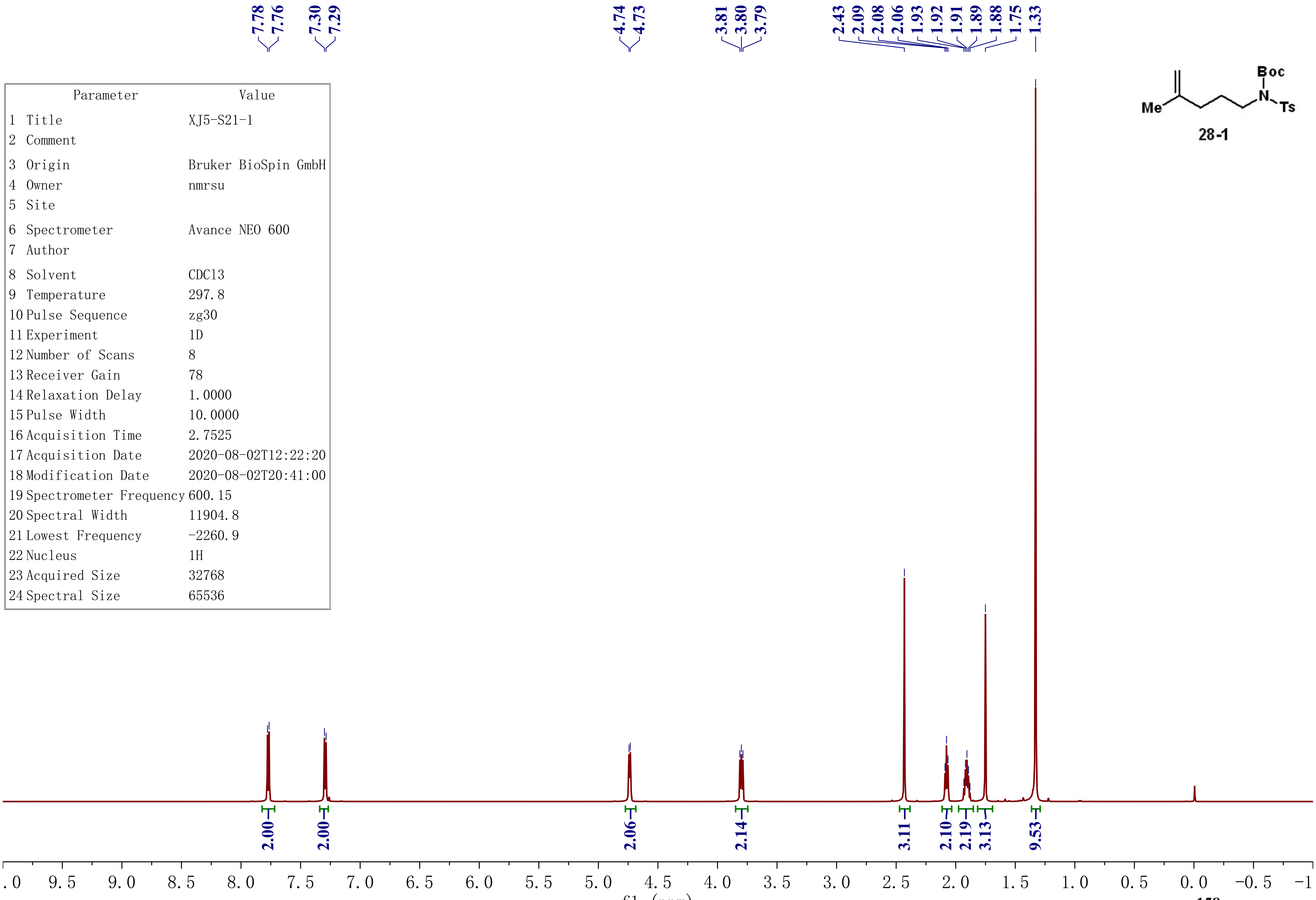

Supplementary Figure 86. ^{1}H NMR spectrum of compound 28-1

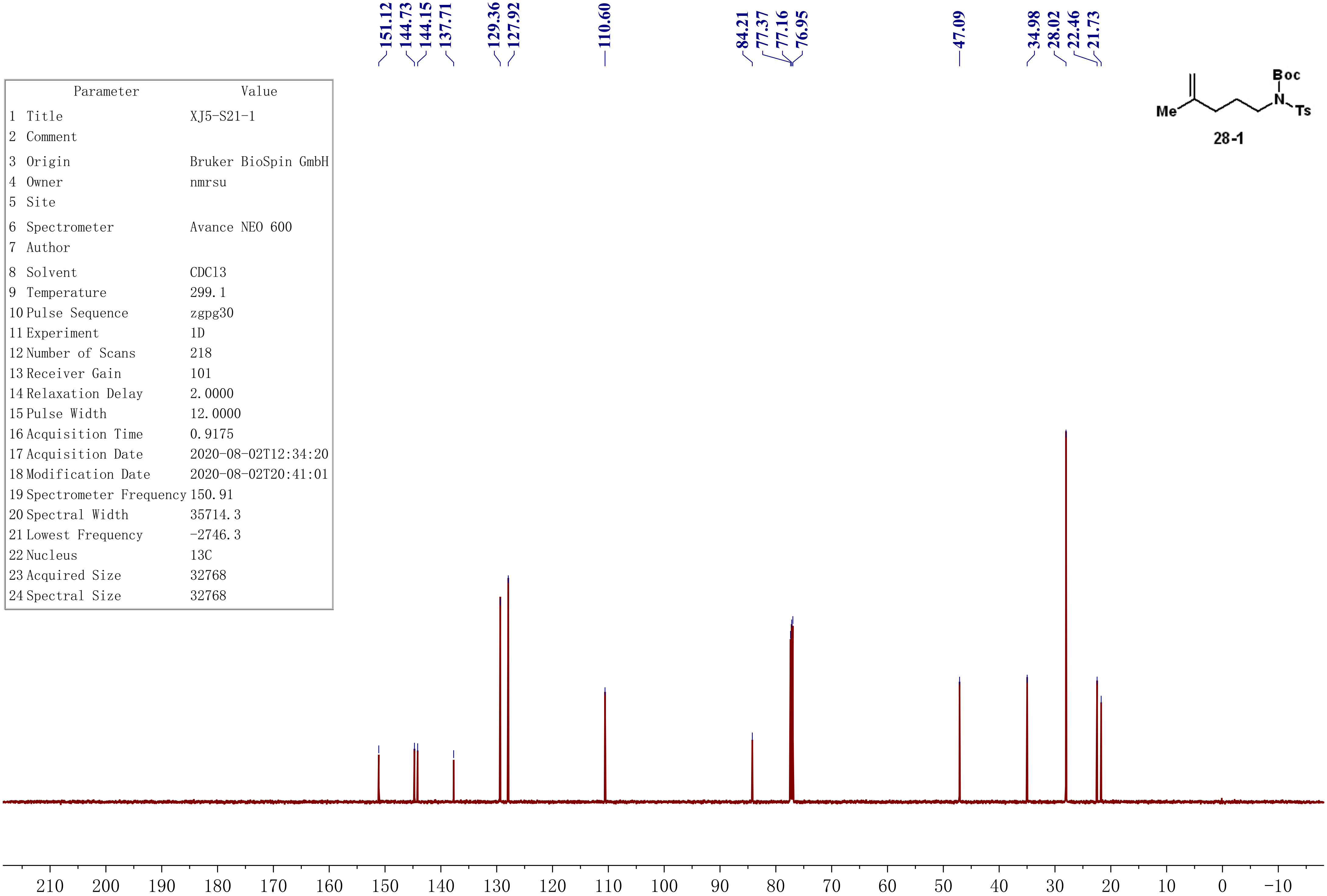

	Parameter	Value
1	Title	XJ5-S21-1
2	Comment	
3	Origin	Bruker BioSpin GmbH
4	Owner	nmrsu
5	Site	
6	Spectrometer	Avance NEO 600
7	Author	
8	Solvent	CDCl3
9	Temperature	299.1
10	Pulse Sequence	zgpg30
11	Experiment	1D
12	Number of Scans	218
13	Receiver Gain	101
14	Relaxation Delay	2.0000
15	Pulse Width	12.0000
16	Acquisition Time	0.9175
17	Acquisition Date	2020-08-02T12:34:20
18	Modification Date	2020-08-02T20:41:01
19	Spectrometer Frequency	150.91
20	Spectral Width	35714.3
21	Lowest Frequency	-2746.3
22	Nucleus	13C
23	Acquired Size	32768
24	Spectral Size	32768

Supplementary Figure 87. ^{13}C NMR spectrum of compound 28-1

Parameter	Value
1 Title	XJ2-16
2 Comment	
3 Origin	Bruker BioSpin GmbH
4 Owner	nmrsu
5 Site	
6 Spectrometer	Avance NEO 600
7 Author	
8 Solvent	CDCl3
9 Temperature	296.8
10 Pulse Sequence	zg30
11 Experiment	1D
12 Number of Scans	11
13 Receiver Gain	71
14 Relaxation Delay	1.0000
15 Pulse Width	10.0000
16 Acquisition Time	2.7525
17 Acquisition Date	2018-11-09T12:45:04
18 Modification Date	2018-11-09T12:55:19
19 Spectrometer Frequency	600.15
20 Spectral Width	11904.8
21 Lowest Frequency	-2260.8
22 Nucleus	1H
23 Acquired Size	32768
24 Spectral Size	65536

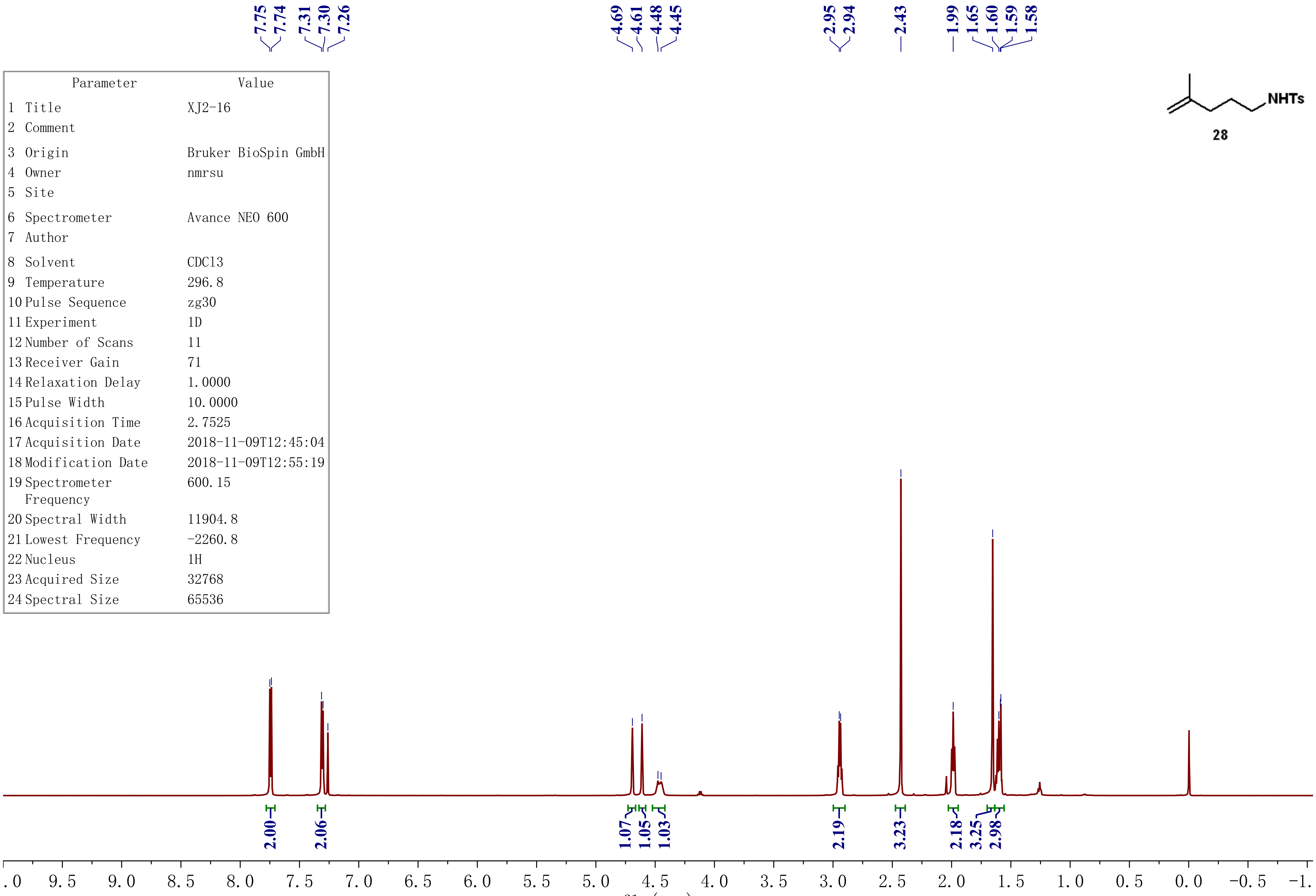

Supplementary Figure 88. ^{1}H NMR spectrum of compound 28

	Parameter	Value
1	Title	XJ2-16
2	Comment	
3	Origin	Bruker BioSpin GmbH
4	Owner	nmrsu
5	Site	
6	Spectrometer	Avance NEO 600
7	Author	
8	Solvent	CDCl3
9	Temperature	298.1
10	Pulse Sequence	zgpg30
11	Experiment	1D
12	Number of Scans	105
13	Receiver Gain	101
14	Relaxation Delay	2.0000
15	Pulse Width	12.0000
16	Acquisition Time	0.9175
17	Acquisition Date	2018-11-09T12:51:11
18	Modification Date	2018-11-09T12:55:20
19	Spectrometer Frequency	150.91
20	Spectral Width	35714.3
21	Lowest Frequency	-2747.0
22	Nucleus	13C
23	Acquired Size	32768
24	Spectral Size	32768

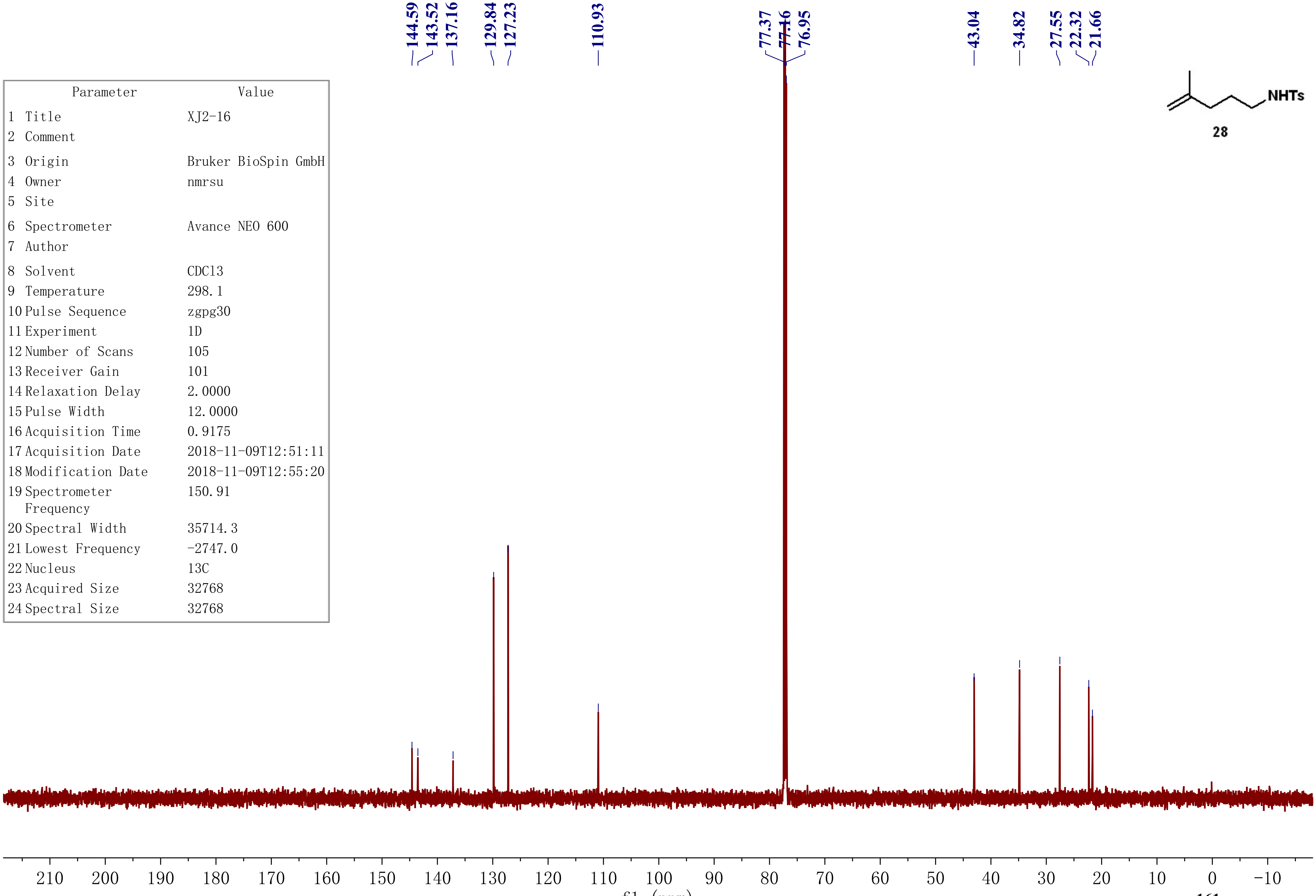

28

Supplementary Figure 89. ^{13}C NMR spectrum of compound 28

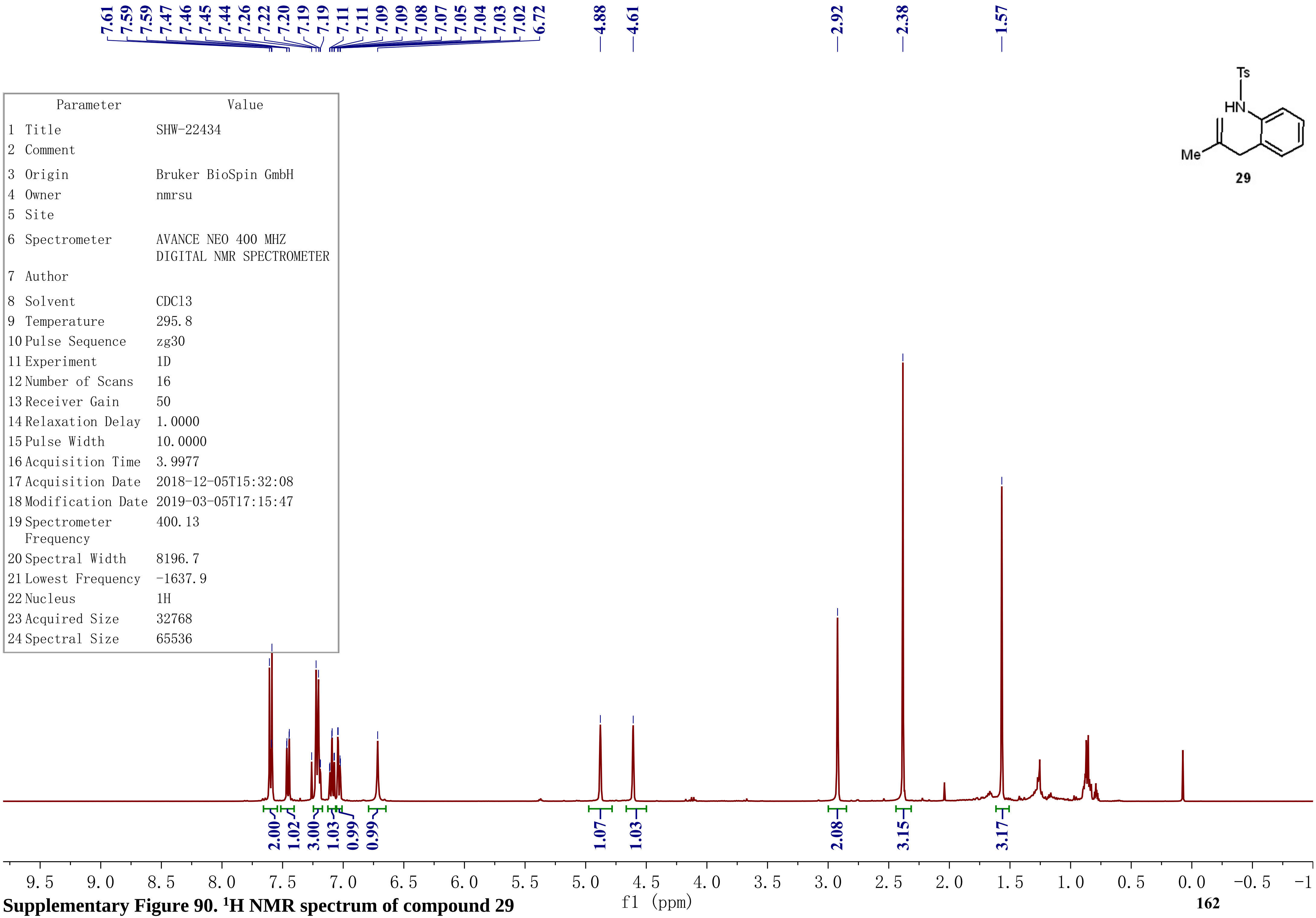

Parameter	Value
1 Title	SHW-22434
2 Comment	
3 Origin	Bruker BioSpin GmbH
4 Owner	nmrsu
5 Site	
6 Spectrometer	AVANCE NEO 400 MHZ DIGITAL NMR SPECTROMETER
7 Author	
8 Solvent	CDCl3
9 Temperature	295.8
10 Pulse Sequence	zg30
11 Experiment	1D
12 Number of Scans	16
13 Receiver Gain	50
14 Relaxation Delay	1.0000
15 Pulse Width	10.0000
16 Acquisition Time	3.9977
17 Acquisition Date	2018-12-05T15:32:08
18 Modification Date	2019-03-05T17:15:47
19 Spectrometer Frequency	400.13
20 Spectral Width	8196.7
21 Lowest Frequency	-1637.9
22 Nucleus	1H
23 Acquired Size	32768
24 Spectral Size	65536

29

Supplementary Figure 90. ^{1}H NMR spectrum of compound 29

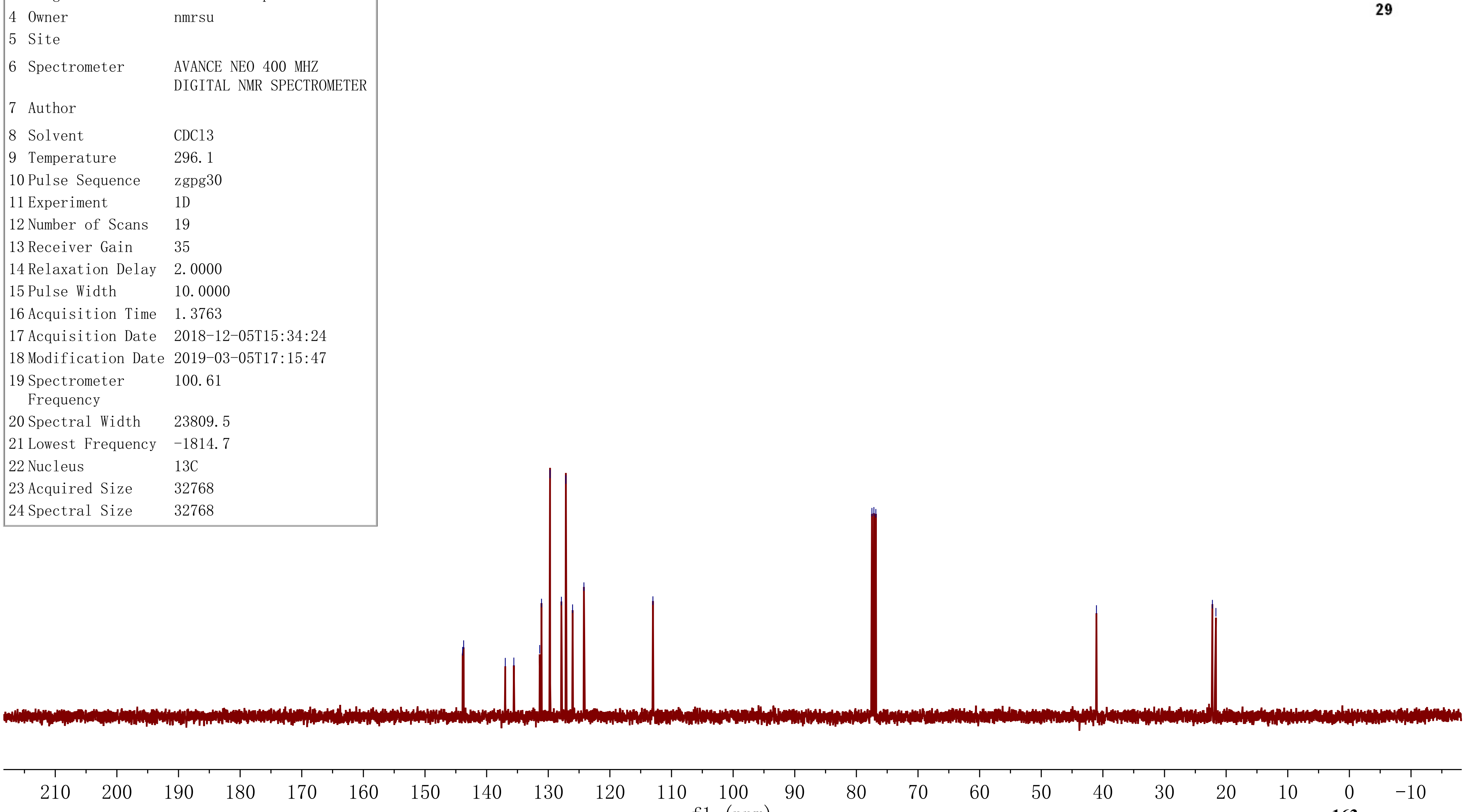

	Parameter	Value
1	Title	SHW-22434
2	Comment	
3	Origin	Bruker BioSpin GmbH
4	Owner	nmrsu
5	Site	
6	Spectrometer	AVANCE NEO 400 MHZ DIGITAL NMR SPECTROMETER
7	Author	
8	Solvent	CDCl3
9	Temperature	296.1
10	Pulse Sequence	zgpg30
11	Experiment	1D
12	Number of Scans	19
13	Receiver Gain	35
14	Relaxation Delay	2.0000
15	Pulse Width	10.0000
16	Acquisition Time	1.3763
17	Acquisition Date	2018-12-05T15:34:24
18	Modification Date	2019-03-05T17:15:47
19	Spectrometer Frequency	100.61
20	Spectral Width	23809.5
21	Lowest Frequency	-1814.7
22	Nucleus	13C
23	Acquired Size	32768
24	Spectral Size	32768

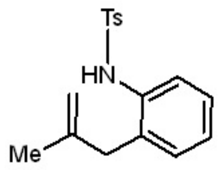

29

Supplementary Figure 91. ^{13}C NMR spectrum of compound 29

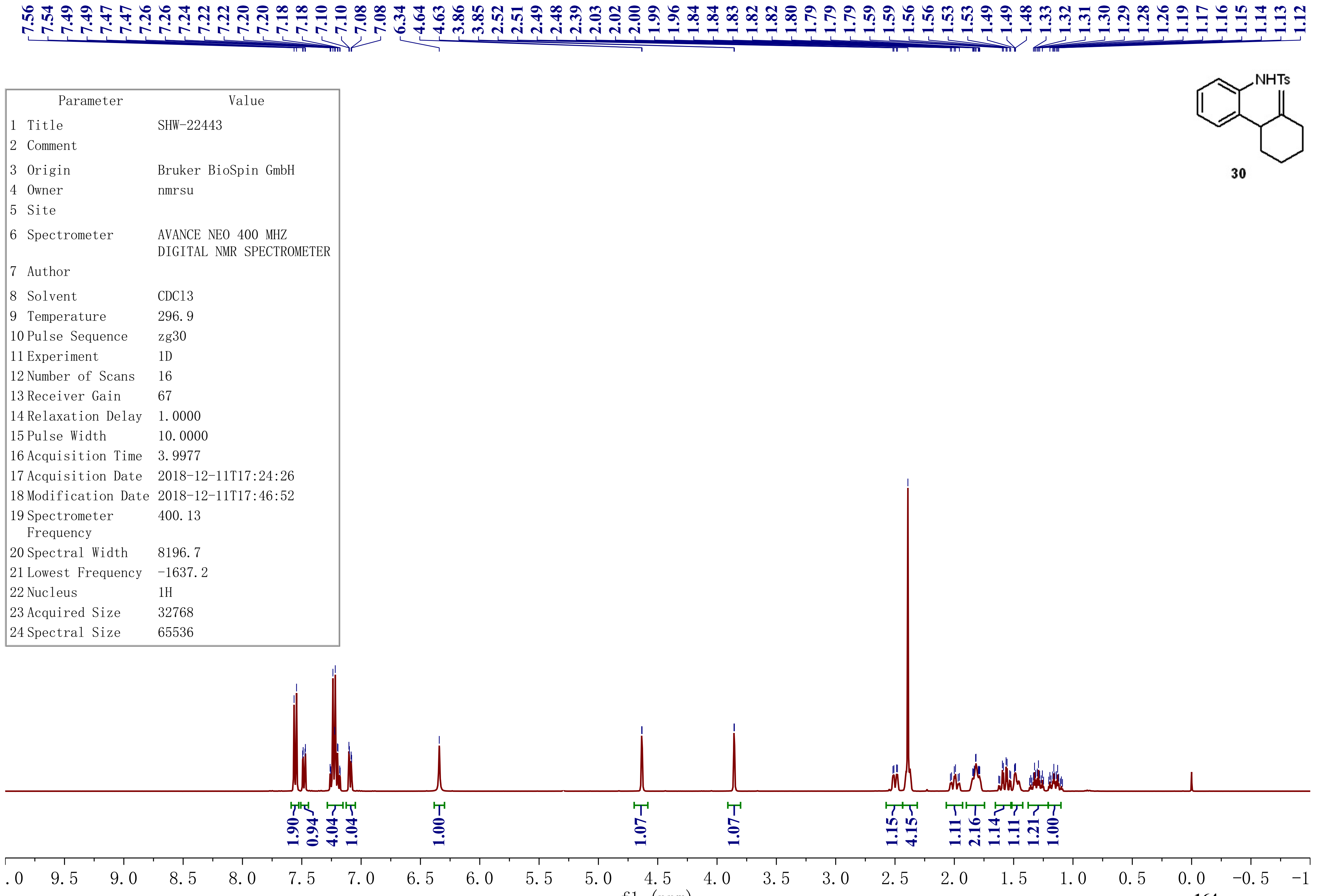

	Parameter	Value
1	Title	SHW-22443
2	Comment	
3	Origin	Bruker BioSpin GmbH
4	Owner	nmrsu
5	Site	
6	Spectrometer	AVANCE NEO 400 MHZ DIGITAL NMR SPECTROMETER
7	Author	
8	Solvent	CDCl3
9	Temperature	296.9
10	Pulse Sequence	zg30
11	Experiment	1D
12	Number of Scans	16
13	Receiver Gain	67
14	Relaxation Delay	1.0000
15	Pulse Width	10.0000
16	Acquisition Time	3.9977
17	Acquisition Date	2018-12-11T17:24:26
18	Modification Date	2018-12-11T17:46:52
19	Spectrometer Frequency	400.13
20	Spectral Width	8196.7
21	Lowest Frequency	-1637.2
22	Nucleus	1H
23	Acquired Size	32768
24	Spectral Size	65536

Supplementary Figure 92. ^{1}H NMR spectrum of compound 30

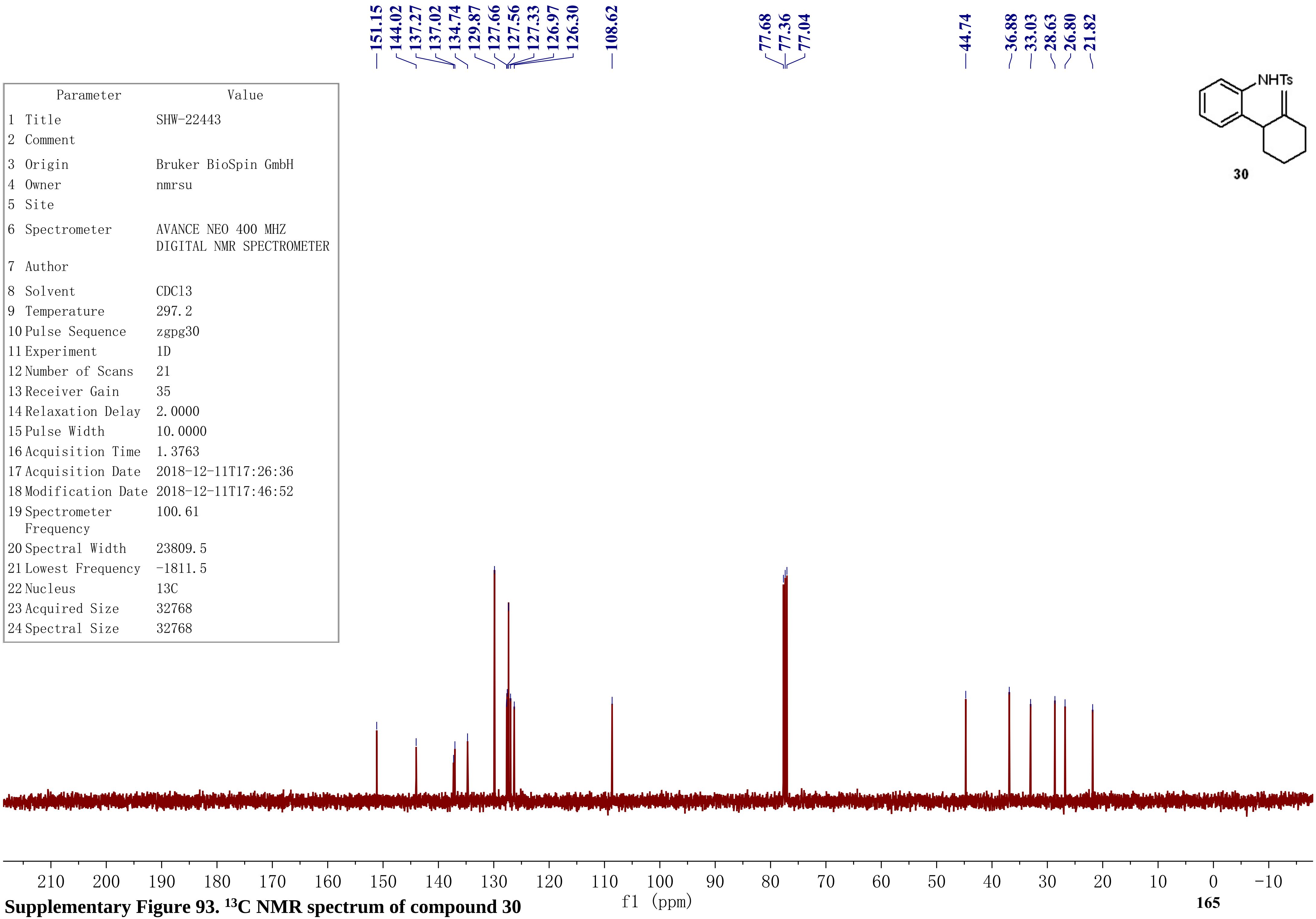

	Parameter	Value
1	Title	SHW-22443
2	Comment	
3	Origin	Bruker BioSpin GmbH
4	Owner	nmrsu
5	Site	
6	Spectrometer	AVANCE NEO 400 MHZ DIGITAL NMR SPECTROMETER
7	Author	
8	Solvent	CDCl3
9	Temperature	297.2
10	Pulse Sequence	zgpg30
11	Experiment	1D
12	Number of Scans	21
13	Receiver Gain	35
14	Relaxation Delay	2.0000
15	Pulse Width	10.0000
16	Acquisition Time	1.3763
17	Acquisition Date	2018-12-11T17:26:36
18	Modification Date	2018-12-11T17:46:52
19	Spectrometer Frequency	100.61
20	Spectral Width	23809.5
21	Lowest Frequency	-1811.5
22	Nucleus	13C
23	Acquired Size	32768
24	Spectral Size	32768

30

Supplementary Figure 93. ^{13}C NMR spectrum of compound 30

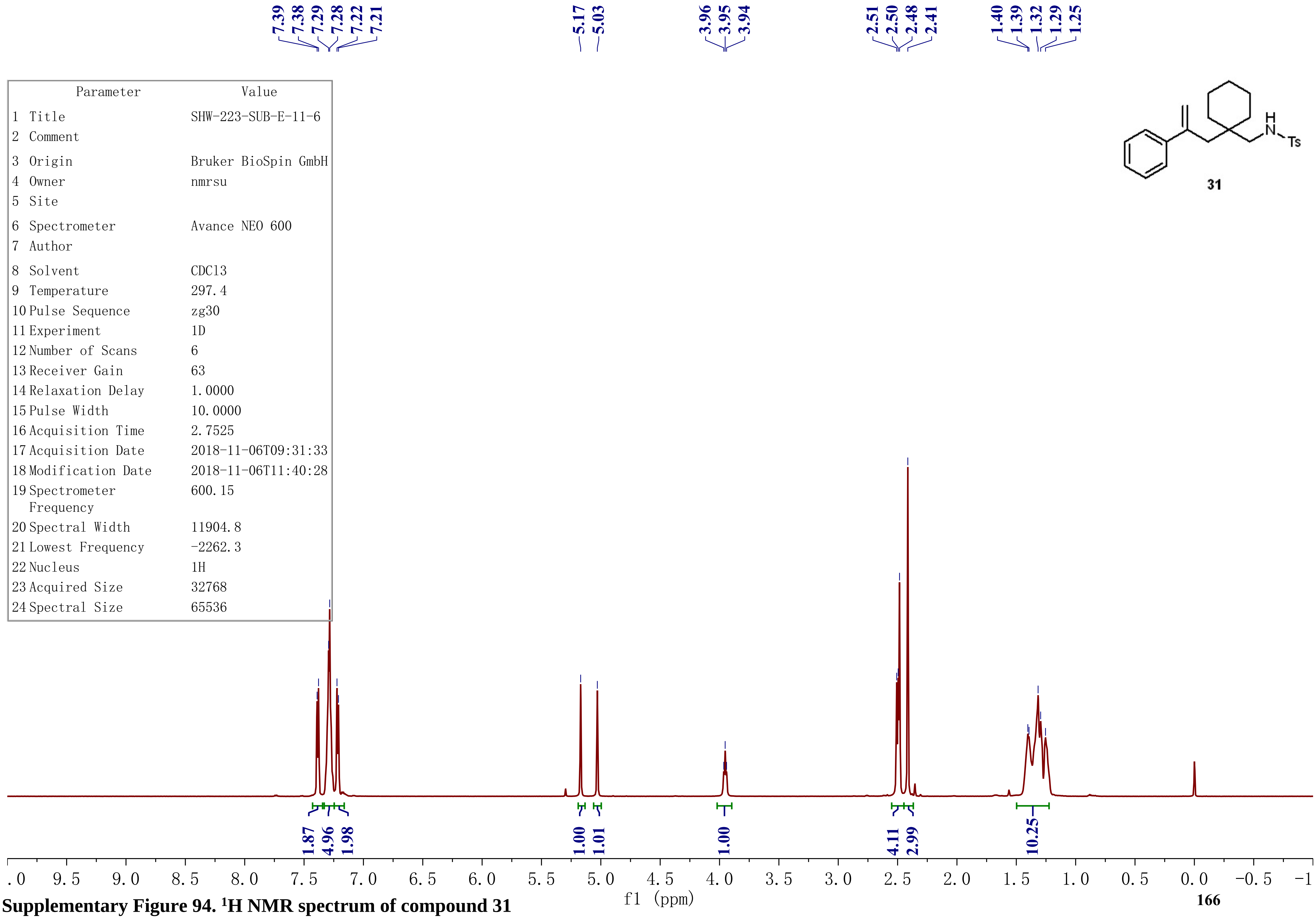

Supplementary Figure 94. ^{1}H NMR spectrum of compound 31

	Parameter	Value
1	Title	SHW-223-SUB-E-11-6
2	Comment	
3	Origin	Bruker BioSpin GmbH
4	Owner	nmrsu
5	Site	
6	Spectrometer	Avance NEO 600
7	Author	
8	Solvent	CDCl3
9	Temperature	297.8
10	Pulse Sequence	zgpg30
11	Experiment	1D
12	Number of Scans	65
13	Receiver Gain	101
14	Relaxation Delay	2.0000
15	Pulse Width	12.0000
16	Acquisition Time	0.9175
17	Acquisition Date	2018-11-06T09:35:42
18	Modification Date	2018-11-06T11:40:29
19	Spectrometer Frequency	150.91
20	Spectral Width	35714.3
21	Lowest Frequency	-2715.3
22	Nucleus	13C
23	Acquired Size	32768
24	Spectral Size	32768

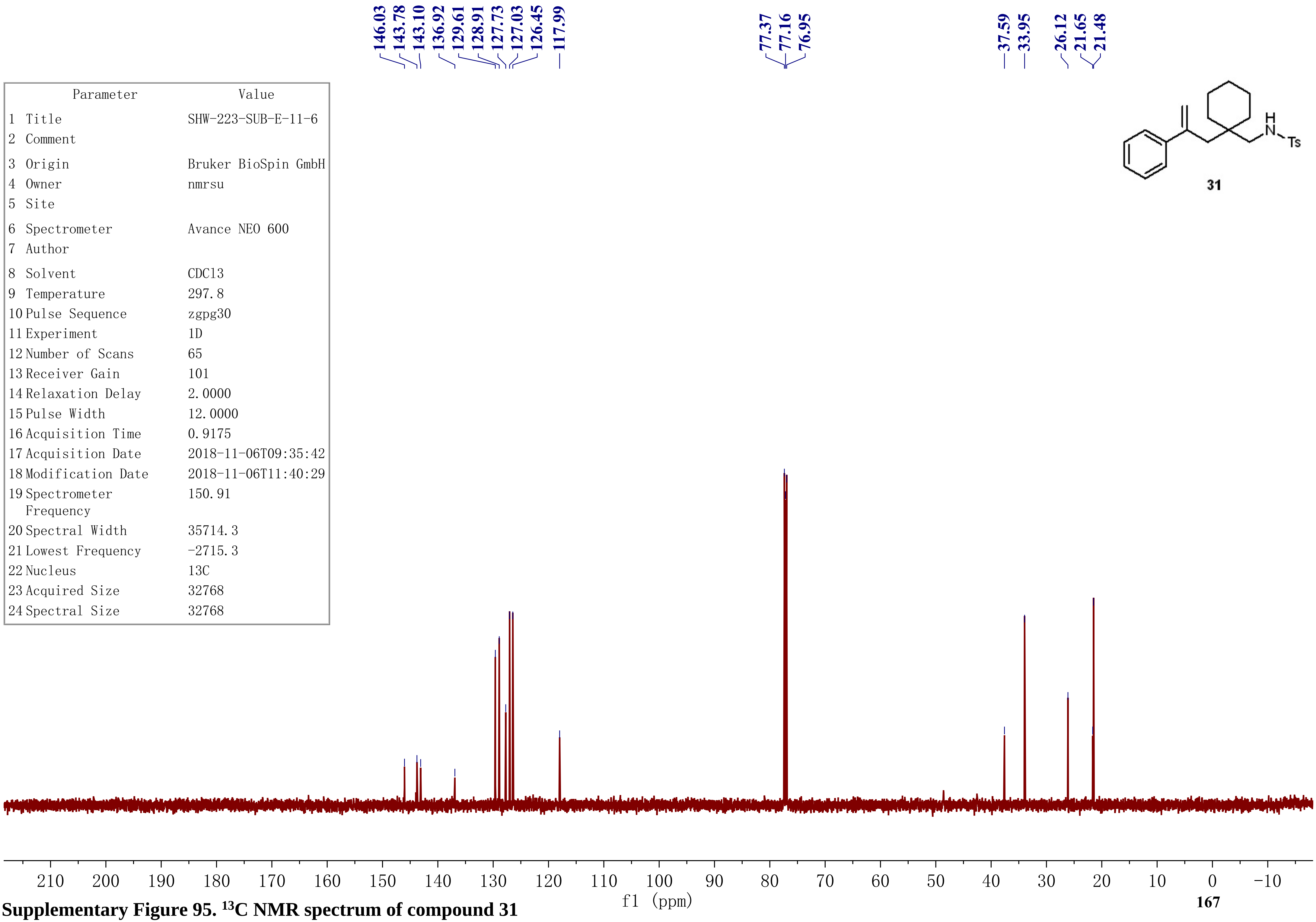

31

Supplementary Figure 95. ^{13}C NMR spectrum of compound 31

Parameter	Value
1 Title	SHW-22340
2 Comment	
3 Origin	Bruker BioSpin GmbH
4 Owner	nmrsu
5 Site	
6 Spectrometer	AVANCE NEO 400 MHZ DIGITAL NMR SPECTROMETER
7 Author	
8 Solvent	CDCl3
9 Temperature	297.4
10 Pulse Sequence	zg30
11 Experiment	1D
12 Number of Scans	13
13 Receiver Gain	101
14 Relaxation Delay	1.0000
15 Pulse Width	10.0000
16 Acquisition Time	3.9977
17 Acquisition Date	2018-12-10T14:00:53
18 Modification Date	2018-12-21T21:09:24
19 Spectrometer Frequency	400.13
20 Spectral Width	8196.7
21 Lowest Frequency	-1638.0
22 Nucleus	1H
23 Acquired Size	32768
24 Spectral Size	65536

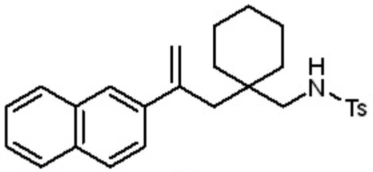

32

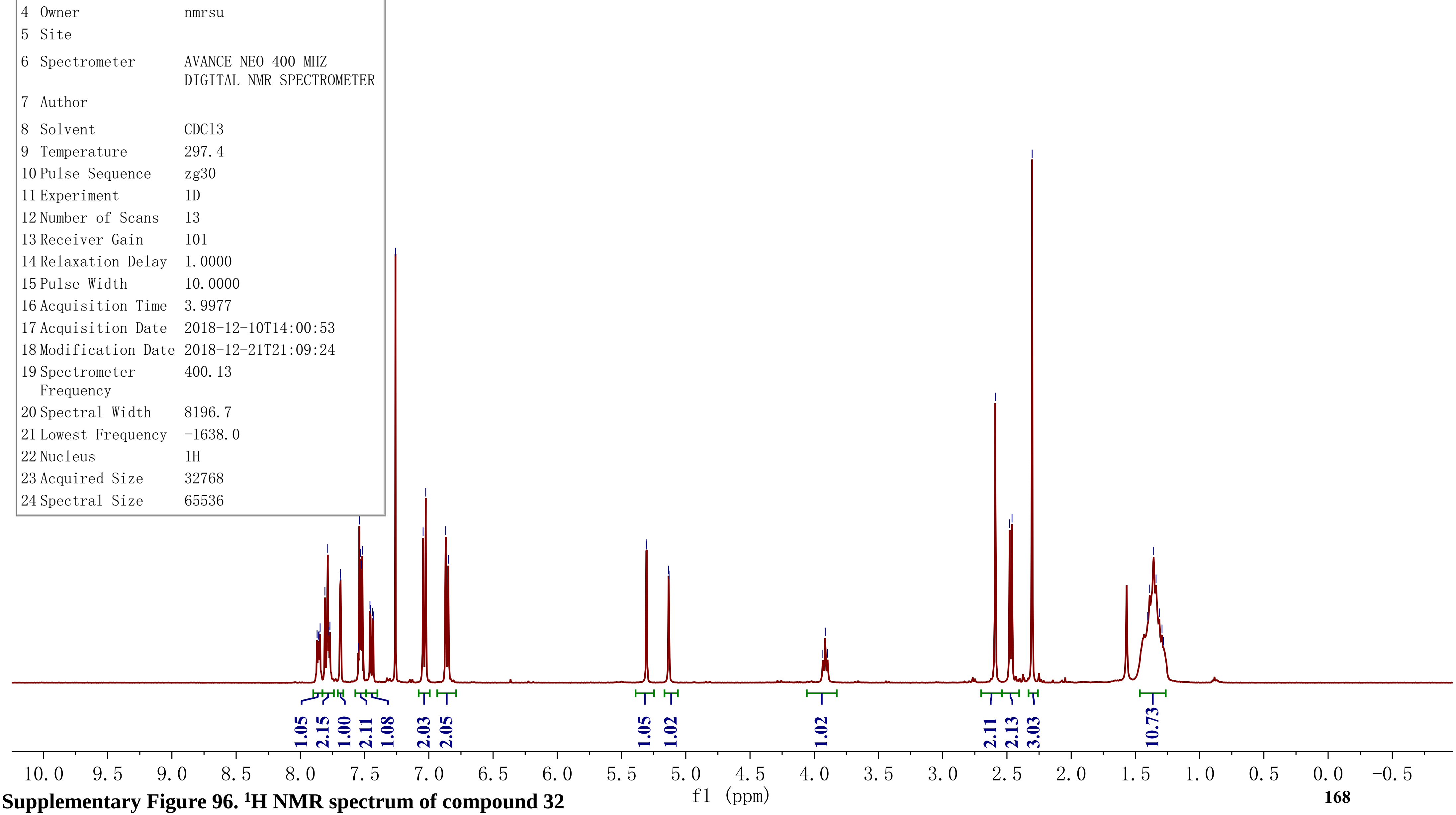

Supplementary Figure 96. ^{1}H NMR spectrum of compound 32

Parameter	Value
1 Title	SHW-22440-C
2 Comment	
3 Origin	Bruker BioSpin GmbH
4 Owner	nmrsu
5 Site	
6 Spectrometer	AVANCE NEO 400 MHZ DIGITAL NMR SPECTROMETER
7 Author	
8 Solvent	CDCl3
9 Temperature	296.0
10 Pulse Sequence	zgpg30
11 Experiment	1D
12 Number of Scans	69
13 Receiver Gain	29
14 Relaxation Delay	2.0000
15 Pulse Width	10.0000
16 Acquisition Time	1.3763
17 Acquisition Date	2018-12-21T22:06:24
18 Modification Date	2018-12-21T22:28:38
19 Spectrometer Frequency	100.61
20 Spectral Width	23809.5
21 Lowest Frequency	-1813.1
22 Nucleus	13C
23 Acquired Size	32768
24 Spectral Size	32768

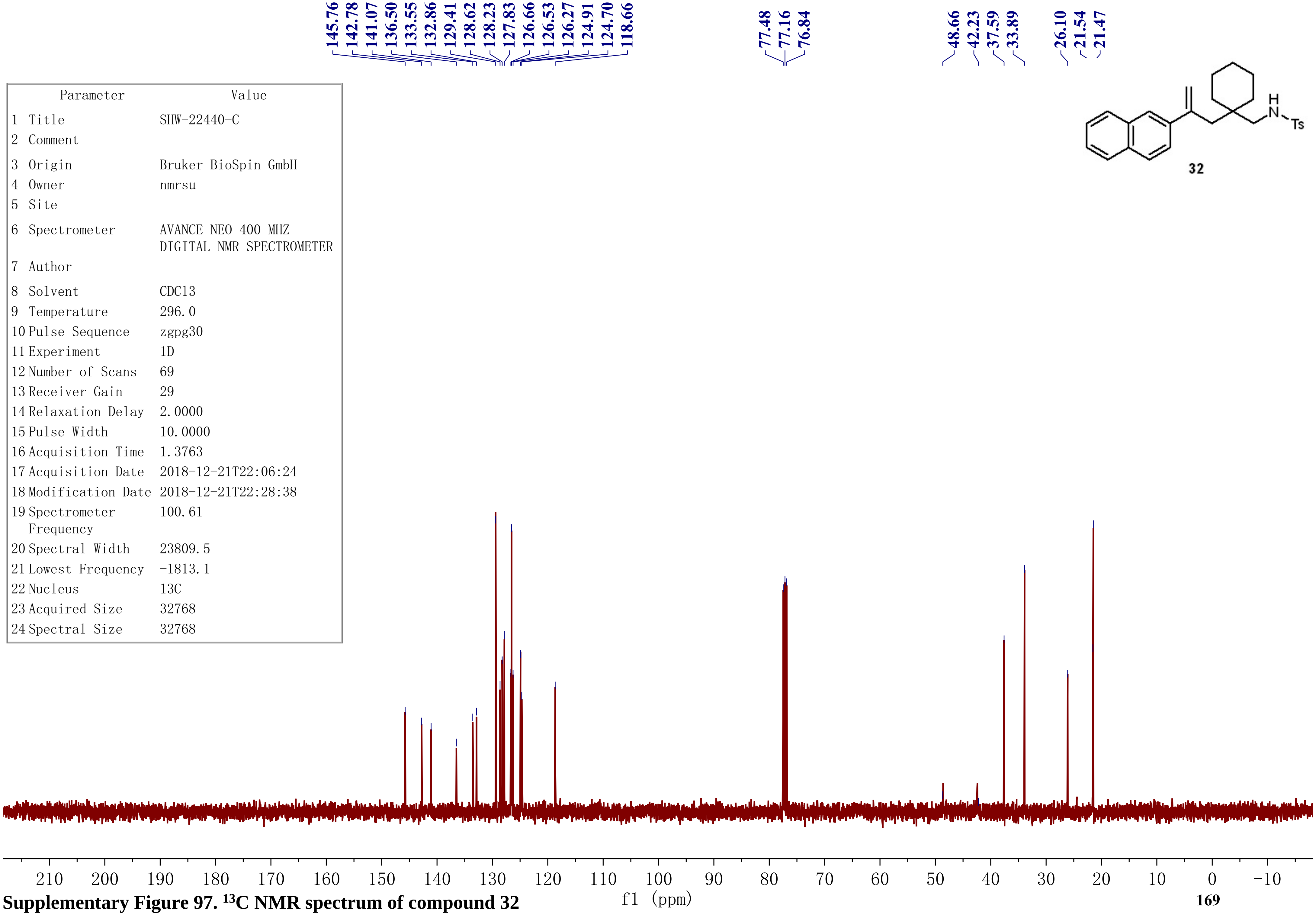

Supplementary Figure 97. ^{13}C NMR spectrum of compound 32

Parameter	Value
1 Title	XJ5-179
2 Comment	
3 Origin	Bruker BioSpin GmbH
4 Owner	nmrsu
5 Site	
6 Spectrometer	Avance NEO 600
7 Author	
8 Solvent	CDCl3
9 Temperature	297.8
10 Pulse Sequence	zg30
11 Experiment	1D
12 Number of Scans	8
13 Receiver Gain	101
14 Relaxation Delay	1.0000
15 Pulse Width	10.0000
16 Acquisition Time	2.7525
17 Acquisition Date	2020-08-03T14:01:44
18 Modification Date	2020-08-04T22:08:00
19 Spectrometer Frequency	600.15
20 Spectral Width	11904.8
21 Lowest Frequency	-2261.1
22 Nucleus	1H
23 Acquired Size	32768
24 Spectral Size	65536

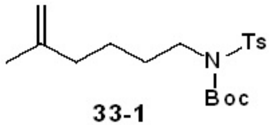

33-1

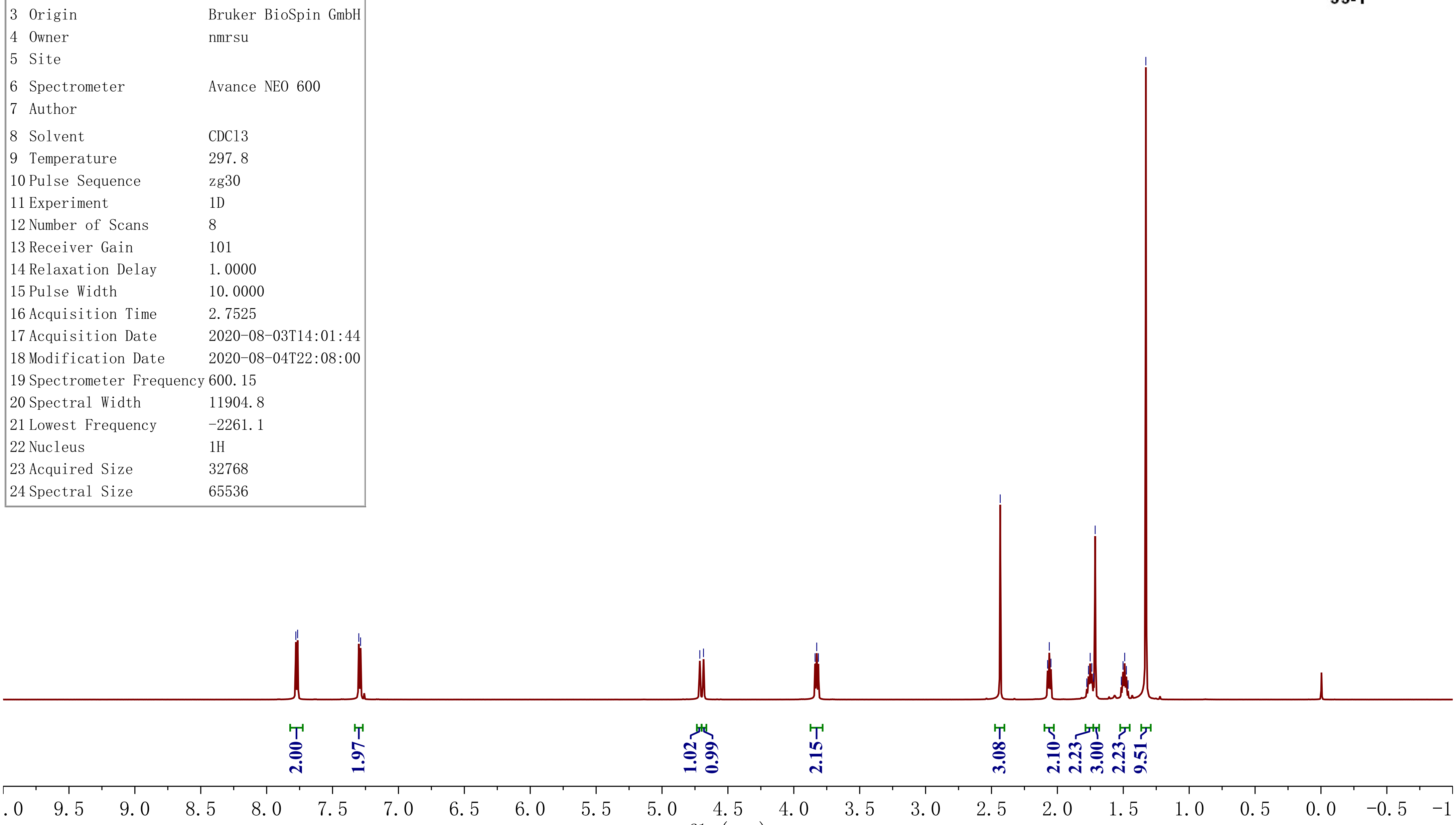

Supplementary Figure 98. ^{1}H NMR spectrum of compound 33-1

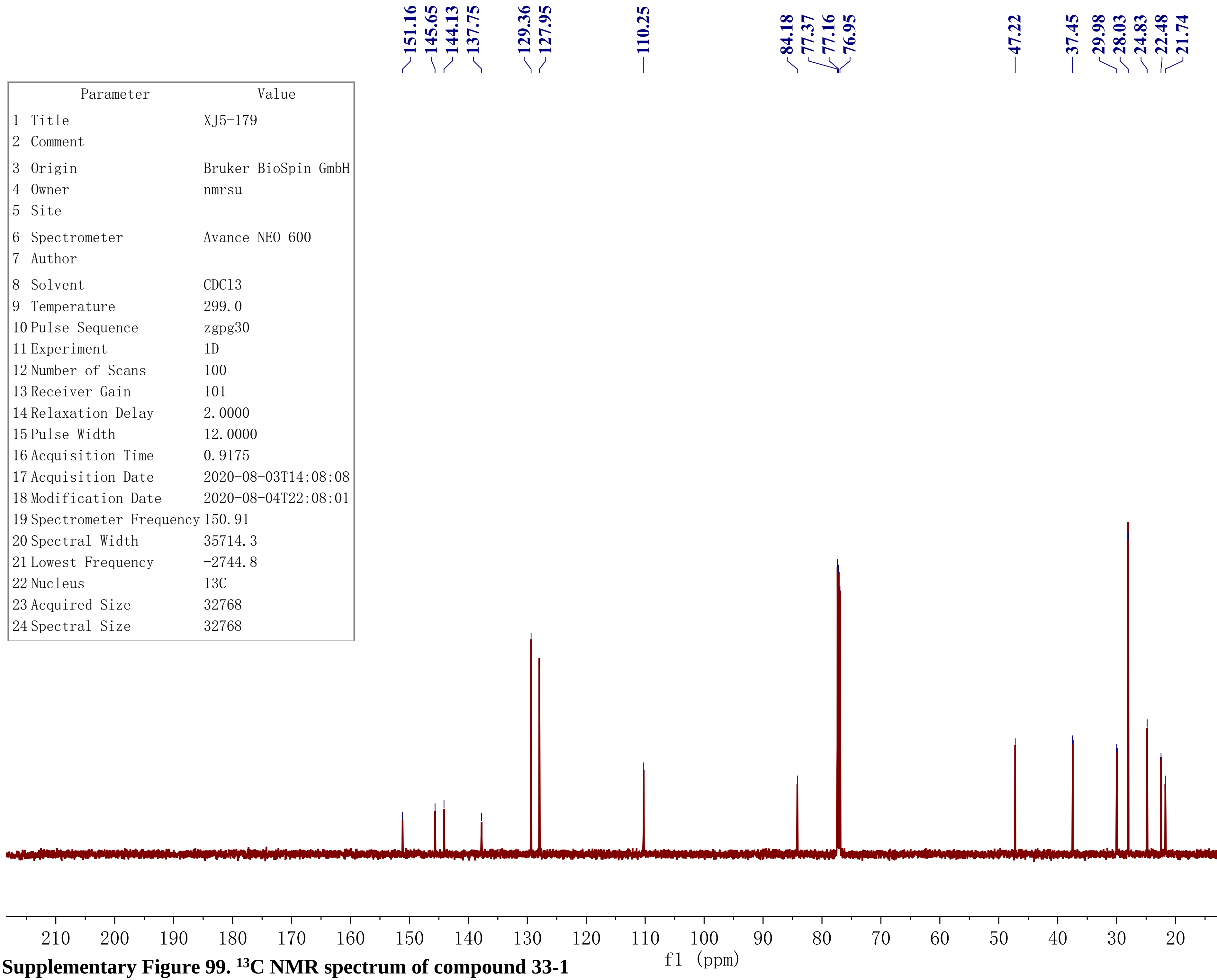

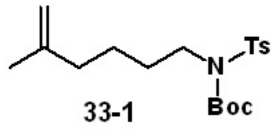

Parameter	Value
1 Title	XJ5-179
2 Comment	
3 Origin	Bruker BioSpin GmbH
4 Owner	nmrsu
5 Site	
6 Spectrometer	Avance NEO 600
7 Author	
8 Solvent	CDCl3
9 Temperature	299.0
10 Pulse Sequence	zgpg30
11 Experiment	1D
12 Number of Scans	100
13 Receiver Gain	101
14 Relaxation Delay	2.0000
15 Pulse Width	12.0000
16 Acquisition Time	0.9175
17 Acquisition Date	2020-08-03T14:08:08
18 Modification Date	2020-08-04T22:08:01
19 Spectrometer Frequency	150.91
20 Spectral Width	35714.3
21 Lowest Frequency	-2744.8
22 Nucleus	13C
23 Acquired Size	32768
24 Spectral Size	32768

Supplementary Figure 99. ^{13}C NMR spectrum of compound 33-1

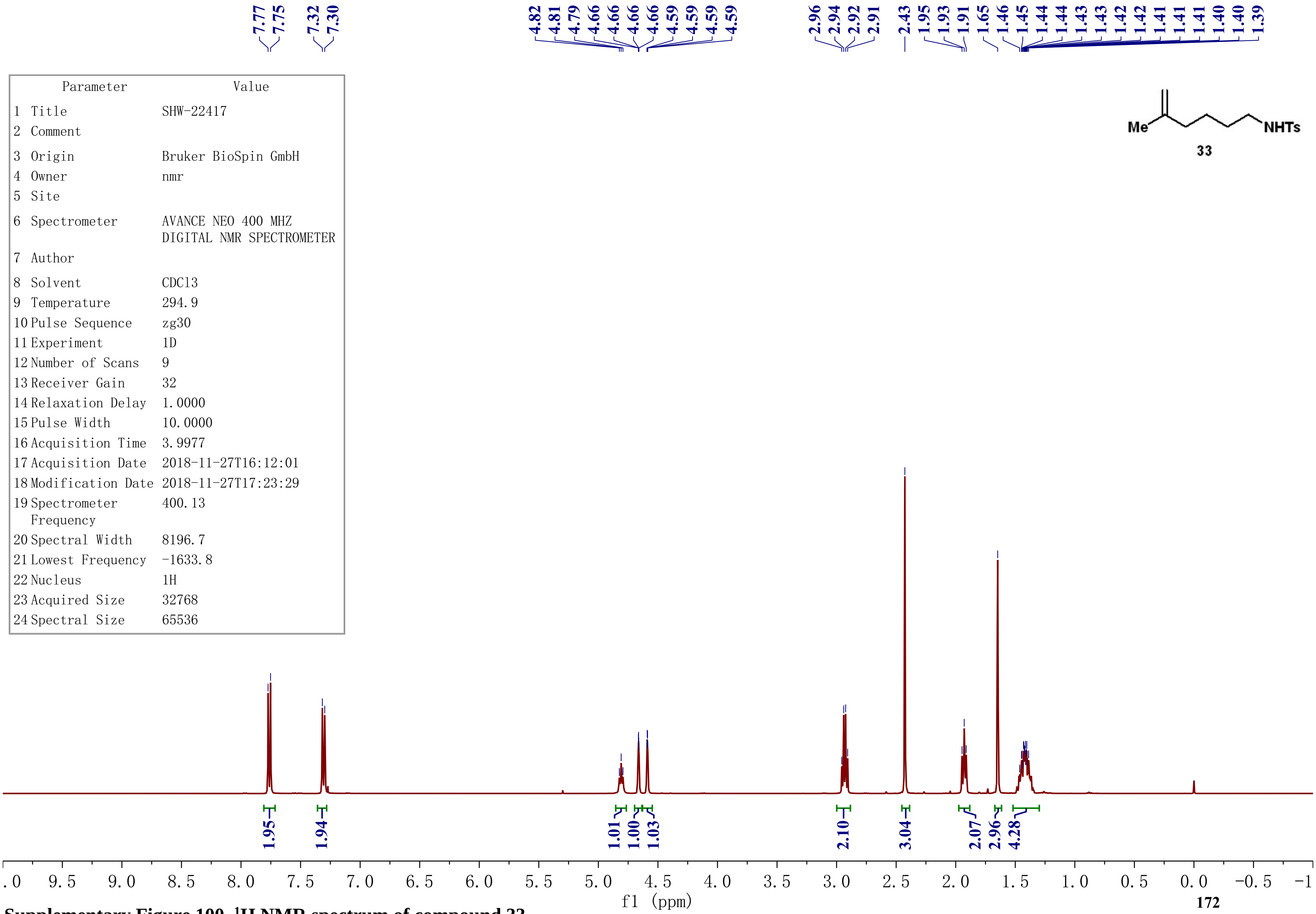

Parameter	Value
1 Title	SHW-22417
2 Comment	
3 Origin	Bruker BioSpin GmbH
4 Owner	nmr
5 Site	
6 Spectrometer	AVANCE NEO 400 MHZ DIGITAL NMR SPECTROMETER
7 Author	
8 Solvent	CDCl3
9 Temperature	294.9
10 Pulse Sequence	zg30
11 Experiment	1D
12 Number of Scans	9
13 Receiver Gain	32
14 Relaxation Delay	1.0000
15 Pulse Width	10.0000
16 Acquisition Time	3.9977
17 Acquisition Date	2018-11-27T16:12:01
18 Modification Date	2018-11-27T17:23:29
19 Spectrometer Frequency	400.13
20 Spectral Width	8196.7
21 Lowest Frequency	-1633.8
22 Nucleus	1H
23 Acquired Size	32768
24 Spectral Size	65536

Supplementary Figure 100. ^{1}H NMR spectrum of compound 33

Parameter	Value
1 Title	SHW-22417-C
2 Comment	
3 Origin	Bruker BioSpin GmbH
4 Owner	nmr
5 Site	
6 Spectrometer	AVANCE NEO 400 MHZ DIGITAL NMR SPECTROMETER
7 Author	
8 Solvent	CDCl3
9 Temperature	295.1
10 Pulse Sequence	zgpg30
11 Experiment	1D
12 Number of Scans	11
13 Receiver Gain	32
14 Relaxation Delay	2.0000
15 Pulse Width	10.0000
16 Acquisition Time	1.3763
17 Acquisition Date	2018-11-27T16:13:36
18 Modification Date	2018-11-27T17:23:23
19 Spectrometer Frequency	100.61
20 Spectral Width	23809.5
21 Lowest Frequency	-1814.3
22 Nucleus	13C
23 Acquired Size	32768
24 Spectral Size	32768

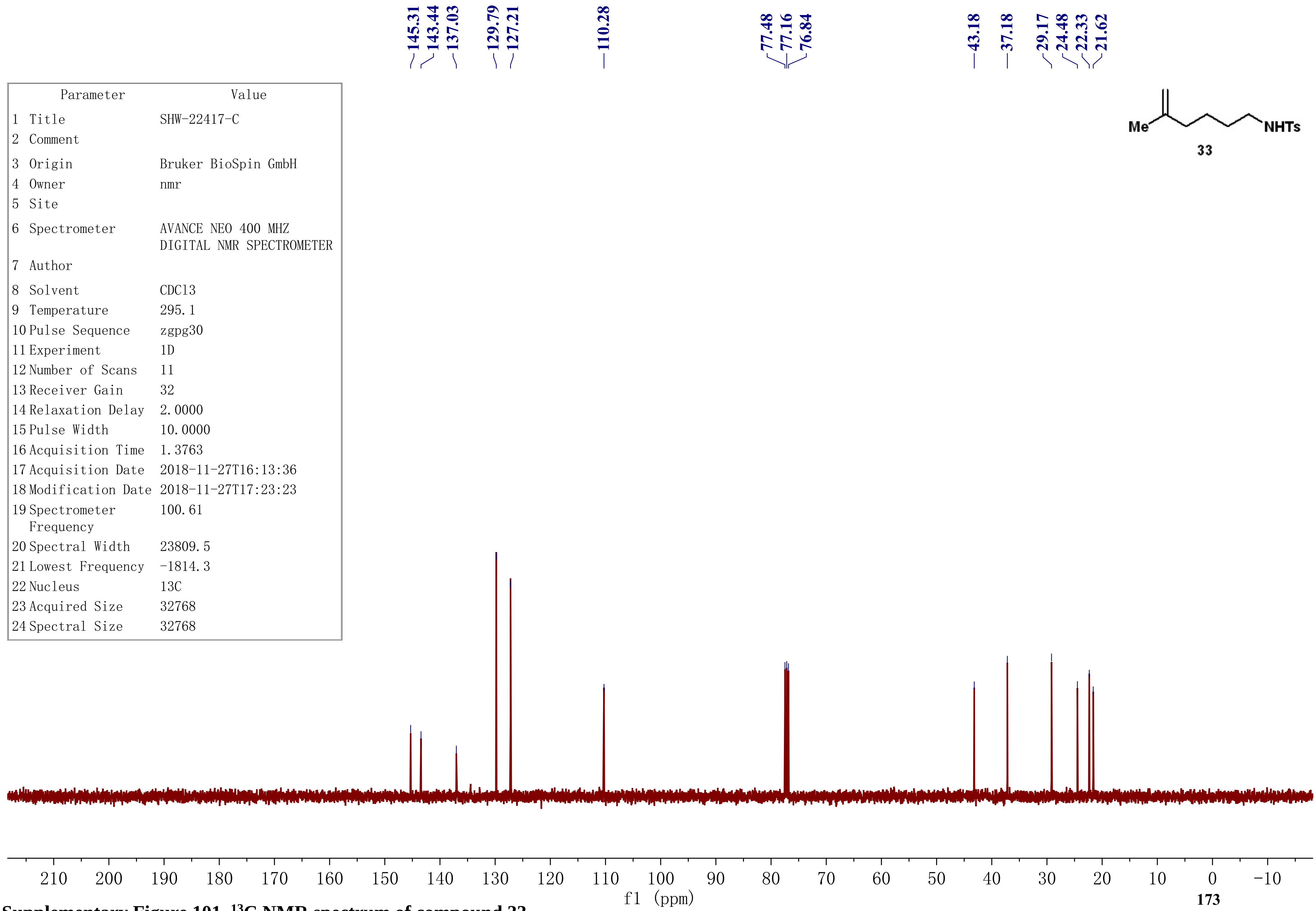

33

Supplementary Figure 101. ^{13}C NMR spectrum of compound 33

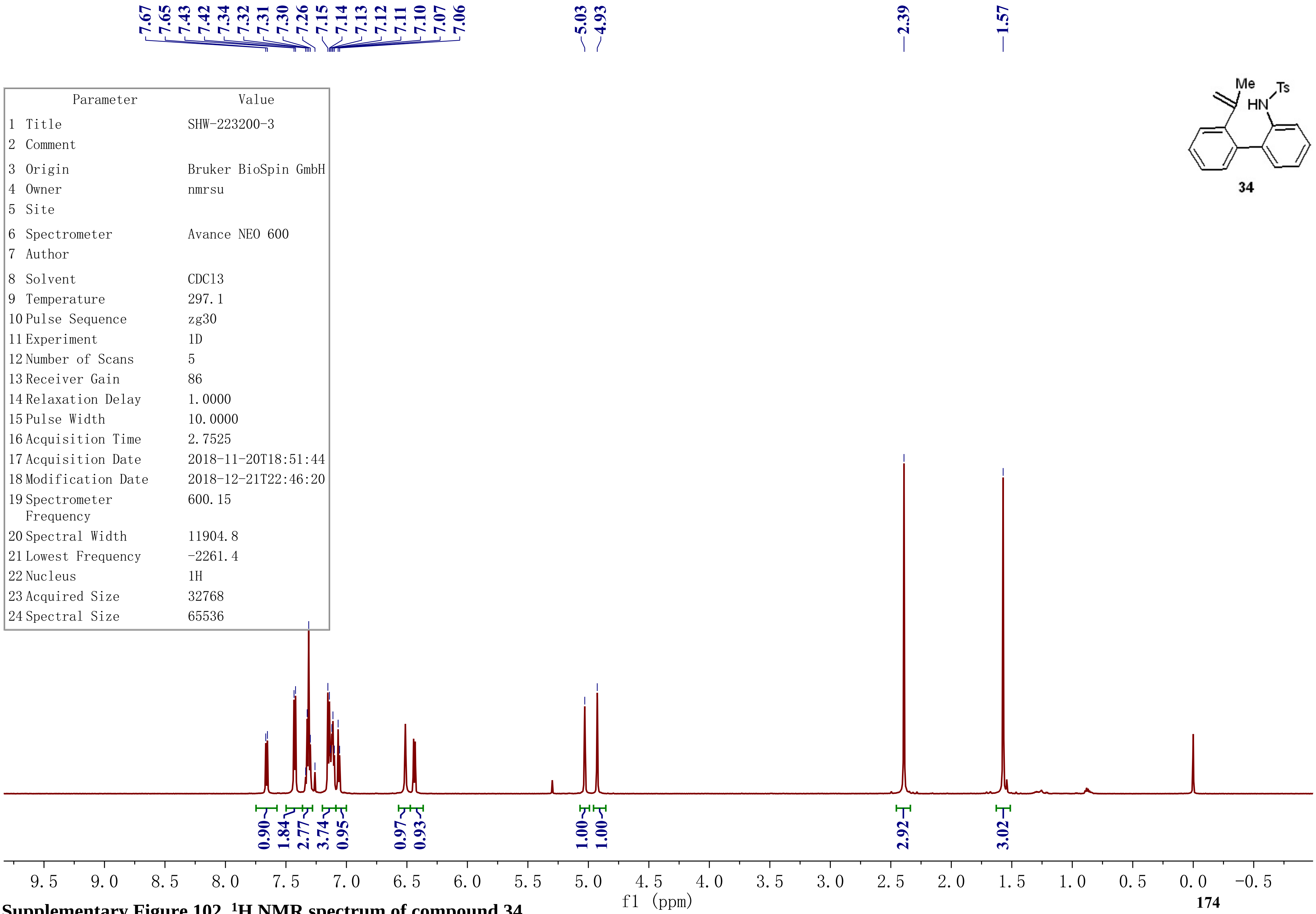

	Parameter	Value
1	Title	SHW-223200-3
2	Comment	
3	Origin	Bruker BioSpin GmbH
4	Owner	nmrsu
5	Site	
6	Spectrometer	Avance NEO 600
7	Author	
8	Solvent	CDCl3
9	Temperature	297.1
10	Pulse Sequence	zg30
11	Experiment	1D
12	Number of Scans	5
13	Receiver Gain	86
14	Relaxation Delay	1.0000
15	Pulse Width	10.0000
16	Acquisition Time	2.7525
17	Acquisition Date	2018-11-20T18:51:44
18	Modification Date	2018-12-21T22:46:20
19	Spectrometer Frequency	600.15
20	Spectral Width	11904.8
21	Lowest Frequency	-2261.4
22	Nucleus	1H
23	Acquired Size	32768
24	Spectral Size	65536

Supplementary Figure 102. ^{1}H NMR spectrum of compound 34

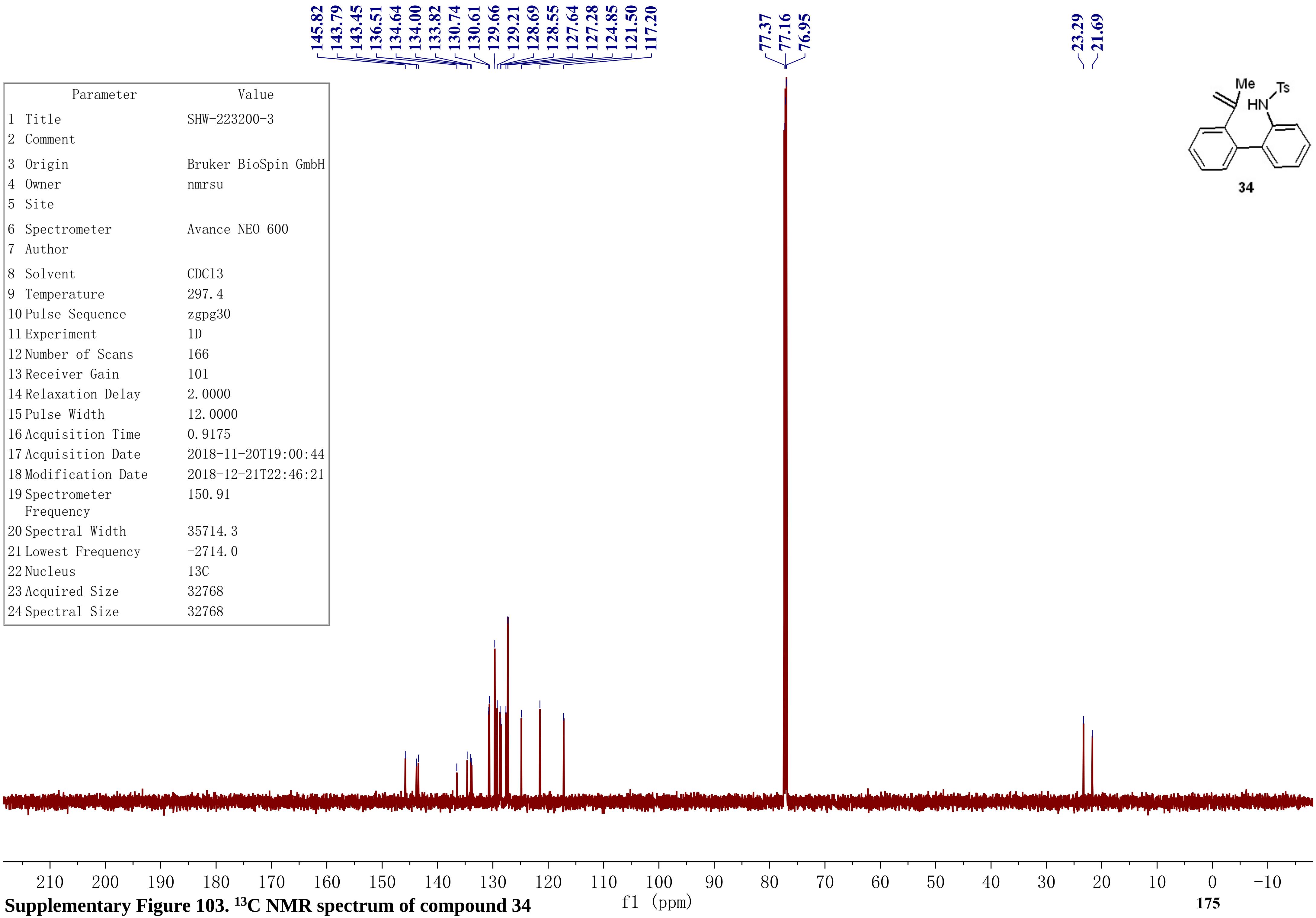

	Parameter	Value
1	Title	SHW-223200-3
2	Comment	
3	Origin	Bruker BioSpin GmbH
4	Owner	nmrsu
5	Site	
6	Spectrometer	Avance NEO 600
7	Author	
8	Solvent	CDCl3
9	Temperature	297.4
10	Pulse Sequence	zgpg30
11	Experiment	1D
12	Number of Scans	166
13	Receiver Gain	101
14	Relaxation Delay	2.0000
15	Pulse Width	12.0000
16	Acquisition Time	0.9175
17	Acquisition Date	2018-11-20T19:00:44
18	Modification Date	2018-12-21T22:46:21
19	Spectrometer Frequency	150.91
20	Spectral Width	35714.3
21	Lowest Frequency	-2714.0
22	Nucleus	13C
23	Acquired Size	32768
24	Spectral Size	32768

34

Supplementary Figure 103. ^{13}C NMR spectrum of compound 34

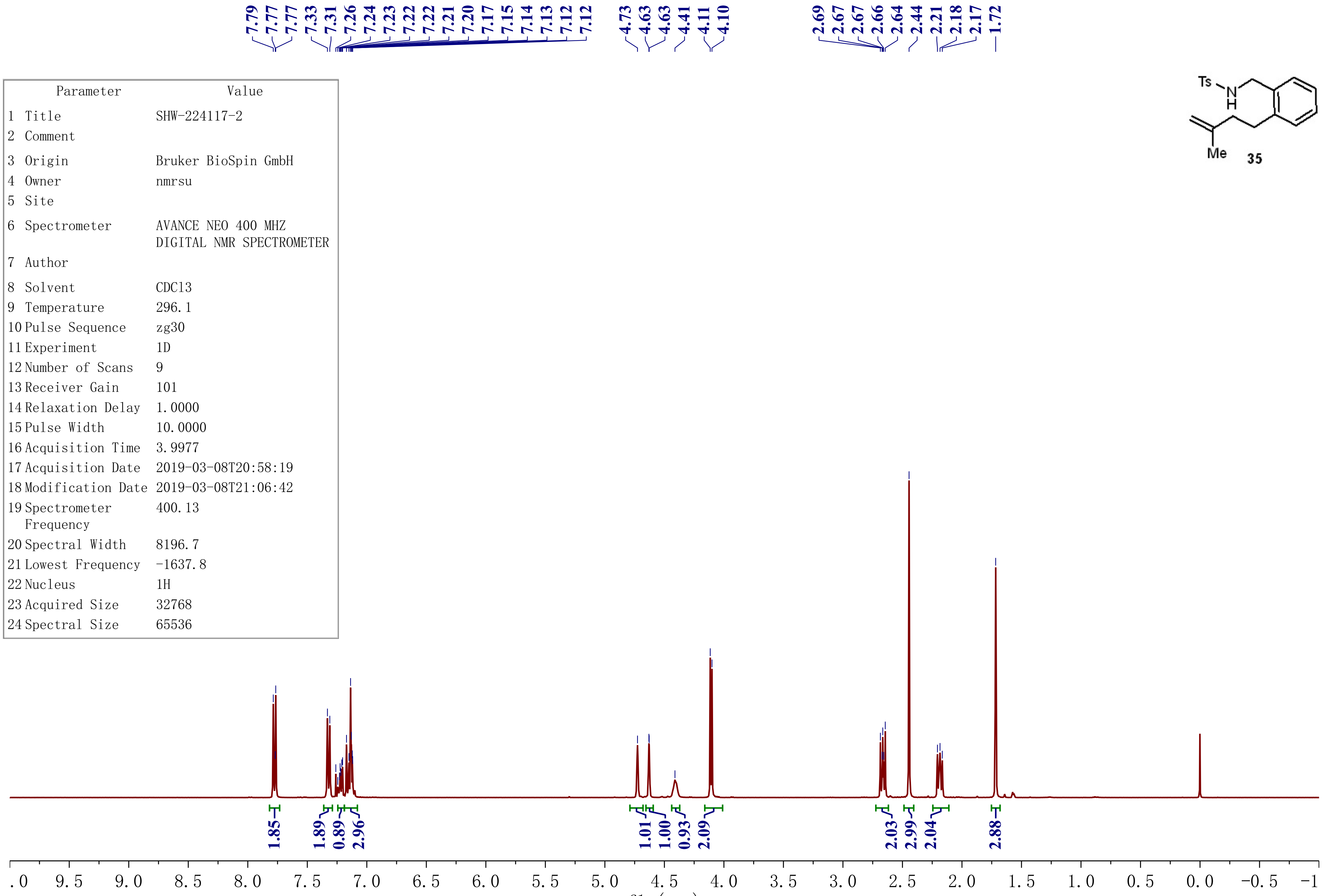

Parameter		Value
1	Title	SHW-224117-2
2	Comment	
3	Origin	Bruker BioSpin GmbH
4	Owner	nmrsu
5	Site	
6	Spectrometer	AVANCE NEO 400 MHZ DIGITAL NMR SPECTROMETER
7	Author	
8	Solvent	CDCl3
9	Temperature	296.1
10	Pulse Sequence	zg30
11	Experiment	1D
12	Number of Scans	9
13	Receiver Gain	101
14	Relaxation Delay	1.0000
15	Pulse Width	10.0000
16	Acquisition Time	3.9977
17	Acquisition Date	2019-03-08T20:58:19
18	Modification Date	2019-03-08T21:06:42
19	Spectrometer Frequency	400.13
20	Spectral Width	8196.7
21	Lowest Frequency	-1637.8
22	Nucleus	1H
23	Acquired Size	32768
24	Spectral Size	65536

35

Supplementary Figure 104. ^{1}H NMR spectrum of compound 35

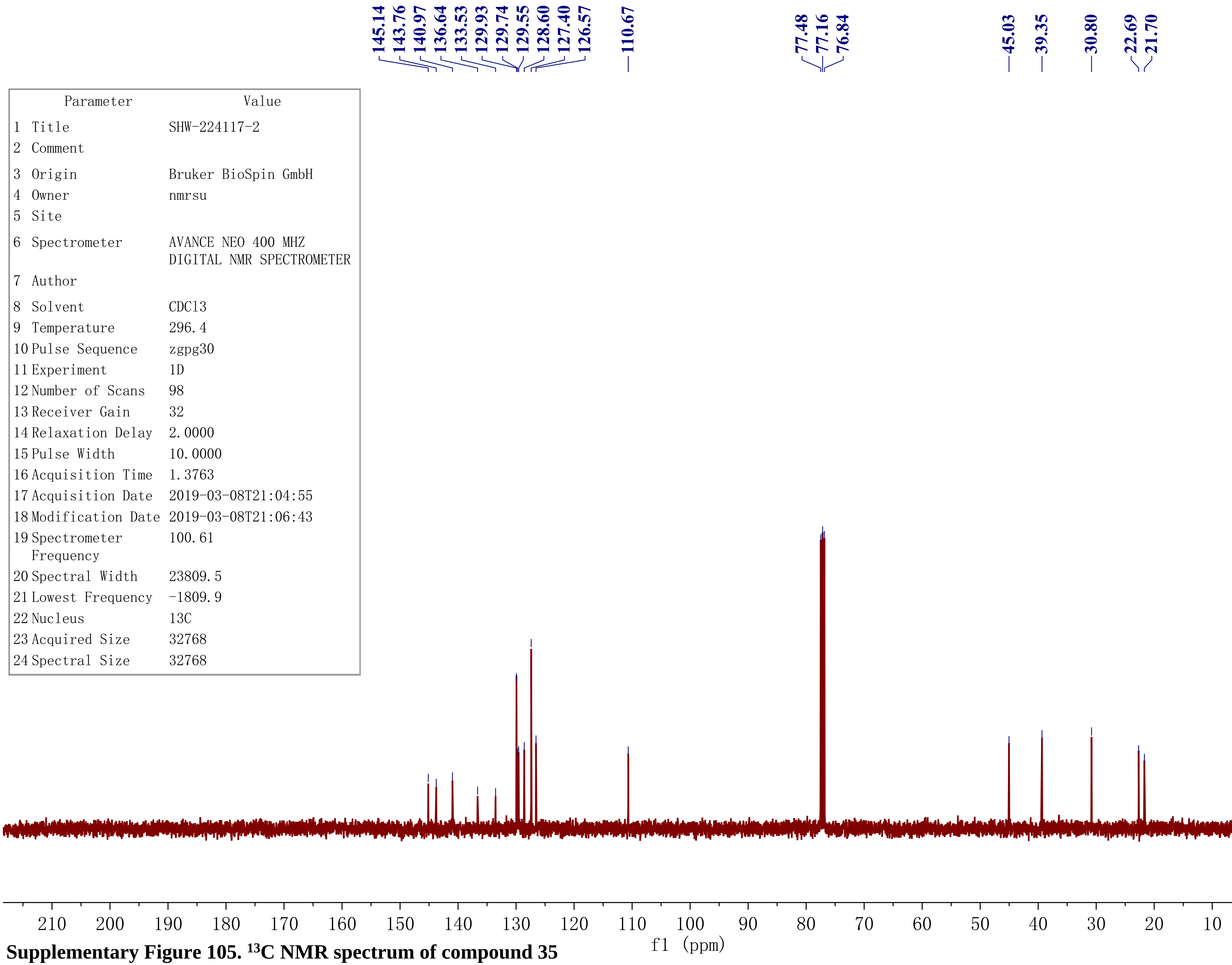

Parameter	Value
1 Title	SHW-224117-2
2 Comment	
3 Origin	Bruker BioSpin GmbH
4 Owner	nmrsu
5 Site	
6 Spectrometer	AVANCE NEO 400 MHZ DIGITAL NMR SPECTROMETER
7 Author	
8 Solvent	CDCl3
9 Temperature	296.4
10 Pulse Sequence	zgpg30
11 Experiment	1D
12 Number of Scans	98
13 Receiver Gain	32
14 Relaxation Delay	2.0000
15 Pulse Width	10.0000
16 Acquisition Time	1.3763
17 Acquisition Date	2019-03-08T21:04:55
18 Modification Date	2019-03-08T21:06:43
19 Spectrometer Frequency	100.61
20 Spectral Width	23809.5
21 Lowest Frequency	-1809.9
22 Nucleus	13C
23 Acquired Size	32768
24 Spectral Size	32768

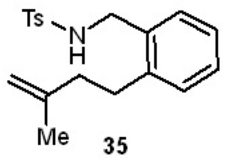

35

Supplementary Figure 105. ^{13}C NMR spectrum of compound 35

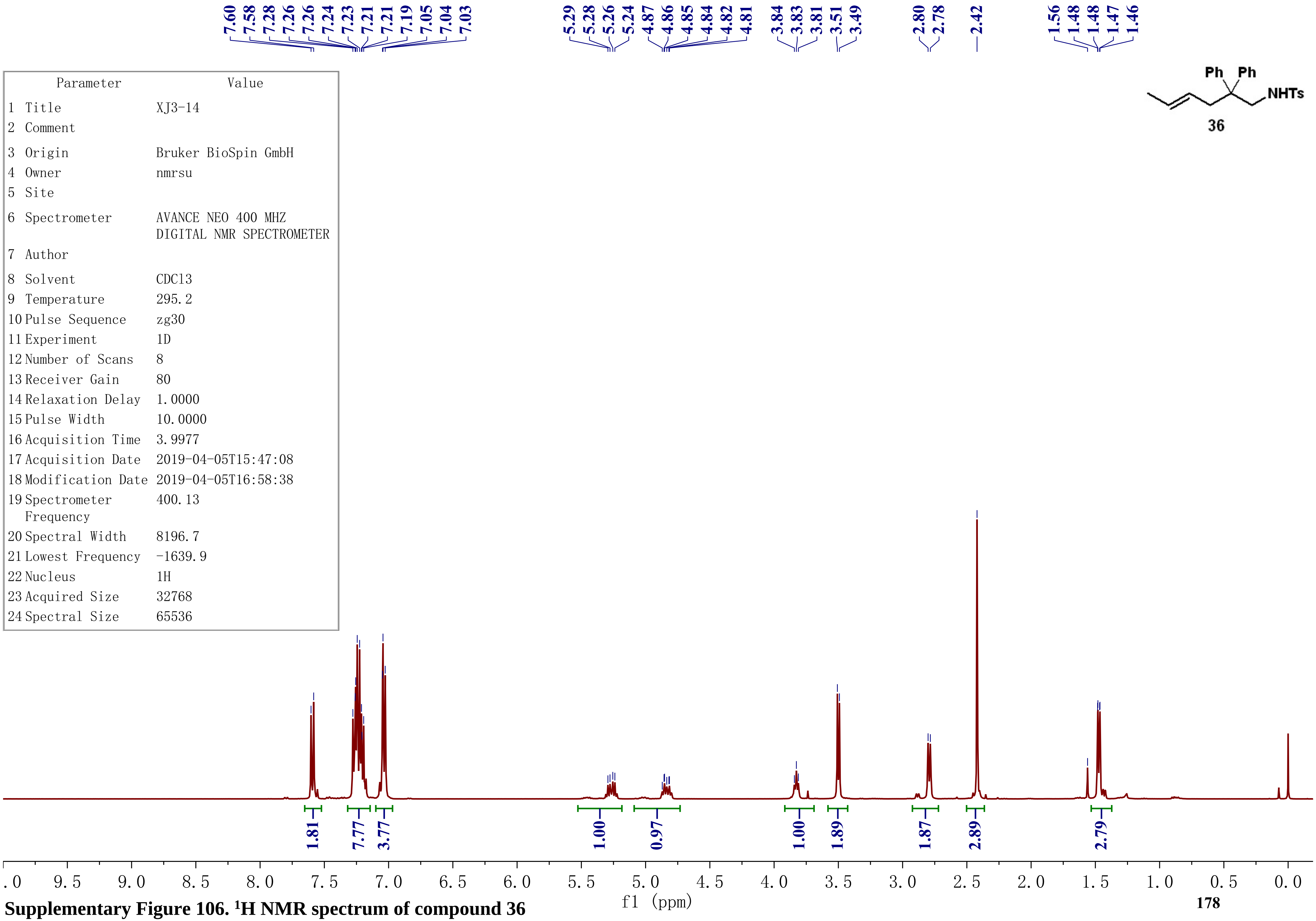

	Parameter	Value
1	Title	XJ3-14
2	Comment	
3	Origin	Bruker BioSpin GmbH
4	Owner	nmrsu
5	Site	
6	Spectrometer	AVANCE NEO 400 MHZ DIGITAL NMR SPECTROMETER
7	Author	
8	Solvent	CDCl3
9	Temperature	295.2
10	Pulse Sequence	zg30
11	Experiment	1D
12	Number of Scans	8
13	Receiver Gain	80
14	Relaxation Delay	1.0000
15	Pulse Width	10.0000
16	Acquisition Time	3.9977
17	Acquisition Date	2019-04-05T15:47:08
18	Modification Date	2019-04-05T16:58:38
19	Spectrometer Frequency	400.13
20	Spectral Width	8196.7
21	Lowest Frequency	-1639.9
22	Nucleus	1H
23	Acquired Size	32768
24	Spectral Size	65536

36

Supplementary Figure 106. ^1H NMR spectrum of compound 36

36

Parameter		Value
1	Title	XJ3-14
2	Comment	
3	Origin	Bruker BioSpin GmbH
4	Owner	nmrsu
5	Site	
6	Spectrometer	AVANCE NEO 400 MHZ DIGITAL NMR SPECTROMETER
7	Author	
8	Solvent	CDCl3
9	Temperature	295.8
10	Pulse Sequence	zgpg30
11	Experiment	1D
12	Number of Scans	29
13	Receiver Gain	32
14	Relaxation Delay	2.0000
15	Pulse Width	10.0000
16	Acquisition Time	1.3763
17	Acquisition Date	2019-04-05T15:50:01
18	Modification Date	2019-04-05T16:58:39
19	Spectrometer Frequency	100.61
20	Spectral Width	23809.5
21	Lowest Frequency	-1833.4
22	Nucleus	13C
23	Acquired Size	32768
24	Spectral Size	32768

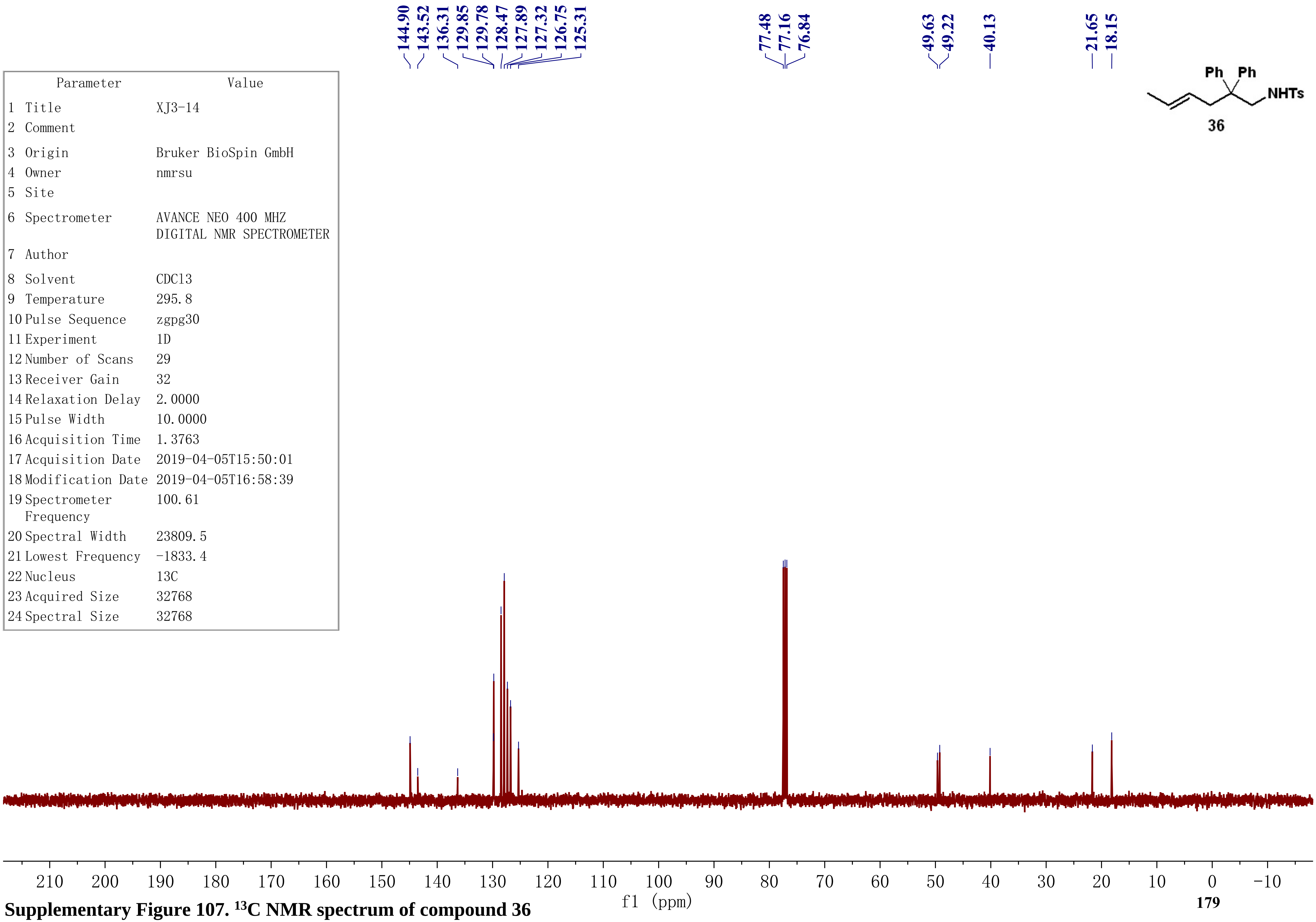

Supplementary Figure 107. ^{13}**C NMR spectrum of compound 36**

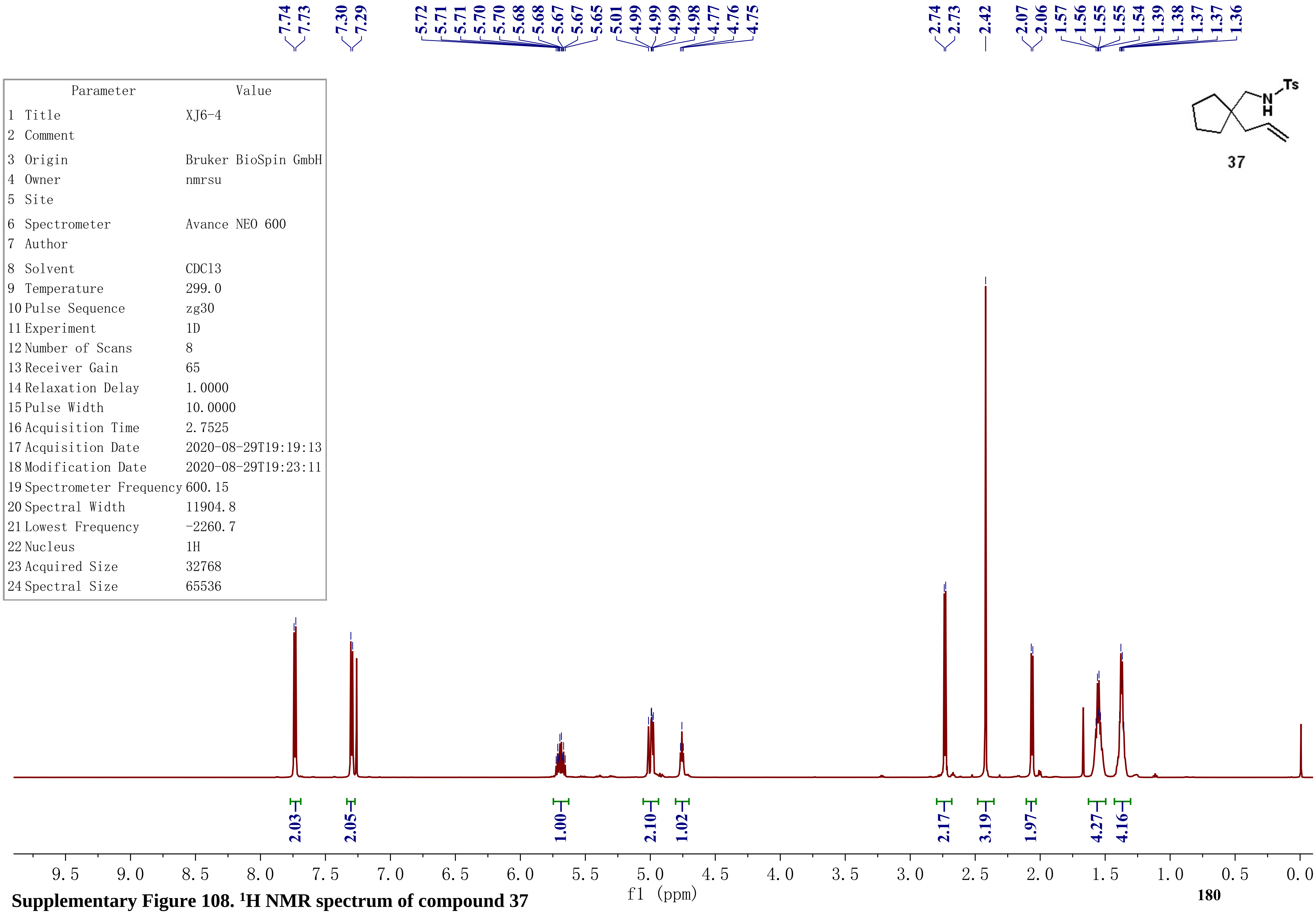

Supplementary Figure 108. ^{1}H NMR spectrum of compound 37

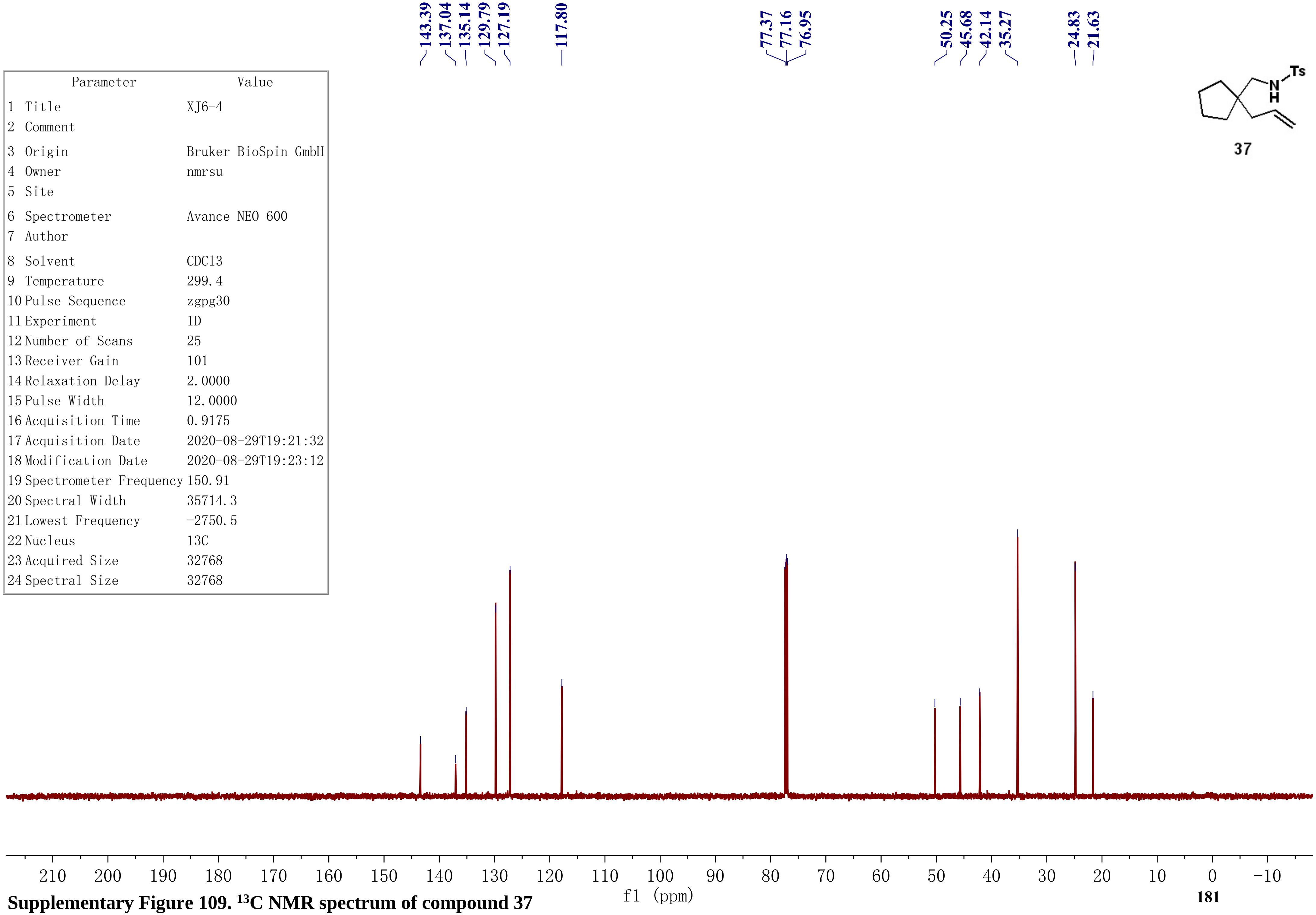

	Parameter	Value
1	Title	XJ6-4
2	Comment	
3	Origin	Bruker BioSpin GmbH
4	Owner	nmrsu
5	Site	
6	Spectrometer	Avance NEO 600
7	Author	
8	Solvent	CDCl3
9	Temperature	299.4
10	Pulse Sequence	zgpg30
11	Experiment	1D
12	Number of Scans	25
13	Receiver Gain	101
14	Relaxation Delay	2.0000
15	Pulse Width	12.0000
16	Acquisition Time	0.9175
17	Acquisition Date	2020-08-29T19:21:32
18	Modification Date	2020-08-29T19:23:12
19	Spectrometer Frequency	150.91
20	Spectral Width	35714.3
21	Lowest Frequency	-2750.5
22	Nucleus	13C
23	Acquired Size	32768
24	Spectral Size	32768

37

Supplementary Figure 109. ^{13}C NMR spectrum of compound 37

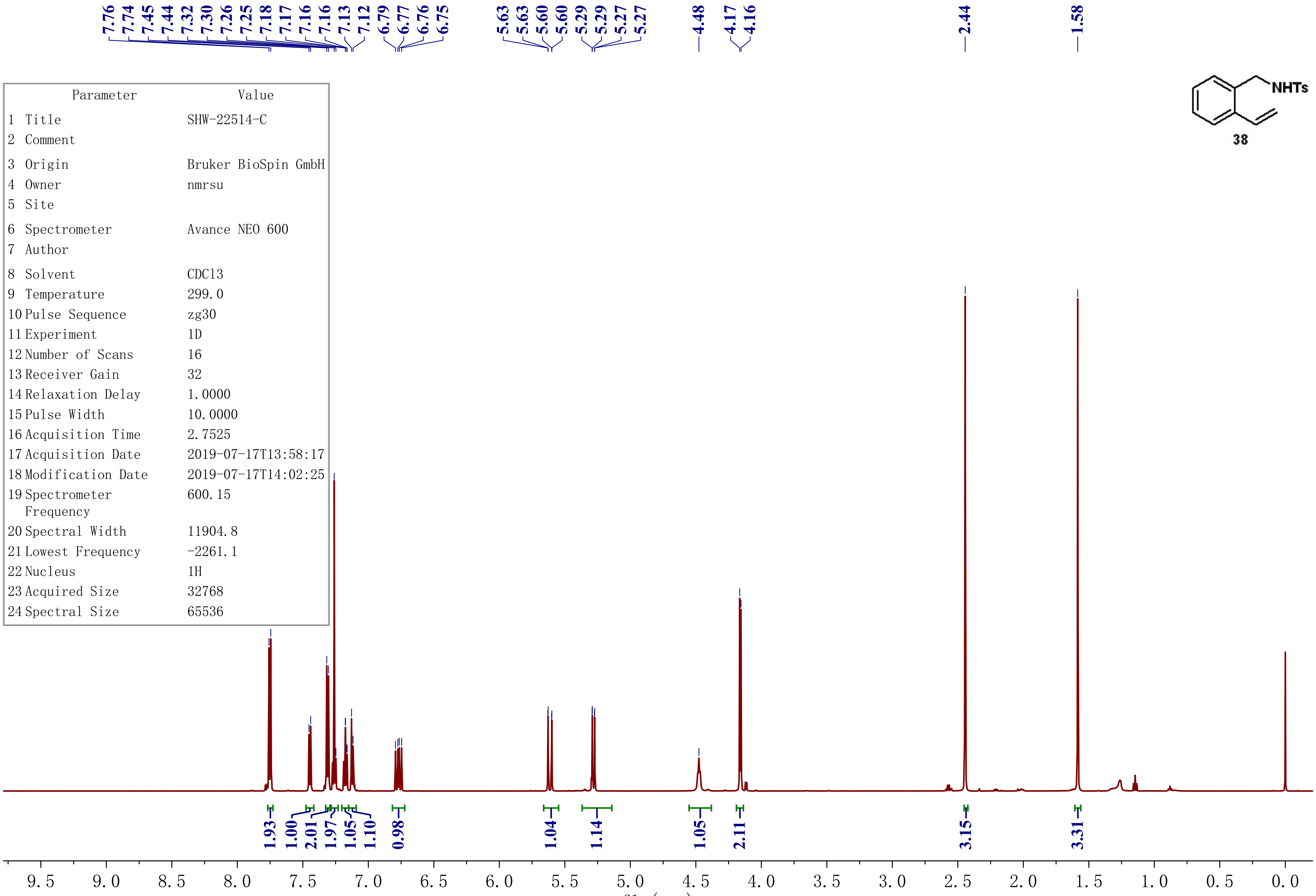

	Parameter	Value
1	Title	SHW-22514-C
2	Comment	
3	Origin	Bruker BioSpin GmbH
4	Owner	nmrsu
5	Site	
6	Spectrometer	Avance NEO 600
7	Author	
8	Solvent	CDCl3
9	Temperature	299.0
10	Pulse Sequence	zg30
11	Experiment	1D
12	Number of Scans	16
13	Receiver Gain	32
14	Relaxation Delay	1.0000
15	Pulse Width	10.0000
16	Acquisition Time	2.7525
17	Acquisition Date	2019-07-17T13:58:17
18	Modification Date	2019-07-17T14:02:25
19	Spectrometer Frequency	600.15
20	Spectral Width	11904.8
21	Lowest Frequency	-2261.1
22	Nucleus	1H
23	Acquired Size	32768
24	Spectral Size	65536

38

Supplementary Figure 110. ^{1}H NMR spectrum of compound 38

Parameter	Value
1 Title	SHW-22514-C
2 Comment	
3 Origin	Bruker BioSpin GmbH
4 Owner	nmrsu
5 Site	
6 Spectrometer	Avance NEO 600
7 Author	
8 Solvent	CDCl3
9 Temperature	300.0
10 Pulse Sequence	zgpg30
11 Experiment	1D
12 Number of Scans	377
13 Receiver Gain	101
14 Relaxation Delay	2.0000
15 Pulse Width	12.0000
16 Acquisition Time	0.9175
17 Acquisition Date	2019-07-17T13:56:41
18 Modification Date	2019-07-17T14:02:25
19 Spectrometer Frequency	150.91
20 Spectral Width	35714.3
21 Lowest Frequency	-2715.8
22 Nucleus	13C
23 Acquired Size	32768
24 Spectral Size	32768

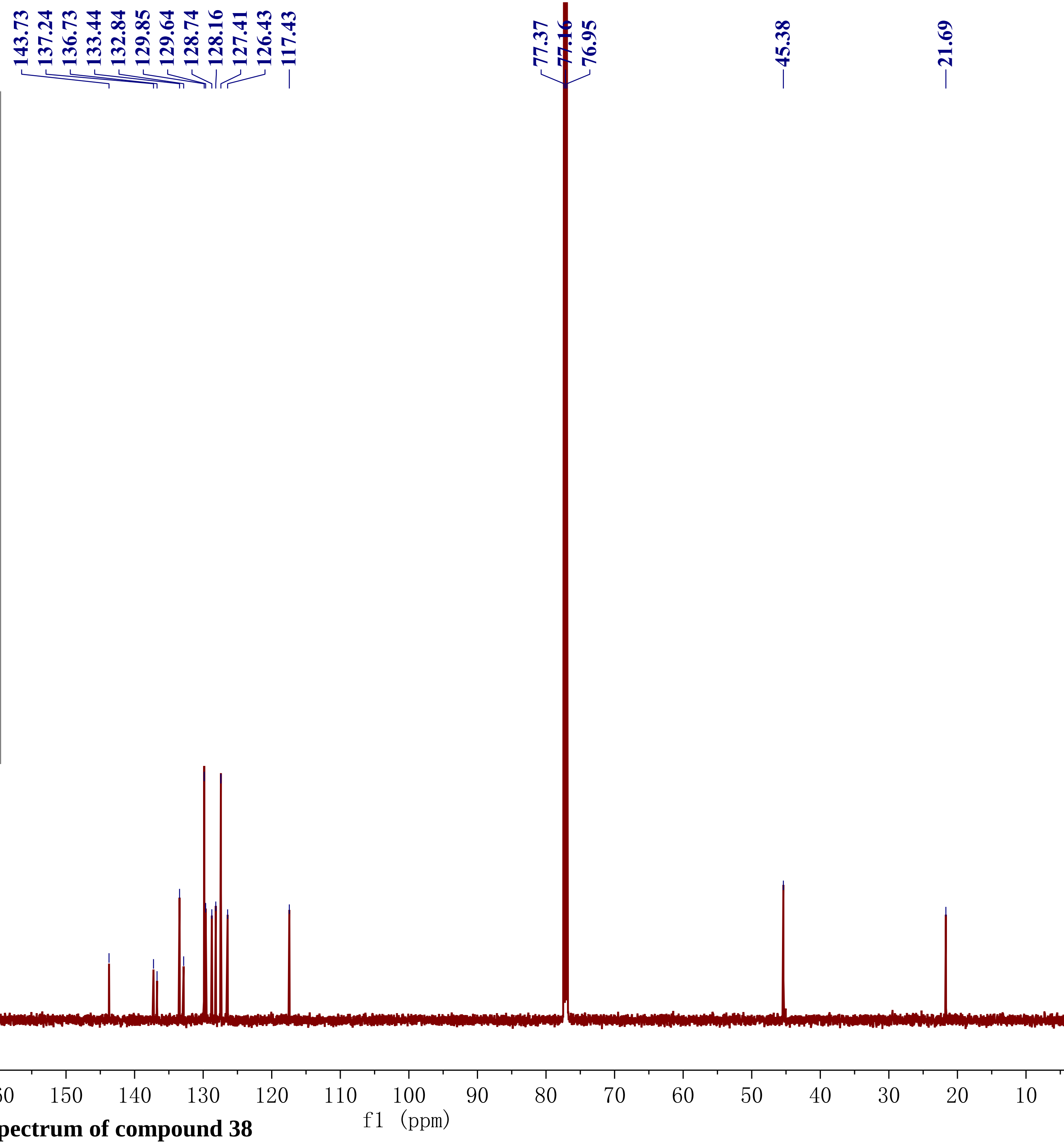

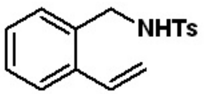

38

Supplementary Figure 111. ^{13}C **NMR spectrum of compound 38**

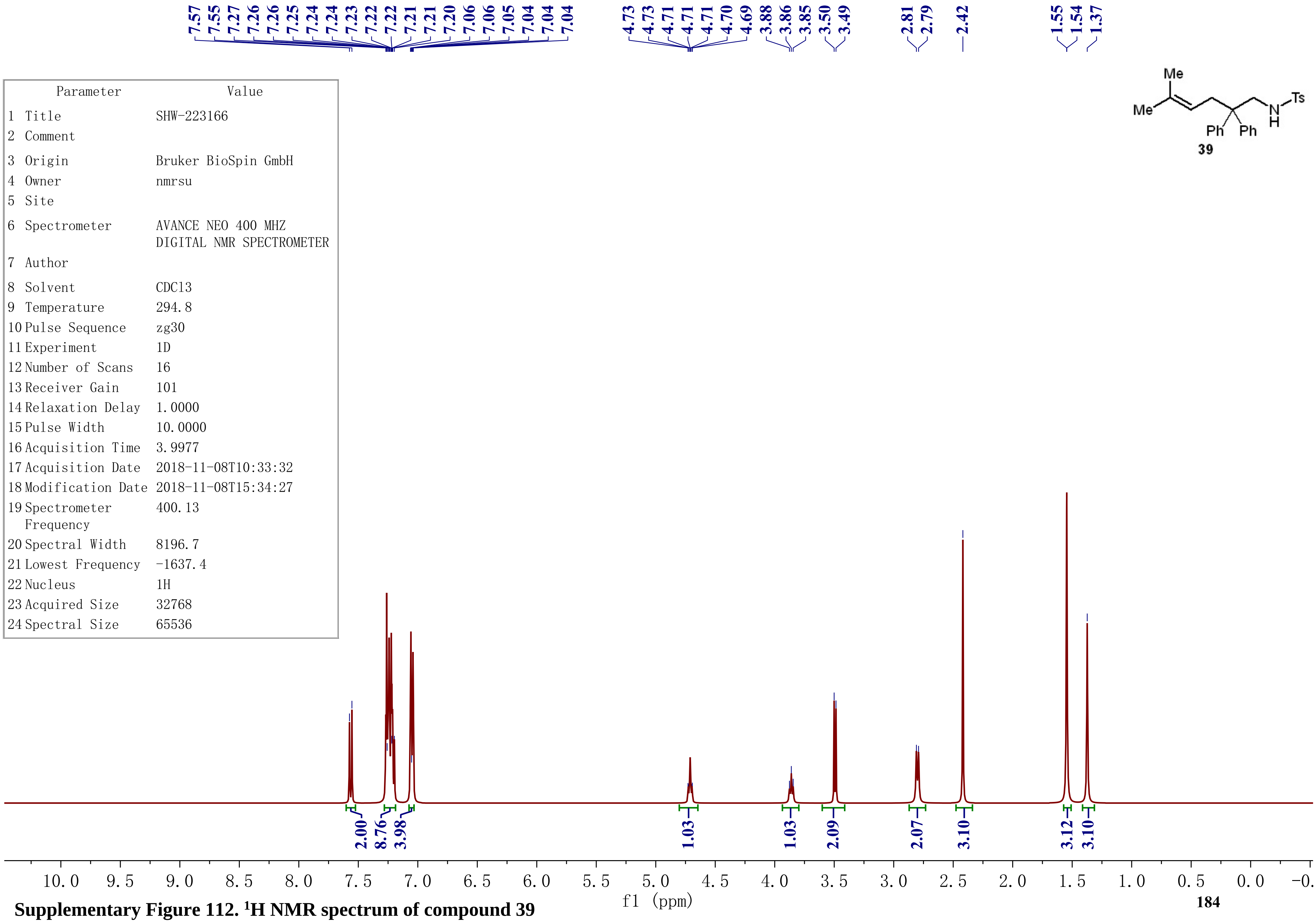

Parameter		Value
1	Title	SHW-223166
2	Comment	
3	Origin	Bruker BioSpin GmbH
4	Owner	nmrsu
5	Site	
6	Spectrometer	AVANCE NEO 400 MHZ DIGITAL NMR SPECTROMETER
7	Author	
8	Solvent	CDCl3
9	Temperature	294.8
10	Pulse Sequence	zg30
11	Experiment	1D
12	Number of Scans	16
13	Receiver Gain	101
14	Relaxation Delay	1.0000
15	Pulse Width	10.0000
16	Acquisition Time	3.9977
17	Acquisition Date	2018-11-08T10:33:32
18	Modification Date	2018-11-08T15:34:27
19	Spectrometer Frequency	400.13
20	Spectral Width	8196.7
21	Lowest Frequency	-1637.4
22	Nucleus	1H
23	Acquired Size	32768
24	Spectral Size	65536

39

Supplementary Figure 112. ^{1}H NMR spectrum of compound 39

Parameter		Value
1	Title	SHW-223166-C
2	Comment	
3	Origin	Bruker BioSpin GmbH
4	Owner	nmrsu
5	Site	
6	Spectrometer	AVANCE NEO 400 MHZ DIGITAL NMR SPECTROMETER
7	Author	
8	Solvent	CDCl3
9	Temperature	296.2
10	Pulse Sequence	zgpg30
11	Experiment	1D
12	Number of Scans	71
13	Receiver Gain	32
14	Relaxation Delay	2.0000
15	Pulse Width	10.0000
16	Acquisition Time	1.3763
17	Acquisition Date	2018-12-21T22:14:19
18	Modification Date	2018-12-21T22:26:48
19	Spectrometer Frequency	100.61
20	Spectral Width	23809.5
21	Lowest Frequency	-1843.5
22	Nucleus	13C
23	Acquired Size	32768
24	Spectral Size	32768

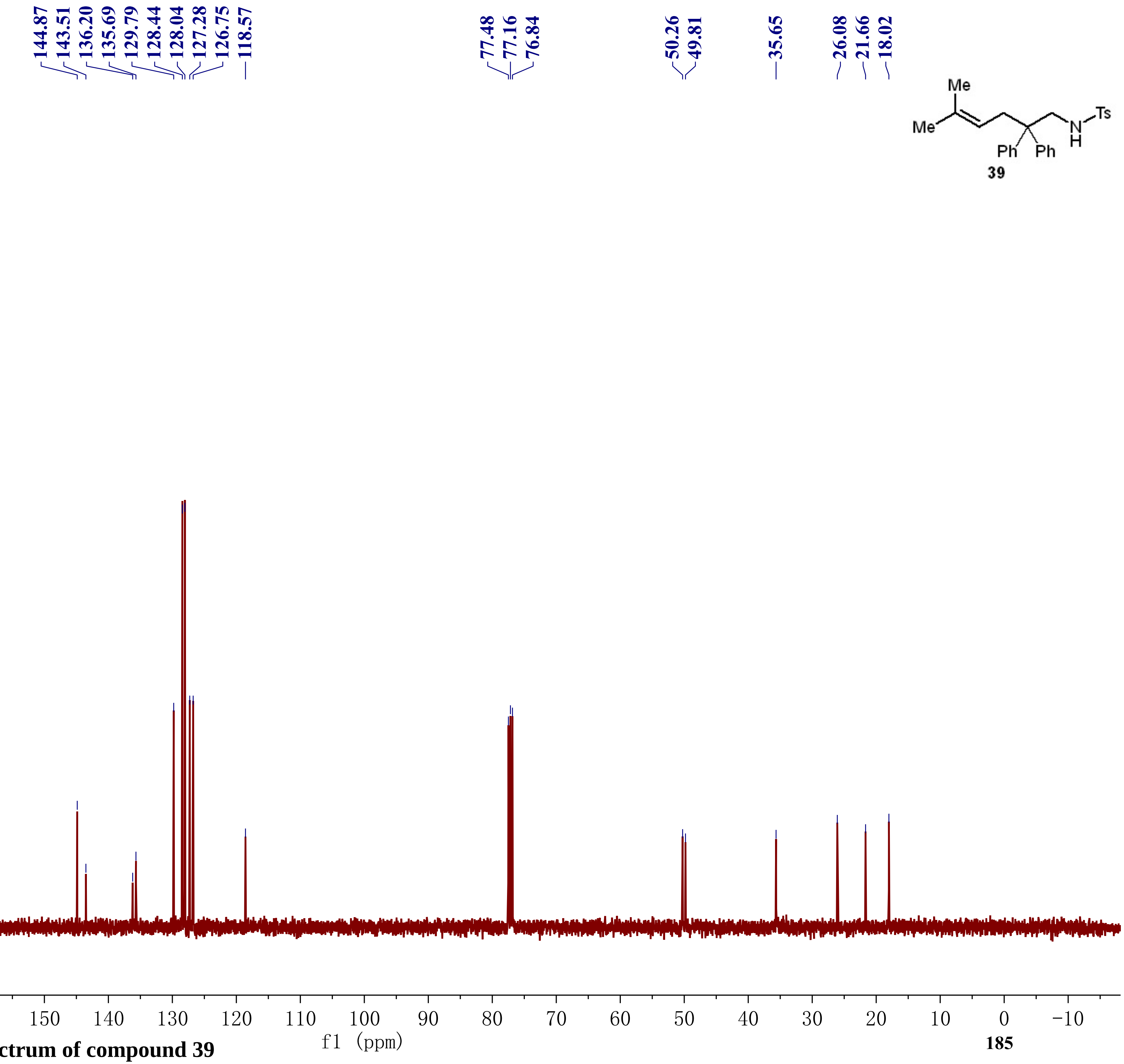

Supplementary Figure 113. ^{13}C NMR spectrum of compound 39

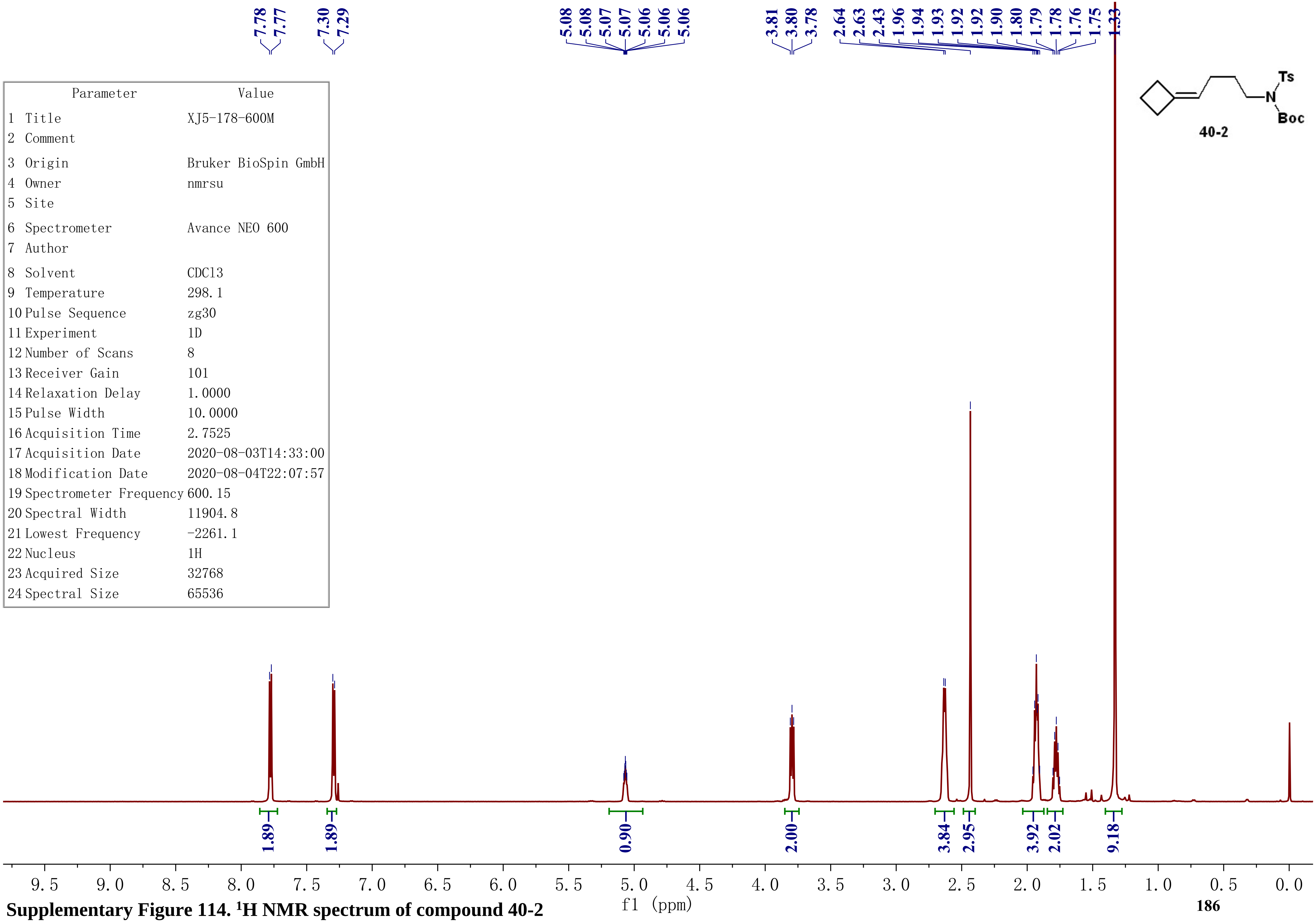

	Parameter	Value
1	Title	XJ5-178-600M
2	Comment	
3	Origin	Bruker BioSpin GmbH
4	Owner	nmrsu
5	Site	
6	Spectrometer	Avance NEO 600
7	Author	
8	Solvent	CDCl3
9	Temperature	298.1
10	Pulse Sequence	zg30
11	Experiment	1D
12	Number of Scans	8
13	Receiver Gain	101
14	Relaxation Delay	1.0000
15	Pulse Width	10.0000
16	Acquisition Time	2.7525
17	Acquisition Date	2020-08-03T14:33:00
18	Modification Date	2020-08-04T22:07:57
19	Spectrometer Frequency	600.15
20	Spectral Width	11904.8
21	Lowest Frequency	-2261.1
22	Nucleus	1H
23	Acquired Size	32768
24	Spectral Size	65536

Supplementary Figure 114. ^{1}H NMR spectrum of compound 40-2

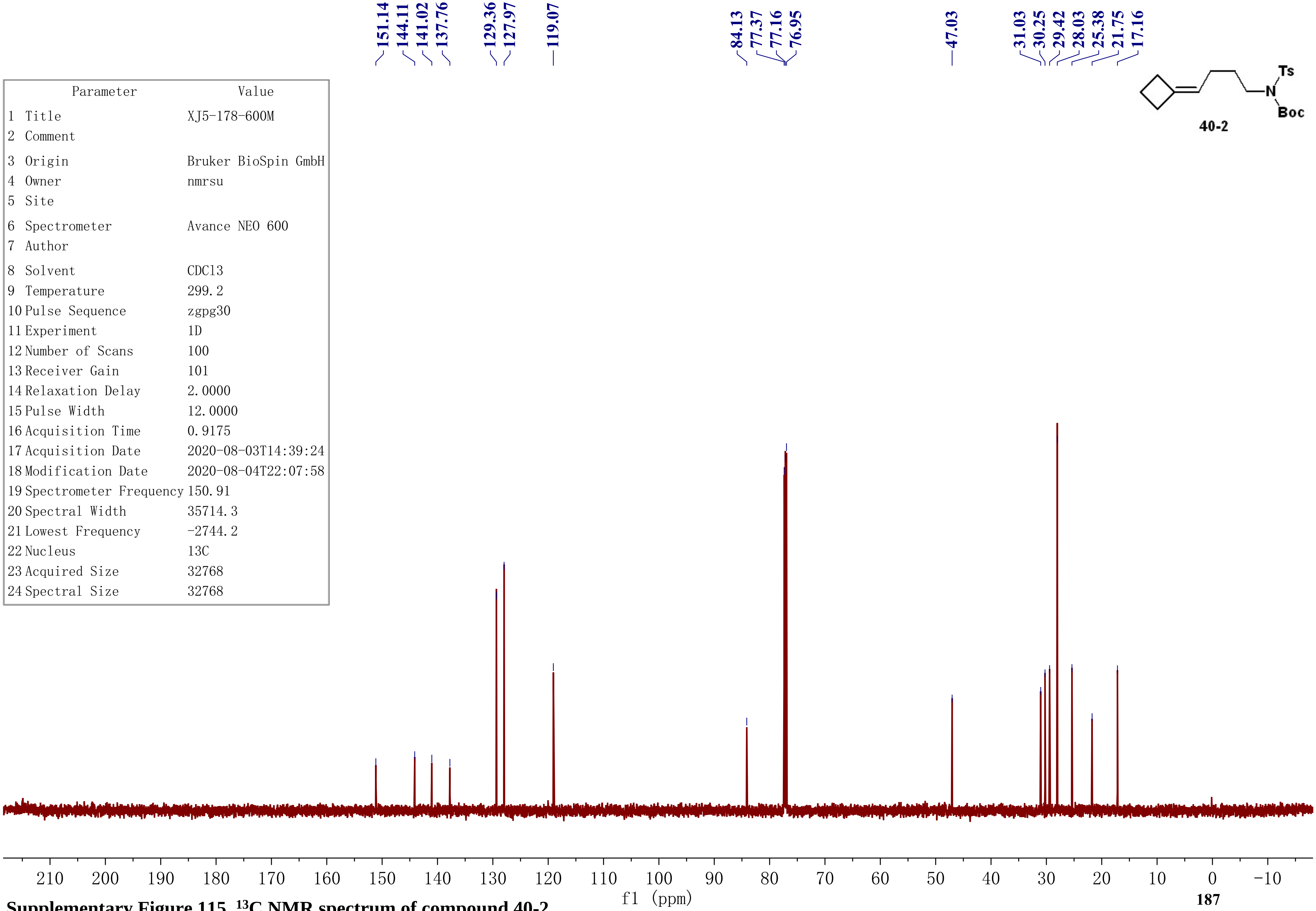

	Parameter	Value
1	Title	XJ5-178-600M
2	Comment	
3	Origin	Bruker BioSpin GmbH
4	Owner	nmrsu
5	Site	
6	Spectrometer	Avance NEO 600
7	Author	
8	Solvent	CDCl3
9	Temperature	299.2
10	Pulse Sequence	zgpg30
11	Experiment	1D
12	Number of Scans	100
13	Receiver Gain	101
14	Relaxation Delay	2.0000
15	Pulse Width	12.0000
16	Acquisition Time	0.9175
17	Acquisition Date	2020-08-03T14:39:24
18	Modification Date	2020-08-04T22:07:58
19	Spectrometer Frequency	150.91
20	Spectral Width	35714.3
21	Lowest Frequency	-2744.2
22	Nucleus	13C
23	Acquired Size	32768
24	Spectral Size	32768

40-2

Supplementary Figure 115. ^{13}C NMR spectrum of compound 40-2

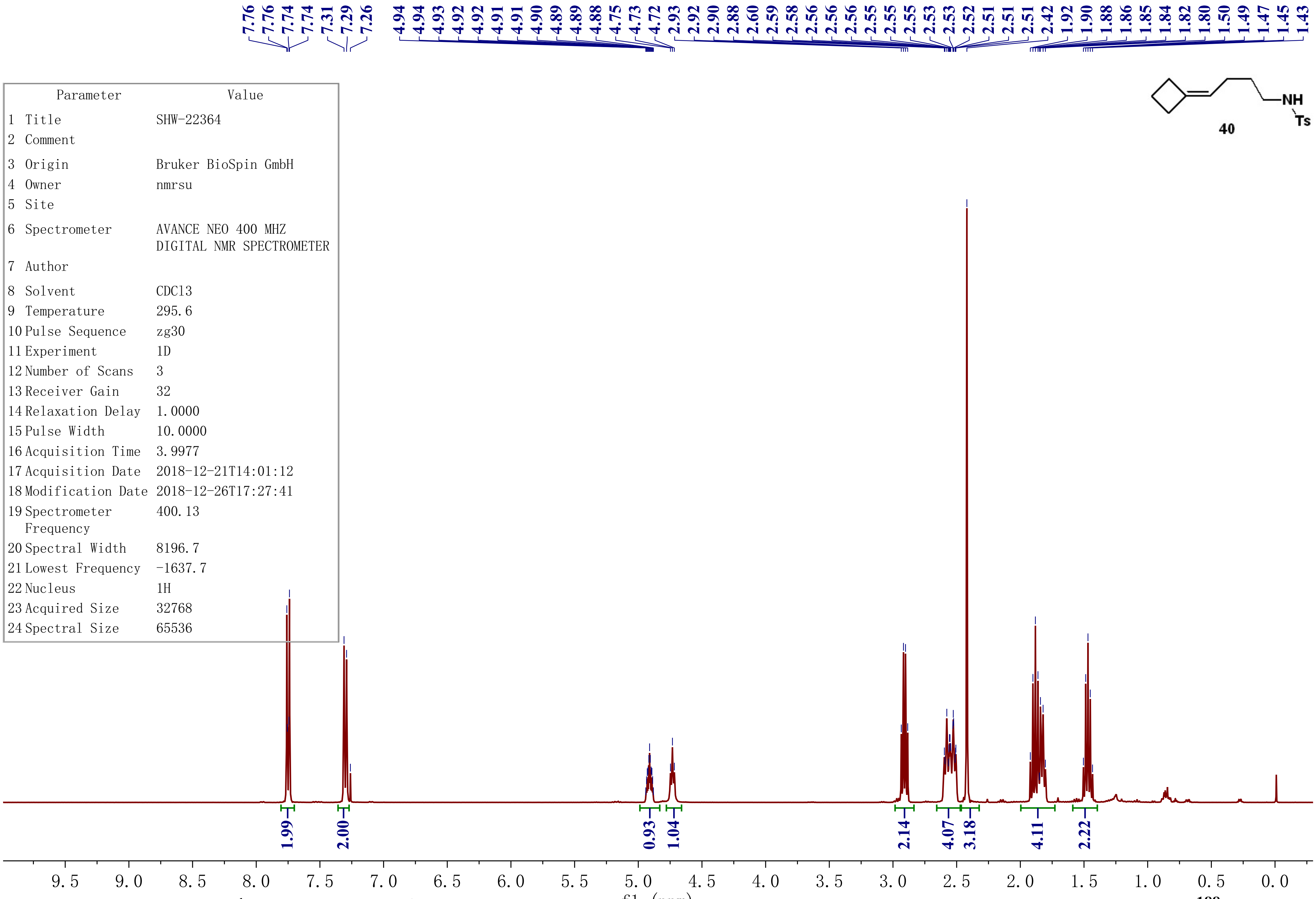

Supplementary Figure 116. ^{1}H NMR spectrum of compound 40

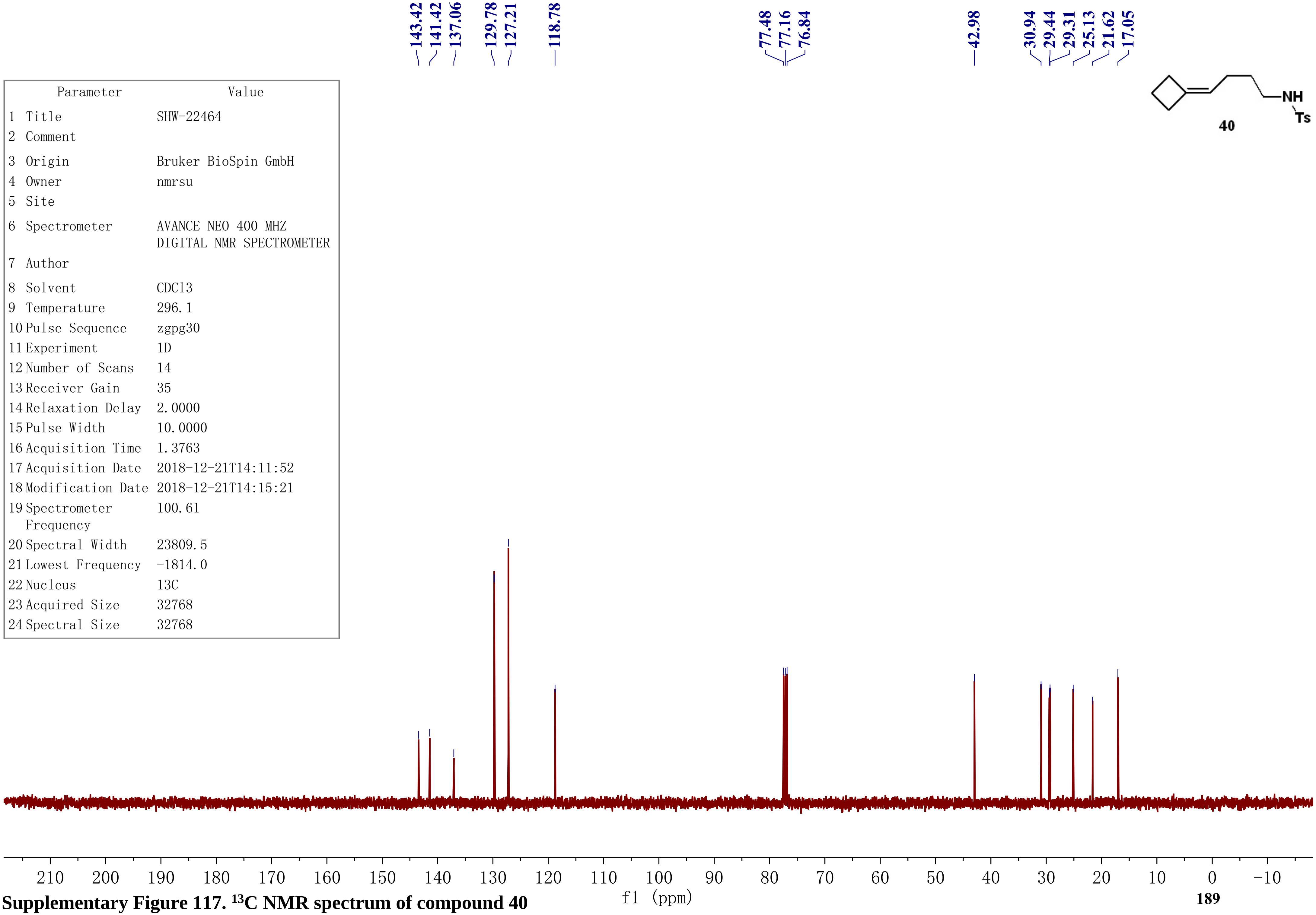

	Parameter	Value
1	Title	SHW-22464
2	Comment	
3	Origin	Bruker BioSpin GmbH
4	Owner	nmrsu
5	Site	
6	Spectrometer	AVANCE NEO 400 MHZ DIGITAL NMR SPECTROMETER
7	Author	
8	Solvent	CDCl3
9	Temperature	296.1
10	Pulse Sequence	zgpg30
11	Experiment	1D
12	Number of Scans	14
13	Receiver Gain	35
14	Relaxation Delay	2.0000
15	Pulse Width	10.0000
16	Acquisition Time	1.3763
17	Acquisition Date	2018-12-21T14:11:52
18	Modification Date	2018-12-21T14:15:21
19	Spectrometer Frequency	100.61
20	Spectral Width	23809.5
21	Lowest Frequency	-1814.0
22	Nucleus	13C
23	Acquired Size	32768
24	Spectral Size	32768

40

Supplementary Figure 117. ^{13}C NMR spectrum of compound 40

Parameter	Value
1 Title	SHW-224189
2 Comment	
3 Origin	Bruker BioSpin GmbH
4 Owner	nmrsu
5 Site	
6 Spectrometer	AVANCE NEO 400 MHZ DIGITAL NMR SPECTROMETER
7 Author	
8 Solvent	CDCl3
9 Temperature	299.0
10 Pulse Sequence	zg30
11 Experiment	1D
12 Number of Scans	16
13 Receiver Gain	101
14 Relaxation Delay	1.0000
15 Pulse Width	10.0000
16 Acquisition Time	3.9977
17 Acquisition Date	2019-07-16T21:47:23
18 Modification Date	2019-07-17T12:53:01
19 Spectrometer Frequency	400.13
20 Spectral Width	8196.7
21 Lowest Frequency	-1638.5
22 Nucleus	1H
23 Acquired Size	32768
24 Spectral Size	65536

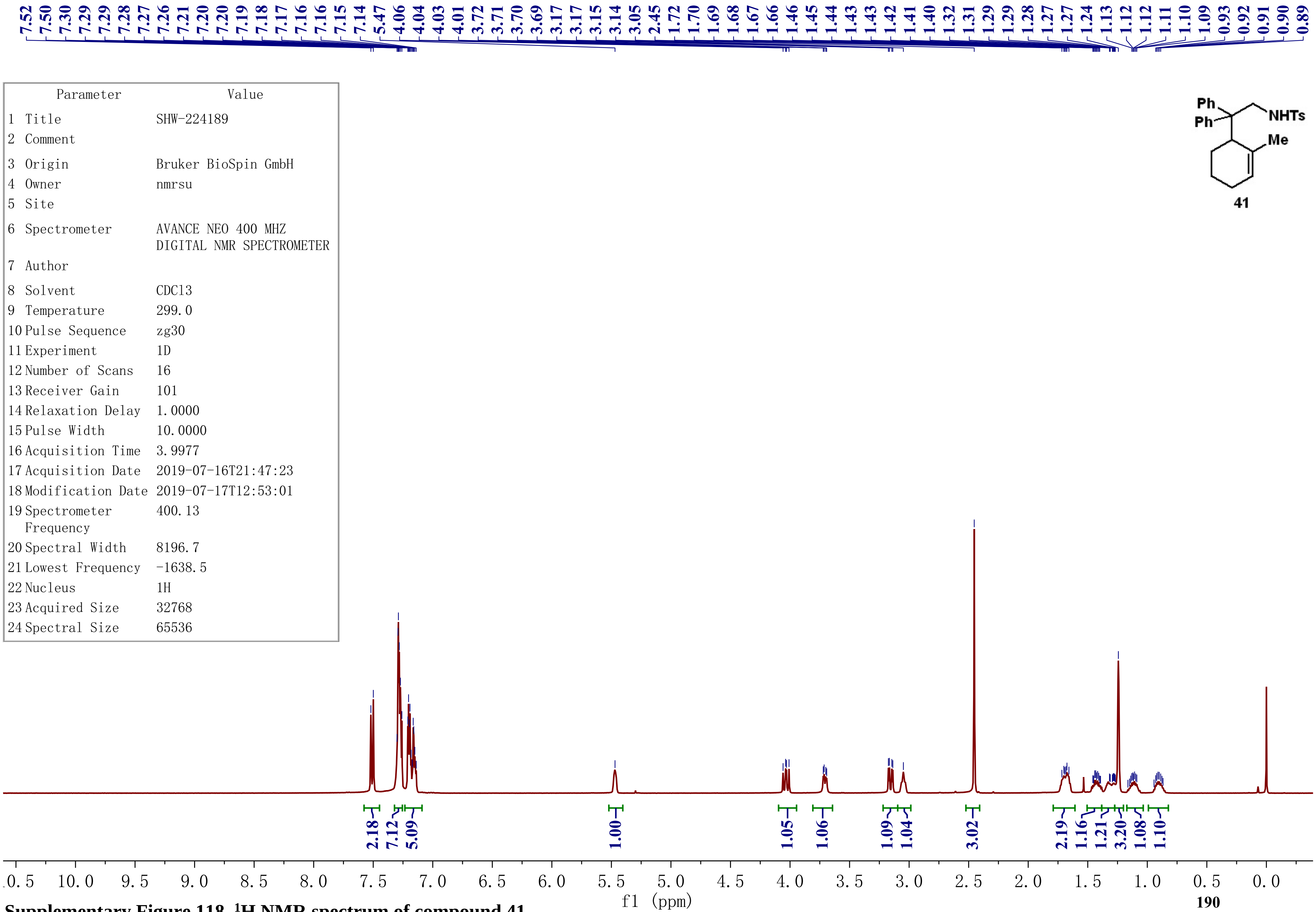

41

Supplementary Figure 118. ^{1}H NMR spectrum of compound 41

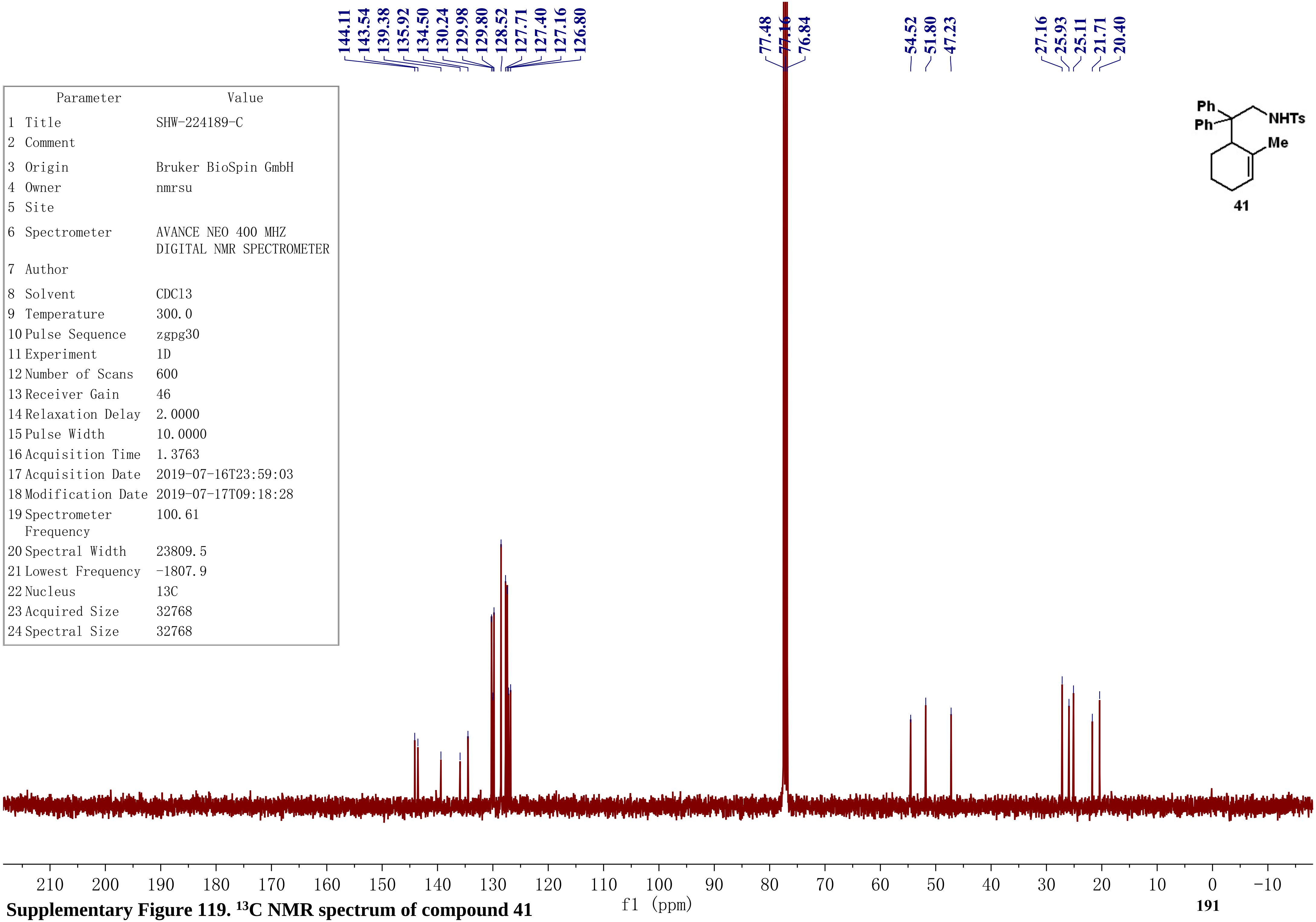

	Parameter	Value
1	Title	SHW-224189-C
2	Comment	
3	Origin	Bruker BioSpin GmbH
4	Owner	nmrsu
5	Site	
6	Spectrometer	AVANCE NEO 400 MHZ DIGITAL NMR SPECTROMETER
7	Author	
8	Solvent	CDCl3
9	Temperature	300.0
10	Pulse Sequence	zgpg30
11	Experiment	1D
12	Number of Scans	600
13	Receiver Gain	46
14	Relaxation Delay	2.0000
15	Pulse Width	10.0000
16	Acquisition Time	1.3763
17	Acquisition Date	2019-07-16T23:59:03
18	Modification Date	2019-07-17T09:18:28
19	Spectrometer Frequency	100.61
20	Spectral Width	23809.5
21	Lowest Frequency	-1807.9
22	Nucleus	13C
23	Acquired Size	32768
24	Spectral Size	32768

Supplementary Figure 119. ^{13}C NMR spectrum of compound 41

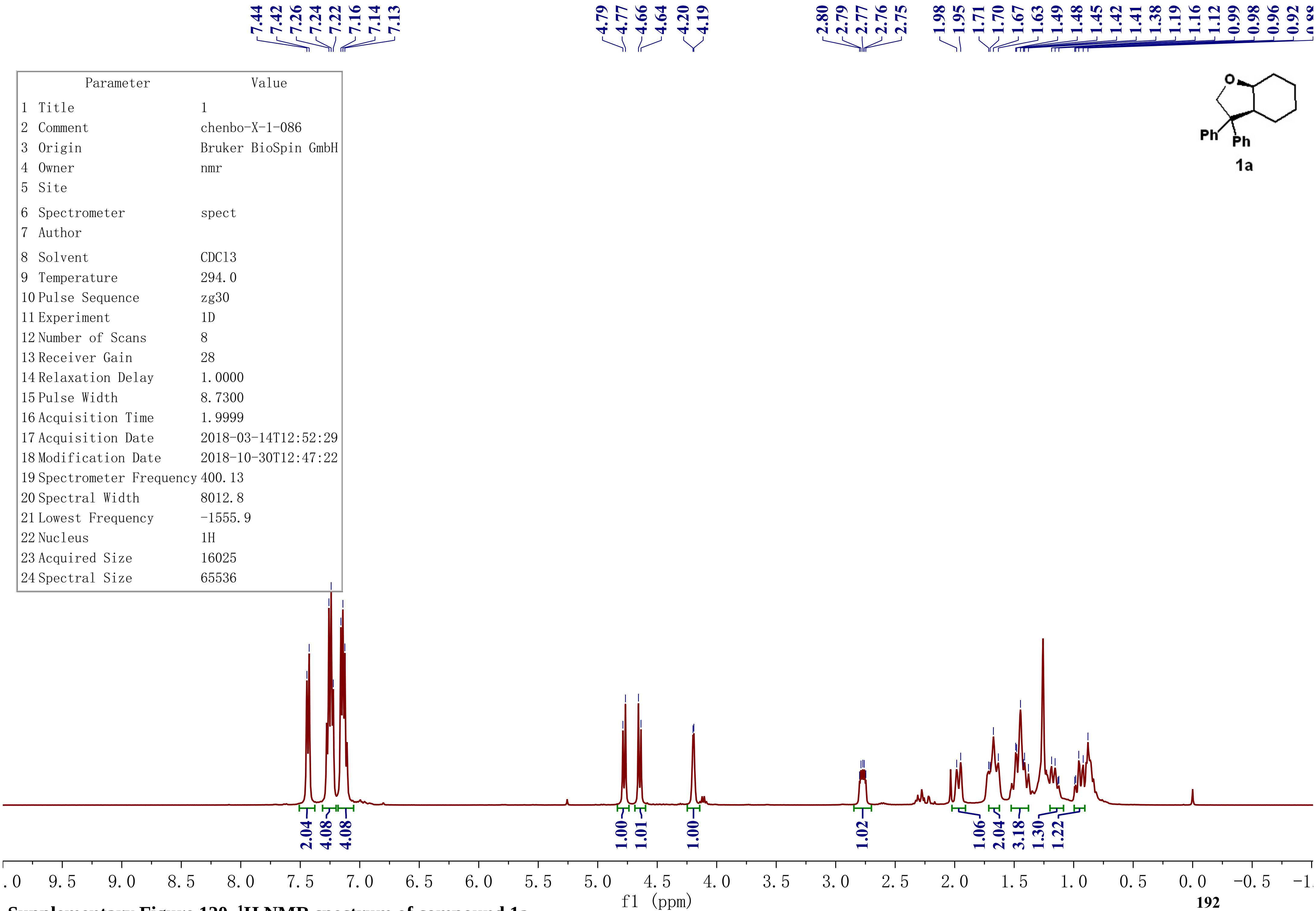

Parameter		Value
1	Title	1
2	Comment	chenbo-X-1-086
3	Origin	Bruker BioSpin GmbH
4	Owner	nmr
5	Site	
6	Spectrometer	spect
7	Author	
8	Solvent	CDCl3
9	Temperature	294.0
10	Pulse Sequence	zg30
11	Experiment	1D
12	Number of Scans	8
13	Receiver Gain	28
14	Relaxation Delay	1.0000
15	Pulse Width	8.7300
16	Acquisition Time	1.9999
17	Acquisition Date	2018-03-14T12:52:29
18	Modification Date	2018-10-30T12:47:22
19	Spectrometer Frequency	400.13
20	Spectral Width	8012.8
21	Lowest Frequency	-1555.9
22	Nucleus	1H
23	Acquired Size	16025
24	Spectral Size	65536

Supplementary Figure 120. ^{1}H NMR spectrum of compound 1a

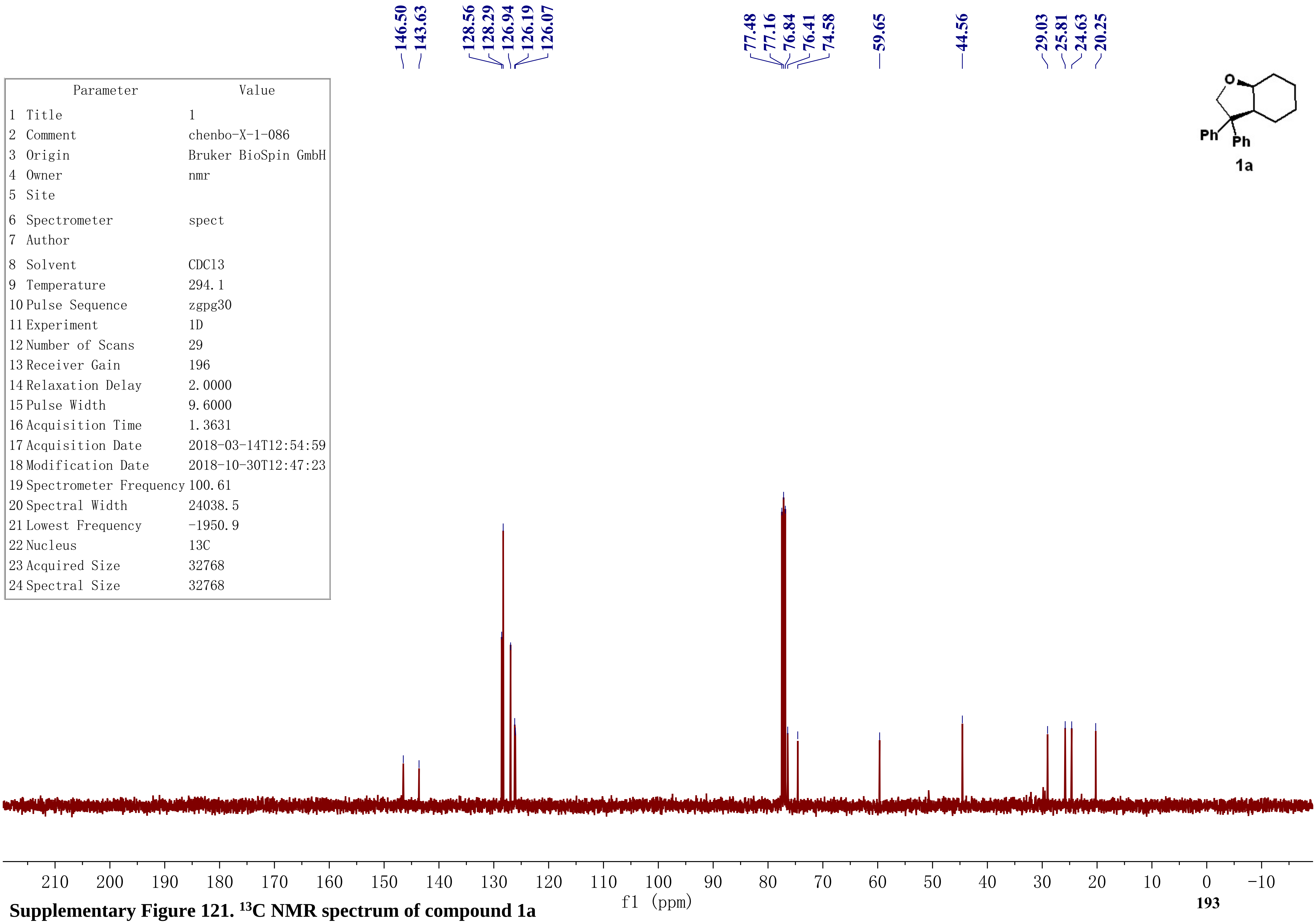

	Parameter	Value
1	Title	1
2	Comment	chenbo-X-1-086
3	Origin	Bruker BioSpin GmbH
4	Owner	nmr
5	Site	
6	Spectrometer	spect
7	Author	
8	Solvent	CDCl3
9	Temperature	294.1
10	Pulse Sequence	zgpg30
11	Experiment	1D
12	Number of Scans	29
13	Receiver Gain	196
14	Relaxation Delay	2.0000
15	Pulse Width	9.6000
16	Acquisition Time	1.3631
17	Acquisition Date	2018-03-14T12:54:59
18	Modification Date	2018-10-30T12:47:23
19	Spectrometer Frequency	100.61
20	Spectral Width	24038.5
21	Lowest Frequency	-1950.9
22	Nucleus	13C
23	Acquired Size	32768
24	Spectral Size	32768

1a

Supplementary Figure 121. ^{13}C **NMR spectrum of compound 1a**

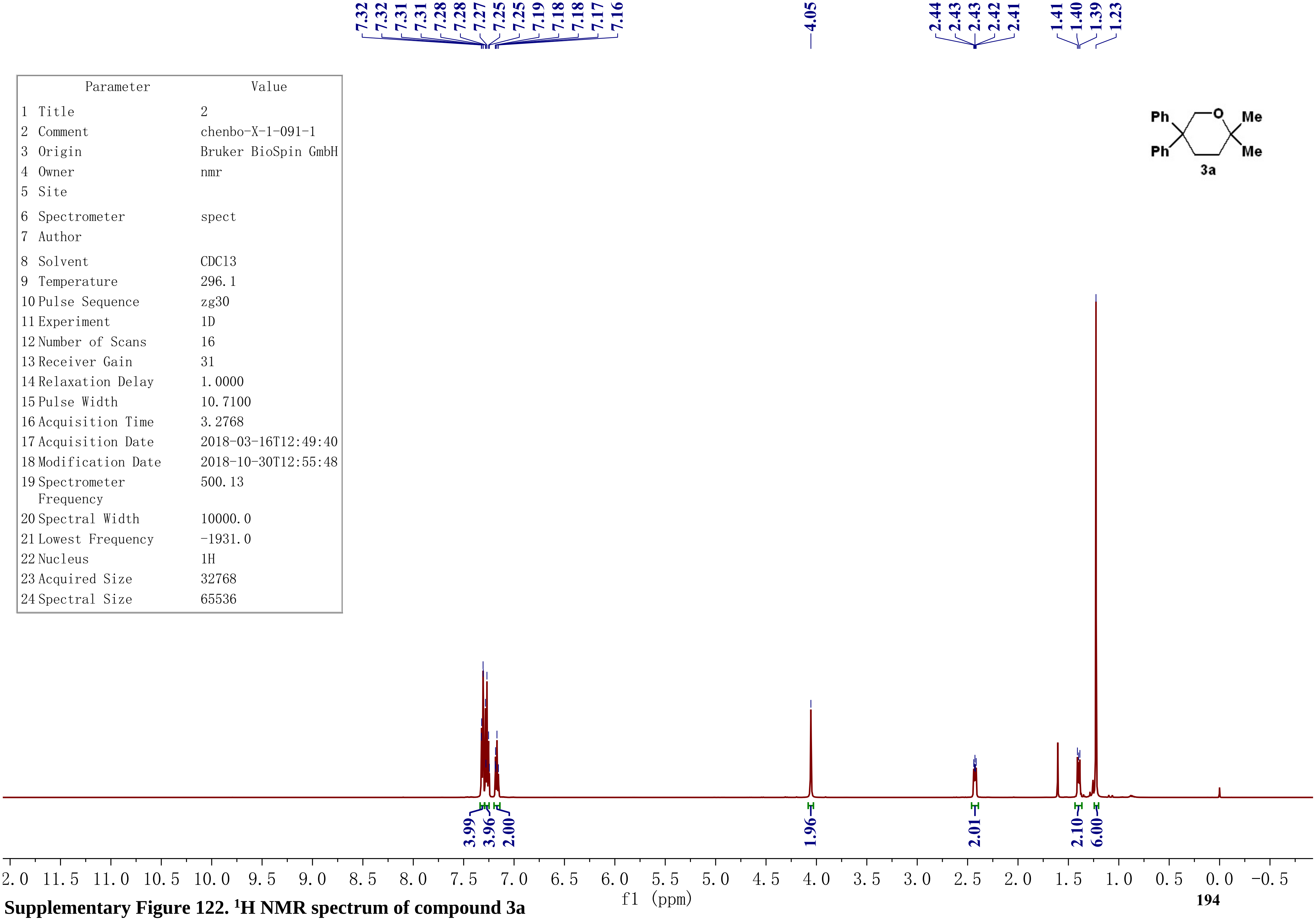

	Parameter	Value
1	Title	2
2	Comment	chenbo-X-1-091-1
3	Origin	Bruker BioSpin GmbH
4	Owner	nmr
5	Site	
6	Spectrometer	spect
7	Author	
8	Solvent	CDCl3
9	Temperature	296.1
10	Pulse Sequence	zg30
11	Experiment	1D
12	Number of Scans	16
13	Receiver Gain	31
14	Relaxation Delay	1.0000
15	Pulse Width	10.7100
16	Acquisition Time	3.2768
17	Acquisition Date	2018-03-16T12:49:40
18	Modification Date	2018-10-30T12:55:48
19	Spectrometer Frequency	500.13
20	Spectral Width	10000.0
21	Lowest Frequency	-1931.0
22	Nucleus	1H
23	Acquired Size	32768
24	Spectral Size	65536

Supplementary Figure 122. ^{1}H NMR spectrum of compound 3a

	Parameter	Value
1	Title	2
2	Comment	chenbo-X-1-091-1
3	Origin	Bruker BioSpin GmbH
4	Owner	nmr
5	Site	
6	Spectrometer	spect
7	Author	
8	Solvent	CDCl3
9	Temperature	296.2
10	Pulse Sequence	zgpg30
11	Experiment	1D
12	Number of Scans	27
13	Receiver Gain	193
14	Relaxation Delay	2.0000
15	Pulse Width	9.6000
16	Acquisition Time	1.1010
17	Acquisition Date	2018-03-16T12:52:00
18	Modification Date	2018-10-30T12:55:49
19	Spectrometer Frequency	125.76
20	Spectral Width	29761.9
21	Lowest Frequency	-2293.6
22	Nucleus	13C
23	Acquired Size	32768
24	Spectral Size	32768

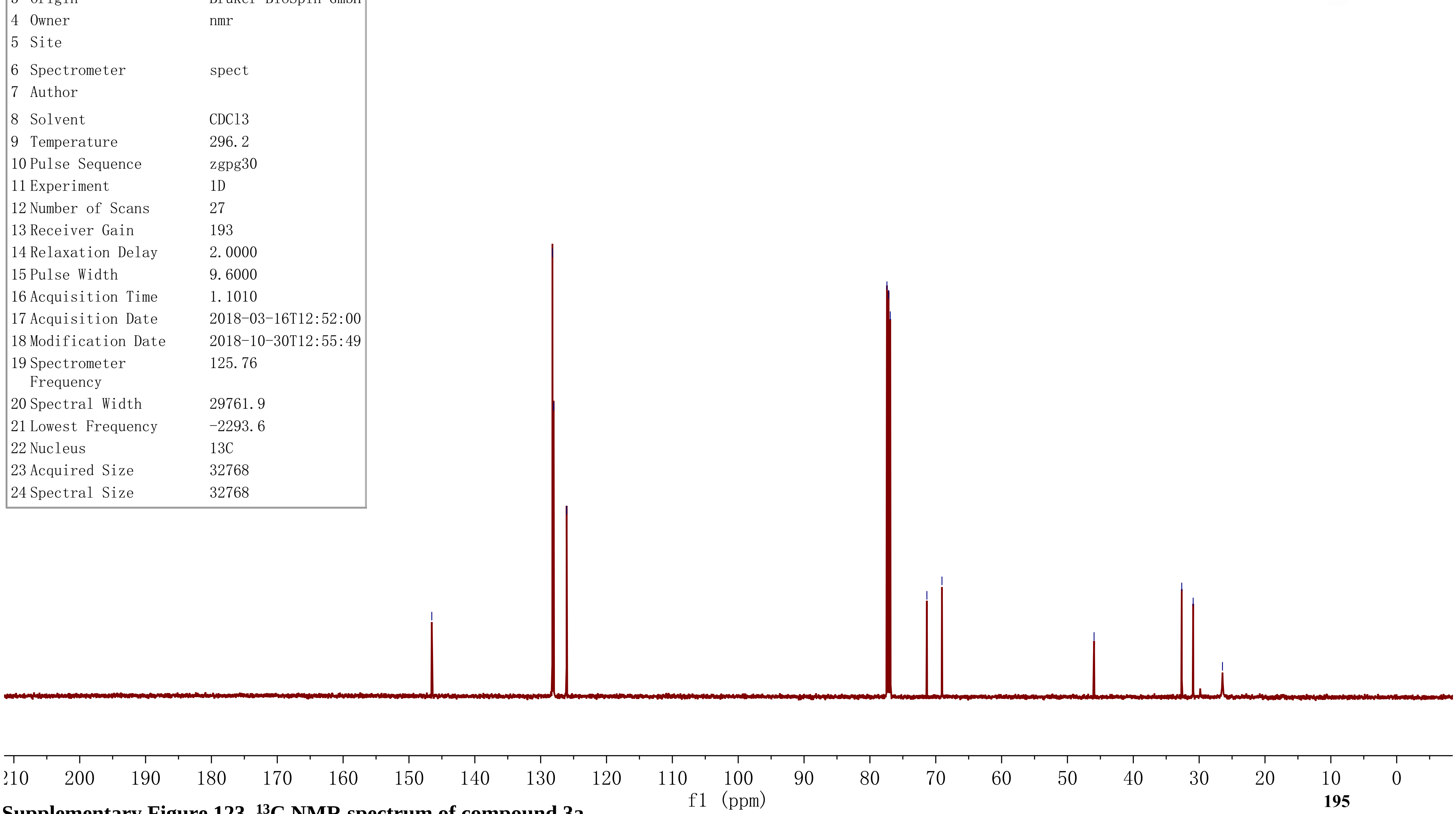

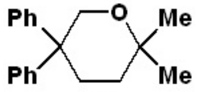

3a

Supplementary Figure 123. ^{13}C NMR spectrum of compound 3a

	Parameter	Value
1	Title	chenbo-X-1-183-1
2	Comment	chenbo-X-1-183-1
3	Origin	Bruker BioSpin GmbH
4	Owner	nmr
5	Site	
6	Spectrometer	AVANCE NEO 400 MHZ DIGITAL NMR SPECTROMETER
7	Author	
8	Solvent	CDCl3
9	Temperature	293.3
10	Pulse Sequence	zg30
11	Experiment	1D
12	Number of Scans	16
13	Receiver Gain	12
14	Relaxation Delay	1.0000
15	Pulse Width	10.0000
16	Acquisition Time	3.9977
17	Acquisition Date	2018-06-02T13:46:13
18	Modification Date	2018-10-30T12:55:50
19	Spectrometer Frequency	400.13
20	Spectral Width	8196.7
21	Lowest Frequency	-1638.2
22	Nucleus	1H
23	Acquired Size	32768
24	Spectral Size	65536

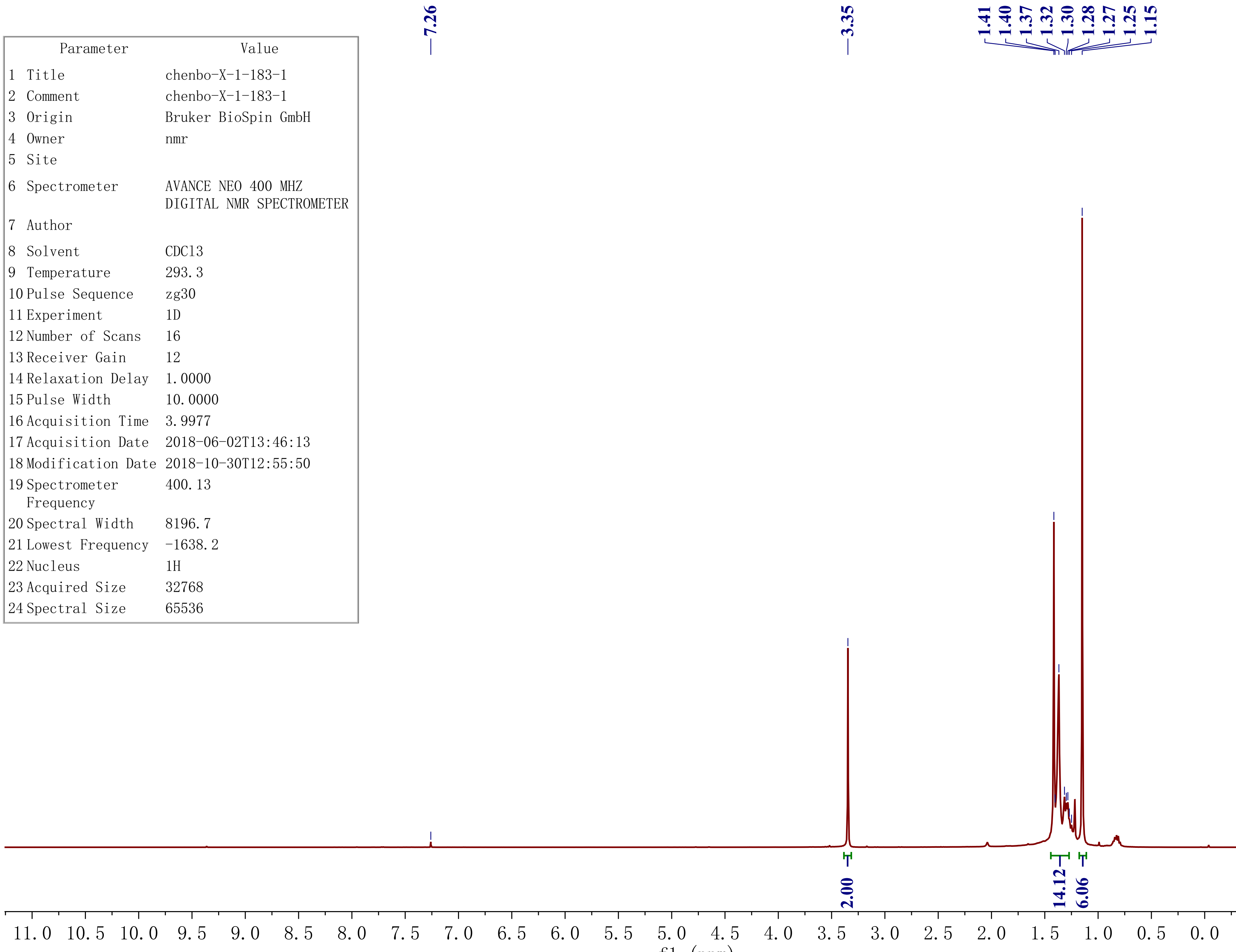

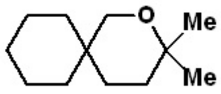

4a

Supplementary Figure 124. ^{1}H NMR spectrum of compound 4a

Parameter		Value
1	Title	chenbo-X-1-183-1
2	Comment	chenbo-X-1-183-1
3	Origin	Bruker BioSpin GmbH
4	Owner	nmr
5	Site	
6	Spectrometer	AVANCE NEO 400 MHZ DIGITAL NMR SPECTROMETER
7	Author	
8	Solvent	CDCl3
9	Temperature	293.7
10	Pulse Sequence	zgpg30
11	Experiment	1D
12	Number of Scans	22
13	Receiver Gain	29
14	Relaxation Delay	2.0000
15	Pulse Width	10.0000
16	Acquisition Time	1.3763
17	Acquisition Date	2018-06-02T13:48:36
18	Modification Date	2018-10-30T12:55:51
19	Spectrometer Frequency	100.61
20	Spectral Width	23809.5
21	Lowest Frequency	-1835.1
22	Nucleus	13C
23	Acquired Size	32768
24	Spectral Size	32768

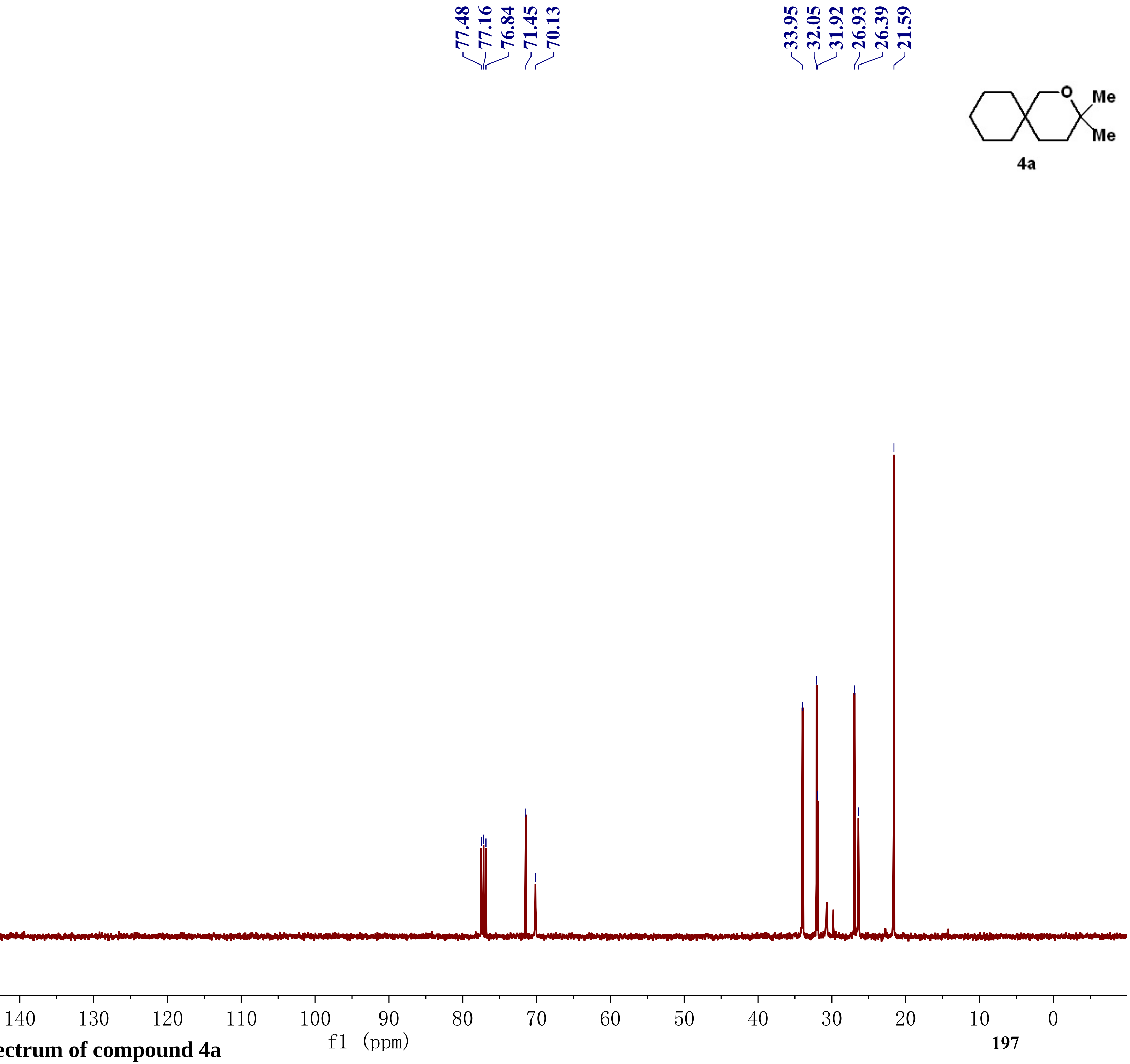

4a

Supplementary Figure 125. ^{13}C NMR spectrum of compound 4a

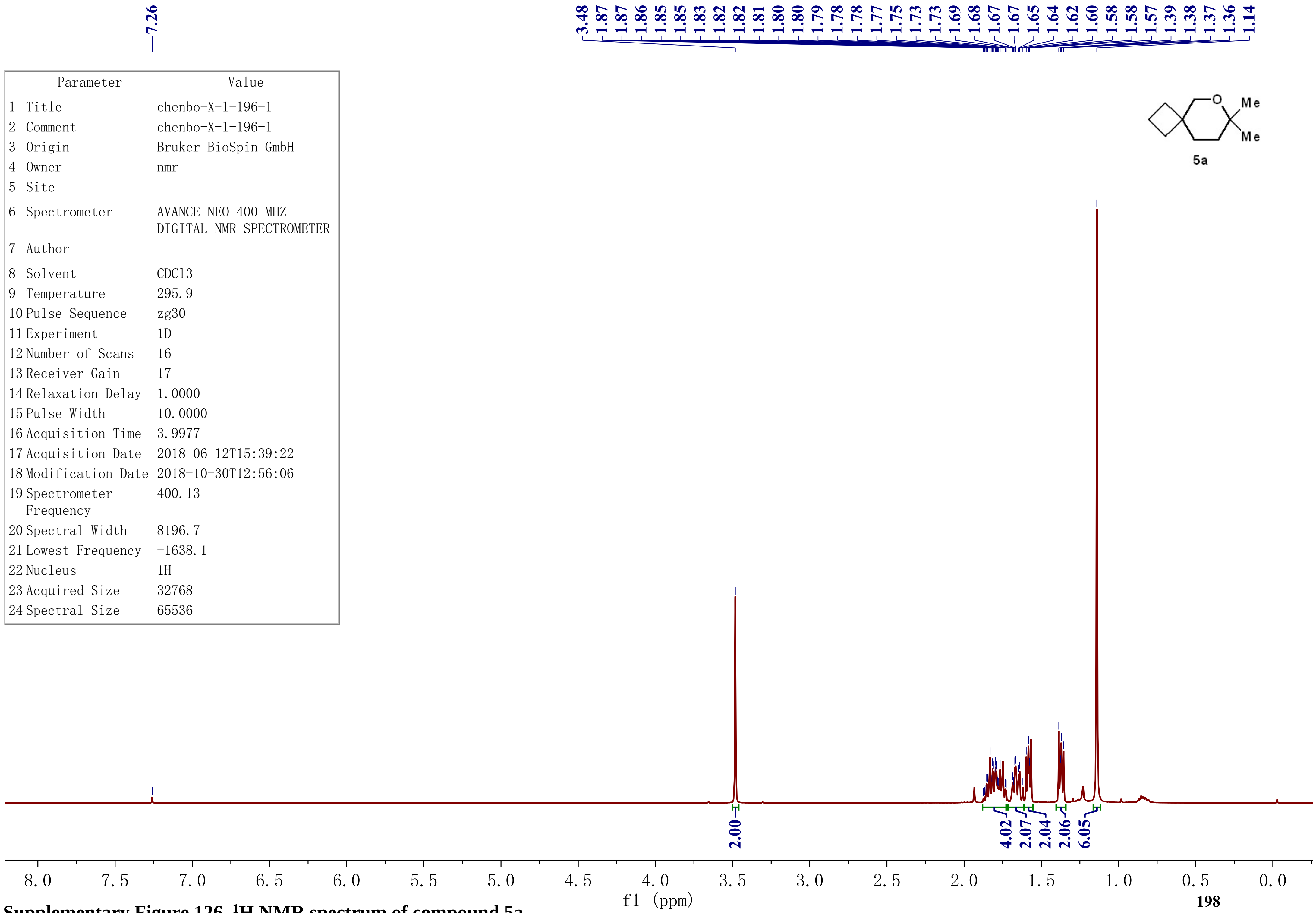

	Parameter	Value
1	Title	chenbo-X-1-196-1
2	Comment	chenbo-X-1-196-1
3	Origin	Bruker BioSpin GmbH
4	Owner	nmr
5	Site	
6	Spectrometer	AVANCE NEO 400 MHZ DIGITAL NMR SPECTROMETER
7	Author	
8	Solvent	CDCl3
9	Temperature	295.9
10	Pulse Sequence	zg30
11	Experiment	1D
12	Number of Scans	16
13	Receiver Gain	17
14	Relaxation Delay	1.0000
15	Pulse Width	10.0000
16	Acquisition Time	3.9977
17	Acquisition Date	2018-06-12T15:39:22
18	Modification Date	2018-10-30T12:56:06
19	Spectrometer Frequency	400.13
20	Spectral Width	8196.7
21	Lowest Frequency	-1638.1
22	Nucleus	1H
23	Acquired Size	32768
24	Spectral Size	65536

Supplementary Figure 126. ^{1}H NMR spectrum of compound 5a

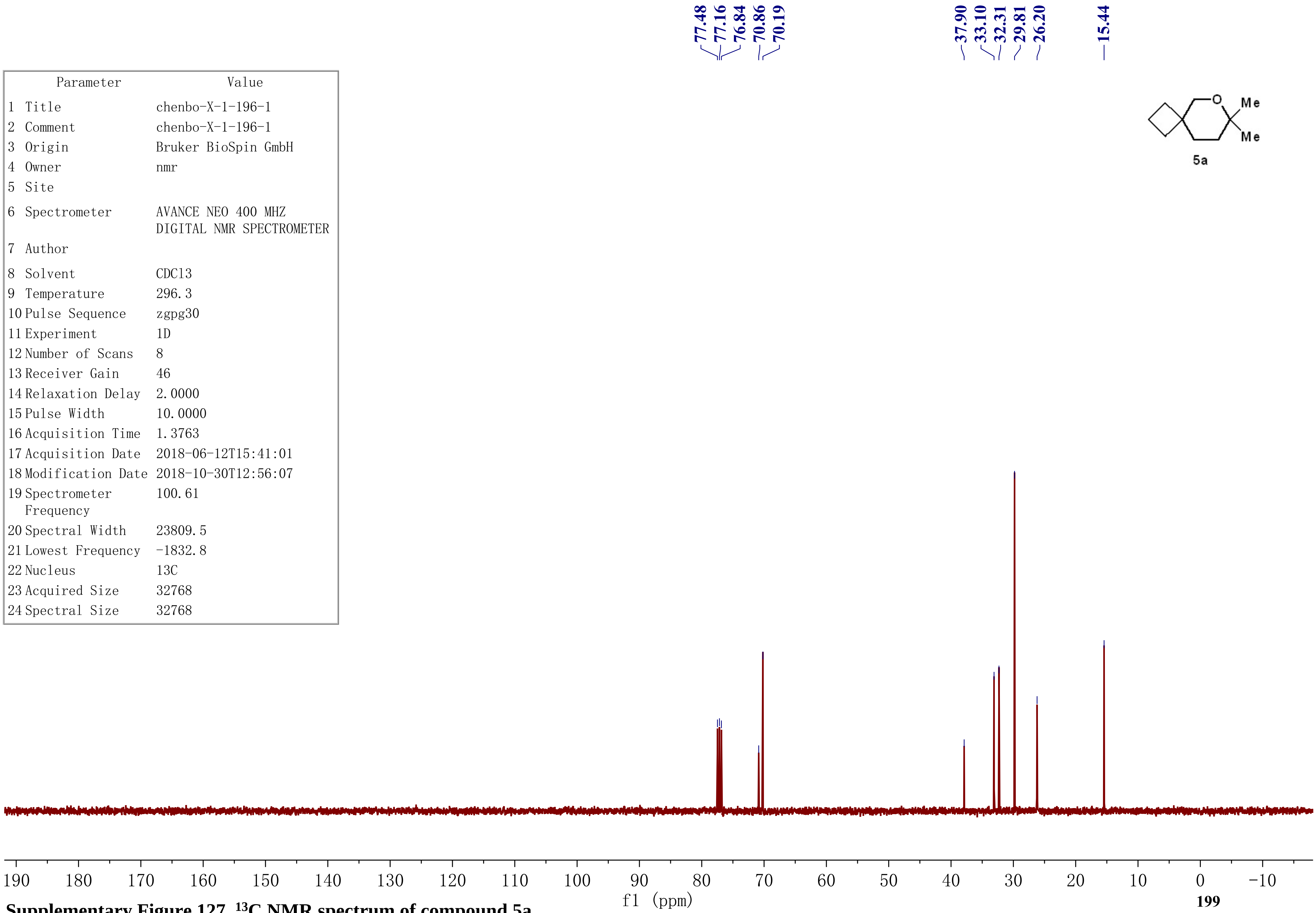

	Parameter	Value
1	Title	chenbo-X-1-196-1
2	Comment	chenbo-X-1-196-1
3	Origin	Bruker BioSpin GmbH
4	Owner	nmr
5	Site	
6	Spectrometer	AVANCE NEO 400 MHZ DIGITAL NMR SPECTROMETER
7	Author	
8	Solvent	CDCl3
9	Temperature	296.3
10	Pulse Sequence	zgpg30
11	Experiment	1D
12	Number of Scans	8
13	Receiver Gain	46
14	Relaxation Delay	2.0000
15	Pulse Width	10.0000
16	Acquisition Time	1.3763
17	Acquisition Date	2018-06-12T15:41:01
18	Modification Date	2018-10-30T12:56:07
19	Spectrometer Frequency	100.61
20	Spectral Width	23809.5
21	Lowest Frequency	-1832.8
22	Nucleus	13C
23	Acquired Size	32768
24	Spectral Size	32768

Supplementary Figure 127. ^{13}C NMR spectrum of compound 5a

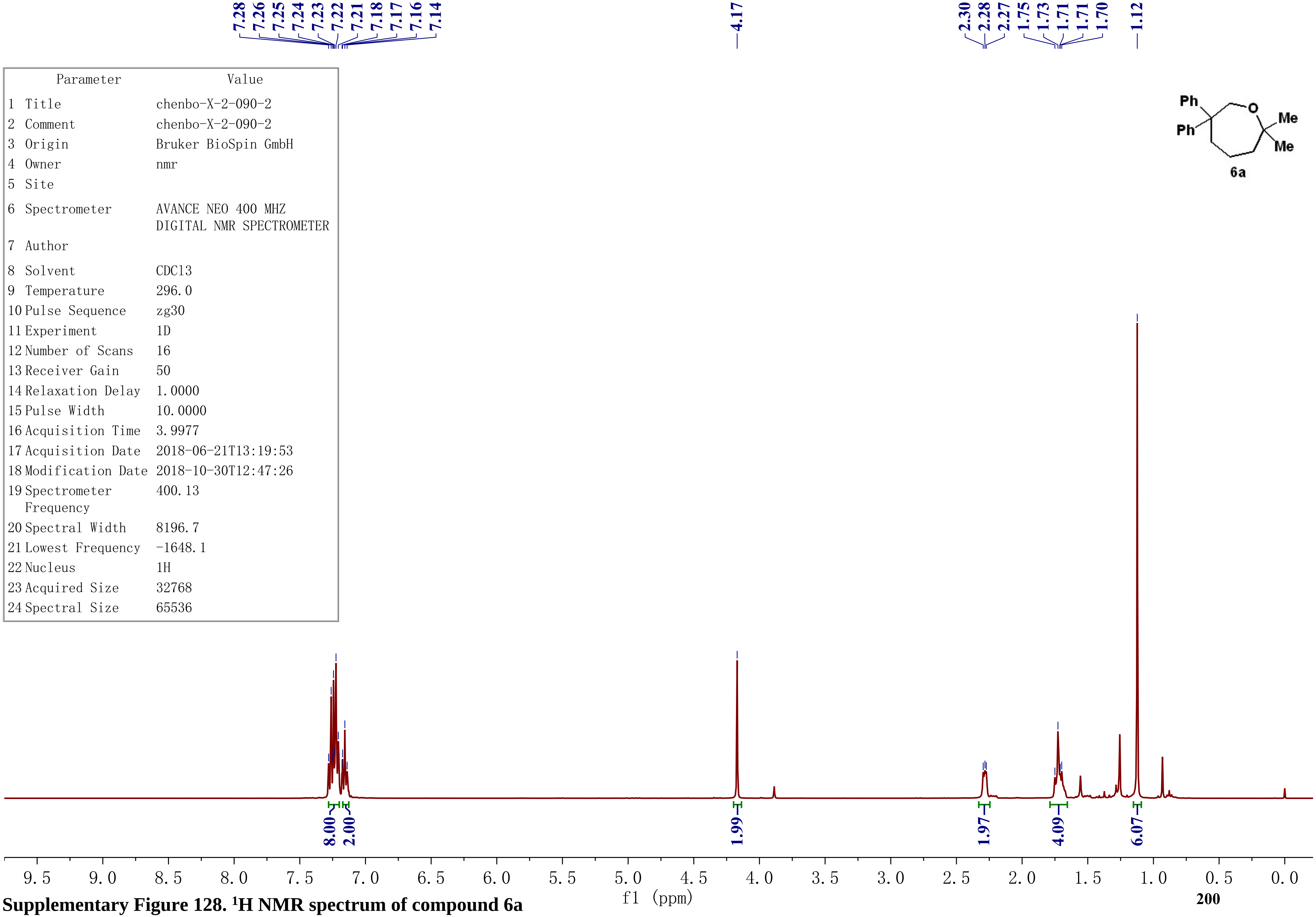

	Parameter	Value
1	Title	chenbo-X-2-090-2
2	Comment	chenbo-X-2-090-2
3	Origin	Bruker BioSpin GmbH
4	Owner	nmr
5	Site	
6	Spectrometer	AVANCE NEO 400 MHZ DIGITAL NMR SPECTROMETER
7	Author	
8	Solvent	CDCl3
9	Temperature	296.0
10	Pulse Sequence	zg30
11	Experiment	1D
12	Number of Scans	16
13	Receiver Gain	50
14	Relaxation Delay	1.0000
15	Pulse Width	10.0000
16	Acquisition Time	3.9977
17	Acquisition Date	2018-06-21T13:19:53
18	Modification Date	2018-10-30T12:47:26
19	Spectrometer Frequency	400.13
20	Spectral Width	8196.7
21	Lowest Frequency	-1648.1
22	Nucleus	1H
23	Acquired Size	32768
24	Spectral Size	65536

Supplementary Figure 128. ^{1}H NMR spectrum of compound 6a

	Parameter	Value
1	Title	chenbo-X-2-090-2
2	Comment	chenbo-X-2-090-2
3	Origin	Bruker BioSpin GmbH
4	Owner	nmr
5	Site	
6	Spectrometer	AVANCE NEO 400 MHZ DIGITAL NMR SPECTROMETER
7	Author	
8	Solvent	CDCl3
9	Temperature	296.4
10	Pulse Sequence	zgpg30
11	Experiment	1D
12	Number of Scans	40
13	Receiver Gain	48
14	Relaxation Delay	2.0000
15	Pulse Width	10.0000
16	Acquisition Time	1.3763
17	Acquisition Date	2018-06-21T13:23:24
18	Modification Date	2018-10-30T12:47:27
19	Spectrometer Frequency	100.61
20	Spectral Width	23809.5
21	Lowest Frequency	-1834.5
22	Nucleus	13C
23	Acquired Size	32768
24	Spectral Size	32768

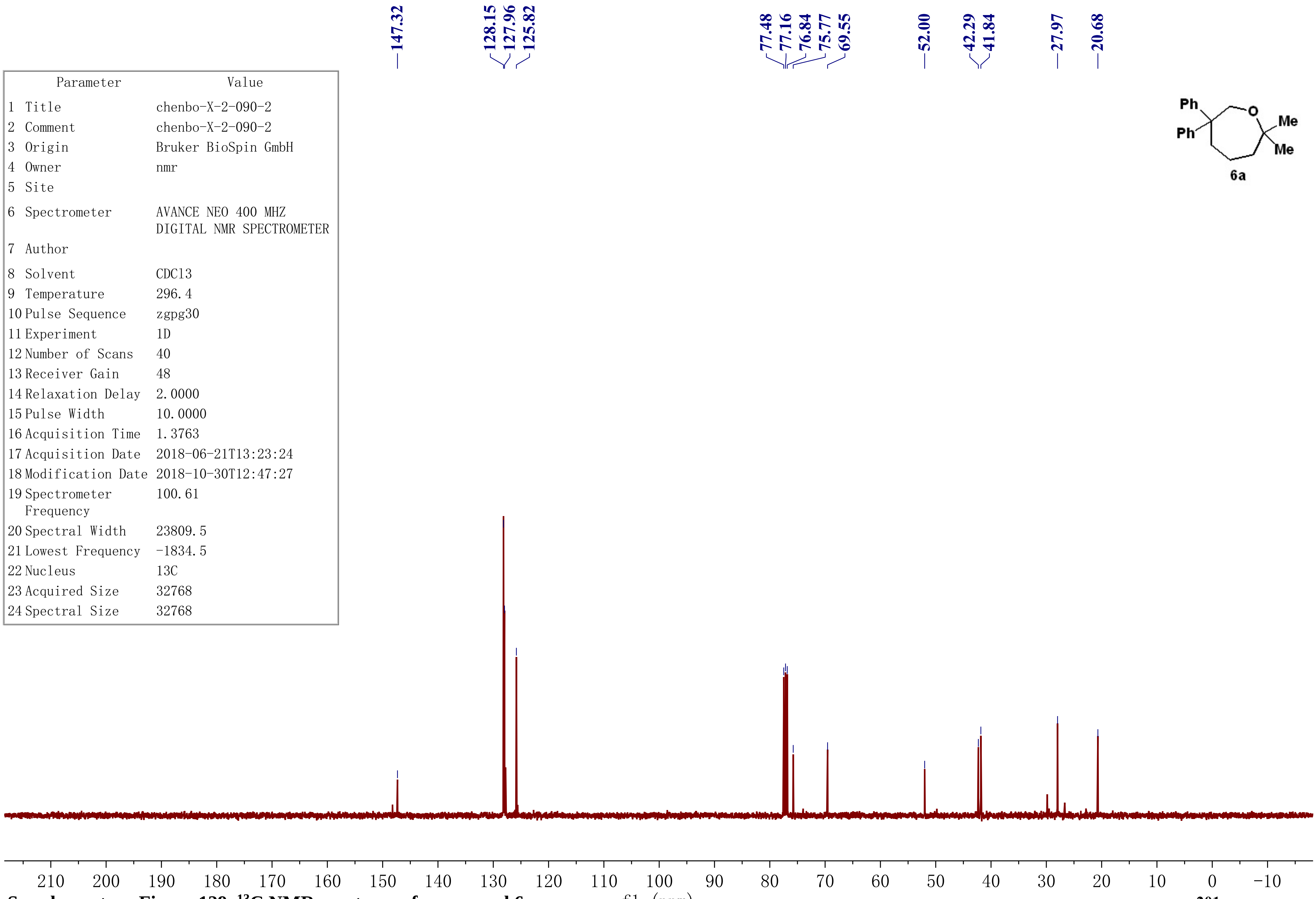

6a

Supplementary Figure 129. ^{13}**C NMR spectrum of compound 6a**

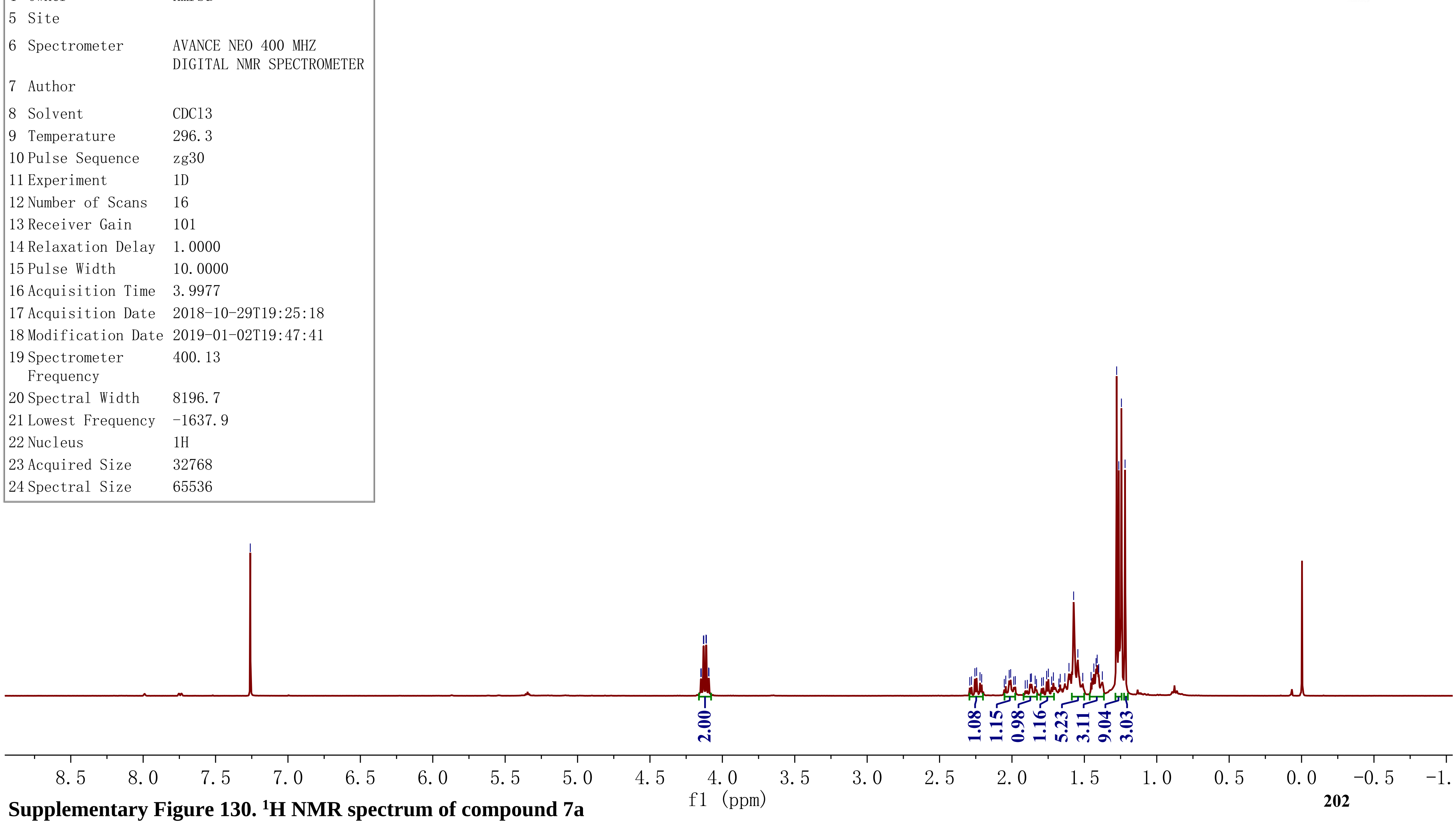

	Parameter	Value
1	Title	XJ1-195
2	Comment	
3	Origin	Bruker BioSpin GmbH
4	Owner	nmrsu
5	Site	
6	Spectrometer	AVANCE NEO 400 MHZ DIGITAL NMR SPECTROMETER
7	Author	
8	Solvent	CDCl3
9	Temperature	296.3
10	Pulse Sequence	zg30
11	Experiment	1D
12	Number of Scans	16
13	Receiver Gain	101
14	Relaxation Delay	1.0000
15	Pulse Width	10.0000
16	Acquisition Time	3.9977
17	Acquisition Date	2018-10-29T19:25:18
18	Modification Date	2019-01-02T19:47:41
19	Spectrometer Frequency	400.13
20	Spectral Width	8196.7
21	Lowest Frequency	-1637.9
22	Nucleus	1H
23	Acquired Size	32768
24	Spectral Size	65536

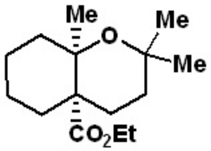

7a

Supplementary Figure 130. ^{1}H NMR spectrum of compound 7a

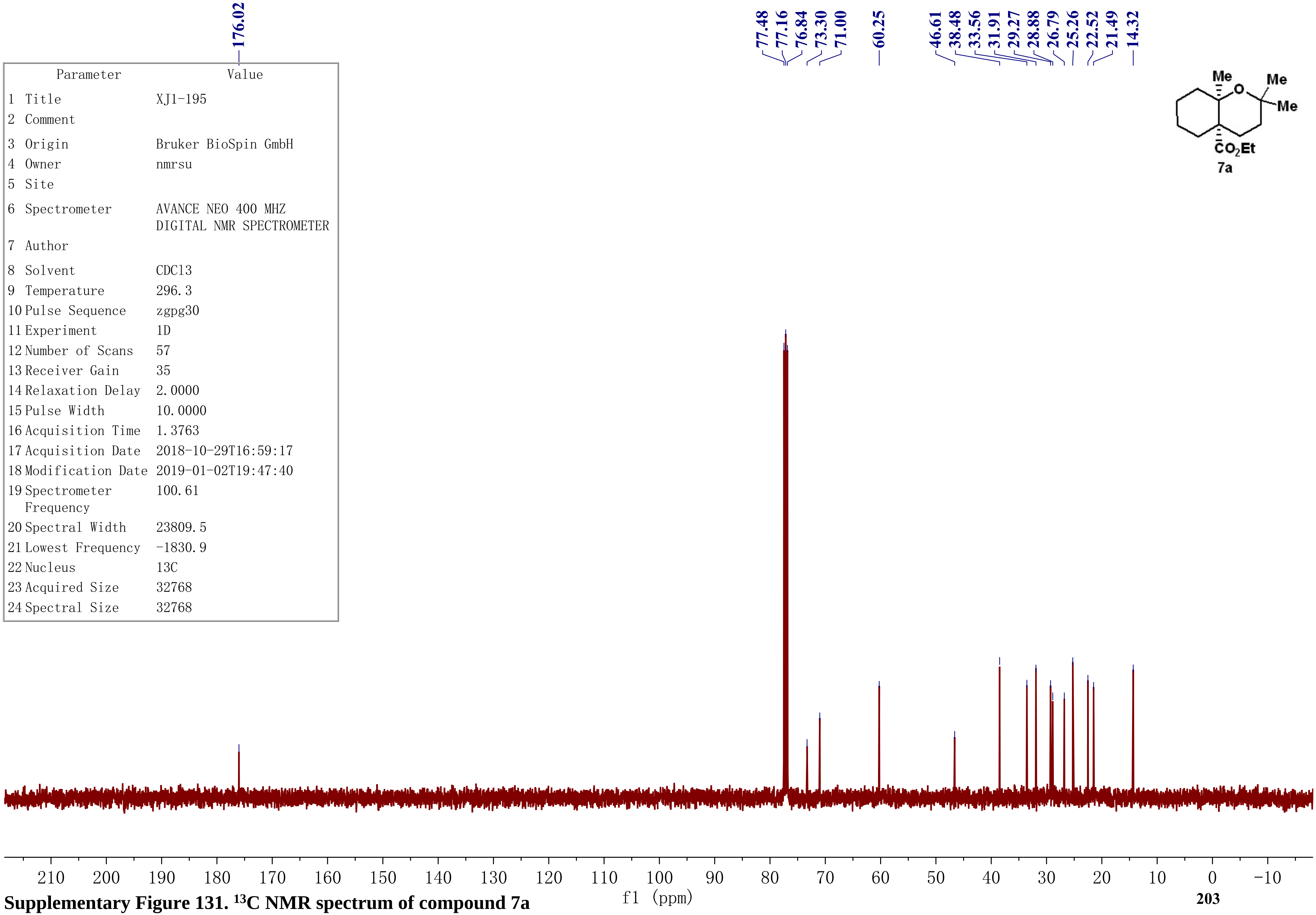

7a

	Parameter	Value
1	Title	XJ1-195
2	Comment	
3	Origin	Bruker BioSpin GmbH
4	Owner	nmrsu
5	Site	
6	Spectrometer	AVANCE NEO 400 MHZ DIGITAL NMR SPECTROMETER
7	Author	
8	Solvent	CDCl3
9	Temperature	296.3
10	Pulse Sequence	zgpg30
11	Experiment	1D
12	Number of Scans	57
13	Receiver Gain	35
14	Relaxation Delay	2.0000
15	Pulse Width	10.0000
16	Acquisition Time	1.3763
17	Acquisition Date	2018-10-29T16:59:17
18	Modification Date	2019-01-02T19:47:40
19	Spectrometer Frequency	100.61
20	Spectral Width	23809.5
21	Lowest Frequency	-1830.9
22	Nucleus	13C
23	Acquired Size	32768
24	Spectral Size	32768

Supplementary Figure 131. ^{13}C NMR spectrum of compound 7a

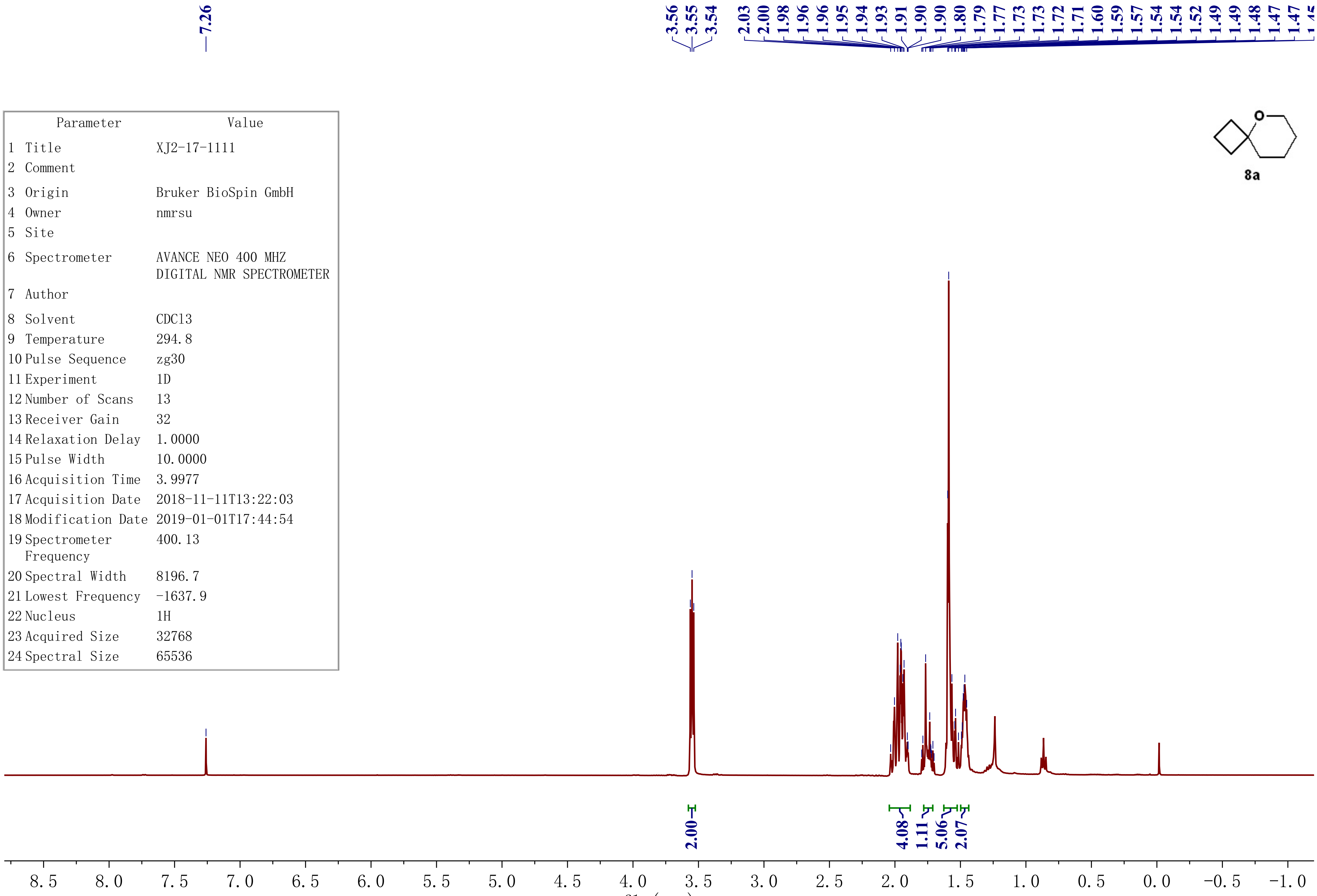

Parameter	Value
1 Title	XJ2-17-1111
2 Comment	
3 Origin	Bruker BioSpin GmbH
4 Owner	nmrsu
5 Site	
6 Spectrometer	AVANCE NEO 400 MHZ DIGITAL NMR SPECTROMETER
7 Author	
8 Solvent	CDCl3
9 Temperature	294.8
10 Pulse Sequence	zg30
11 Experiment	1D
12 Number of Scans	13
13 Receiver Gain	32
14 Relaxation Delay	1.0000
15 Pulse Width	10.0000
16 Acquisition Time	3.9977
17 Acquisition Date	2018-11-11T13:22:03
18 Modification Date	2019-01-01T17:44:54
19 Spectrometer Frequency	400.13
20 Spectral Width	8196.7
21 Lowest Frequency	-1637.9
22 Nucleus	1H
23 Acquired Size	32768
24 Spectral Size	65536

Supplementary Figure 132. ^{1}H NMR spectrum of compound 8a

	Parameter	Value
1	Title	XJ2-17-1111
2	Comment	
3	Origin	Bruker BioSpin GmbH
4	Owner	nmrsu
5	Site	
6	Spectrometer	AVANCE NEO 400 MHZ DIGITAL NMR SPECTROMETER
7	Author	
8	Solvent	CDCl3
9	Temperature	296.1
10	Pulse Sequence	zgpg30
11	Experiment	1D
12	Number of Scans	10
13	Receiver Gain	50
14	Relaxation Delay	2.0000
15	Pulse Width	10.0000
16	Acquisition Time	1.3763
17	Acquisition Date	2018-11-11T13:49:03
18	Modification Date	2019-01-01T17:44:58
19	Spectrometer Frequency	100.61
20	Spectral Width	23809.5
21	Lowest Frequency	-1831.7
22	Nucleus	13C
23	Acquired Size	32768
24	Spectral Size	32768

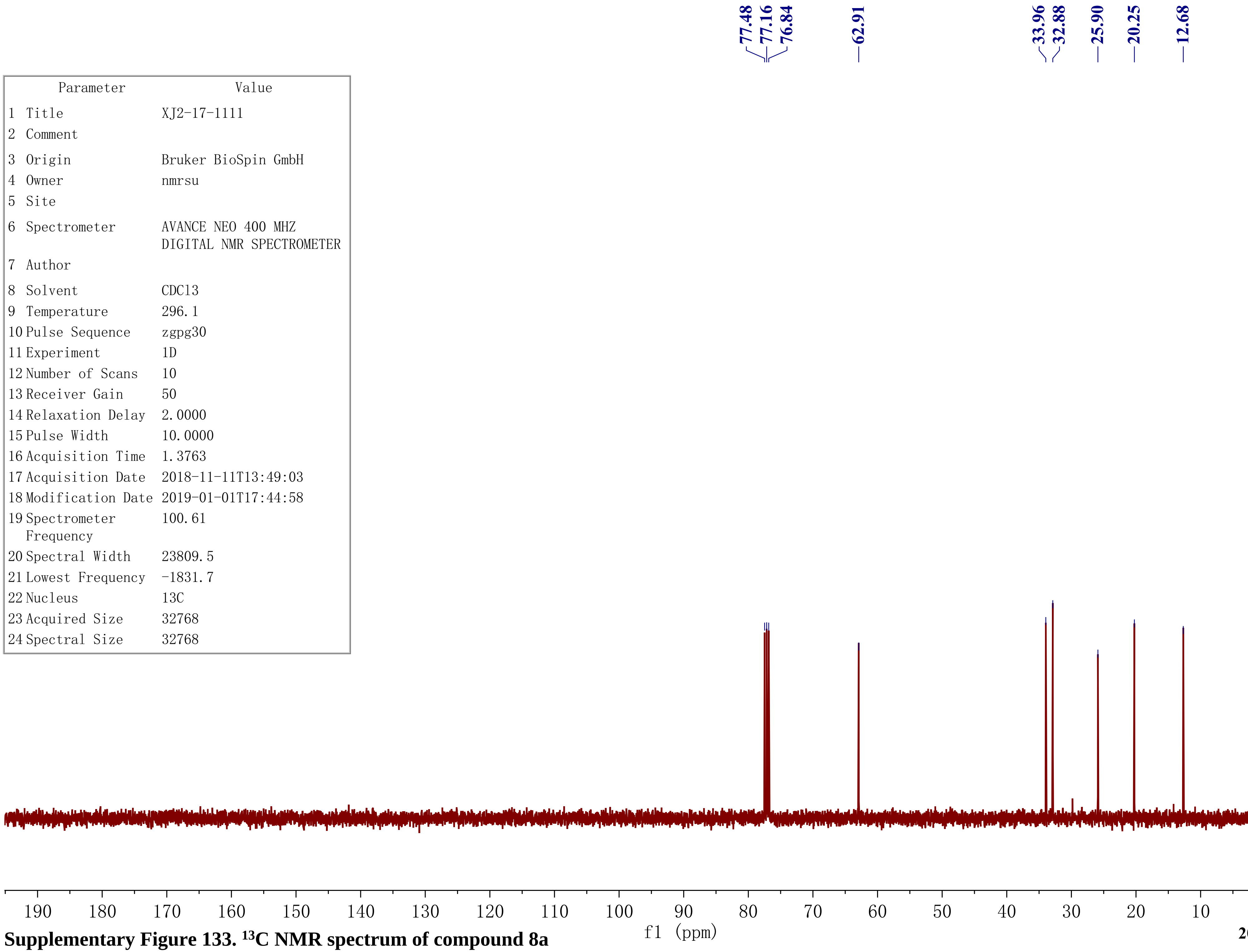

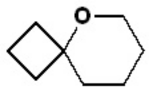

8a

Supplementary Figure 133. ^{13}C NMR spectrum of compound 8a

Parameter	Value
1 Title	ZYL226-1
2 Comment	
3 Origin	Bruker BioSpin GmbH
4 Owner	nmrsu
5 Site	
6 Spectrometer	Avance NEO 600
7 Author	
8 Solvent	CDCl3
9 Temperature	297.1
10 Pulse Sequence	zg30
11 Experiment	1D
12 Number of Scans	8
13 Receiver Gain	32
14 Relaxation Delay	1.0000
15 Pulse Width	10.0000
16 Acquisition Time	2.7525
17 Acquisition Date	2018-11-21T16:48:59
18 Modification Date	2018-11-21T17:05:58
19 Spectrometer Frequency	600.15
20 Spectral Width	11904.8
21 Lowest Frequency	-2314.6
22 Nucleus	1H
23 Acquired Size	32768
24 Spectral Size	65536

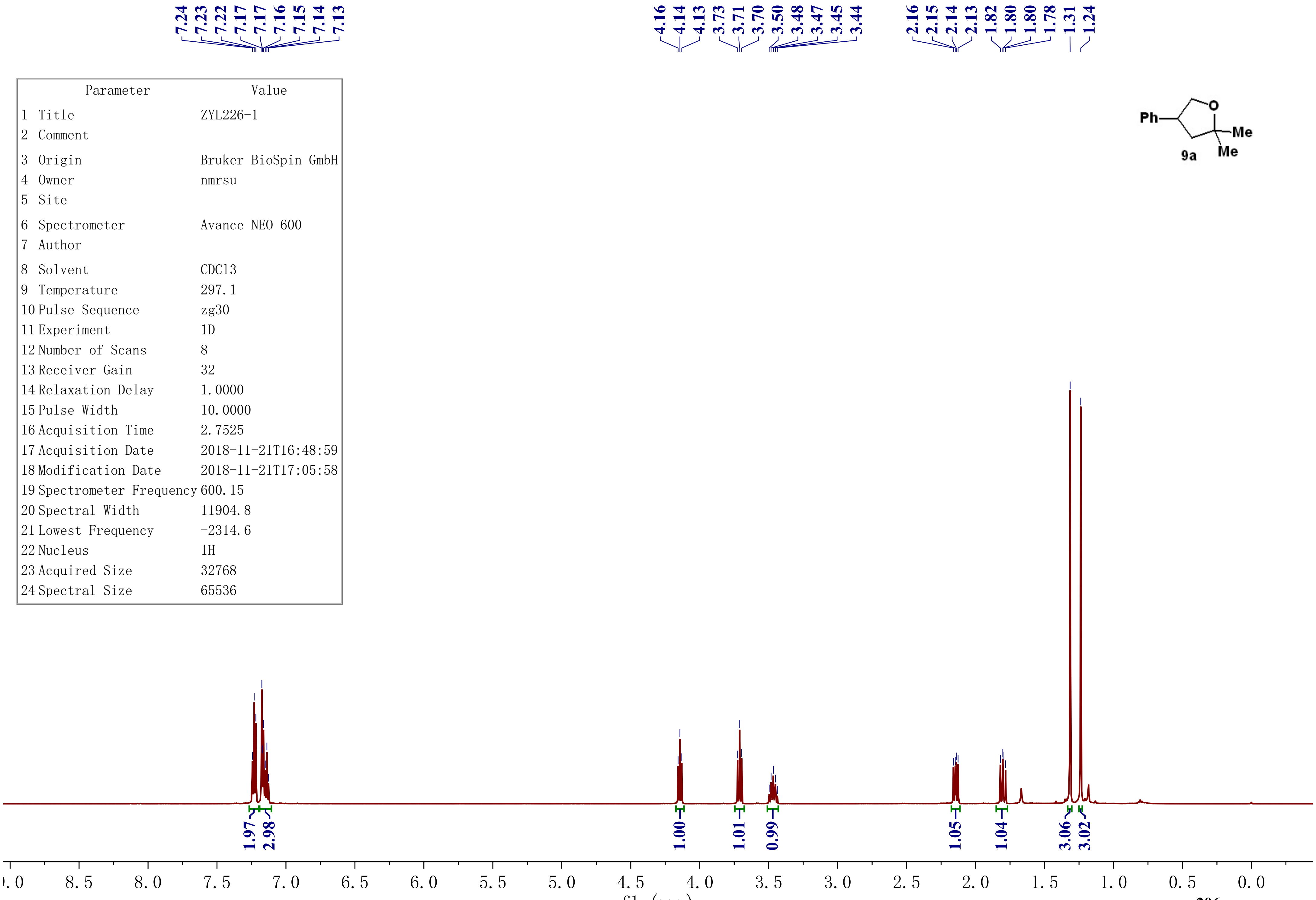

Supplementary Figure 134. ^{1}H NMR spectrum of compound 9a

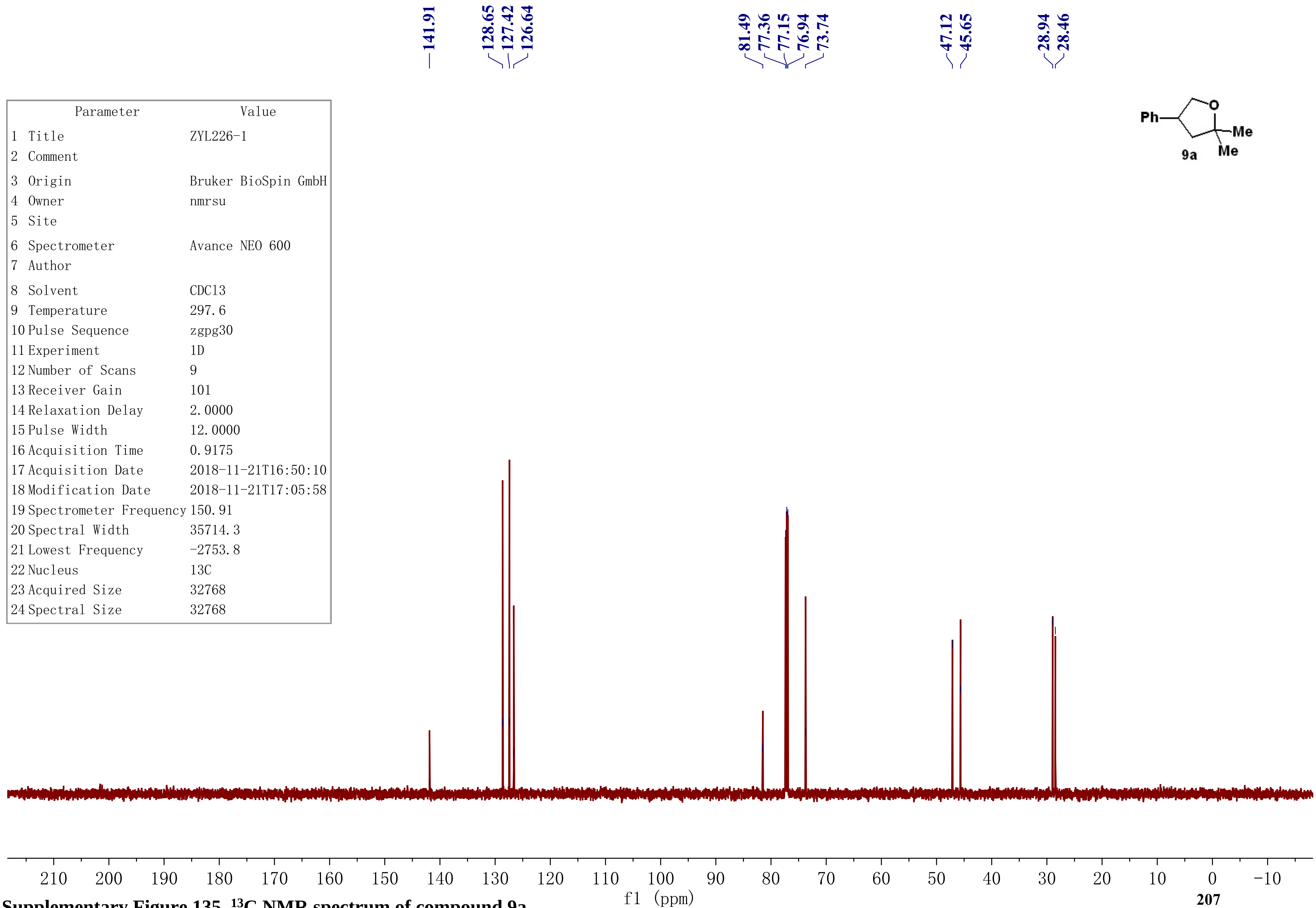

Supplementary Figure 135. ^{13}C NMR spectrum of compound 9a

	Parameter	Value
1	Title	ZYL183C
2	Comment	
3	Origin	Bruker BioSpin GmbH
4	Owner	nmrsu
5	Site	
6	Spectrometer	AVANCE NEO 400 MHZ DIGITAL NMR SPECTROMETER
7	Author	
8	Solvent	CDCl3
9	Temperature	295.1
10	Pulse Sequence	zg30
11	Experiment	1D
12	Number of Scans	16
13	Receiver Gain	57
14	Relaxation Delay	1.0000
15	Pulse Width	10.0000
16	Acquisition Time	3.9977
17	Acquisition Date	2018-11-01T15:12:01
18	Modification Date	2018-11-01T15:27:07
19	Spectrometer Frequency	400.13
20	Spectral Width	8196.7
21	Lowest Frequency	-1672.3
22	Nucleus	1H
23	Acquired Size	32768
24	Spectral Size	65536

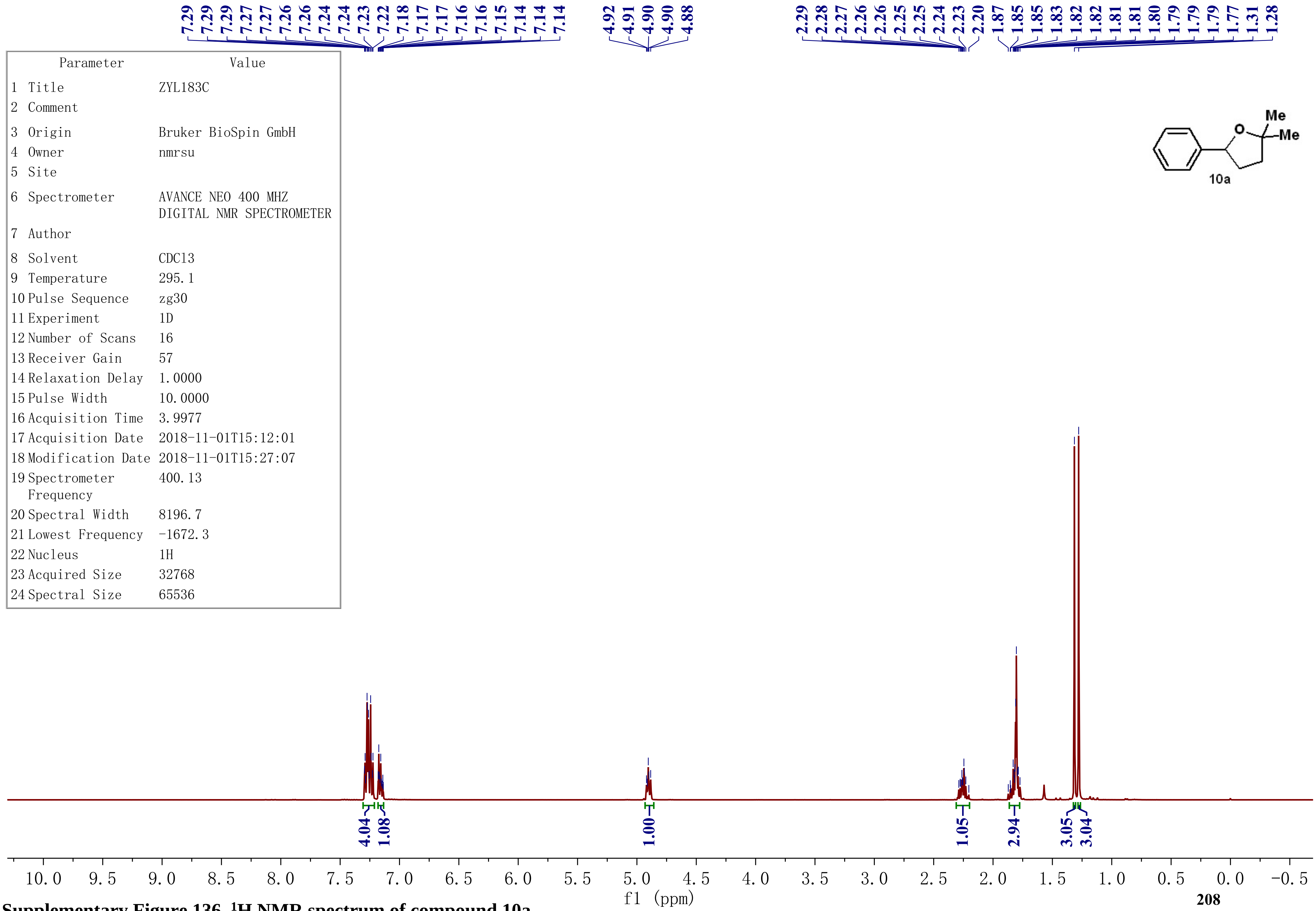

Supplementary Figure 136. ^{1}H NMR spectrum of compound 10a

	Parameter	Value
1	Title	ZYL183C
2	Comment	
3	Origin	Bruker BioSpin GmbH
4	Owner	nmrsu
5	Site	
6	Spectrometer	AVANCE NEO 400 MHZ DIGITAL NMR SPECTROMETER
7	Author	
8	Solvent	CDCl3
9	Temperature	295.5
10	Pulse Sequence	zgpg30
11	Experiment	1D
12	Number of Scans	22
13	Receiver Gain	32
14	Relaxation Delay	2.0000
15	Pulse Width	10.0000
16	Acquisition Time	1.3763
17	Acquisition Date	2018-11-01T15:14:20
18	Modification Date	2018-11-01T15:27:08
19	Spectrometer Frequency	100.61
20	Spectral Width	23809.5
21	Lowest Frequency	-1834.2
22	Nucleus	13C
23	Acquired Size	32768
24	Spectral Size	32768

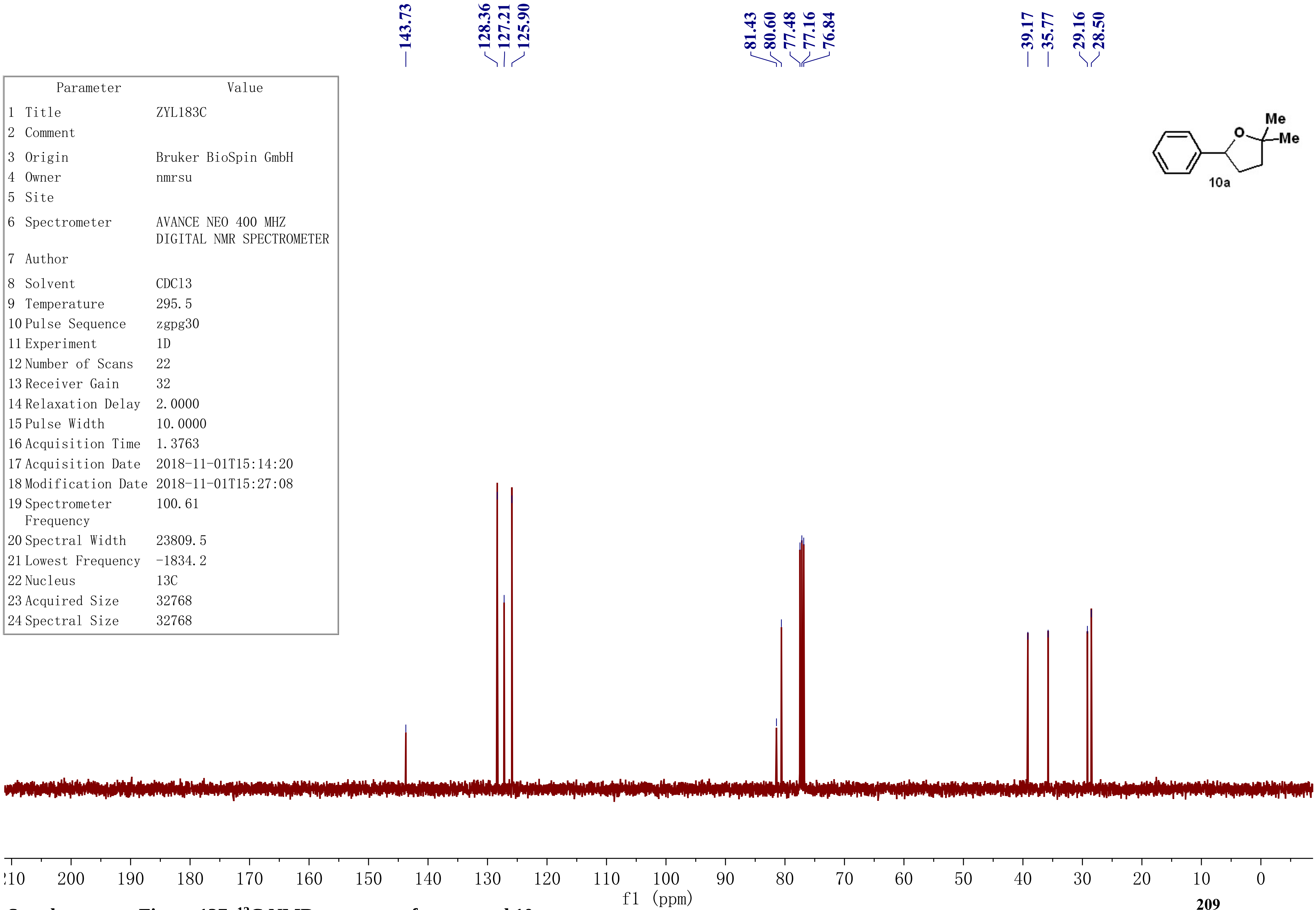

10a

Supplementary Figure 137. ^{13}C NMR spectrum of compound 10a

Parameter	Value
1 Title	chenbo-X-1-190
2 Comment	chenbo-X-1-190
3 Origin	Bruker BioSpin GmbH
4 Owner	nmr
5 Site	
6 Spectrometer	AVANCE NEO 400 MHZ DIGITAL NMR SPECTROMETER
7 Author	
8 Solvent	CDCl3
9 Temperature	294.4
10 Pulse Sequence	zg30
11 Experiment	1D
12 Number of Scans	16
13 Receiver Gain	13
14 Relaxation Delay	1.0000
15 Pulse Width	10.0000
16 Acquisition Time	3.9977
17 Acquisition Date	2018-06-08T10:42:23
18 Modification Date	2018-10-30T12:55:57
19 Spectrometer Frequency	400.13
20 Spectral Width	8196.7
21 Lowest Frequency	-1637.9
22 Nucleus	1H
23 Acquired Size	32768
24 Spectral Size	65536

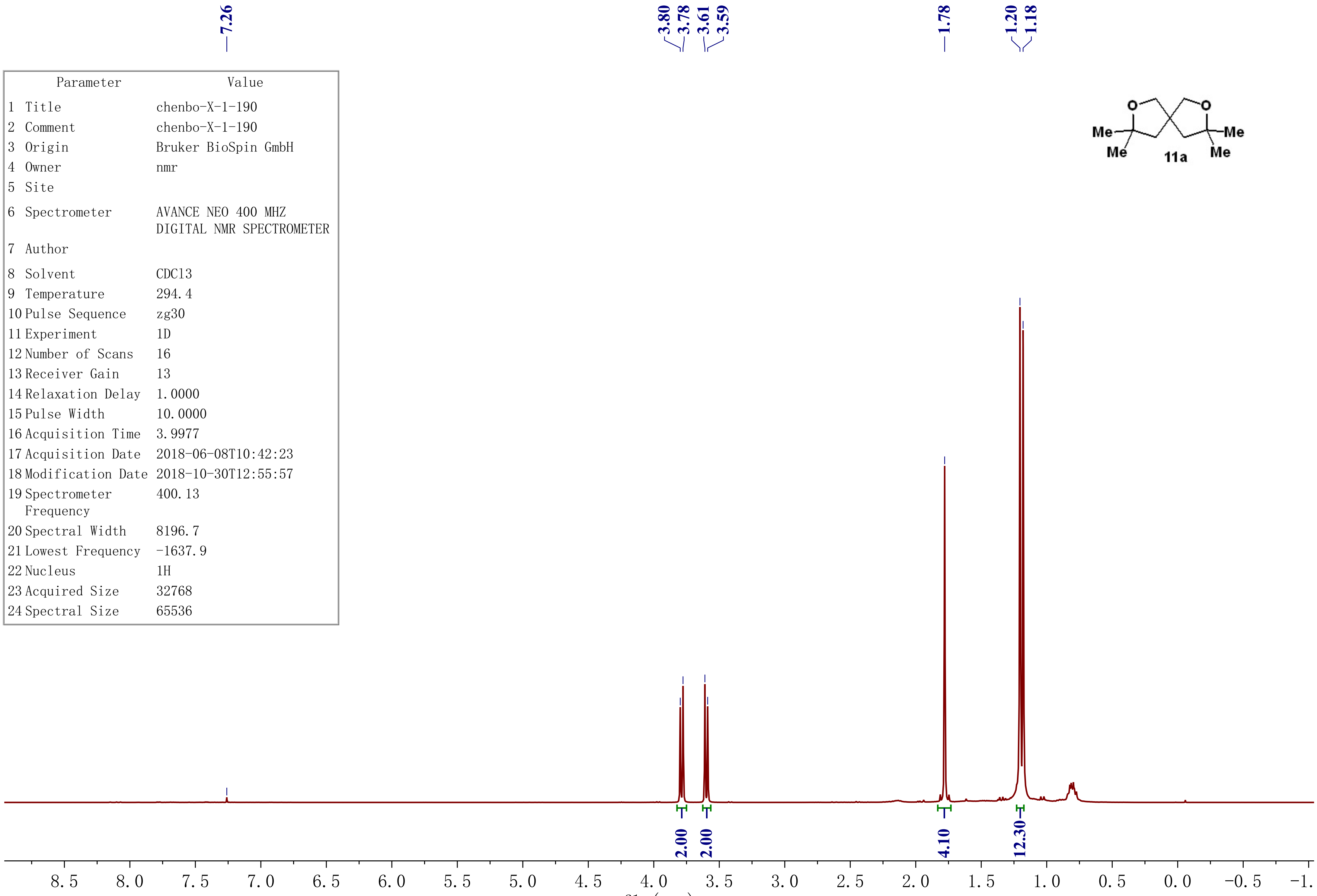

Supplementary Figure 138. ^{1}H NMR spectrum of compound 11a

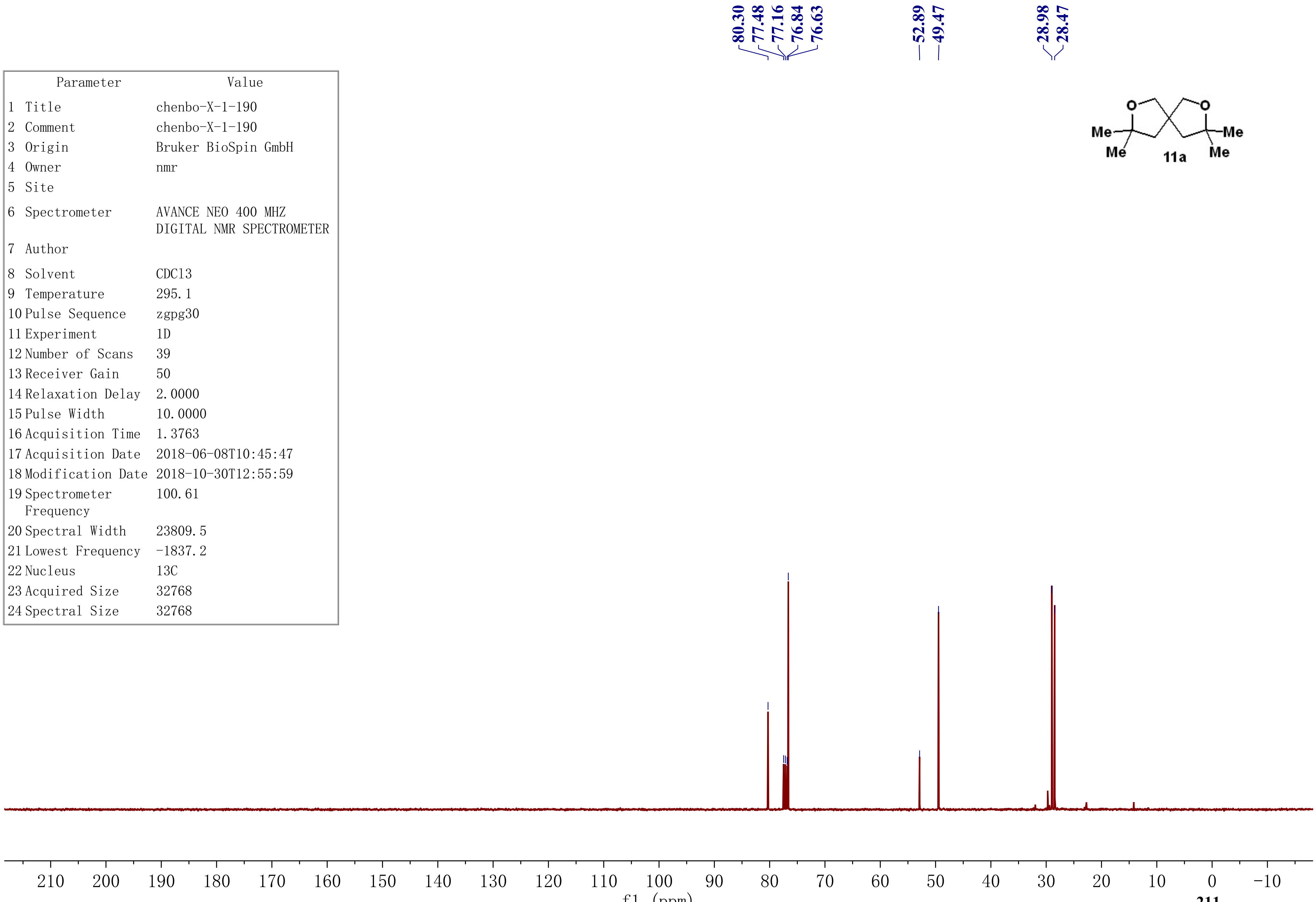

	Parameter	Value
1	Title	chenbo-X-1-190
2	Comment	chenbo-X-1-190
3	Origin	Bruker BioSpin GmbH
4	Owner	nmr
5	Site	
6	Spectrometer	AVANCE NEO 400 MHZ DIGITAL NMR SPECTROMETER
7	Author	
8	Solvent	CDCl3
9	Temperature	295.1
10	Pulse Sequence	zgpg30
11	Experiment	1D
12	Number of Scans	39
13	Receiver Gain	50
14	Relaxation Delay	2.0000
15	Pulse Width	10.0000
16	Acquisition Time	1.3763
17	Acquisition Date	2018-06-08T10:45:47
18	Modification Date	2018-10-30T12:55:59
19	Spectrometer Frequency	100.61
20	Spectral Width	23809.5
21	Lowest Frequency	-1837.2
22	Nucleus	13C
23	Acquired Size	32768
24	Spectral Size	32768

11a

Supplementary Figure 139. ^{13}C NMR spectrum of compound 11a

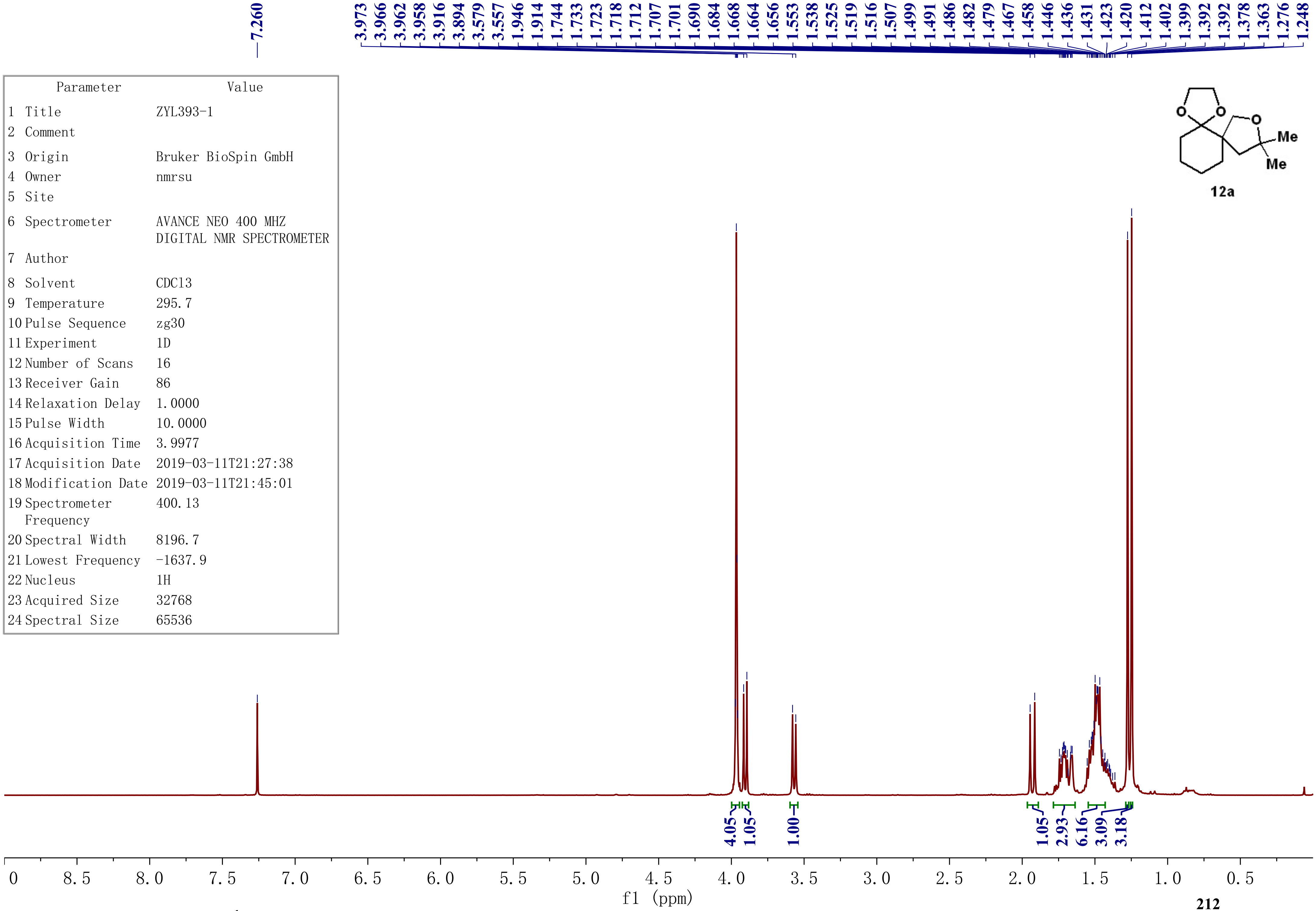

	Parameter	Value
1	Title	ZYL393-1
2	Comment	
3	Origin	Bruker BioSpin GmbH
4	Owner	nmrsu
5	Site	
6	Spectrometer	AVANCE NEO 400 MHZ DIGITAL NMR SPECTROMETER
7	Author	
8	Solvent	CDCl3
9	Temperature	295.7
10	Pulse Sequence	zg30
11	Experiment	1D
12	Number of Scans	16
13	Receiver Gain	86
14	Relaxation Delay	1.0000
15	Pulse Width	10.0000
16	Acquisition Time	3.9977
17	Acquisition Date	2019-03-11T21:27:38
18	Modification Date	2019-03-11T21:45:01
19	Spectrometer Frequency	400.13
20	Spectral Width	8196.7
21	Lowest Frequency	-1637.9
22	Nucleus	1H
23	Acquired Size	32768
24	Spectral Size	65536

Supplementary Figure 140. ^{1}H NMR spectrum of compound 12a

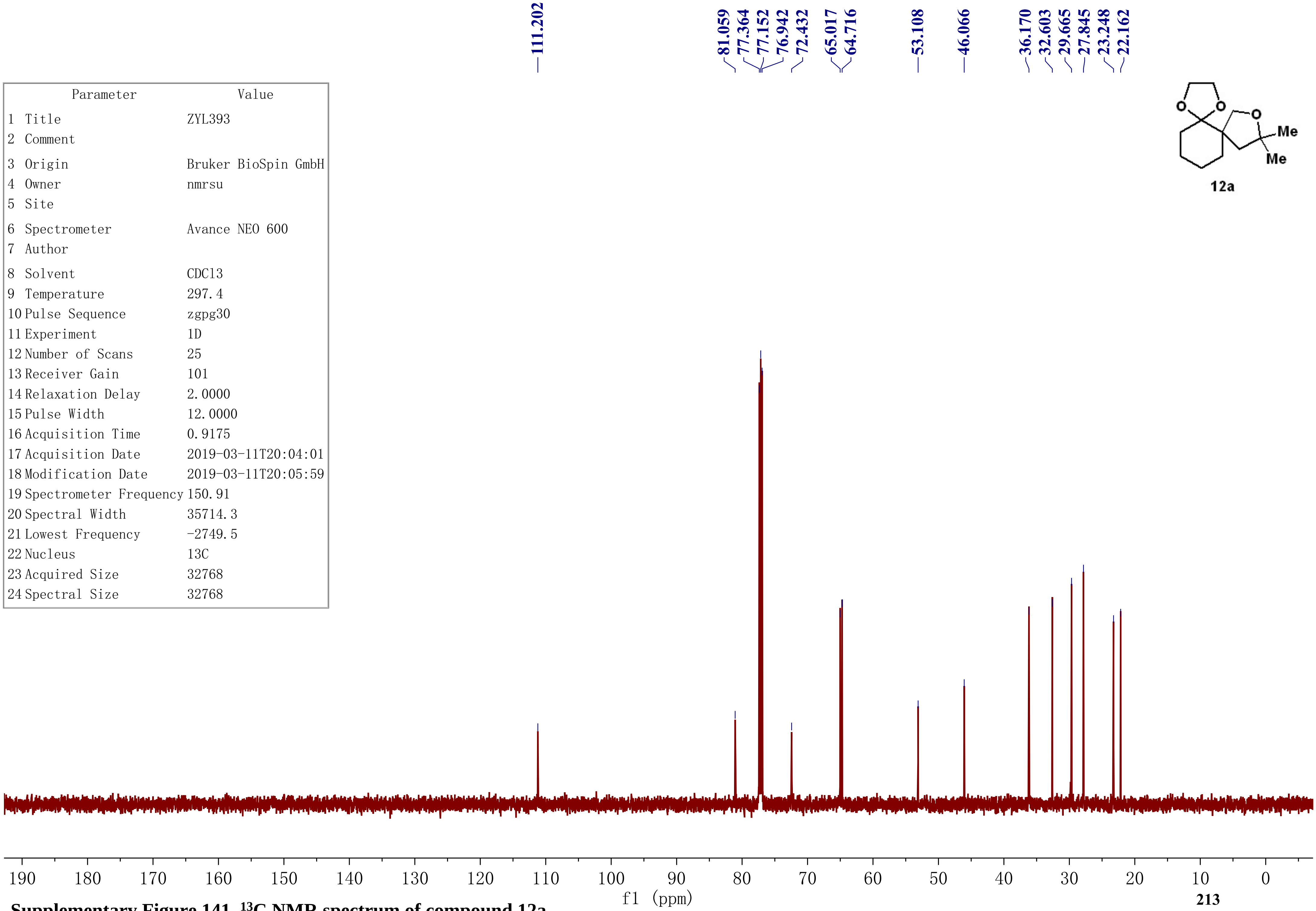

	Parameter	Value
1	Title	ZYL393
2	Comment	
3	Origin	Bruker BioSpin GmbH
4	Owner	nmrsu
5	Site	
6	Spectrometer	Avance NEO 600
7	Author	
8	Solvent	CDCl3
9	Temperature	297.4
10	Pulse Sequence	zgpg30
11	Experiment	1D
12	Number of Scans	25
13	Receiver Gain	101
14	Relaxation Delay	2.0000
15	Pulse Width	12.0000
16	Acquisition Time	0.9175
17	Acquisition Date	2019-03-11T20:04:01
18	Modification Date	2019-03-11T20:05:59
19	Spectrometer Frequency	150.91
20	Spectral Width	35714.3
21	Lowest Frequency	-2749.5
22	Nucleus	13C
23	Acquired Size	32768
24	Spectral Size	32768

12a

Supplementary Figure 141. ^{13}C NMR spectrum of compound 12a

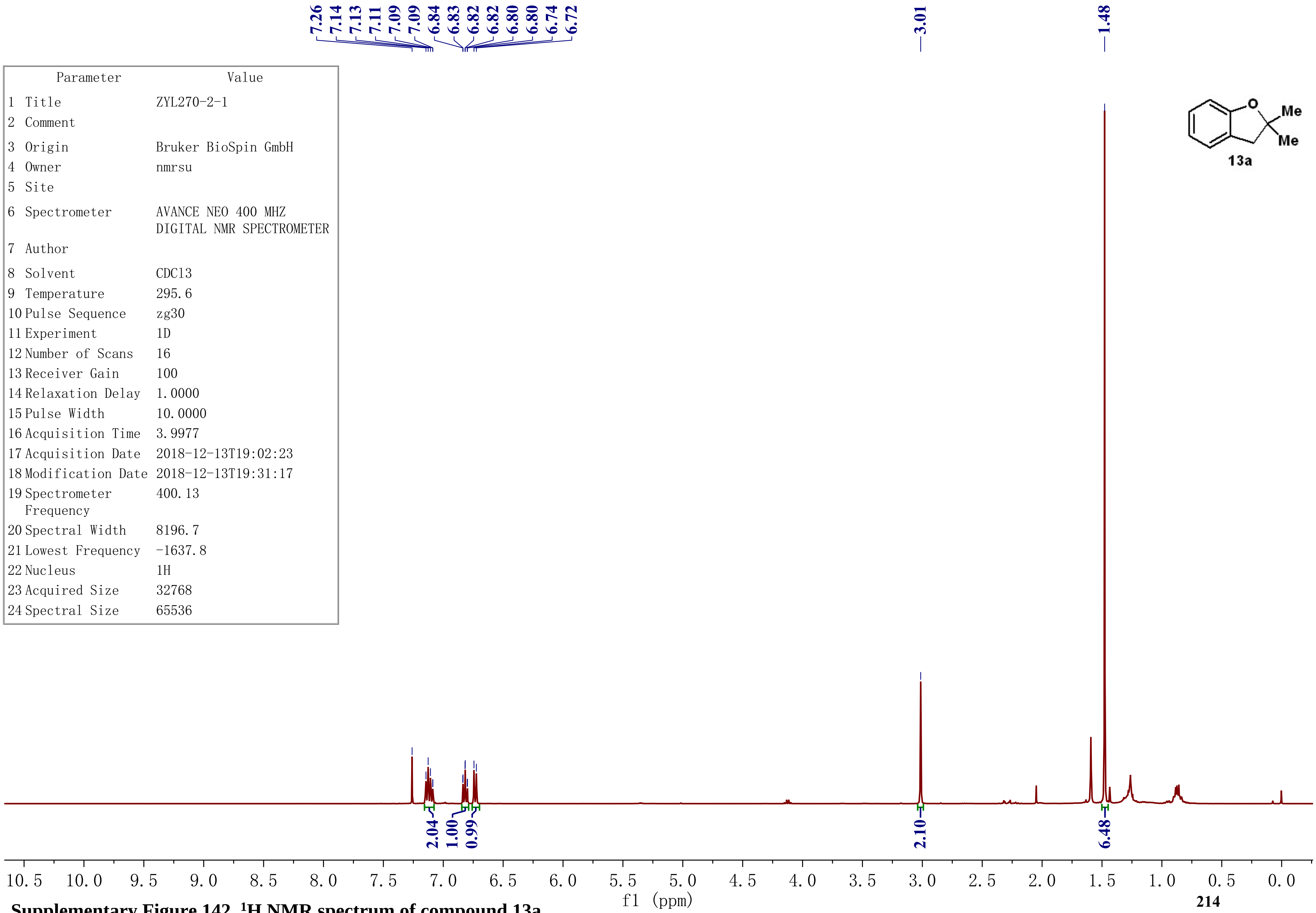

	Parameter	Value
1	Title	ZYL270-2-1
2	Comment	
3	Origin	Bruker BioSpin GmbH
4	Owner	nmrsu
5	Site	
6	Spectrometer	AVANCE NEO 400 MHZ DIGITAL NMR SPECTROMETER
7	Author	
8	Solvent	CDCl3
9	Temperature	295.6
10	Pulse Sequence	zg30
11	Experiment	1D
12	Number of Scans	16
13	Receiver Gain	100
14	Relaxation Delay	1.0000
15	Pulse Width	10.0000
16	Acquisition Time	3.9977
17	Acquisition Date	2018-12-13T19:02:23
18	Modification Date	2018-12-13T19:31:17
19	Spectrometer Frequency	400.13
20	Spectral Width	8196.7
21	Lowest Frequency	-1637.8
22	Nucleus	1H
23	Acquired Size	32768
24	Spectral Size	65536

13a

Supplementary Figure 142. ^{1}H NMR spectrum of compound 13a

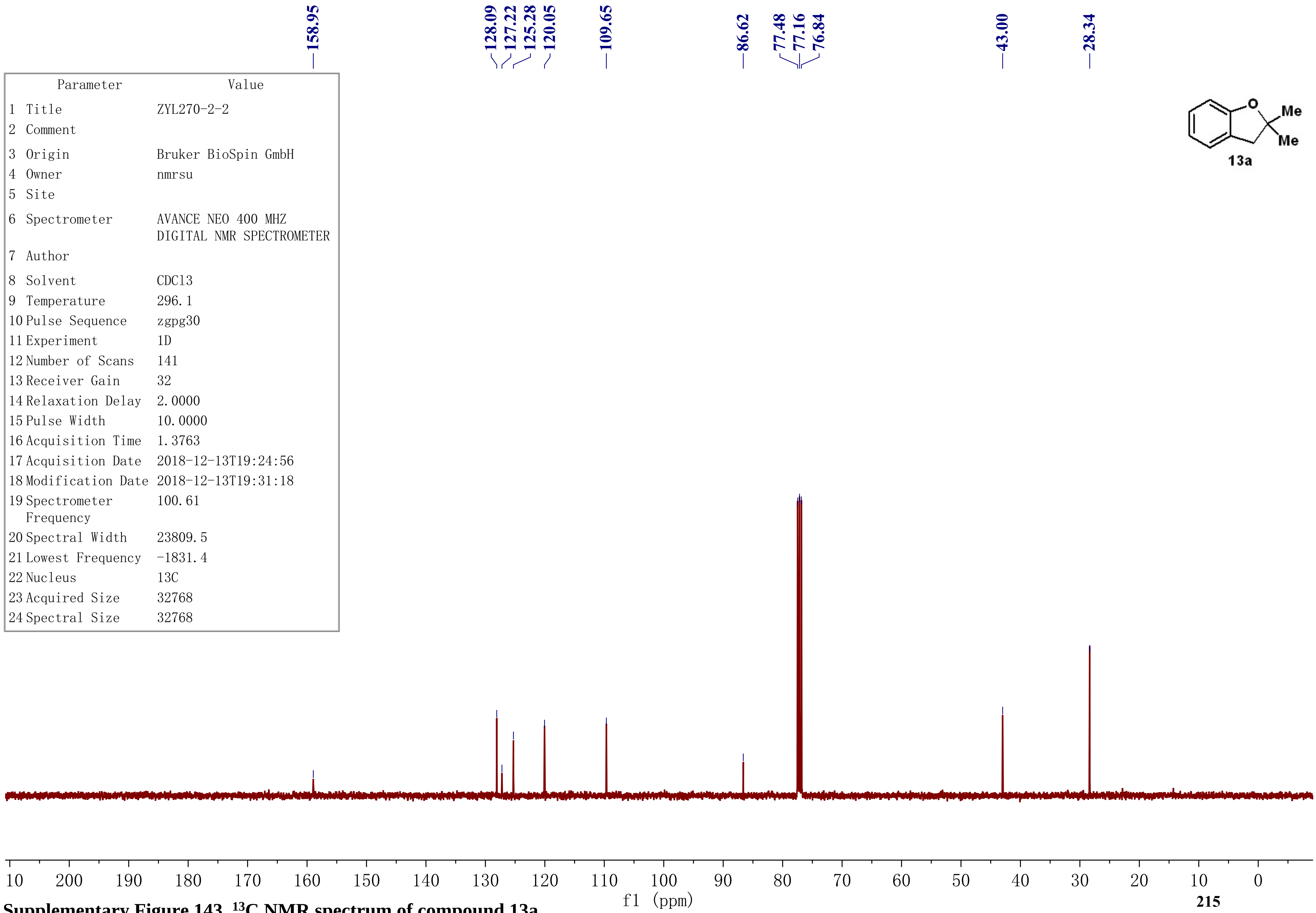

	Parameter	Value
1	Title	ZYL270-2-2
2	Comment	
3	Origin	Bruker BioSpin GmbH
4	Owner	nmrsu
5	Site	
6	Spectrometer	AVANCE NEO 400 MHZ DIGITAL NMR SPECTROMETER
7	Author	
8	Solvent	CDCl3
9	Temperature	296.1
10	Pulse Sequence	zgpg30
11	Experiment	1D
12	Number of Scans	141
13	Receiver Gain	32
14	Relaxation Delay	2.0000
15	Pulse Width	10.0000
16	Acquisition Time	1.3763
17	Acquisition Date	2018-12-13T19:24:56
18	Modification Date	2018-12-13T19:31:18
19	Spectrometer Frequency	100.61
20	Spectral Width	23809.5
21	Lowest Frequency	-1831.4
22	Nucleus	13C
23	Acquired Size	32768
24	Spectral Size	32768

Supplementary Figure 143. ^{13}C NMR spectrum of compound 13a

Parameter		Value
1	Title	ZYL303
2	Comment	
3	Origin	Bruker BioSpin GmbH
4	Owner	nmrsu
5	Site	
6	Spectrometer	AVANCE NEO 400 MHZ DIGITAL NMR SPECTROMETER
7	Author	
8	Solvent	CDCl3
9	Temperature	295.9
10	Pulse Sequence	zg30
11	Experiment	1D
12	Number of Scans	16
13	Receiver Gain	32
14	Relaxation Delay	1.0000
15	Pulse Width	10.0000
16	Acquisition Time	3.9977
17	Acquisition Date	2019-01-10T20:25:44
18	Modification Date	2019-01-10T21:24:50
19	Spectrometer Frequency	400.13
20	Spectral Width	8196.7
21	Lowest Frequency	-1648.3
22	Nucleus	1H
23	Acquired Size	32768
24	Spectral Size	65536

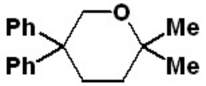

14a

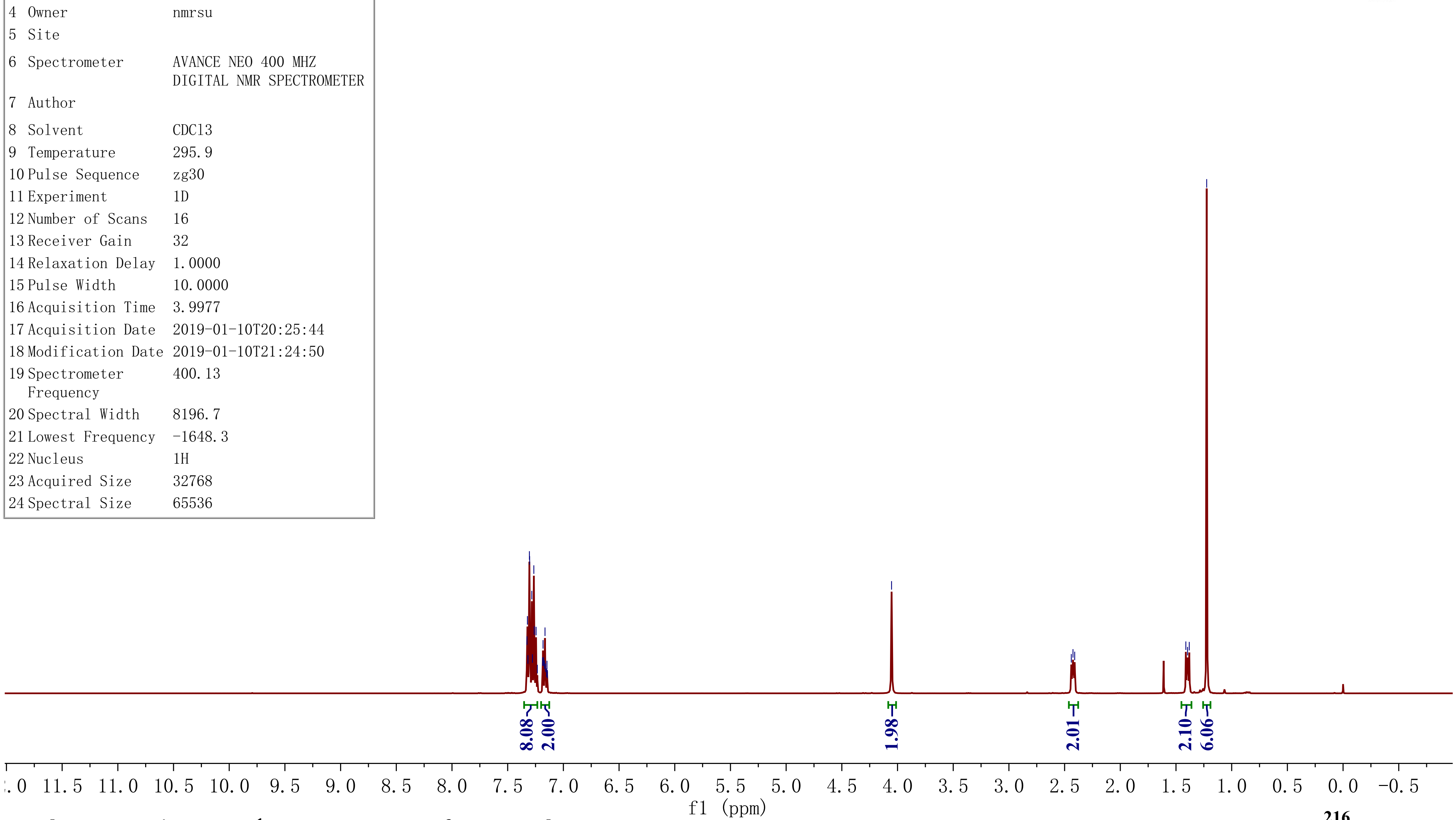

Supplementary Figure 144. ^{1}H NMR spectrum of compound 14a

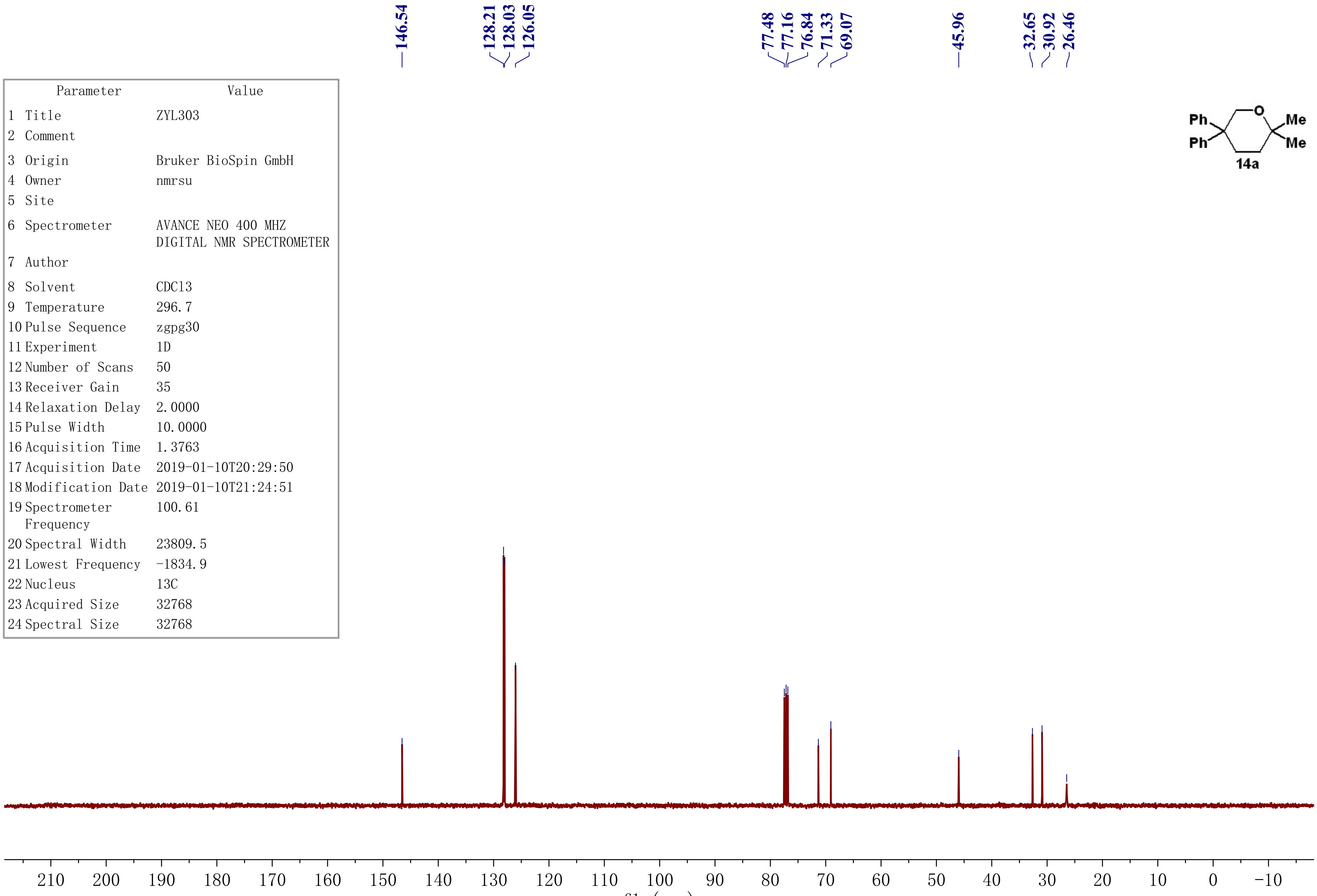

	Parameter	Value
1	Title	ZYL303
2	Comment	
3	Origin	Bruker BioSpin GmbH
4	Owner	nmrsu
5	Site	
6	Spectrometer	AVANCE NEO 400 MHZ DIGITAL NMR SPECTROMETER
7	Author	
8	Solvent	CDCl3
9	Temperature	296.7
10	Pulse Sequence	zgpg30
11	Experiment	1D
12	Number of Scans	50
13	Receiver Gain	35
14	Relaxation Delay	2.0000
15	Pulse Width	10.0000
16	Acquisition Time	1.3763
17	Acquisition Date	2019-01-10T20:29:50
18	Modification Date	2019-01-10T21:24:51
19	Spectrometer Frequency	100.61
20	Spectral Width	23809.5
21	Lowest Frequency	-1834.9
22	Nucleus	13C
23	Acquired Size	32768
24	Spectral Size	32768

14a

Supplementary Figure 145. ^{13}**C NMR spectrum of compound 14a**

	Parameter	Value
1	Title	chenbo-X-1-183-2-L
2	Comment	chenbo-X-1-183-2-L
3	Origin	Bruker BioSpin GmbH
4	Owner	nmr
5	Site	
6	Spectrometer	AVANCE NEO 400 MHZ DIGITAL NMR SPECTROMETER
7	Author	
8	Solvent	CDCl3
9	Temperature	293.2
10	Pulse Sequence	zg30
11	Experiment	1D
12	Number of Scans	16
13	Receiver Gain	15
14	Relaxation Delay	1.0000
15	Pulse Width	10.0000
16	Acquisition Time	3.9977
17	Acquisition Date	2018-06-02T14:00:58
18	Modification Date	2018-10-30T12:55:54
19	Spectrometer Frequency	400.13
20	Spectral Width	8196.7
21	Lowest Frequency	-1637.9
22	Nucleus	1H
23	Acquired Size	32768
24	Spectral Size	65536

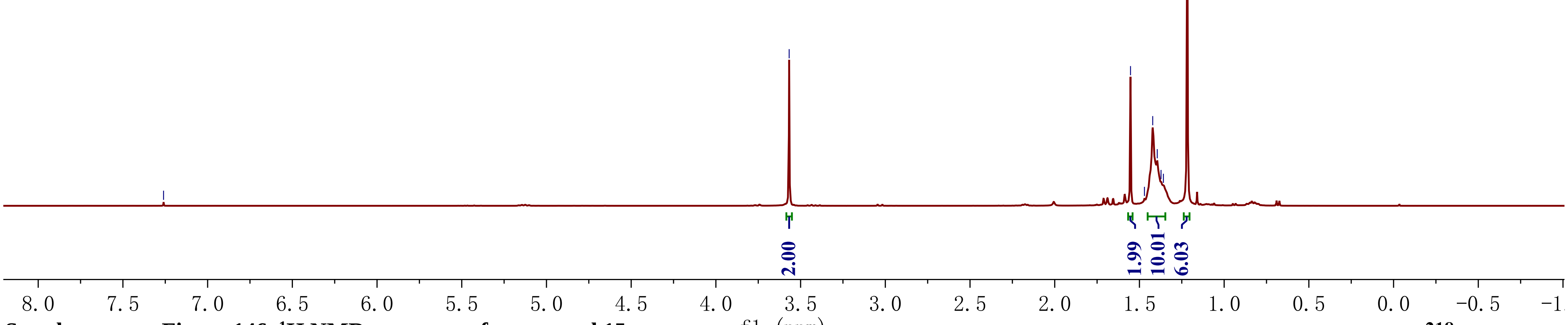

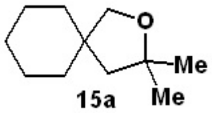

Supplementary Figure 146. ^{1}H NMR spectrum of compound 15a

chenbo-X-1-183-2-L
chenbo-X-1-183-2-L

	Parameter	Value
1	Title	chenbo-X-1-183-2-L
2	Comment	chenbo-X-1-183-2-L
3	Origin	Bruker BioSpin GmbH
4	Owner	nmr
5	Site	
6	Spectrometer	AVANCE NEO 400 MHZ DIGITAL NMR SPECTROMETER
7	Author	
8	Solvent	CDCl3
9	Temperature	293.6
10	Pulse Sequence	zgpg30
11	Experiment	1D
12	Number of Scans	59
13	Receiver Gain	46
14	Relaxation Delay	2.0000
15	Pulse Width	10.0000
16	Acquisition Time	1.3763
17	Acquisition Date	2018-06-02T14:05:33
18	Modification Date	2018-10-30T12:55:55
19	Spectrometer Frequency	100.61
20	Spectral Width	23809.5
21	Lowest Frequency	-1835.2
22	Nucleus	13C
23	Acquired Size	32768
24	Spectral Size	32768

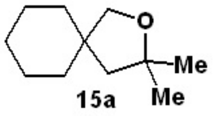

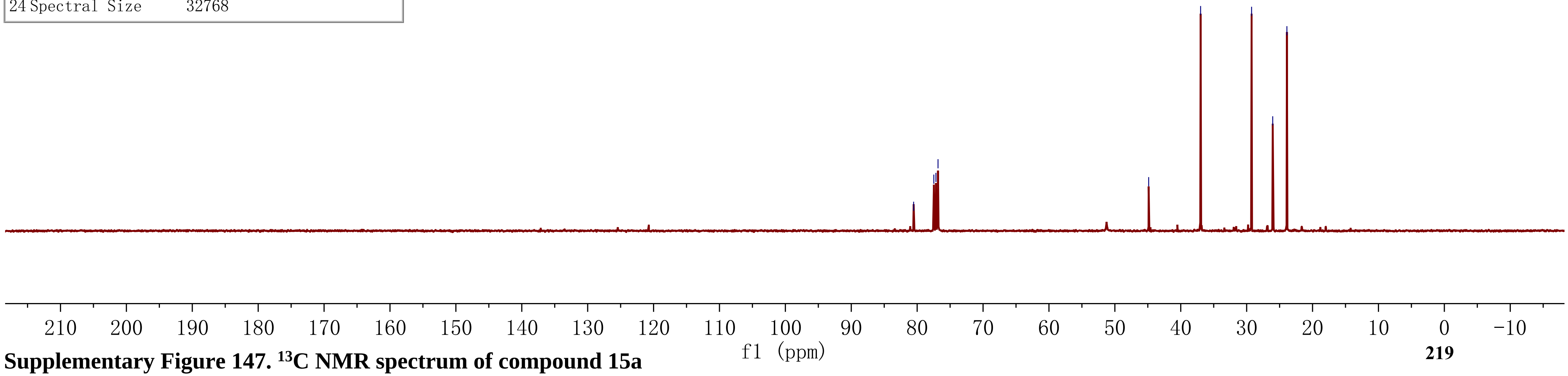

Supplementary Figure 147. ^{13}C NMR spectrum of compound 15a

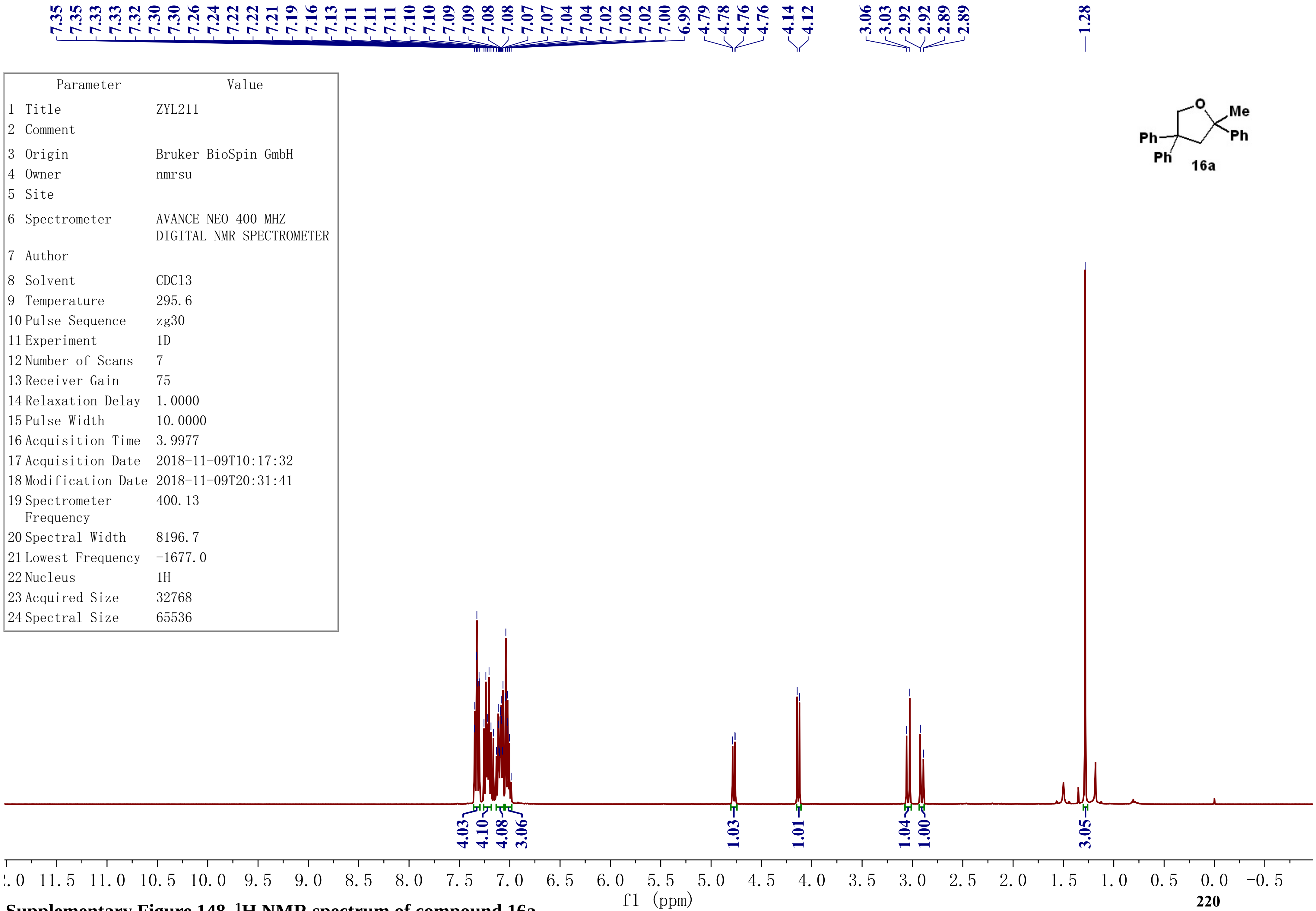

	Parameter	Value
1	Title	ZYL211
2	Comment	
3	Origin	Bruker BioSpin GmbH
4	Owner	nmrsu
5	Site	
6	Spectrometer	AVANCE NEO 400 MHZ DIGITAL NMR SPECTROMETER
7	Author	
8	Solvent	CDCl3
9	Temperature	295.6
10	Pulse Sequence	zg30
11	Experiment	1D
12	Number of Scans	7
13	Receiver Gain	75
14	Relaxation Delay	1.0000
15	Pulse Width	10.0000
16	Acquisition Time	3.9977
17	Acquisition Date	2018-11-09T10:17:32
18	Modification Date	2018-11-09T20:31:41
19	Spectrometer Frequency	400.13
20	Spectral Width	8196.7
21	Lowest Frequency	-1677.0
22	Nucleus	1H
23	Acquired Size	32768
24	Spectral Size	65536

Supplementary Figure 148. ^{1}H NMR spectrum of compound 16a

Parameter	Value
1 Title	ZYL211
2 Comment	
3 Origin	Bruker BioSpin GmbH
4 Owner	nmrsu
5 Site	
6 Spectrometer	AVANCE NEO 400 MHZ DIGITAL NMR SPECTROMETER
7 Author	
8 Solvent	CDCl3
9 Temperature	295.9
10 Pulse Sequence	zgpg30
11 Experiment	1D
12 Number of Scans	21
13 Receiver Gain	35
14 Relaxation Delay	2.0000
15 Pulse Width	10.0000
16 Acquisition Time	1.3763
17 Acquisition Date	2018-11-09T10:19:41
18 Modification Date	2018-11-09T20:31:41
19 Spectrometer Frequency	100.61
20 Spectral Width	23809.5
21 Lowest Frequency	-1834.0
22 Nucleus	13C
23 Acquired Size	32768
24 Spectral Size	32768

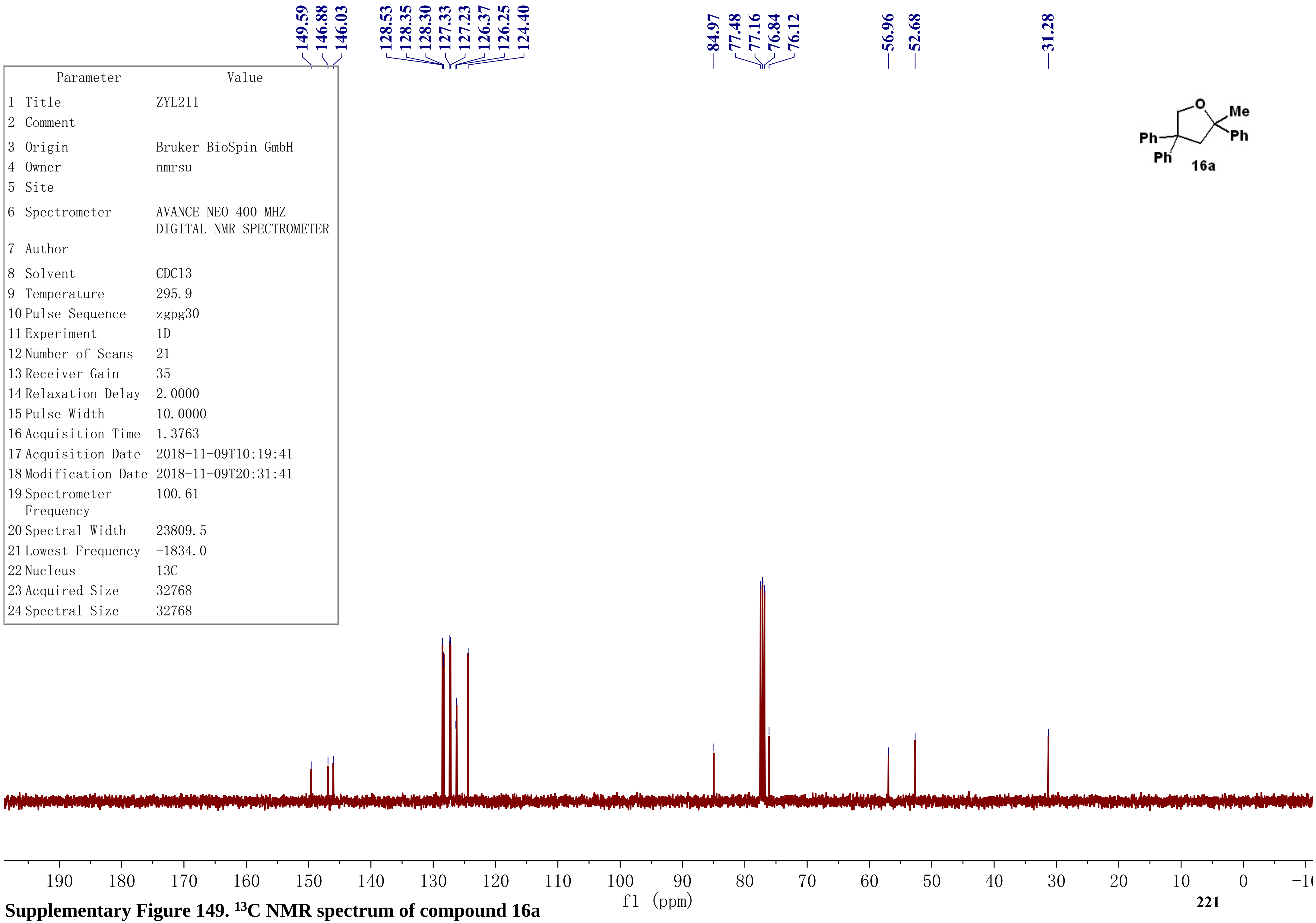

16a

Supplementary Figure 149. ^{13}C **NMR spectrum of compound 16a**

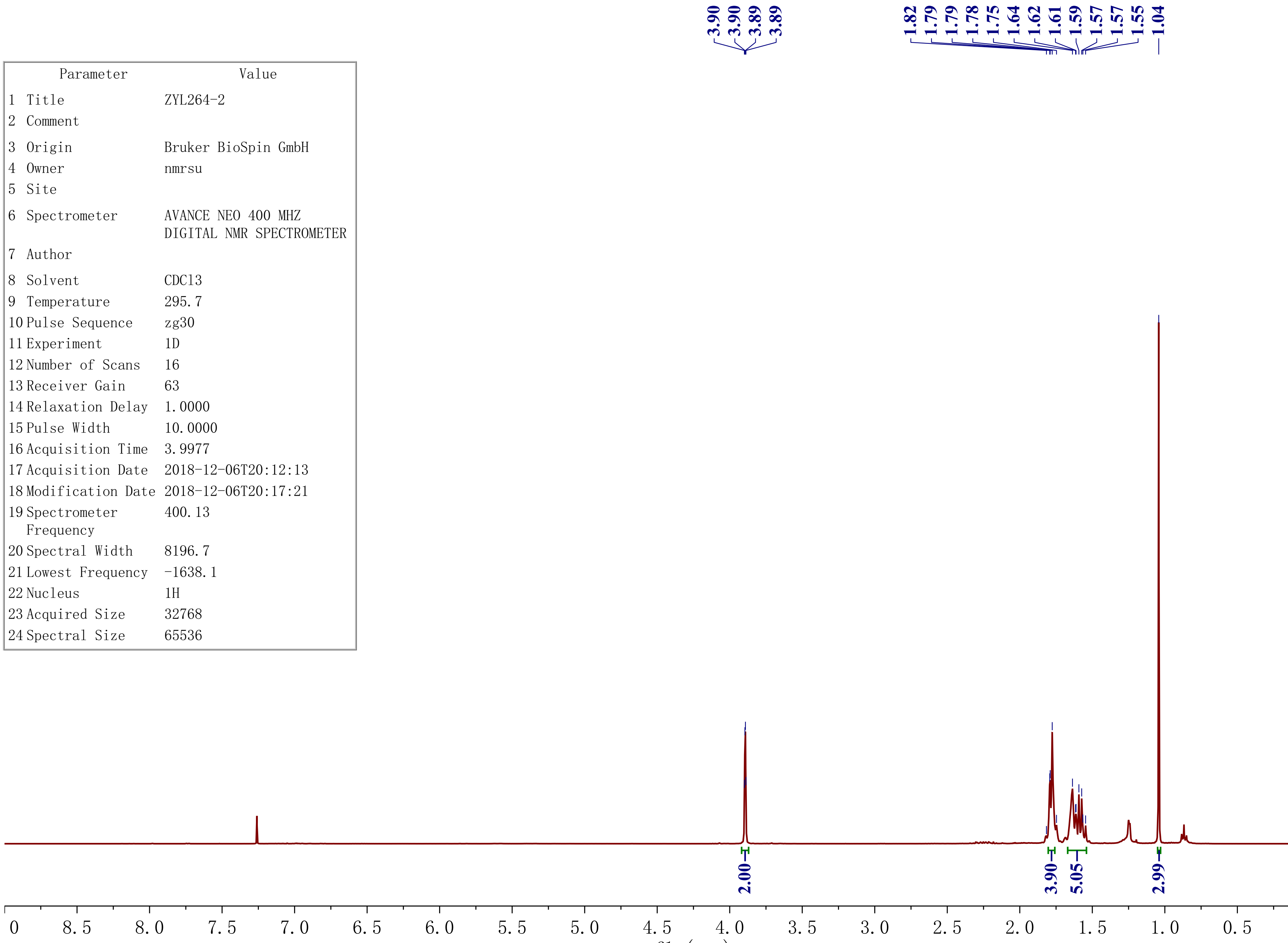

Parameter	Value
1 Title	ZYL264-2
2 Comment	
3 Origin	Bruker BioSpin GmbH
4 Owner	nmrsu
5 Site	
6 Spectrometer	AVANCE NEO 400 MHZ DIGITAL NMR SPECTROMETER
7 Author	
8 Solvent	CDCl3
9 Temperature	295.7
10 Pulse Sequence	zg30
11 Experiment	1D
12 Number of Scans	16
13 Receiver Gain	63
14 Relaxation Delay	1.0000
15 Pulse Width	10.0000
16 Acquisition Time	3.9977
17 Acquisition Date	2018-12-06T20:12:13
18 Modification Date	2018-12-06T20:17:21
19 Spectrometer Frequency	400.13
20 Spectral Width	8196.7
21 Lowest Frequency	-1638.1
22 Nucleus	1H
23 Acquired Size	32768
24 Spectral Size	65536

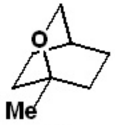

17a

Supplementary Figure 150. ^{1}H NMR spectrum of compound 17a

Parameter	Value
1 Title	ZYL264-2
2 Comment	
3 Origin	Bruker BioSpin GmbH
4 Owner	nmrsu
5 Site	
6 Spectrometer	AVANCE NEO 400 MHZ DIGITAL NMR SPECTROMETER
7 Author	
8 Solvent	CDCl3
9 Temperature	296.2
10 Pulse Sequence	zgpg30
11 Experiment	1D
12 Number of Scans	21
13 Receiver Gain	32
14 Relaxation Delay	2.0000
15 Pulse Width	10.0000
16 Acquisition Time	1.3763
17 Acquisition Date	2018-12-06T20:14:24
18 Modification Date	2018-12-06T20:17:22
19 Spectrometer Frequency	100.61
20 Spectral Width	23809.5
21 Lowest Frequency	-1831.7
22 Nucleus	13C
23 Acquired Size	32768
24 Spectral Size	32768

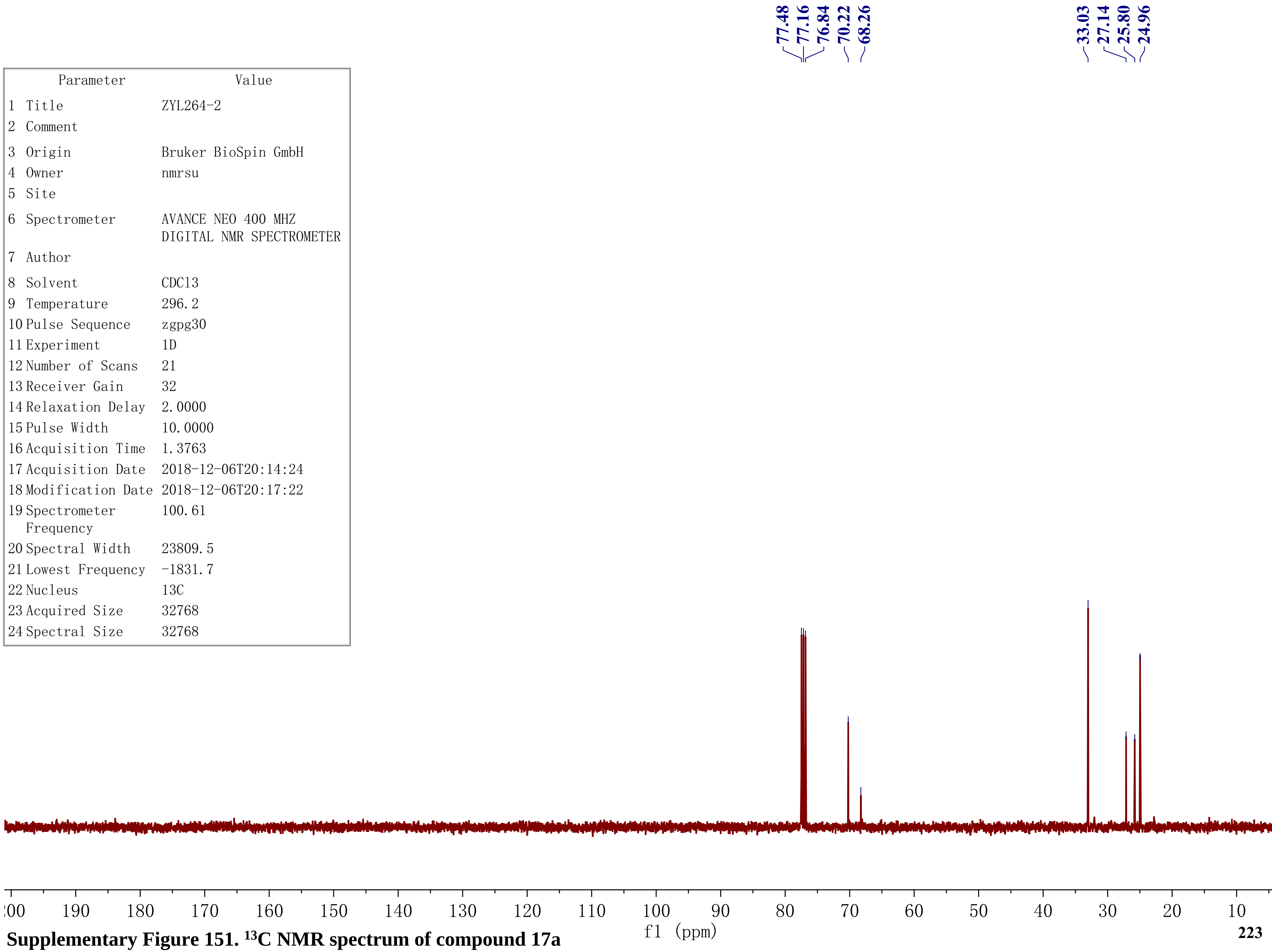

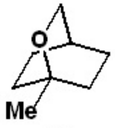

17a

Supplementary Figure 151. ^{13}C NMR spectrum of compound 17a

Parameter	Value
1 Title	chenbo-X-1-197-2-up
2 Comment	chenbo-X-1-197-2-up
3 Origin	Bruker BioSpin GmbH
4 Owner	nmr
5 Site	
6 Spectrometer	AVANCE NEO 400 MHZ DIGITAL NMR SPECTROMETER
7 Author	
8 Solvent	CDCl3
9 Temperature	295.5
10 Pulse Sequence	zg30
11 Experiment	1D
12 Number of Scans	16
13 Receiver Gain	52
14 Relaxation Delay	1.0000
15 Pulse Width	10.0000
16 Acquisition Time	3.9977
17 Acquisition Date	2018-06-13T13:13:37
18 Modification Date	2018-10-30T12:55:44
19 Spectrometer Frequency	400.13
20 Spectral Width	8196.7
21 Lowest Frequency	-1637.9
22 Nucleus	1H
23 Acquired Size	32768
24 Spectral Size	65536

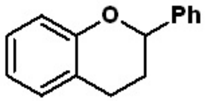

18a

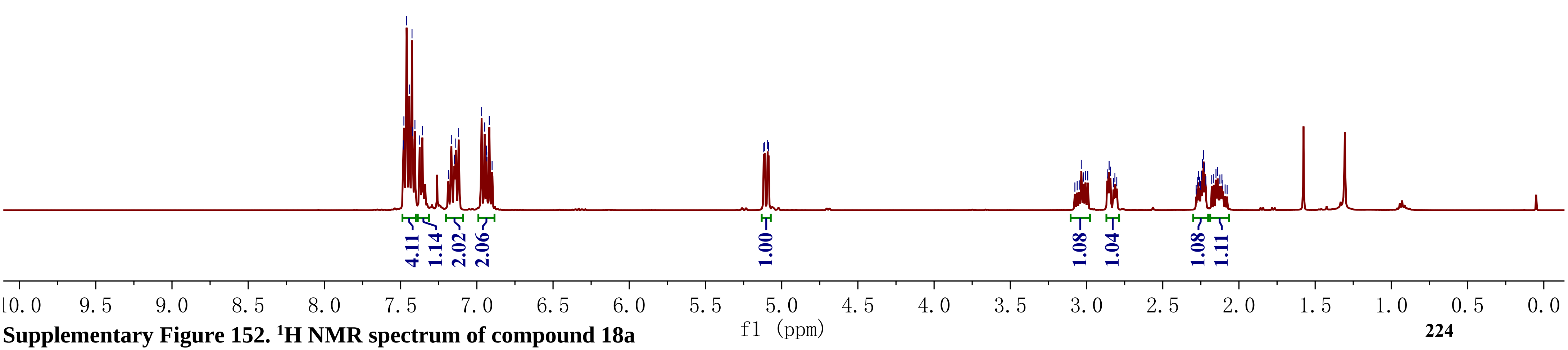

Supplementary Figure 152. ^1H NMR spectrum of compound 18a

Parameter	Value
1 Title	chenbo-X-1-197-2-up
2 Comment	chenbo-X-1-197-2-up
3 Origin	Bruker BioSpin GmbH
4 Owner	nmr
5 Site	
6 Spectrometer	AVANCE NEO 400 MHZ DIGITAL NMR SPECTROMETER
7 Author	
8 Solvent	CDCl3
9 Temperature	296.3
10 Pulse Sequence	zgpg30
11 Experiment	1D
12 Number of Scans	43
13 Receiver Gain	46
14 Relaxation Delay	2.0000
15 Pulse Width	10.0000
16 Acquisition Time	1.3763
17 Acquisition Date	2018-06-13T13:17:17
18 Modification Date	2018-10-30T12:55:46
19 Spectrometer Frequency	100.61
20 Spectral Width	23809.5
21 Lowest Frequency	-1837.6
22 Nucleus	13C
23 Acquired Size	32768
24 Spectral Size	32768

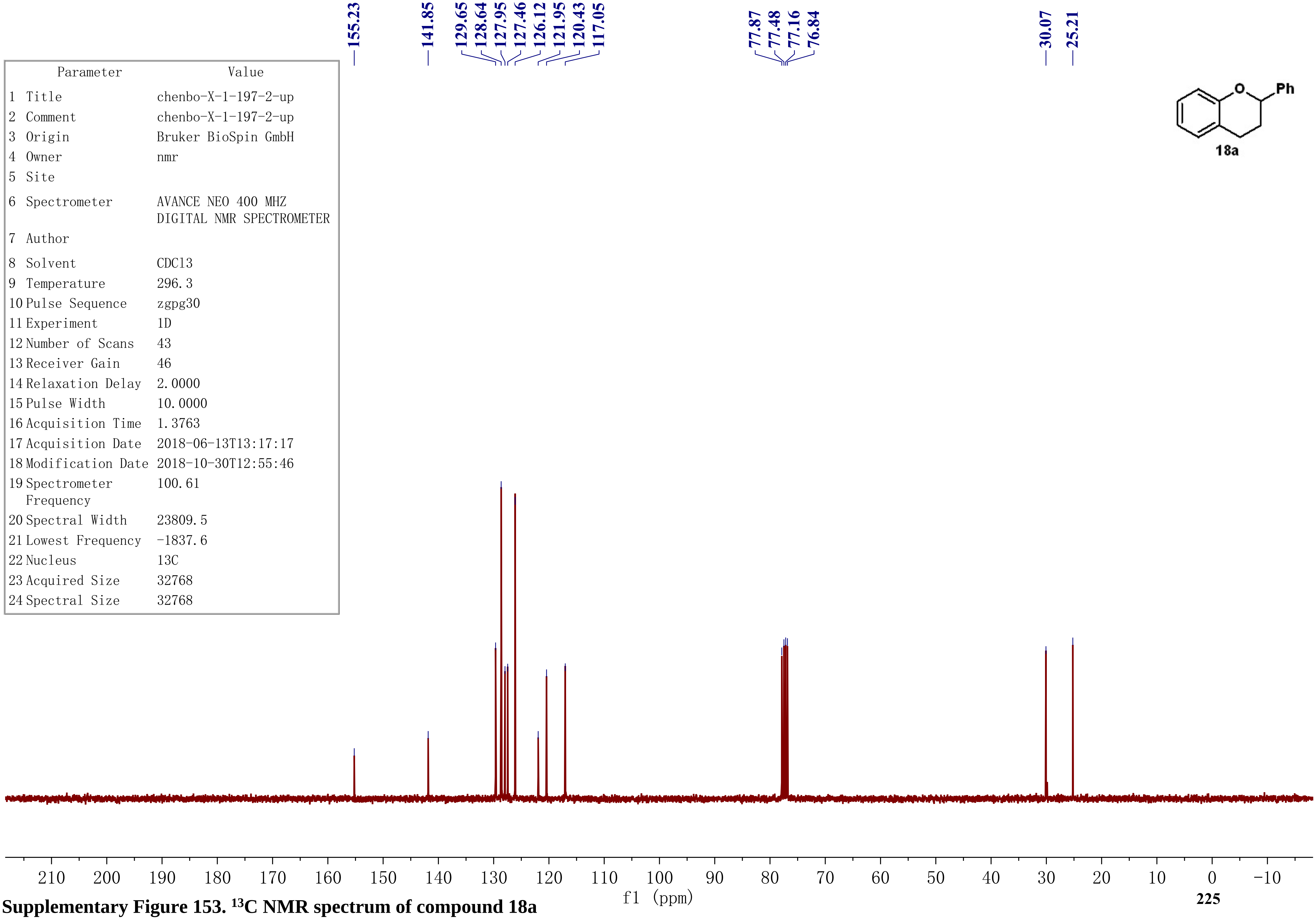

18a

Supplementary Figure 153. ^{13}C NMR spectrum of compound 18a

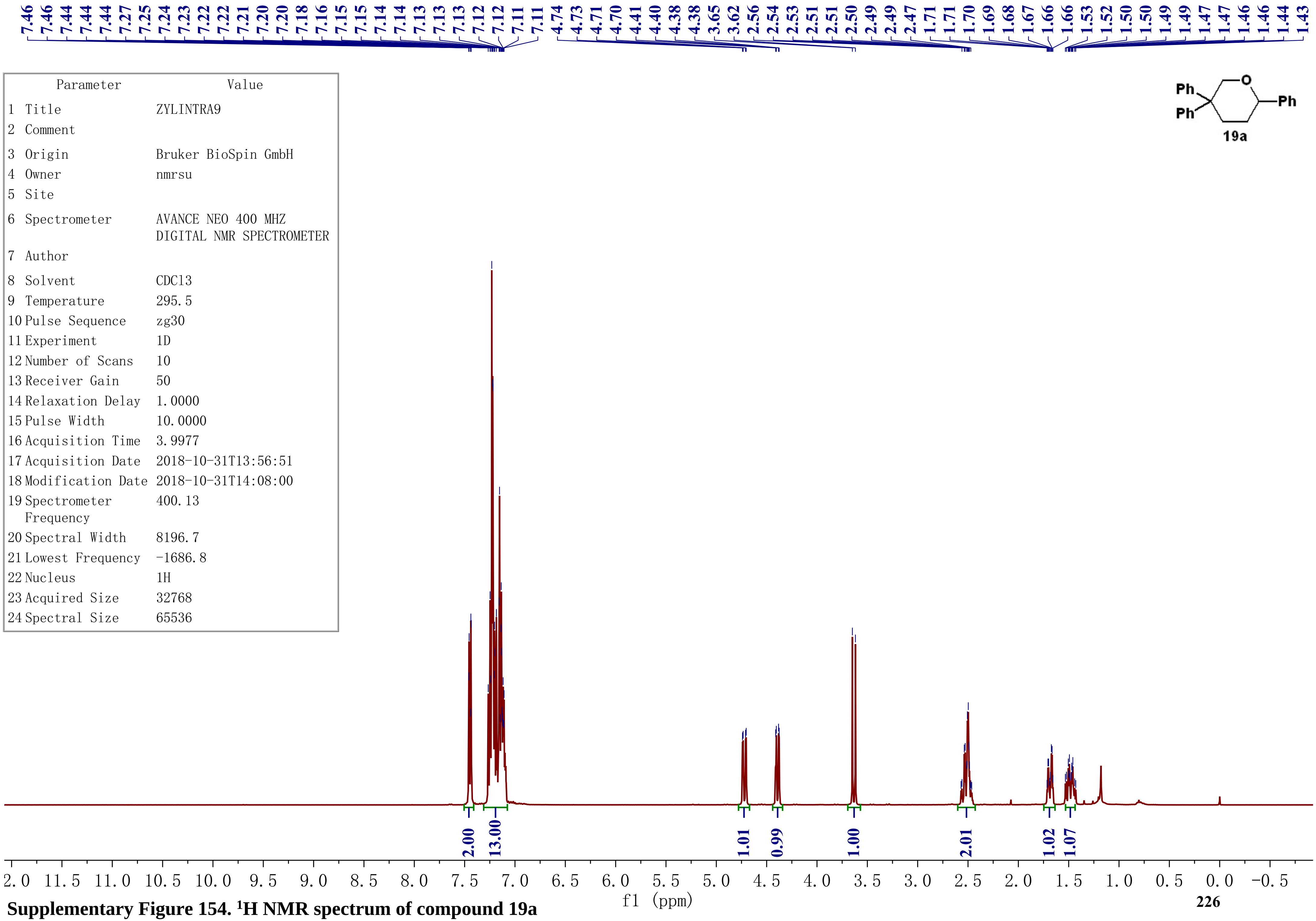

	Parameter	Value
1	Title	ZYLINTRA9
2	Comment	
3	Origin	Bruker BioSpin GmbH
4	Owner	nmrsu
5	Site	
6	Spectrometer	AVANCE NEO 400 MHZ DIGITAL NMR SPECTROMETER
7	Author	
8	Solvent	CDCl3
9	Temperature	295.5
10	Pulse Sequence	zg30
11	Experiment	1D
12	Number of Scans	10
13	Receiver Gain	50
14	Relaxation Delay	1.0000
15	Pulse Width	10.0000
16	Acquisition Time	3.9977
17	Acquisition Date	2018-10-31T13:56:51
18	Modification Date	2018-10-31T14:08:00
19	Spectrometer Frequency	400.13
20	Spectral Width	8196.7
21	Lowest Frequency	-1686.8
22	Nucleus	1H
23	Acquired Size	32768
24	Spectral Size	65536

Supplementary Figure 154. ^{1}H NMR spectrum of compound 19a

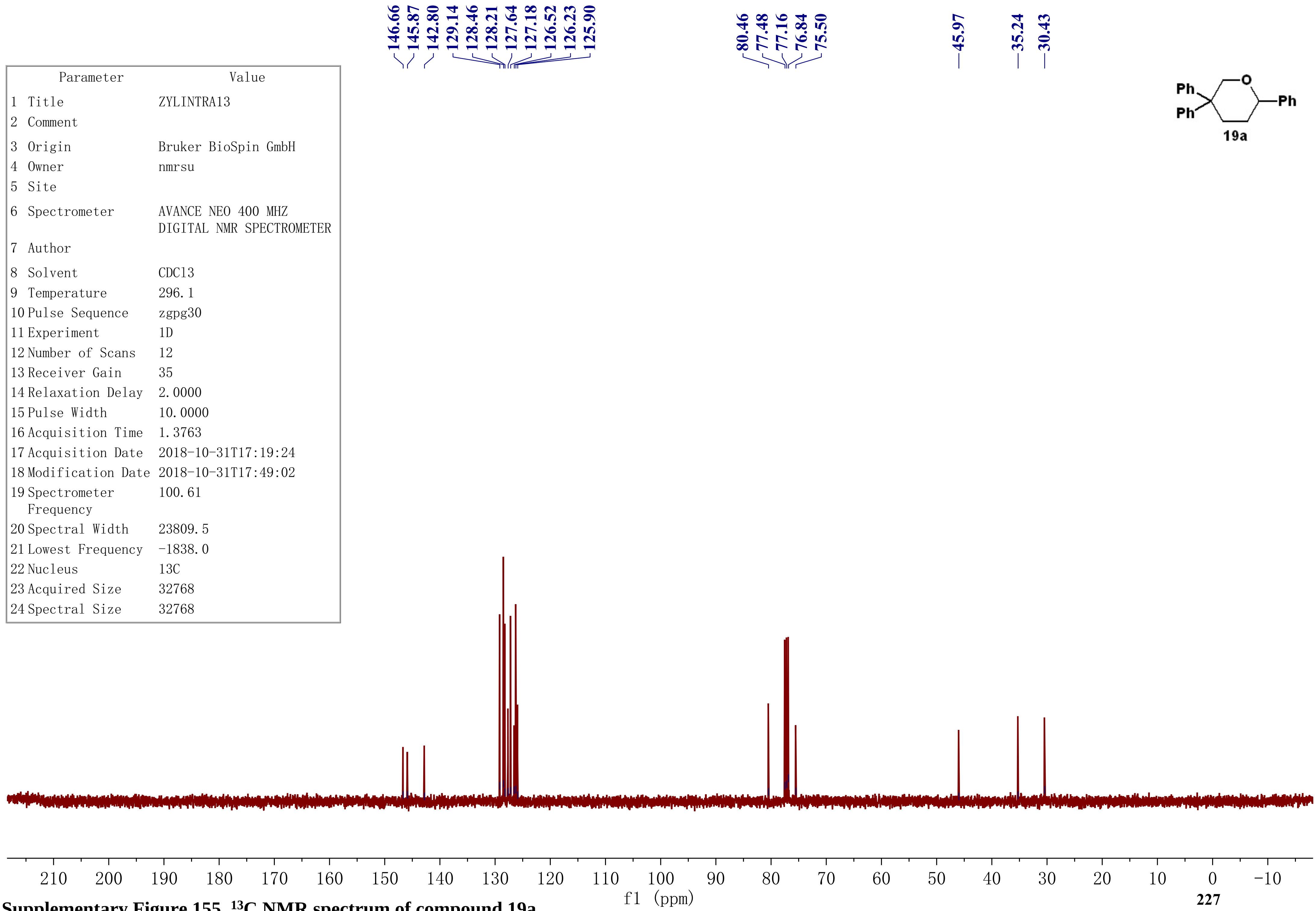

Parameter	Value
1 Title	ZYLINTRA13
2 Comment	
3 Origin	Bruker BioSpin GmbH
4 Owner	nmrsu
5 Site	
6 Spectrometer	AVANCE NEO 400 MHZ DIGITAL NMR SPECTROMETER
7 Author	
8 Solvent	CDCl3
9 Temperature	296.1
10 Pulse Sequence	zgpg30
11 Experiment	1D
12 Number of Scans	12
13 Receiver Gain	35
14 Relaxation Delay	2.0000
15 Pulse Width	10.0000
16 Acquisition Time	1.3763
17 Acquisition Date	2018-10-31T17:19:24
18 Modification Date	2018-10-31T17:49:02
19 Spectrometer Frequency	100.61
20 Spectral Width	23809.5
21 Lowest Frequency	-1838.0
22 Nucleus	13C
23 Acquired Size	32768
24 Spectral Size	32768

19a

Supplementary Figure 155. ^{13}C NMR spectrum of compound 19a

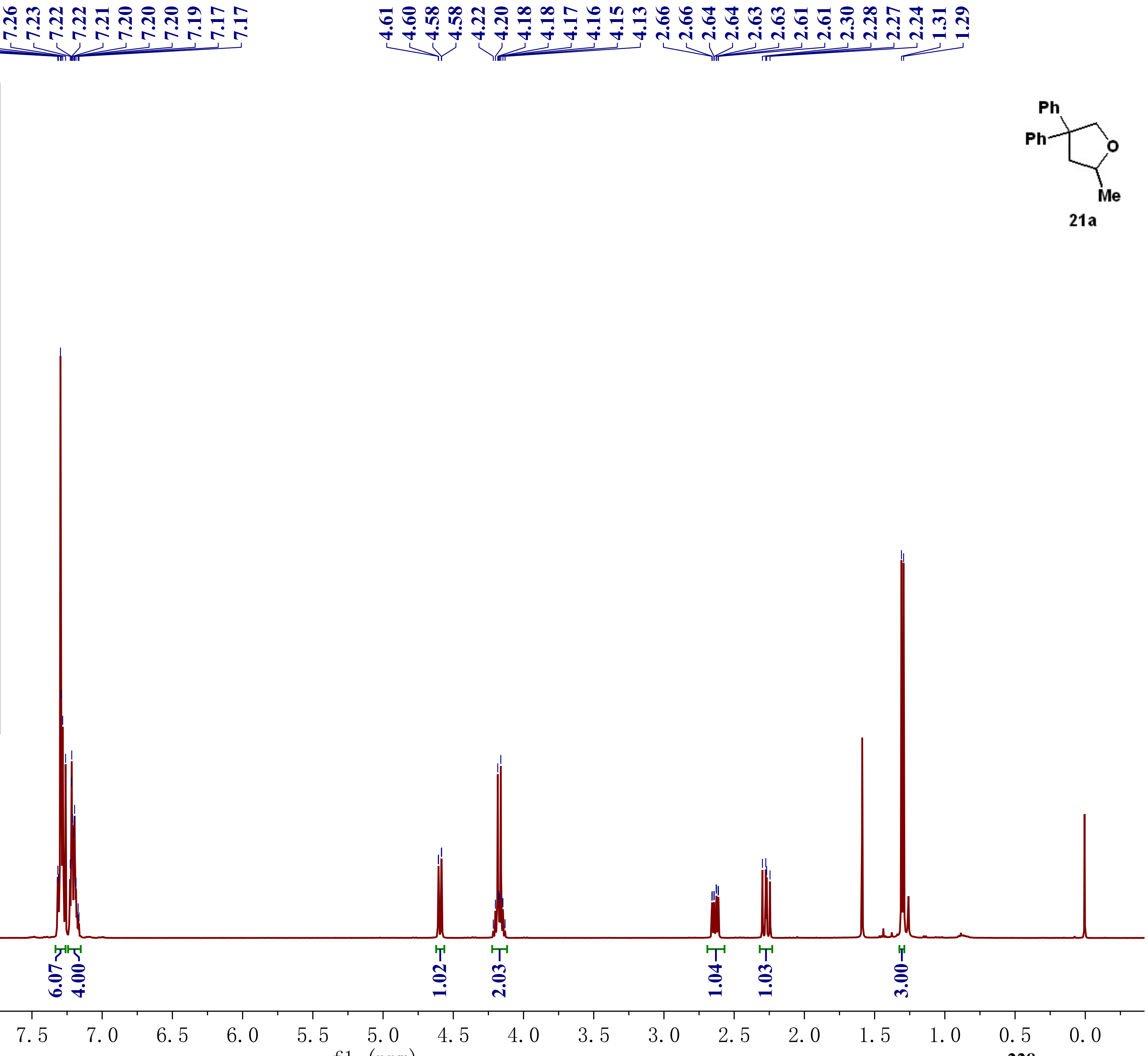

Parameter	Value
1 Title	chenbo-X-1-034-b
2 Comment	chenbo-X-1-034-b
3 Origin	Bruker BioSpin GmbH
4 Owner	nmr
5 Site	
6 Spectrometer	AVANCE NEO 400 MHZ DIGITAL NMR SPECTROMETER
7 Author	
8 Solvent	CDCl3
9 Temperature	293.7
10 Pulse Sequence	zg30
11 Experiment	1D
12 Number of Scans	8
13 Receiver Gain	101
14 Relaxation Delay	1.0000
15 Pulse Width	10.0000
16 Acquisition Time	3.9977
17 Acquisition Date	2018-01-18T11:37:05
18 Modification Date	2018-10-30T12:47:24
19 Spectrometer Frequency	400.13
20 Spectral Width	8196.7
21 Lowest Frequency	-1387.7
22 Nucleus	1H
23 Acquired Size	32768
24 Spectral Size	65536

21a

Supplementary Figure 156. ^{1}H NMR spectrum of compound 21a

Parameter	Value
1 Title	chenbo-X-1-034-b
2 Comment	chenbo-X-1-034-b
3 Origin	Bruker BioSpin GmbH
4 Owner	nmr
5 Site	
6 Spectrometer	AVANCE NEO 400 MHZ DIGITAL NMR SPECTROMETER
7 Author	
8 Solvent	CDCl3
9 Temperature	294.3
10 Pulse Sequence	zgpg30
11 Experiment	1D
12 Number of Scans	79
13 Receiver Gain	48
14 Relaxation Delay	2.0000
15 Pulse Width	10.0000
16 Acquisition Time	1.3763
17 Acquisition Date	2018-01-18T11:47:28
18 Modification Date	2018-10-30T12:47:25
19 Spectrometer Frequency	100.61
20 Spectral Width	23809.5
21 Lowest Frequency	-834.8
22 Nucleus	13C
23 Acquired Size	32768
24 Spectral Size	32768

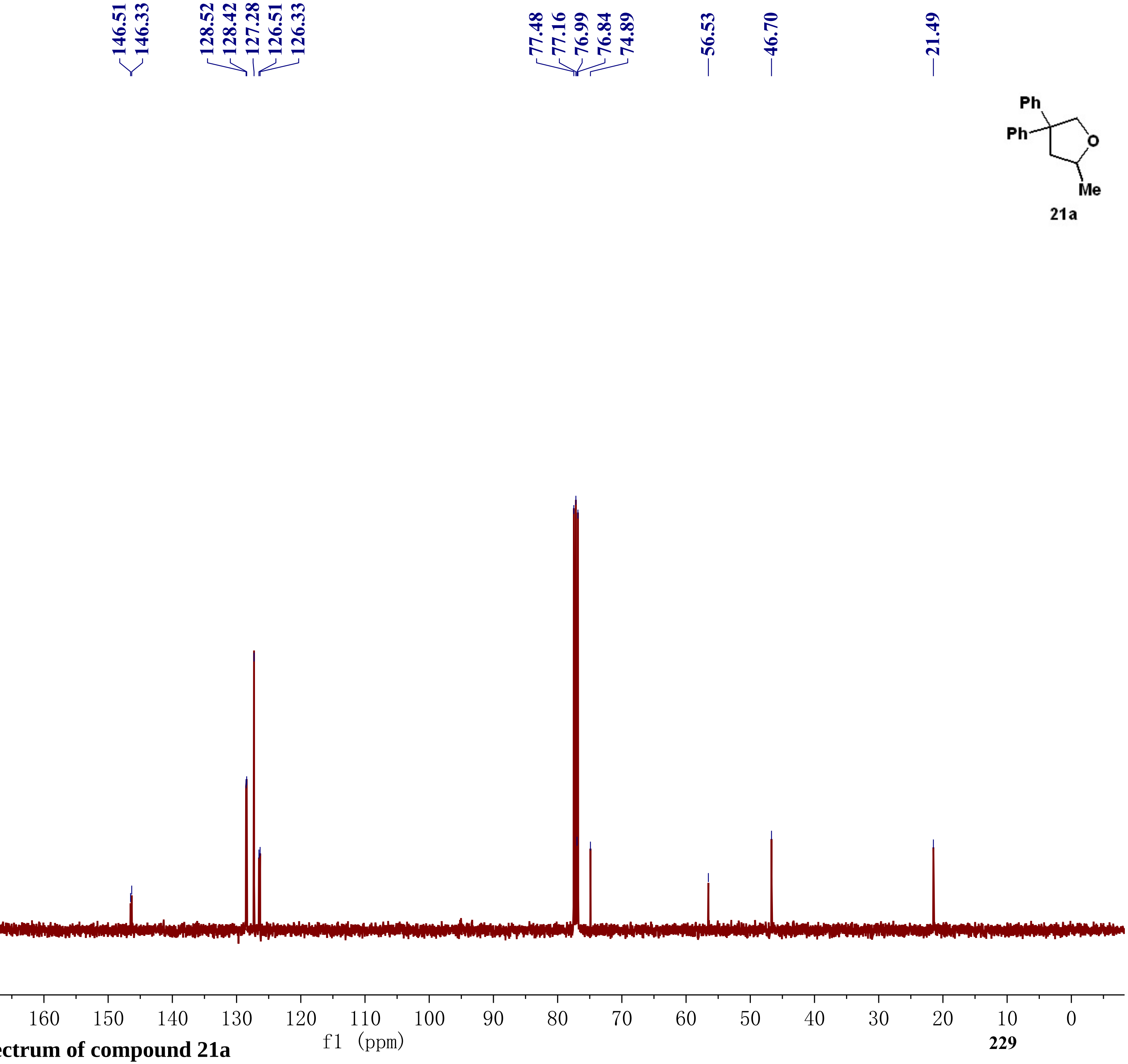

21a

Supplementary Figure 157. ^{13}C NMR spectrum of compound 21a

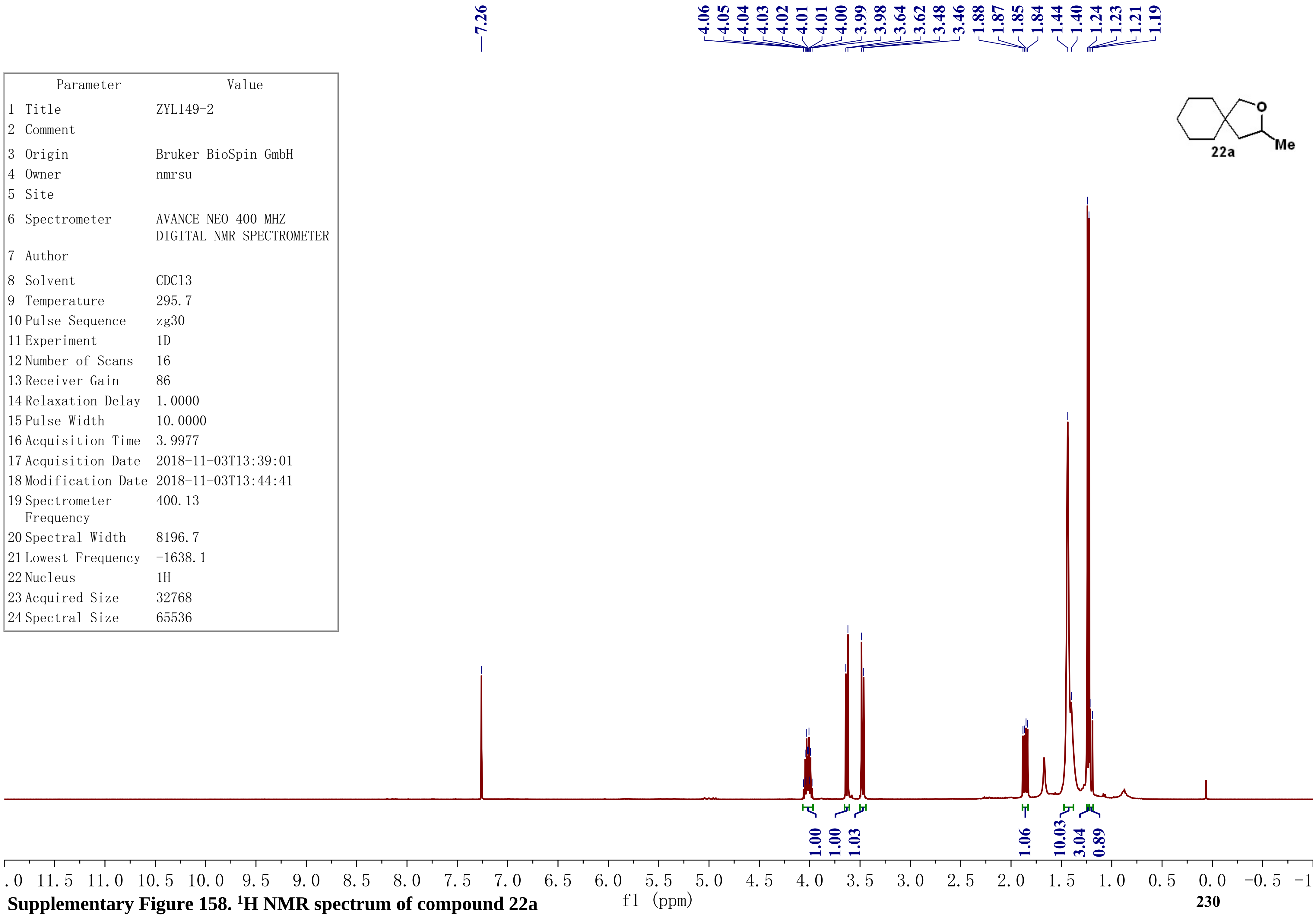

	Parameter	Value
1	Title	ZYL149-2
2	Comment	
3	Origin	Bruker BioSpin GmbH
4	Owner	nmrsu
5	Site	
6	Spectrometer	AVANCE NEO 400 MHZ DIGITAL NMR SPECTROMETER
7	Author	
8	Solvent	CDCl3
9	Temperature	295.7
10	Pulse Sequence	zg30
11	Experiment	1D
12	Number of Scans	16
13	Receiver Gain	86
14	Relaxation Delay	1.0000
15	Pulse Width	10.0000
16	Acquisition Time	3.9977
17	Acquisition Date	2018-11-03T13:39:01
18	Modification Date	2018-11-03T13:44:41
19	Spectrometer Frequency	400.13
20	Spectral Width	8196.7
21	Lowest Frequency	-1638.1
22	Nucleus	1H
23	Acquired Size	32768
24	Spectral Size	65536

Supplementary Figure 158. ^{1}H NMR spectrum of compound 22a

Parameter		Value
1	Title	ZYL149-2
2	Comment	
3	Origin	Bruker BioSpin GmbH
4	Owner	nmrsu
5	Site	
6	Spectrometer	AVANCE NEO 400 MHZ DIGITAL NMR SPECTROMETER
7	Author	
8	Solvent	CDCl3
9	Temperature	296.0
10	Pulse Sequence	zgpg30
11	Experiment	1D
12	Number of Scans	14
13	Receiver Gain	35
14	Relaxation Delay	2.0000
15	Pulse Width	10.0000
16	Acquisition Time	1.3763
17	Acquisition Date	2018-11-03T13:40:47
18	Modification Date	2018-11-03T13:44:41
19	Spectrometer Frequency	100.61
20	Spectral Width	23809.5
21	Lowest Frequency	-1831.1
22	Nucleus	13C
23	Acquired Size	32768
24	Spectral Size	32768

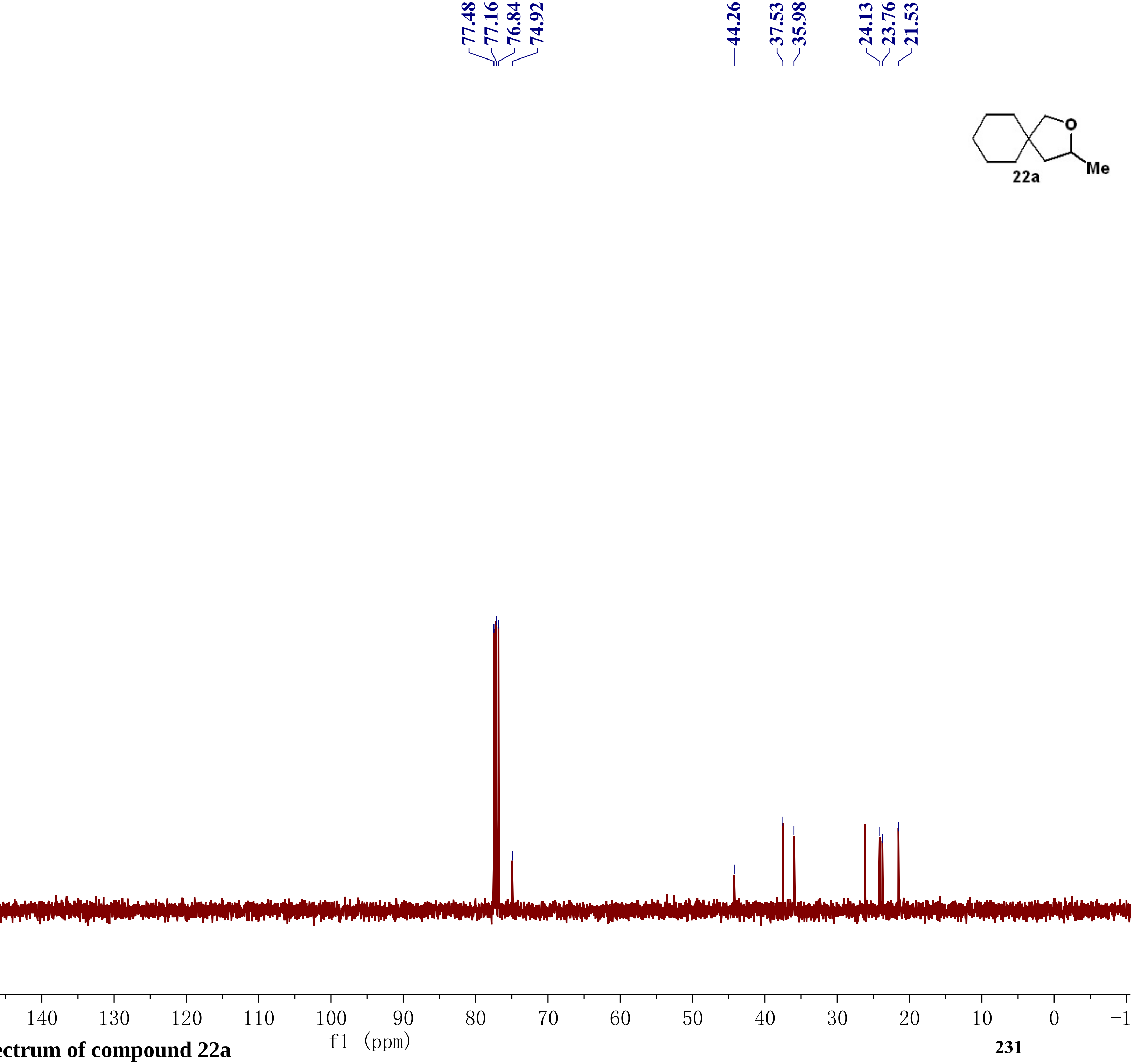

Supplementary Figure 159. ^{13}C NMR spectrum of compound 22a

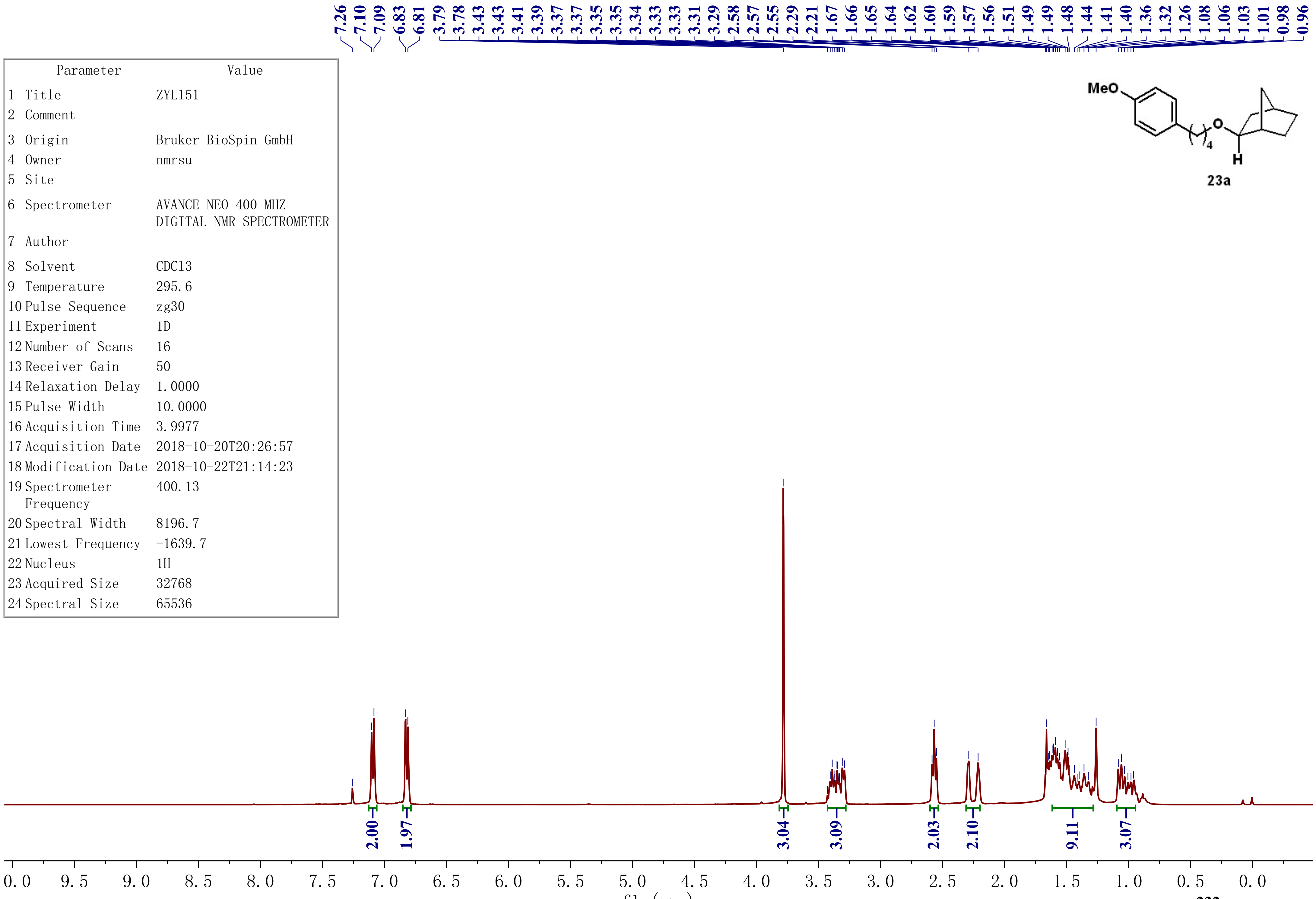

Parameter	Value
1 Title	ZYL151
2 Comment	
3 Origin	Bruker BioSpin GmbH
4 Owner	nmrsu
5 Site	
6 Spectrometer	AVANCE NEO 400 MHZ DIGITAL NMR SPECTROMETER
7 Author	
8 Solvent	CDCl3
9 Temperature	295.6
10 Pulse Sequence	zg30
11 Experiment	1D
12 Number of Scans	16
13 Receiver Gain	50
14 Relaxation Delay	1.0000
15 Pulse Width	10.0000
16 Acquisition Time	3.9977
17 Acquisition Date	2018-10-20T20:26:57
18 Modification Date	2018-10-22T21:14:23
19 Spectrometer Frequency	400.13
20 Spectral Width	8196.7
21 Lowest Frequency	-1639.7
22 Nucleus	1H
23 Acquired Size	32768
24 Spectral Size	65536

23a

Supplementary Figure 160. ¹H NMR spectrum of compound 23a

	Parameter	Value
1	Title	ZYL151C
2	Comment	
3	Origin	Bruker BioSpin GmbH
4	Owner	nmrsu
5	Site	
6	Spectrometer	AVANCE NEO 400 MHZ DIGITAL NMR SPECTROMETER
7	Author	
8	Solvent	CDCl3
9	Temperature	295.9
10	Pulse Sequence	zgpg30
11	Experiment	1D
12	Number of Scans	13
13	Receiver Gain	35
14	Relaxation Delay	2.0000
15	Pulse Width	10.0000
16	Acquisition Time	1.3763
17	Acquisition Date	2018-10-20T21:49:47
18	Modification Date	2018-10-22T21:14:26
19	Spectrometer Frequency	100.61
20	Spectral Width	23809.5
21	Lowest Frequency	-1833.6
22	Nucleus	13C
23	Acquired Size	32768
24	Spectral Size	32768

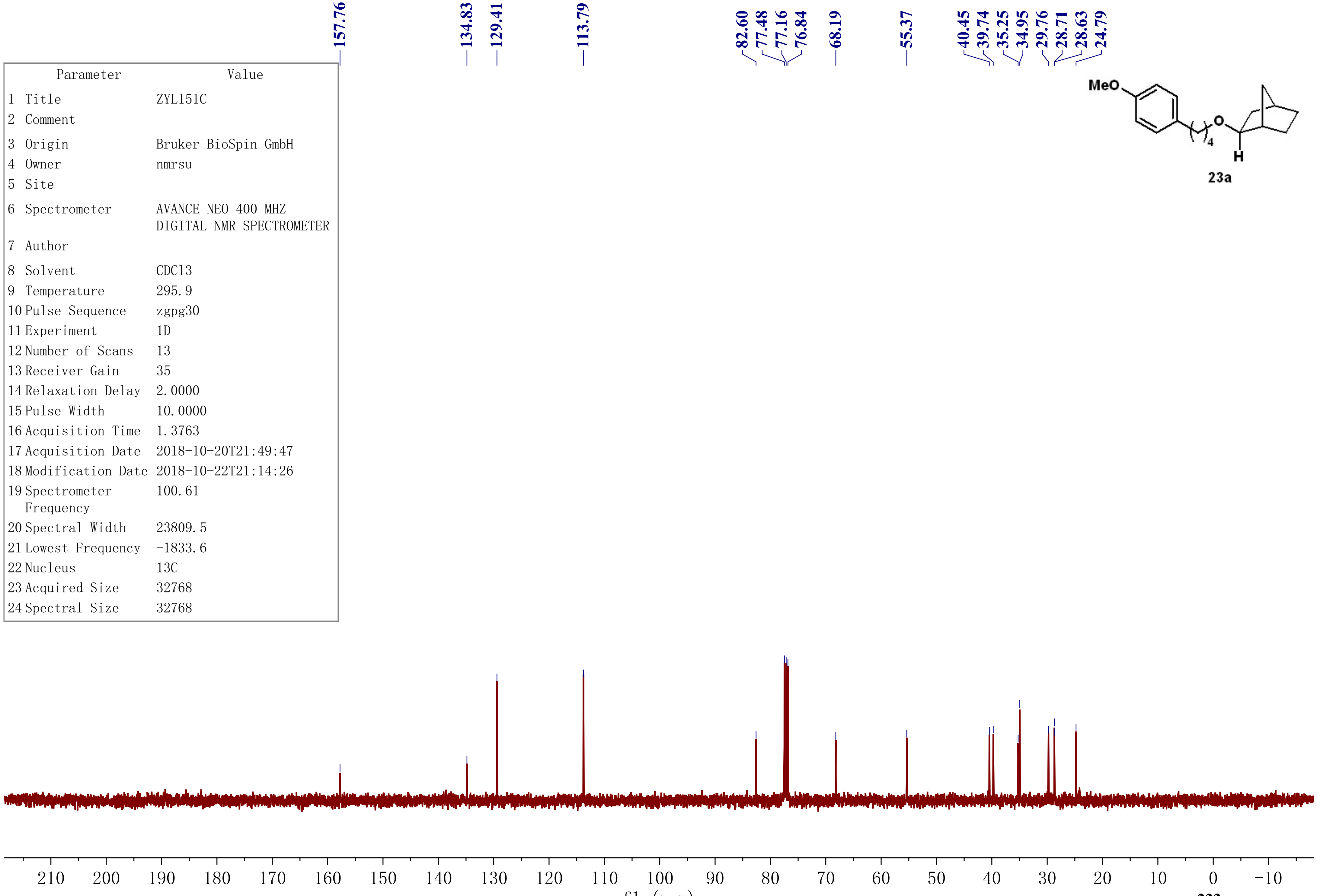

Supplementary Figure 161. ^{13}C **NMR spectrum of compound 23a**

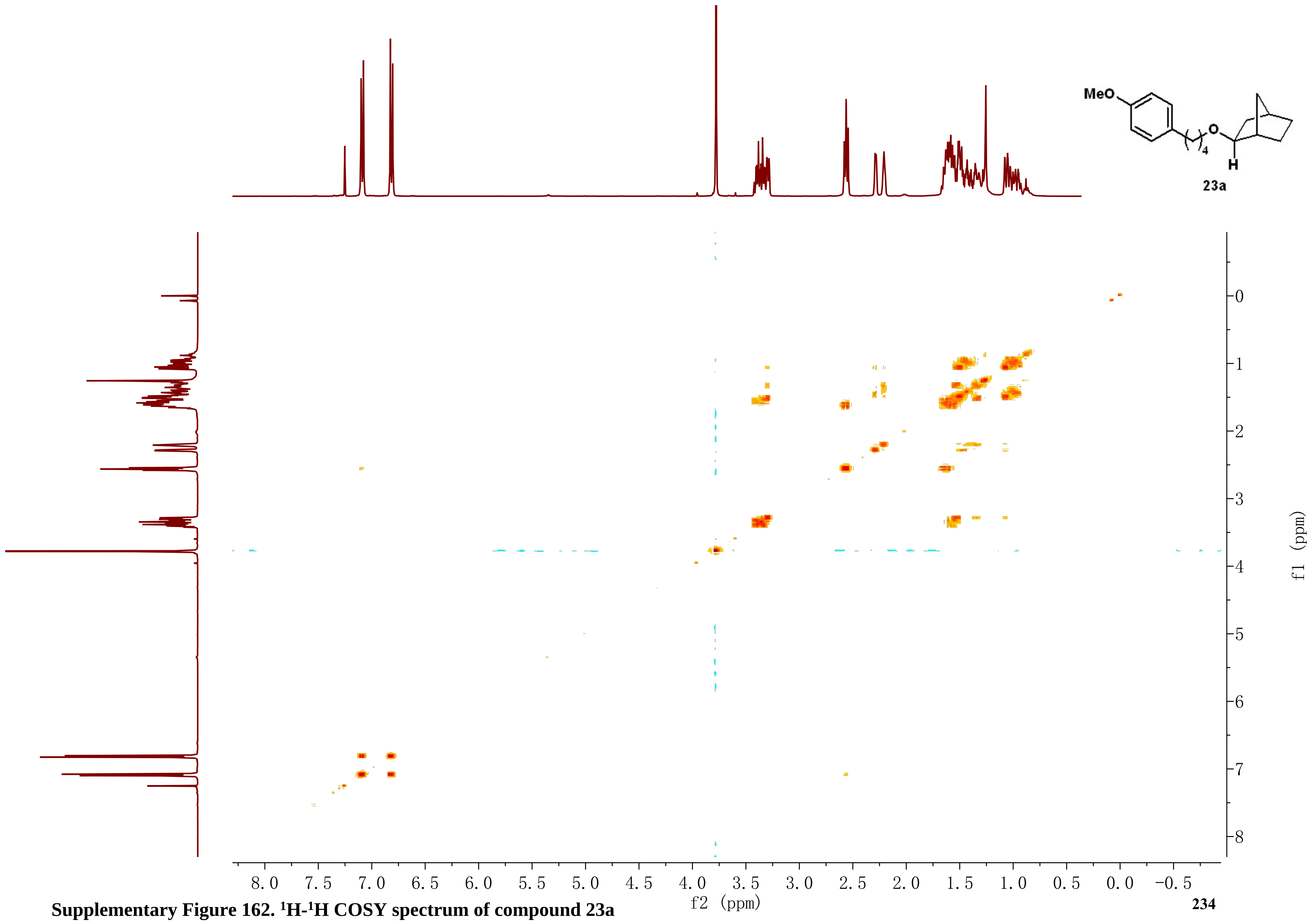

Supplementary Figure 162. ^1H-^1H COSY spectrum of compound 23a

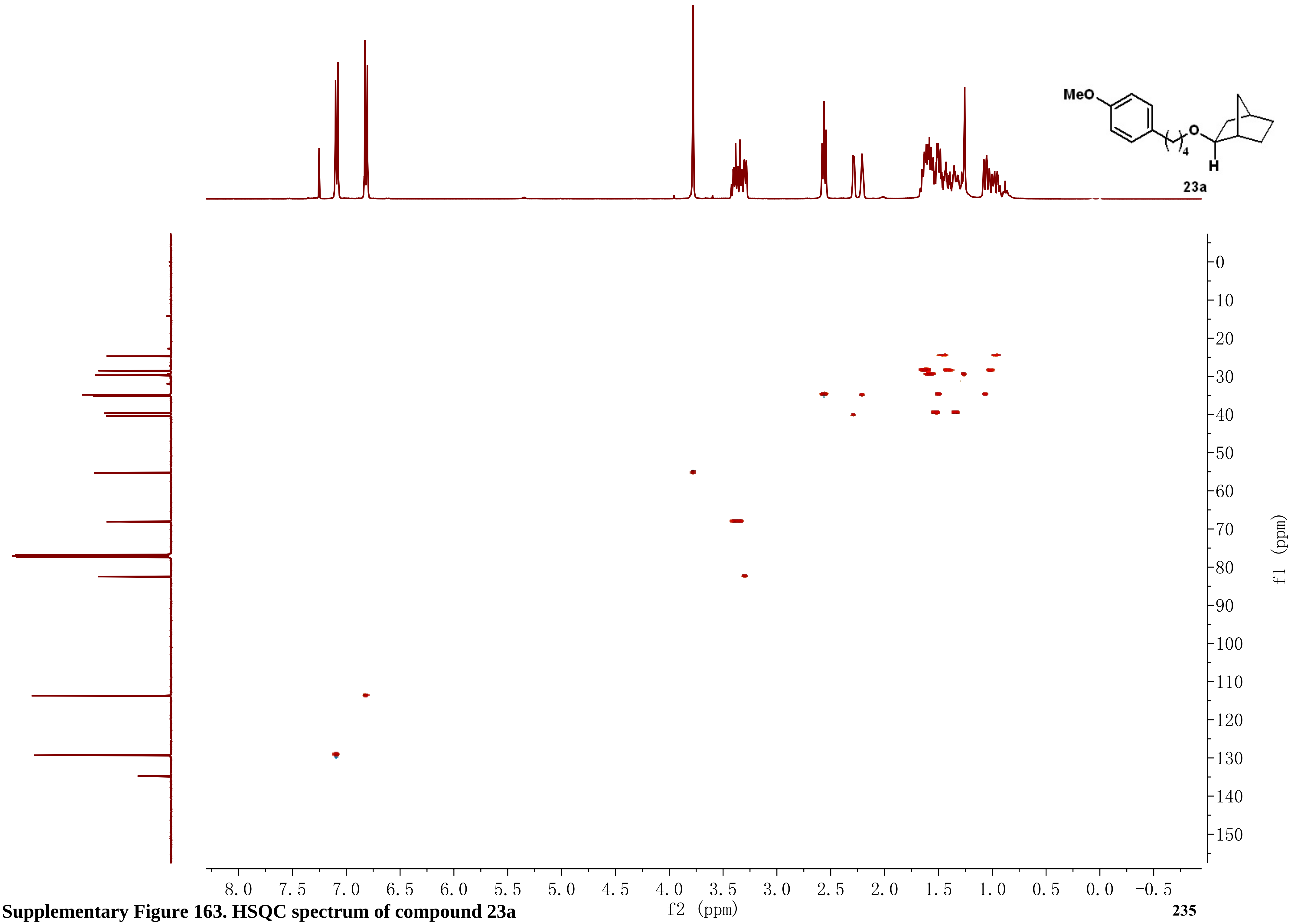

Supplementary Figure 163. HSQC spectrum of compound 23a

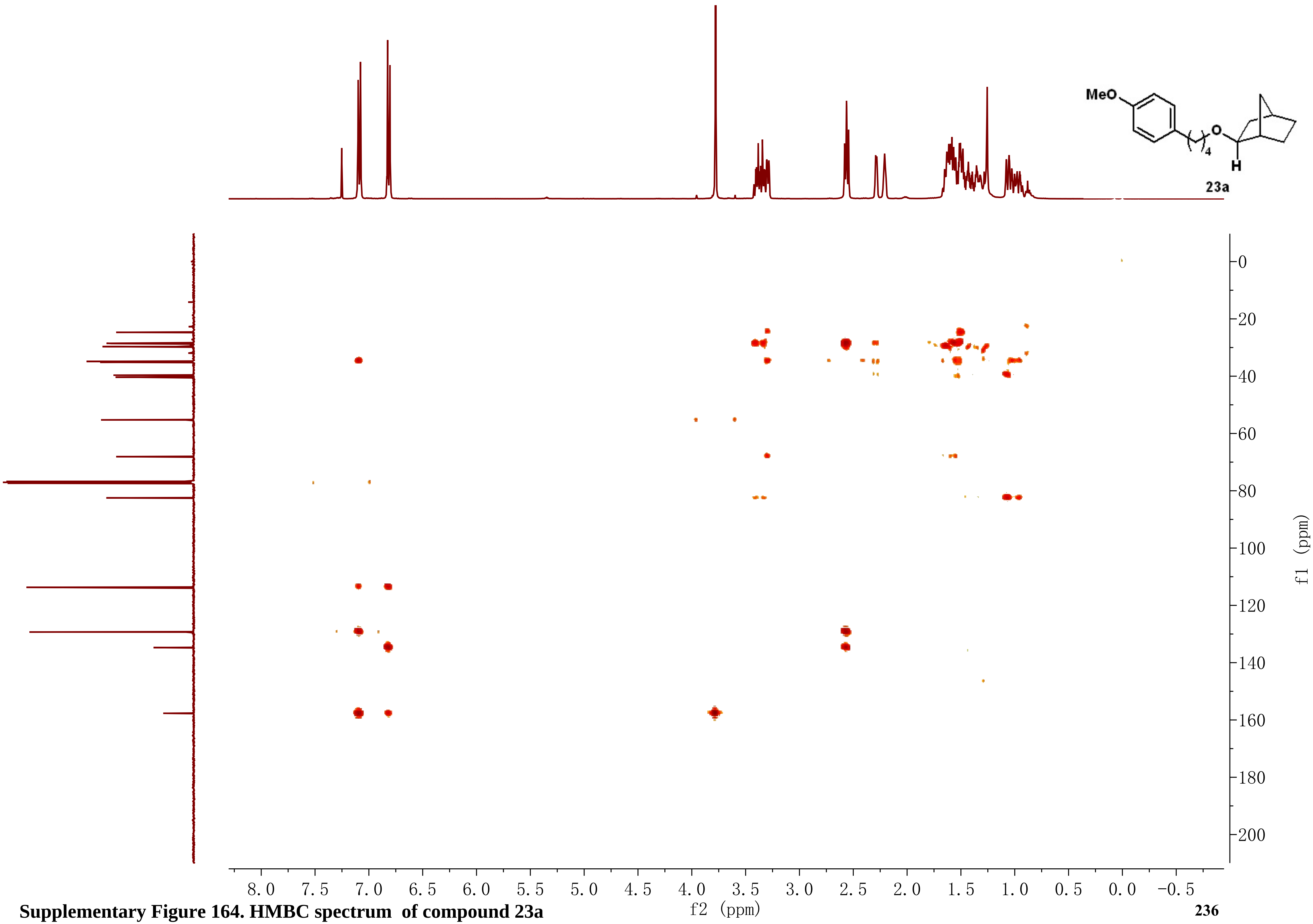

Supplementary Figure 164. HMBC spectrum of compound 23a

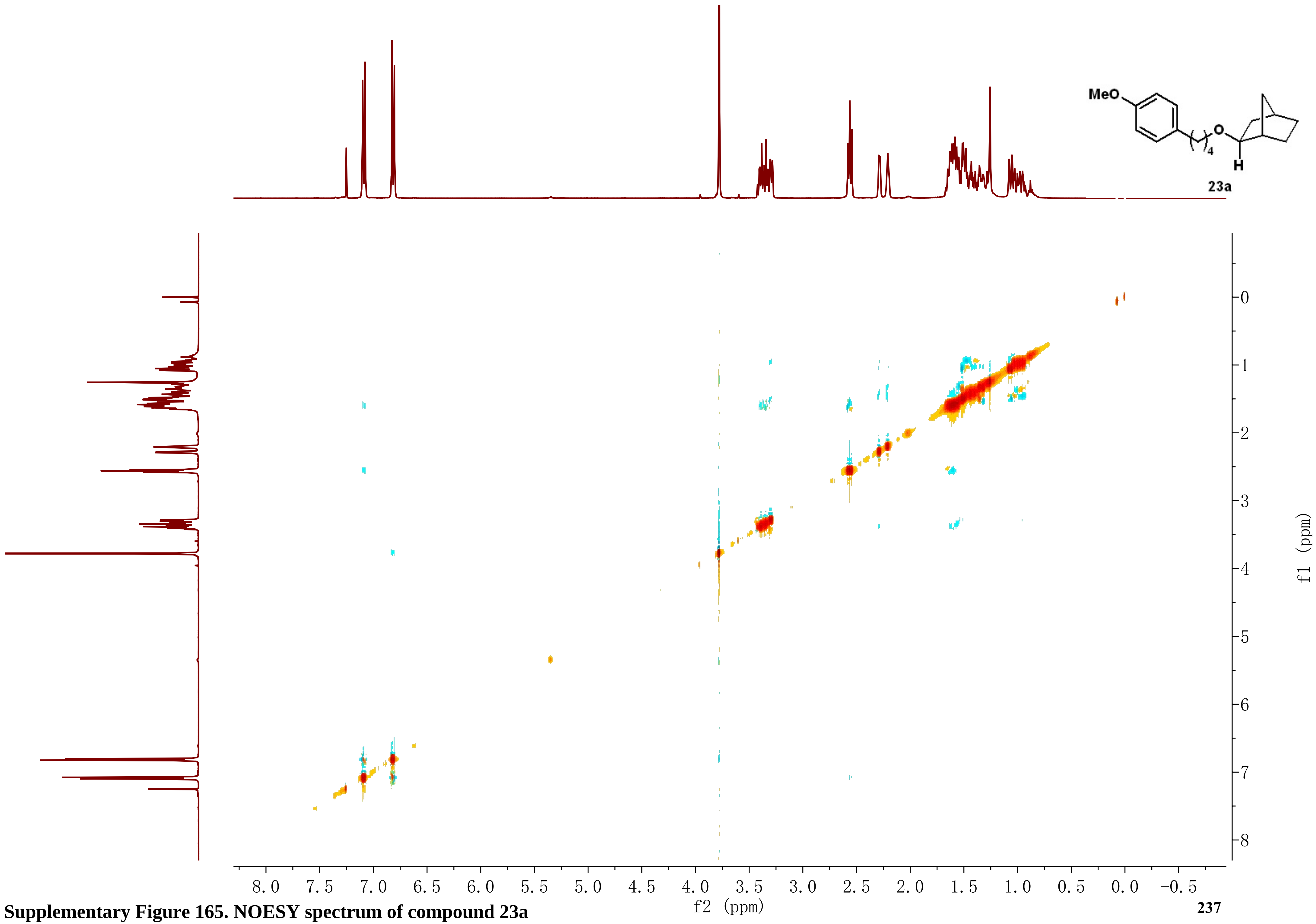

Supplementary Figure 165. NOESY spectrum of compound 23a

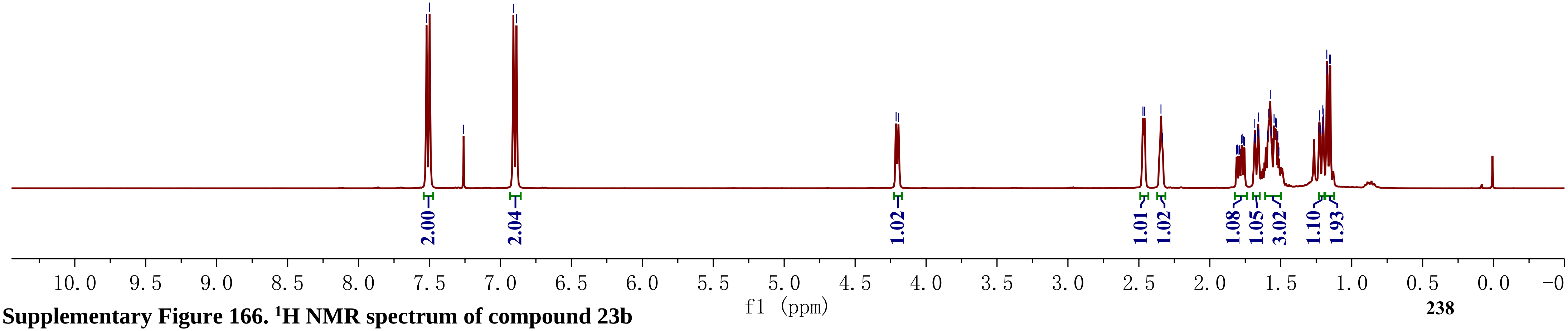

	Parameter	Value
1	Title	ZYLT167-4
2	Comment	
3	Origin	Bruker BioSpin GmbH
4	Owner	nmrsu
5	Site	
6	Spectrometer	AVANCE NEO 400 MHZ DIGITAL NMR SPECTROMETER
7	Author	
8	Solvent	CDCl3
9	Temperature	297.7
10	Pulse Sequence	zg30
11	Experiment	1D
12	Number of Scans	16
13	Receiver Gain	57
14	Relaxation Delay	1.0000
15	Pulse Width	10.0000
16	Acquisition Time	3.9977
17	Acquisition Date	2019-08-08T20:51:56
18	Modification Date	2019-08-08T20:57:18
19	Spectrometer Frequency	400.13
20	Spectral Width	8196.7
21	Lowest Frequency	-1638.1
22	Nucleus	1H
23	Acquired Size	32768
24	Spectral Size	65536

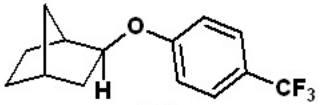

23b

Supplementary Figure 166. ^{1}H NMR spectrum of compound 23b

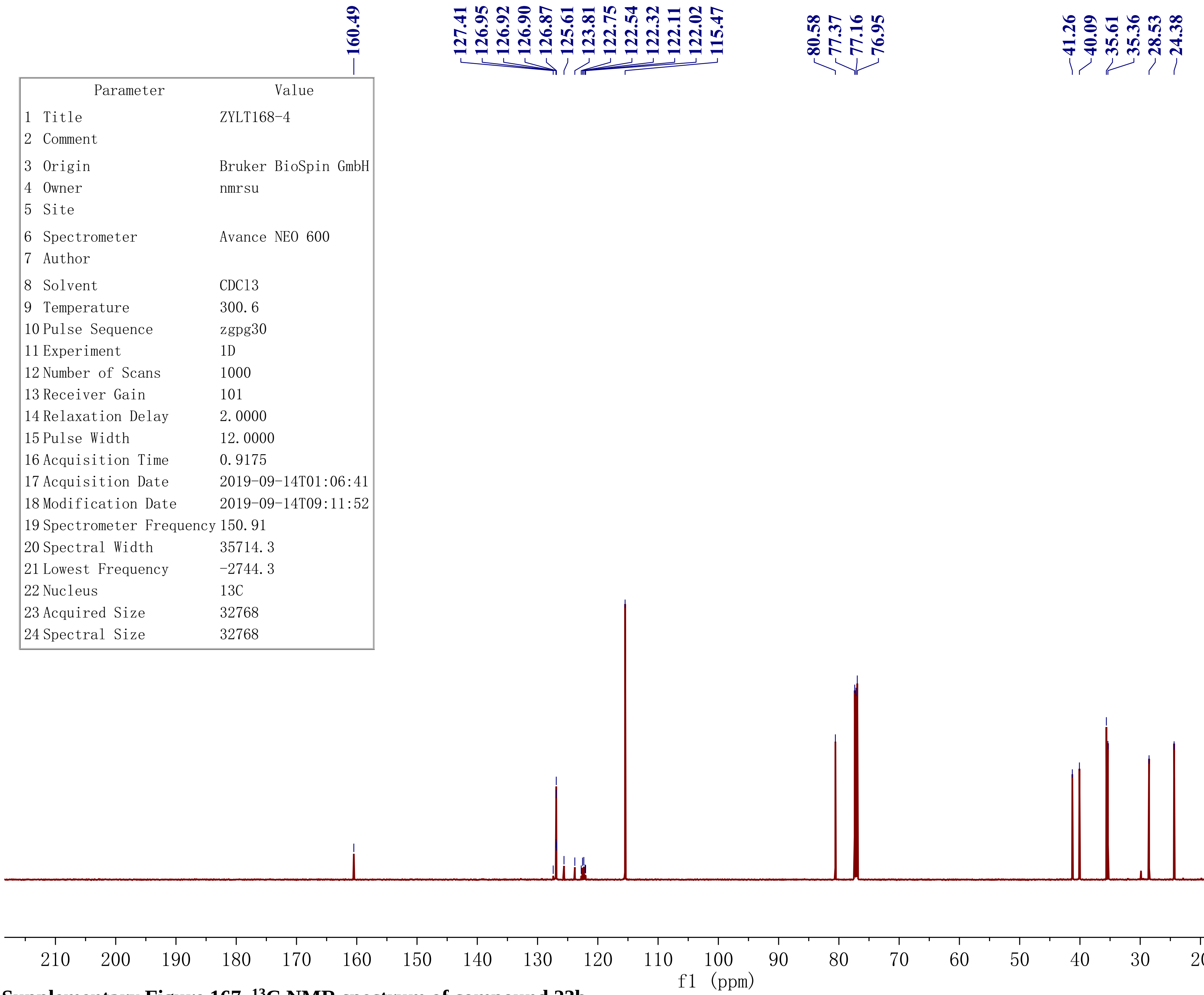

	Parameter	Value
1	Title	ZYLT168-4
2	Comment	
3	Origin	Bruker BioSpin GmbH
4	Owner	nmrsu
5	Site	
6	Spectrometer	Avance NEO 600
7	Author	
8	Solvent	CDCl3
9	Temperature	300.6
10	Pulse Sequence	zgpg30
11	Experiment	1D
12	Number of Scans	1000
13	Receiver Gain	101
14	Relaxation Delay	2.0000
15	Pulse Width	12.0000
16	Acquisition Time	0.9175
17	Acquisition Date	2019-09-14T01:06:41
18	Modification Date	2019-09-14T09:11:52
19	Spectrometer Frequency	150.91
20	Spectral Width	35714.3
21	Lowest Frequency	-2744.3
22	Nucleus	13C
23	Acquired Size	32768
24	Spectral Size	32768

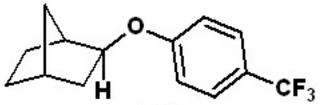

23b

Supplementary Figure 167. ^{13}C NMR spectrum of compound 23b

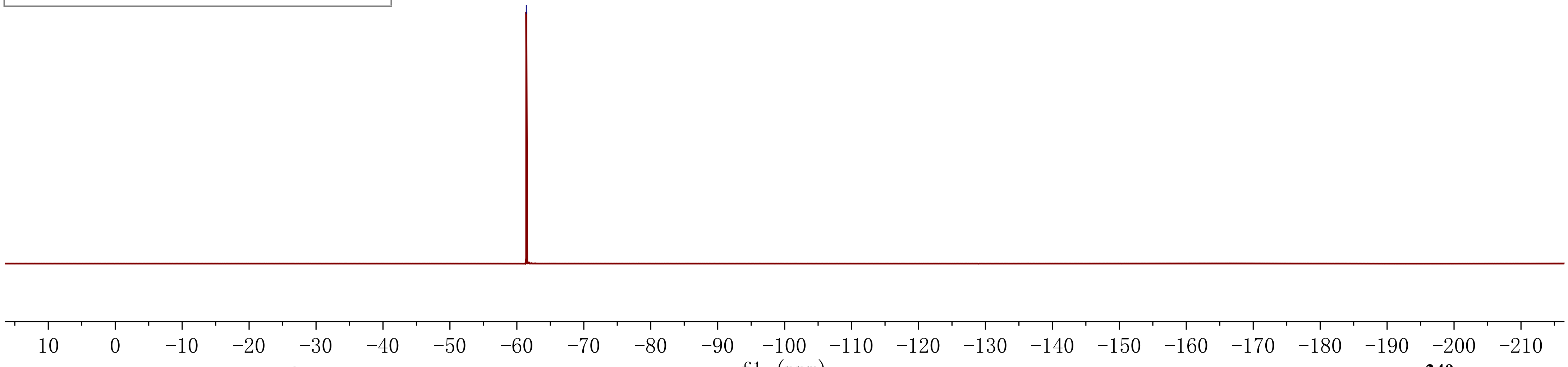

	Parameter	Value
1	Title	ZYLT168-4
2	Comment	
3	Origin	Bruker BioSpin GmbH
4	Owner	nmrsu
5	Site	
6	Spectrometer	Avance NEO 600
7	Author	
8	Solvent	CDCl3
9	Temperature	299.7
10	Pulse Sequence	zgig
11	Experiment	1D
12	Number of Scans	16
13	Receiver Gain	101
14	Relaxation Delay	1.0000
15	Pulse Width	12.0000
16	Acquisition Time	0.4981
17	Acquisition Date	2019-09-14T01:08:09
18	Modification Date	2019-09-14T09:11:53
19	Spectrometer Frequency	564.71
20	Spectral Width	131579.0
21	Lowest Frequency	-122260.0
22	Nucleus	19F
23	Acquired Size	65536
24	Spectral Size	65536

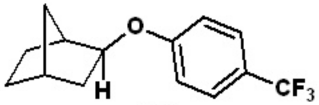

23b

Supplementary Figure 168. ^{19}F NMR spectrum of compound 23b

Parameter	Value
1 Title	ZYLT167-1
2 Comment	
3 Origin	Bruker BioSpin GmbH
4 Owner	nmrsu
5 Site	
6 Spectrometer	AVANCE NEO 400 MHZ DIGITAL NMR SPECTROMETER
7 Author	
8 Solvent	CDCl3
9 Temperature	297.2
10 Pulse Sequence	zg30
11 Experiment	1D
12 Number of Scans	16
13 Receiver Gain	92
14 Relaxation Delay	1.0000
15 Pulse Width	10.0000
16 Acquisition Time	3.9977
17 Acquisition Date	2019-09-04T05:21:26
18 Modification Date	2019-09-04T18:35:08
19 Spectrometer Frequency	400.13
20 Spectral Width	8196.7
21 Lowest Frequency	-1638.1
22 Nucleus	1H
23 Acquired Size	32768
24 Spectral Size	65536

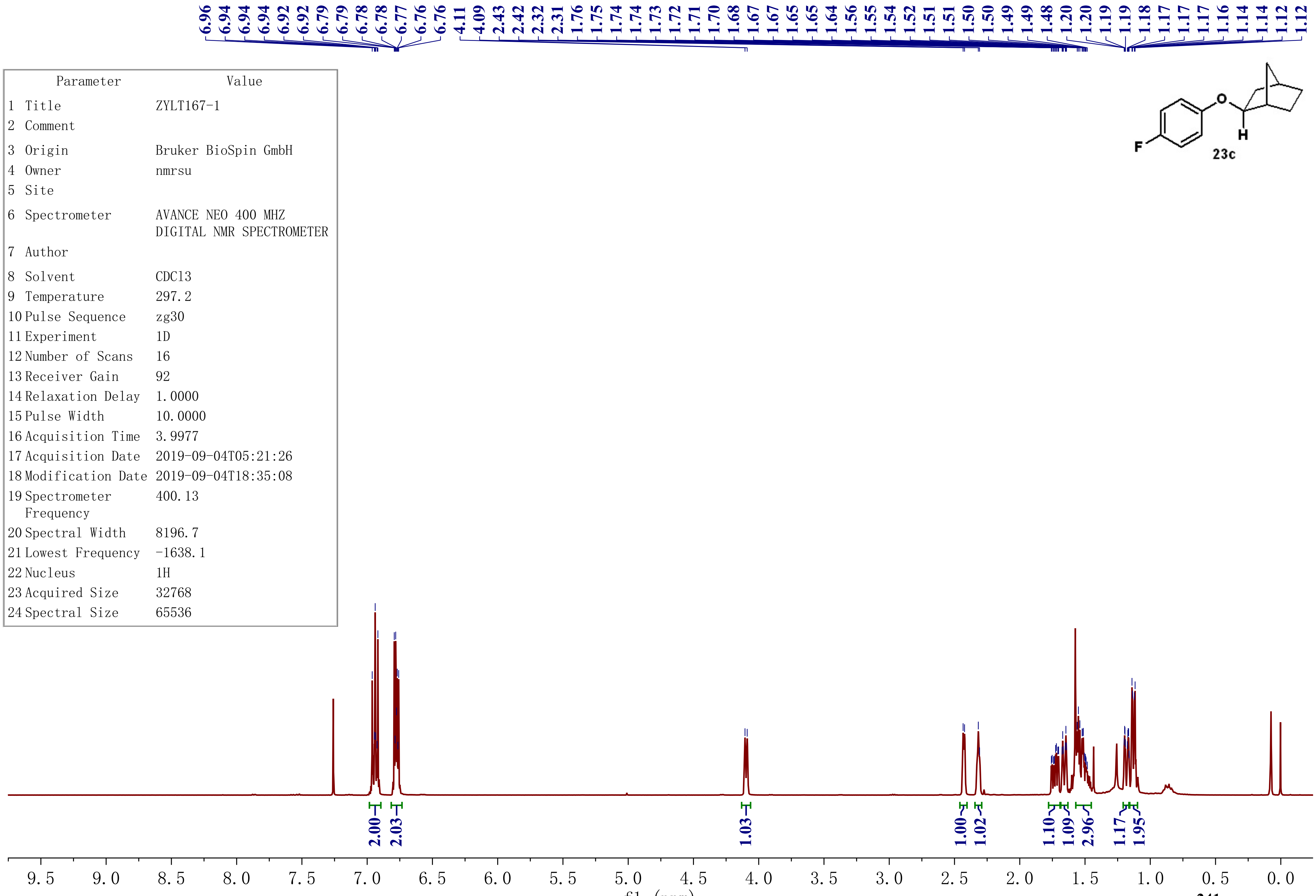

Supplementary Figure 169. ^{1}H NMR spectrum of compound 23c

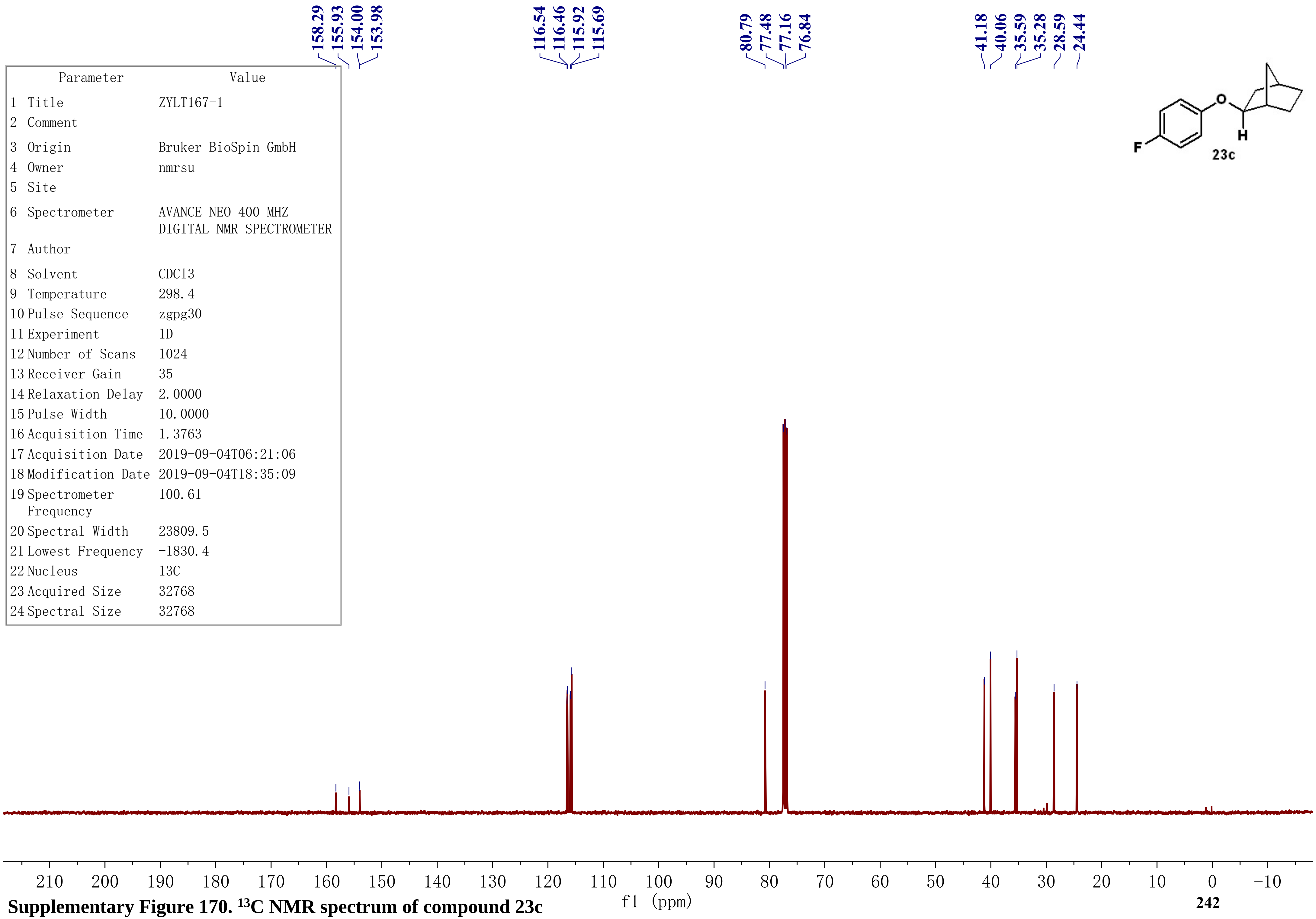

23c

Supplementary Figure 170. ^{13}C NMR spectrum of compound 23c

	Parameter	Value
1	Title	ZYLT167-1
2	Comment	
3	Origin	Bruker BioSpin GmbH
4	Owner	nmrsu
5	Site	
6	Spectrometer	AVANCE NEO 400 MHZ DIGITAL NMR SPECTROMETER
7	Author	
8	Solvent	CDCl3
9	Temperature	298.1
10	Pulse Sequence	zgig
11	Experiment	1D
12	Number of Scans	16
13	Receiver Gain	101
14	Relaxation Delay	1.0000
15	Pulse Width	18.0000
16	Acquisition Time	0.7209
17	Acquisition Date	2019-09-04T06:22:33
18	Modification Date	2019-09-04T18:35:09
19	Spectrometer Frequency	376.50
20	Spectral Width	90909.1
21	Lowest Frequency	-83104.4
22	Nucleus	19F
23	Acquired Size	65536
24	Spectral Size	65536

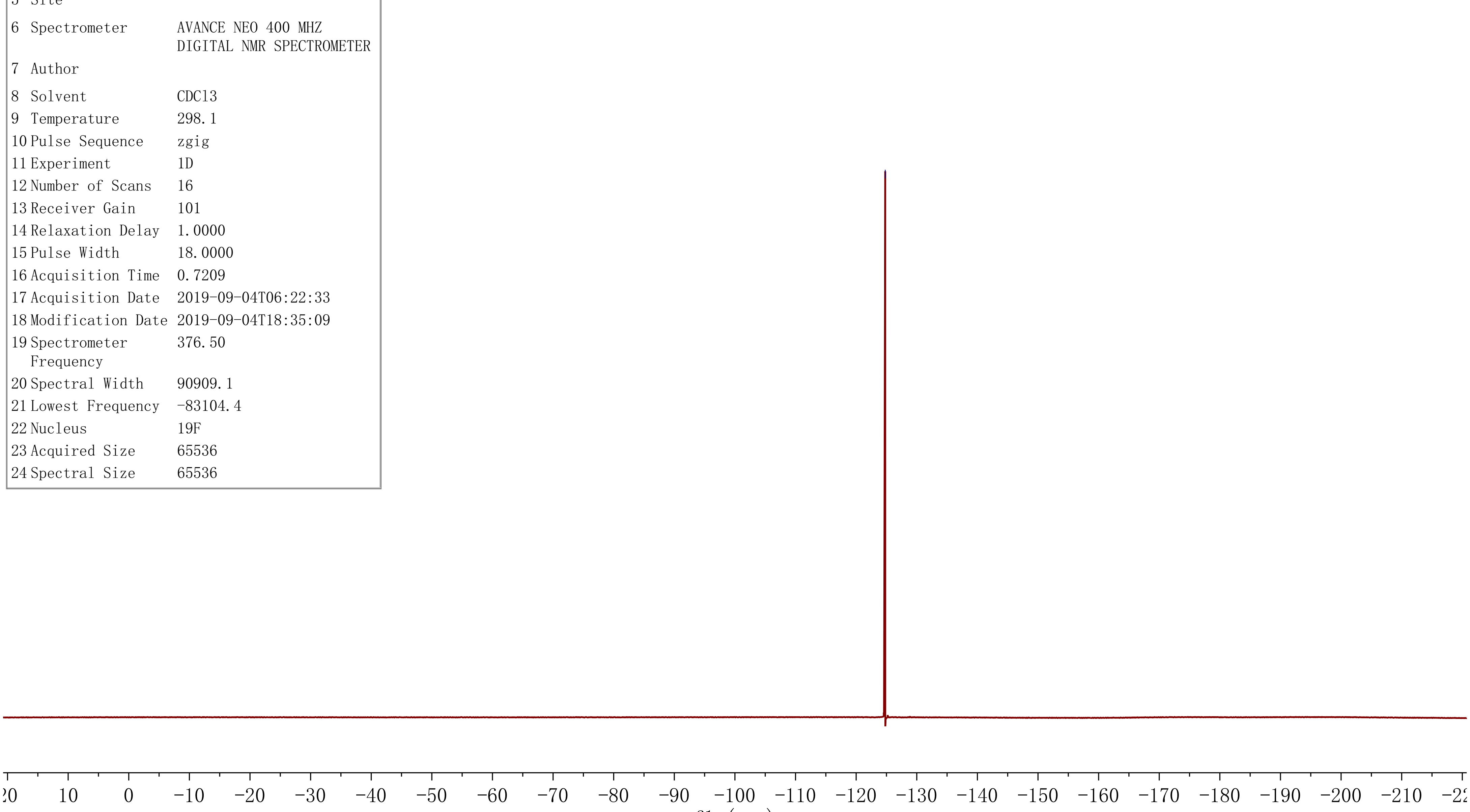

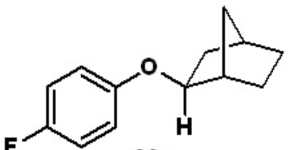

23c

Supplementary Figure 171. ^{19}F NMR spectrum of compound 23c

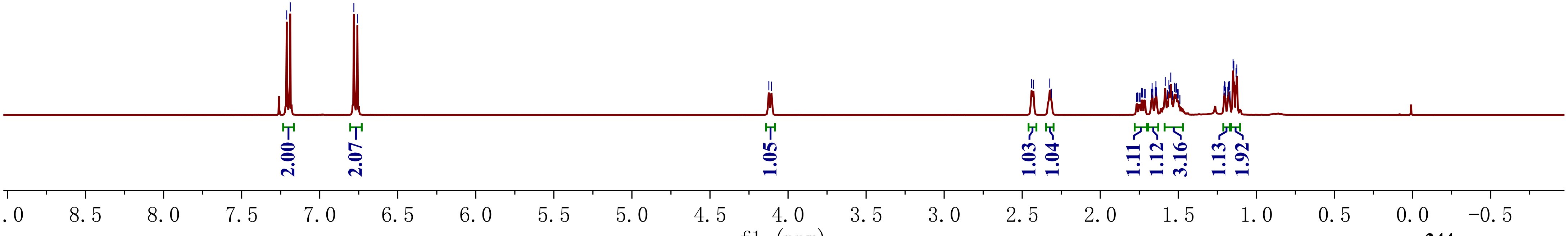

Parameter	Value
1 Title	ZYLT167-2
2 Comment	
3 Origin	Bruker BioSpin GmbH
4 Owner	nmrsu
5 Site	
6 Spectrometer	AVANCE NEO 400 MHZ DIGITAL NMR SPECTROMETER
7 Author	
8 Solvent	CDCl3
9 Temperature	297.3
10 Pulse Sequence	zg30
11 Experiment	1D
12 Number of Scans	16
13 Receiver Gain	57
14 Relaxation Delay	1.0000
15 Pulse Width	10.0000
16 Acquisition Time	3.9977
17 Acquisition Date	2019-08-08T20:38:42
18 Modification Date	2019-08-08T20:57:19
19 Spectrometer Frequency	400.13
20 Spectral Width	8196.7
21 Lowest Frequency	-1637.8
22 Nucleus	1H
23 Acquired Size	32768
24 Spectral Size	65536

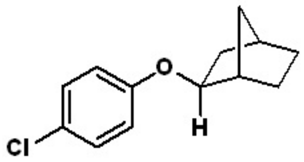

23d

Supplementary Figure 172. ^1H NMR spectrum of compound 23d

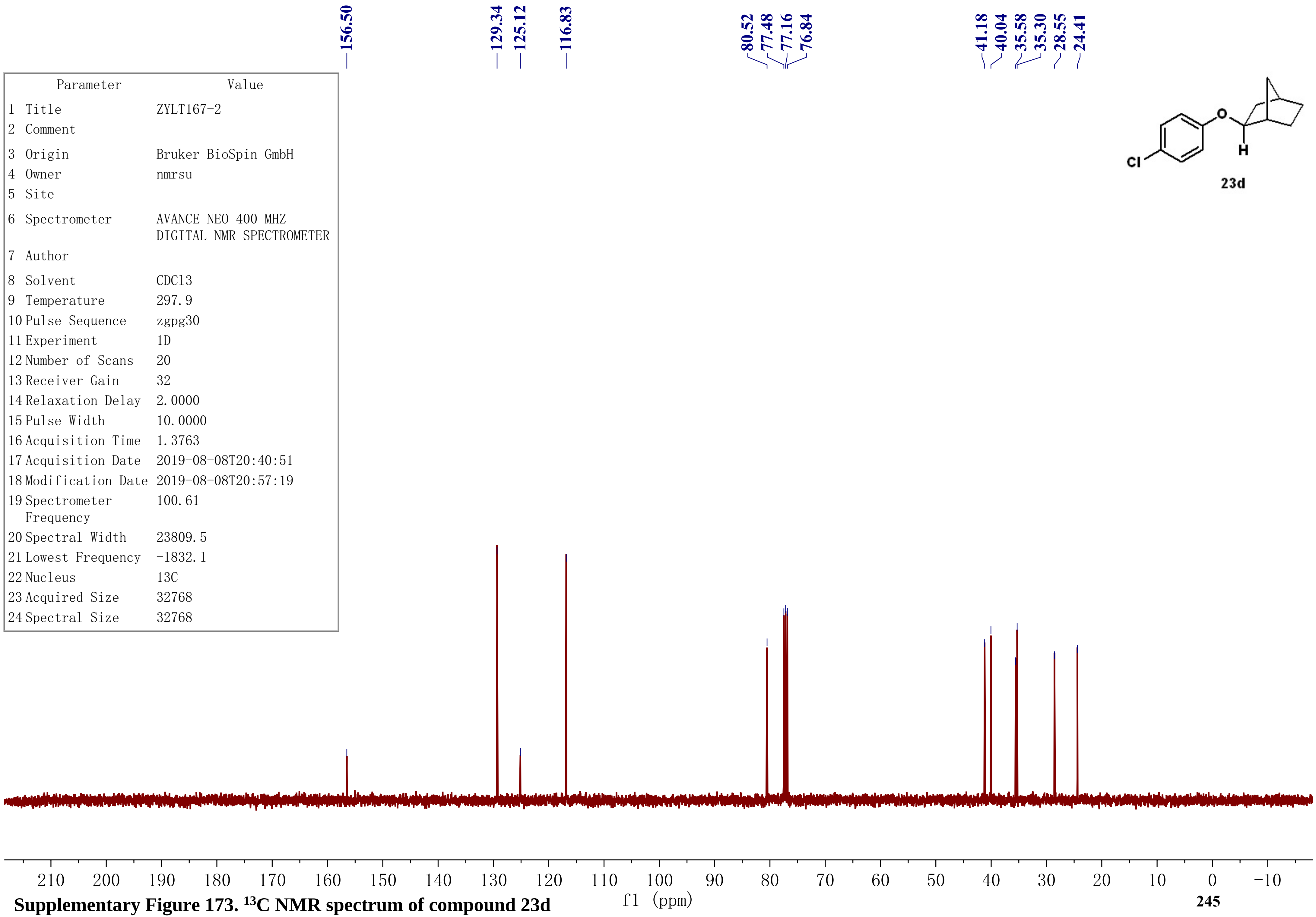

	Parameter	Value
1	Title	ZYLT167-2
2	Comment	
3	Origin	Bruker BioSpin GmbH
4	Owner	nmrsu
5	Site	
6	Spectrometer	AVANCE NEO 400 MHZ DIGITAL NMR SPECTROMETER
7	Author	
8	Solvent	CDCl3
9	Temperature	297.9
10	Pulse Sequence	zgpg30
11	Experiment	1D
12	Number of Scans	20
13	Receiver Gain	32
14	Relaxation Delay	2.0000
15	Pulse Width	10.0000
16	Acquisition Time	1.3763
17	Acquisition Date	2019-08-08T20:40:51
18	Modification Date	2019-08-08T20:57:19
19	Spectrometer Frequency	100.61
20	Spectral Width	23809.5
21	Lowest Frequency	-1832.1
22	Nucleus	13C
23	Acquired Size	32768
24	Spectral Size	32768

Supplementary Figure 173. ^{13}C NMR spectrum of compound 23d

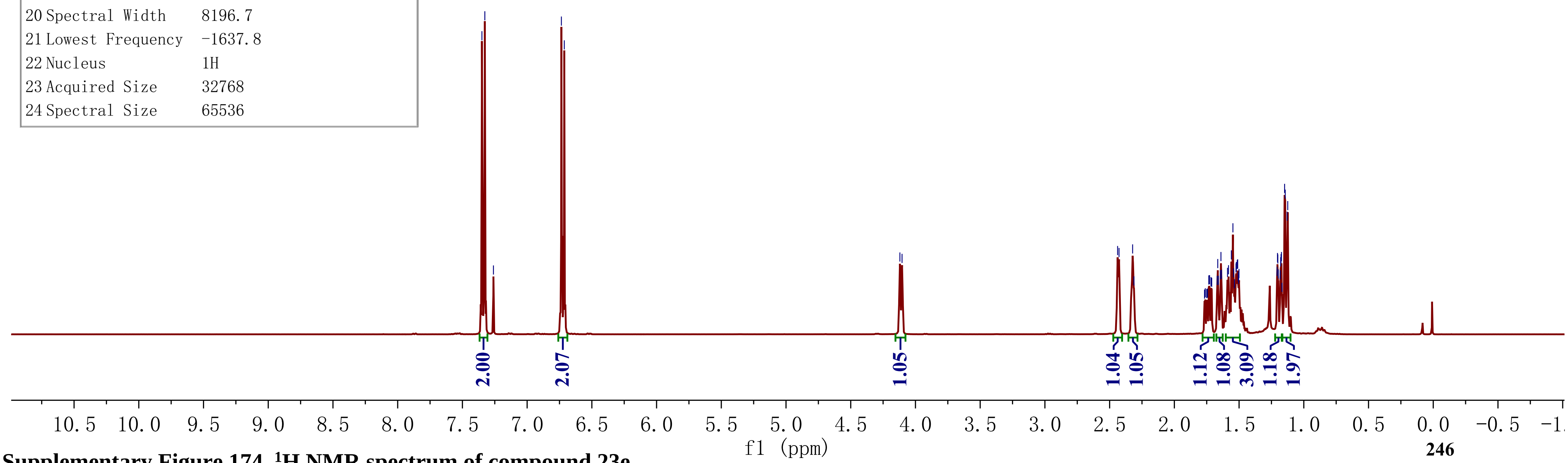

Parameter	Value
1 Title	ZYLT167-3
2 Comment	
3 Origin	Bruker BioSpin GmbH
4 Owner	nmrsu
5 Site	
6 Spectrometer	AVANCE NEO 400 MHZ DIGITAL NMR SPECTROMETER
7 Author	
8 Solvent	CDCl3
9 Temperature	297.5
10 Pulse Sequence	zg30
11 Experiment	1D
12 Number of Scans	16
13 Receiver Gain	55
14 Relaxation Delay	1.0000
15 Pulse Width	10.0000
16 Acquisition Time	3.9977
17 Acquisition Date	2019-08-08T20:45:18
18 Modification Date	2019-08-08T20:57:18
19 Spectrometer Frequency	400.13
20 Spectral Width	8196.7
21 Lowest Frequency	-1637.8
22 Nucleus	1H
23 Acquired Size	32768
24 Spectral Size	65536

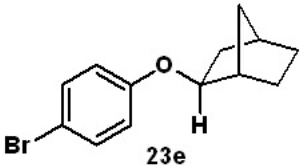

23e

Supplementary Figure 174. ^{1}H NMR spectrum of compound 23e

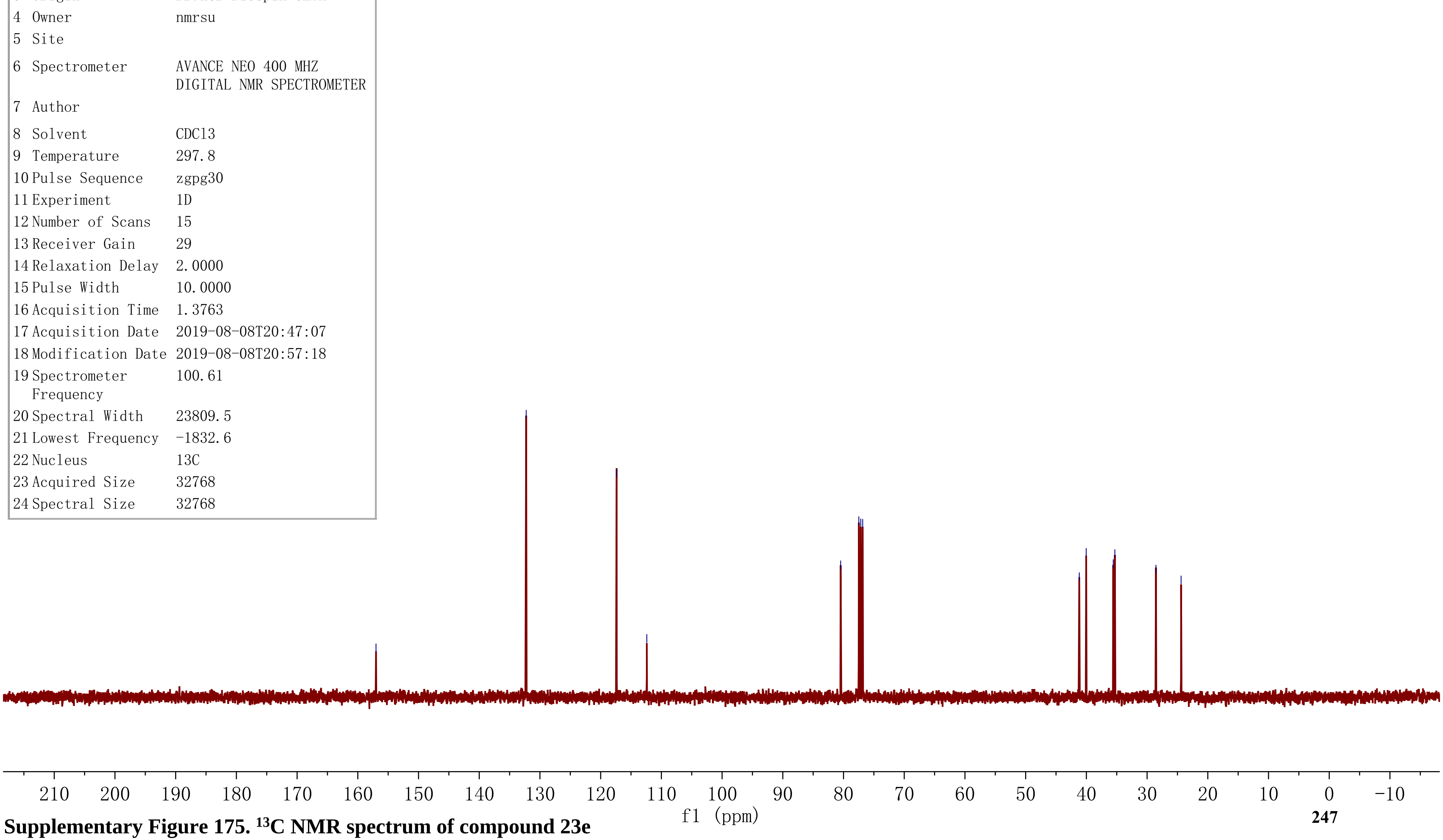

Parameter	Value
1 Title	ZYLT167-3
2 Comment	
3 Origin	Bruker BioSpin GmbH
4 Owner	nmrsu
5 Site	
6 Spectrometer	AVANCE NEO 400 MHZ DIGITAL NMR SPECTROMETER
7 Author	
8 Solvent	CDCl3
9 Temperature	297.8
10 Pulse Sequence	zgpg30
11 Experiment	1D
12 Number of Scans	15
13 Receiver Gain	29
14 Relaxation Delay	2.0000
15 Pulse Width	10.0000
16 Acquisition Time	1.3763
17 Acquisition Date	2019-08-08T20:47:07
18 Modification Date	2019-08-08T20:57:18
19 Spectrometer Frequency	100.61
20 Spectral Width	23809.5
21 Lowest Frequency	-1832.6
22 Nucleus	13C
23 Acquired Size	32768
24 Spectral Size	32768

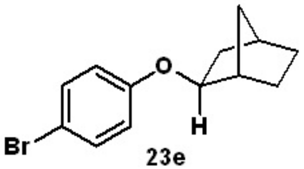

23e

Supplementary Figure 175. ^{13}C **NMR spectrum of compound 23e**

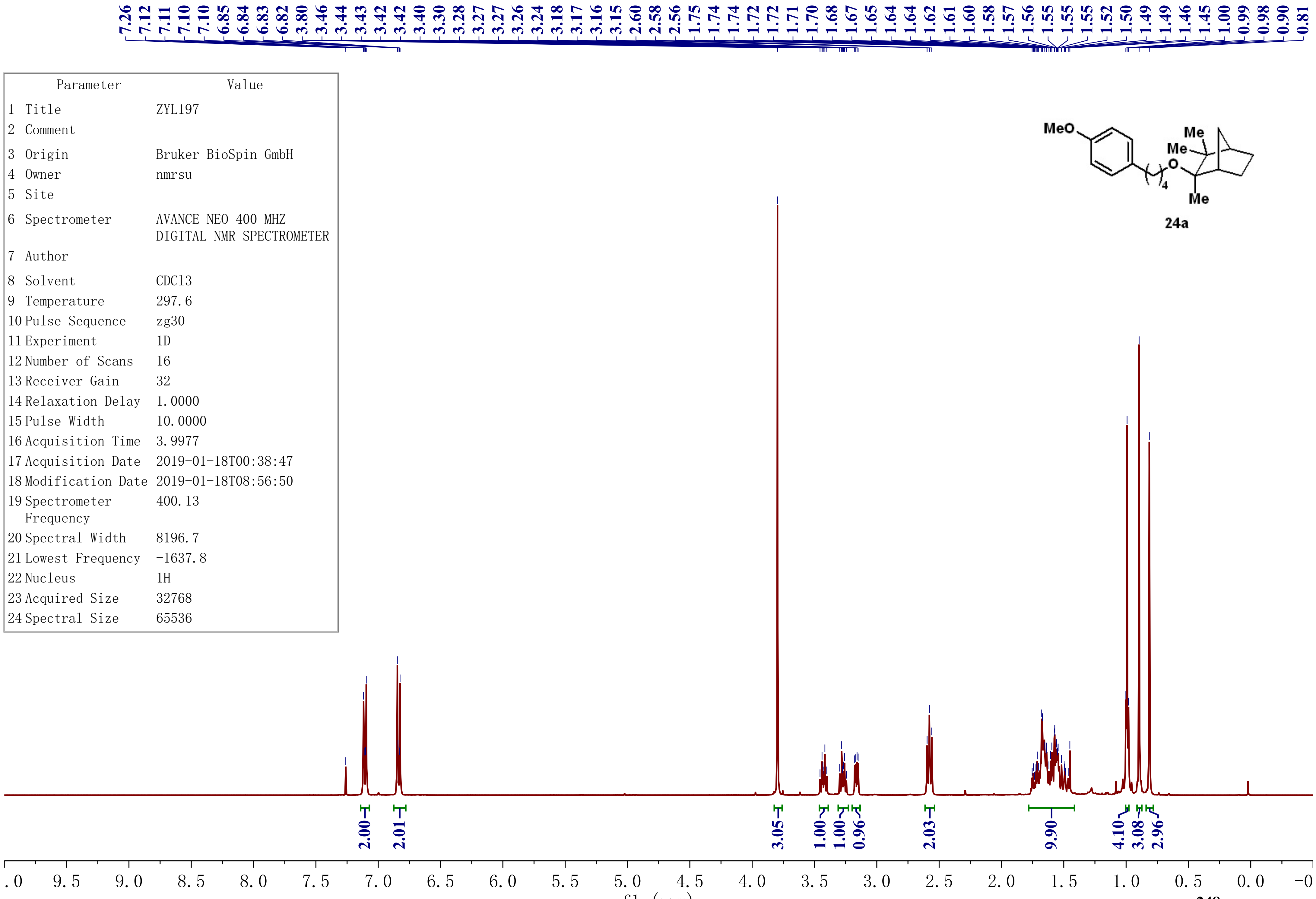

Parameter	Value
1 Title	ZYL197
2 Comment	
3 Origin	Bruker BioSpin GmbH
4 Owner	nmrsu
5 Site	
6 Spectrometer	AVANCE NEO 400 MHZ DIGITAL NMR SPECTROMETER
7 Author	
8 Solvent	CDCl3
9 Temperature	297.6
10 Pulse Sequence	zg30
11 Experiment	1D
12 Number of Scans	16
13 Receiver Gain	32
14 Relaxation Delay	1.0000
15 Pulse Width	10.0000
16 Acquisition Time	3.9977
17 Acquisition Date	2019-01-18T00:38:47
18 Modification Date	2019-01-18T08:56:50
19 Spectrometer Frequency	400.13
20 Spectral Width	8196.7
21 Lowest Frequency	-1637.8
22 Nucleus	1H
23 Acquired Size	32768
24 Spectral Size	65536

24a

Supplementary Figure 176. ^{1}H NMR spectrum of compound 24a

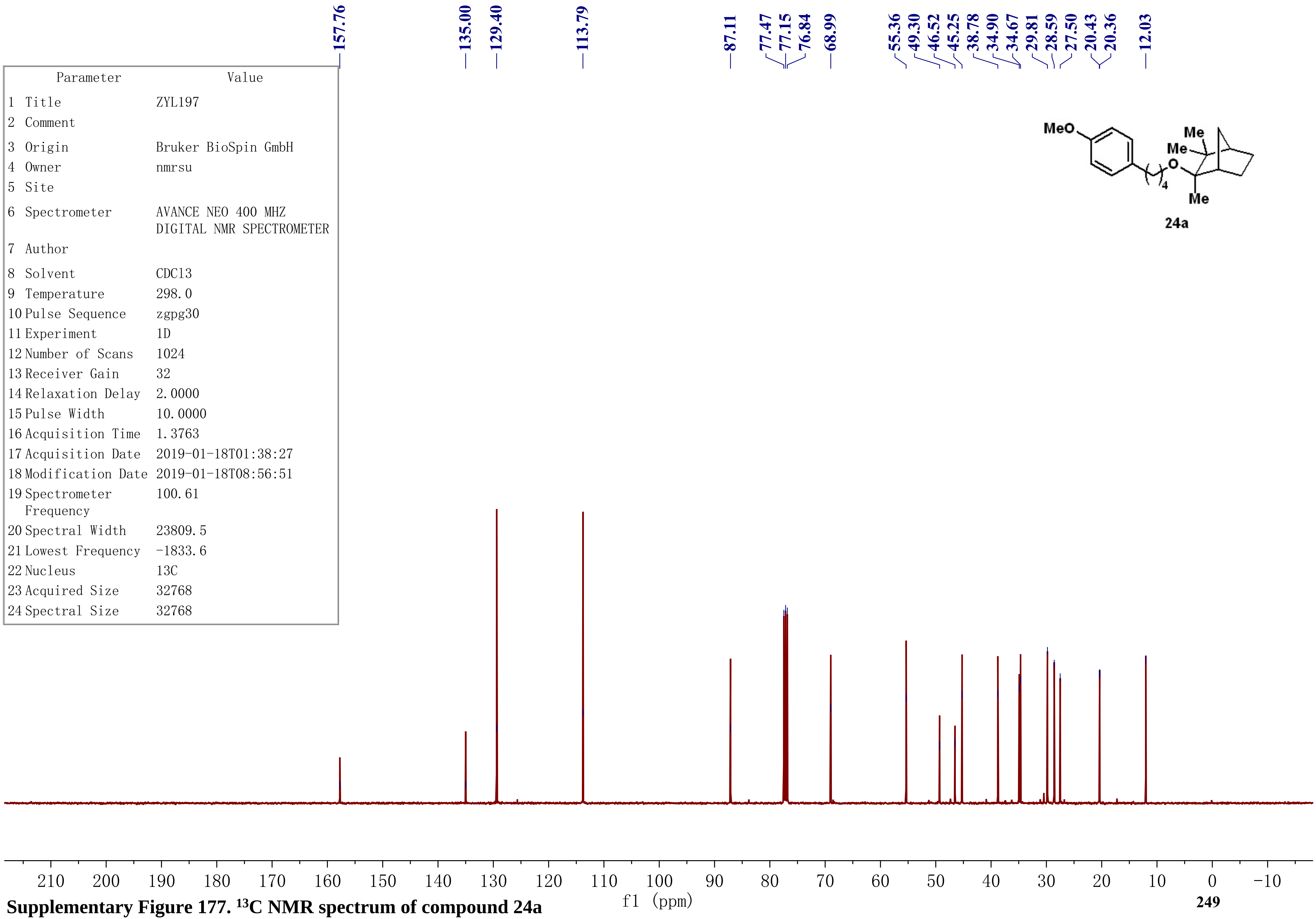

	Parameter	Value
1	Title	ZYL197
2	Comment	
3	Origin	Bruker BioSpin GmbH
4	Owner	nmrsu
5	Site	
6	Spectrometer	AVANCE NEO 400 MHZ DIGITAL NMR SPECTROMETER
7	Author	
8	Solvent	CDCl3
9	Temperature	298.0
10	Pulse Sequence	zgpg30
11	Experiment	1D
12	Number of Scans	1024
13	Receiver Gain	32
14	Relaxation Delay	2.0000
15	Pulse Width	10.0000
16	Acquisition Time	1.3763
17	Acquisition Date	2019-01-18T01:38:27
18	Modification Date	2019-01-18T08:56:51
19	Spectrometer Frequency	100.61
20	Spectral Width	23809.5
21	Lowest Frequency	-1833.6
22	Nucleus	13C
23	Acquired Size	32768
24	Spectral Size	32768

Supplementary Figure 177. ^{13}C NMR spectrum of compound 24a

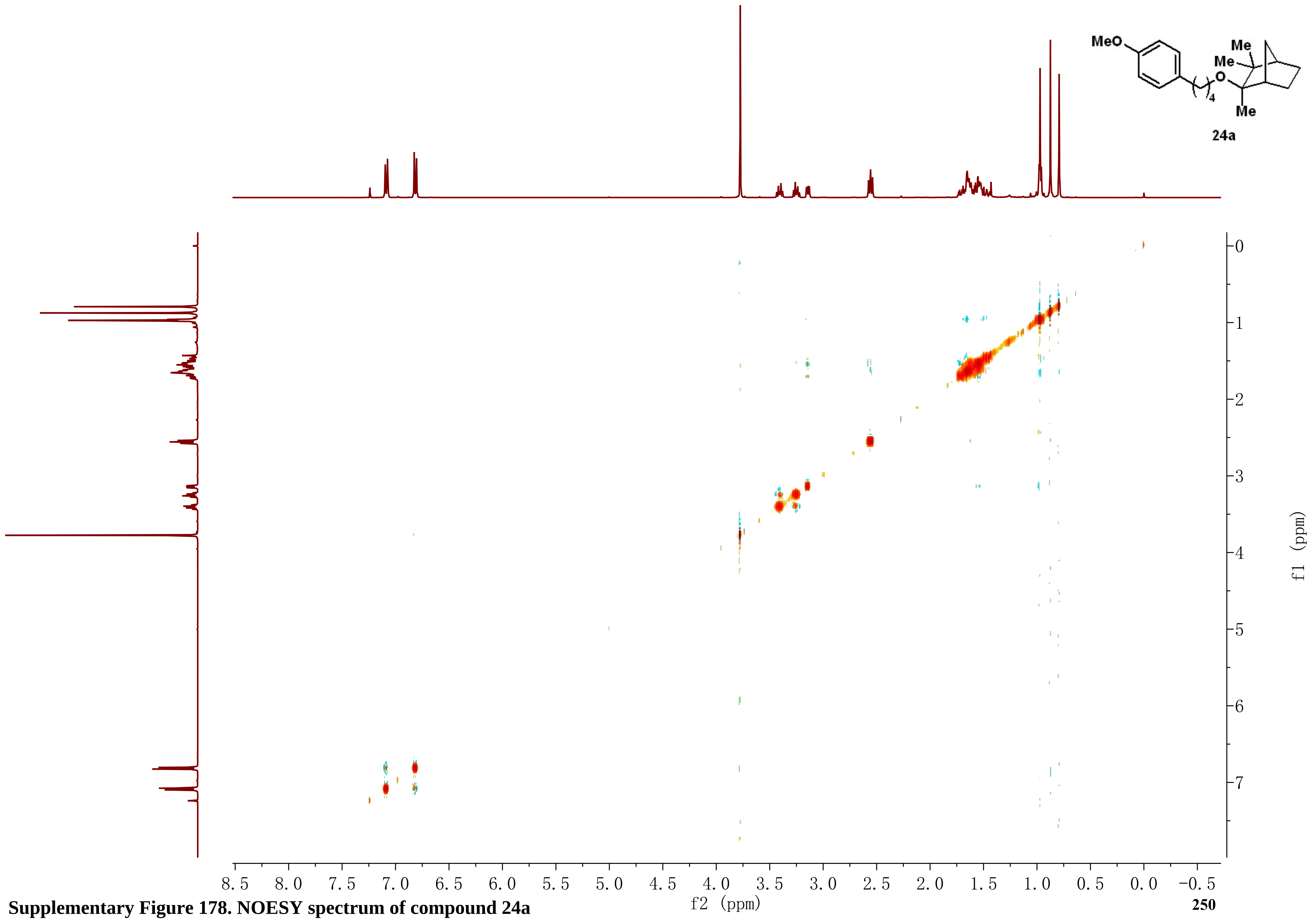

Supplementary Figure 178. NOESY spectrum of compound 24a

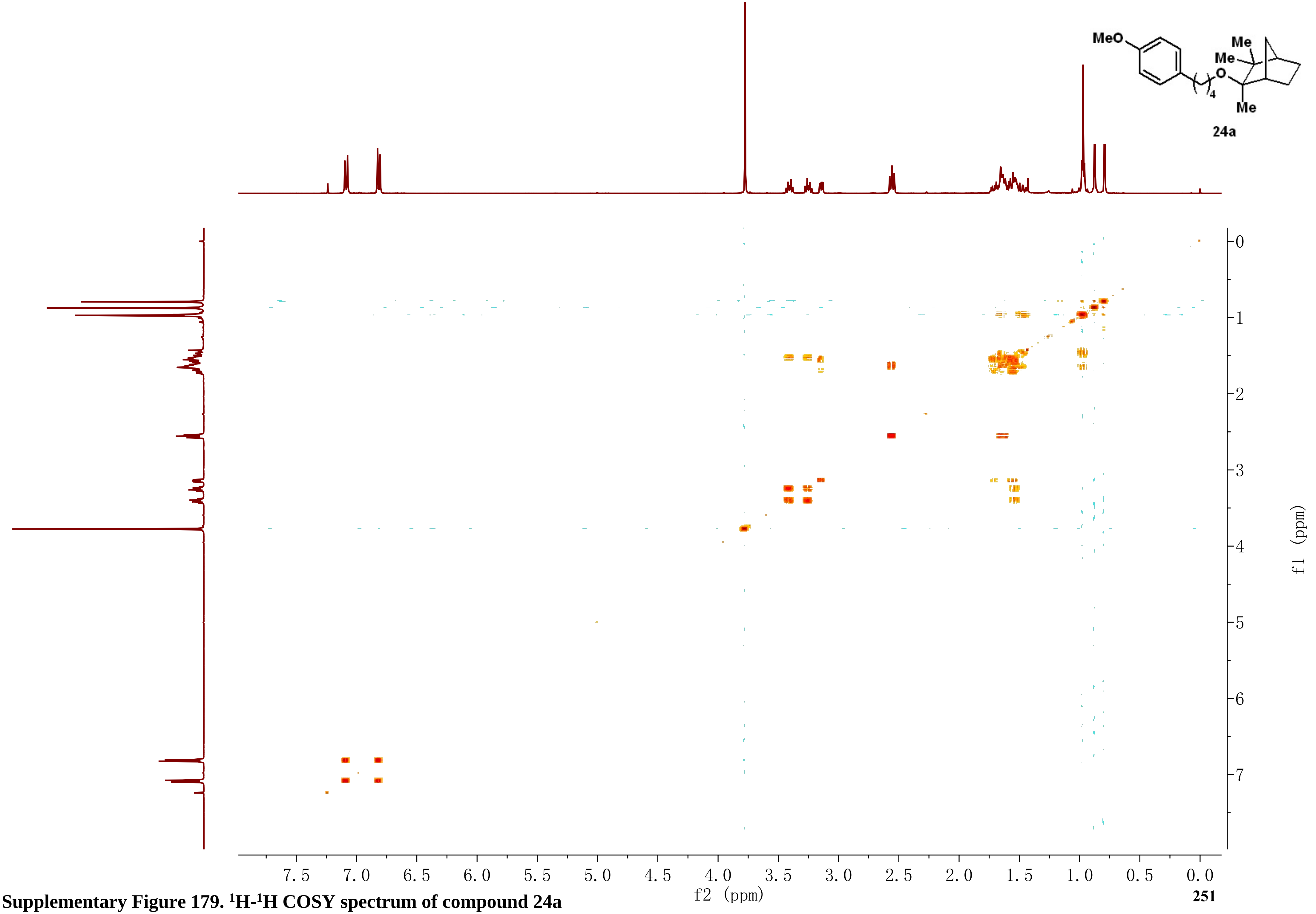

24a

Supplementary Figure 179. ^{1}H-^{1}H COSY spectrum of compound 24a

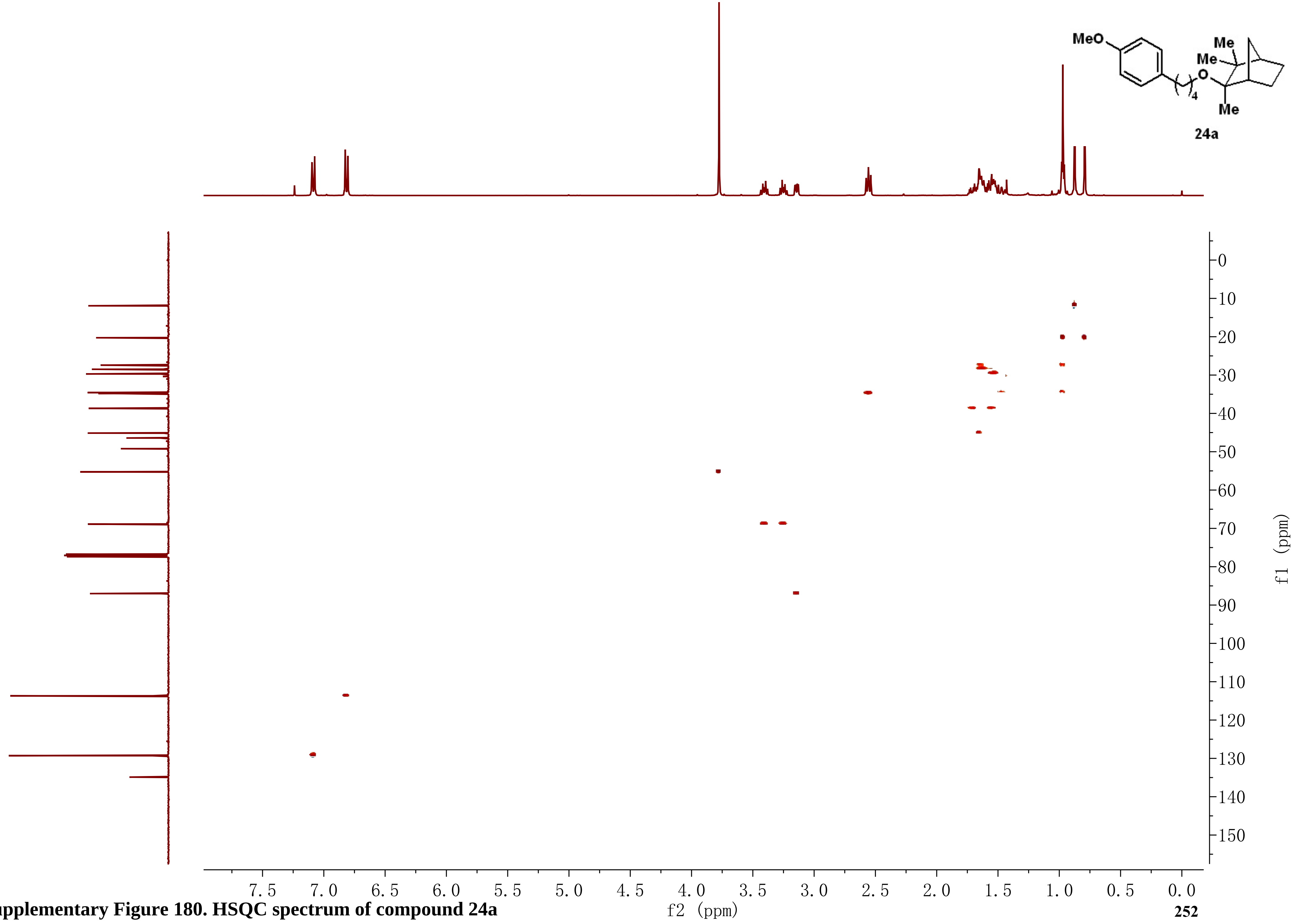

Supplementary Figure 180. HSQC spectrum of compound 24a

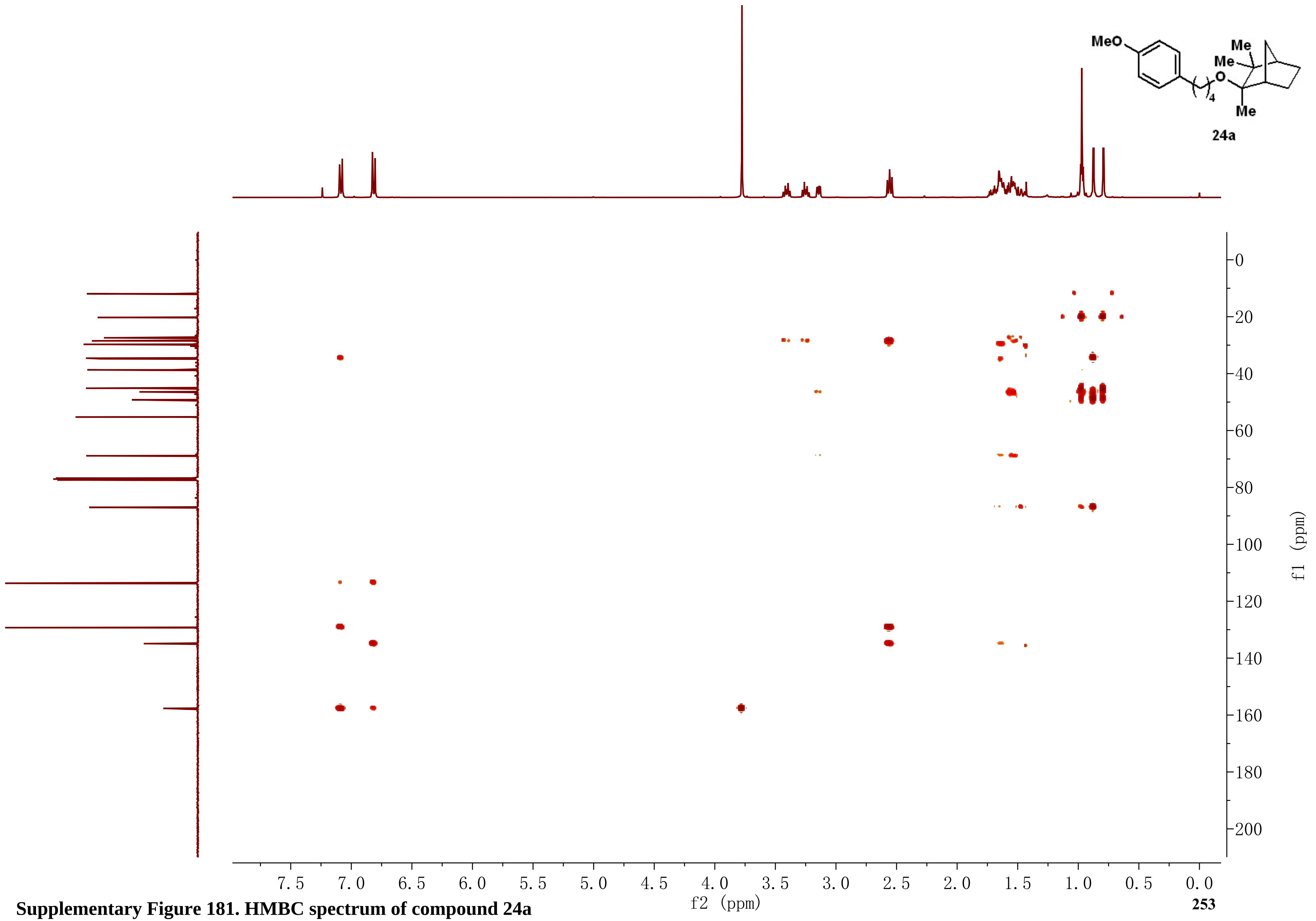

Supplementary Figure 181. HMBC spectrum of compound 24a

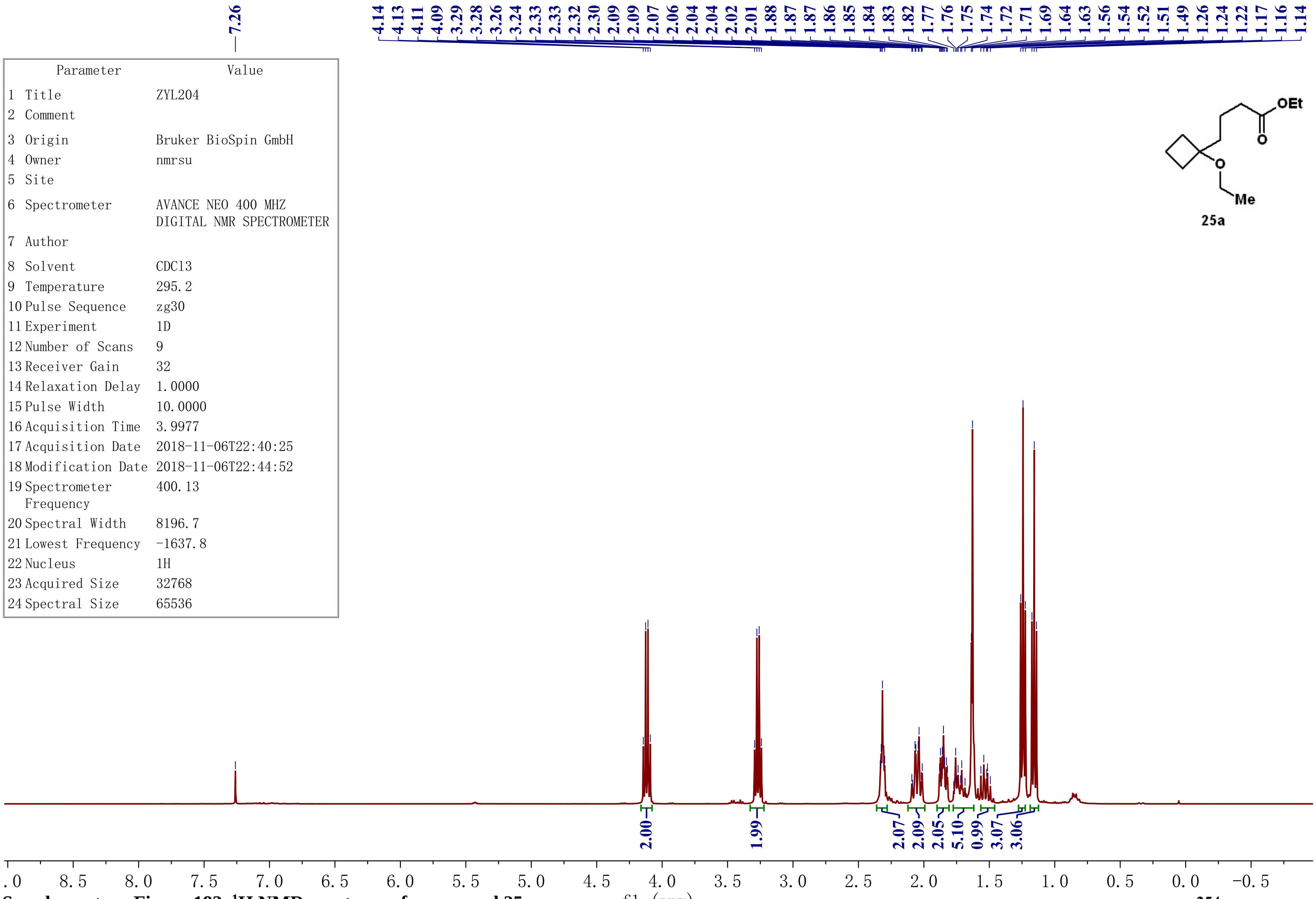

Parameter	Value
1 Title	ZYL204
2 Comment	
3 Origin	Bruker BioSpin GmbH
4 Owner	nmrsu
5 Site	
6 Spectrometer	AVANCE NEO 400 MHZ DIGITAL NMR SPECTROMETER
7 Author	
8 Solvent	CDCl3
9 Temperature	295.2
10 Pulse Sequence	zg30
11 Experiment	1D
12 Number of Scans	9
13 Receiver Gain	32
14 Relaxation Delay	1.0000
15 Pulse Width	10.0000
16 Acquisition Time	3.9977
17 Acquisition Date	2018-11-06T22:40:25
18 Modification Date	2018-11-06T22:44:52
19 Spectrometer Frequency	400.13
20 Spectral Width	8196.7
21 Lowest Frequency	-1637.8
22 Nucleus	1H
23 Acquired Size	32768
24 Spectral Size	65536

Supplementary Figure 182. ^{1}H NMR spectrum of compound 25a

Parameter	Value
1 Title	ZYL204
2 Comment	
3 Origin	Bruker BioSpin GmbH
4 Owner	nmrsu
5 Site	
6 Spectrometer	AVANCE NEO 400 MHZ DIGITAL NMR SPECTROMETER
7 Author	
8 Solvent	CDCl3
9 Temperature	295.5
10 Pulse Sequence	zgpg30
11 Experiment	1D
12 Number of Scans	8
13 Receiver Gain	35
14 Relaxation Delay	2.0000
15 Pulse Width	10.0000
16 Acquisition Time	1.3763
17 Acquisition Date	2018-11-06T22:42:05
18 Modification Date	2018-11-06T22:44:52
19 Spectrometer Frequency	100.61
20 Spectral Width	23809.5
21 Lowest Frequency	-1832.7
22 Nucleus	13C
23 Acquired Size	32768
24 Spectral Size	32768

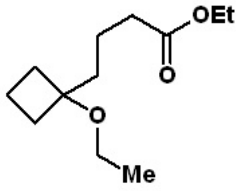

25a

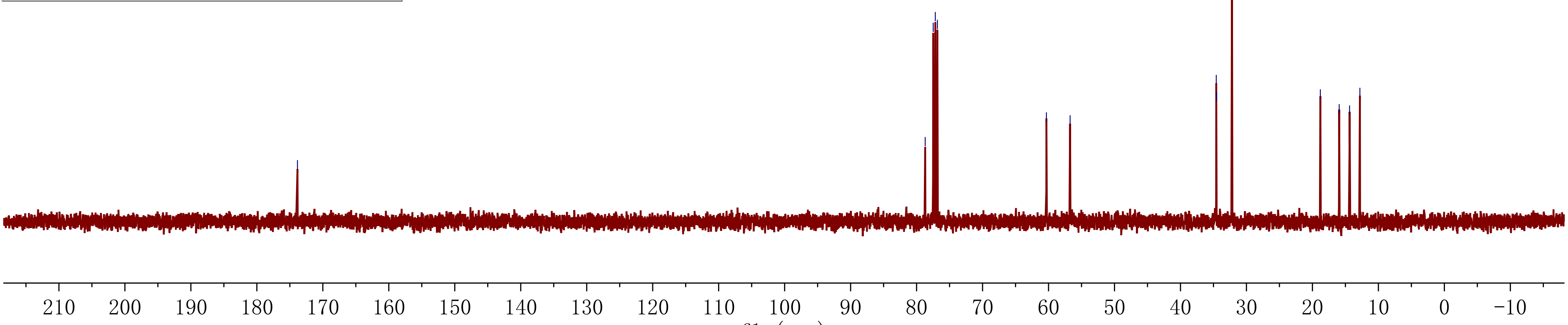

Supplementary Figure 183. ^{13}**C NMR spectrum of compound 25a**

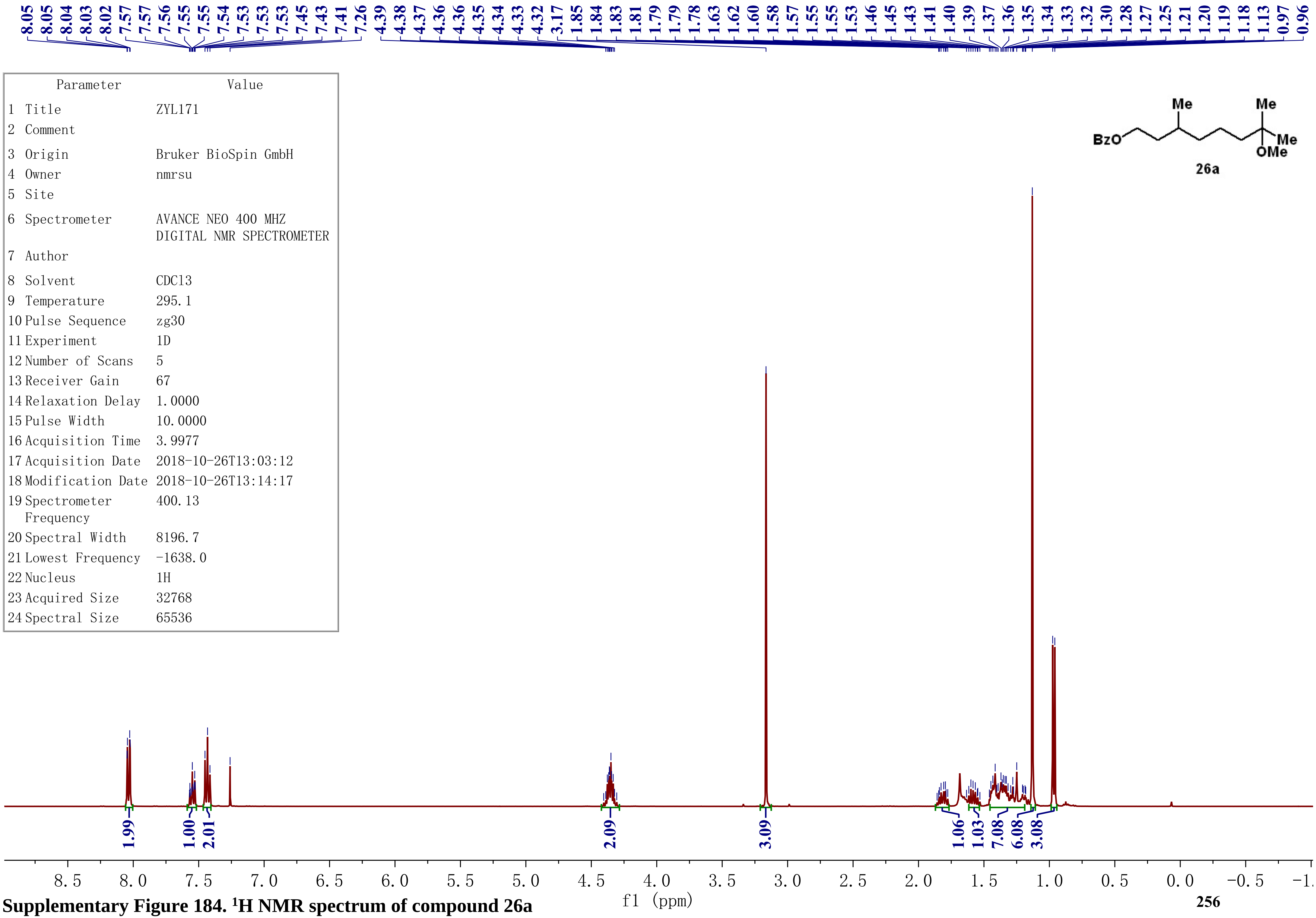

Supplementary Figure 184. ^{1}H NMR spectrum of compound 26a

	Parameter	Value
1	Title	ZYL171C
2	Comment	
3	Origin	Bruker BioSpin GmbH
4	Owner	nmrsu
5	Site	
6	Spectrometer	AVANCE NEO 400 MHZ DIGITAL NMR SPECTROMETER
7	Author	
8	Solvent	CDCl3
9	Temperature	295.7
10	Pulse Sequence	zgpg30
11	Experiment	1D
12	Number of Scans	20
13	Receiver Gain	32
14	Relaxation Delay	2.0000
15	Pulse Width	10.0000
16	Acquisition Time	1.3763
17	Acquisition Date	2018-10-26T21:33:38
18	Modification Date	2018-10-27T22:23:17
19	Spectrometer Frequency	100.61
20	Spectral Width	23809.5
21	Lowest Frequency	-1832.3
22	Nucleus	13C
23	Acquired Size	32768
24	Spectral Size	32768

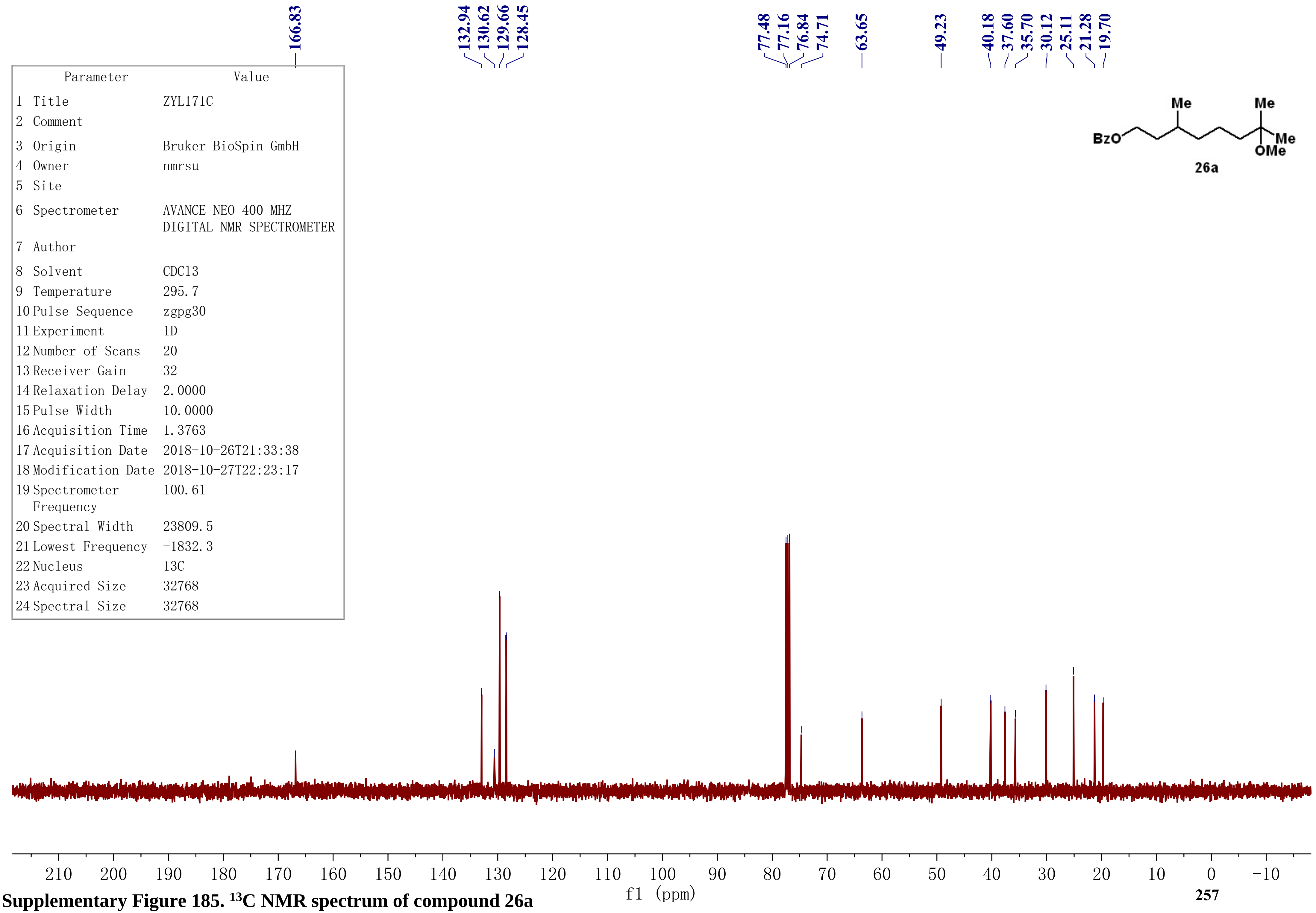

Supplementary Figure 185. ^{13}C **NMR spectrum of compound 26a**

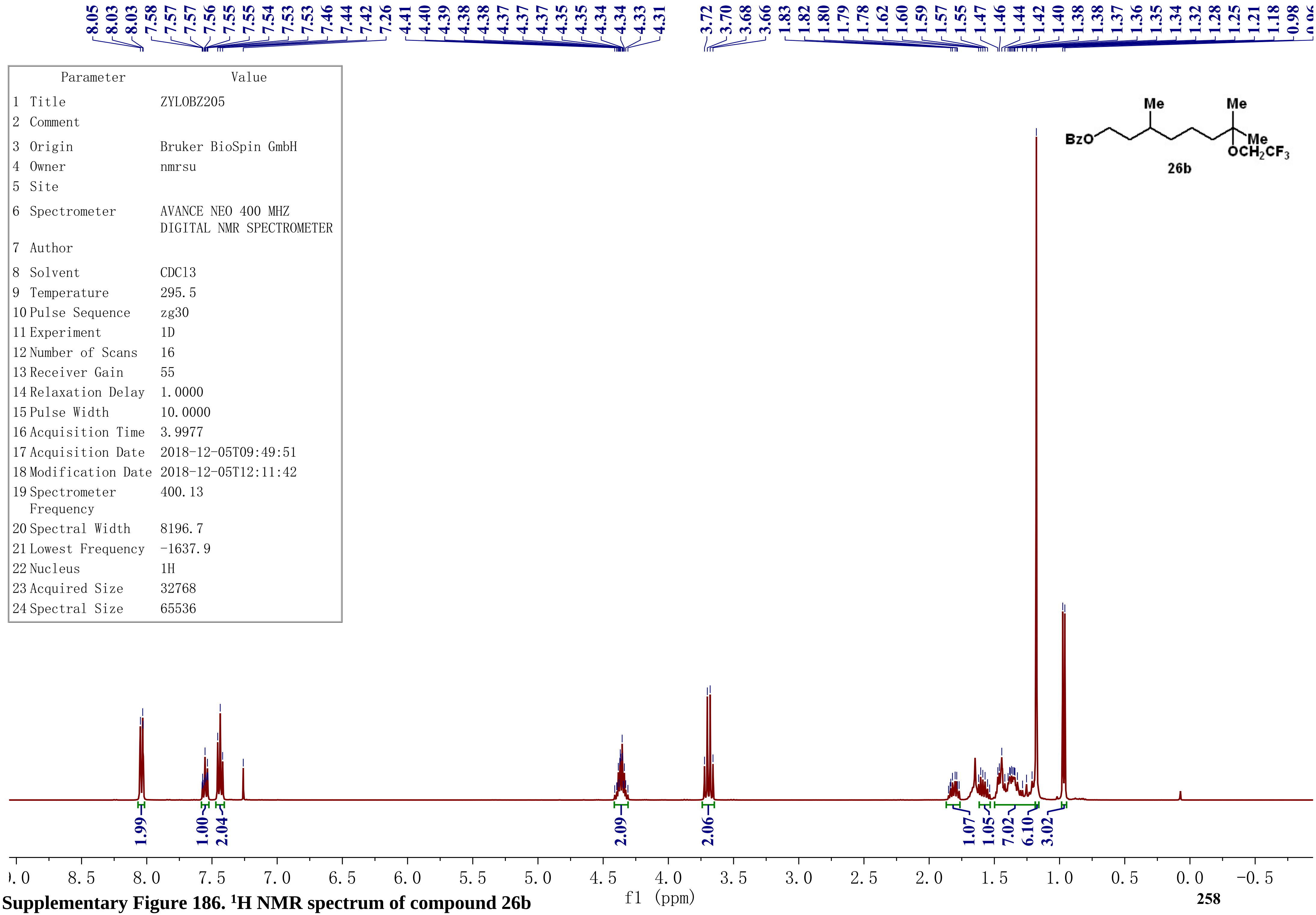

	Parameter	Value
1	Title	ZYLOBZ205
2	Comment	
3	Origin	Bruker BioSpin GmbH
4	Owner	nmrsu
5	Site	
6	Spectrometer	AVANCE NEO 400 MHZ DIGITAL NMR SPECTROMETER
7	Author	
8	Solvent	CDCl3
9	Temperature	295.5
10	Pulse Sequence	zg30
11	Experiment	1D
12	Number of Scans	16
13	Receiver Gain	55
14	Relaxation Delay	1.0000
15	Pulse Width	10.0000
16	Acquisition Time	3.9977
17	Acquisition Date	2018-12-05T09:49:51
18	Modification Date	2018-12-05T12:11:42
19	Spectrometer Frequency	400.13
20	Spectral Width	8196.7
21	Lowest Frequency	-1637.9
22	Nucleus	1H
23	Acquired Size	32768
24	Spectral Size	65536

Supplementary Figure 186. ^{1}H NMR spectrum of compound 26b

Parameter	Value
1 Title	ZYLOBZ205
2 Comment	
3 Origin	Bruker BioSpin GmbH
4 Owner	nmrsu
5 Site	
6 Spectrometer	AVANCE NEO 400 MHZ DIGITAL NMR SPECTROMETER
7 Author	
8 Solvent	CDCl3
9 Temperature	296.0
10 Pulse Sequence	zgpg30
11 Experiment	1D
12 Number of Scans	286
13 Receiver Gain	29
14 Relaxation Delay	2.0000
15 Pulse Width	10.0000
16 Acquisition Time	1.3763
17 Acquisition Date	2018-12-05T10:07:29
18 Modification Date	2018-12-05T12:11:42
19 Spectrometer Frequency	100.61
20 Spectral Width	23809.5
21 Lowest Frequency	-1831.7
22 Nucleus	13C
23 Acquired Size	32768
24 Spectral Size	32768

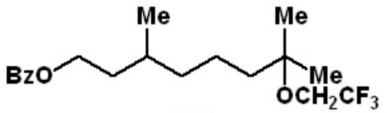

26b

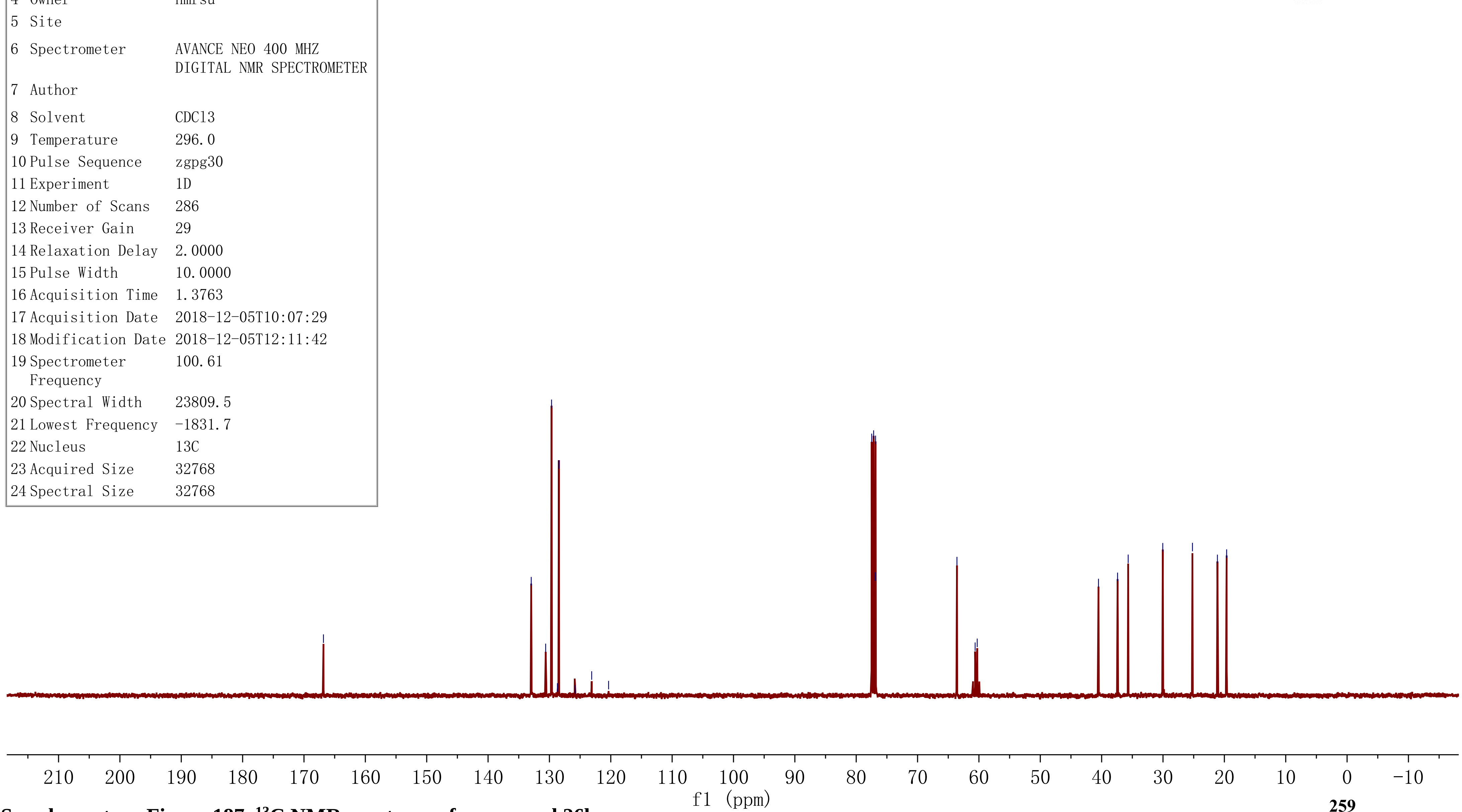

Supplementary Figure 187. ^{13}C NMR spectrum of compound 26b

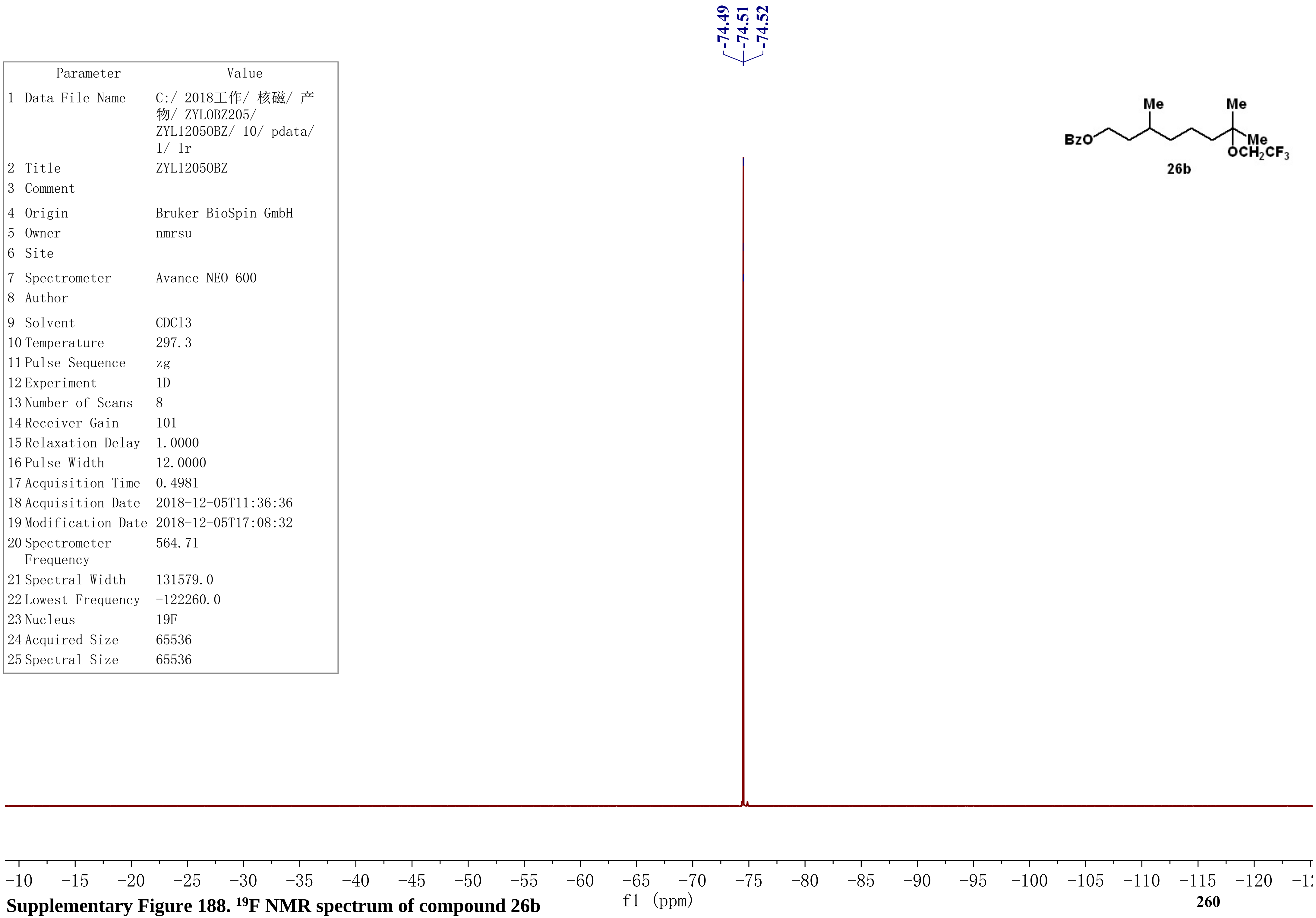

26b

	Parameter	Value
1	Data File Name	C:/ 2018工作/ 核磁/ 产物/ ZYLOBZ205/ ZYL12050BZ/ 10/ pdata/ 1/ 1r
2	Title	ZYL12050BZ
3	Comment	
4	Origin	Bruker BioSpin GmbH
5	Owner	nmrsu
6	Site	
7	Spectrometer	Avance NEO 600
8	Author	
9	Solvent	CDCl3
10	Temperature	297.3
11	Pulse Sequence	zg
12	Experiment	1D
13	Number of Scans	8
14	Receiver Gain	101
15	Relaxation Delay	1.0000
16	Pulse Width	12.0000
17	Acquisition Time	0.4981
18	Acquisition Date	2018-12-05T11:36:36
19	Modification Date	2018-12-05T17:08:32
20	Spectrometer Frequency	564.71
21	Spectral Width	131579.0
22	Lowest Frequency	-122260.0
23	Nucleus	19F
24	Acquired Size	65536
25	Spectral Size	65536

Supplementary Figure 188. ^{19}F NMR spectrum of compound 26b

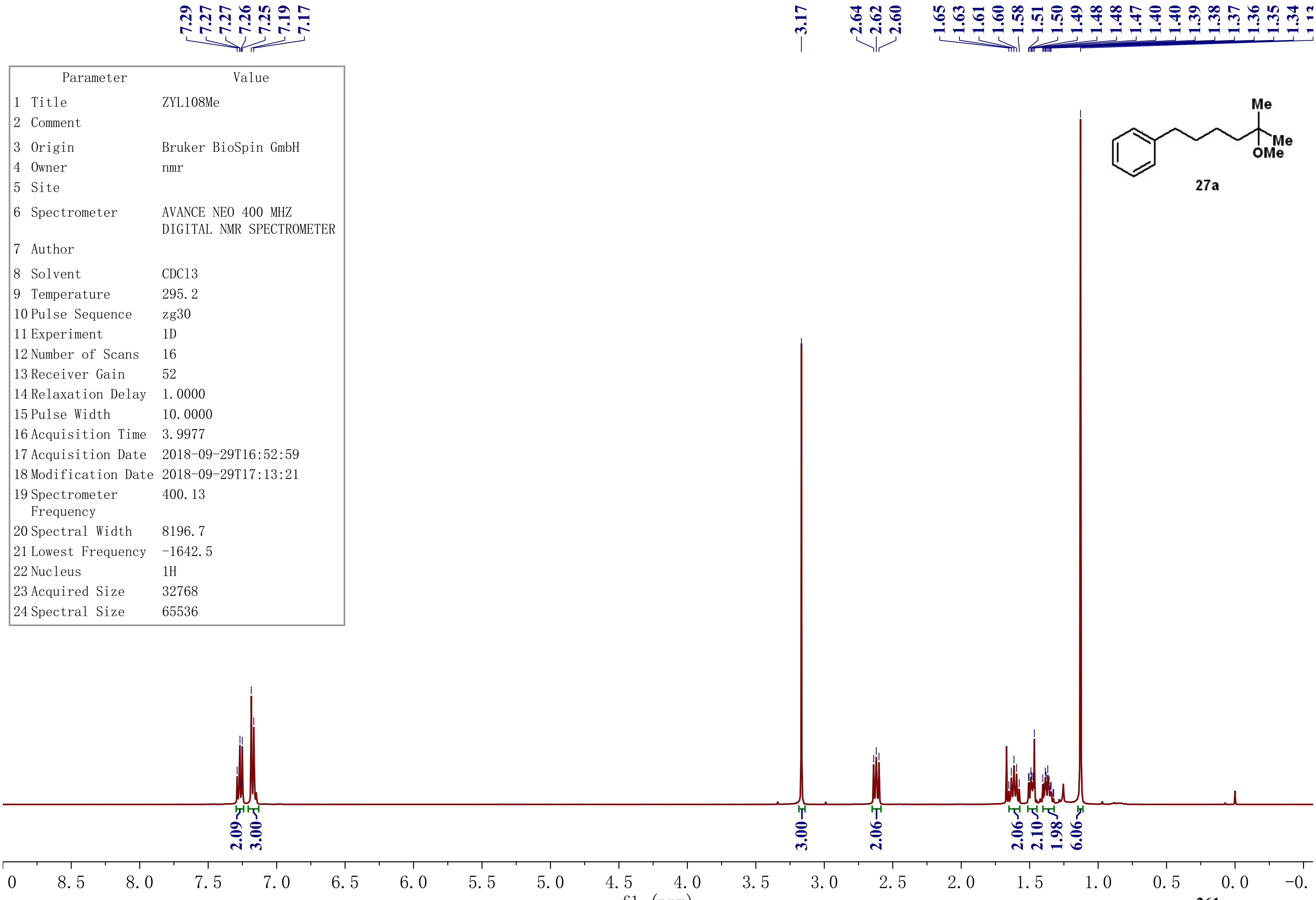

	Parameter	Value
1	Title	ZYL108Me
2	Comment	
3	Origin	Bruker BioSpin GmbH
4	Owner	nmr
5	Site	
6	Spectrometer	AVANCE NEO 400 MHZ DIGITAL NMR SPECTROMETER
7	Author	
8	Solvent	CDCl3
9	Temperature	295.2
10	Pulse Sequence	zg30
11	Experiment	1D
12	Number of Scans	16
13	Receiver Gain	52
14	Relaxation Delay	1.0000
15	Pulse Width	10.0000
16	Acquisition Time	3.9977
17	Acquisition Date	2018-09-29T16:52:59
18	Modification Date	2018-09-29T17:13:21
19	Spectrometer Frequency	400.13
20	Spectral Width	8196.7
21	Lowest Frequency	-1642.5
22	Nucleus	1H
23	Acquired Size	32768
24	Spectral Size	65536

Supplementary Figure 189. ^{1}H NMR spectrum of compound 27a

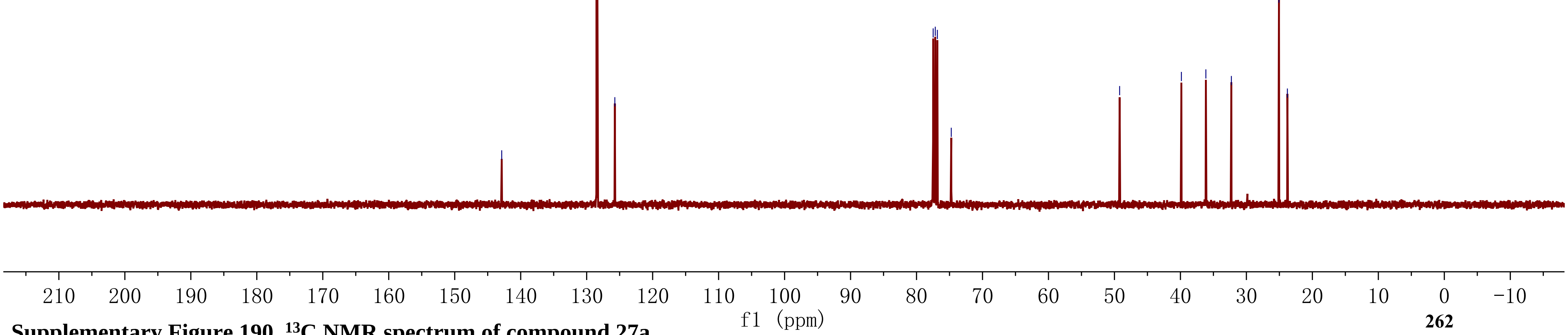

Parameter	Value
1 Title	ZYL108Me
2 Comment	
3 Origin	Bruker BioSpin GmbH
4 Owner	nmr
5 Site	
6 Spectrometer	AVANCE NEO 400 MHZ DIGITAL NMR SPECTROMETER
7 Author	
8 Solvent	CDCl3
9 Temperature	295.9
10 Pulse Sequence	zgpg30
11 Experiment	1D
12 Number of Scans	50
13 Receiver Gain	35
14 Relaxation Delay	2.0000
15 Pulse Width	10.0000
16 Acquisition Time	1.3763
17 Acquisition Date	2018-09-29T18:38:40
18 Modification Date	2018-09-29T19:50:10
19 Spectrometer Frequency	100.61
20 Spectral Width	23809.5
21 Lowest Frequency	-1833.5
22 Nucleus	13C
23 Acquired Size	32768
24 Spectral Size	32768

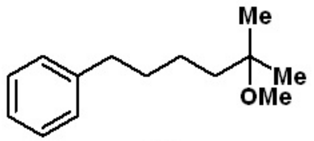

27a

Supplementary Figure 190. ^{13}C NMR spectrum of compound 27a

Parameter	Value
1 Title	ZYL108ET
2 Comment	
3 Origin	Bruker BioSpin GmbH
4 Owner	nmr
5 Site	
6 Spectrometer	AVANCE NEO 400 MHZ DIGITAL NMR SPECTROMETER
7 Author	
8 Solvent	CDCl3
9 Temperature	296.2
10 Pulse Sequence	zg30
11 Experiment	1D
12 Number of Scans	16
13 Receiver Gain	71
14 Relaxation Delay	1.0000
15 Pulse Width	10.0000
16 Acquisition Time	3.9977
17 Acquisition Date	2018-09-30T15:22:47
18 Modification Date	2018-09-30T15:41:20
19 Spectrometer Frequency	400.13
20 Spectral Width	8196.7
21 Lowest Frequency	-1640.6
22 Nucleus	1H
23 Acquired Size	32768
24 Spectral Size	65536

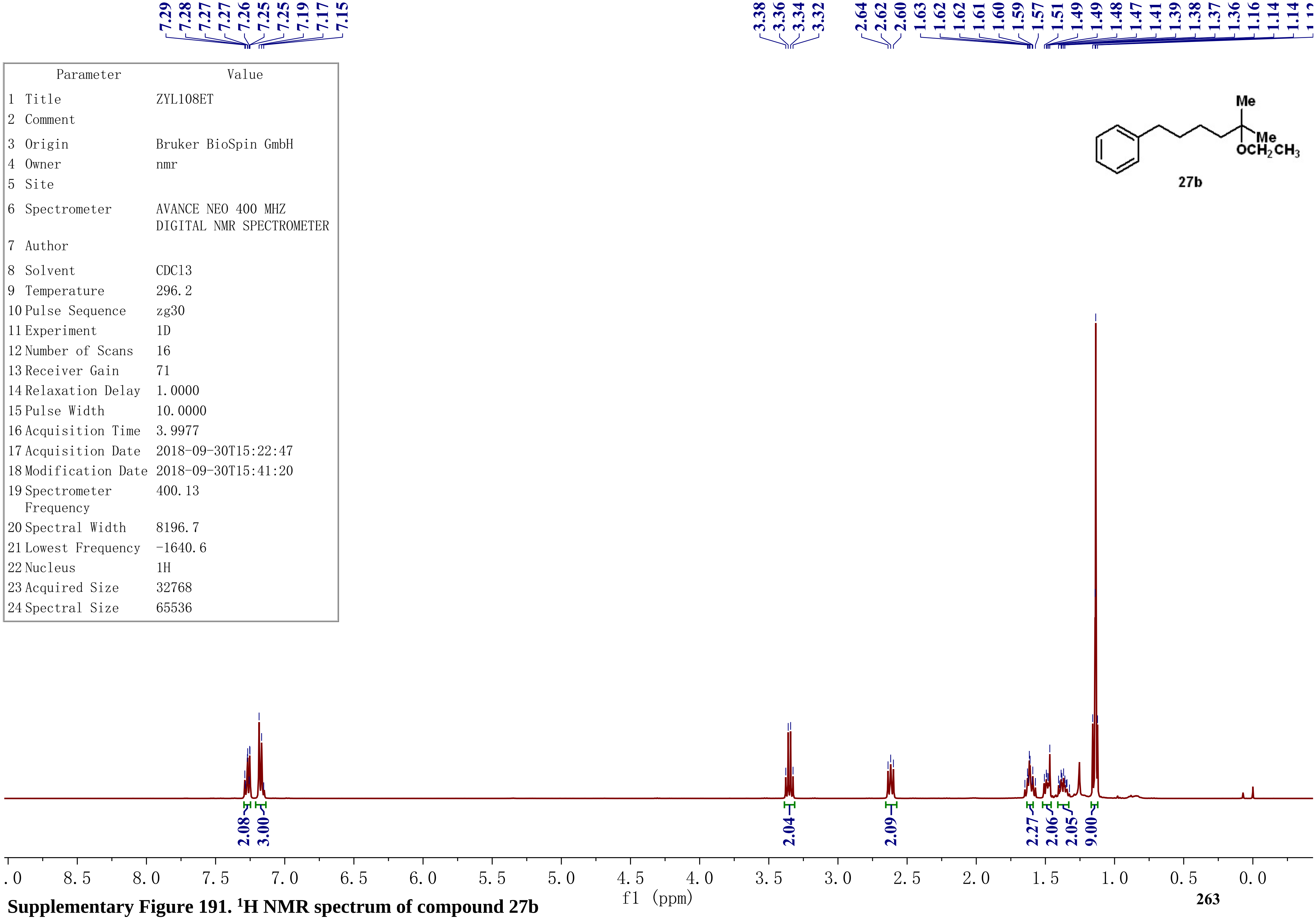

Supplementary Figure 191. ^{1}H NMR spectrum of compound 27b

Parameter		Value
1	Title	ZYL108ET
2	Comment	
3	Origin	Bruker BioSpin GmbH
4	Owner	nmr
5	Site	
6	Spectrometer	AVANCE NEO 400 MHZ DIGITAL NMR SPECTROMETER
7	Author	
8	Solvent	CDCl3
9	Temperature	295.9
10	Pulse Sequence	zgpg30
11	Experiment	1D
12	Number of Scans	14
13	Receiver Gain	32
14	Relaxation Delay	2.0000
15	Pulse Width	10.0000
16	Acquisition Time	1.3763
17	Acquisition Date	2018-09-30T15:37:52
18	Modification Date	2018-09-30T15:41:21
19	Spectrometer Frequency	100.61
20	Spectral Width	23809.5
21	Lowest Frequency	-1832.2
22	Nucleus	13C
23	Acquired Size	32768
24	Spectral Size	32768

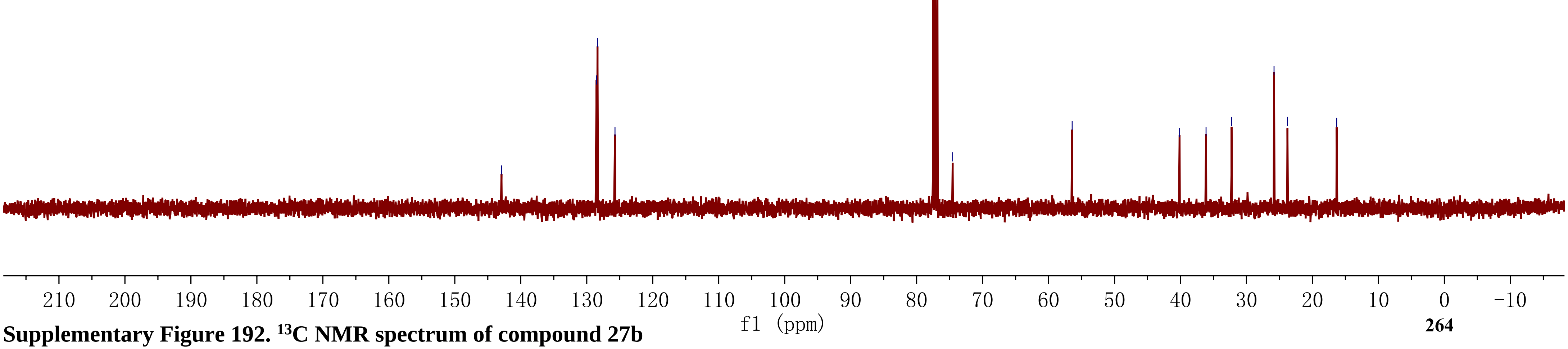

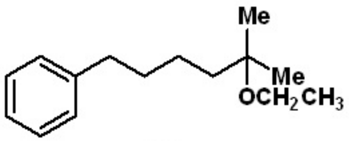

27b

Supplementary Figure 192. ^{13}C NMR spectrum of compound 27b

Parameter	Value
1 Title	zyl1205cf3
2 Comment	
3 Origin	Bruker BioSpin GmbH
4 Owner	nmrsu
5 Site	
6 Spectrometer	Avance NEO 600
7 Author	
8 Solvent	CDCl3
9 Temperature	296.8
10 Pulse Sequence	zg30
11 Experiment	1D
12 Number of Scans	16
13 Receiver Gain	32
14 Relaxation Delay	1.0000
15 Pulse Width	10.0000
16 Acquisition Time	2.7525
17 Acquisition Date	2018-12-05T11:08:08
18 Modification Date	2018-12-05T17:08:33
19 Spectrometer Frequency	600.15
20 Spectral Width	11904.8
21 Lowest Frequency	-2261.5
22 Nucleus	1H
23 Acquired Size	32768
24 Spectral Size	65536

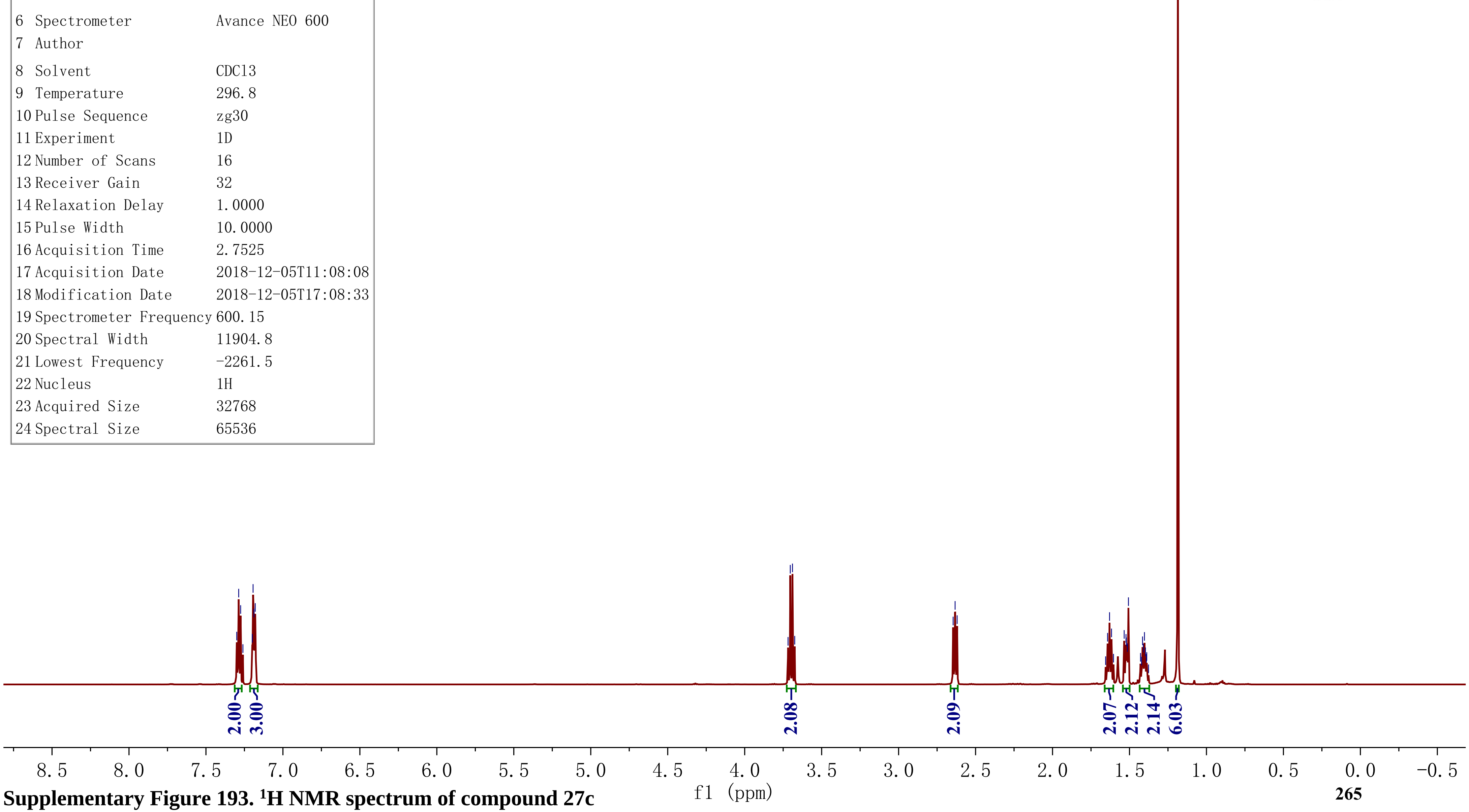

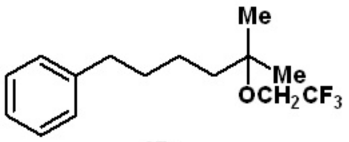

27c

Supplementary Figure 193. ^{1}H NMR spectrum of compound 27c

Parameter	Value
1 Title	zyl1205cf3
2 Comment	
3 Origin	Bruker BioSpin GmbH
4 Owner	nmrsu
5 Site	
6 Spectrometer	Avance NEO 600
7 Author	
8 Solvent	CDCl3
9 Temperature	297.9
10 Pulse Sequence	zgpg30
11 Experiment	1D
12 Number of Scans	199
13 Receiver Gain	101
14 Relaxation Delay	2.0000
15 Pulse Width	12.0000
16 Acquisition Time	0.9175
17 Acquisition Date	2018-12-05T11:22:54
18 Modification Date	2018-12-05T17:08:34
19 Spectrometer Frequency	150.91
20 Spectral Width	35714.3
21 Lowest Frequency	-2747.8
22 Nucleus	13C
23 Acquired Size	32768
24 Spectral Size	32768

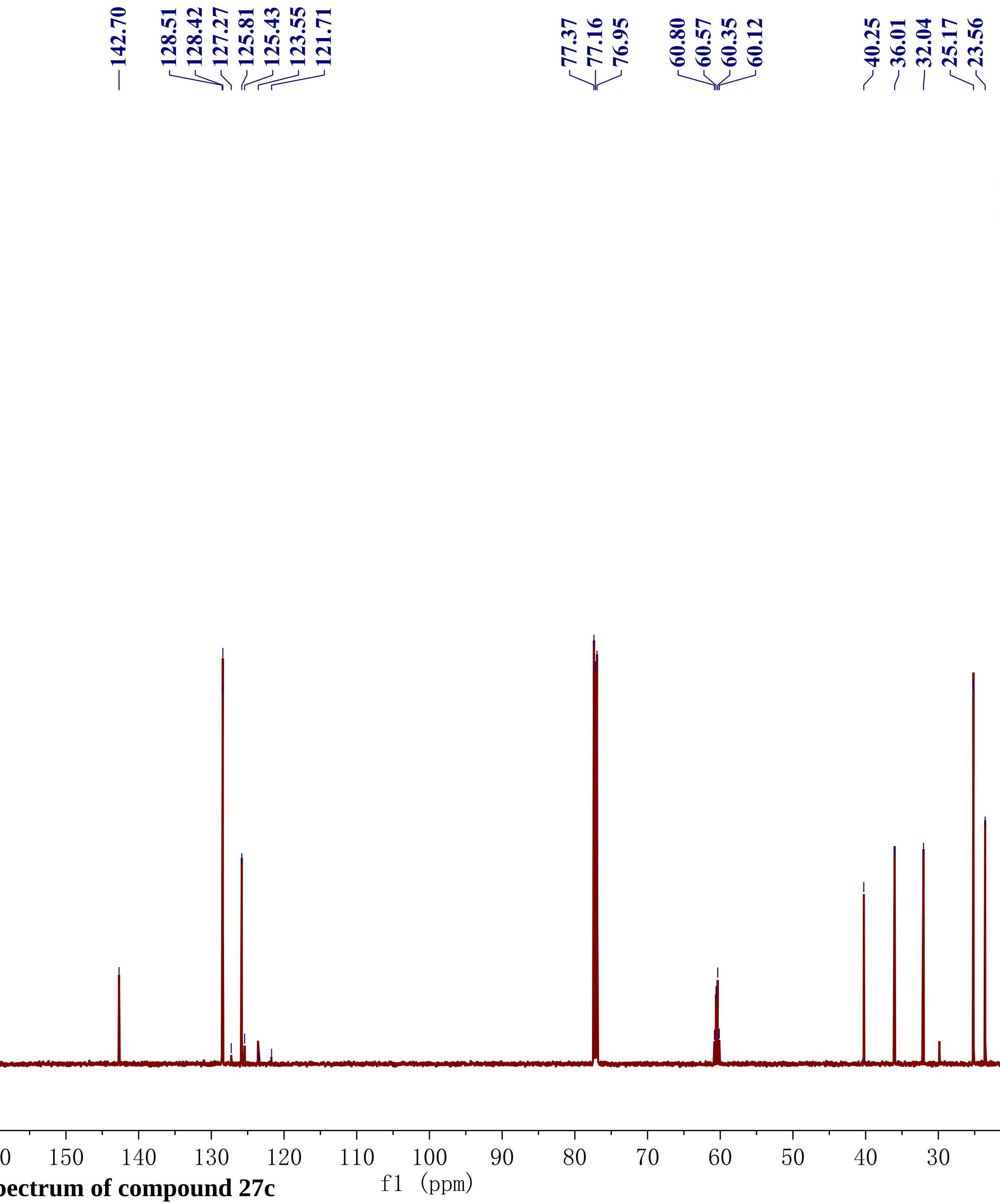

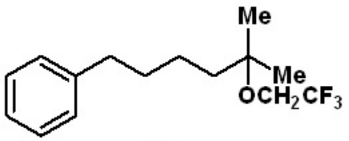

27c

Supplementary Figure 194. ^{13}C NMR spectrum of compound 27c

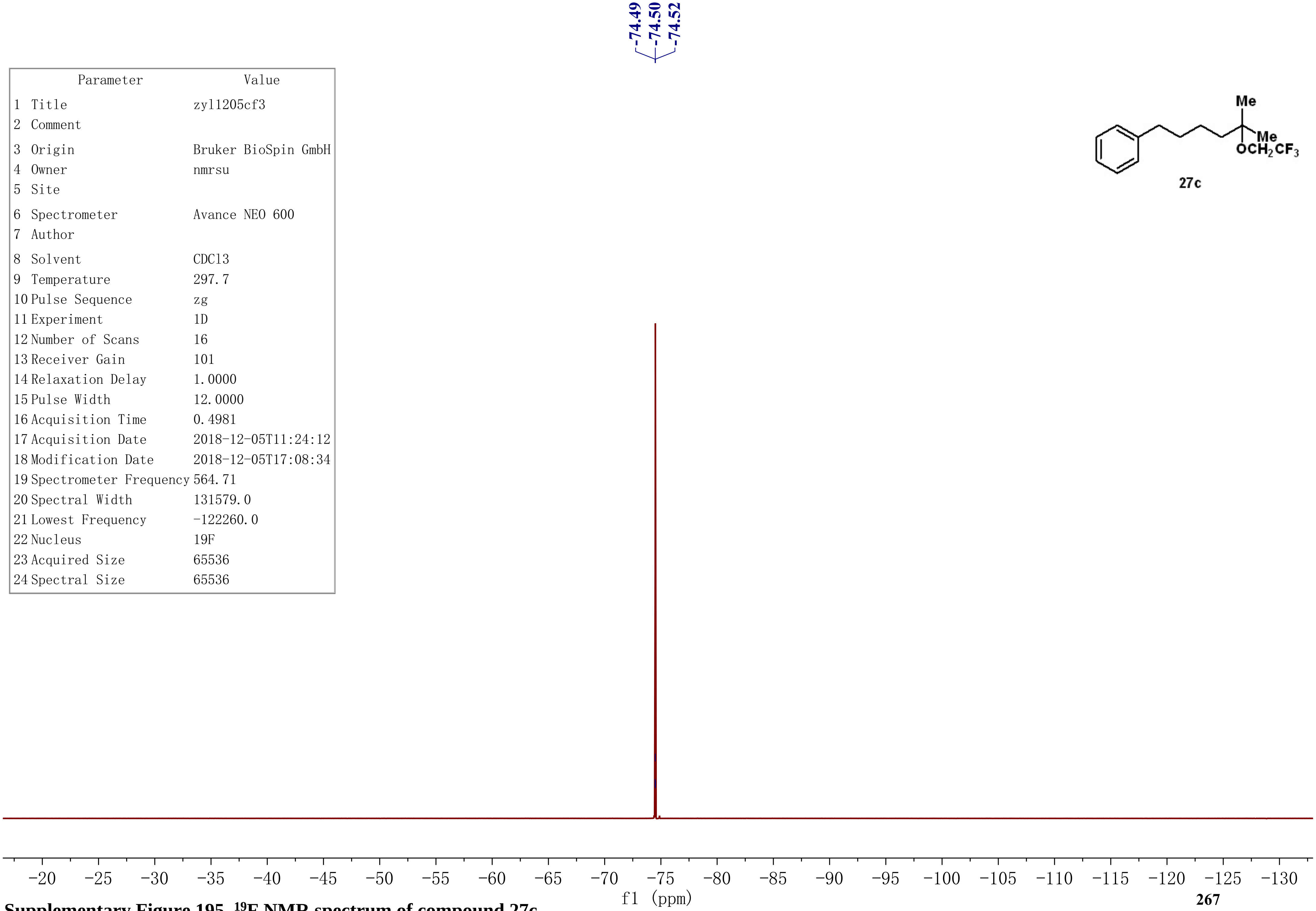

Parameter		Value
1	Title	zyl1205cf3
2	Comment	
3	Origin	Bruker BioSpin GmbH
4	Owner	nmrsu
5	Site	
6	Spectrometer	Avance NEO 600
7	Author	
8	Solvent	CDCl3
9	Temperature	297.7
10	Pulse Sequence	zg
11	Experiment	1D
12	Number of Scans	16
13	Receiver Gain	101
14	Relaxation Delay	1.0000
15	Pulse Width	12.0000
16	Acquisition Time	0.4981
17	Acquisition Date	2018-12-05T11:24:12
18	Modification Date	2018-12-05T17:08:34
19	Spectrometer Frequency	564.71
20	Spectral Width	131579.0
21	Lowest Frequency	-122260.0
22	Nucleus	19F
23	Acquired Size	65536
24	Spectral Size	65536

27c

Supplementary Figure 195. ^{19}F NMR spectrum of compound 27c

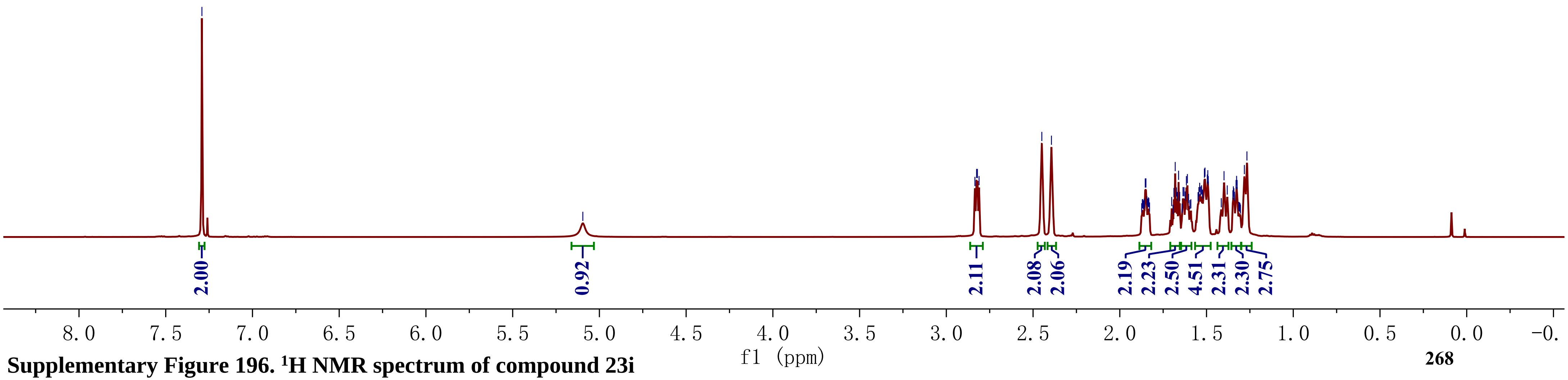

	Parameter	Value
1	Title	ZYLF-29-2
2	Comment	
3	Origin	Bruker BioSpin GmbH
4	Owner	nmrsu
5	Site	
6	Spectrometer	Avance NEO 600
7	Author	
8	Solvent	CDCl3
9	Temperature	298.0
10	Pulse Sequence	zg30
11	Experiment	1D
12	Number of Scans	16
13	Receiver Gain	48
14	Relaxation Delay	1.0000
15	Pulse Width	10.0000
16	Acquisition Time	2.7525
17	Acquisition Date	2019-10-10T09:26:22
18	Modification Date	2019-10-10T10:03:14
19	Spectrometer Frequency	600.15
20	Spectral Width	11904.8
21	Lowest Frequency	-2261.3
22	Nucleus	1H
23	Acquired Size	32768
24	Spectral Size	65536

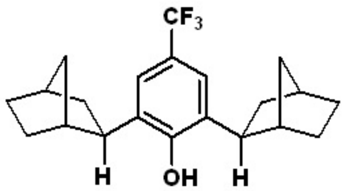

23i

Supplementary Figure 196. ^{1}H NMR spectrum of compound 23i

Parameter	Value
1 Title	ZYLF-29-2
2 Comment	
3 Origin	Bruker BioSpin GmbH
4 Owner	nmrsu
5 Site	
6 Spectrometer	Avance NEO 600
7 Author	
8 Solvent	CDCl3
9 Temperature	299.5
10 Pulse Sequence	zgpg30
11 Experiment	1D
12 Number of Scans	200
13 Receiver Gain	101
14 Relaxation Delay	2.0000
15 Pulse Width	12.0000
16 Acquisition Time	0.9175
17 Acquisition Date	2019-10-10T09:37:55
18 Modification Date	2019-10-21T09:12:55
19 Spectrometer Frequency	150.91
20 Spectral Width	35714.3
21 Lowest Frequency	-2746.6
22 Nucleus	13C
23 Acquired Size	32768
24 Spectral Size	32768

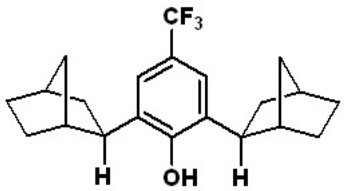

23i

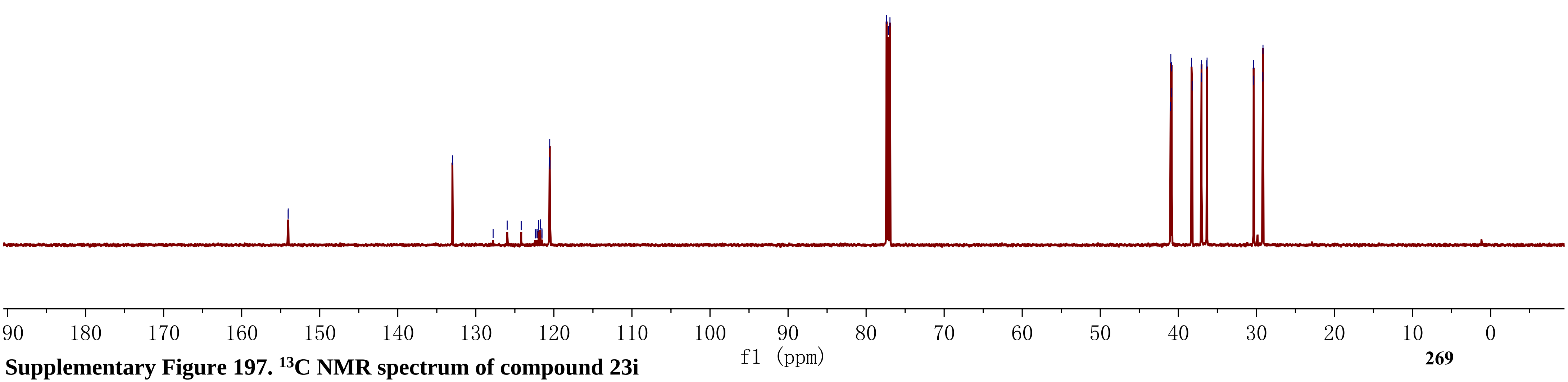

Supplementary Figure 197. ^{13}C NMR spectrum of compound 23i

Parameter	Value
1 Title	ZYLF-29-2
2 Comment	
3 Origin	Bruker BioSpin GmbH
4 Owner	nmrsu
5 Site	
6 Spectrometer	Avance NEO 600
7 Author	
8 Solvent	CDCl3
9 Temperature	298.8
10 Pulse Sequence	zgig
11 Experiment	1D
12 Number of Scans	16
13 Receiver Gain	101
14 Relaxation Delay	1.0000
15 Pulse Width	12.0000
16 Acquisition Time	0.4981
17 Acquisition Date	2019-10-20T13:57:28
18 Modification Date	2019-10-21T09:12:55
19 Spectrometer Frequency	564.71
20 Spectral Width	131579.0
21 Lowest Frequency	-122260.0
22 Nucleus	19F
23 Acquired Size	65536
24 Spectral Size	65536

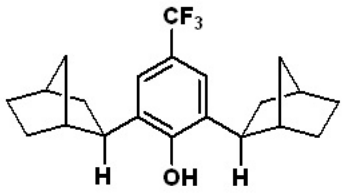

23i

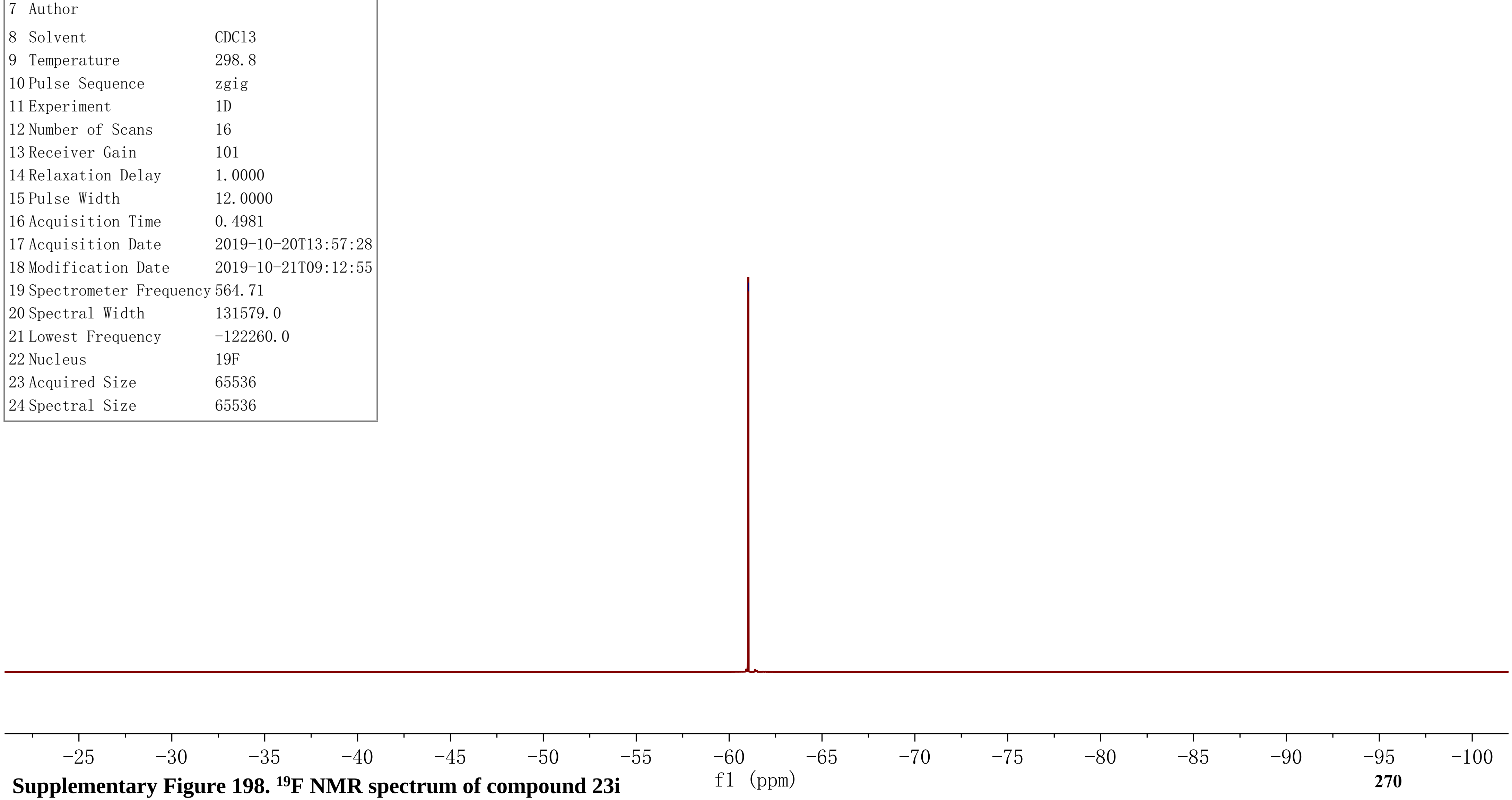

Supplementary Figure 198. ^{19}F NMR spectrum of compound 23i

Parameter	Value
1 Title	ZYLF-29-1
2 Comment	
3 Origin	Bruker BioSpin GmbH
4 Owner	nmrsu
5 Site	
6 Spectrometer	Avance NEO 600
7 Author	
8 Solvent	CDCl3
9 Temperature	298.2
10 Pulse Sequence	zg30
11 Experiment	1D
12 Number of Scans	8
13 Receiver Gain	67
14 Relaxation Delay	1.0000
15 Pulse Width	10.0000
16 Acquisition Time	2.7525
17 Acquisition Date	2019-10-10T09:41:46
18 Modification Date	2019-10-10T10:03:15
19 Spectrometer Frequency	600.15
20 Spectral Width	11904.8
21 Lowest Frequency	-2260.7
22 Nucleus	1H
23 Acquired Size	32768
24 Spectral Size	65536

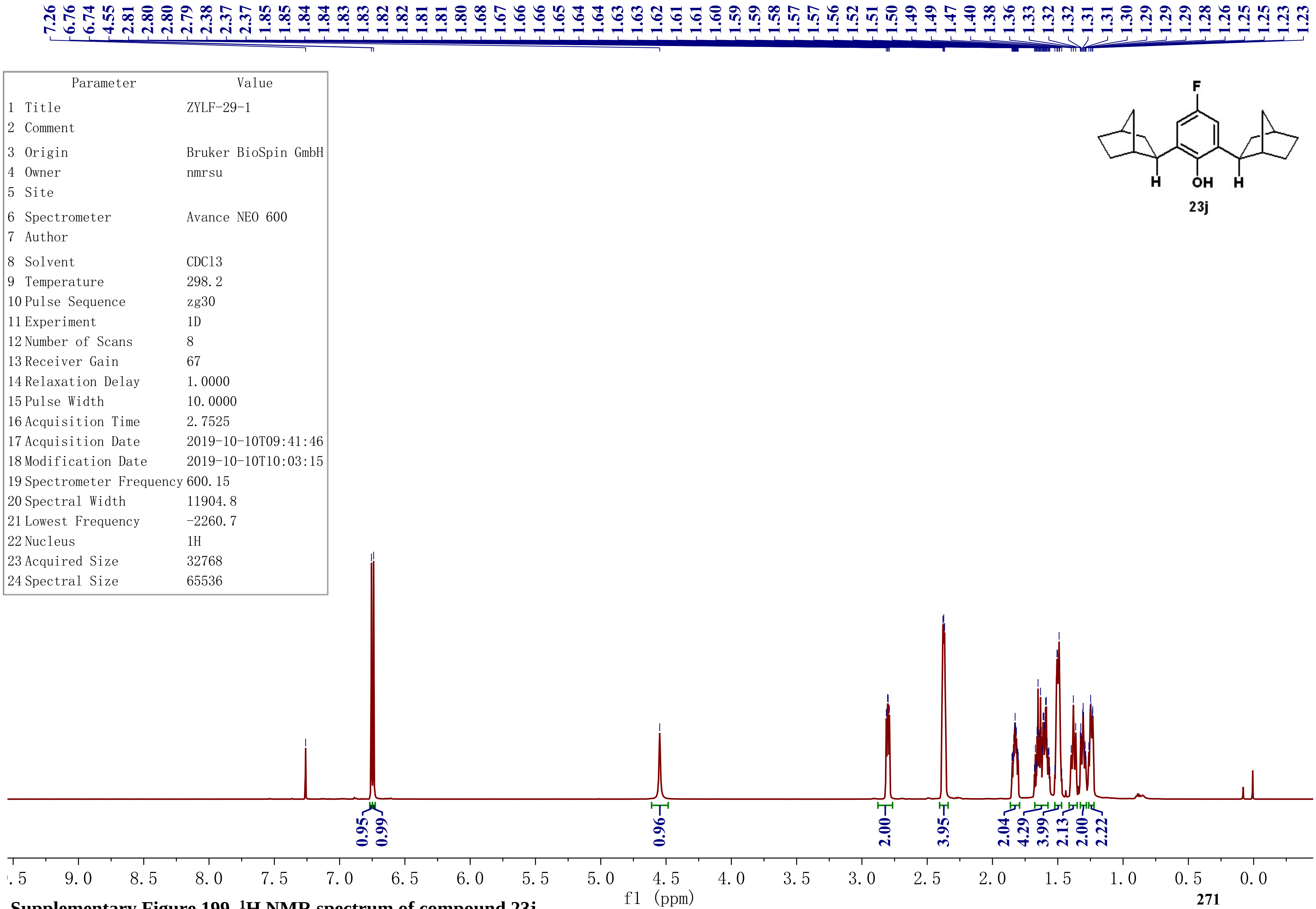

Supplementary Figure 199. ^{1}H NMR spectrum of compound 23j

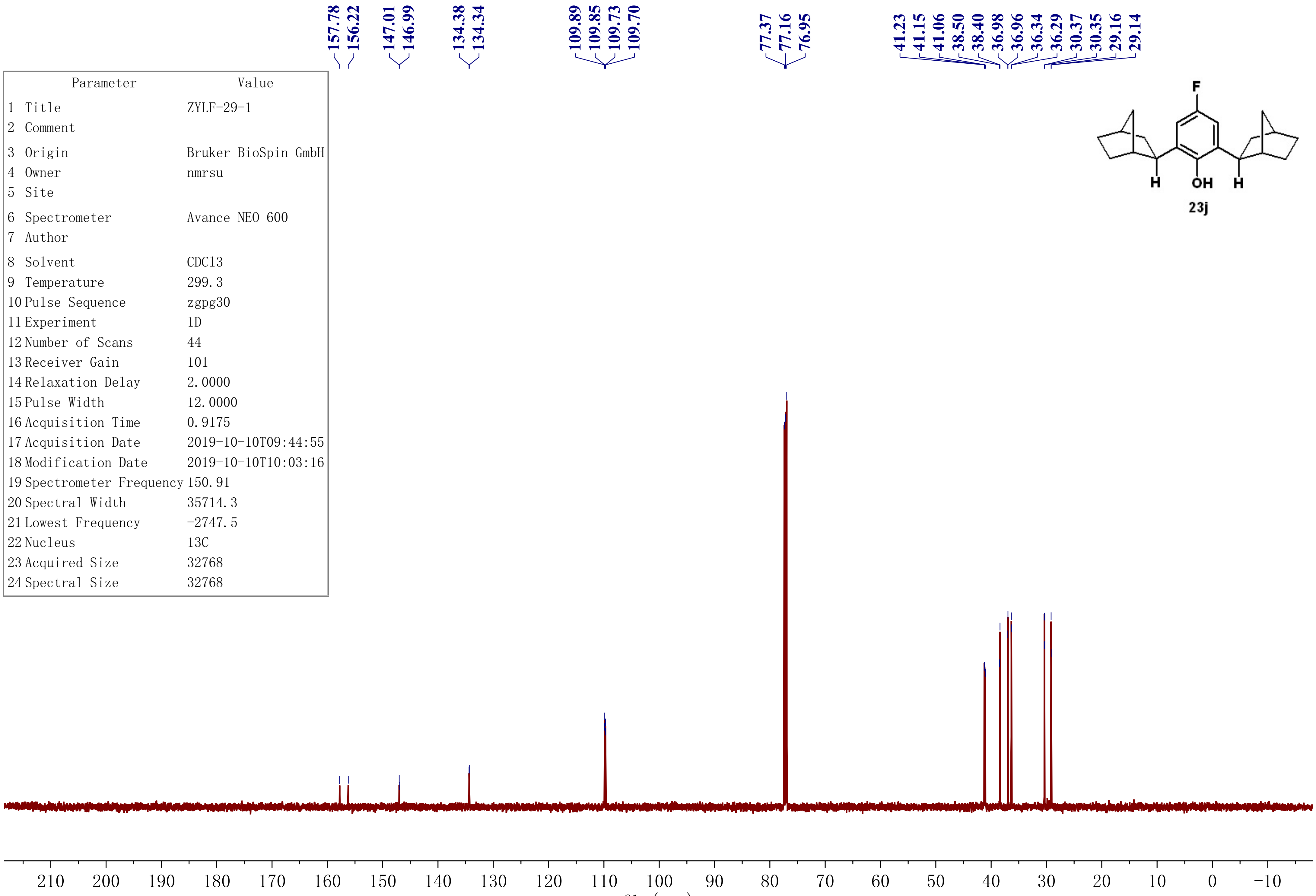

	Parameter	Value
1	Title	ZYLF-29-1
2	Comment	
3	Origin	Bruker BioSpin GmbH
4	Owner	nmrsu
5	Site	
6	Spectrometer	Avance NEO 600
7	Author	
8	Solvent	CDCl3
9	Temperature	299.3
10	Pulse Sequence	zgpg30
11	Experiment	1D
12	Number of Scans	44
13	Receiver Gain	101
14	Relaxation Delay	2.0000
15	Pulse Width	12.0000
16	Acquisition Time	0.9175
17	Acquisition Date	2019-10-10T09:44:55
18	Modification Date	2019-10-10T10:03:16
19	Spectrometer Frequency	150.91
20	Spectral Width	35714.3
21	Lowest Frequency	-2747.5
22	Nucleus	13C
23	Acquired Size	32768
24	Spectral Size	32768

23j

Supplementary Figure 200. ^{13}C NMR spectrum of compound 23j

Parameter	Value
1 Title	ZYLF-29-1
2 Comment	
3 Origin	Bruker BioSpin GmbH
4 Owner	nmrsu
5 Site	
6 Spectrometer	Avance NEO 600
7 Author	
8 Solvent	CDCl3
9 Temperature	298.8
10 Pulse Sequence	zgig
11 Experiment	1D
12 Number of Scans	16
13 Receiver Gain	101
14 Relaxation Delay	1.0000
15 Pulse Width	12.0000
16 Acquisition Time	0.4981
17 Acquisition Date	2019-10-20T13:53:28
18 Modification Date	2019-10-21T09:12:58
19 Spectrometer Frequency	564.71
20 Spectral Width	131579.0
21 Lowest Frequency	-122260.0
22 Nucleus	19F
23 Acquired Size	65536
24 Spectral Size	65536

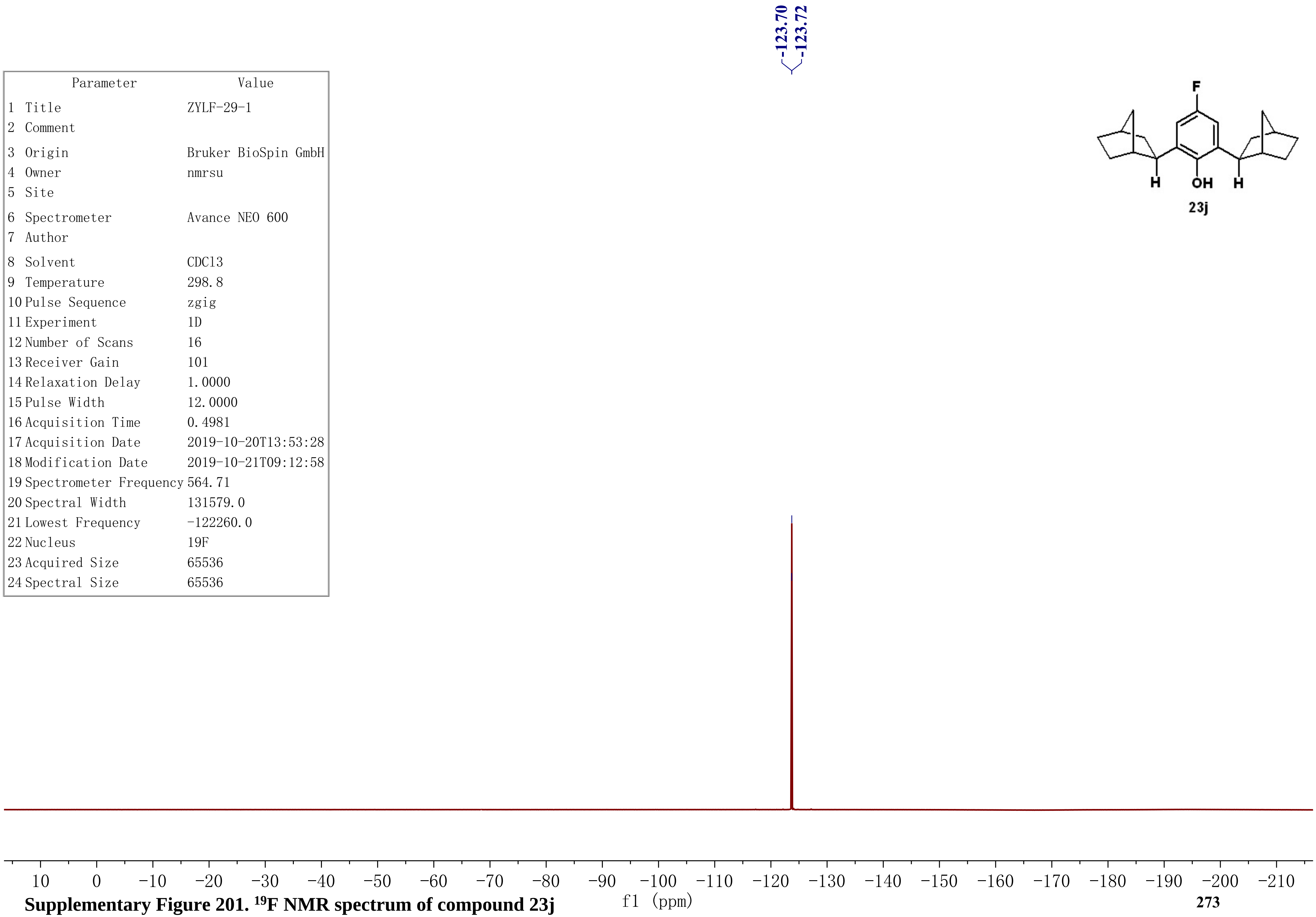

Supplementary Figure 201. ^{19}F NMR spectrum of compound 23j

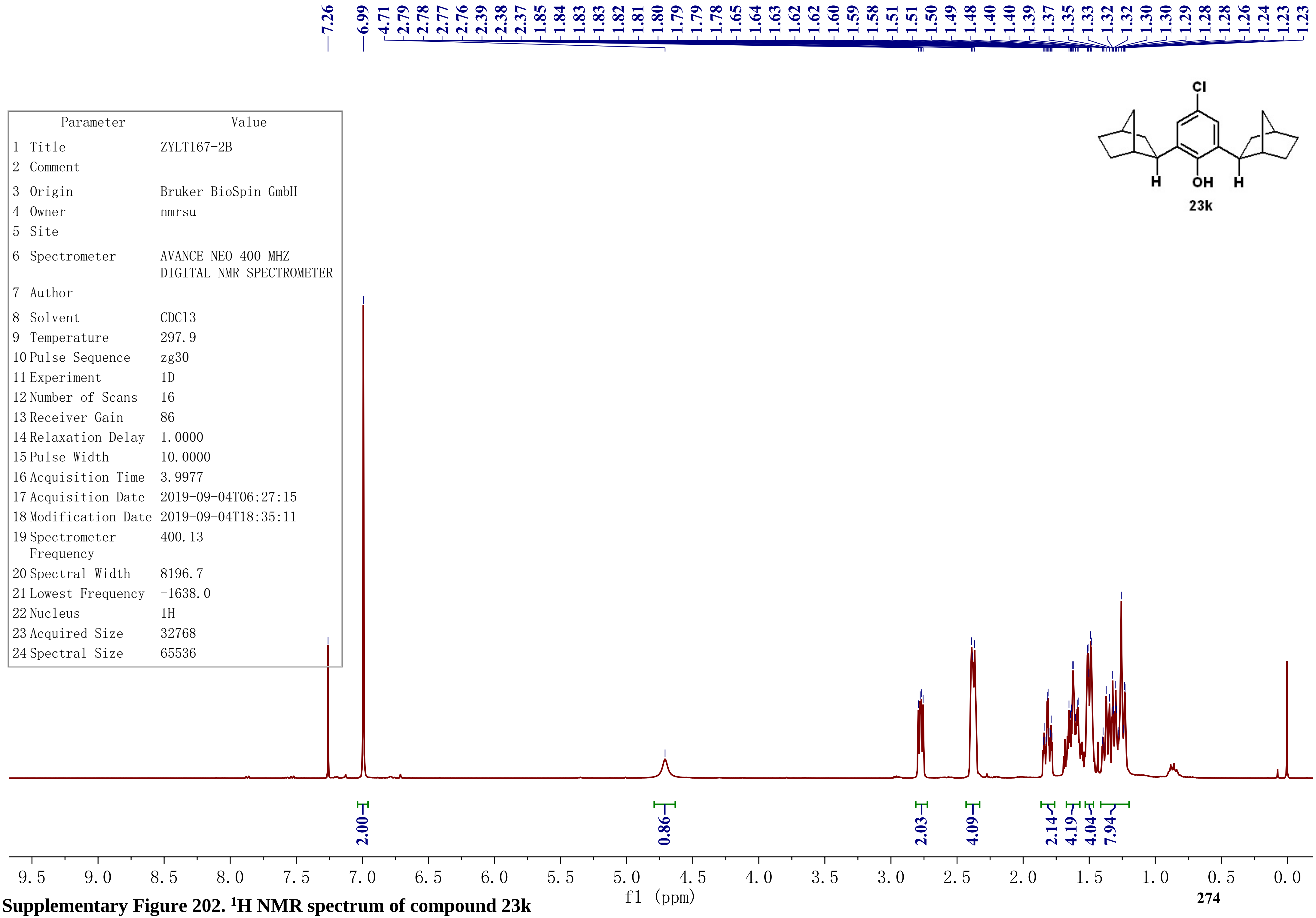

Supplementary Figure 202. ^{1}H NMR spectrum of compound 23k

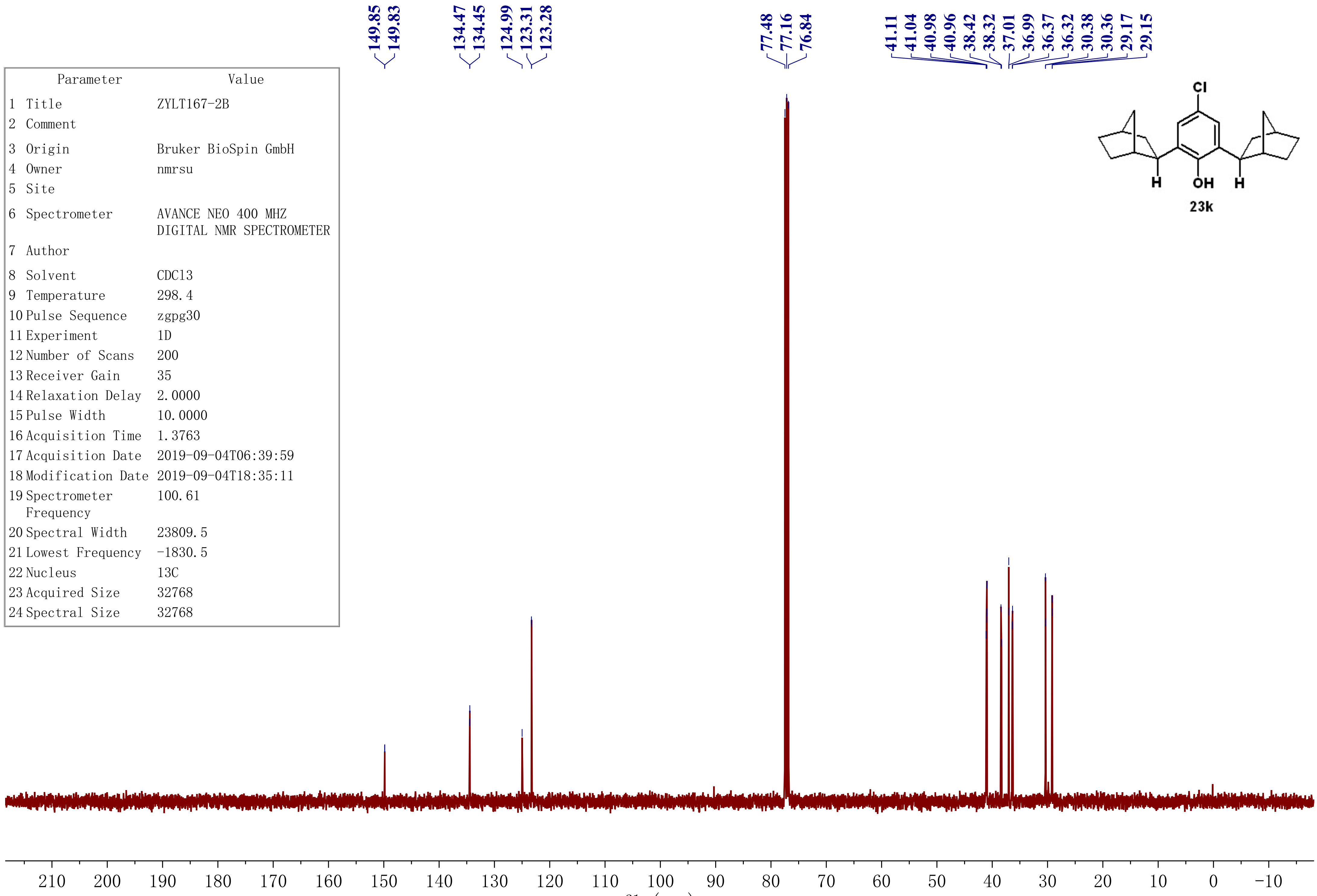

Supplementary Figure 203. ^{13}C NMR spectrum of compound 23k

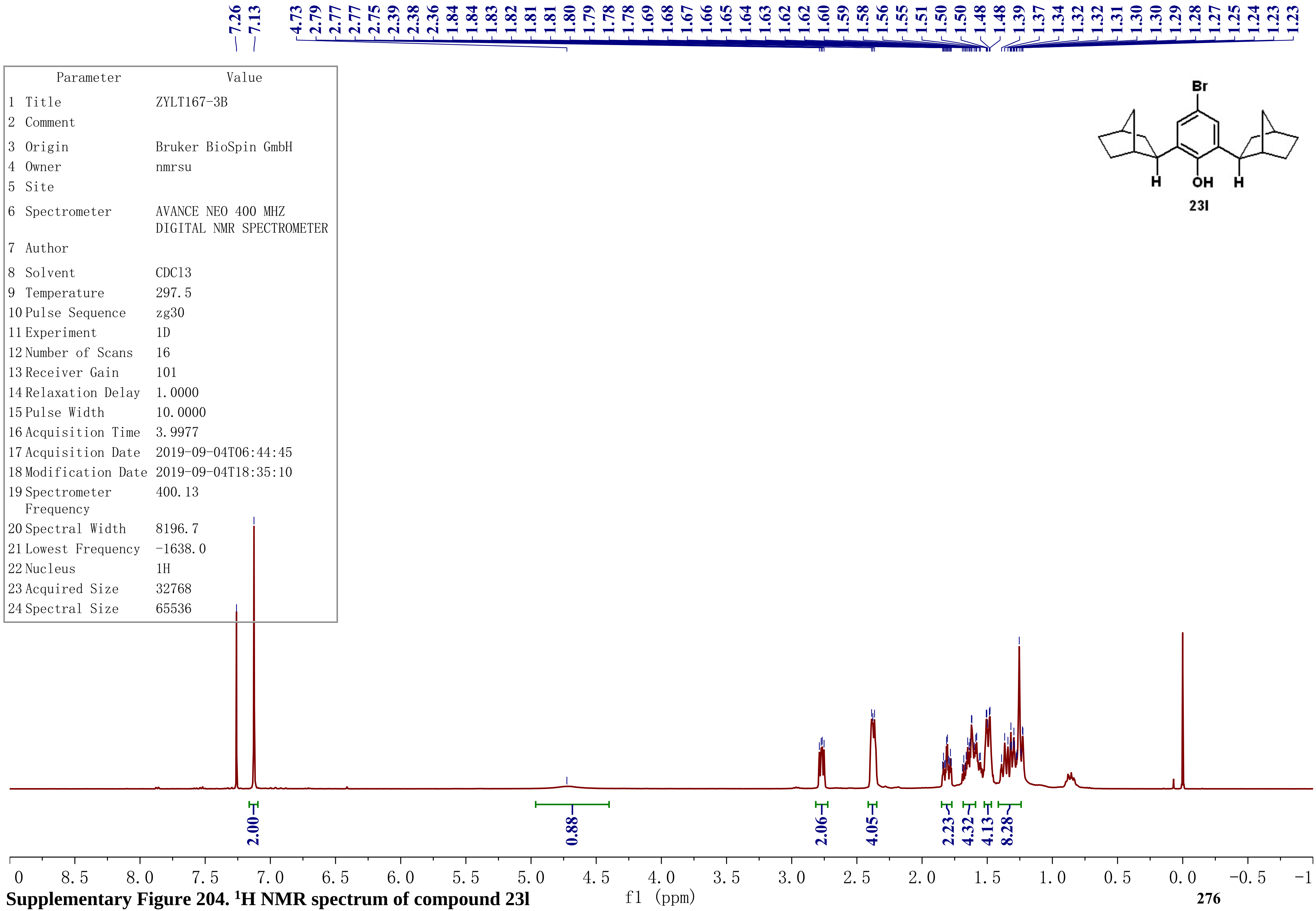

Parameter	Value
1 Title	ZYLT167-3B
2 Comment	
3 Origin	Bruker BioSpin GmbH
4 Owner	nmrsu
5 Site	
6 Spectrometer	AVANCE NEO 400 MHZ DIGITAL NMR SPECTROMETER
7 Author	
8 Solvent	CDCl3
9 Temperature	297.5
10 Pulse Sequence	zg30
11 Experiment	1D
12 Number of Scans	16
13 Receiver Gain	101
14 Relaxation Delay	1.0000
15 Pulse Width	10.0000
16 Acquisition Time	3.9977
17 Acquisition Date	2019-09-04T06:44:45
18 Modification Date	2019-09-04T18:35:10
19 Spectrometer Frequency	400.13
20 Spectral Width	8196.7
21 Lowest Frequency	-1638.0
22 Nucleus	1H
23 Acquired Size	32768
24 Spectral Size	65536

Supplementary Figure 204. ^{1}H NMR spectrum of compound 23l

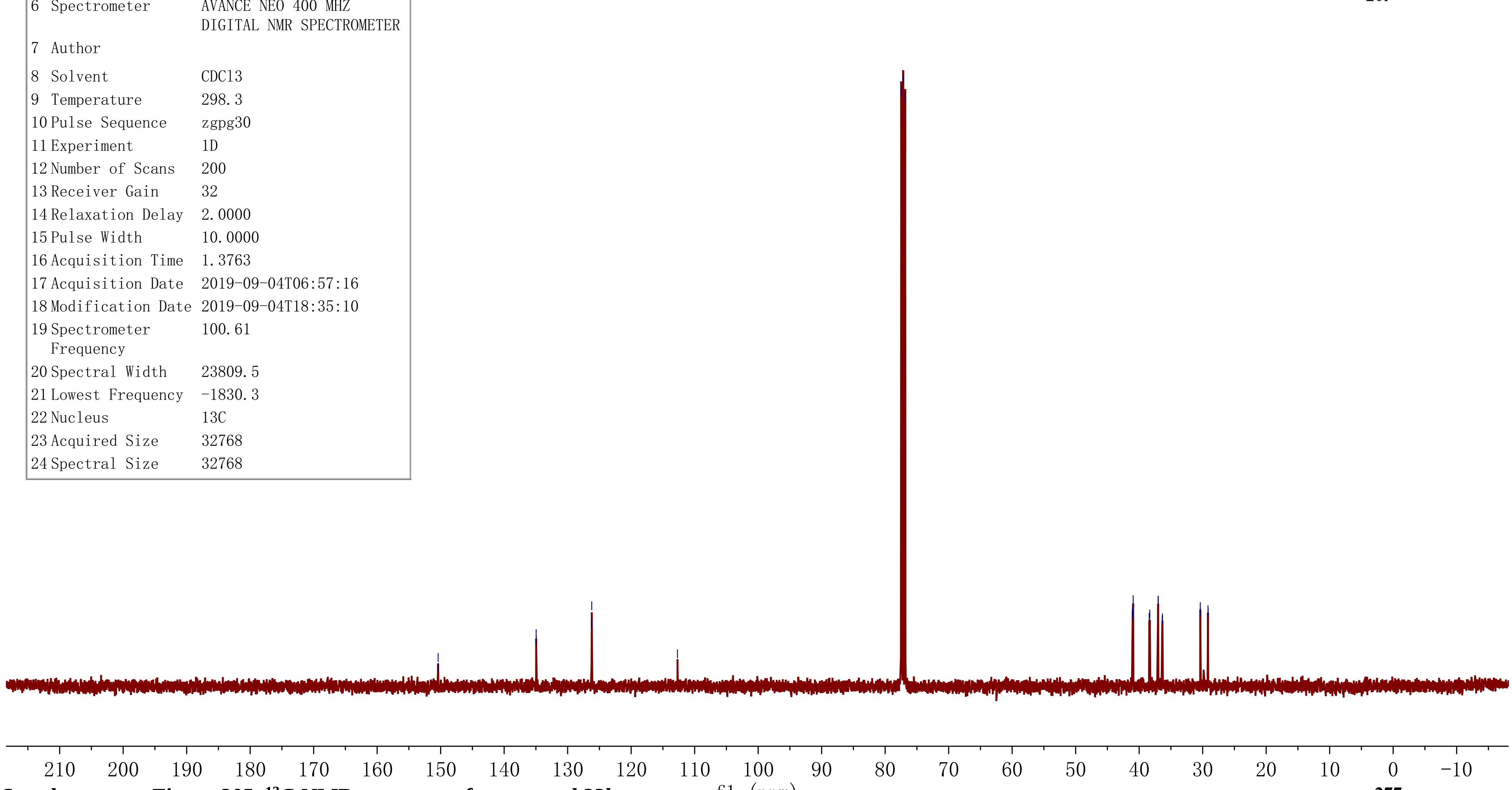

Parameter		Value
1	Title	ZYLT167-3B
2	Comment	
3	Origin	Bruker BioSpin GmbH
4	Owner	nmrsu
5	Site	
6	Spectrometer	AVANCE NEO 400 MHZ DIGITAL NMR SPECTROMETER
7	Author	
8	Solvent	CDCl3
9	Temperature	298.3
10	Pulse Sequence	zgpg30
11	Experiment	1D
12	Number of Scans	200
13	Receiver Gain	32
14	Relaxation Delay	2.0000
15	Pulse Width	10.0000
16	Acquisition Time	1.3763
17	Acquisition Date	2019-09-04T06:57:16
18	Modification Date	2019-09-04T18:35:10
19	Spectrometer Frequency	100.61
20	Spectral Width	23809.5
21	Lowest Frequency	-1830.3
22	Nucleus	13C
23	Acquired Size	32768
24	Spectral Size	32768

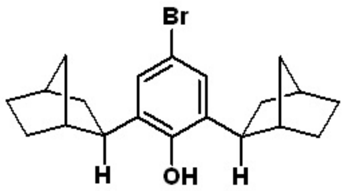

23l

Supplementary Figure 205. ^{13}C NMR spectrum of compound 23l

Parameter	Value
1 Title	XJ5-185
2 Comment	
3 Origin	Bruker BioSpin GmbH
4 Owner	nmrsu
5 Site	
6 Spectrometer	Avance NEO 600
7 Author	
8 Solvent	CDCl3
9 Temperature	298.5
10 Pulse Sequence	zg30
11 Experiment	1D
12 Number of Scans	8
13 Receiver Gain	78
14 Relaxation Delay	1.0000
15 Pulse Width	10.0000
16 Acquisition Time	2.7525
17 Acquisition Date	2020-08-11T17:39:30
18 Modification Date	2020-08-11T18:18:10
19 Spectrometer Frequency	600.15
20 Spectral Width	11904.8
21 Lowest Frequency	-2255.4
22 Nucleus	1H
23 Acquired Size	32768
24 Spectral Size	65536

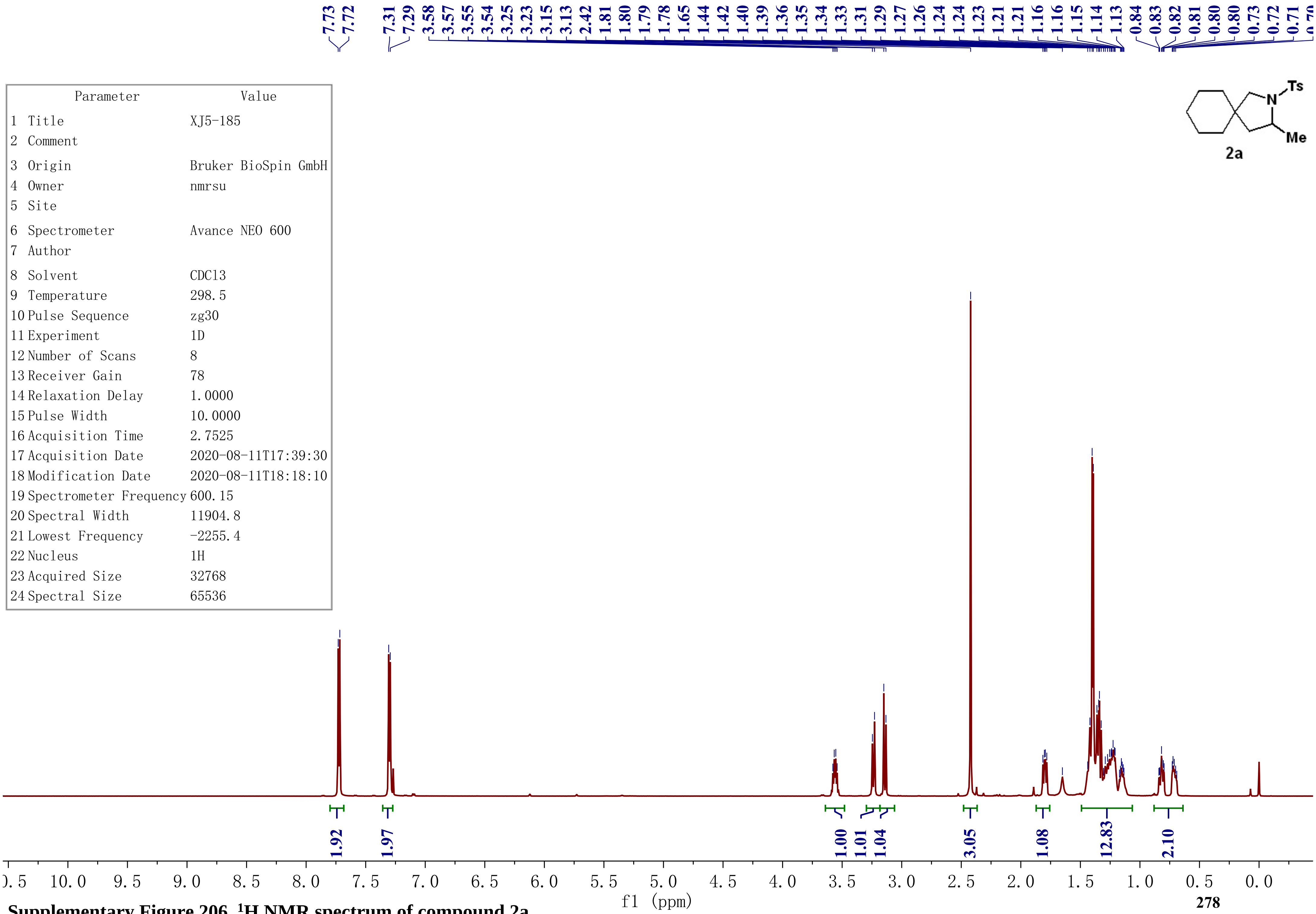

Supplementary Figure 206. ¹H NMR spectrum of compound 2a

Parameter	Value
1 Title	XJ5-185
2 Comment	
3 Origin	Bruker BioSpin GmbH
4 Owner	nmrsu
5 Site	
6 Spectrometer	Avance NEO 600
7 Author	
8 Solvent	CDCl3
9 Temperature	299.5
10 Pulse Sequence	zgpg30
11 Experiment	1D
12 Number of Scans	65
13 Receiver Gain	101
14 Relaxation Delay	2.0000
15 Pulse Width	12.0000
16 Acquisition Time	0.9175
17 Acquisition Date	2020-08-11T17:44:02
18 Modification Date	2020-08-11T18:18:11
19 Spectrometer Frequency	150.91
20 Spectral Width	35714.3
21 Lowest Frequency	-2766.4
22 Nucleus	13C
23 Acquired Size	32768
24 Spectral Size	32768

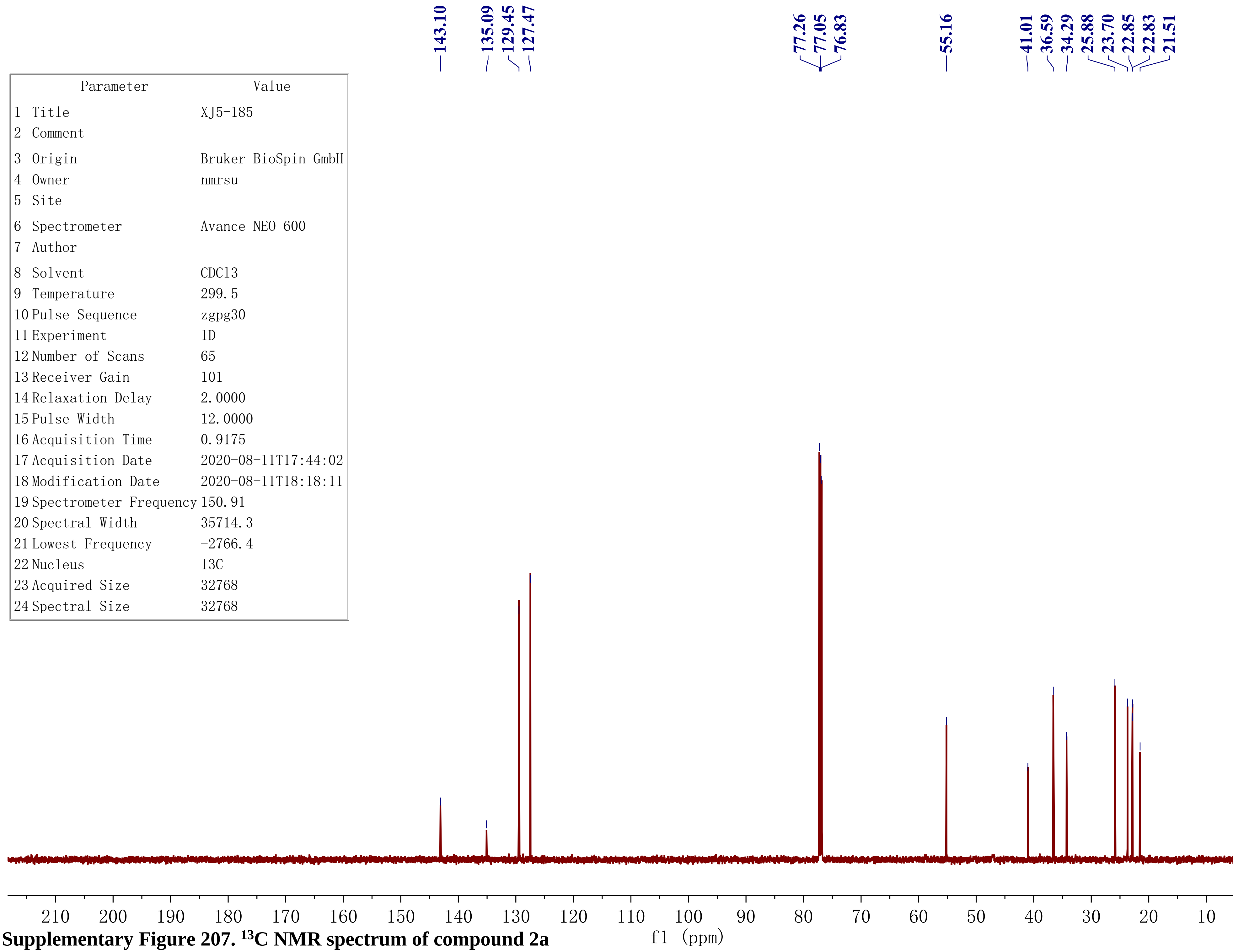

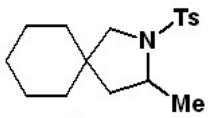

2a

Supplementary Figure 207. ^{13}C NMR spectrum of compound 2a

Parameter	Value
1 Title	XJ2-21
2 Comment	
3 Origin	Bruker BioSpin GmbH
4 Owner	nmrsu
5 Site	
6 Spectrometer	AVANCE NEO 400 MHZ DIGITAL NMR SPECTROMETER
7 Author	
8 Solvent	CDCl3
9 Temperature	295.0
10 Pulse Sequence	zg30
11 Experiment	1D
12 Number of Scans	12
13 Receiver Gain	80
14 Relaxation Delay	1.0000
15 Pulse Width	10.0000
16 Acquisition Time	3.9977
17 Acquisition Date	2018-11-10T21:19:50
18 Modification Date	2019-07-27T09:49:15
19 Spectrometer Frequency	400.13
20 Spectral Width	8196.7
21 Lowest Frequency	-1634.7
22 Nucleus	1H
23 Acquired Size	32768
24 Spectral Size	65536

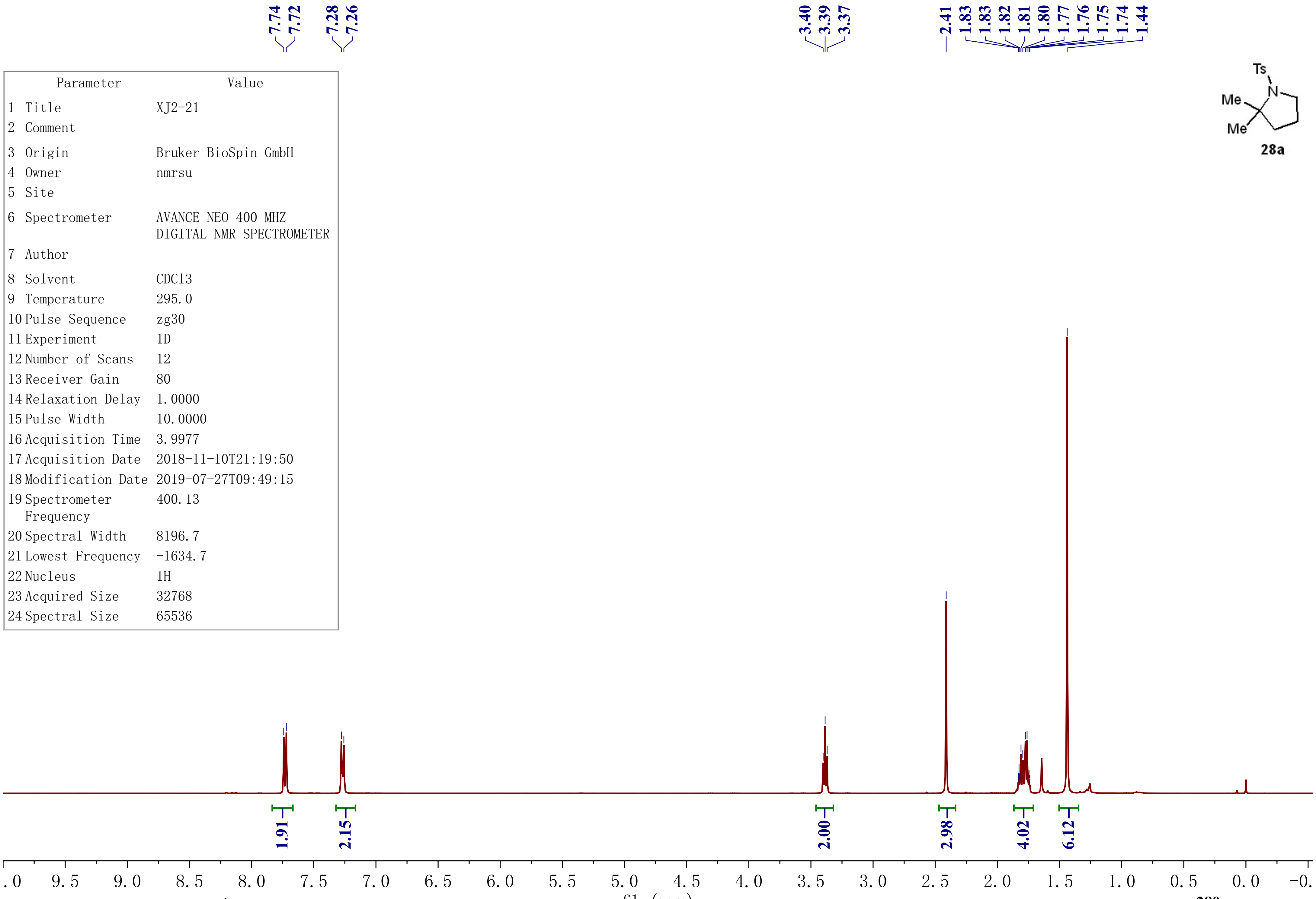

28a

Supplementary Figure 208. ^{1}H **NMR spectrum of compound 28a**

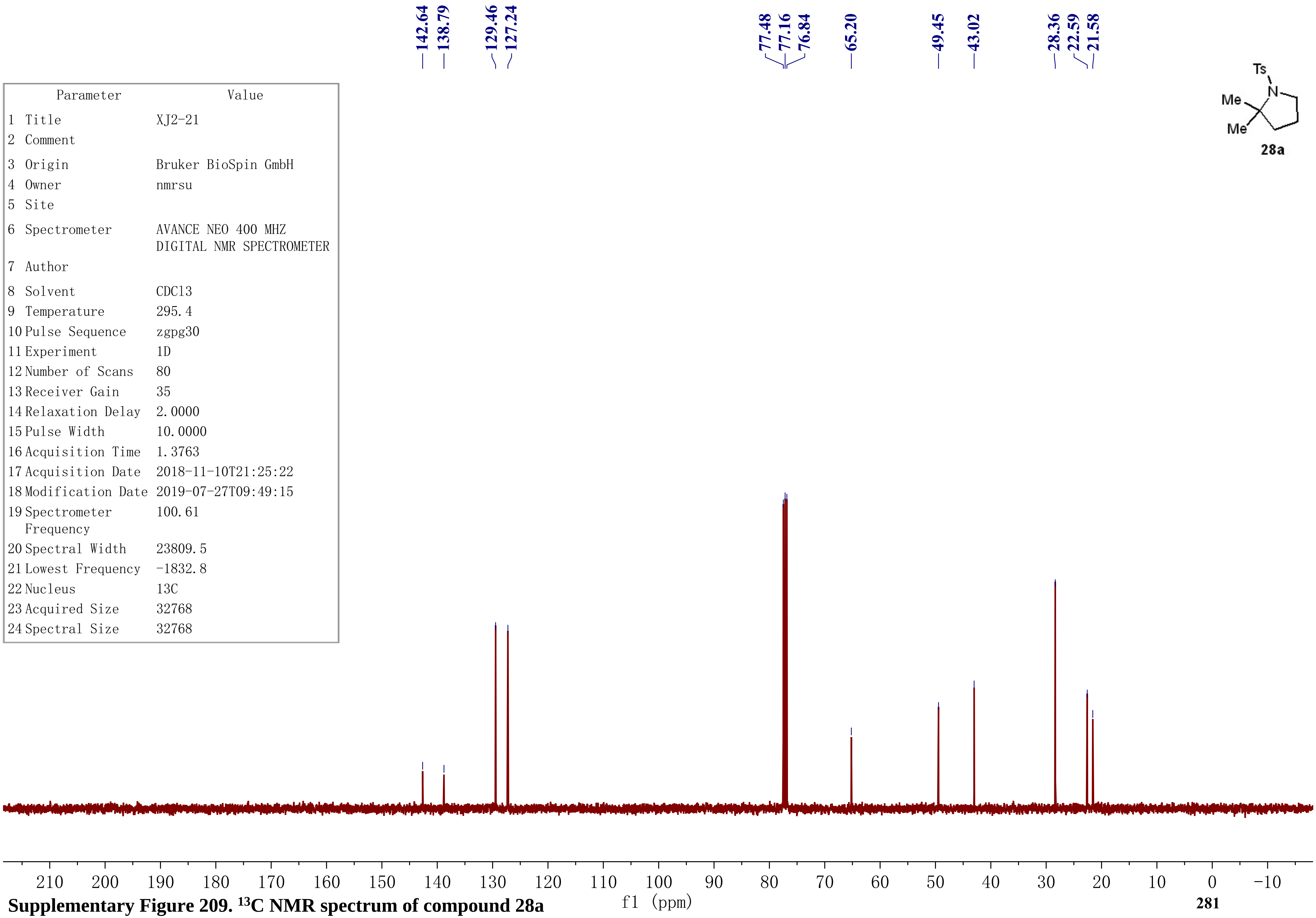

Parameter		Value
1	Title	XJ2-21
2	Comment	
3	Origin	Bruker BioSpin GmbH
4	Owner	nmrsu
5	Site	
6	Spectrometer	AVANCE NEO 400 MHZ DIGITAL NMR SPECTROMETER
7	Author	
8	Solvent	CDCl3
9	Temperature	295.4
10	Pulse Sequence	zgpg30
11	Experiment	1D
12	Number of Scans	80
13	Receiver Gain	35
14	Relaxation Delay	2.0000
15	Pulse Width	10.0000
16	Acquisition Time	1.3763
17	Acquisition Date	2018-11-10T21:25:22
18	Modification Date	2019-07-27T09:49:15
19	Spectrometer Frequency	100.61
20	Spectral Width	23809.5
21	Lowest Frequency	-1832.8
22	Nucleus	13C
23	Acquired Size	32768
24	Spectral Size	32768

28a

Supplementary Figure 209. ^{13}C NMR spectrum of compound 28a

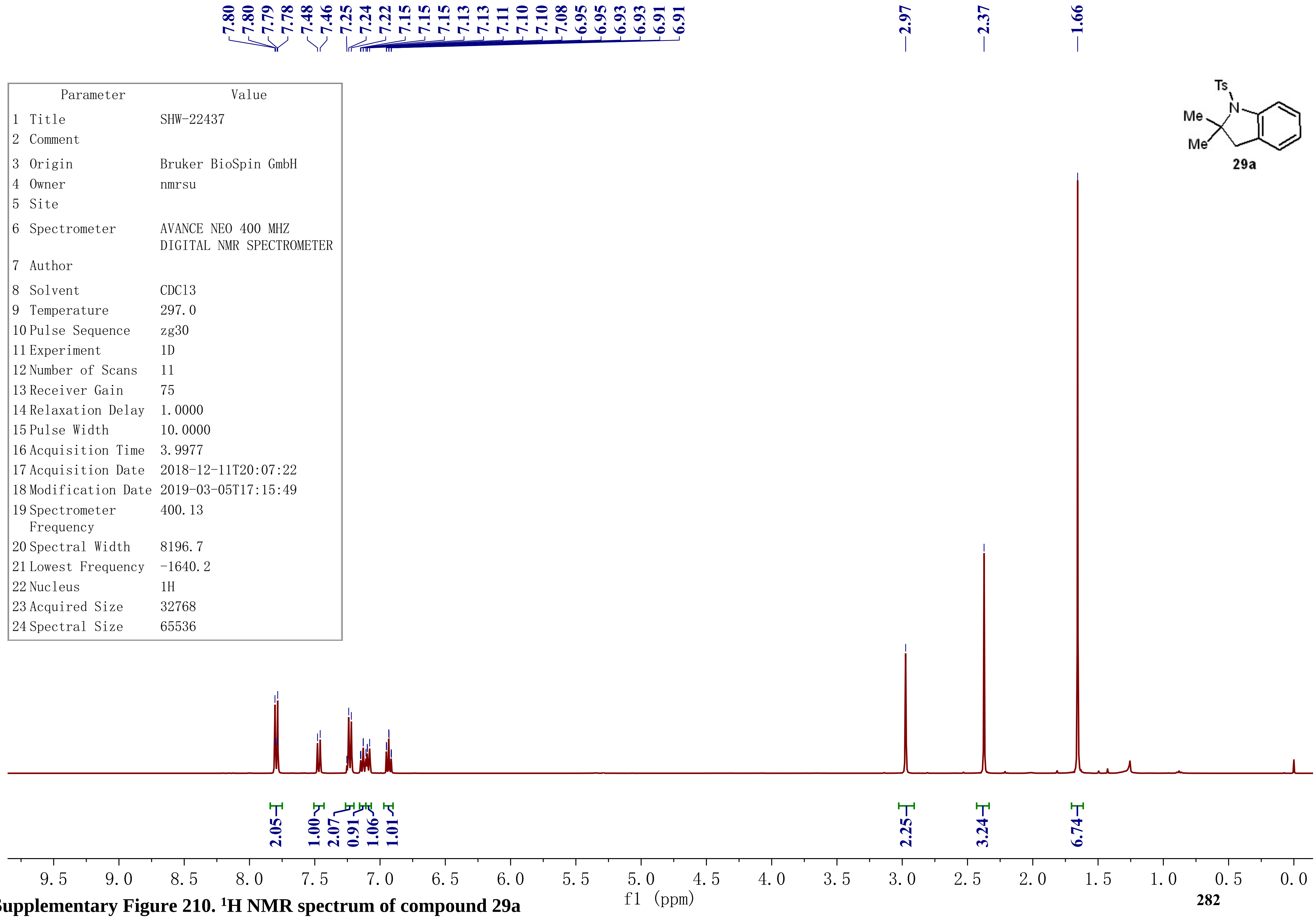

Supplementary Figure 210. ¹H NMR spectrum of compound 29a

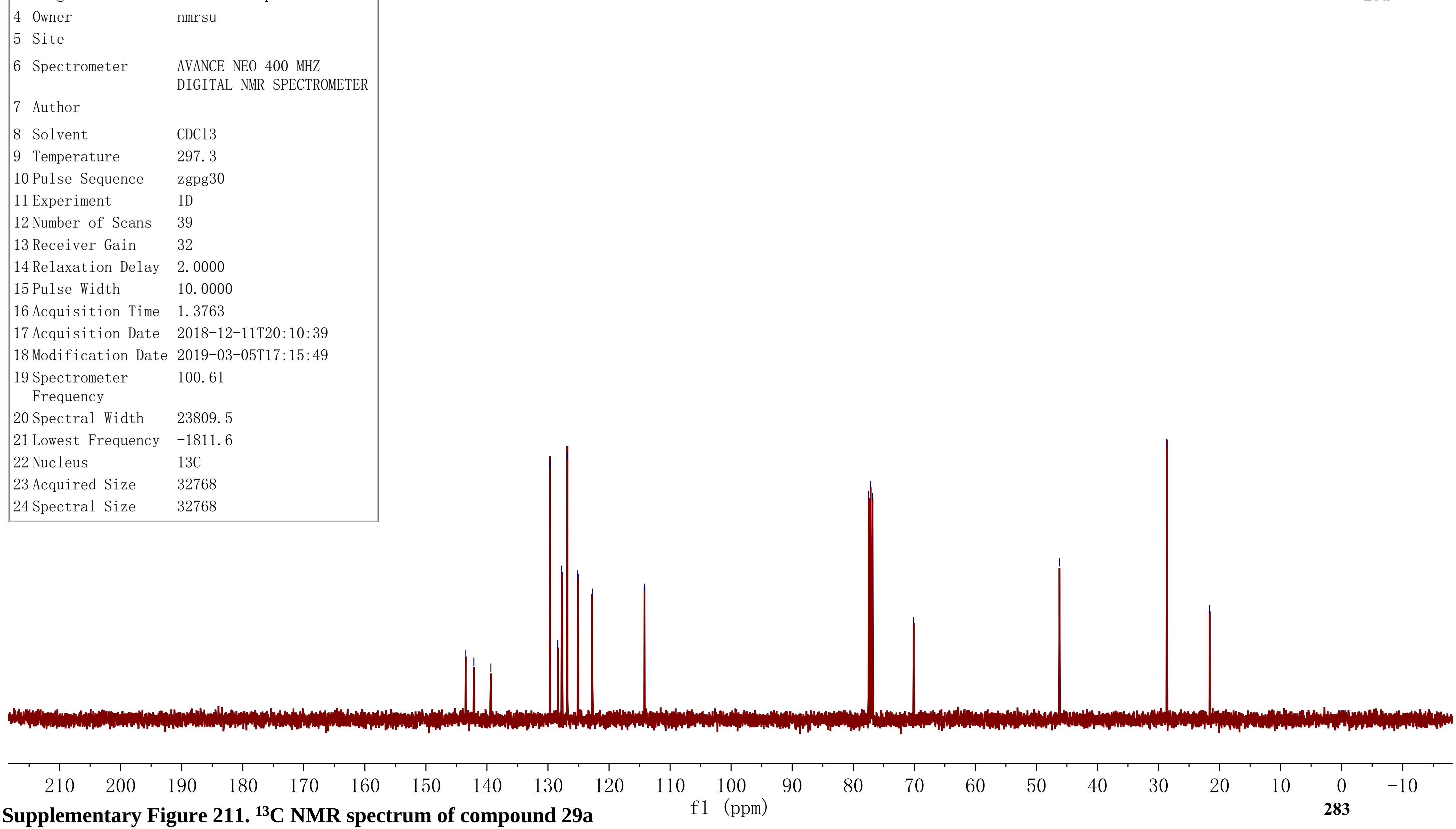

Parameter	Value
1 Title	SHW-22437
2 Comment	
3 Origin	Bruker BioSpin GmbH
4 Owner	nmrsu
5 Site	
6 Spectrometer	AVANCE NEO 400 MHZ DIGITAL NMR SPECTROMETER
7 Author	
8 Solvent	CDCl3
9 Temperature	297.3
10 Pulse Sequence	zgpg30
11 Experiment	1D
12 Number of Scans	39
13 Receiver Gain	32
14 Relaxation Delay	2.0000
15 Pulse Width	10.0000
16 Acquisition Time	1.3763
17 Acquisition Date	2018-12-11T20:10:39
18 Modification Date	2019-03-05T17:15:49
19 Spectrometer Frequency	100.61
20 Spectral Width	23809.5
21 Lowest Frequency	-1811.6
22 Nucleus	13C
23 Acquired Size	32768
24 Spectral Size	32768

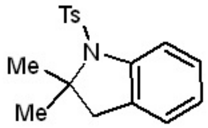

29a

Supplementary Figure 211. ^{13}C NMR spectrum of compound 29a

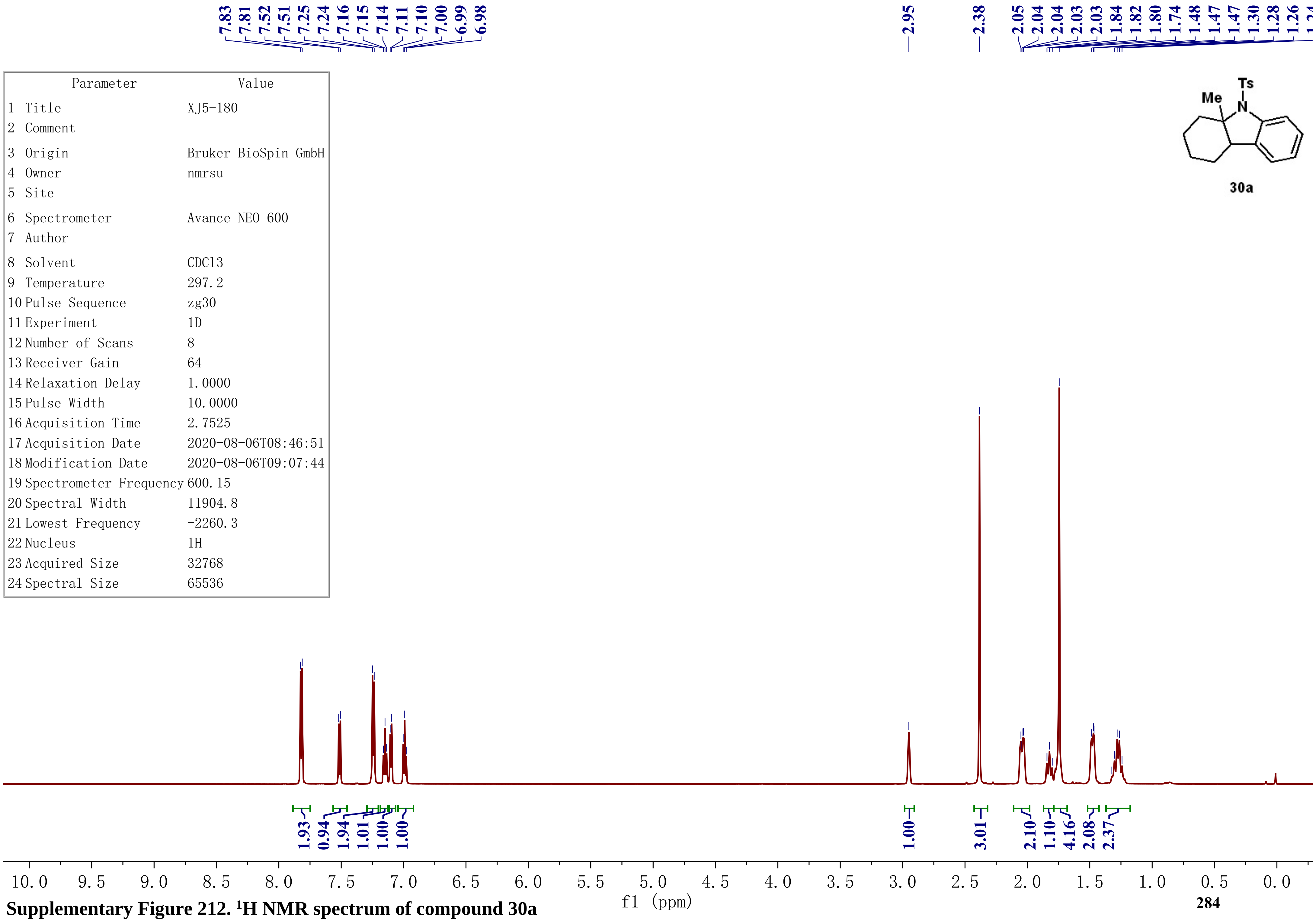

	Parameter	Value
1	Title	XJ5-180
2	Comment	
3	Origin	Bruker BioSpin GmbH
4	Owner	nmrsu
5	Site	
6	Spectrometer	Avance NEO 600
7	Author	
8	Solvent	CDCl3
9	Temperature	297.2
10	Pulse Sequence	zg30
11	Experiment	1D
12	Number of Scans	8
13	Receiver Gain	64
14	Relaxation Delay	1.0000
15	Pulse Width	10.0000
16	Acquisition Time	2.7525
17	Acquisition Date	2020-08-06T08:46:51
18	Modification Date	2020-08-06T09:07:44
19	Spectrometer Frequency	600.15
20	Spectral Width	11904.8
21	Lowest Frequency	-2260.3
22	Nucleus	1H
23	Acquired Size	32768
24	Spectral Size	65536

Supplementary Figure 212. ^{1}H NMR spectrum of compound 30a

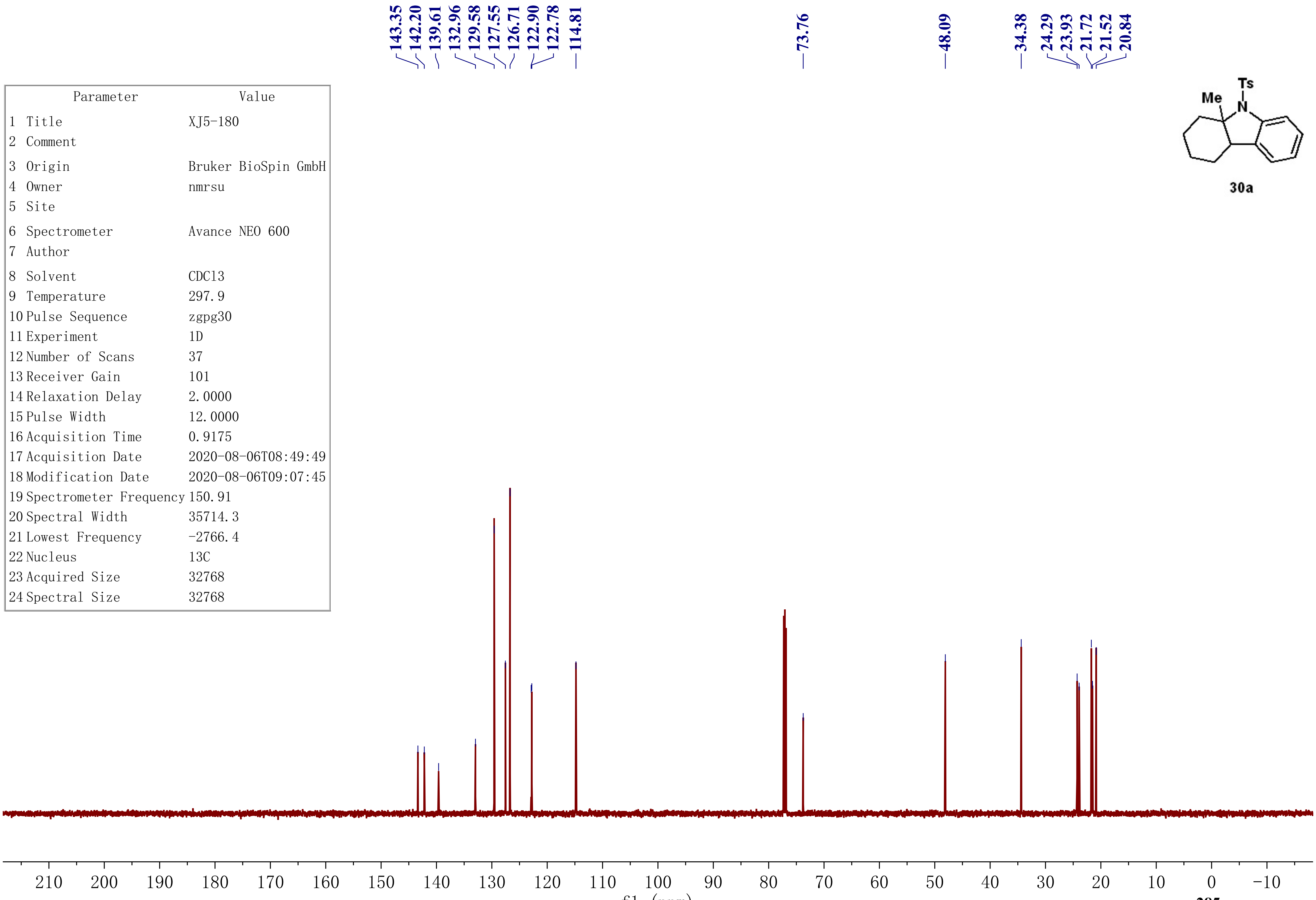

	Parameter	Value
1	Title	XJ5-180
2	Comment	
3	Origin	Bruker BioSpin GmbH
4	Owner	nmrsu
5	Site	
6	Spectrometer	Avance NEO 600
7	Author	
8	Solvent	CDCl3
9	Temperature	297.9
10	Pulse Sequence	zgpg30
11	Experiment	1D
12	Number of Scans	37
13	Receiver Gain	101
14	Relaxation Delay	2.0000
15	Pulse Width	12.0000
16	Acquisition Time	0.9175
17	Acquisition Date	2020-08-06T08:49:49
18	Modification Date	2020-08-06T09:07:45
19	Spectrometer Frequency	150.91
20	Spectral Width	35714.3
21	Lowest Frequency	-2766.4
22	Nucleus	13C
23	Acquired Size	32768
24	Spectral Size	32768

Supplementary Figure 213. ^{13}C NMR spectrum of compound 30a

Parameter	Value
1 Title	SHW-223168-2
2 Comment	
3 Origin	Bruker BioSpin GmbH
4 Owner	nmrsu
5 Site	
6 Spectrometer	AVANCE NEO 400 MHZ DIGITAL NMR SPECTROMETER
7 Author	
8 Solvent	CDCl3
9 Temperature	295.2
10 Pulse Sequence	zg30
11 Experiment	1D
12 Number of Scans	8
13 Receiver Gain	50
14 Relaxation Delay	1.0000
15 Pulse Width	10.0000
16 Acquisition Time	3.9977
17 Acquisition Date	2018-11-09T14:19:38
18 Modification Date	2018-11-09T15:15:29
19 Spectrometer Frequency	400.13
20 Spectral Width	8196.7
21 Lowest Frequency	-1639.6
22 Nucleus	1H
23 Acquired Size	32768
24 Spectral Size	65536

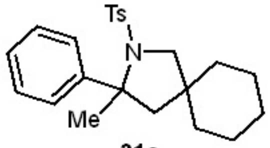

31a

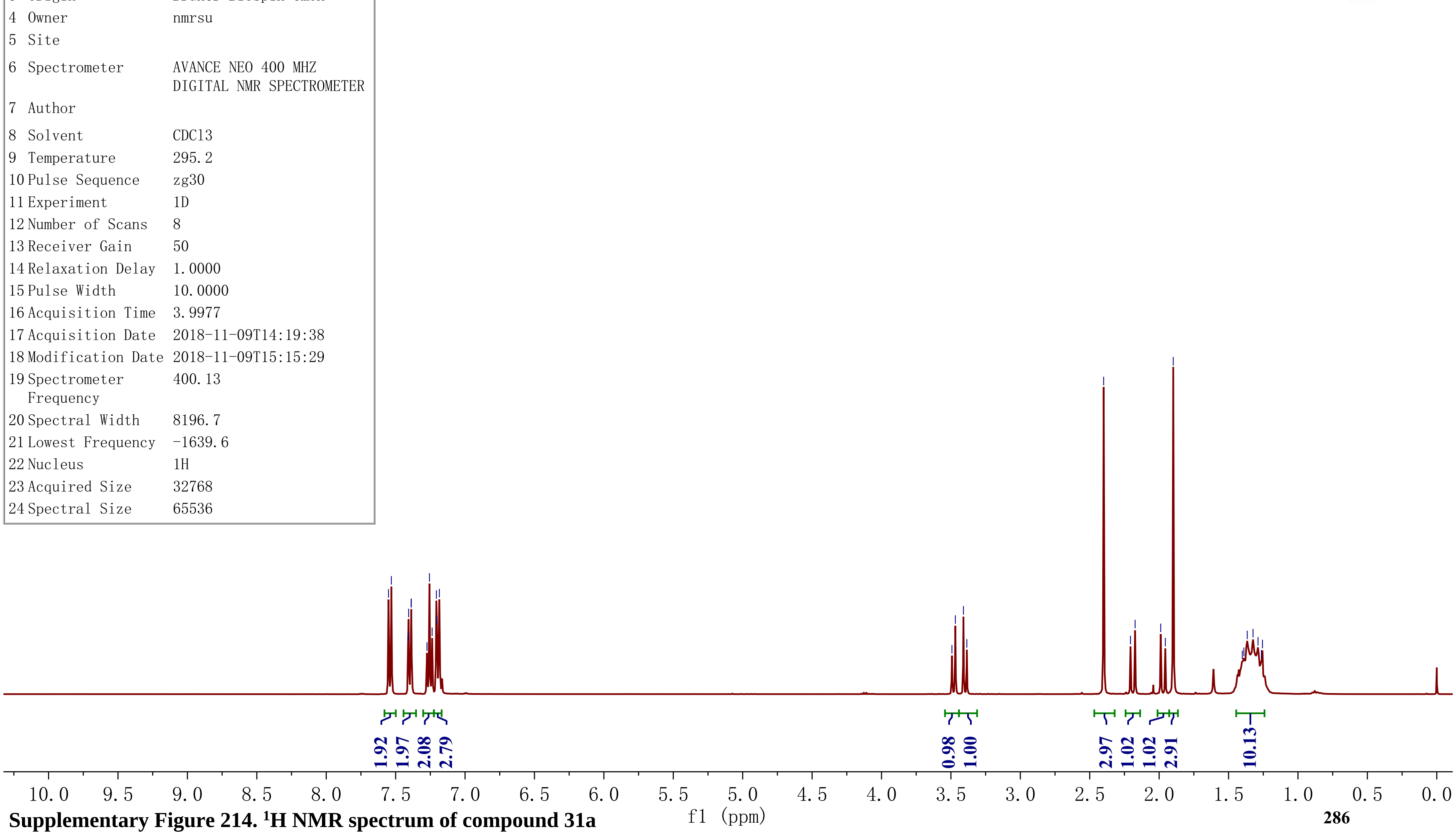

Supplementary Figure 214. ^{1}H NMR spectrum of compound 31a

Parameter	Value
1 Title	SHW-223168-2
2 Comment	
3 Origin	Bruker BioSpin GmbH
4 Owner	nmrsu
5 Site	
6 Spectrometer	AVANCE NEO 400 MHZ DIGITAL NMR SPECTROMETER
7 Author	
8 Solvent	CDCl3
9 Temperature	295.7
10 Pulse Sequence	zgpg30
11 Experiment	1D
12 Number of Scans	34
13 Receiver Gain	35
14 Relaxation Delay	2.0000
15 Pulse Width	10.0000
16 Acquisition Time	1.3763
17 Acquisition Date	2018-11-09T14:22:50
18 Modification Date	2018-11-09T15:15:29
19 Spectrometer Frequency	100.61
20 Spectral Width	23809.5
21 Lowest Frequency	-1815.0
22 Nucleus	13C
23 Acquired Size	32768
24 Spectral Size	32768

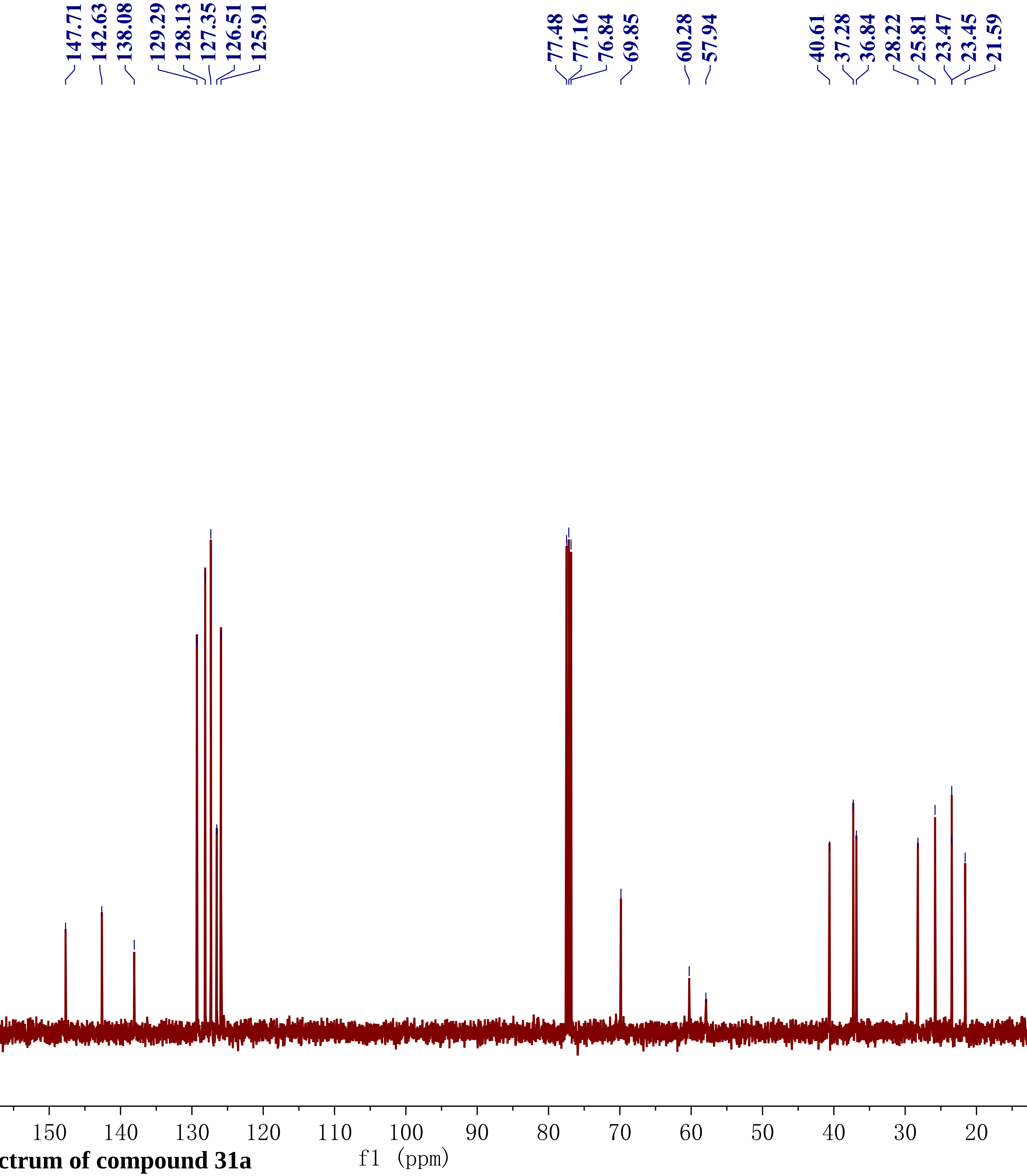

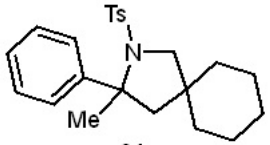

31a

Supplementary Figure 215. ^{13}C NMR spectrum of compound 31a

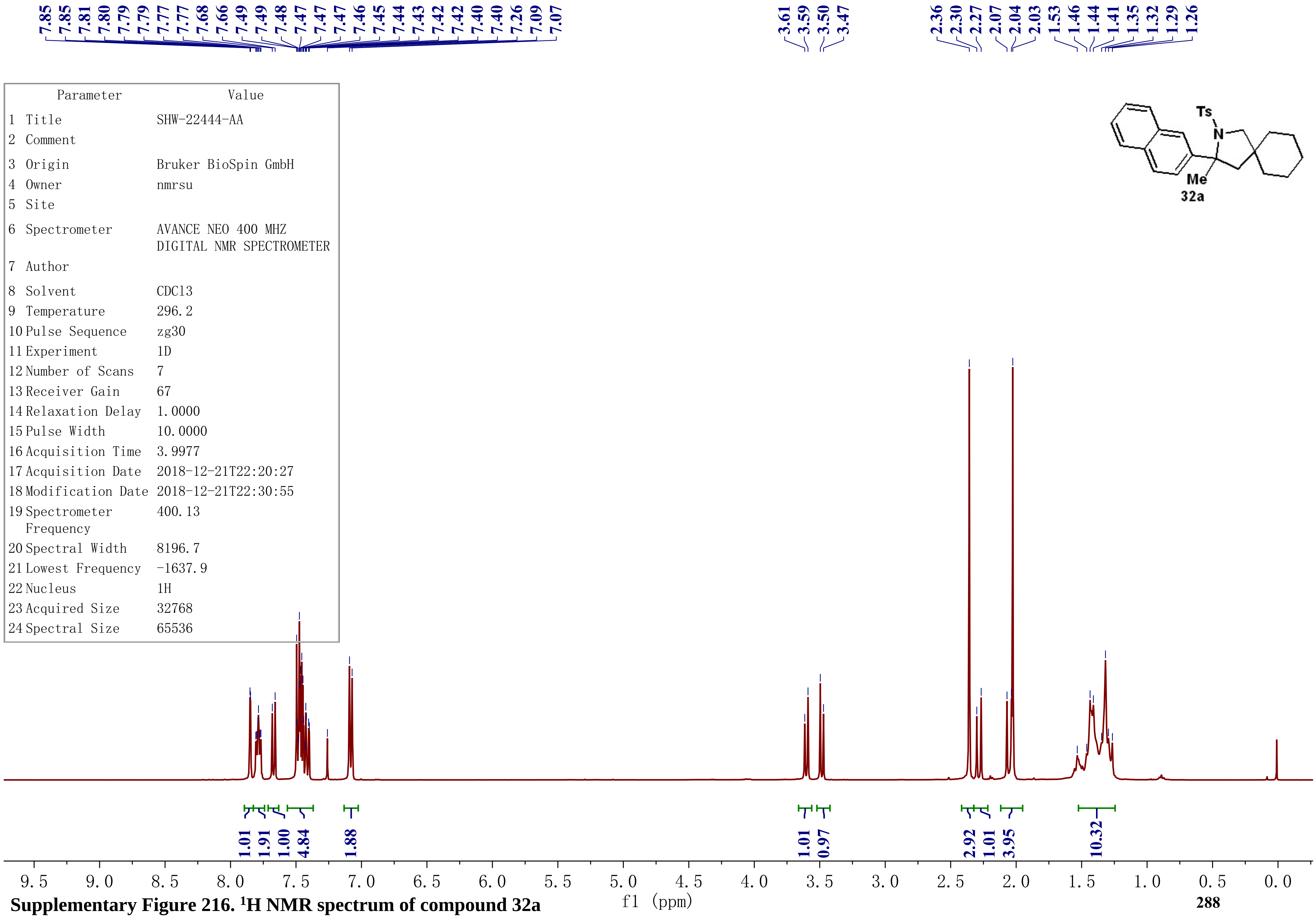

	Parameter	Value
1	Title	SHW-22444-AA
2	Comment	
3	Origin	Bruker BioSpin GmbH
4	Owner	nmrsu
5	Site	
6	Spectrometer	AVANCE NEO 400 MHZ DIGITAL NMR SPECTROMETER
7	Author	
8	Solvent	CDCl3
9	Temperature	296.2
10	Pulse Sequence	zg30
11	Experiment	1D
12	Number of Scans	7
13	Receiver Gain	67
14	Relaxation Delay	1.0000
15	Pulse Width	10.0000
16	Acquisition Time	3.9977
17	Acquisition Date	2018-12-21T22:20:27
18	Modification Date	2018-12-21T22:30:55
19	Spectrometer Frequency	400.13
20	Spectral Width	8196.7
21	Lowest Frequency	-1637.9
22	Nucleus	1H
23	Acquired Size	32768
24	Spectral Size	65536

32a

Supplementary Figure 216. ^{1}H NMR spectrum of compound 32a

Parameter	Value
1 Title	SHW-22444-AA
2 Comment	
3 Origin	Bruker BioSpin GmbH
4 Owner	nmrsu
5 Site	
6 Spectrometer	AVANCE NEO 400 MHZ DIGITAL NMR SPECTROMETER
7 Author	
8 Solvent	CDCl3
9 Temperature	296.7
10 Pulse Sequence	zgpg30
11 Experiment	1D
12 Number of Scans	43
13 Receiver Gain	50
14 Relaxation Delay	2.0000
15 Pulse Width	10.0000
16 Acquisition Time	1.3763
17 Acquisition Date	2018-12-21T22:23:57
18 Modification Date	2018-12-21T22:30:55
19 Spectrometer Frequency	100.61
20 Spectral Width	23809.5
21 Lowest Frequency	-1812.5
22 Nucleus	13C
23 Acquired Size	32768
24 Spectral Size	32768

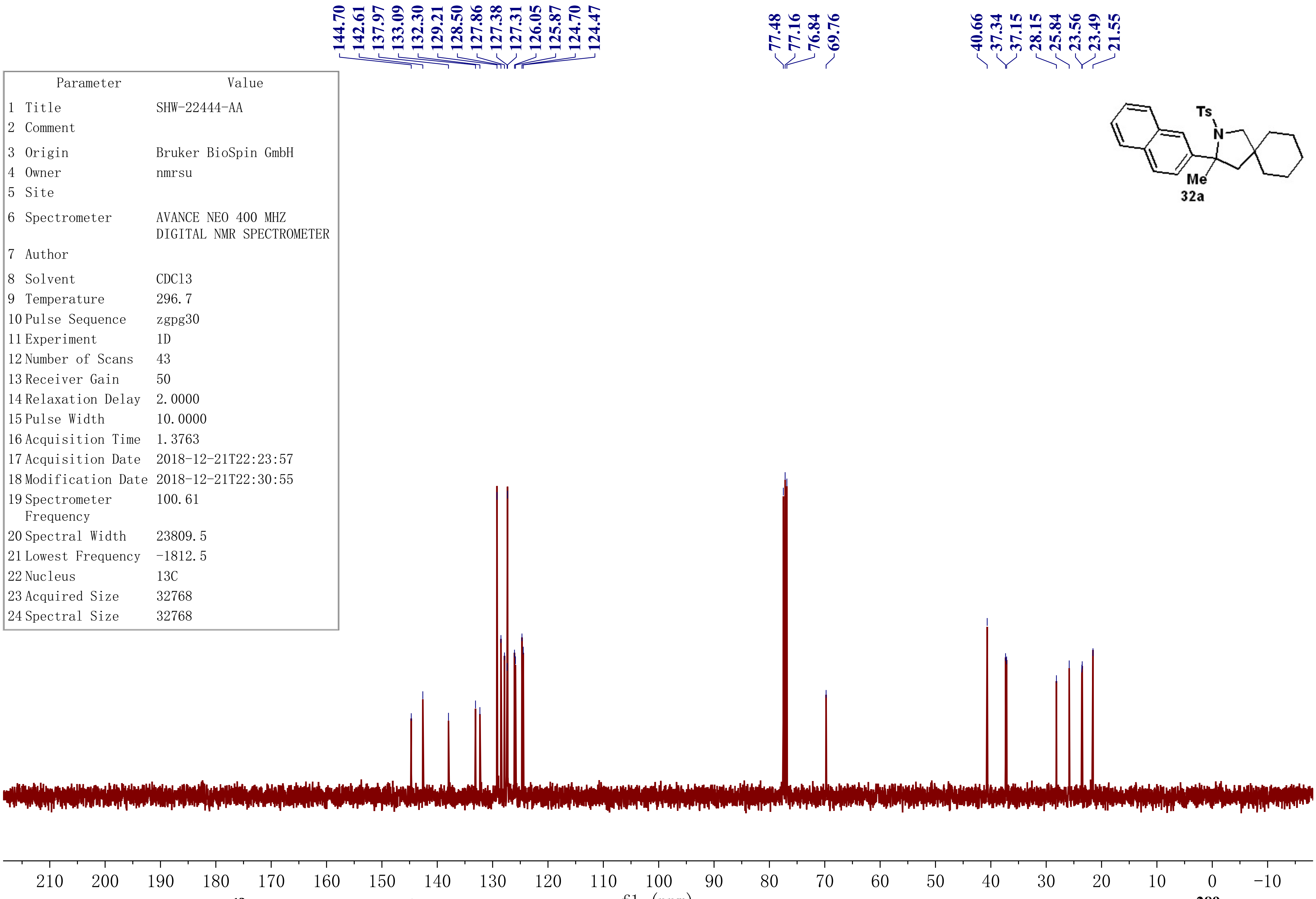

Supplementary Figure 217. ^{13}C NMR spectrum of compound 32a

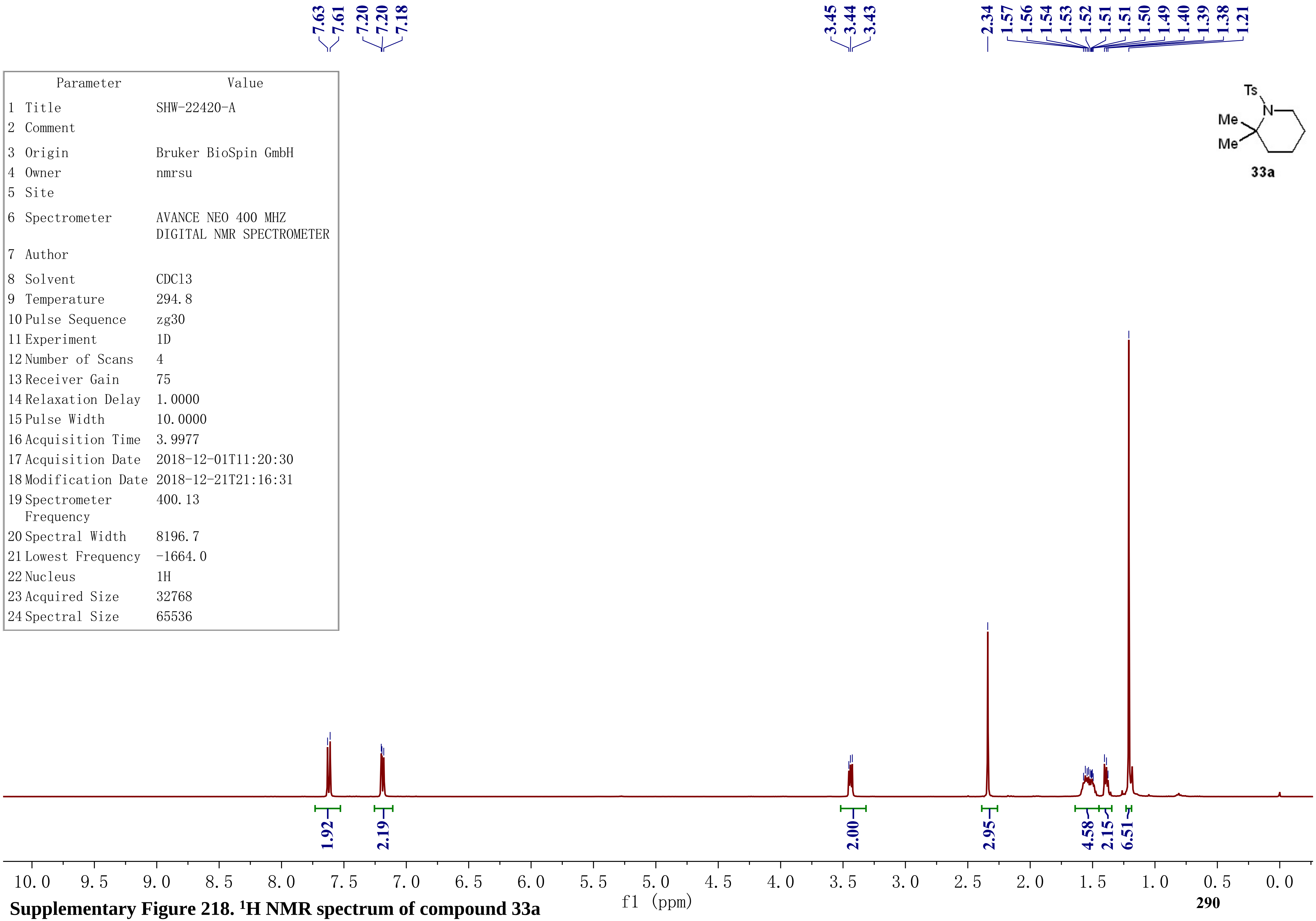

	Parameter	Value
1	Title	SHW-22420-A
2	Comment	
3	Origin	Bruker BioSpin GmbH
4	Owner	nmrsu
5	Site	
6	Spectrometer	AVANCE NEO 400 MHZ DIGITAL NMR SPECTROMETER
7	Author	
8	Solvent	CDCl3
9	Temperature	294.8
10	Pulse Sequence	zg30
11	Experiment	1D
12	Number of Scans	4
13	Receiver Gain	75
14	Relaxation Delay	1.0000
15	Pulse Width	10.0000
16	Acquisition Time	3.9977
17	Acquisition Date	2018-12-01T11:20:30
18	Modification Date	2018-12-21T21:16:31
19	Spectrometer Frequency	400.13
20	Spectral Width	8196.7
21	Lowest Frequency	-1664.0
22	Nucleus	1H
23	Acquired Size	32768
24	Spectral Size	65536

33a

Supplementary Figure 218. ^{1}H NMR spectrum of compound 33a

Parameter	Value
1 Title	SHW-22420-A
2 Comment	
3 Origin	Bruker BioSpin GmbH
4 Owner	nmrsu
5 Site	
6 Spectrometer	AVANCE NEO 400 MHZ DIGITAL NMR SPECTROMETER
7 Author	
8 Solvent	CDCl3
9 Temperature	295.1
10 Pulse Sequence	zgpg30
11 Experiment	1D
12 Number of Scans	35
13 Receiver Gain	32
14 Relaxation Delay	2.0000
15 Pulse Width	10.0000
16 Acquisition Time	1.3763
17 Acquisition Date	2018-12-01T11:23:29
18 Modification Date	2018-12-21T21:16:32
19 Spectrometer Frequency	100.61
20 Spectral Width	23809.5
21 Lowest Frequency	-1812.5
22 Nucleus	13C
23 Acquired Size	32768
24 Spectral Size	32768

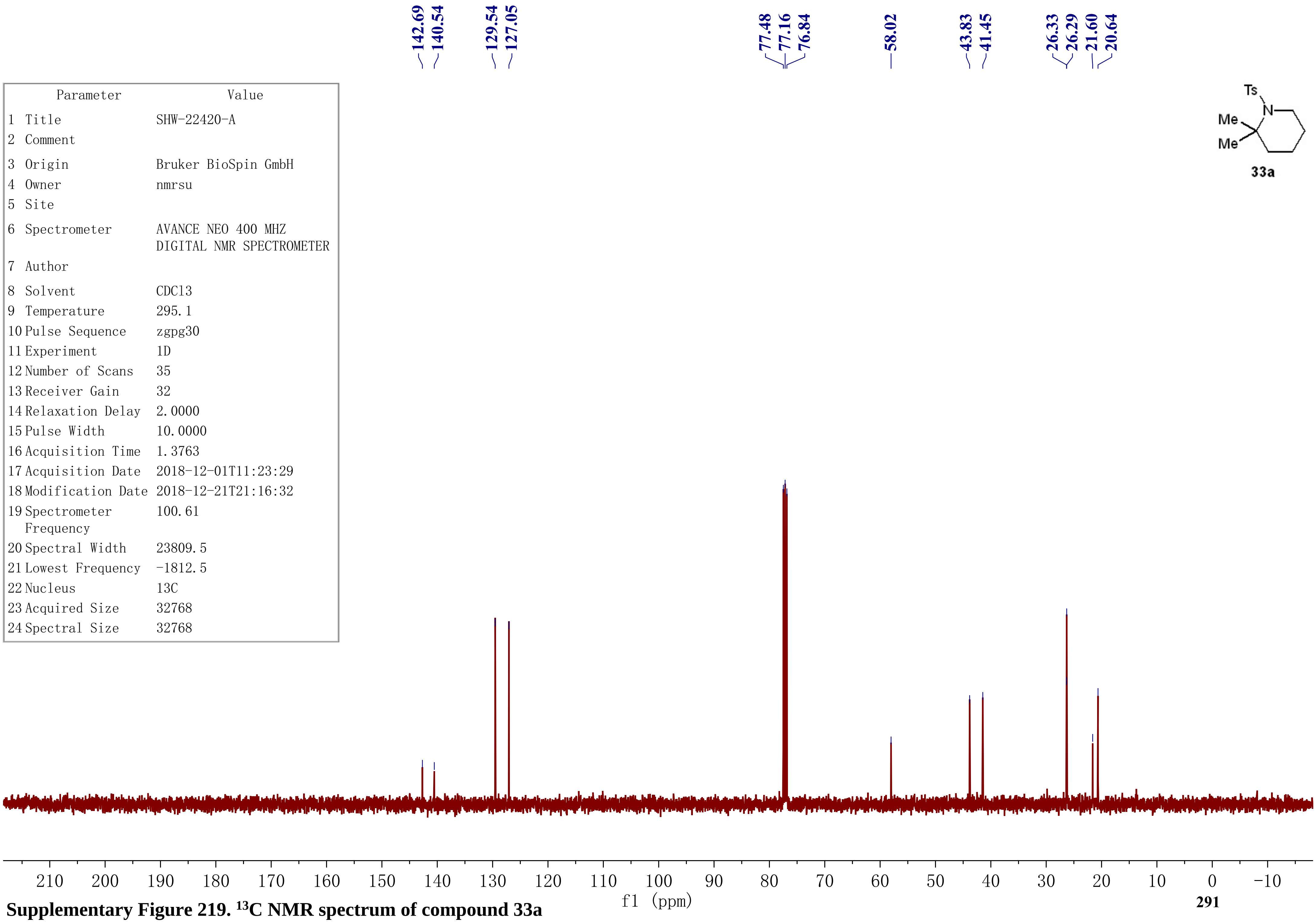

33a

Supplementary Figure 219. ^{13}C NMR spectrum of compound 33a

Parameter	Value
1 Title	SHW-22405-A
2 Comment	
3 Origin	Bruker BioSpin GmbH
4 Owner	nmrsu
5 Site	
6 Spectrometer	Avance NEO 600
7 Author	
8 Solvent	CDCl3
9 Temperature	297.4
10 Pulse Sequence	zg30
11 Experiment	1D
12 Number of Scans	16
13 Receiver Gain	67
14 Relaxation Delay	1.0000
15 Pulse Width	10.0000
16 Acquisition Time	2.7525
17 Acquisition Date	2019-01-14T22:02:18
18 Modification Date	2019-07-03T21:50:17
19 Spectrometer Frequency	600.15
20 Spectral Width	11904.8
21 Lowest Frequency	-2261.3
22 Nucleus	1H
23 Acquired Size	32768
24 Spectral Size	65536

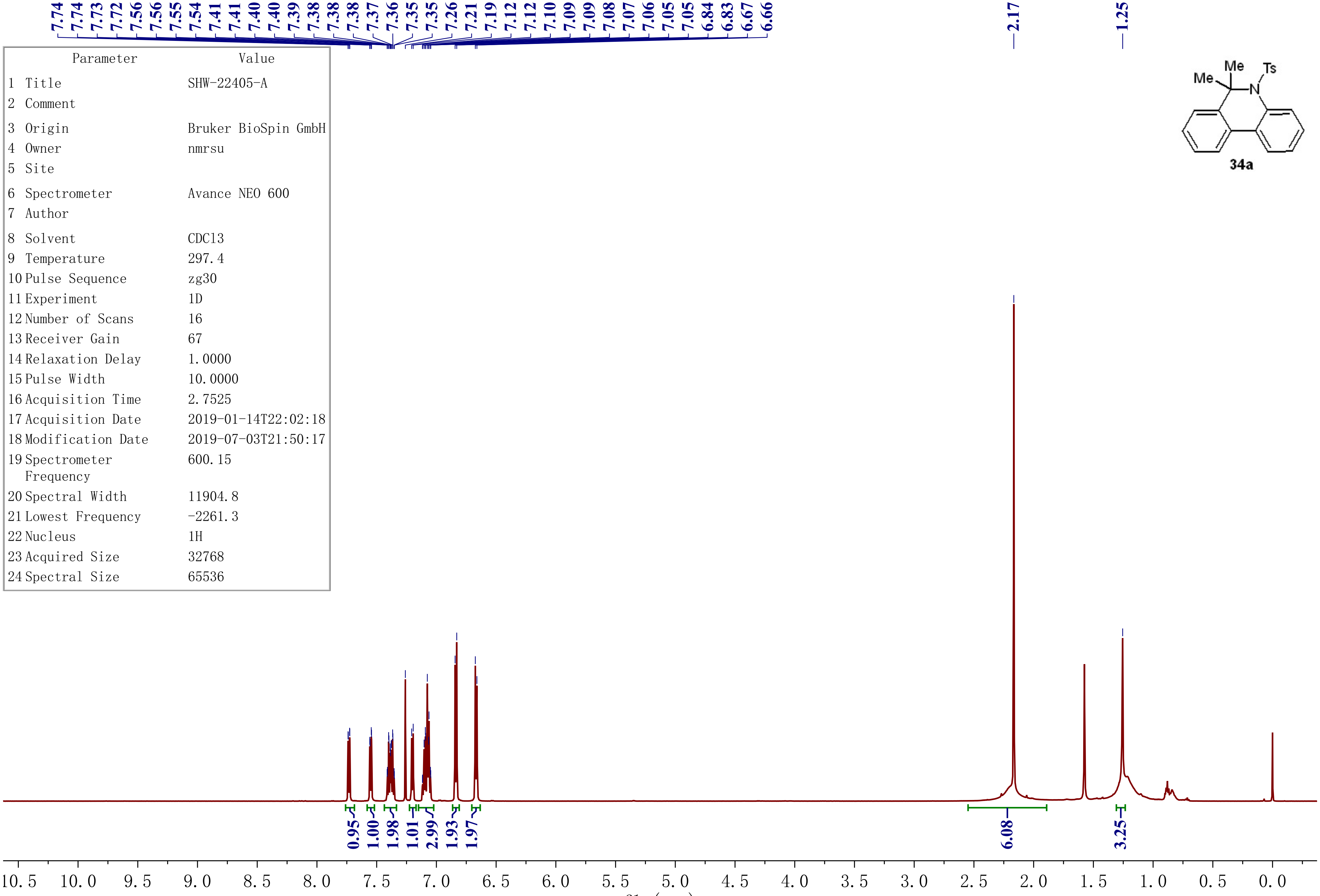

34a

Supplementary Figure 220. ^{1}H NMR spectrum of compound 34a

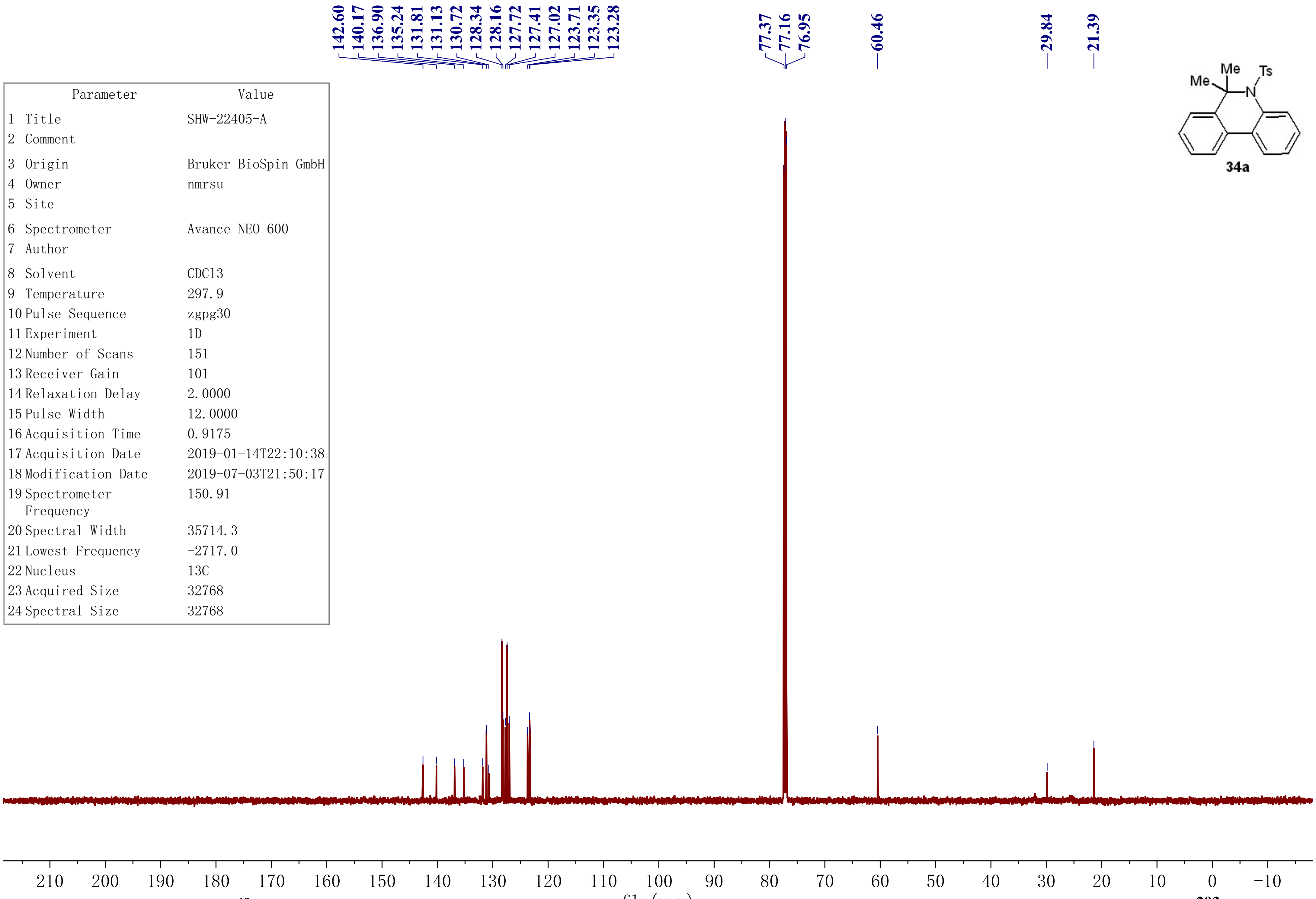

	Parameter	Value
1	Title	SHW-22405-A
2	Comment	
3	Origin	Bruker BioSpin GmbH
4	Owner	nmrsu
5	Site	
6	Spectrometer	Avance NEO 600
7	Author	
8	Solvent	CDCl3
9	Temperature	297.9
10	Pulse Sequence	zgpg30
11	Experiment	1D
12	Number of Scans	151
13	Receiver Gain	101
14	Relaxation Delay	2.0000
15	Pulse Width	12.0000
16	Acquisition Time	0.9175
17	Acquisition Date	2019-01-14T22:10:38
18	Modification Date	2019-07-03T21:50:17
19	Spectrometer Frequency	150.91
20	Spectral Width	35714.3
21	Lowest Frequency	-2717.0
22	Nucleus	13C
23	Acquired Size	32768
24	Spectral Size	32768

Supplementary Figure 221. ^{13}C NMR spectrum of compound 34a

Parameter	Value
1 Title	SHW-224125-2
2 Comment	
3 Origin	Bruker BioSpin GmbH
4 Owner	nmrsu
5 Site	
6 Spectrometer	Avance NEO 600
7 Author	
8 Solvent	CDCl3
9 Temperature	297.4
10 Pulse Sequence	zg30
11 Experiment	1D
12 Number of Scans	14
13 Receiver Gain	57
14 Relaxation Delay	1.0000
15 Pulse Width	10.0000
16 Acquisition Time	2.7525
17 Acquisition Date	2019-01-24T11:46:23
18 Modification Date	2019-01-24T12:07:26
19 Spectrometer Frequency	600.15
20 Spectral Width	11904.8
21 Lowest Frequency	-2262.4
22 Nucleus	1H
23 Acquired Size	32768
24 Spectral Size	65536

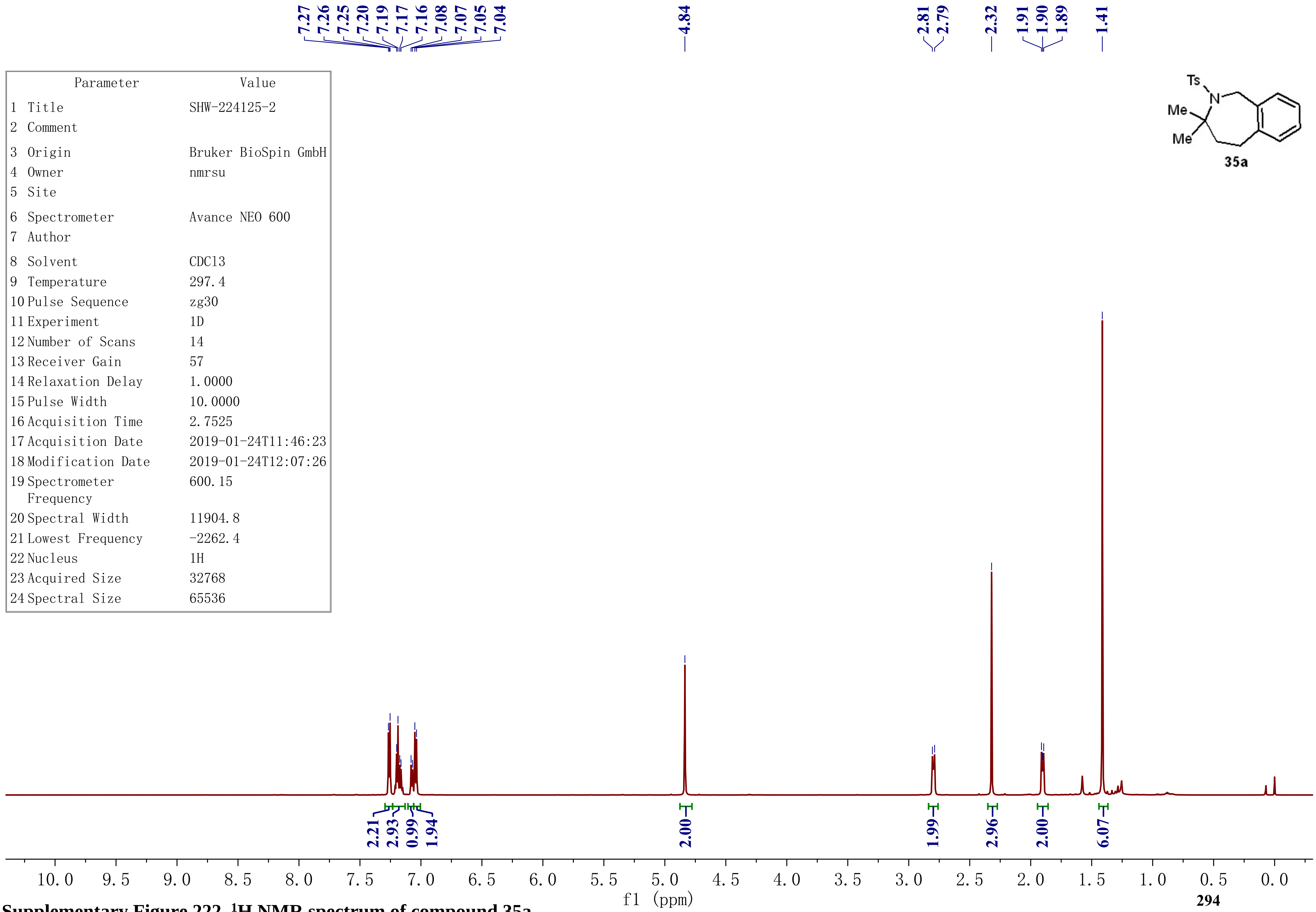

35a

Supplementary Figure 222. ^{1}H NMR spectrum of compound 35a

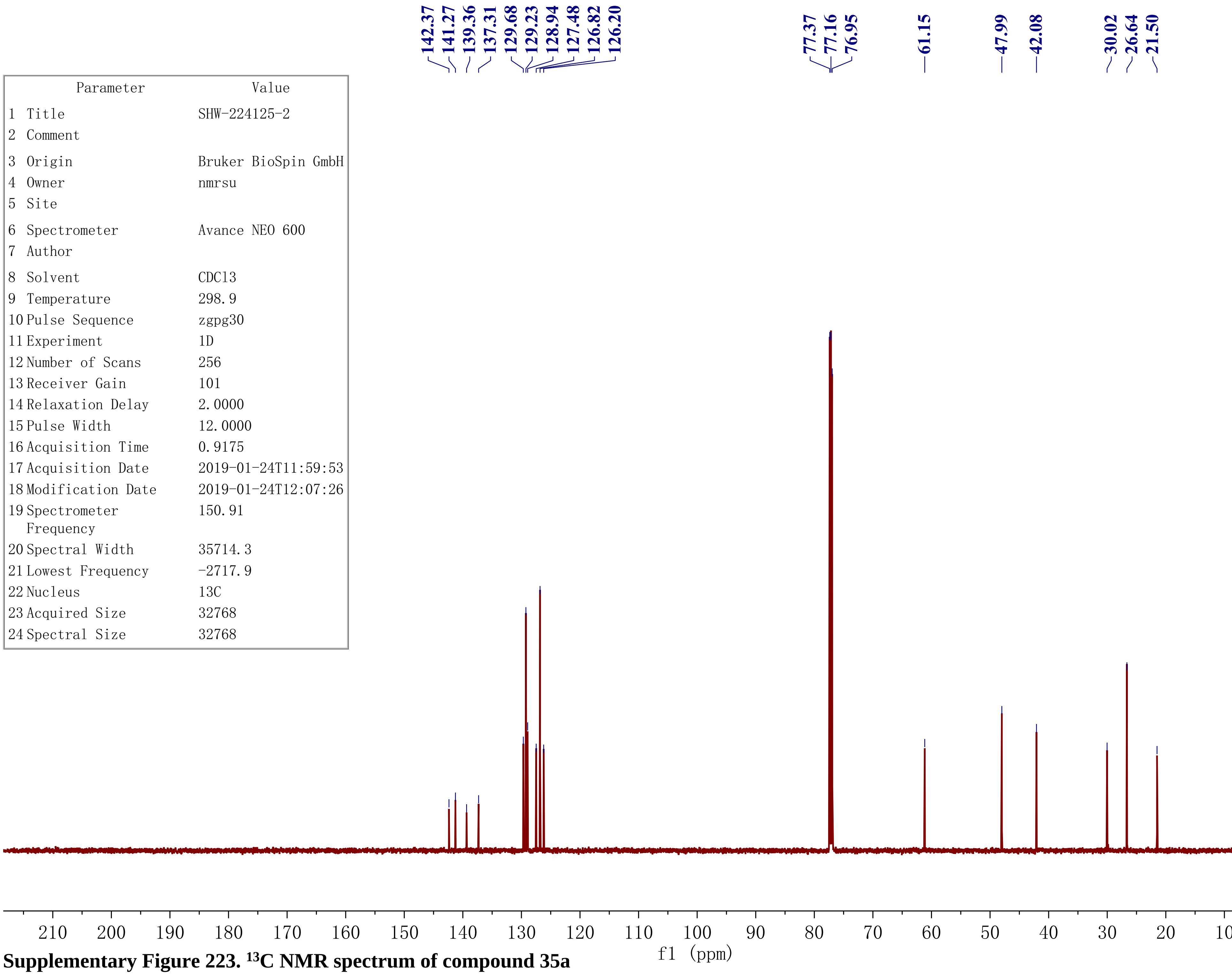

	Parameter	Value
1	Title	SHW-224125-2
2	Comment	
3	Origin	Bruker BioSpin GmbH
4	Owner	nmrsu
5	Site	
6	Spectrometer	Avance NEO 600
7	Author	
8	Solvent	CDCl3
9	Temperature	298.9
10	Pulse Sequence	zgpg30
11	Experiment	1D
12	Number of Scans	256
13	Receiver Gain	101
14	Relaxation Delay	2.0000
15	Pulse Width	12.0000
16	Acquisition Time	0.9175
17	Acquisition Date	2019-01-24T11:59:53
18	Modification Date	2019-01-24T12:07:26
19	Spectrometer Frequency	150.91
20	Spectral Width	35714.3
21	Lowest Frequency	-2717.9
22	Nucleus	13C
23	Acquired Size	32768
24	Spectral Size	32768

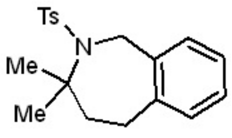

35a

Supplementary Figure 223. ^{13}**C NMR spectrum of compound 35a**

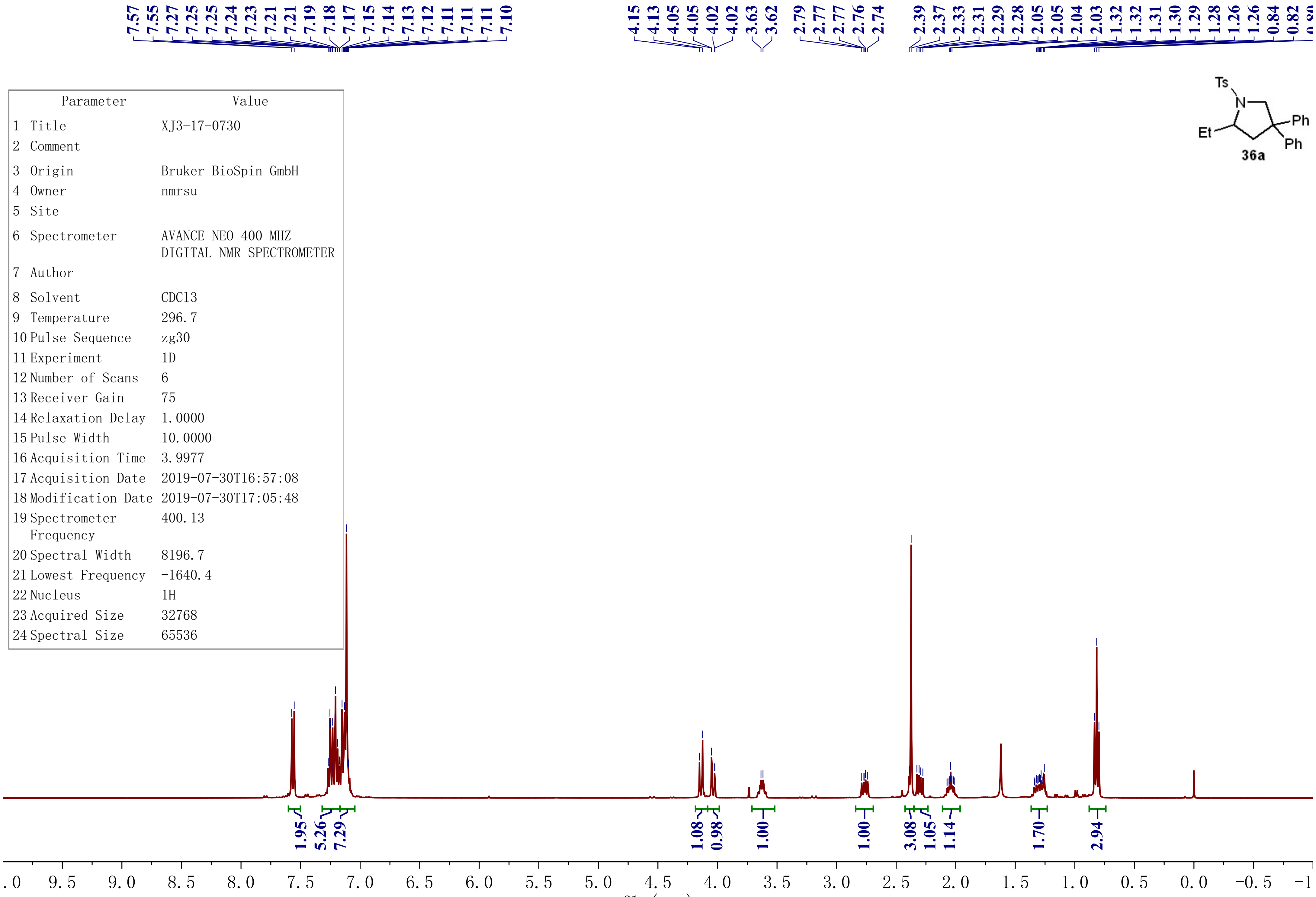

Parameter	Value
1 Title	XJ3-17-0730
2 Comment	
3 Origin	Bruker BioSpin GmbH
4 Owner	nmrsu
5 Site	
6 Spectrometer	AVANCE NEO 400 MHZ DIGITAL NMR SPECTROMETER
7 Author	
8 Solvent	CDCl3
9 Temperature	296.7
10 Pulse Sequence	zg30
11 Experiment	1D
12 Number of Scans	6
13 Receiver Gain	75
14 Relaxation Delay	1.0000
15 Pulse Width	10.0000
16 Acquisition Time	3.9977
17 Acquisition Date	2019-07-30T16:57:08
18 Modification Date	2019-07-30T17:05:48
19 Spectrometer Frequency	400.13
20 Spectral Width	8196.7
21 Lowest Frequency	-1640.4
22 Nucleus	1H
23 Acquired Size	32768
24 Spectral Size	65536

36a

Supplementary Figure 224. ^{1}H NMR spectrum of compound 36a

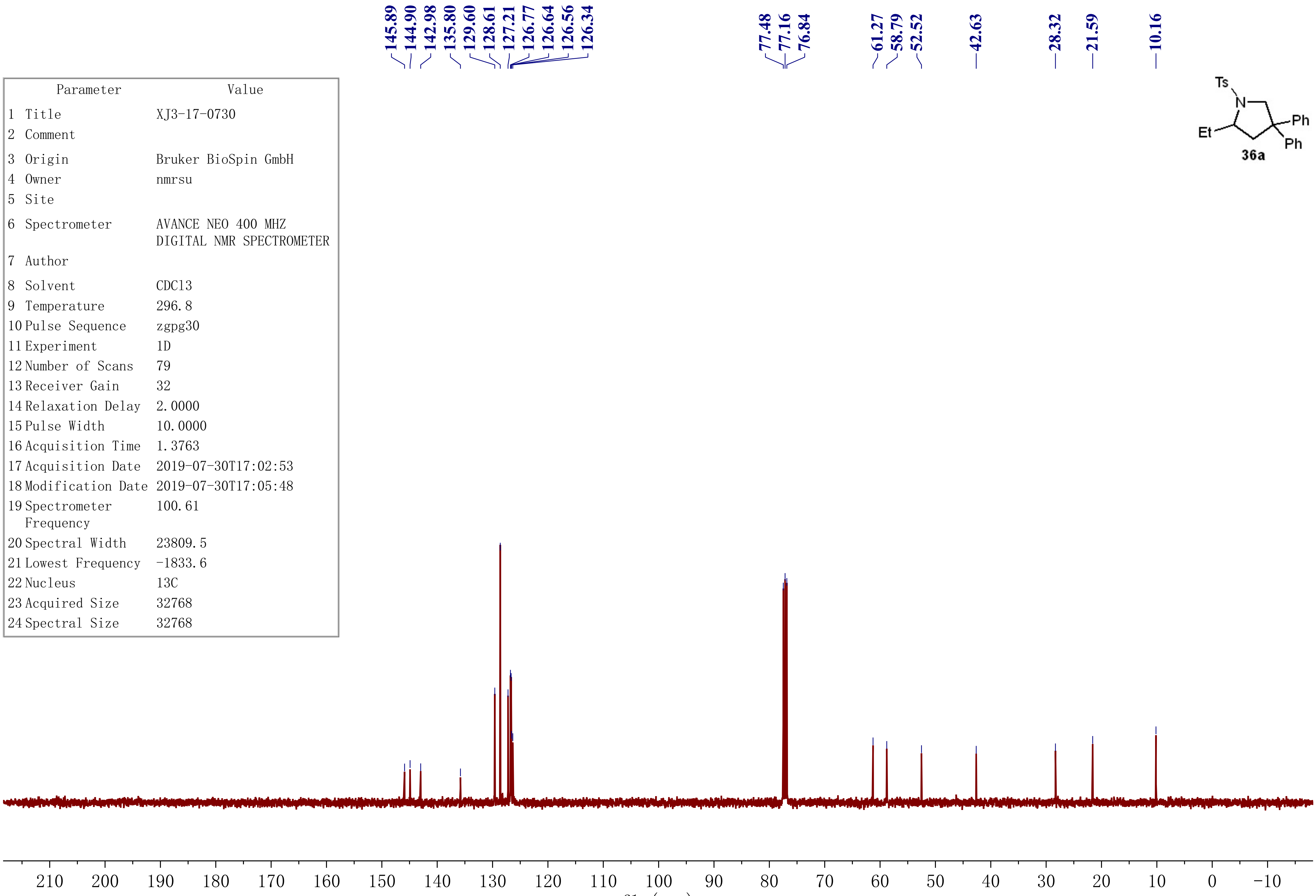

	Parameter	Value
1	Title	XJ3-17-0730
2	Comment	
3	Origin	Bruker BioSpin GmbH
4	Owner	nmrsu
5	Site	
6	Spectrometer	AVANCE NEO 400 MHZ DIGITAL NMR SPECTROMETER
7	Author	
8	Solvent	CDCl3
9	Temperature	296.8
10	Pulse Sequence	zgpg30
11	Experiment	1D
12	Number of Scans	79
13	Receiver Gain	32
14	Relaxation Delay	2.0000
15	Pulse Width	10.0000
16	Acquisition Time	1.3763
17	Acquisition Date	2019-07-30T17:02:53
18	Modification Date	2019-07-30T17:05:48
19	Spectrometer Frequency	100.61
20	Spectral Width	23809.5
21	Lowest Frequency	-1833.6
22	Nucleus	13C
23	Acquired Size	32768
24	Spectral Size	32768

Supplementary Figure 225. ^{13}C NMR spectrum of compound 36a

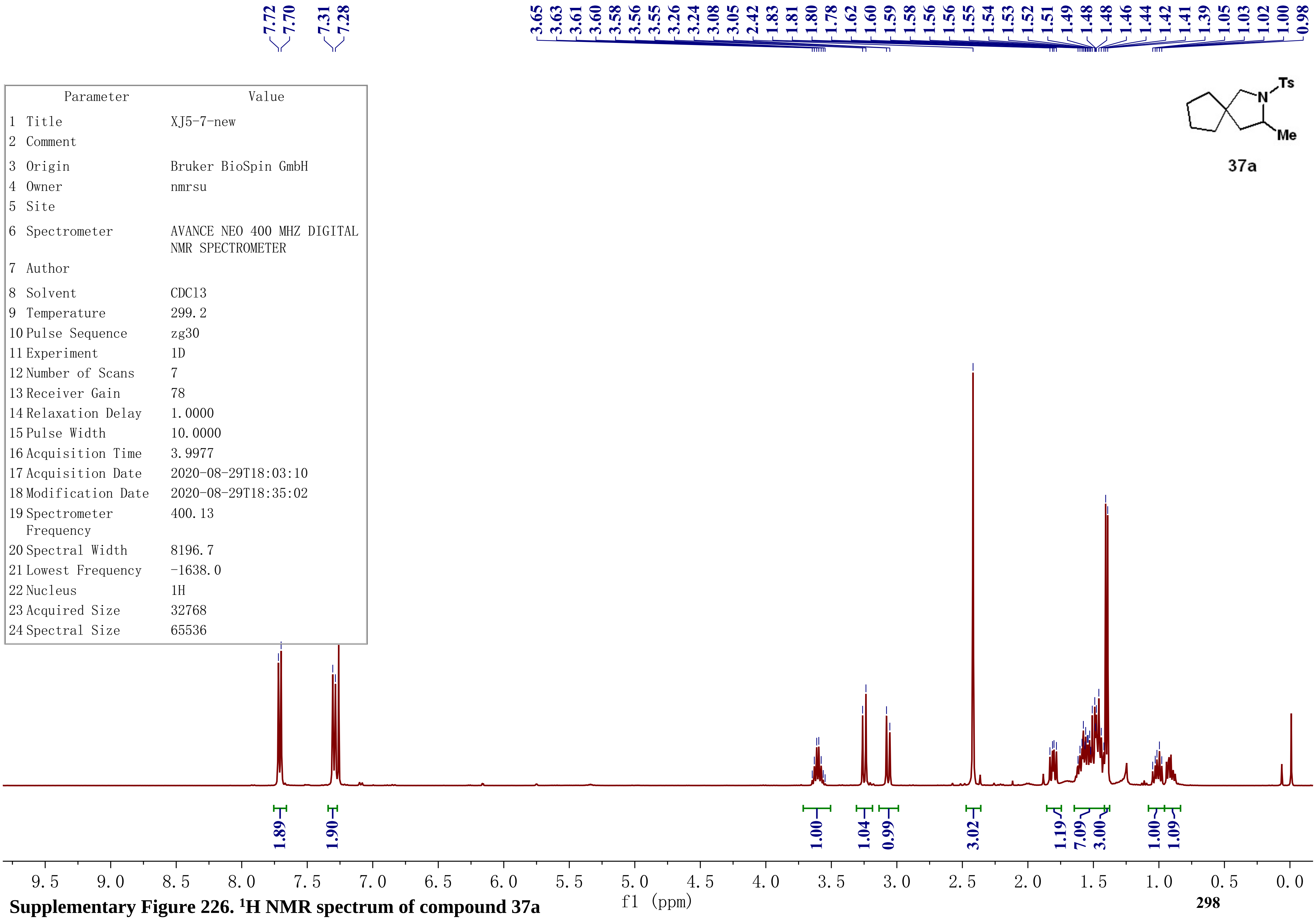

Parameter		Value
1	Title	XJ5-7-new
2	Comment	
3	Origin	Bruker BioSpin GmbH
4	Owner	nmrsu
5	Site	
6	Spectrometer	AVANCE NEO 400 MHZ DIGITAL NMR SPECTROMETER
7	Author	
8	Solvent	CDCl3
9	Temperature	299.2
10	Pulse Sequence	zg30
11	Experiment	1D
12	Number of Scans	7
13	Receiver Gain	78
14	Relaxation Delay	1.0000
15	Pulse Width	10.0000
16	Acquisition Time	3.9977
17	Acquisition Date	2020-08-29T18:03:10
18	Modification Date	2020-08-29T18:35:02
19	Spectrometer Frequency	400.13
20	Spectral Width	8196.7
21	Lowest Frequency	-1638.0
22	Nucleus	1H
23	Acquired Size	32768
24	Spectral Size	65536

37a

Supplementary Figure 226. ^{1}H NMR spectrum of compound 37a

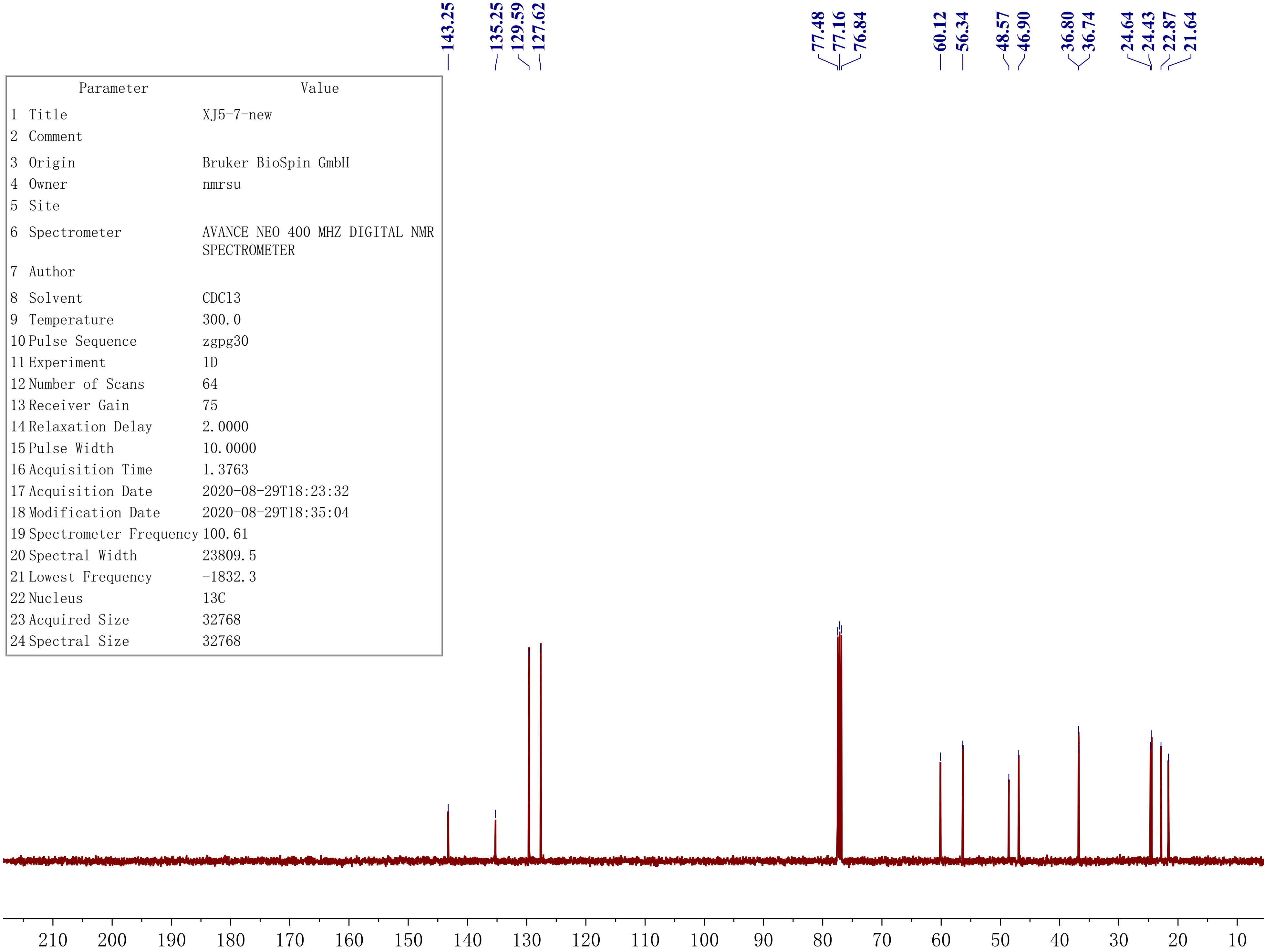

	Parameter	Value
1	Title	XJ5-7-new
2	Comment	
3	Origin	Bruker BioSpin GmbH
4	Owner	nmrsu
5	Site	
6	Spectrometer	AVANCE NEO 400 MHZ DIGITAL NMR SPECTROMETER
7	Author	
8	Solvent	CDCl3
9	Temperature	300.0
10	Pulse Sequence	zgpg30
11	Experiment	1D
12	Number of Scans	64
13	Receiver Gain	75
14	Relaxation Delay	2.0000
15	Pulse Width	10.0000
16	Acquisition Time	1.3763
17	Acquisition Date	2020-08-29T18:23:32
18	Modification Date	2020-08-29T18:35:04
19	Spectrometer Frequency	100.61
20	Spectral Width	23809.5
21	Lowest Frequency	-1832.3
22	Nucleus	13C
23	Acquired Size	32768
24	Spectral Size	32768

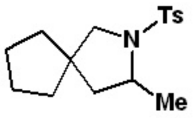

37a

Supplementary Figure 227. ^{13}C NMR spectrum of compound 37a

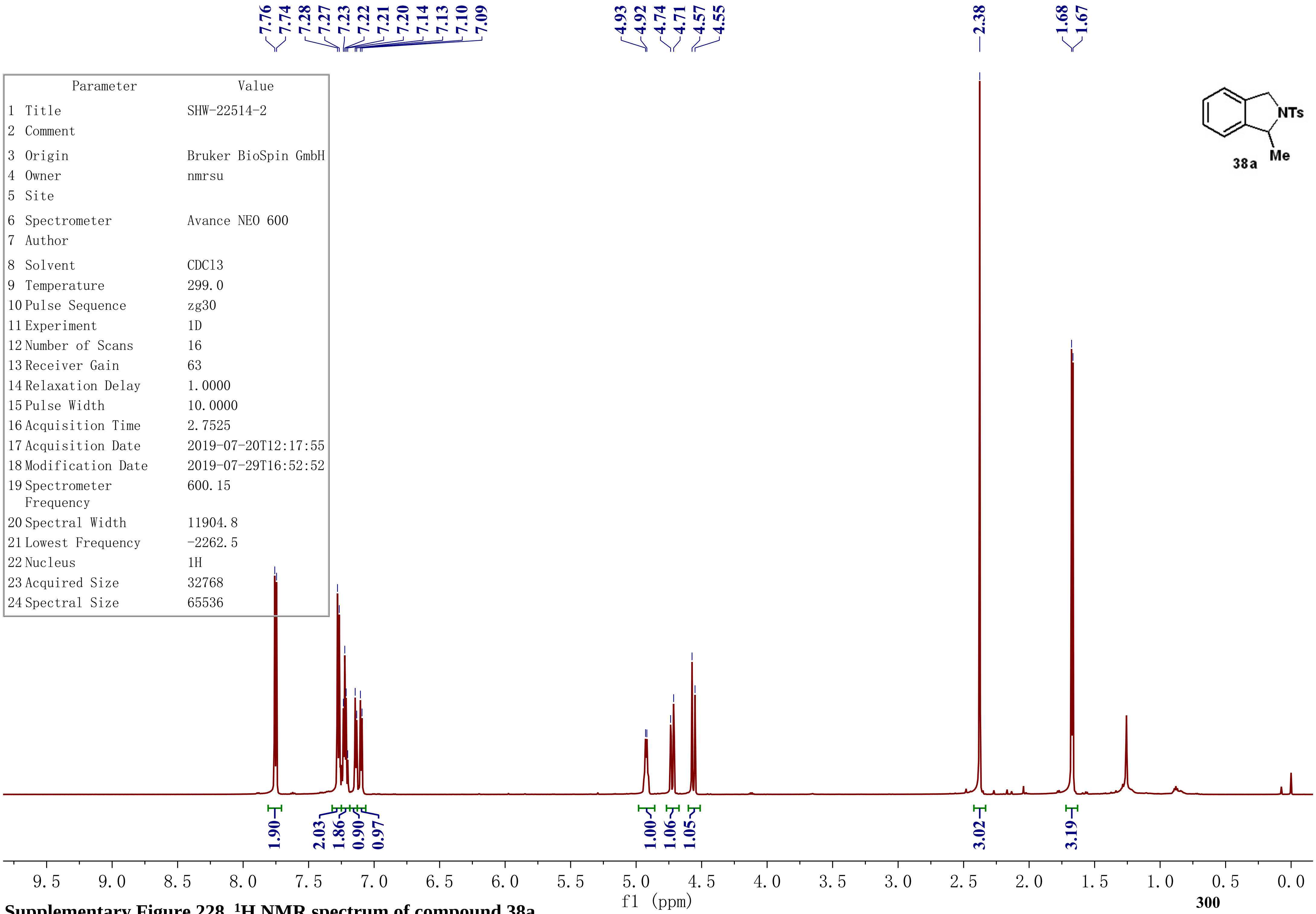

Supplementary Figure 228. ^{1}H NMR spectrum of compound 38a

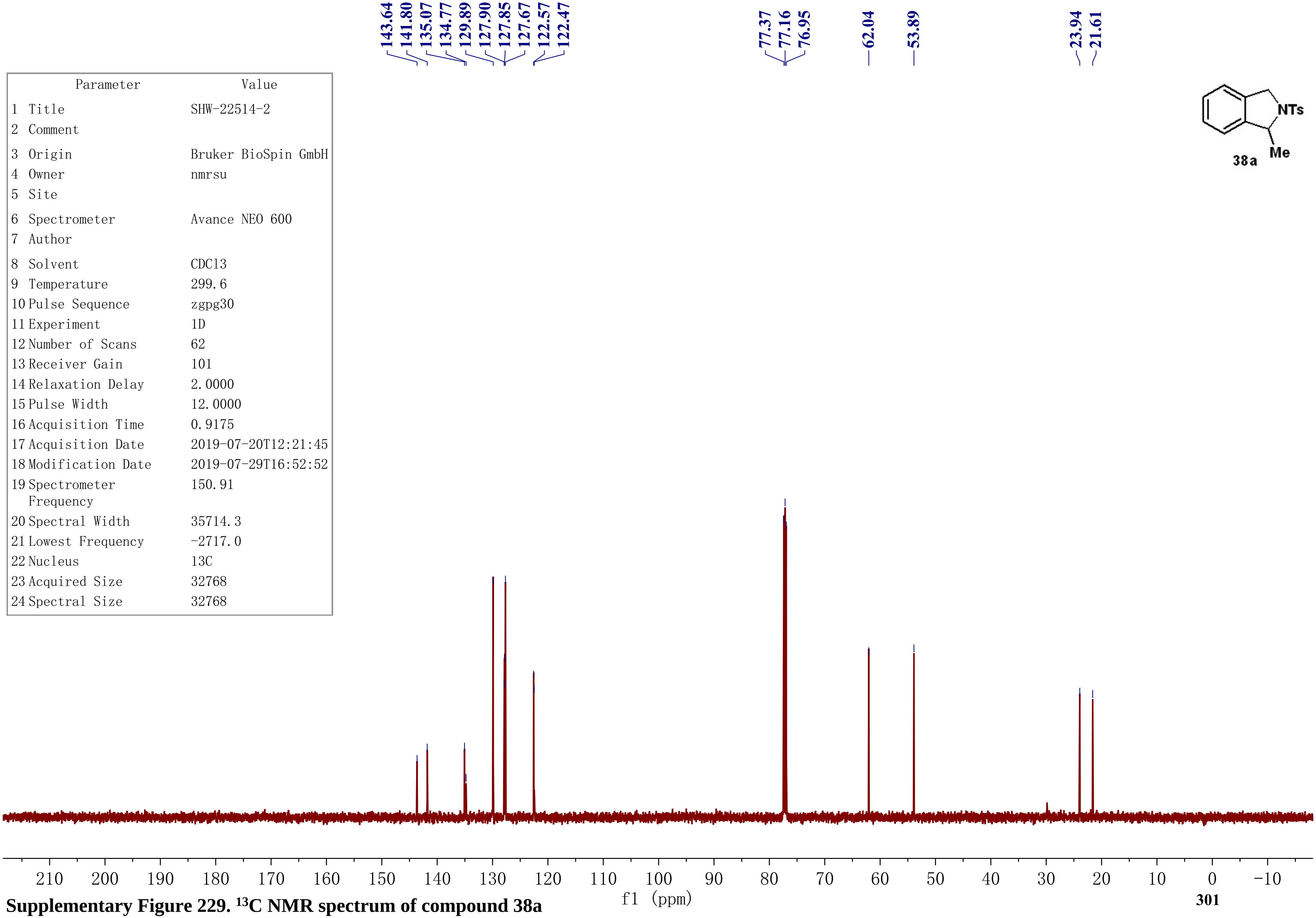

Parameter		Value
1	Title	SHW-22514-2
2	Comment	
3	Origin	Bruker BioSpin GmbH
4	Owner	nmrsu
5	Site	
6	Spectrometer	Avance NEO 600
7	Author	
8	Solvent	CDCl3
9	Temperature	299.6
10	Pulse Sequence	zgpg30
11	Experiment	1D
12	Number of Scans	62
13	Receiver Gain	101
14	Relaxation Delay	2.0000
15	Pulse Width	12.0000
16	Acquisition Time	0.9175
17	Acquisition Date	2019-07-20T12:21:45
18	Modification Date	2019-07-29T16:52:52
19	Spectrometer Frequency	150.91
20	Spectral Width	35714.3
21	Lowest Frequency	-2717.0
22	Nucleus	13C
23	Acquired Size	32768
24	Spectral Size	32768

38a

Supplementary Figure 229. ^{13}C **NMR spectrum of compound 38a**

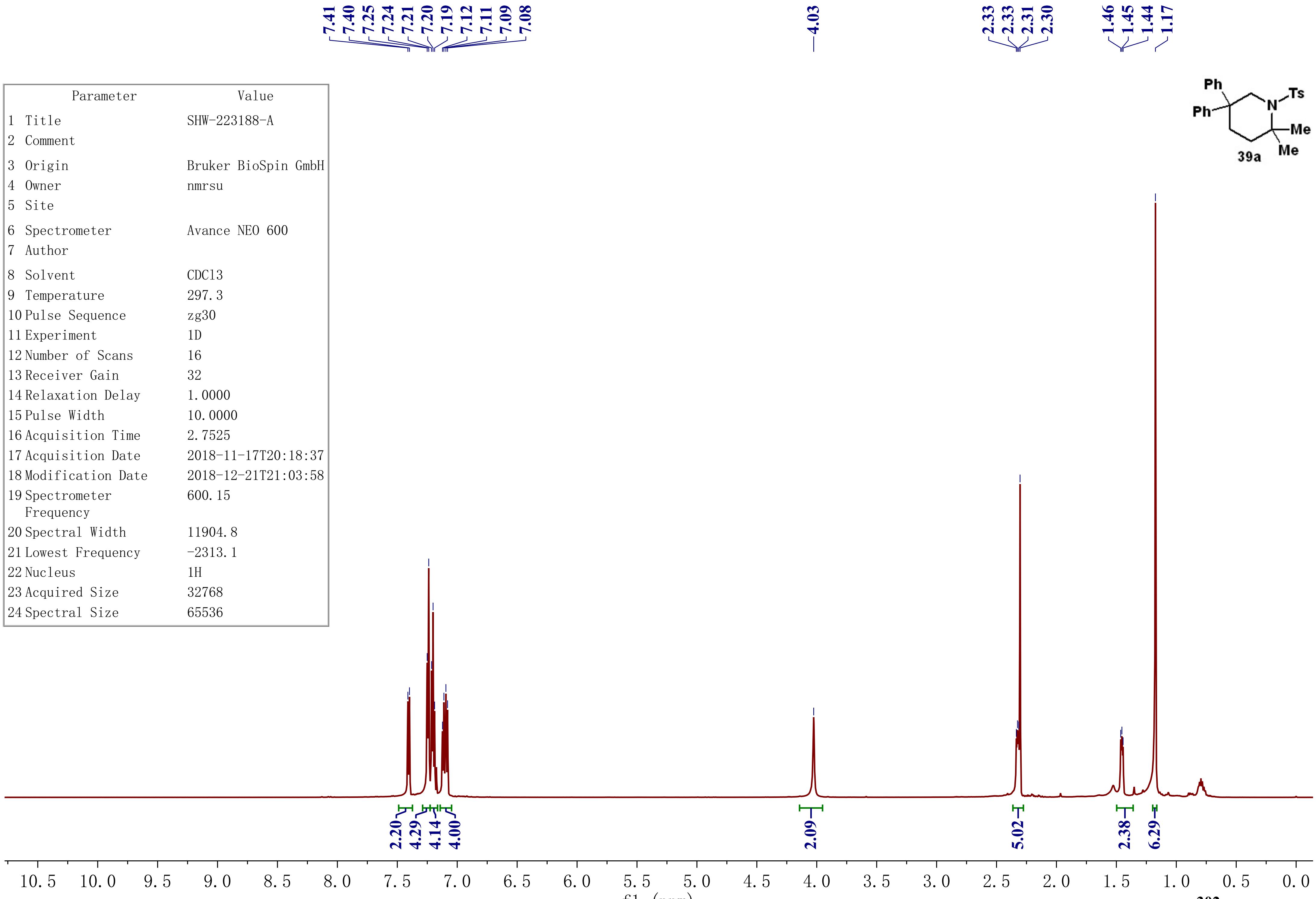

	Parameter	Value
1	Title	SHW-223188-A
2	Comment	
3	Origin	Bruker BioSpin GmbH
4	Owner	nmrsu
5	Site	
6	Spectrometer	Avance NEO 600
7	Author	
8	Solvent	CDCl3
9	Temperature	297.3
10	Pulse Sequence	zg30
11	Experiment	1D
12	Number of Scans	16
13	Receiver Gain	32
14	Relaxation Delay	1.0000
15	Pulse Width	10.0000
16	Acquisition Time	2.7525
17	Acquisition Date	2018-11-17T20:18:37
18	Modification Date	2018-12-21T21:03:58
19	Spectrometer Frequency	600.15
20	Spectral Width	11904.8
21	Lowest Frequency	-2313.1
22	Nucleus	1H
23	Acquired Size	32768
24	Spectral Size	65536

Supplementary Figure 230. ^{1}H NMR spectrum of compound 39a

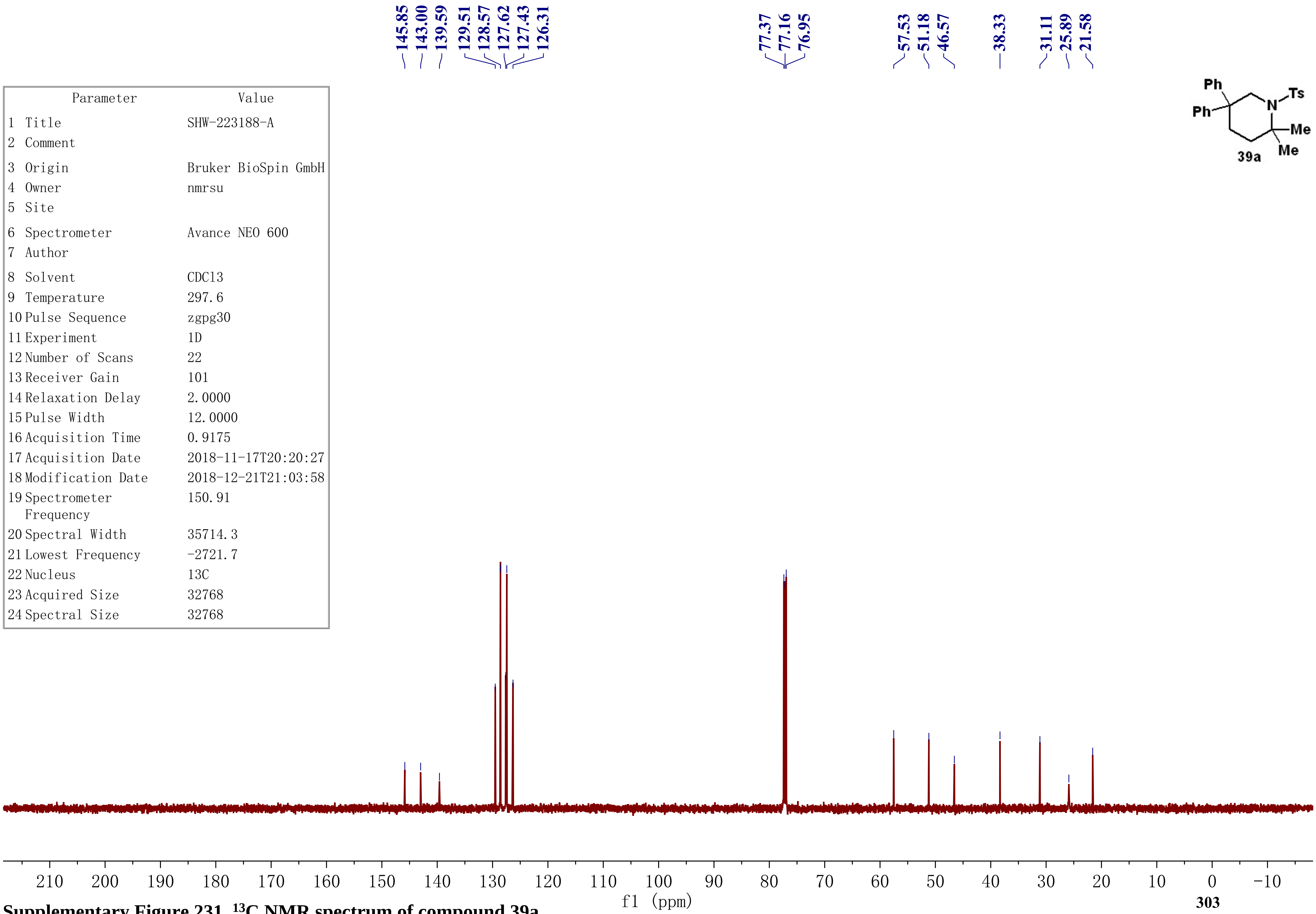

Parameter	Value
1 Title	SHW-223188-A
2 Comment	
3 Origin	Bruker BioSpin GmbH
4 Owner	nmrsu
5 Site	
6 Spectrometer	Avance NEO 600
7 Author	
8 Solvent	CDCl3
9 Temperature	297.6
10 Pulse Sequence	zgpg30
11 Experiment	1D
12 Number of Scans	22
13 Receiver Gain	101
14 Relaxation Delay	2.0000
15 Pulse Width	12.0000
16 Acquisition Time	0.9175
17 Acquisition Date	2018-11-17T20:20:27
18 Modification Date	2018-12-21T21:03:58
19 Spectrometer Frequency	150.91
20 Spectral Width	35714.3
21 Lowest Frequency	-2721.7
22 Nucleus	13C
23 Acquired Size	32768
24 Spectral Size	32768

Supplementary Figure 231. ^{13}C NMR spectrum of compound 39a

Parameter	Value
1 Title	SHW-22465-P
2 Comment	
3 Origin	Bruker BioSpin GmbH
4 Owner	nmrsu
5 Site	
6 Spectrometer	AVANCE NEO 400 MHZ DIGITAL NMR SPECTROMETER
7 Author	
8 Solvent	CDCl3
9 Temperature	295.0
10 Pulse Sequence	zg30
11 Experiment	1D
12 Number of Scans	6
13 Receiver Gain	75
14 Relaxation Delay	1.0000
15 Pulse Width	10.0000
16 Acquisition Time	3.9977
17 Acquisition Date	2018-12-26T20:08:02
18 Modification Date	2018-12-26T20:34:34
19 Spectrometer Frequency	400.13
20 Spectral Width	8196.7
21 Lowest Frequency	-1635.4
22 Nucleus	1H
23 Acquired Size	32768
24 Spectral Size	65536

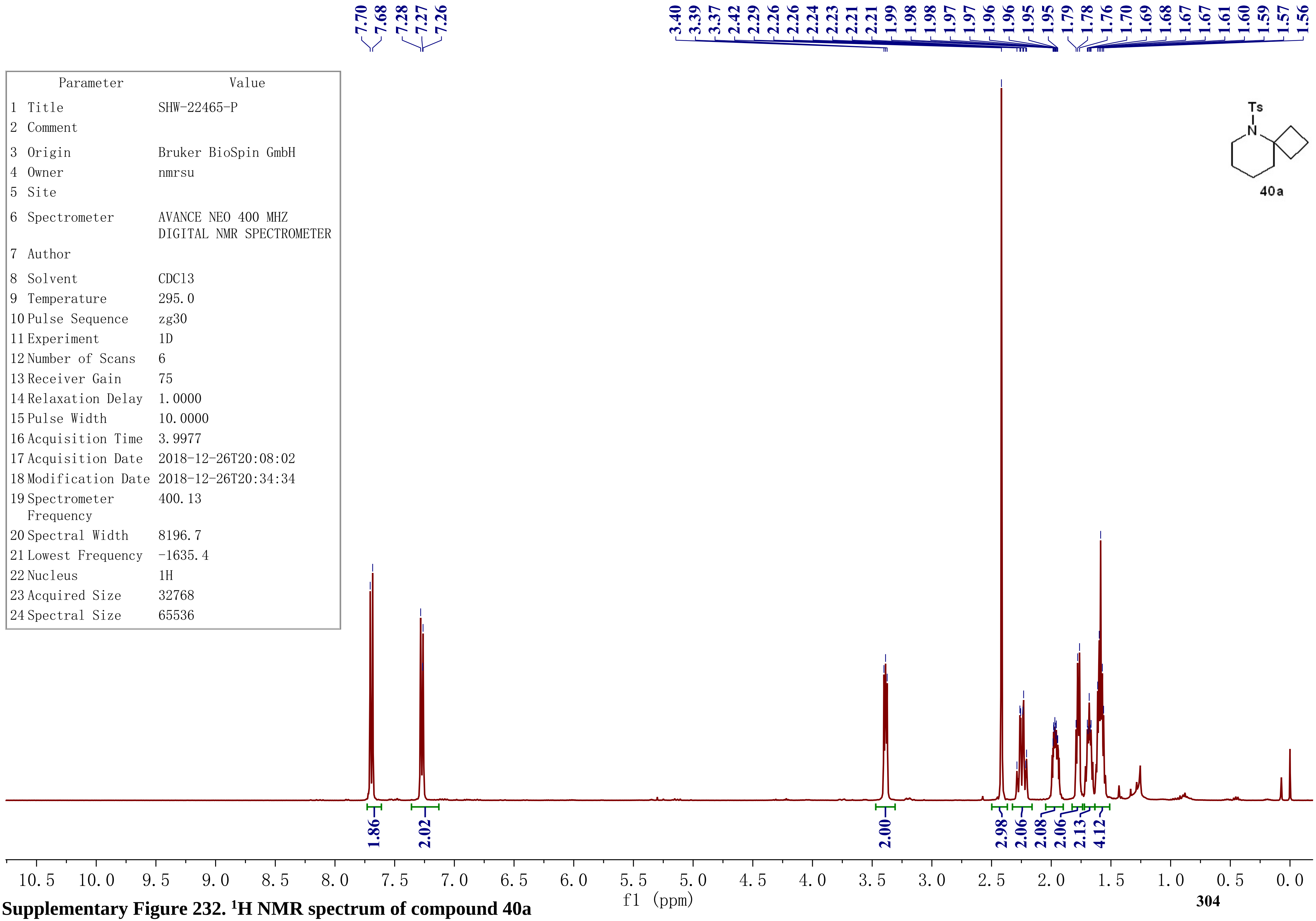

Supplementary Figure 232. ^{1}H NMR spectrum of compound 40a

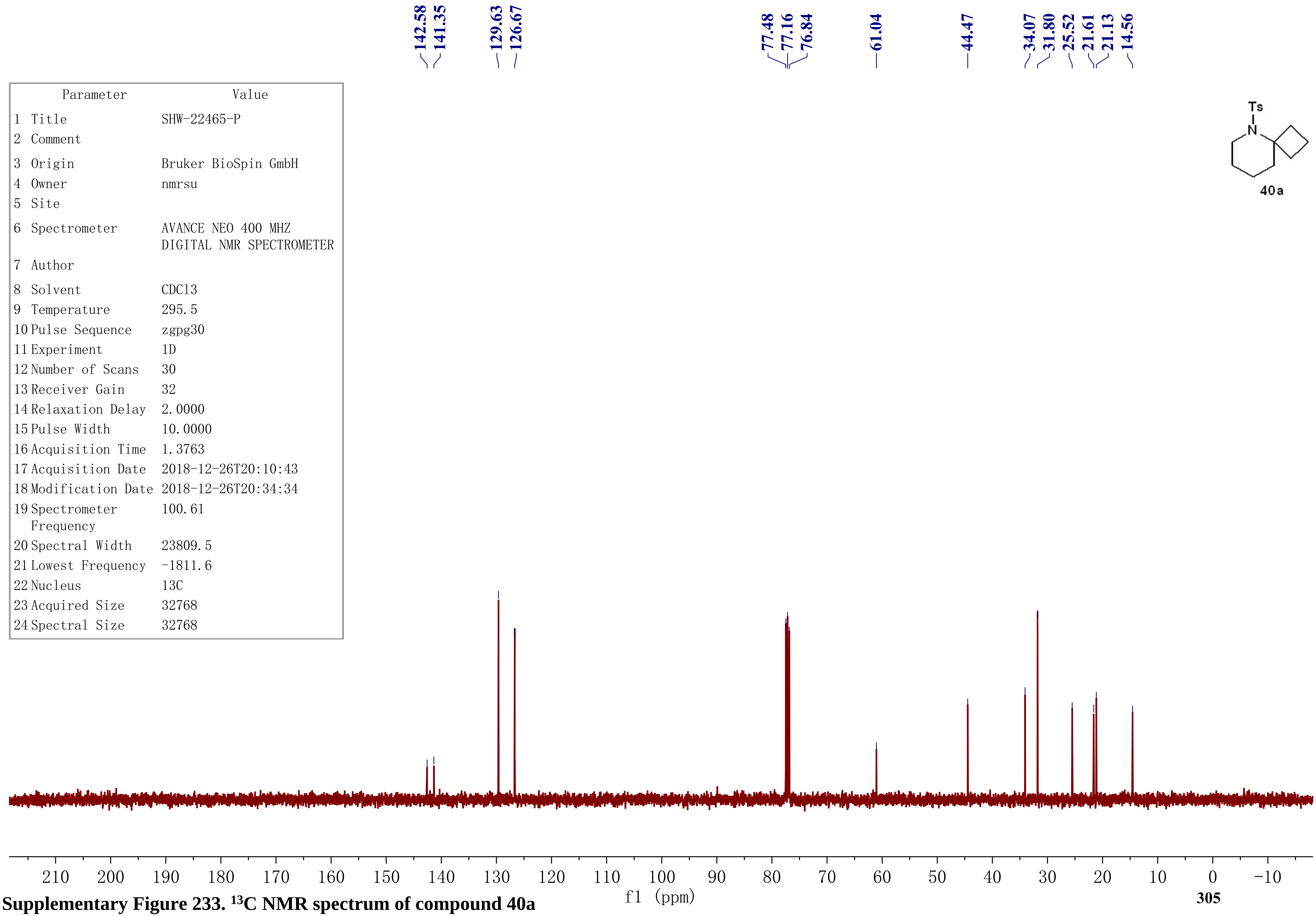

	Parameter	Value
1	Title	SHW-22465-P
2	Comment	
3	Origin	Bruker BioSpin GmbH
4	Owner	nmrsu
5	Site	
6	Spectrometer	AVANCE NEO 400 MHZ DIGITAL NMR SPECTROMETER
7	Author	
8	Solvent	CDCl3
9	Temperature	295.5
10	Pulse Sequence	zgpg30
11	Experiment	1D
12	Number of Scans	30
13	Receiver Gain	32
14	Relaxation Delay	2.0000
15	Pulse Width	10.0000
16	Acquisition Time	1.3763
17	Acquisition Date	2018-12-26T20:10:43
18	Modification Date	2018-12-26T20:34:34
19	Spectrometer Frequency	100.61
20	Spectral Width	23809.5
21	Lowest Frequency	-1811.6
22	Nucleus	13C
23	Acquired Size	32768
24	Spectral Size	32768

40a

Supplementary Figure 233. ^{13}C NMR spectrum of compound 40a

Parameter	Value
1 Title	SHW-224196-1
2 Comment	
3 Origin	Bruker BioSpin GmbH
4 Owner	nmrsu
5 Site	
6 Spectrometer	Avance NEO 600
7 Author	
8 Solvent	CDCl3
9 Temperature	297.5
10 Pulse Sequence	zg30
11 Experiment	1D
12 Number of Scans	4
13 Receiver Gain	17
14 Relaxation Delay	1.0000
15 Pulse Width	10.0000
16 Acquisition Time	2.7525
17 Acquisition Date	2019-04-11T12:32:46
18 Modification Date	2019-04-11T13:28:01
19 Spectrometer Frequency	600.15
20 Spectral Width	11904.8
21 Lowest Frequency	-2379.9
22 Nucleus	1H
23 Acquired Size	32768
24 Spectral Size	65536

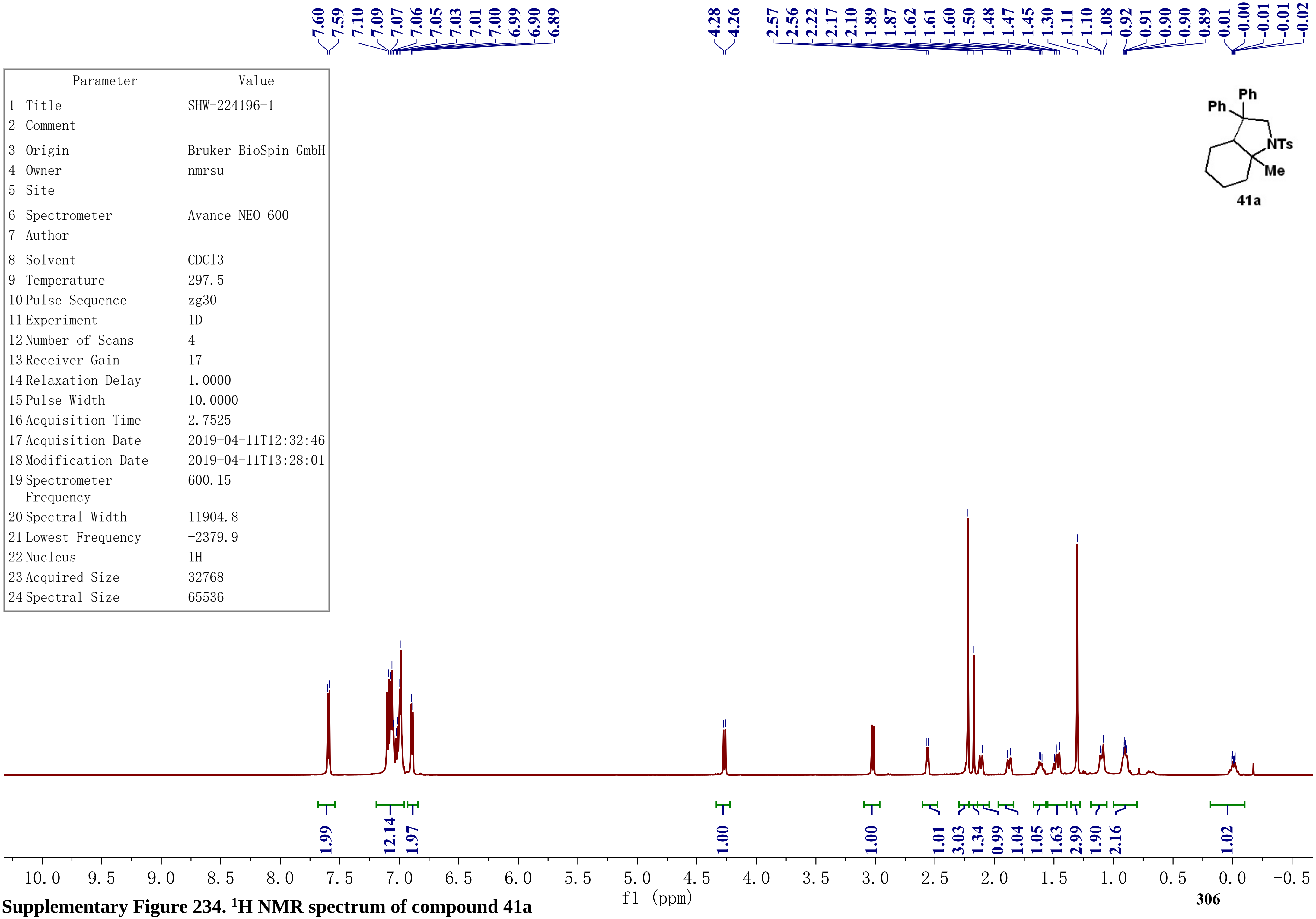

Supplementary Figure 234. ^1H NMR spectrum of compound 41a

Parameter	Value
1 Title	SHW-224196-C
2 Comment	
3 Origin	Bruker BioSpin GmbH
4 Owner	nmrsu
5 Site	
6 Spectrometer	AVANCE NEO 400 MHZ DIGITAL NMR SPECTROMETER
7 Author	
8 Solvent	CDCl3
9 Temperature	297.9
10 Pulse Sequence	zgpg30
11 Experiment	1D
12 Number of Scans	33
13 Receiver Gain	35
14 Relaxation Delay	2.0000
15 Pulse Width	10.0000
16 Acquisition Time	1.3763
17 Acquisition Date	2019-07-29T19:07:21
18 Modification Date	2019-07-29T19:14:17
19 Spectrometer Frequency	100.61
20 Spectral Width	23809.5
21 Lowest Frequency	-1813.8
22 Nucleus	13C
23 Acquired Size	32768
24 Spectral Size	32768

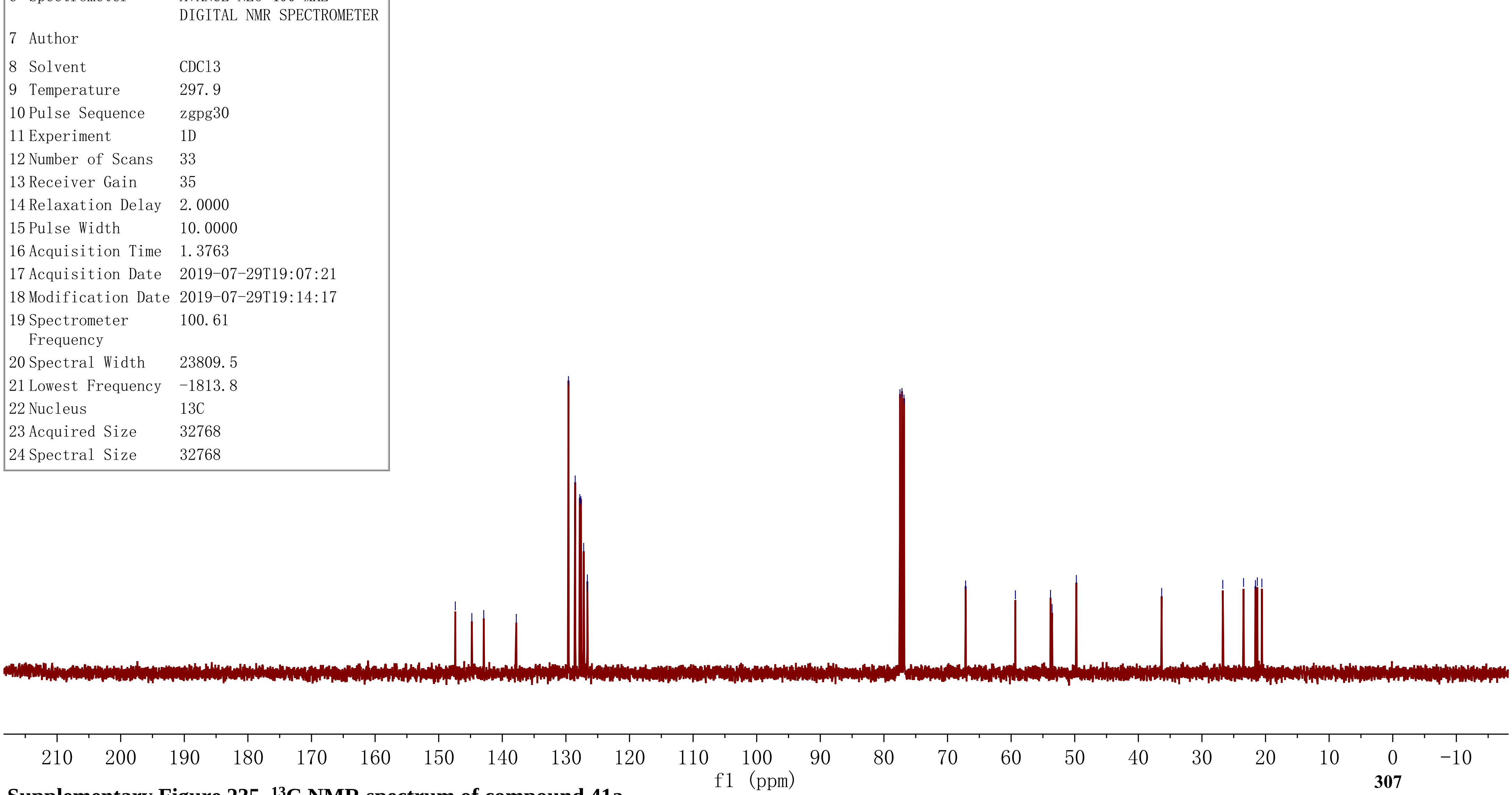

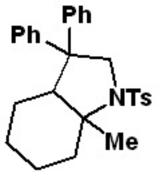

41a

Supplementary Figure 235. ^{13}C NMR spectrum of compound 41a

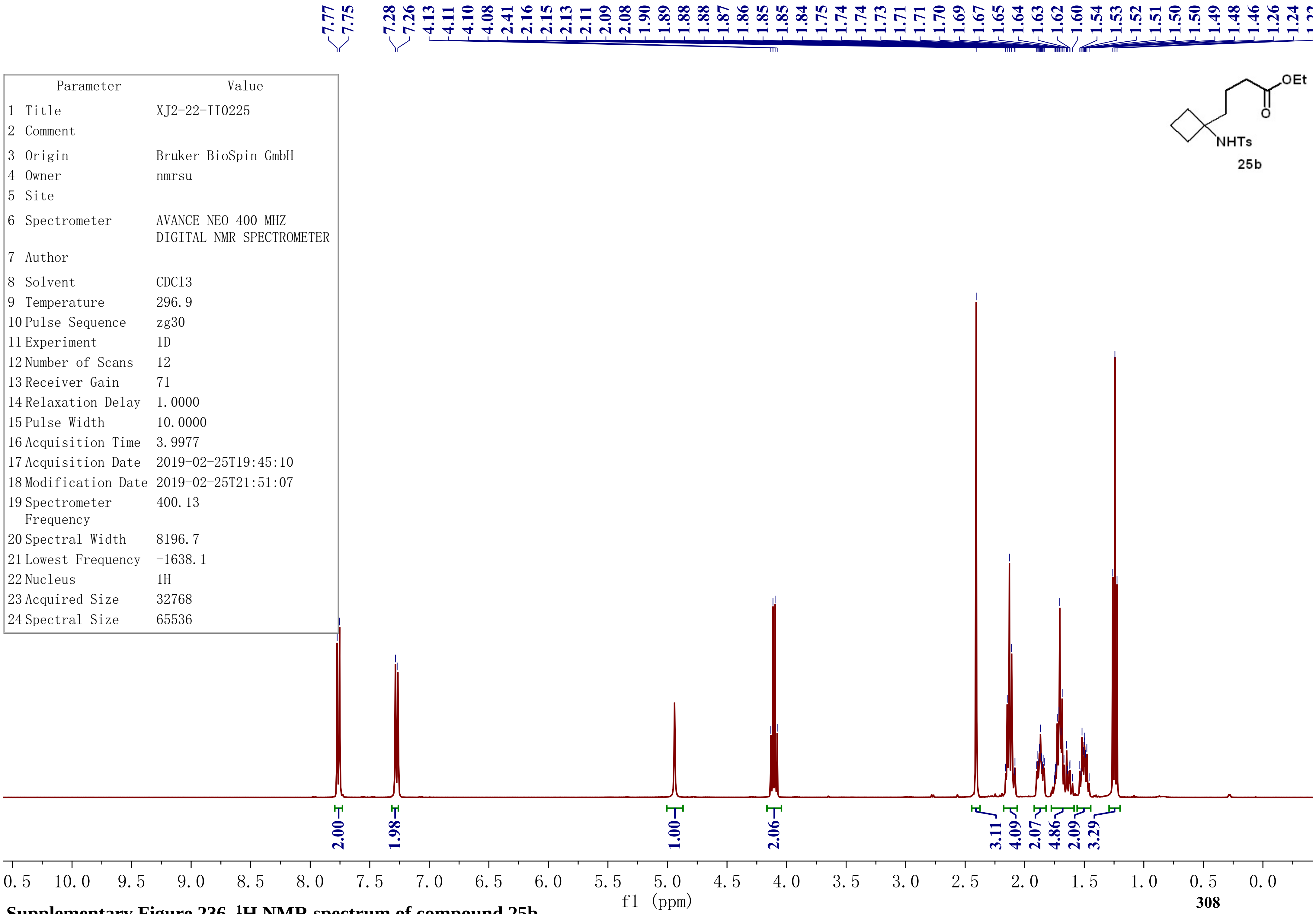

Supplementary Figure 236. ^{1}H NMR spectrum of compound 25b

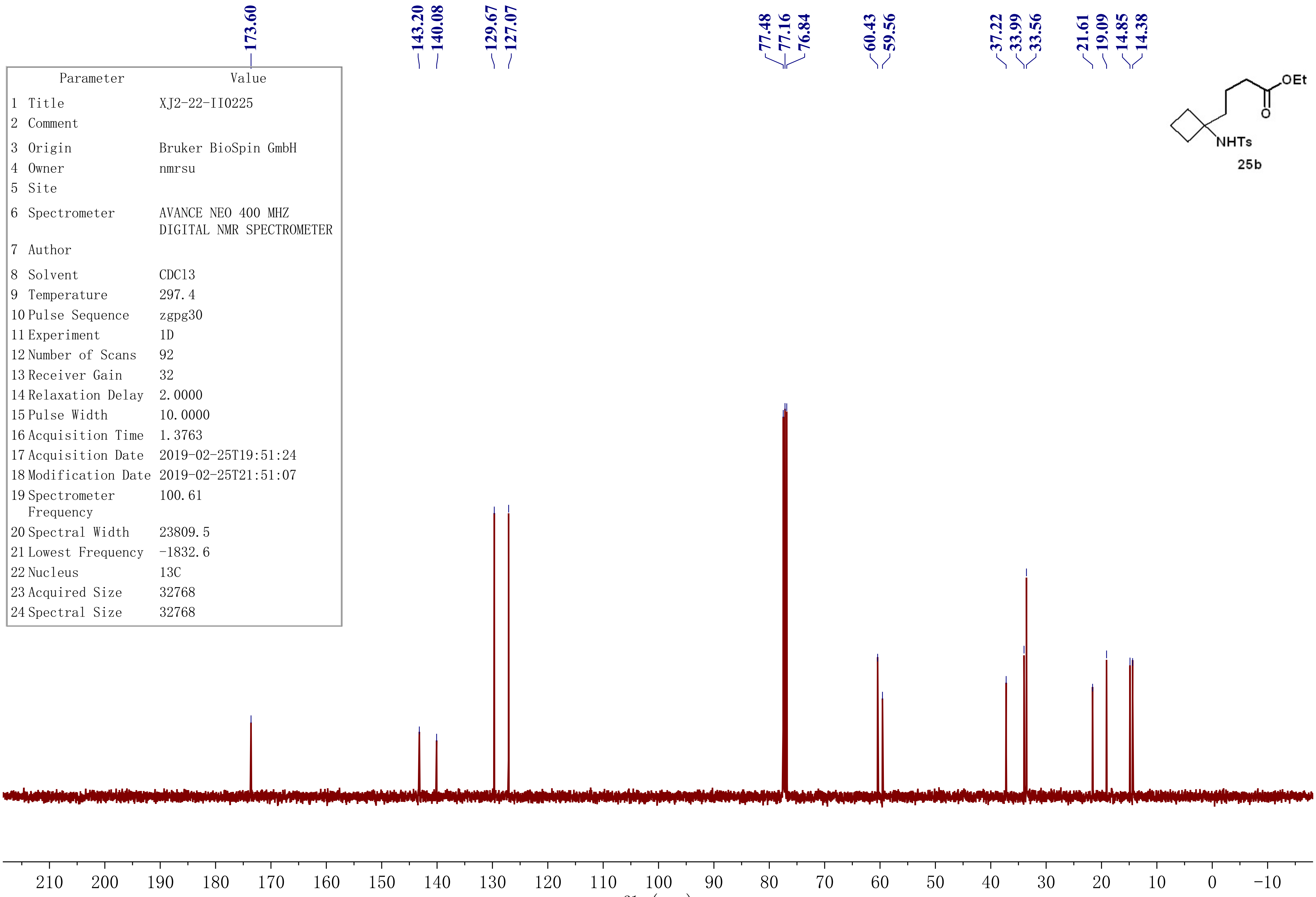

	Parameter	Value
1	Title	XJ2-22-II0225
2	Comment	
3	Origin	Bruker BioSpin GmbH
4	Owner	nmrsu
5	Site	
6	Spectrometer	AVANCE NEO 400 MHZ DIGITAL NMR SPECTROMETER
7	Author	
8	Solvent	CDCl3
9	Temperature	297.4
10	Pulse Sequence	zgpg30
11	Experiment	1D
12	Number of Scans	92
13	Receiver Gain	32
14	Relaxation Delay	2.0000
15	Pulse Width	10.0000
16	Acquisition Time	1.3763
17	Acquisition Date	2019-02-25T19:51:24
18	Modification Date	2019-02-25T21:51:07
19	Spectrometer Frequency	100.61
20	Spectral Width	23809.5
21	Lowest Frequency	-1832.6
22	Nucleus	13C
23	Acquired Size	32768
24	Spectral Size	32768

25b

Supplementary Figure 237. ^{13}C NMR spectrum of compound 25b

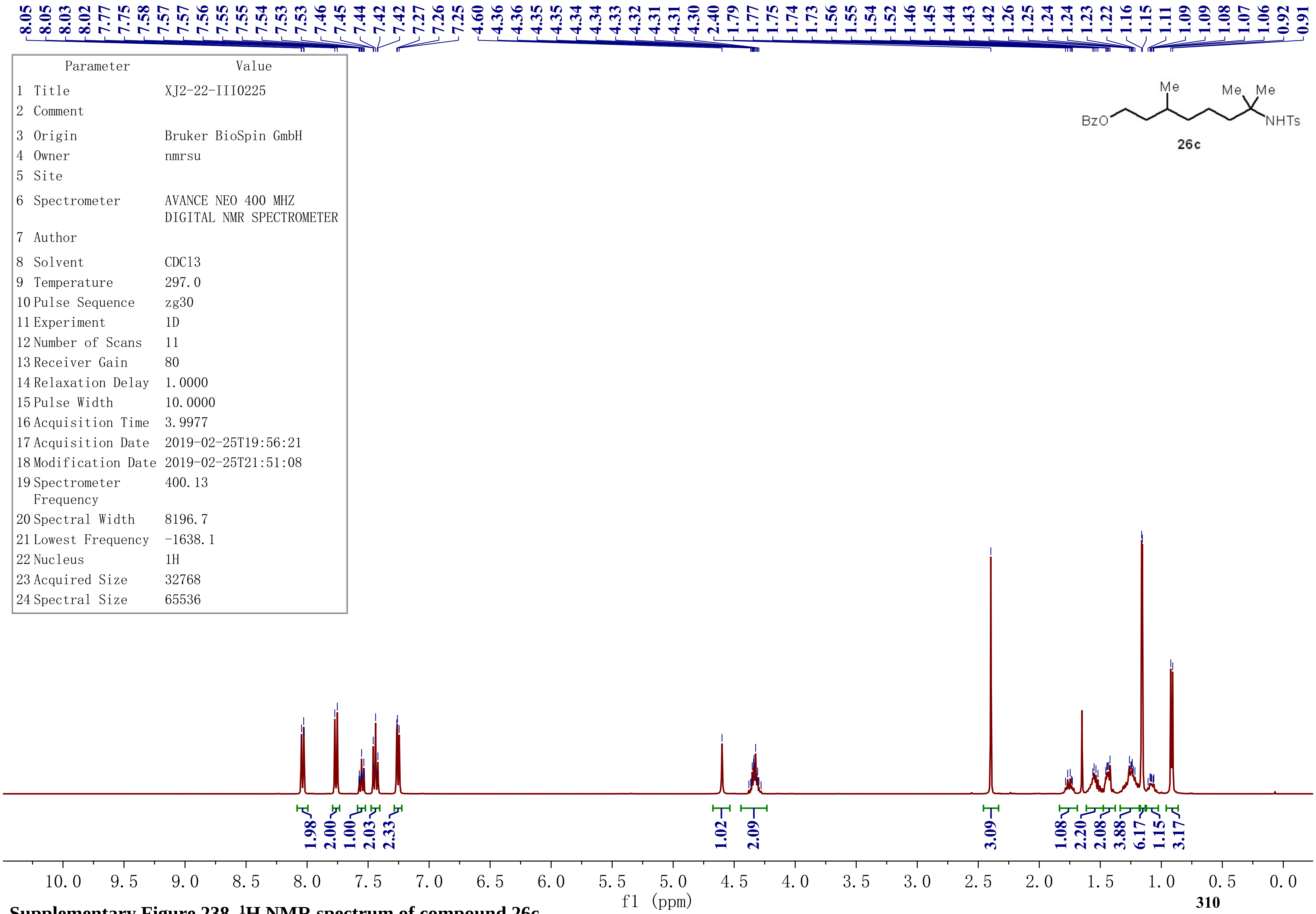

Supplementary Figure 238. ^{1}H NMR spectrum of compound 26c

Parameter	Value
1 Title	XJ2-22-III0225
2 Comment	
3 Origin	Bruker BioSpin GmbH
4 Owner	nmrsu
5 Site	
6 Spectrometer	AVANCE NEO 400 MHZ DIGITAL NMR SPECTROMETER
7 Author	
8 Solvent	CDCl3
9 Temperature	297.5
10 Pulse Sequence	zgpg30
11 Experiment	1D
12 Number of Scans	141
13 Receiver Gain	29
14 Relaxation Delay	2.0000
15 Pulse Width	10.0000
16 Acquisition Time	1.3763
17 Acquisition Date	2019-02-25T20:05:23
18 Modification Date	2019-02-25T21:51:09
19 Spectrometer Frequency	100.61
20 Spectral Width	23809.5
21 Lowest Frequency	-1832.4
22 Nucleus	13C
23 Acquired Size	32768
24 Spectral Size	32768

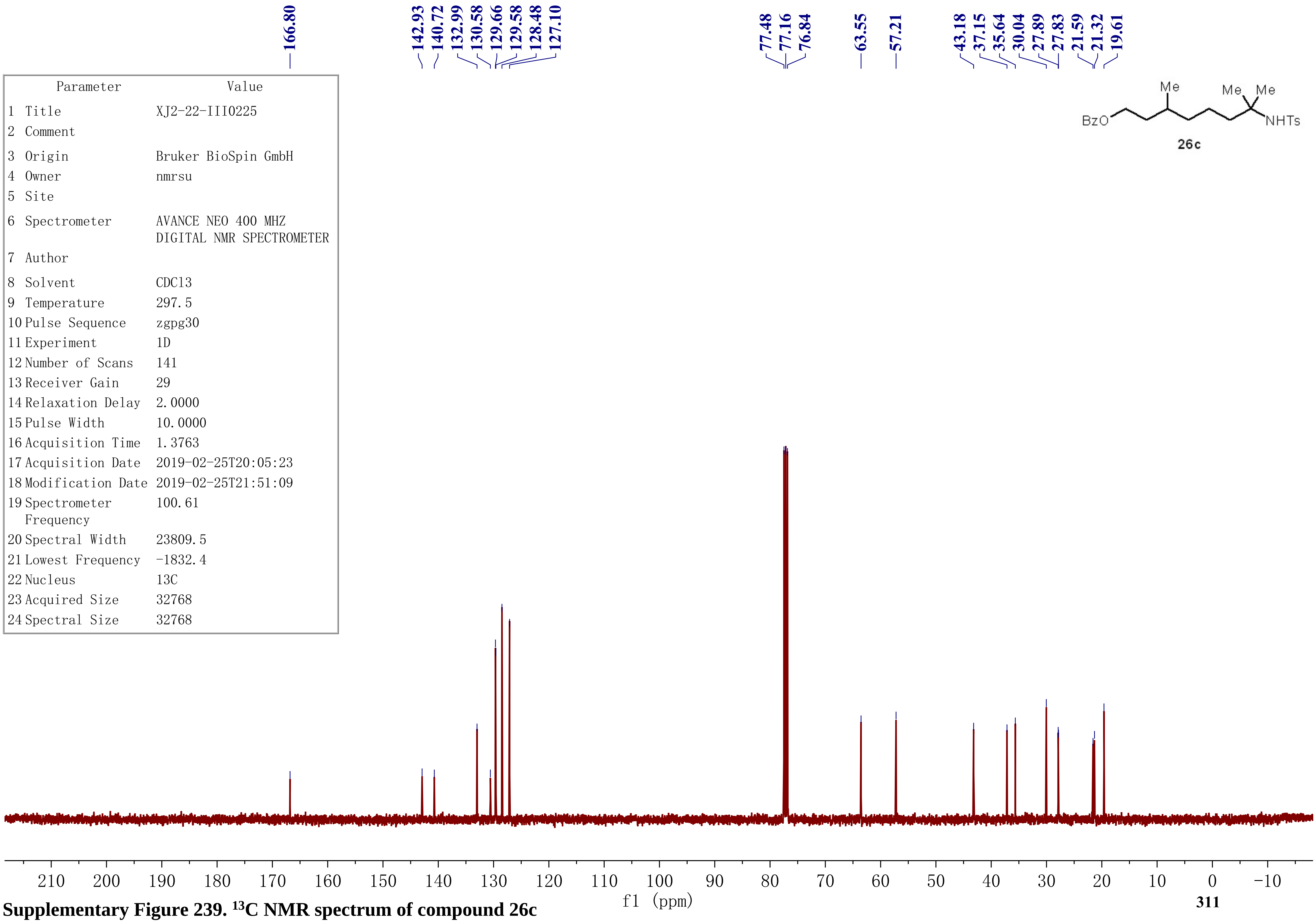

Supplementary Figure 239. ^{13}C NMR spectrum of compound 26c

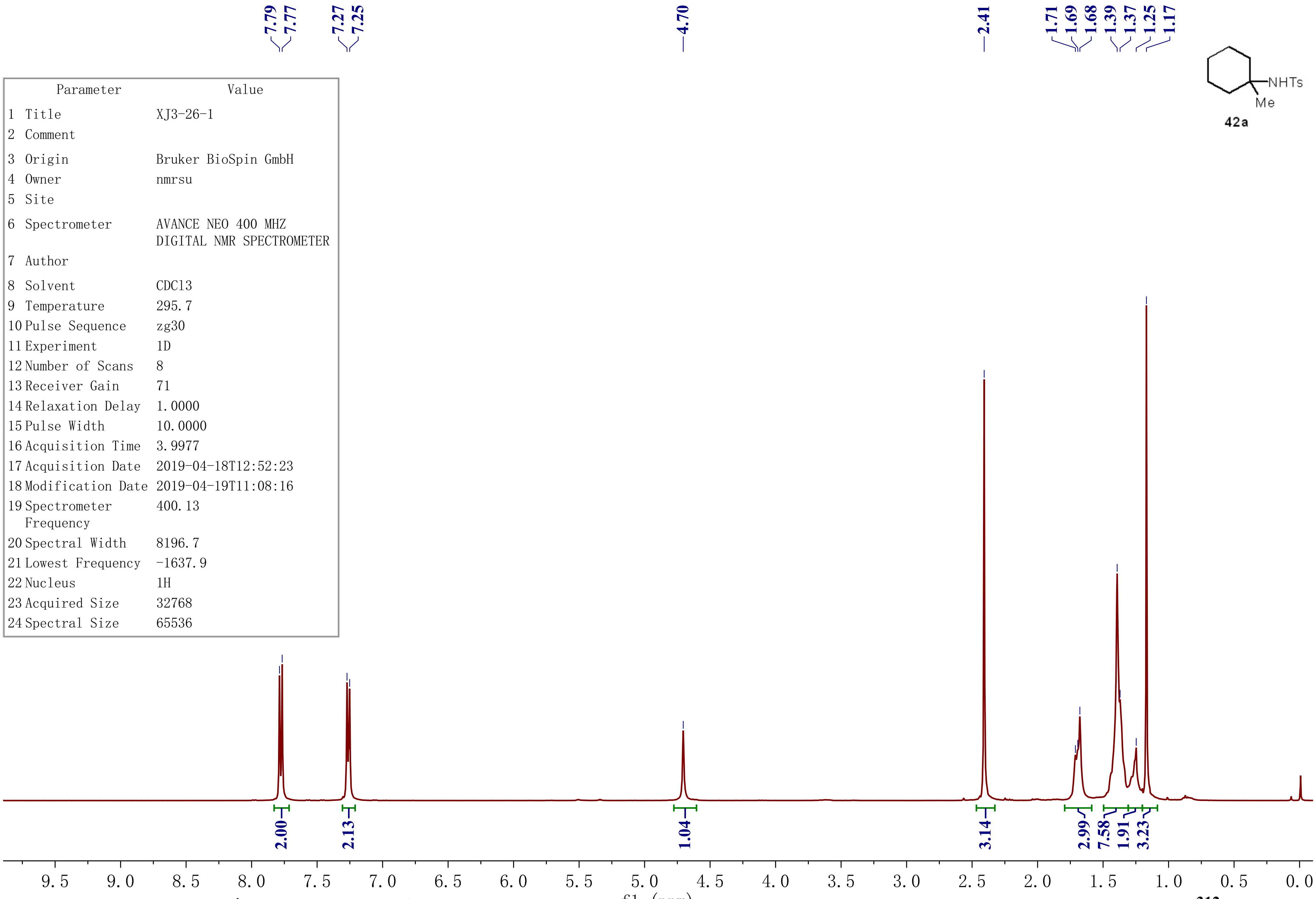

	Parameter	Value
1	Title	XJ3-26-1
2	Comment	
3	Origin	Bruker BioSpin GmbH
4	Owner	nmrsu
5	Site	
6	Spectrometer	AVANCE NEO 400 MHZ DIGITAL NMR SPECTROMETER
7	Author	
8	Solvent	CDCl3
9	Temperature	295.7
10	Pulse Sequence	zg30
11	Experiment	1D
12	Number of Scans	8
13	Receiver Gain	71
14	Relaxation Delay	1.0000
15	Pulse Width	10.0000
16	Acquisition Time	3.9977
17	Acquisition Date	2019-04-18T12:52:23
18	Modification Date	2019-04-19T11:08:16
19	Spectrometer Frequency	400.13
20	Spectral Width	8196.7
21	Lowest Frequency	-1637.9
22	Nucleus	1H
23	Acquired Size	32768
24	Spectral Size	65536

42a

Supplementary Figure 240. ^{1}H NMR spectrum of compound 42a

Parameter	Value
1 Title	XJ3-26-1
2 Comment	
3 Origin	Bruker BioSpin GmbH
4 Owner	nmrsu
5 Site	
6 Spectrometer	AVANCE NEO 400 MHZ DIGITAL NMR SPECTROMETER
7 Author	
8 Solvent	CDCl3
9 Temperature	296.1
10 Pulse Sequence	zgpg30
11 Experiment	1D
12 Number of Scans	68
13 Receiver Gain	42
14 Relaxation Delay	2.0000
15 Pulse Width	10.0000
16 Acquisition Time	1.3763
17 Acquisition Date	2019-04-18T12:57:33
18 Modification Date	2019-04-19T11:08:32
19 Spectrometer Frequency	100.61
20 Spectral Width	23809.5
21 Lowest Frequency	-1833.0
22 Nucleus	13C
23 Acquired Size	32768
24 Spectral Size	32768

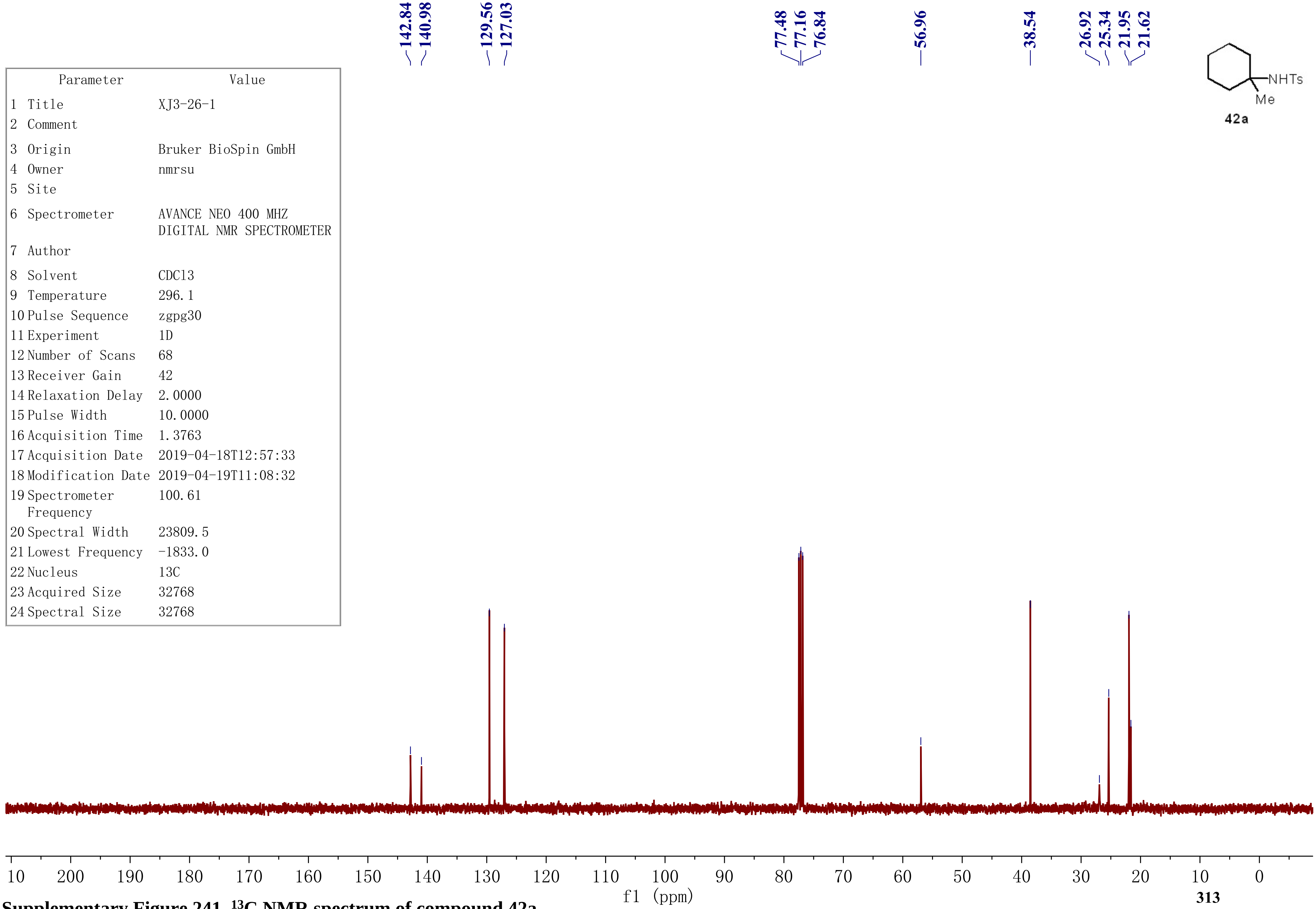

Supplementary Figure 241. ^{13}C NMR spectrum of compound 42a

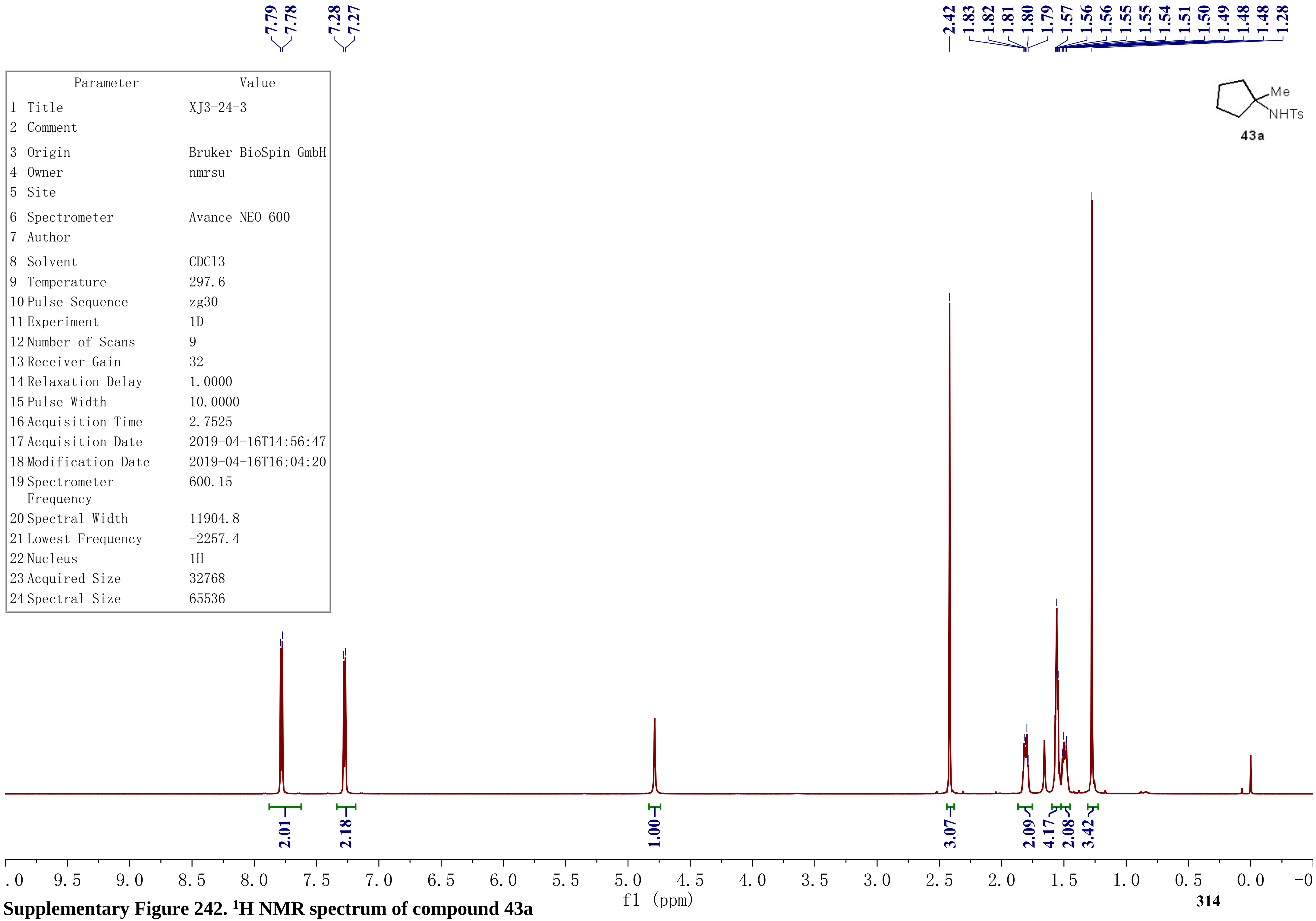

	Parameter	Value
1	Title	XJ3-24-3
2	Comment	
3	Origin	Bruker BioSpin GmbH
4	Owner	nmrsu
5	Site	
6	Spectrometer	Avance NEO 600
7	Author	
8	Solvent	CDCl3
9	Temperature	297.6
10	Pulse Sequence	zg30
11	Experiment	1D
12	Number of Scans	9
13	Receiver Gain	32
14	Relaxation Delay	1.0000
15	Pulse Width	10.0000
16	Acquisition Time	2.7525
17	Acquisition Date	2019-04-16T14:56:47
18	Modification Date	2019-04-16T16:04:20
19	Spectrometer Frequency	600.15
20	Spectral Width	11904.8
21	Lowest Frequency	-2257.4
22	Nucleus	1H
23	Acquired Size	32768
24	Spectral Size	65536

43a

Supplementary Figure 242. ^{1}H NMR spectrum of compound 43a

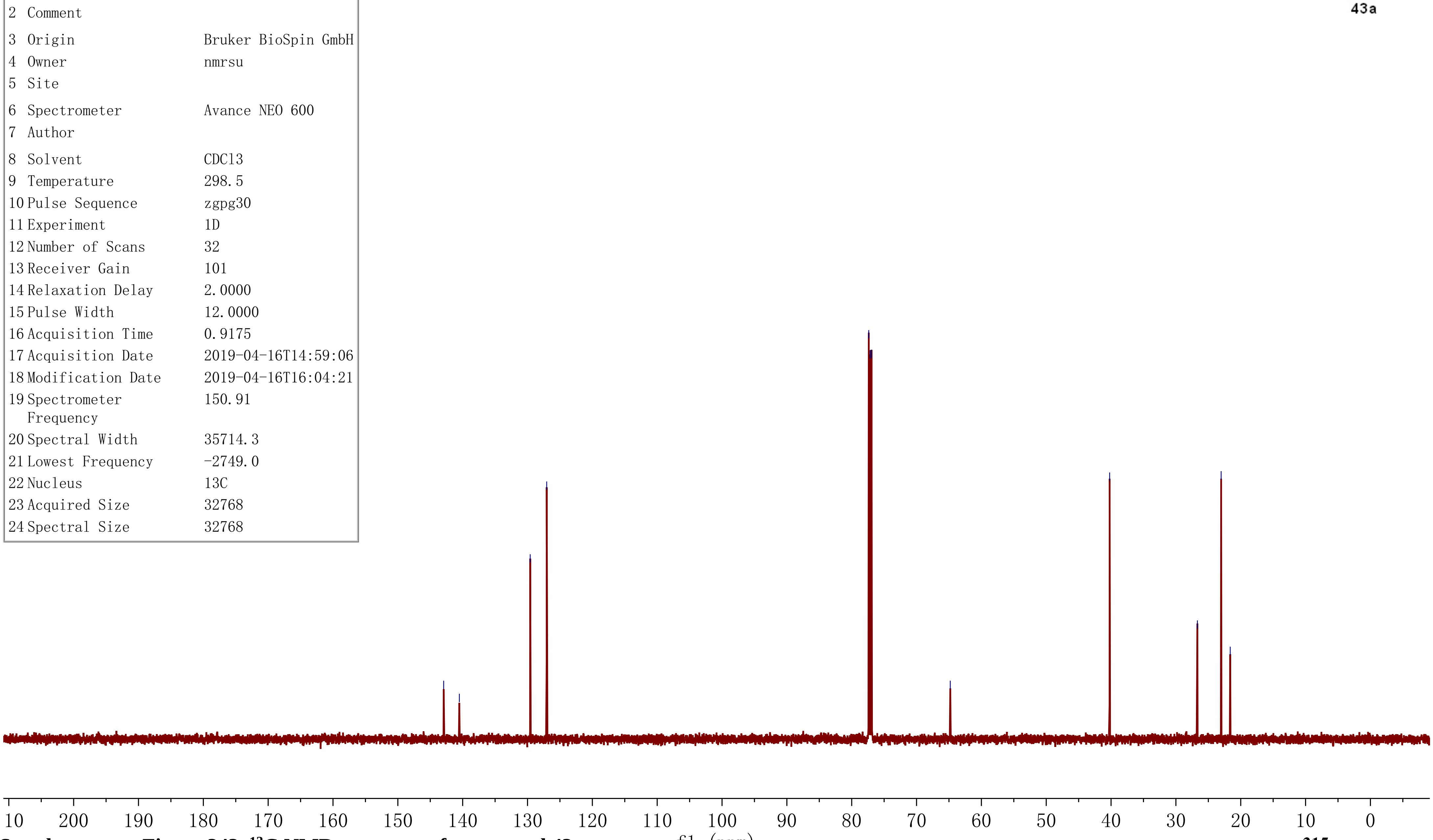

Parameter	Value
1 Title	XJ3-24-3
2 Comment	
3 Origin	Bruker BioSpin GmbH
4 Owner	nmrsu
5 Site	
6 Spectrometer	Avance NEO 600
7 Author	
8 Solvent	CDCl3
9 Temperature	298.5
10 Pulse Sequence	zgpg30
11 Experiment	1D
12 Number of Scans	32
13 Receiver Gain	101
14 Relaxation Delay	2.0000
15 Pulse Width	12.0000
16 Acquisition Time	0.9175
17 Acquisition Date	2019-04-16T14:59:06
18 Modification Date	2019-04-16T16:04:21
19 Spectrometer Frequency	150.91
20 Spectral Width	35714.3
21 Lowest Frequency	-2749.0
22 Nucleus	13C
23 Acquired Size	32768
24 Spectral Size	32768

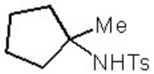

43a

Supplementary Figure 243. ^{13}C NMR spectrum of compound 43a

Parameter	Value
1 Title	XJ3-25-1
2 Comment	
3 Origin	Bruker BioSpin GmbH
4 Owner	nmrsu
5 Site	
6 Spectrometer	Avance NEO 600
7 Author	
8 Solvent	CDCl3
9 Temperature	297.0
10 Pulse Sequence	zg30
11 Experiment	1D
12 Number of Scans	14
13 Receiver Gain	32
14 Relaxation Delay	1.0000
15 Pulse Width	10.0000
16 Acquisition Time	2.7525
17 Acquisition Date	2019-04-19T10:46:37
18 Modification Date	2019-04-19T11:07:43
19 Spectrometer Frequency	600.15
20 Spectral Width	11904.8
21 Lowest Frequency	-2260.9
22 Nucleus	1H
23 Acquired Size	32768
24 Spectral Size	65536

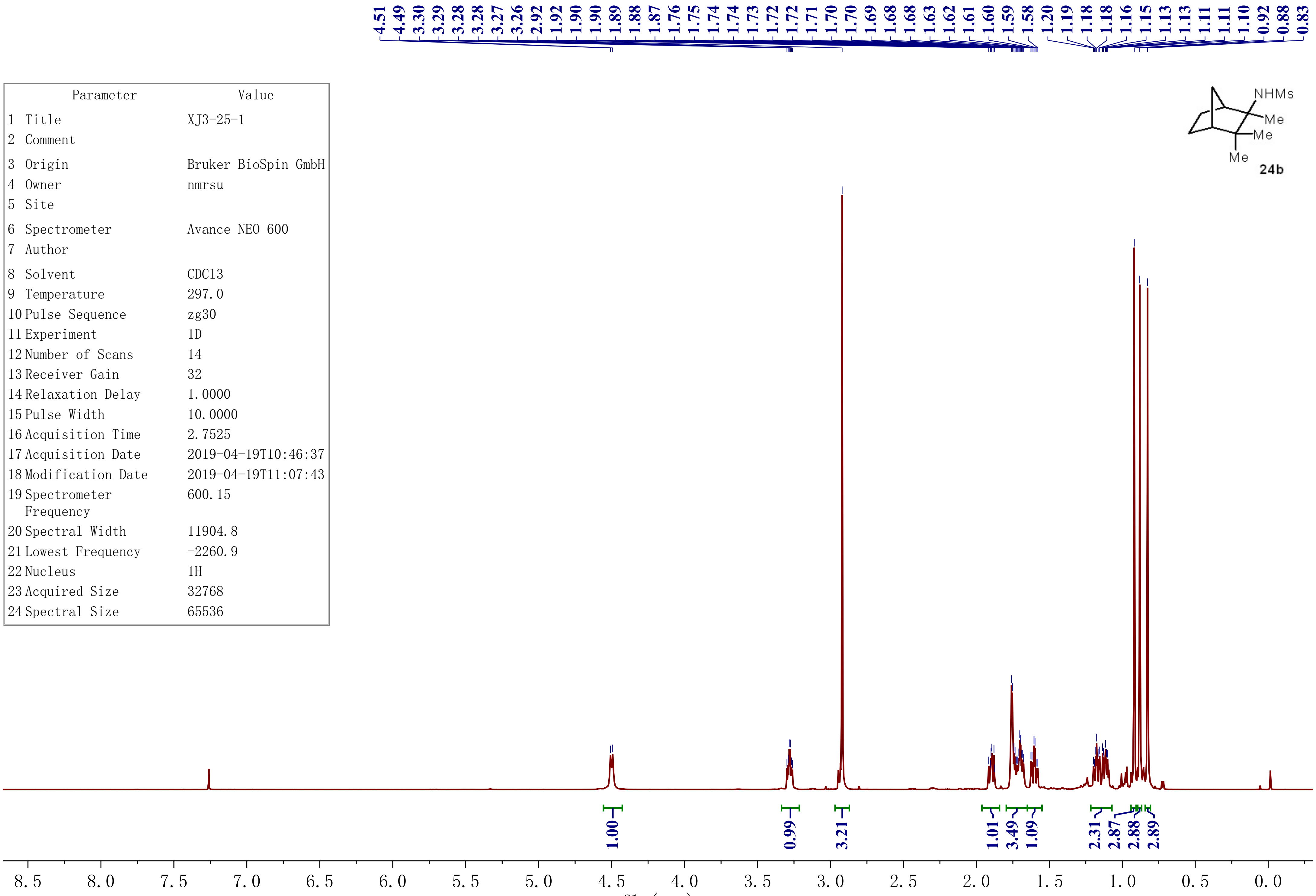

Supplementary Figure 244. ^{1}H NMR spectrum of compound 24b

Parameter		Value
1	Title	XJ3-25-1
2	Comment	
3	Origin	Bruker BioSpin GmbH
4	Owner	nmrsu
5	Site	
6	Spectrometer	Avance NEO 600
7	Author	
8	Solvent	CDCl3
9	Temperature	298.2
10	Pulse Sequence	zgpg30
11	Experiment	1D
12	Number of Scans	41
13	Receiver Gain	101
14	Relaxation Delay	2.0000
15	Pulse Width	12.0000
16	Acquisition Time	0.9175
17	Acquisition Date	2019-04-19T10:49:48
18	Modification Date	2019-04-19T11:07:56
19	Spectrometer Frequency	150.91
20	Spectral Width	35714.3
21	Lowest Frequency	-2751.4
22	Nucleus	13C
23	Acquired Size	32768
24	Spectral Size	32768

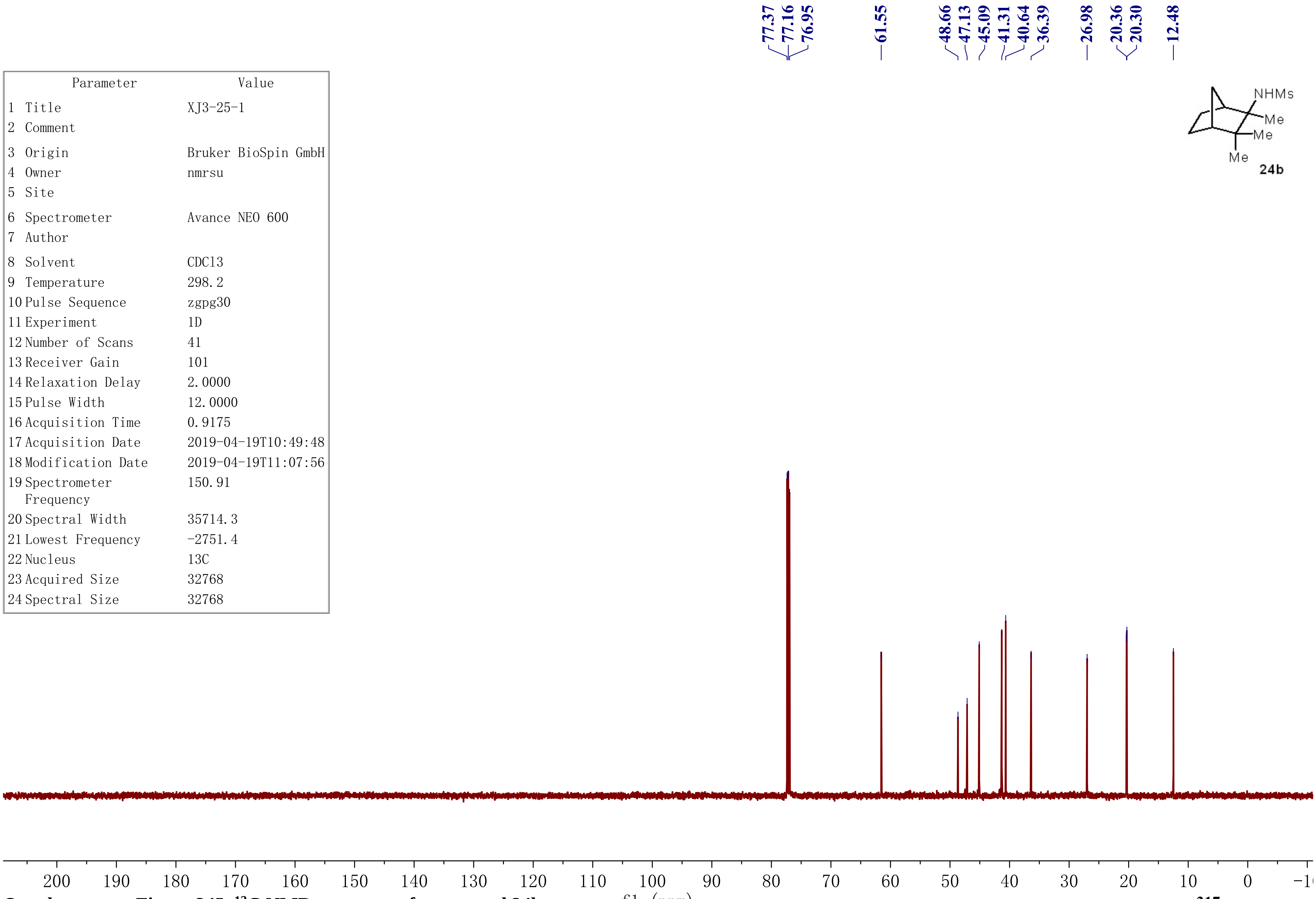

24b

Supplementary Figure 245. ^{13}C NMR spectrum of compound 24b

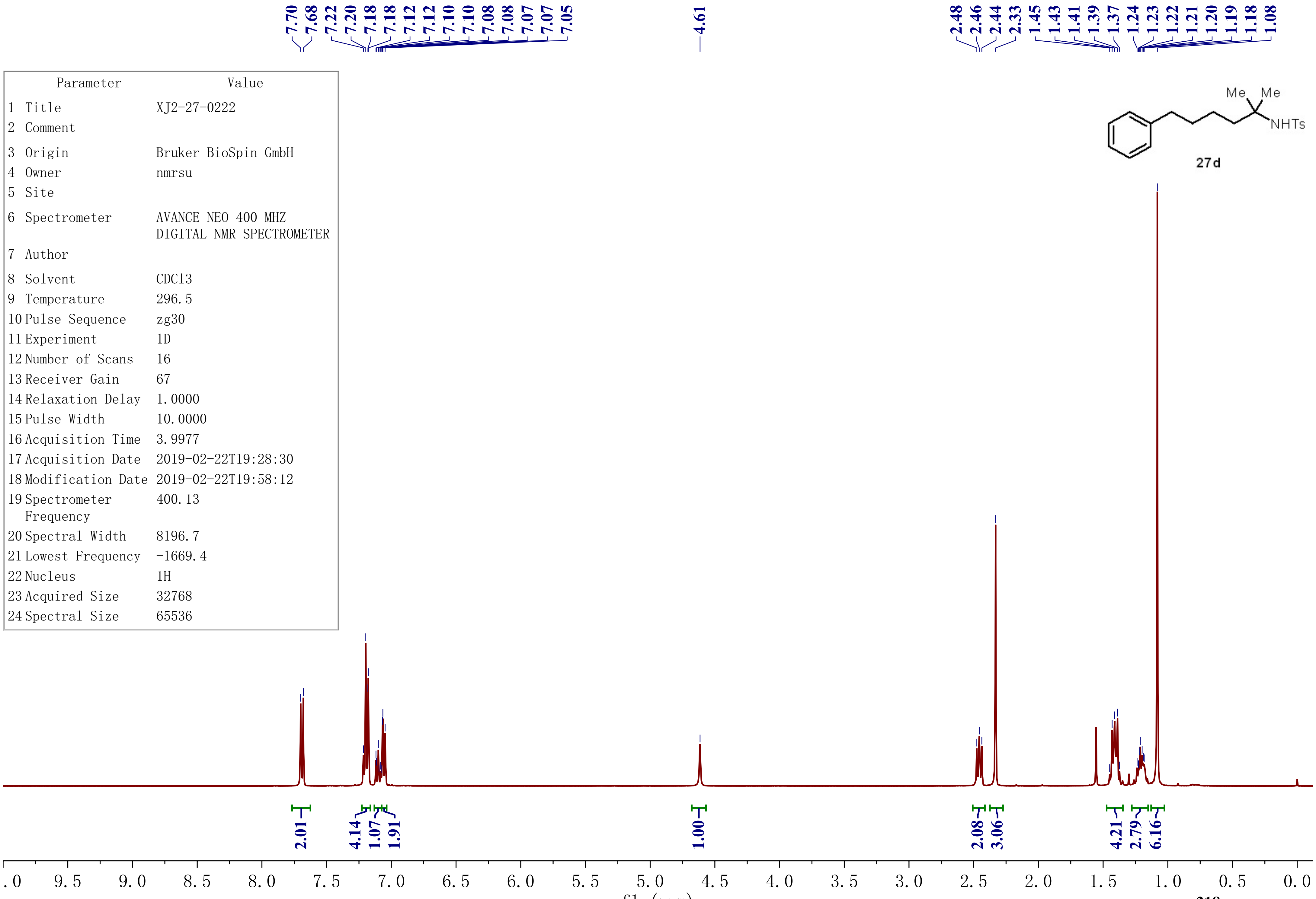

	Parameter	Value
1	Title	XJ2-27-0222
2	Comment	
3	Origin	Bruker BioSpin GmbH
4	Owner	nmrsu
5	Site	
6	Spectrometer	AVANCE NEO 400 MHZ DIGITAL NMR SPECTROMETER
7	Author	
8	Solvent	CDCl3
9	Temperature	296.5
10	Pulse Sequence	zg30
11	Experiment	1D
12	Number of Scans	16
13	Receiver Gain	67
14	Relaxation Delay	1.0000
15	Pulse Width	10.0000
16	Acquisition Time	3.9977
17	Acquisition Date	2019-02-22T19:28:30
18	Modification Date	2019-02-22T19:58:12
19	Spectrometer Frequency	400.13
20	Spectral Width	8196.7
21	Lowest Frequency	-1669.4
22	Nucleus	1H
23	Acquired Size	32768
24	Spectral Size	65536

Supplementary Figure 246. ^1H NMR spectrum of compound 27d

Parameter	Value
1 Title	XJ2-27-0222
2 Comment	
3 Origin	Bruker BioSpin GmbH
4 Owner	nmrsu
5 Site	
6 Spectrometer	AVANCE NEO 400 MHZ DIGITAL NMR SPECTROMETER
7 Author	
8 Solvent	CDCl3
9 Temperature	295.9
10 Pulse Sequence	zgpg30
11 Experiment	1D
12 Number of Scans	76
13 Receiver Gain	29
14 Relaxation Delay	2.0000
15 Pulse Width	10.0000
16 Acquisition Time	1.3763
17 Acquisition Date	2019-02-22T19:55:57
18 Modification Date	2019-02-22T19:58:13
19 Spectrometer Frequency	100.61
20 Spectral Width	23809.5
21 Lowest Frequency	-1834.1
22 Nucleus	13C
23 Acquired Size	32768
24 Spectral Size	32768

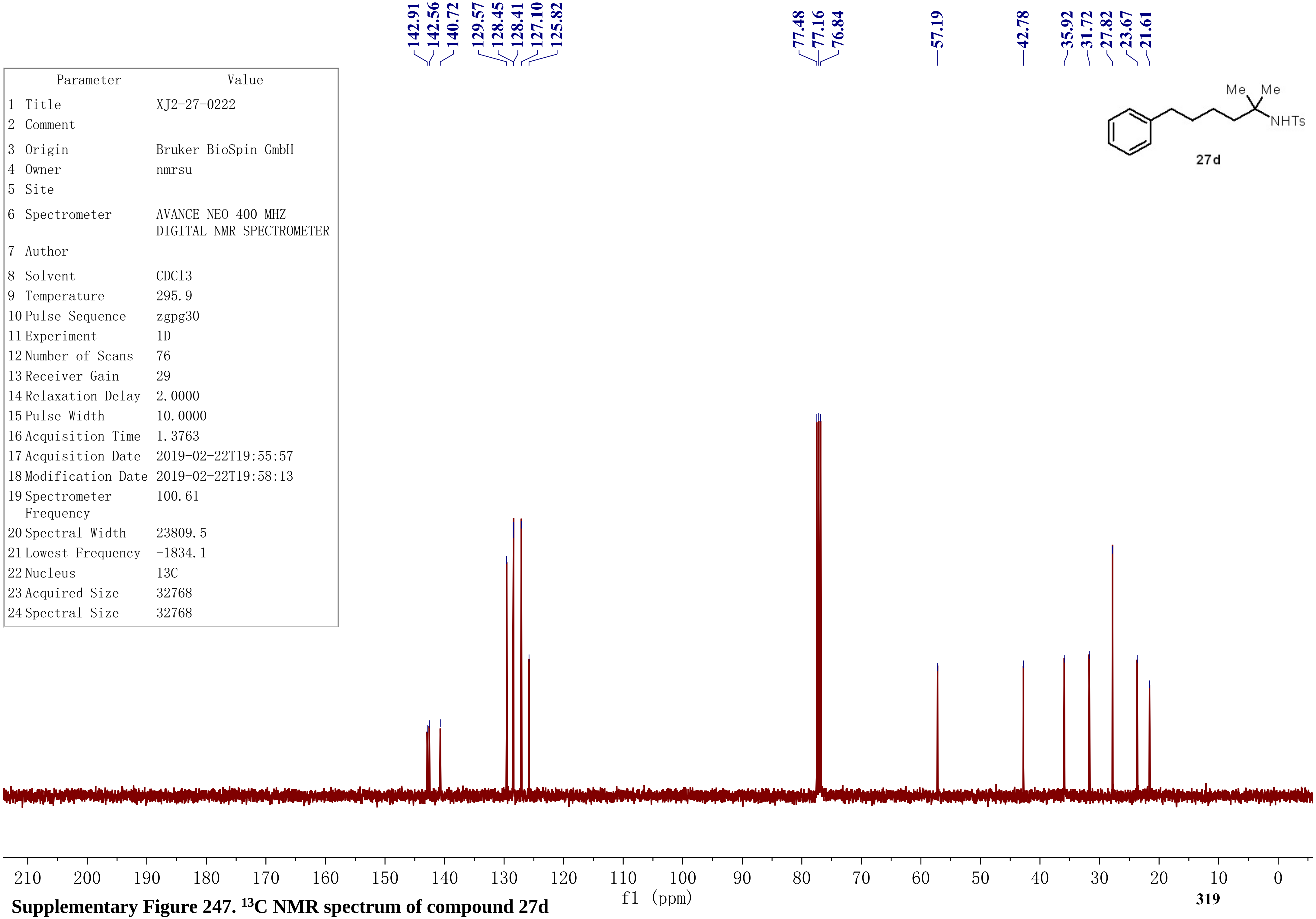

Supplementary Figure 247. ^{13}**C NMR spectrum of compound 27d**

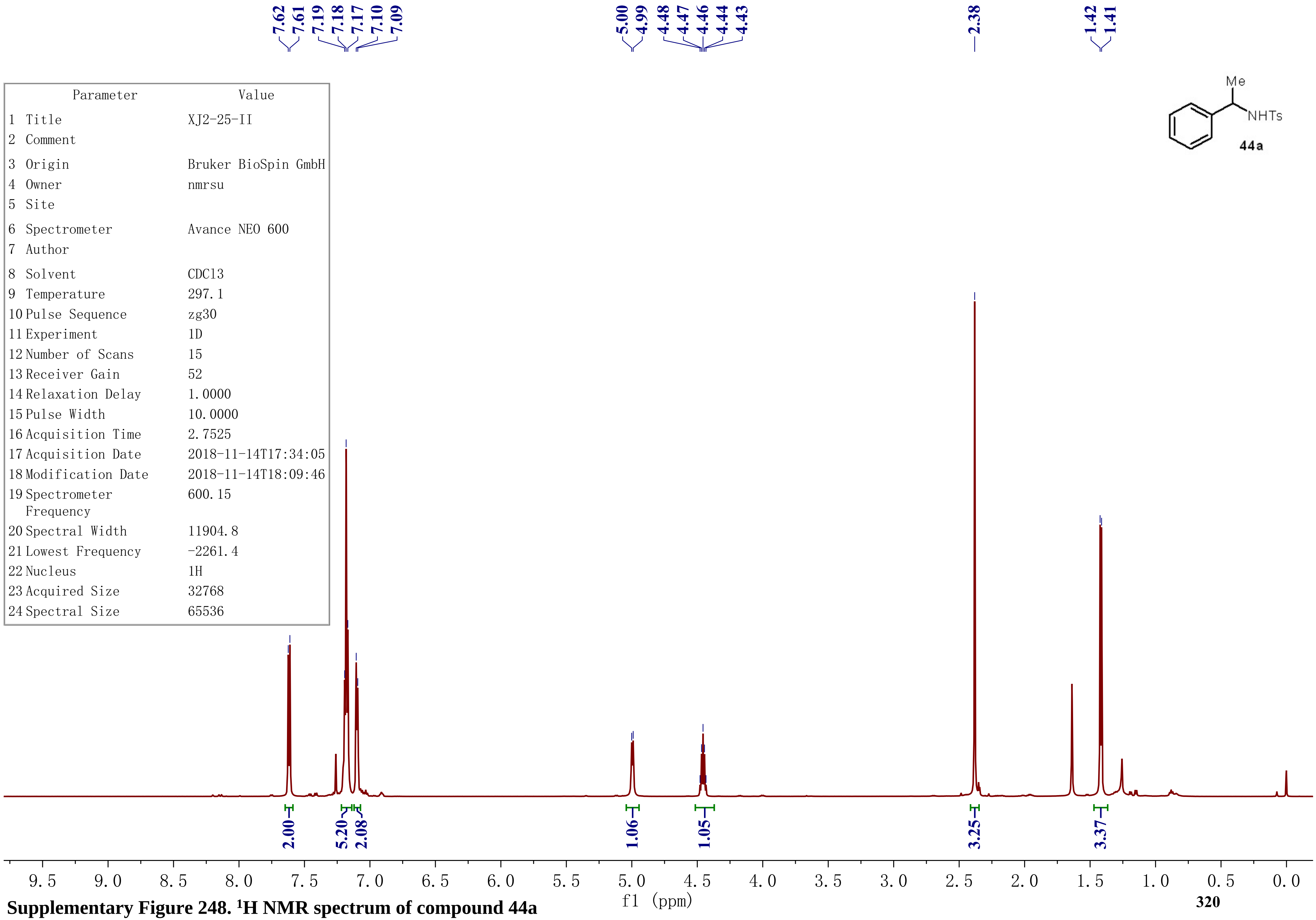

	Parameter	Value
1	Title	XJ2-25-II
2	Comment	
3	Origin	Bruker BioSpin GmbH
4	Owner	nmrsu
5	Site	
6	Spectrometer	Avance NEO 600
7	Author	
8	Solvent	CDCl3
9	Temperature	297.1
10	Pulse Sequence	zg30
11	Experiment	1D
12	Number of Scans	15
13	Receiver Gain	52
14	Relaxation Delay	1.0000
15	Pulse Width	10.0000
16	Acquisition Time	2.7525
17	Acquisition Date	2018-11-14T17:34:05
18	Modification Date	2018-11-14T18:09:46
19	Spectrometer Frequency	600.15
20	Spectral Width	11904.8
21	Lowest Frequency	-2261.4
22	Nucleus	1H
23	Acquired Size	32768
24	Spectral Size	65536

Supplementary Figure 248. ^{1}H NMR spectrum of compound 44a

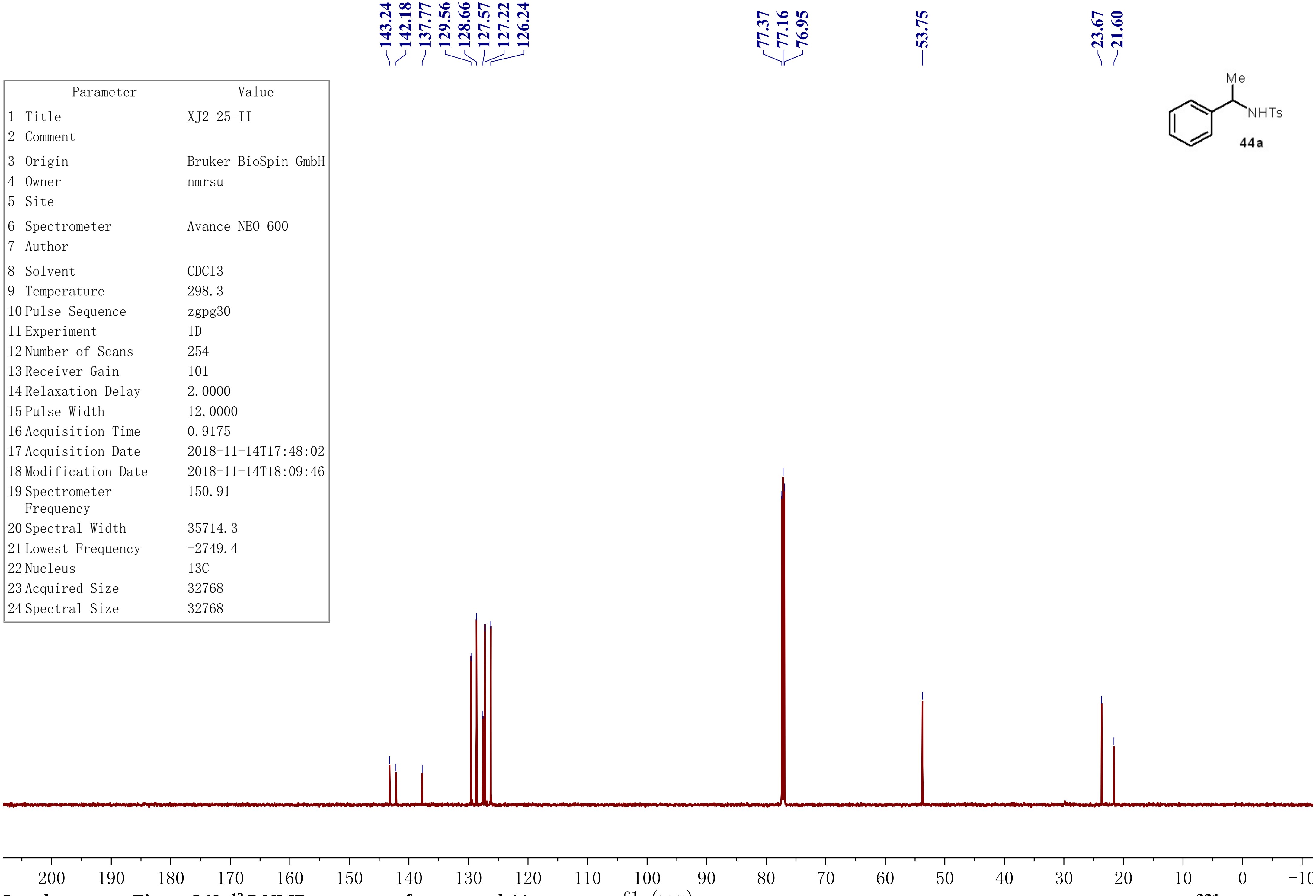

Parameter	Value
1 Title	XJ2-25-II
2 Comment	
3 Origin	Bruker BioSpin GmbH
4 Owner	nmrsu
5 Site	
6 Spectrometer	Avance NEO 600
7 Author	
8 Solvent	CDCl3
9 Temperature	298.3
10 Pulse Sequence	zgpg30
11 Experiment	1D
12 Number of Scans	254
13 Receiver Gain	101
14 Relaxation Delay	2.0000
15 Pulse Width	12.0000
16 Acquisition Time	0.9175
17 Acquisition Date	2018-11-14T17:48:02
18 Modification Date	2018-11-14T18:09:46
19 Spectrometer Frequency	150.91
20 Spectral Width	35714.3
21 Lowest Frequency	-2749.4
22 Nucleus	13C
23 Acquired Size	32768
24 Spectral Size	32768

44a

Supplementary Figure 249. ^{13}C NMR spectrum of compound 44a

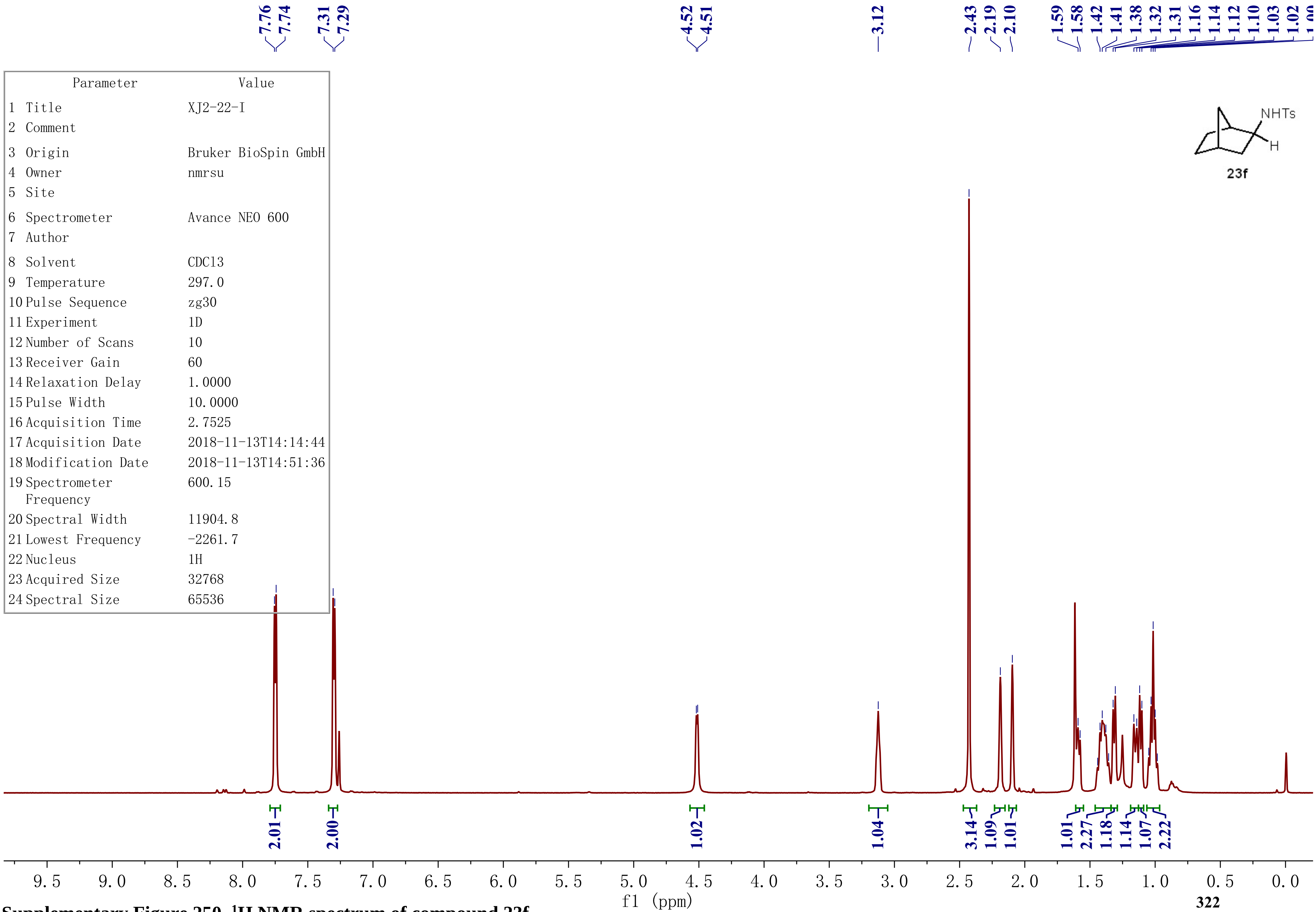

Supplementary Figure 250. ^{1}H **NMR spectrum of compound 23f**

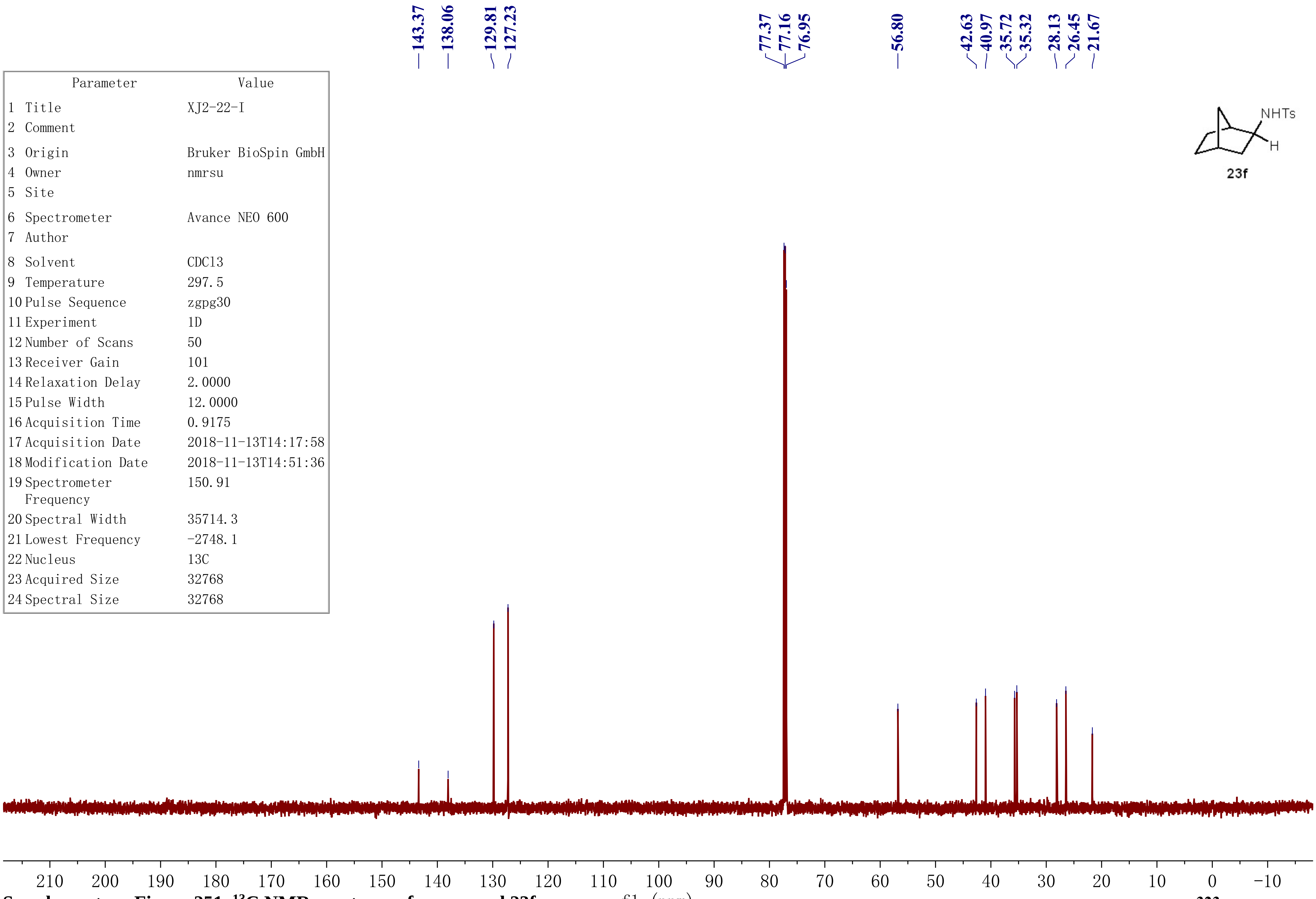

	Parameter	Value
1	Title	XJ2-22-I
2	Comment	
3	Origin	Bruker BioSpin GmbH
4	Owner	nmrsu
5	Site	
6	Spectrometer	Avance NEO 600
7	Author	
8	Solvent	CDCl3
9	Temperature	297.5
10	Pulse Sequence	zgpg30
11	Experiment	1D
12	Number of Scans	50
13	Receiver Gain	101
14	Relaxation Delay	2.0000
15	Pulse Width	12.0000
16	Acquisition Time	0.9175
17	Acquisition Date	2018-11-13T14:17:58
18	Modification Date	2018-11-13T14:51:36
19	Spectrometer Frequency	150.91
20	Spectral Width	35714.3
21	Lowest Frequency	-2748.1
22	Nucleus	13C
23	Acquired Size	32768
24	Spectral Size	32768

NHTs
H
23f

Supplementary Figure 251. ^{13}C NMR spectrum of compound 23f

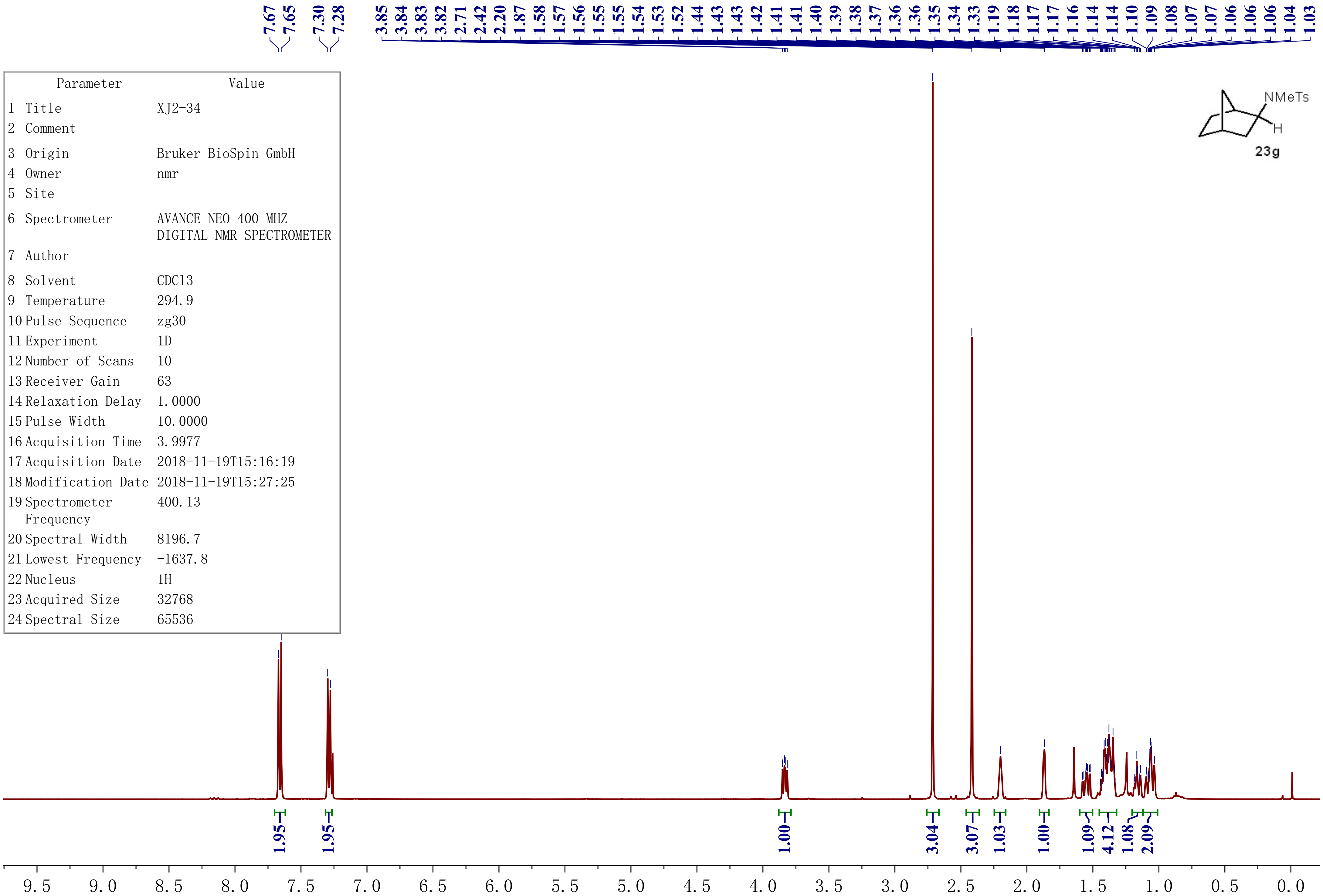

Supplementary Figure 252. ^{1}H **NMR spectrum of compound 23g**

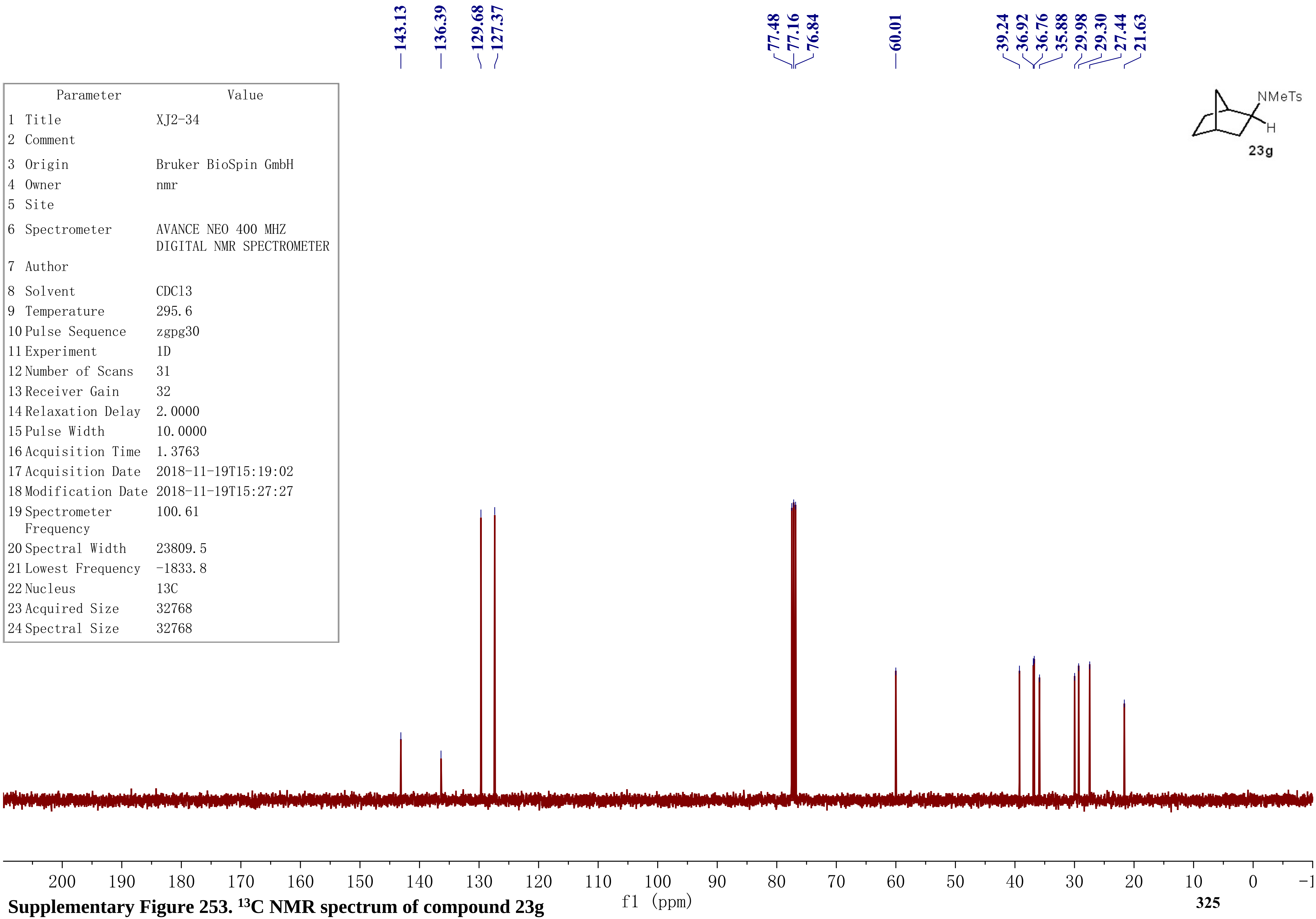

Parameter	Value
1 Title	XJ2-34
2 Comment	
3 Origin	Bruker BioSpin GmbH
4 Owner	nmr
5 Site	
6 Spectrometer	AVANCE NEO 400 MHZ DIGITAL NMR SPECTROMETER
7 Author	
8 Solvent	CDCl3
9 Temperature	295.6
10 Pulse Sequence	zgpg30
11 Experiment	1D
12 Number of Scans	31
13 Receiver Gain	32
14 Relaxation Delay	2.0000
15 Pulse Width	10.0000
16 Acquisition Time	1.3763
17 Acquisition Date	2018-11-19T15:19:02
18 Modification Date	2018-11-19T15:27:27
19 Spectrometer Frequency	100.61
20 Spectral Width	23809.5
21 Lowest Frequency	-1833.8
22 Nucleus	13C
23 Acquired Size	32768
24 Spectral Size	32768

Supplementary Figure 253. ^{13}C NMR spectrum of compound 23g

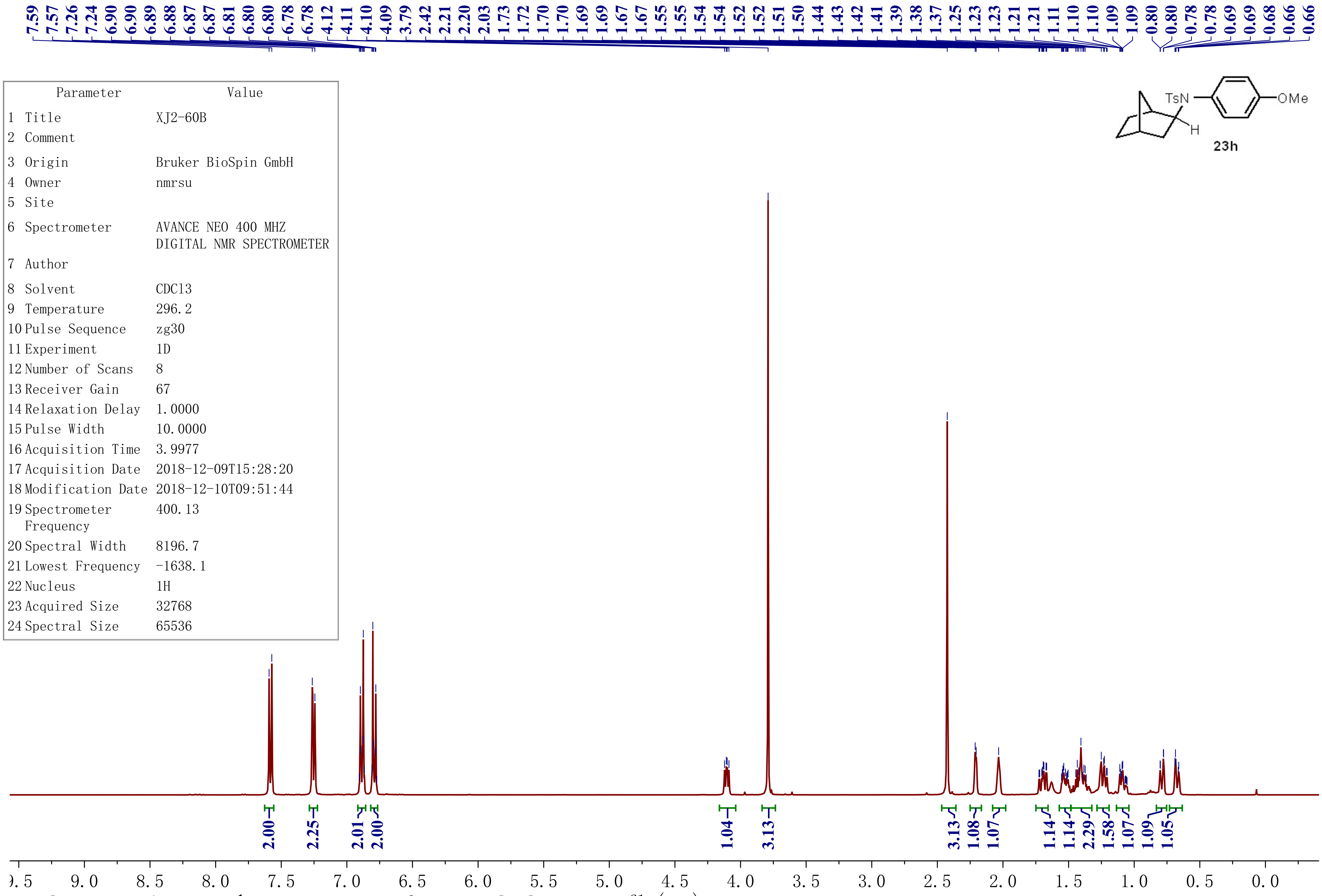

Supplementary Figure 254. ^{1}H NMR spectrum of compound 23h

	Parameter	Value
1	Title	XJ2-60B
2	Comment	
3	Origin	Bruker BioSpin GmbH
4	Owner	nmrsu
5	Site	
6	Spectrometer	AVANCE NEO 400 MHZ DIGITAL NMR SPECTROMETER
7	Author	
8	Solvent	CDCl3
9	Temperature	296.5
10	Pulse Sequence	zgpg30
11	Experiment	1D
12	Number of Scans	100
13	Receiver Gain	32
14	Relaxation Delay	2.0000
15	Pulse Width	10.0000
16	Acquisition Time	1.3763
17	Acquisition Date	2018-12-09T15:35:17
18	Modification Date	2018-12-10T09:51:45
19	Spectrometer Frequency	100.61
20	Spectral Width	23809.5
21	Lowest Frequency	-1833.3
22	Nucleus	13C
23	Acquired Size	32768
24	Spectral Size	32768

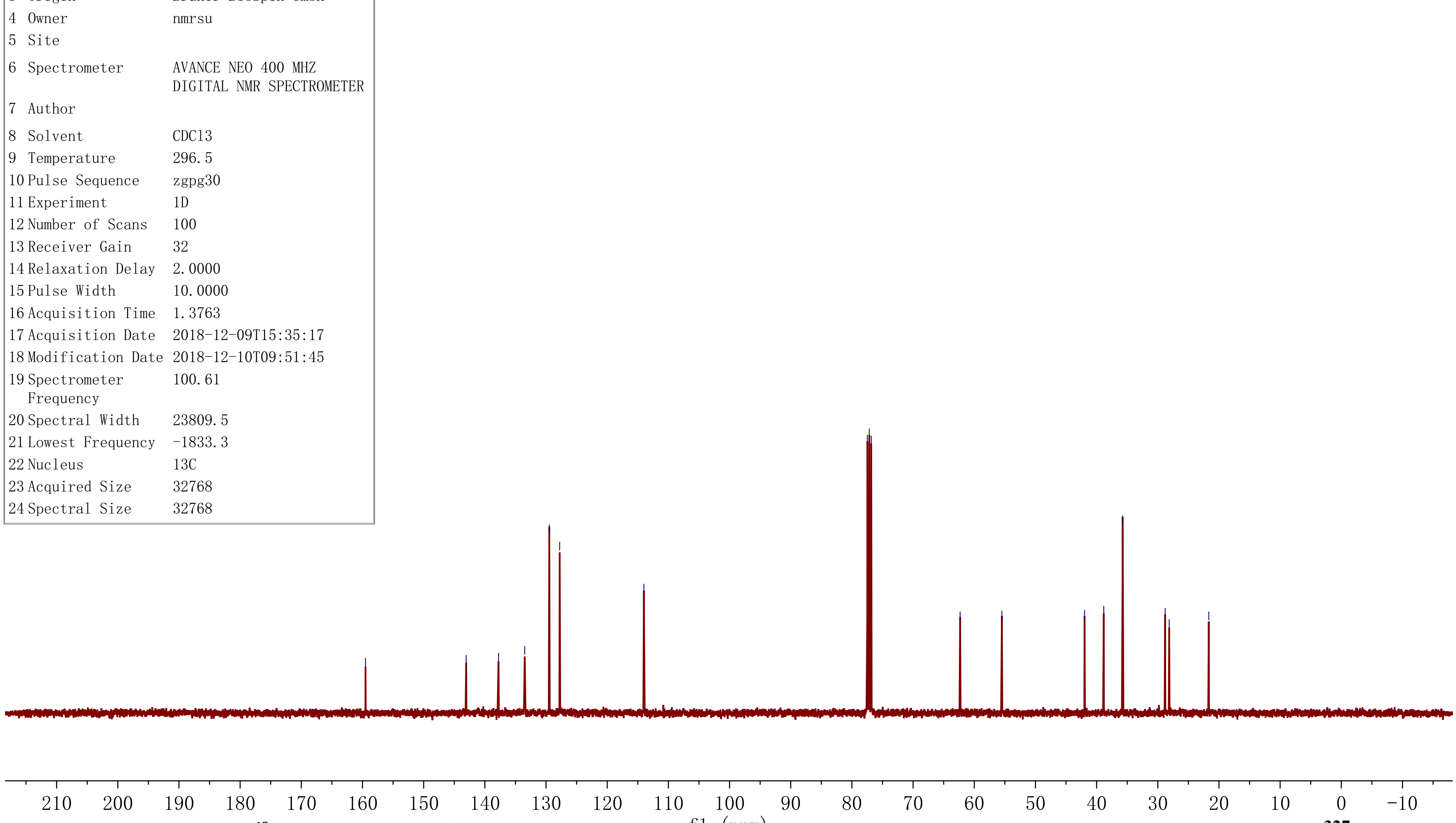

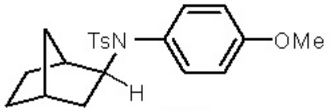

23h

Supplementary Figure 255. ^{13}C NMR spectrum of compound 23h

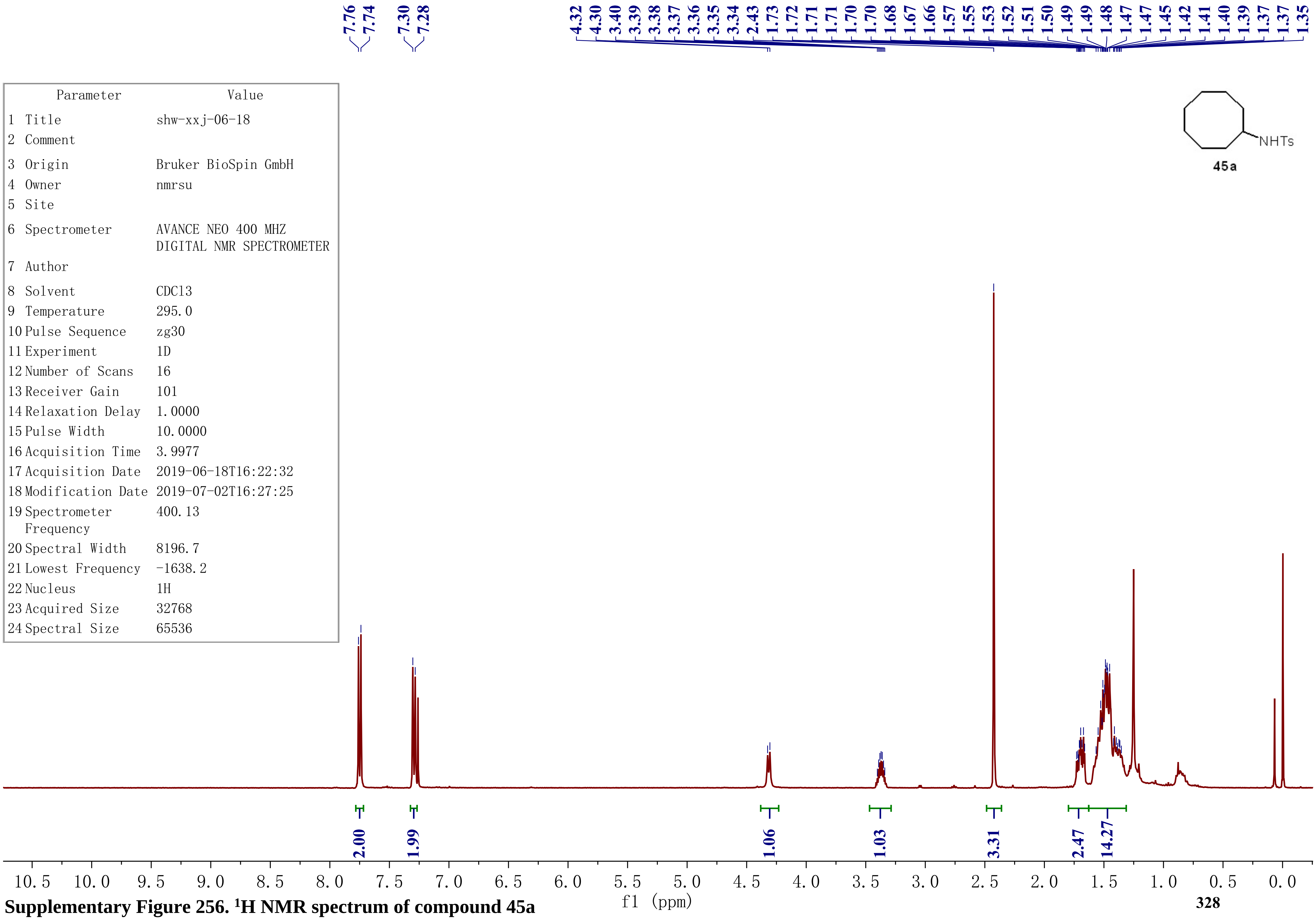

Supplementary Figure 256. ^{1}H NMR spectrum of compound 45a

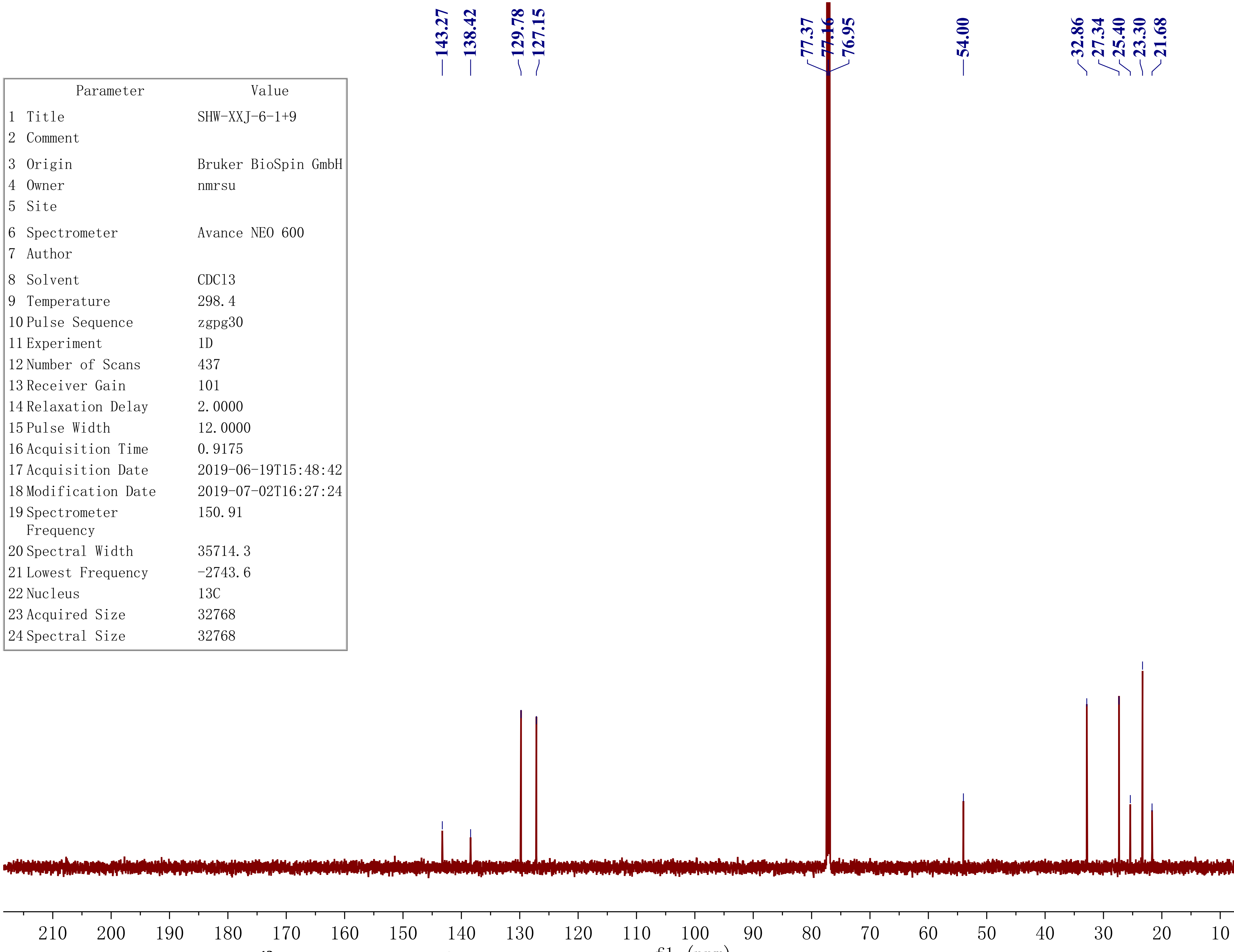

Parameter	Value
1 Title	SHW-XXJ-6-1+9
2 Comment	
3 Origin	Bruker BioSpin GmbH
4 Owner	nmrsu
5 Site	
6 Spectrometer	Avance NEO 600
7 Author	
8 Solvent	CDCl3
9 Temperature	298.4
10 Pulse Sequence	zgpg30
11 Experiment	1D
12 Number of Scans	437
13 Receiver Gain	101
14 Relaxation Delay	2.0000
15 Pulse Width	12.0000
16 Acquisition Time	0.9175
17 Acquisition Date	2019-06-19T15:48:42
18 Modification Date	2019-07-02T16:27:24
19 Spectrometer Frequency	150.91
20 Spectral Width	35714.3
21 Lowest Frequency	-2743.6
22 Nucleus	13C
23 Acquired Size	32768
24 Spectral Size	32768

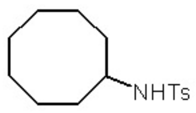

45a

Supplementary Figure 257. ^{13}C **NMR spectrum of compound 45a**

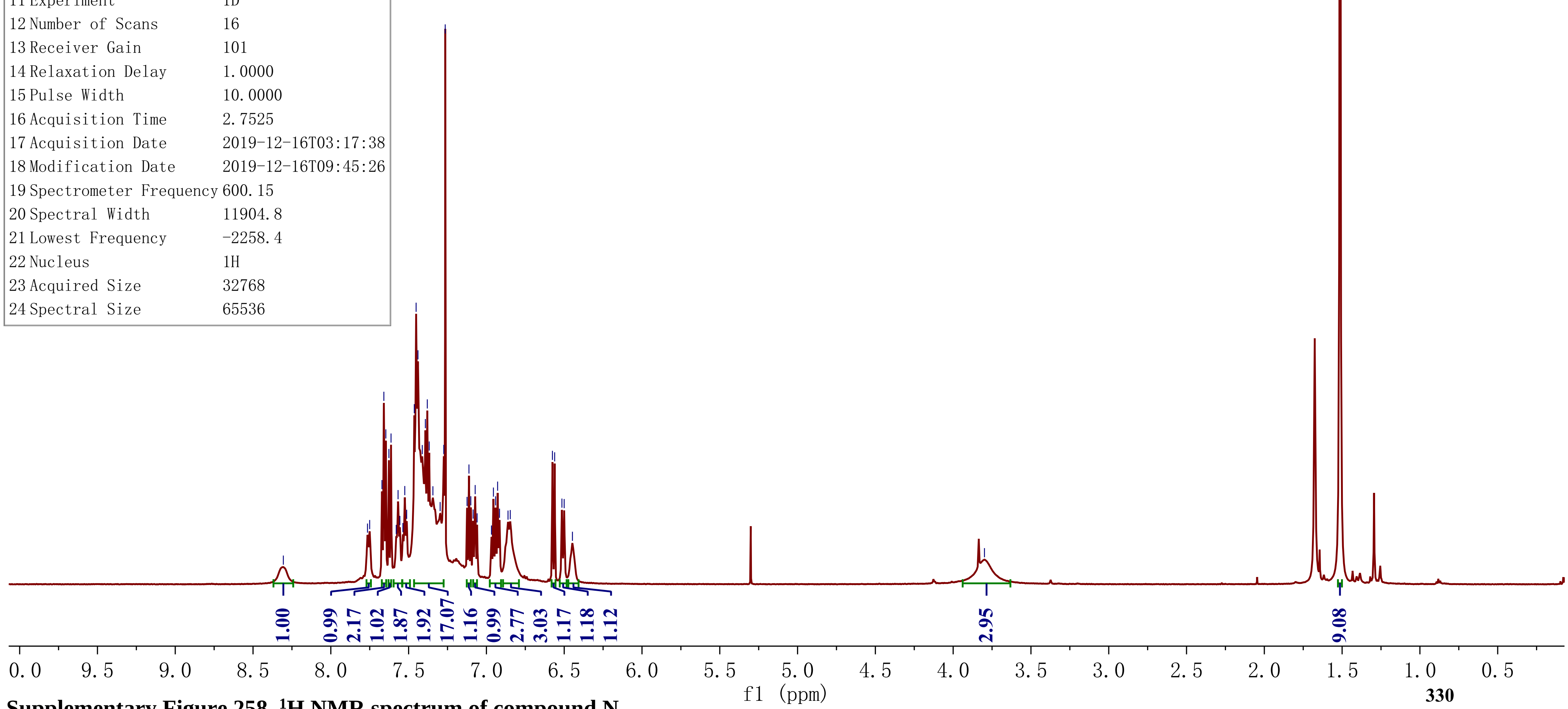

Parameter	Value
1 Title	ZYLF88-1D
2 Comment	
3 Origin	Bruker BioSpin GmbH
4 Owner	nmrsu
5 Site	
6 Spectrometer	Avance NEO 600
7 Author	
8 Solvent	CDCl3
9 Temperature	296.5
10 Pulse Sequence	zg30
11 Experiment	1D
12 Number of Scans	16
13 Receiver Gain	101
14 Relaxation Delay	1.0000
15 Pulse Width	10.0000
16 Acquisition Time	2.7525
17 Acquisition Date	2019-12-16T03:17:38
18 Modification Date	2019-12-16T09:45:26
19 Spectrometer Frequency	600.15
20 Spectral Width	11904.8
21 Lowest Frequency	-2258.4
22 Nucleus	1H
23 Acquired Size	32768
24 Spectral Size	65536

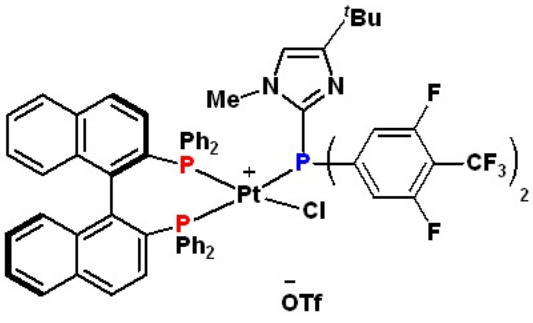

N

Supplementary Figure 258. ^{1}H NMR spectrum of compound N

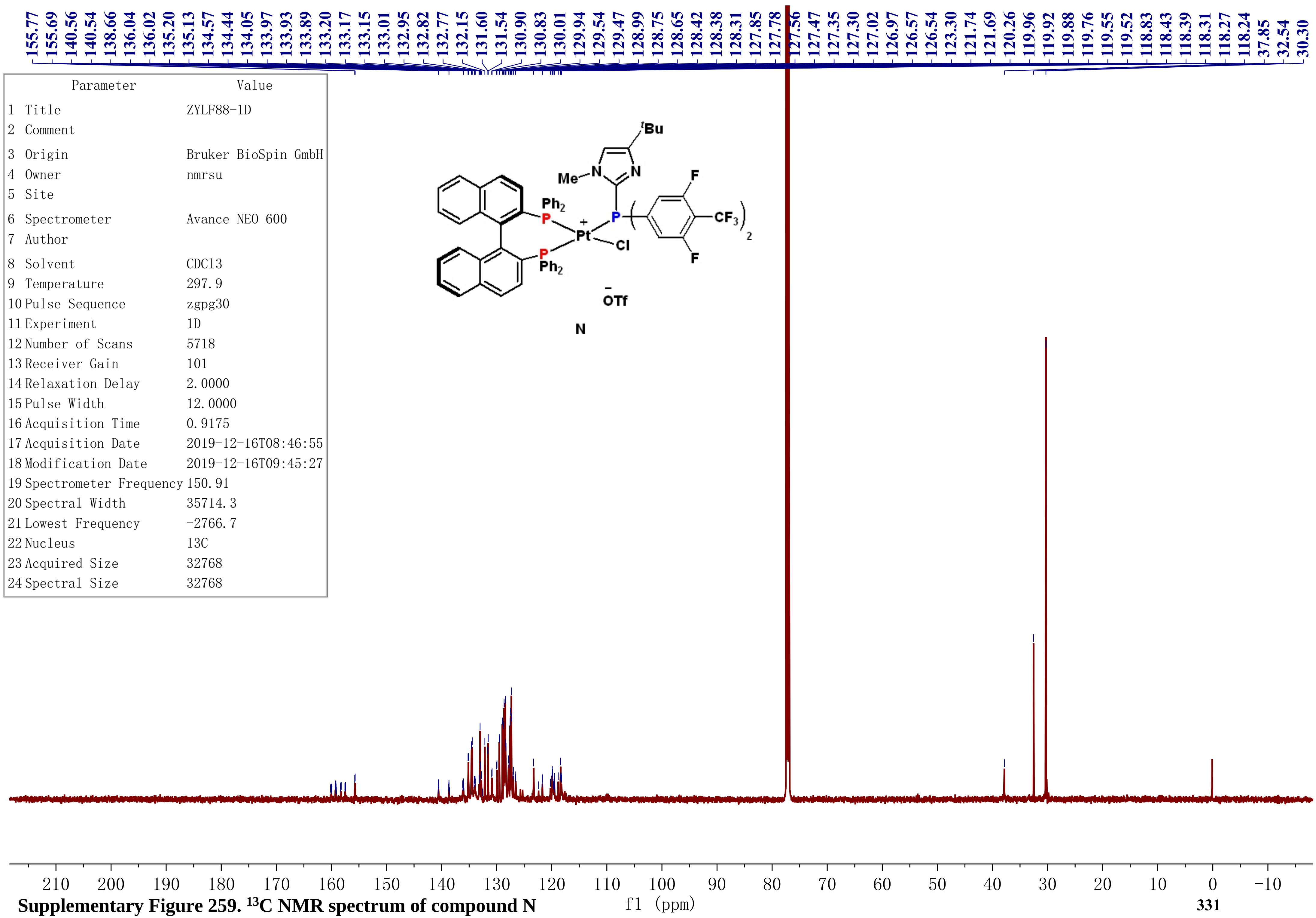

	Parameter	Value
1	Title	ZYLF88-1D
2	Comment	
3	Origin	Bruker BioSpin GmbH
4	Owner	nmrsu
5	Site	
6	Spectrometer	Avance NEO 600
7	Author	
8	Solvent	CDCl3
9	Temperature	297.9
10	Pulse Sequence	zgpg30
11	Experiment	1D
12	Number of Scans	5718
13	Receiver Gain	101
14	Relaxation Delay	2.0000
15	Pulse Width	12.0000
16	Acquisition Time	0.9175
17	Acquisition Date	2019-12-16T08:46:55
18	Modification Date	2019-12-16T09:45:27
19	Spectrometer Frequency	150.91
20	Spectral Width	35714.3
21	Lowest Frequency	-2766.7
22	Nucleus	13C
23	Acquired Size	32768
24	Spectral Size	32768

Supplementary Figure 259. ^{13}C NMR spectrum of compound N

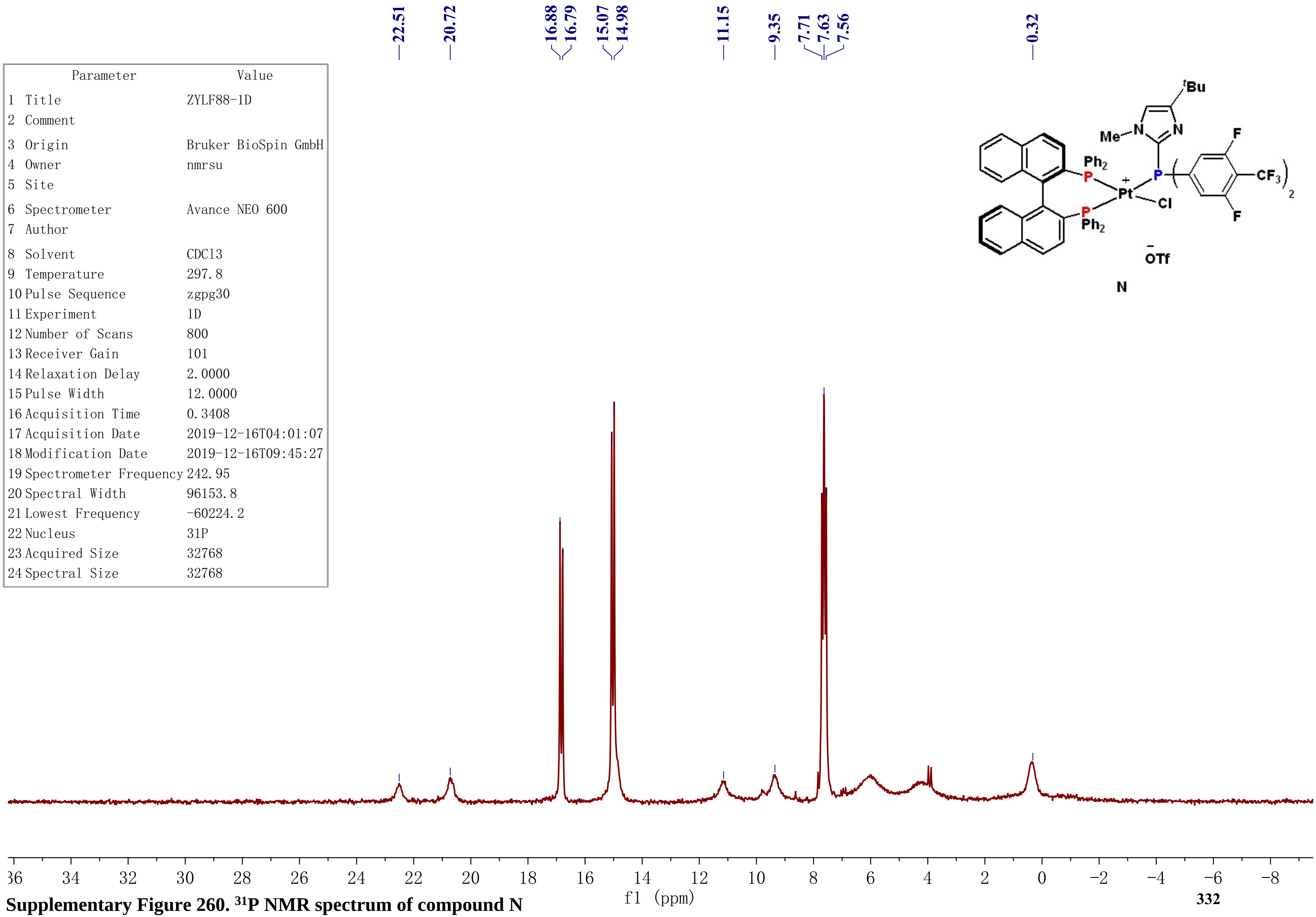

Parameter	Value
1 Title	ZYLF88-1D
2 Comment	
3 Origin	Bruker BioSpin GmbH
4 Owner	nmrsu
5 Site	
6 Spectrometer	Avance NEO 600
7 Author	
8 Solvent	CDCl3
9 Temperature	297.8
10 Pulse Sequence	zgpg30
11 Experiment	1D
12 Number of Scans	800
13 Receiver Gain	101
14 Relaxation Delay	2.0000
15 Pulse Width	12.0000
16 Acquisition Time	0.3408
17 Acquisition Date	2019-12-16T04:01:07
18 Modification Date	2019-12-16T09:45:27
19 Spectrometer Frequency	242.95
20 Spectral Width	96153.8
21 Lowest Frequency	-60224.2
22 Nucleus	31P
23 Acquired Size	32768
24 Spectral Size	32768

Supplementary Figure 260. ^{31}P NMR spectrum of compound N

	Parameter	Value
1	Title	ZYLF88-1D
2	Comment	
3	Origin	Bruker BioSpin GmbH
4	Owner	nmrsu
5	Site	
6	Spectrometer	Avance NEO 600
7	Author	
8	Solvent	CDCl3
9	Temperature	297.1
10	Pulse Sequence	zgig
11	Experiment	1D
12	Number of Scans	300
13	Receiver Gain	101
14	Relaxation Delay	1.0000
15	Pulse Width	12.0000
16	Acquisition Time	0.4981
17	Acquisition Date	2019-12-16T03:27:16
18	Modification Date	2019-12-16T09:45:26
19	Spectrometer Frequency	564.71
20	Spectral Width	131579.0
21	Lowest Frequency	-122260.0
22	Nucleus	19F
23	Acquired Size	65536
24	Spectral Size	65536

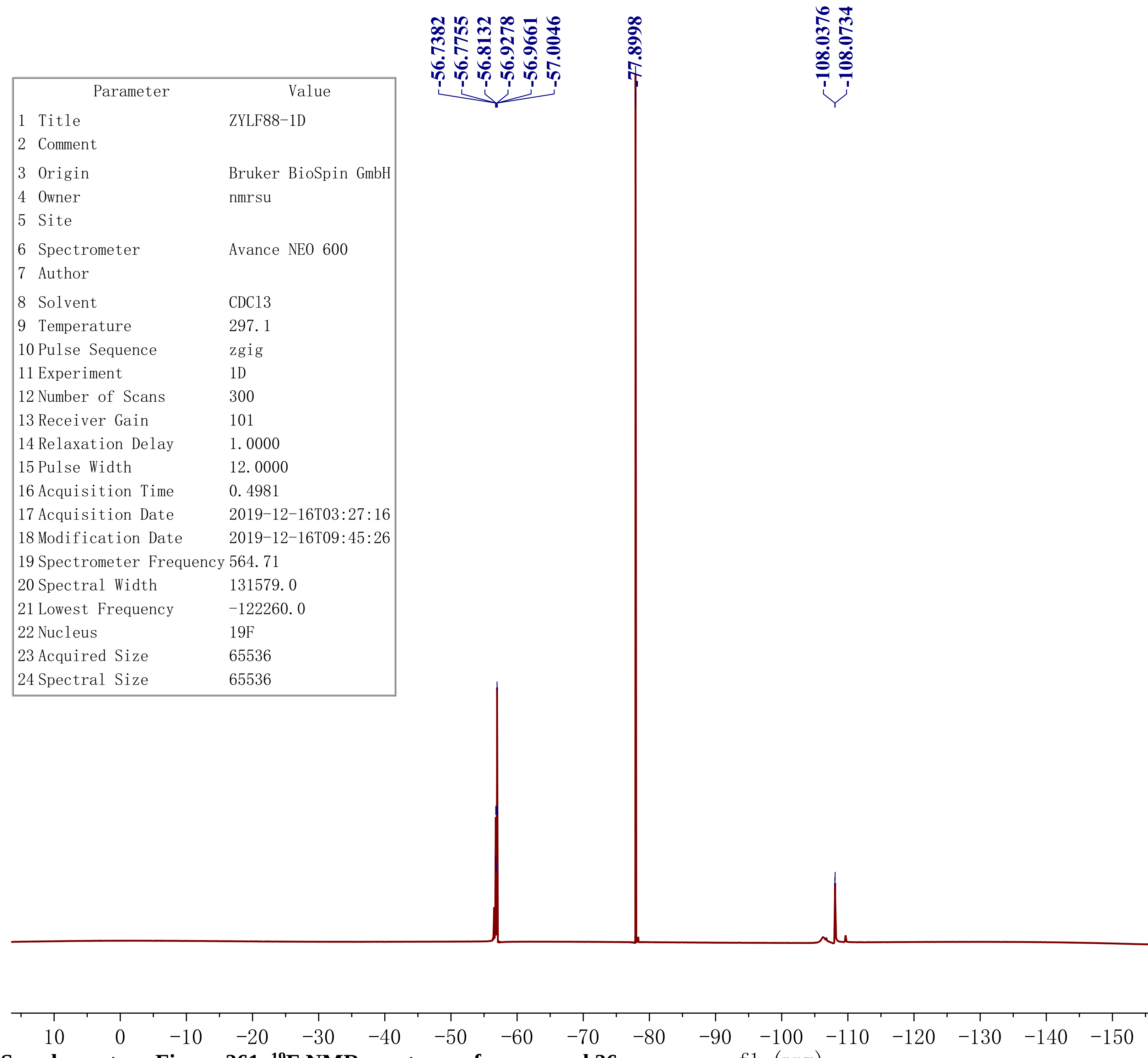

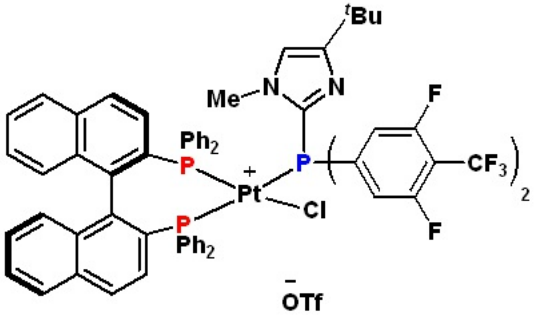

N

Supplementary Figure 261. ^{19}F NMR spectrum of compound 26c

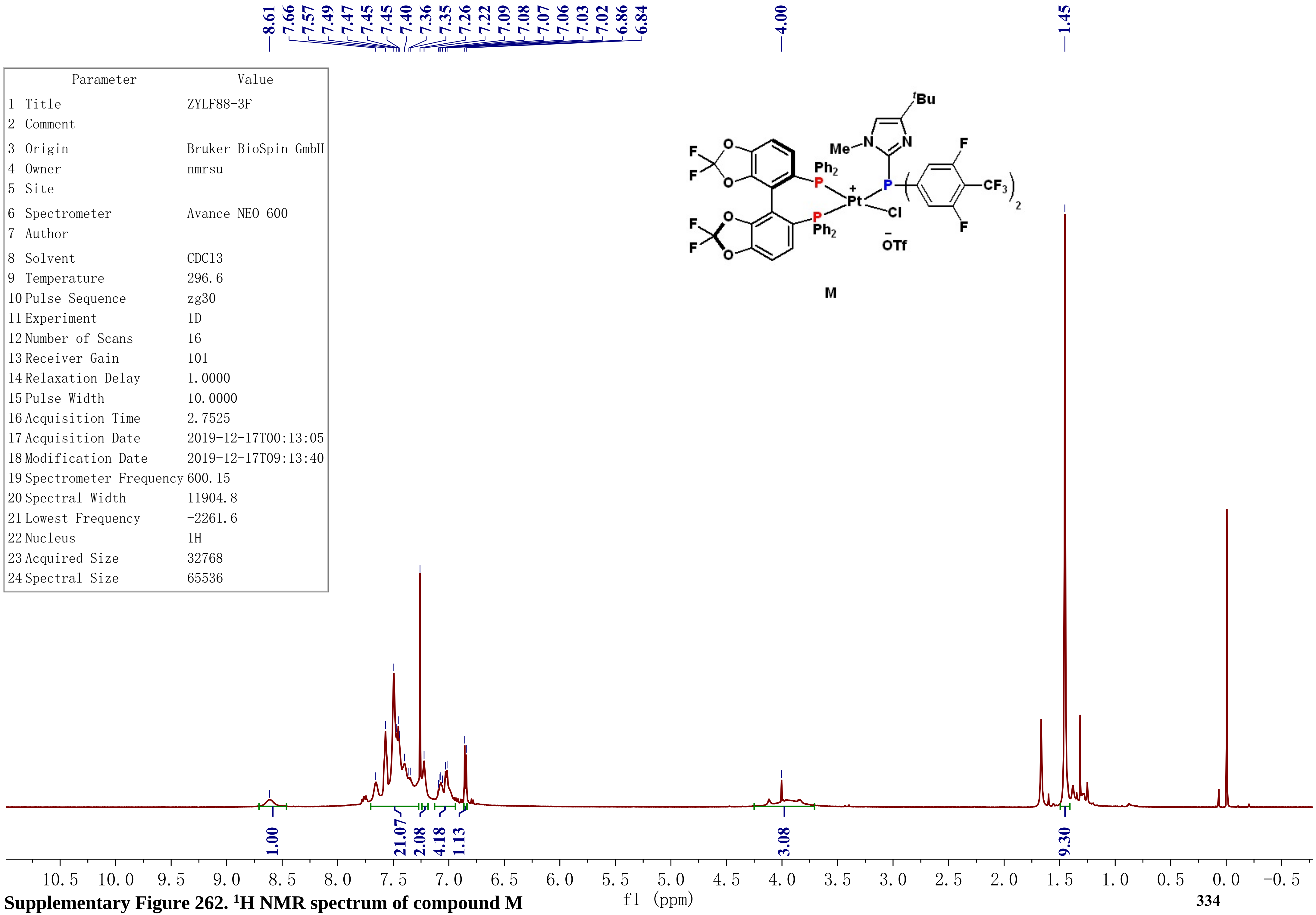

Parameter	Value
1 Title	ZYLF88-3F
2 Comment	
3 Origin	Bruker BioSpin GmbH
4 Owner	nmrsu
5 Site	
6 Spectrometer	Avance NEO 600
7 Author	
8 Solvent	CDCl3
9 Temperature	296.6
10 Pulse Sequence	zg30
11 Experiment	1D
12 Number of Scans	16
13 Receiver Gain	101
14 Relaxation Delay	1.0000
15 Pulse Width	10.0000
16 Acquisition Time	2.7525
17 Acquisition Date	2019-12-17T00:13:05
18 Modification Date	2019-12-17T09:13:40
19 Spectrometer Frequency	600.15
20 Spectral Width	11904.8
21 Lowest Frequency	-2261.6
22 Nucleus	1H
23 Acquired Size	32768
24 Spectral Size	65536

Supplementary Figure 262. ^{1}H NMR spectrum of compound M

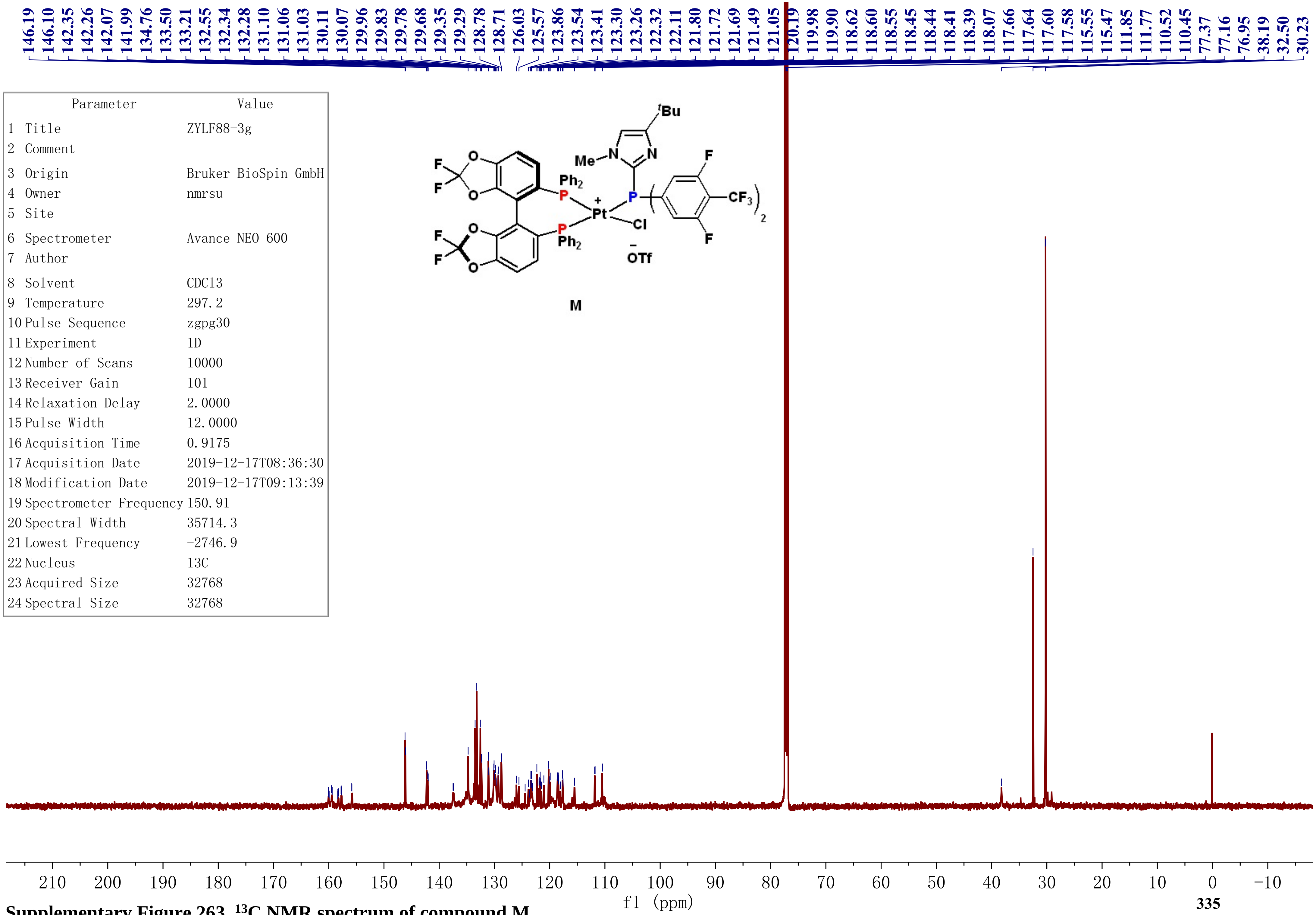

Parameter	Value
1 Title	ZYLF88-3g
2 Comment	
3 Origin	Bruker BioSpin GmbH
4 Owner	nmrsu
5 Site	
6 Spectrometer	Avance NEO 600
7 Author	
8 Solvent	CDCl3
9 Temperature	297.2
10 Pulse Sequence	zgpg30
11 Experiment	1D
12 Number of Scans	10000
13 Receiver Gain	101
14 Relaxation Delay	2.0000
15 Pulse Width	12.0000
16 Acquisition Time	0.9175
17 Acquisition Date	2019-12-17T08:36:30
18 Modification Date	2019-12-17T09:13:39
19 Spectrometer Frequency	150.91
20 Spectral Width	35714.3
21 Lowest Frequency	-2746.9
22 Nucleus	13C
23 Acquired Size	32768
24 Spectral Size	32768

Supplementary Figure 263. ^{13}C NMR spectrum of compound M

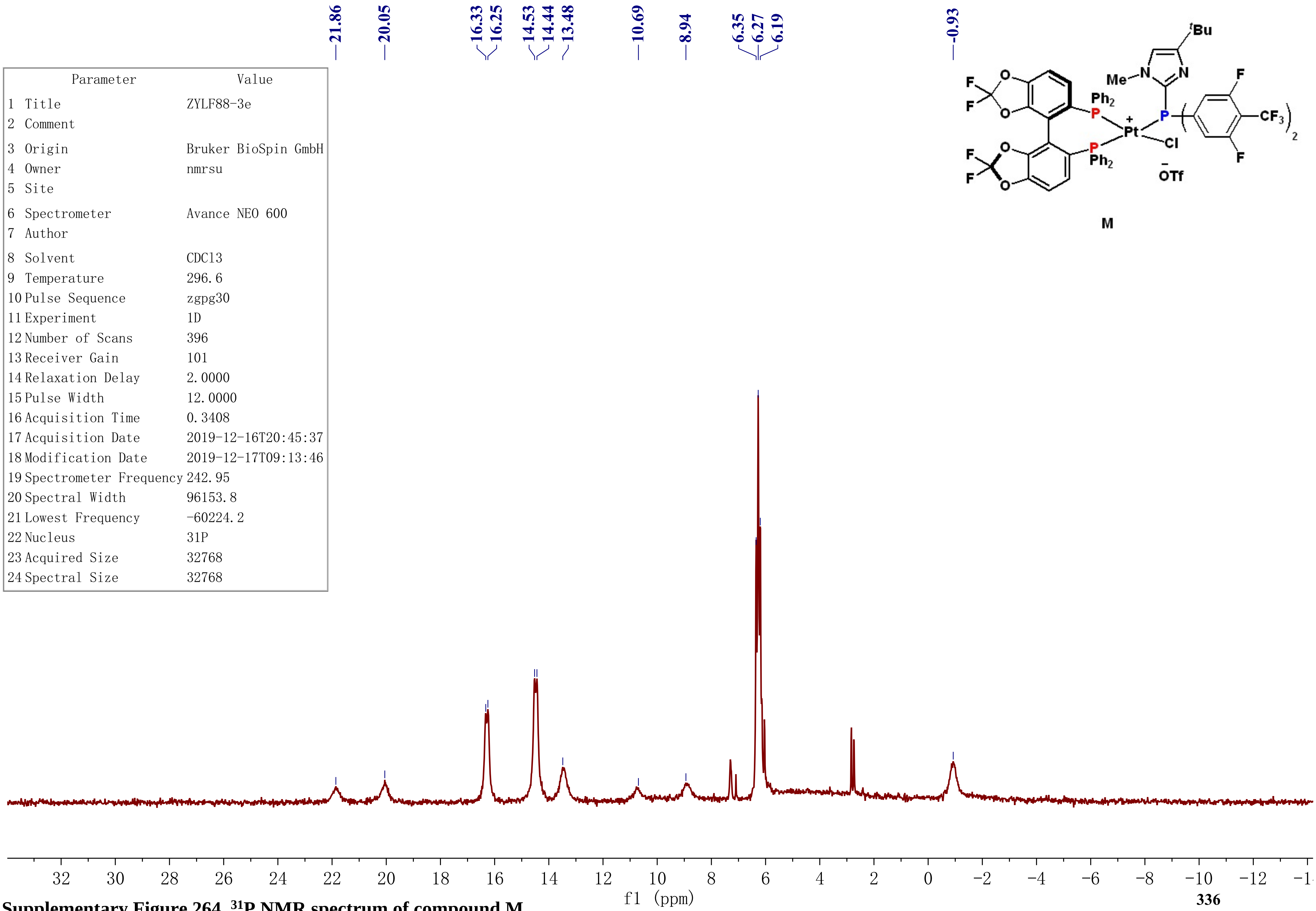

	Parameter	Value
1	Title	ZYLF88-3e
2	Comment	
3	Origin	Bruker BioSpin GmbH
4	Owner	nmrsu
5	Site	
6	Spectrometer	Avance NEO 600
7	Author	
8	Solvent	CDCl3
9	Temperature	296.6
10	Pulse Sequence	zgpg30
11	Experiment	1D
12	Number of Scans	396
13	Receiver Gain	101
14	Relaxation Delay	2.0000
15	Pulse Width	12.0000
16	Acquisition Time	0.3408
17	Acquisition Date	2019-12-16T20:45:37
18	Modification Date	2019-12-17T09:13:46
19	Spectrometer Frequency	242.95
20	Spectral Width	96153.8
21	Lowest Frequency	-60224.2
22	Nucleus	31P
23	Acquired Size	32768
24	Spectral Size	32768

Supplementary Figure 264. ^{31}P NMR spectrum of compound M

Parameter	Value
1 Title	ZYLF88-3F
2 Comment	
3 Origin	Bruker BioSpin GmbH
4 Owner	nmrsu
5 Site	
6 Spectrometer	Avance NEO 600
7 Author	
8 Solvent	CDCl3
9 Temperature	297.0
10 Pulse Sequence	zgig
11 Experiment	1D
12 Number of Scans	158
13 Receiver Gain	101
14 Relaxation Delay	1.0000
15 Pulse Width	12.0000
16 Acquisition Time	0.4981
17 Acquisition Date	2019-12-17T00:18:27
18 Modification Date	2019-12-17T09:13:40
19 Spectrometer Frequency	564.71
20 Spectral Width	131579.0
21 Lowest Frequency	-122260.0
22 Nucleus	19F
23 Acquired Size	65536
24 Spectral Size	65536

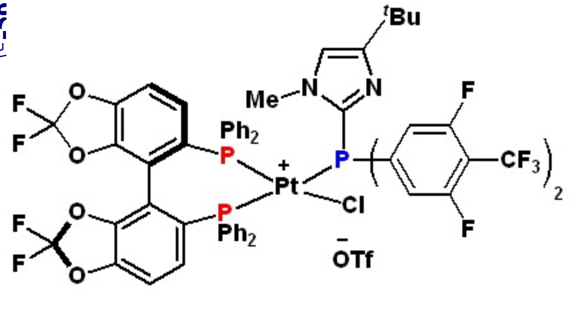

M

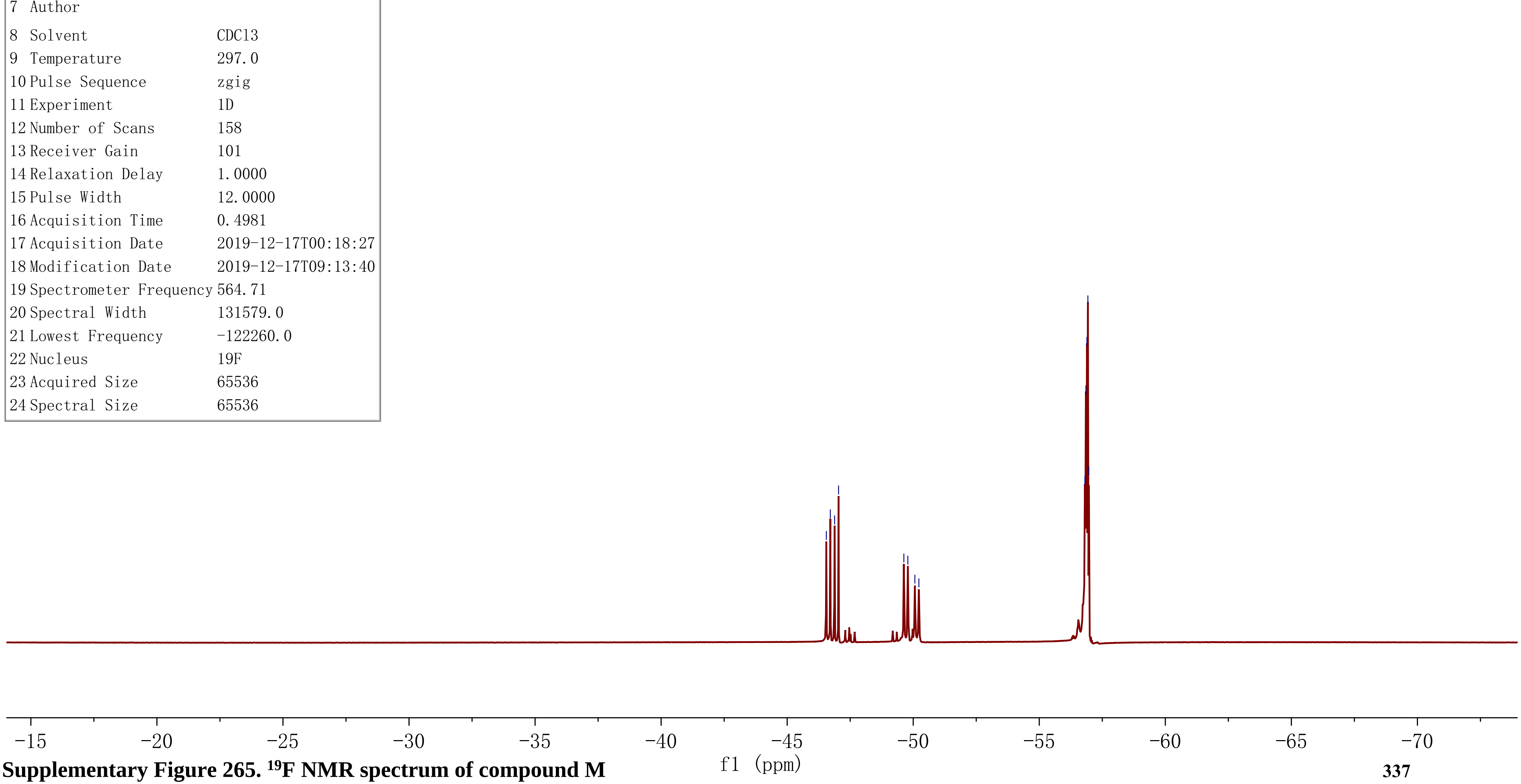

Supplementary Figure 265. ^{19}F NMR spectrum of compound M

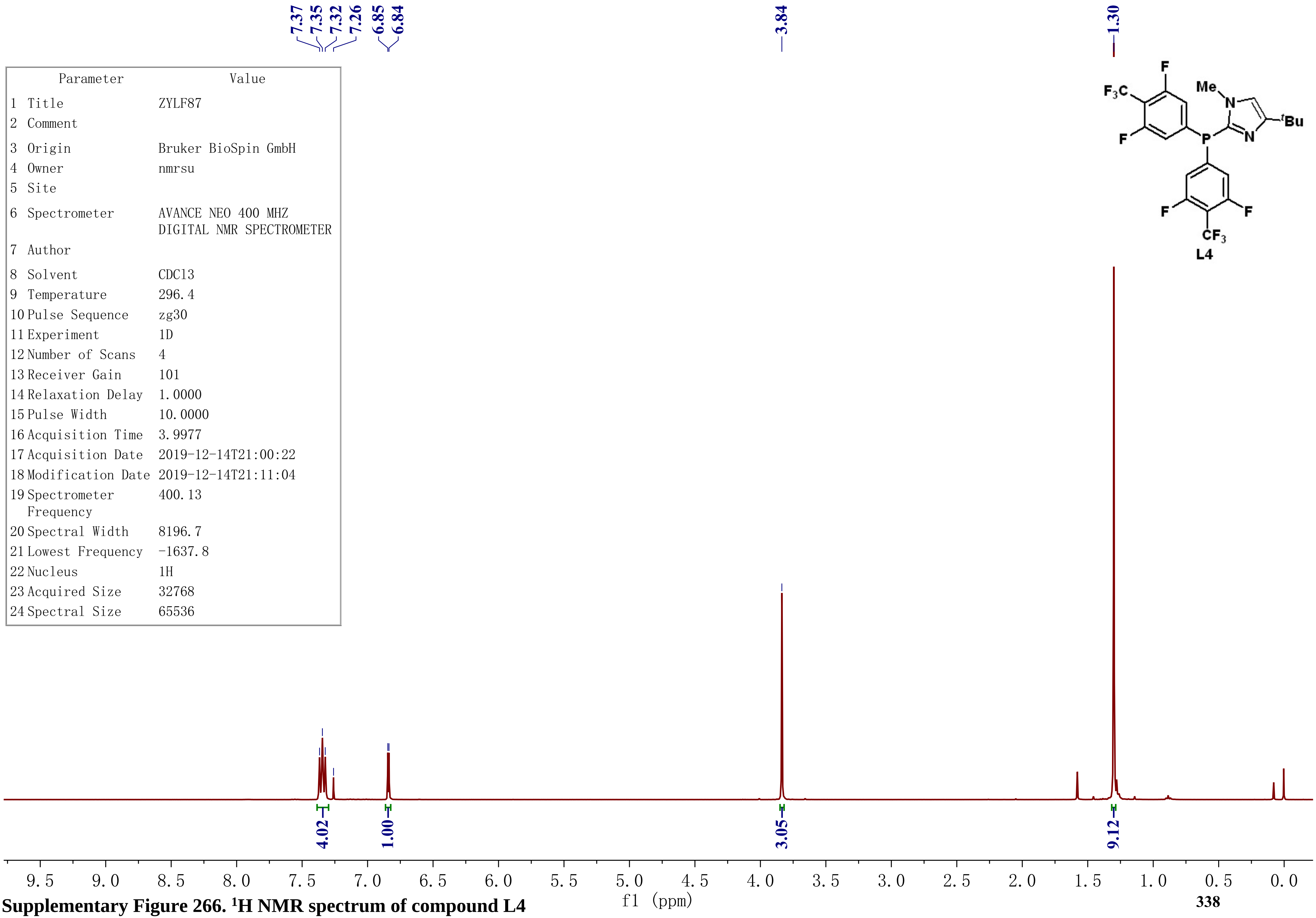

	Parameter	Value
1	Title	ZYLF87
2	Comment	
3	Origin	Bruker BioSpin GmbH
4	Owner	nmrsu
5	Site	
6	Spectrometer	AVANCE NEO 400 MHZ DIGITAL NMR SPECTROMETER
7	Author	
8	Solvent	CDCl3
9	Temperature	296.4
10	Pulse Sequence	zg30
11	Experiment	1D
12	Number of Scans	4
13	Receiver Gain	101
14	Relaxation Delay	1.0000
15	Pulse Width	10.0000
16	Acquisition Time	3.9977
17	Acquisition Date	2019-12-14T21:00:22
18	Modification Date	2019-12-14T21:11:04
19	Spectrometer Frequency	400.13
20	Spectral Width	8196.7
21	Lowest Frequency	-1637.8
22	Nucleus	1H
23	Acquired Size	32768
24	Spectral Size	65536

Supplementary Figure 266. ^{1}H NMR spectrum of compound L4

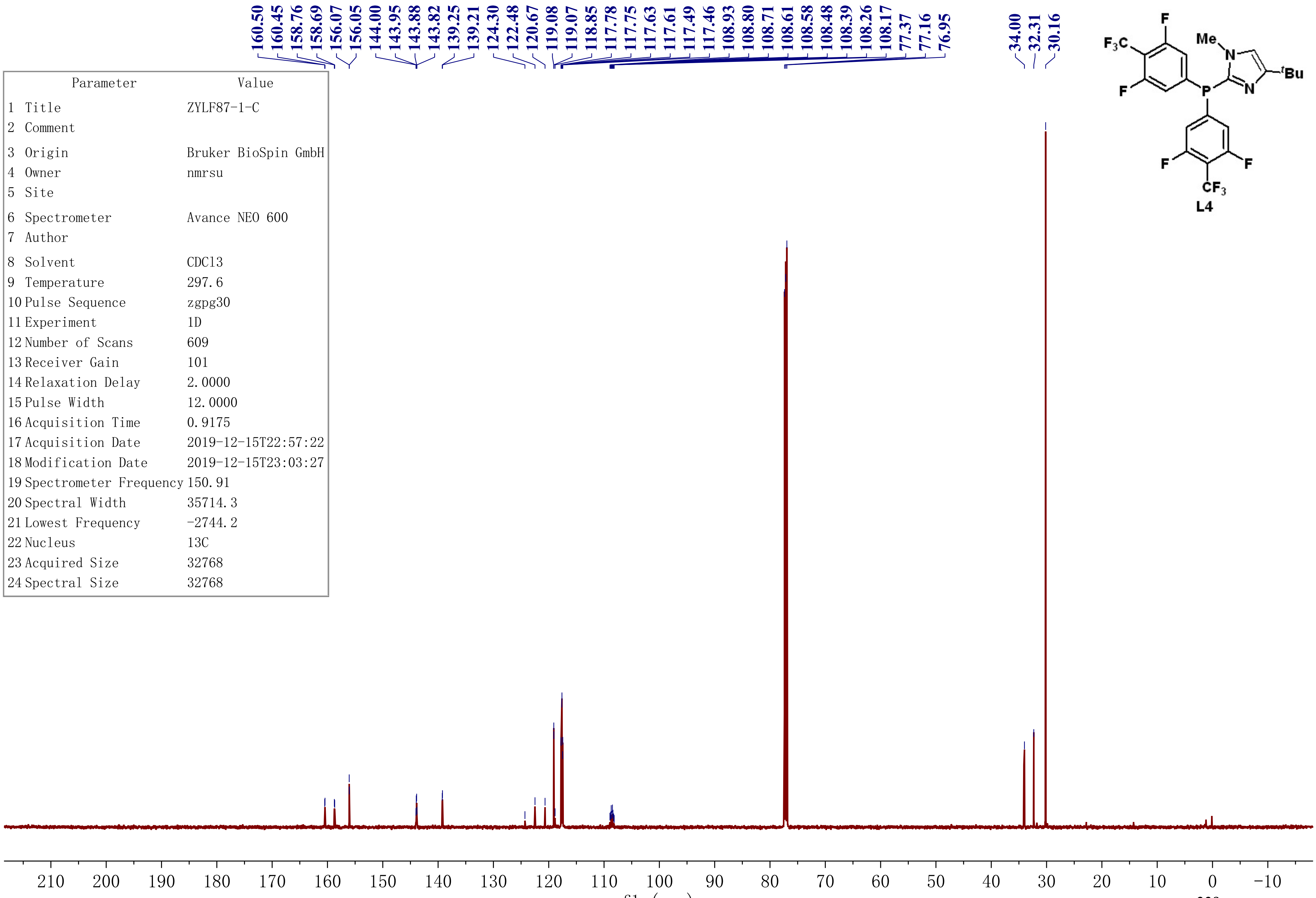

	Parameter	Value
1	Title	ZYLF87-1-C
2	Comment	
3	Origin	Bruker BioSpin GmbH
4	Owner	nmrsu
5	Site	
6	Spectrometer	Avance NEO 600
7	Author	
8	Solvent	CDCl3
9	Temperature	297.6
10	Pulse Sequence	zgpg30
11	Experiment	1D
12	Number of Scans	609
13	Receiver Gain	101
14	Relaxation Delay	2.0000
15	Pulse Width	12.0000
16	Acquisition Time	0.9175
17	Acquisition Date	2019-12-15T22:57:22
18	Modification Date	2019-12-15T23:03:27
19	Spectrometer Frequency	150.91
20	Spectral Width	35714.3
21	Lowest Frequency	-2744.2
22	Nucleus	13C
23	Acquired Size	32768
24	Spectral Size	32768

Supplementary Figure 267. ^{13}C NMR spectrum of compound L4

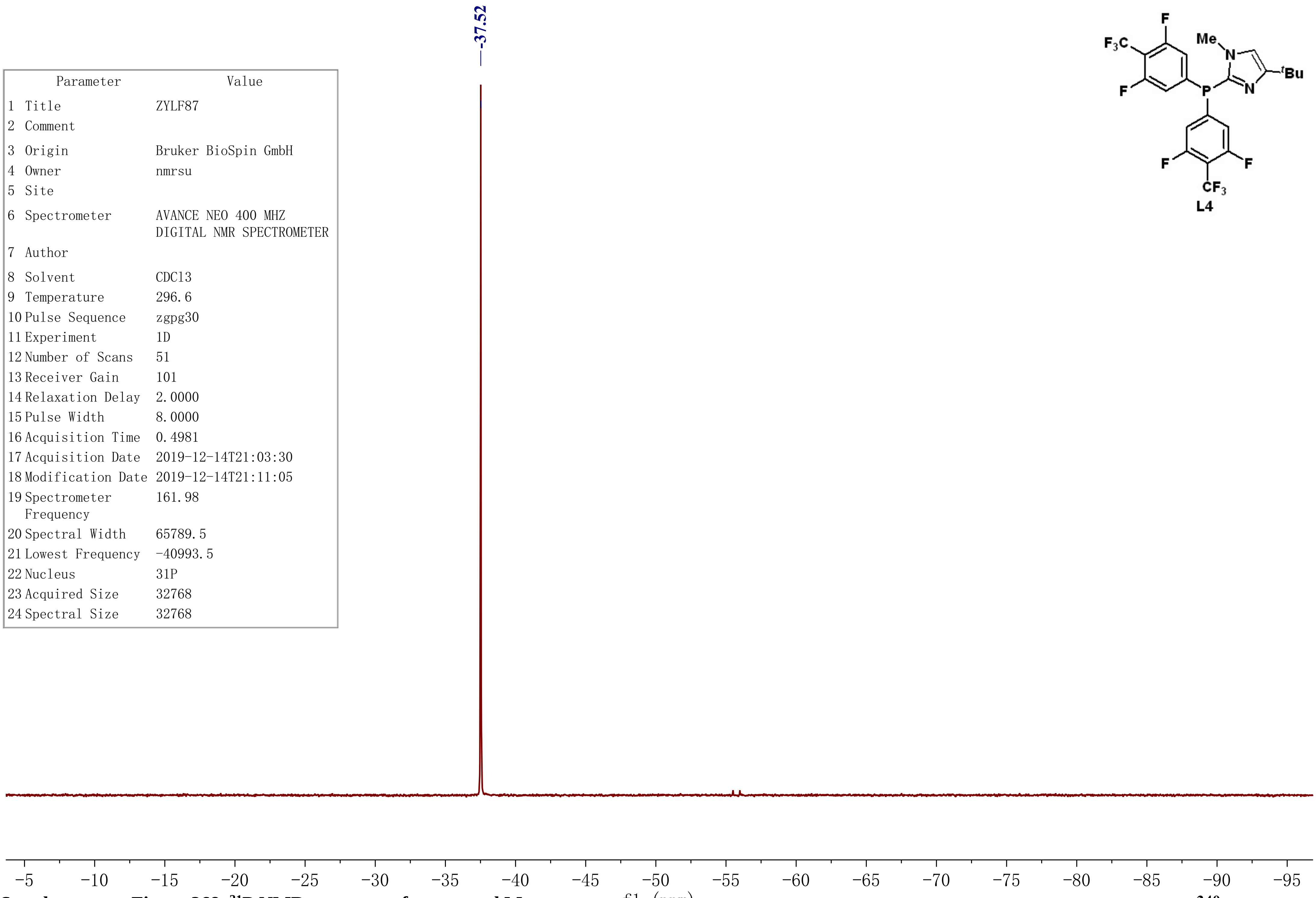

Parameter	Value
1 Title	ZYLF87
2 Comment	
3 Origin	Bruker BioSpin GmbH
4 Owner	nmrsu
5 Site	
6 Spectrometer	AVANCE NEO 400 MHZ DIGITAL NMR SPECTROMETER
7 Author	
8 Solvent	CDCl3
9 Temperature	296.6
10 Pulse Sequence	zgpg30
11 Experiment	1D
12 Number of Scans	51
13 Receiver Gain	101
14 Relaxation Delay	2.0000
15 Pulse Width	8.0000
16 Acquisition Time	0.4981
17 Acquisition Date	2019-12-14T21:03:30
18 Modification Date	2019-12-14T21:11:05
19 Spectrometer Frequency	161.98
20 Spectral Width	65789.5
21 Lowest Frequency	-40993.5
22 Nucleus	31P
23 Acquired Size	32768
24 Spectral Size	32768

Supplementary Figure 268. ^{31}P NMR spectrum of compound M

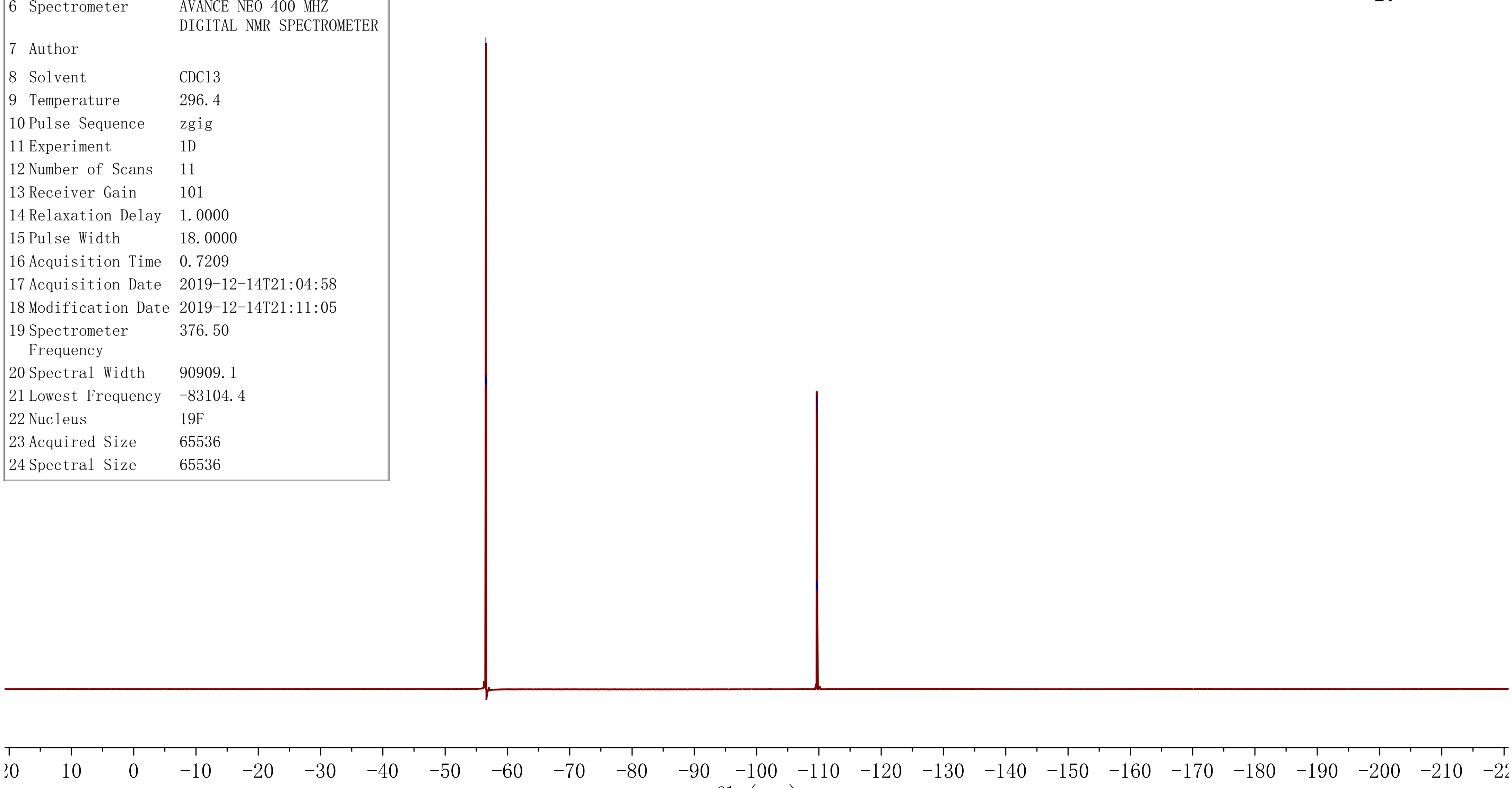

	Parameter	Value
1	Title	ZYLF87
2	Comment	
3	Origin	Bruker BioSpin GmbH
4	Owner	nmrsu
5	Site	
6	Spectrometer	AVANCE NEO 400 MHZ DIGITAL NMR SPECTROMETER
7	Author	
8	Solvent	CDCl3
9	Temperature	296.4
10	Pulse Sequence	zgig
11	Experiment	1D
12	Number of Scans	11
13	Receiver Gain	101
14	Relaxation Delay	1.0000
15	Pulse Width	18.0000
16	Acquisition Time	0.7209
17	Acquisition Date	2019-12-14T21:04:58
18	Modification Date	2019-12-14T21:11:05
19	Spectrometer Frequency	376.50
20	Spectral Width	90909.1
21	Lowest Frequency	-83104.4
22	Nucleus	19F
23	Acquired Size	65536
24	Spectral Size	65536

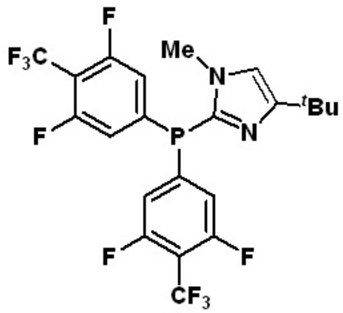

L4

Supplementary Figure 269. ^{19}F NMR spectrum of compound L4

Parameter	Value
1 Title	ZYLT144-F
2 Comment	
3 Origin	Bruker BioSpin GmbH
4 Owner	nmrsu
5 Site	
6 Spectrometer	AVANCE NEO 400 MHZ DIGITAL NMR SPECTROMETER
7 Author	
8 Solvent	CDCl3
9 Temperature	297.0
10 Pulse Sequence	zg30
11 Experiment	1D
12 Number of Scans	16
13 Receiver Gain	32
14 Relaxation Delay	1.0000
15 Pulse Width	10.0000
16 Acquisition Time	3.9977
17 Acquisition Date	2019-09-04T00:20:38
18 Modification Date	2019-09-04T18:35:00
19 Spectrometer Frequency	400.13
20 Spectral Width	8196.7
21 Lowest Frequency	-1637.7
22 Nucleus	1H
23 Acquired Size	32768
24 Spectral Size	65536

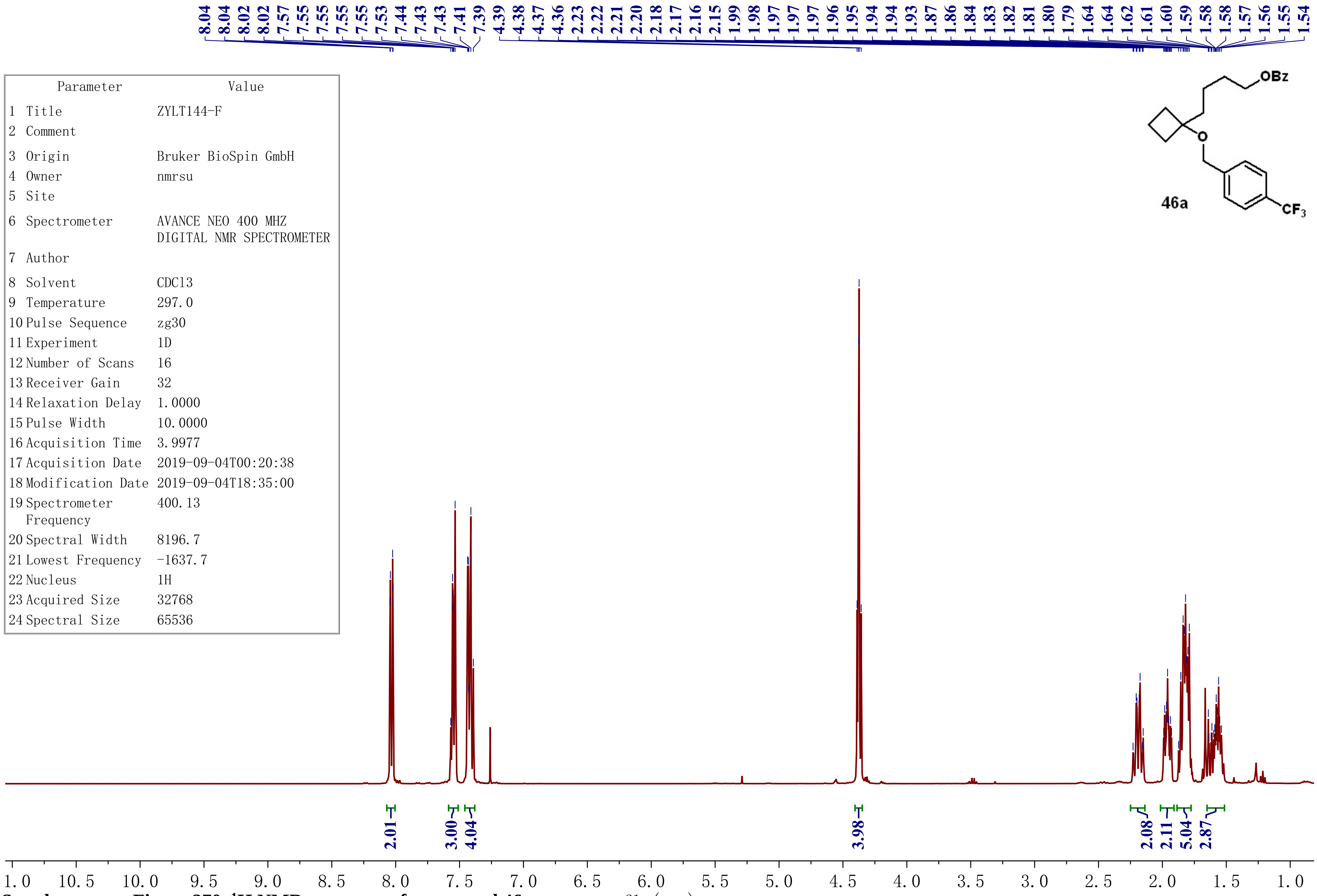

Supplementary Figure 270. ^{1}H NMR spectrum of compound 46a

Parameter	Value
1 Title	ZYLT144-F1
2 Comment	
3 Origin	Bruker BioSpin GmbH
4 Owner	nmrsu
5 Site	
6 Spectrometer	AVANCE NEO 400 MHZ DIGITAL NMR SPECTROMETER
7 Author	
8 Solvent	CDCl3
9 Temperature	298.2
10 Pulse Sequence	zgpg30
11 Experiment	1D
12 Number of Scans	800
13 Receiver Gain	32
14 Relaxation Delay	2.0000
15 Pulse Width	10.0000
16 Acquisition Time	1.3763
17 Acquisition Date	2019-09-04T08:52:43
18 Modification Date	2019-09-04T18:35:13
19 Spectrometer Frequency	100.61
20 Spectral Width	23809.5
21 Lowest Frequency	-1833.4
22 Nucleus	13C
23 Acquired Size	32768
24 Spectral Size	32768

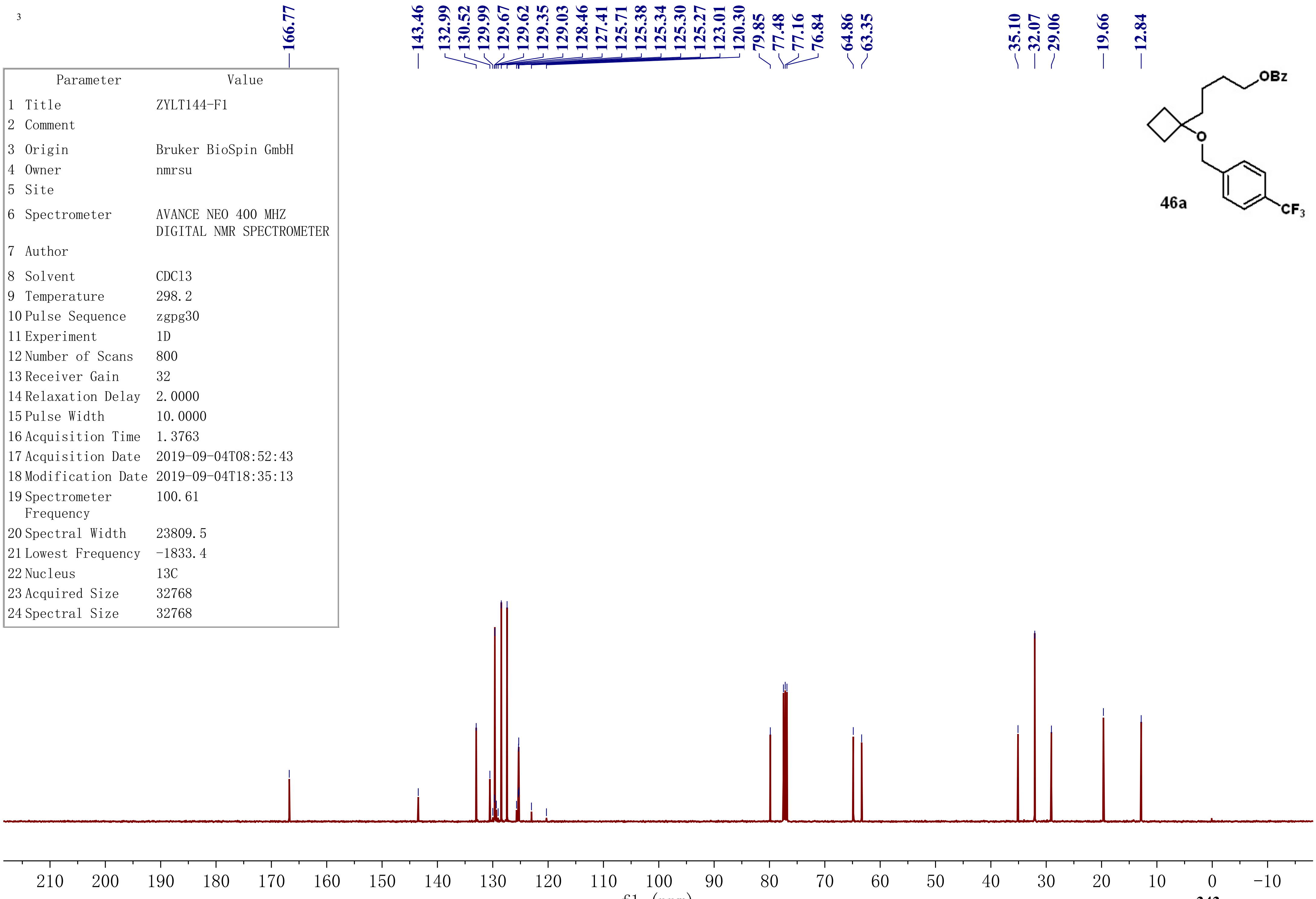

46a

Supplementary Figure 271. ^{13}C NMR spectrum of compound 46a

	Parameter	Value
1	Title	ZYLT144-F
2	Comment	
3	Origin	Bruker BioSpin GmbH
4	Owner	nmrsu
5	Site	
6	Spectrometer	AVANCE NEO 400 MHZ DIGITAL NMR SPECTROMETER
7	Author	
8	Solvent	CDCl3
9	Temperature	297.2
10	Pulse Sequence	zgig
11	Experiment	1D
12	Number of Scans	16
13	Receiver Gain	101
14	Relaxation Delay	1.0000
15	Pulse Width	18.0000
16	Acquisition Time	0.7209
17	Acquisition Date	2019-09-04T00:22:03
18	Modification Date	2019-09-04T18:35:04
19	Spectrometer Frequency	376.50
20	Spectral Width	90909.1
21	Lowest Frequency	-83104.4
22	Nucleus	19F
23	Acquired Size	65536
24	Spectral Size	65536

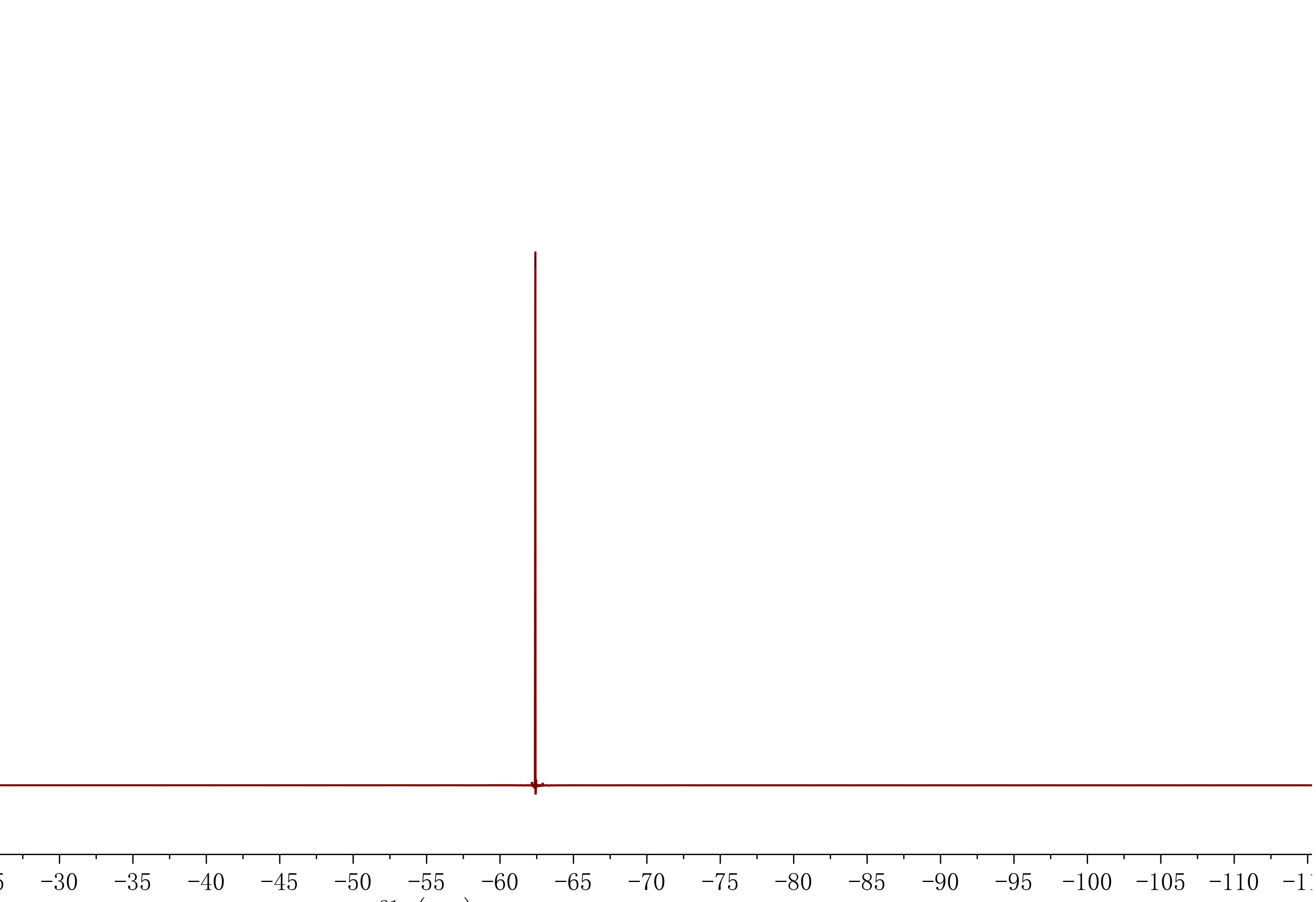

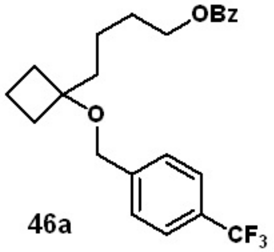

46a

Supplementary Figure 272. ^{19}F NMR spectrum of compound 46a

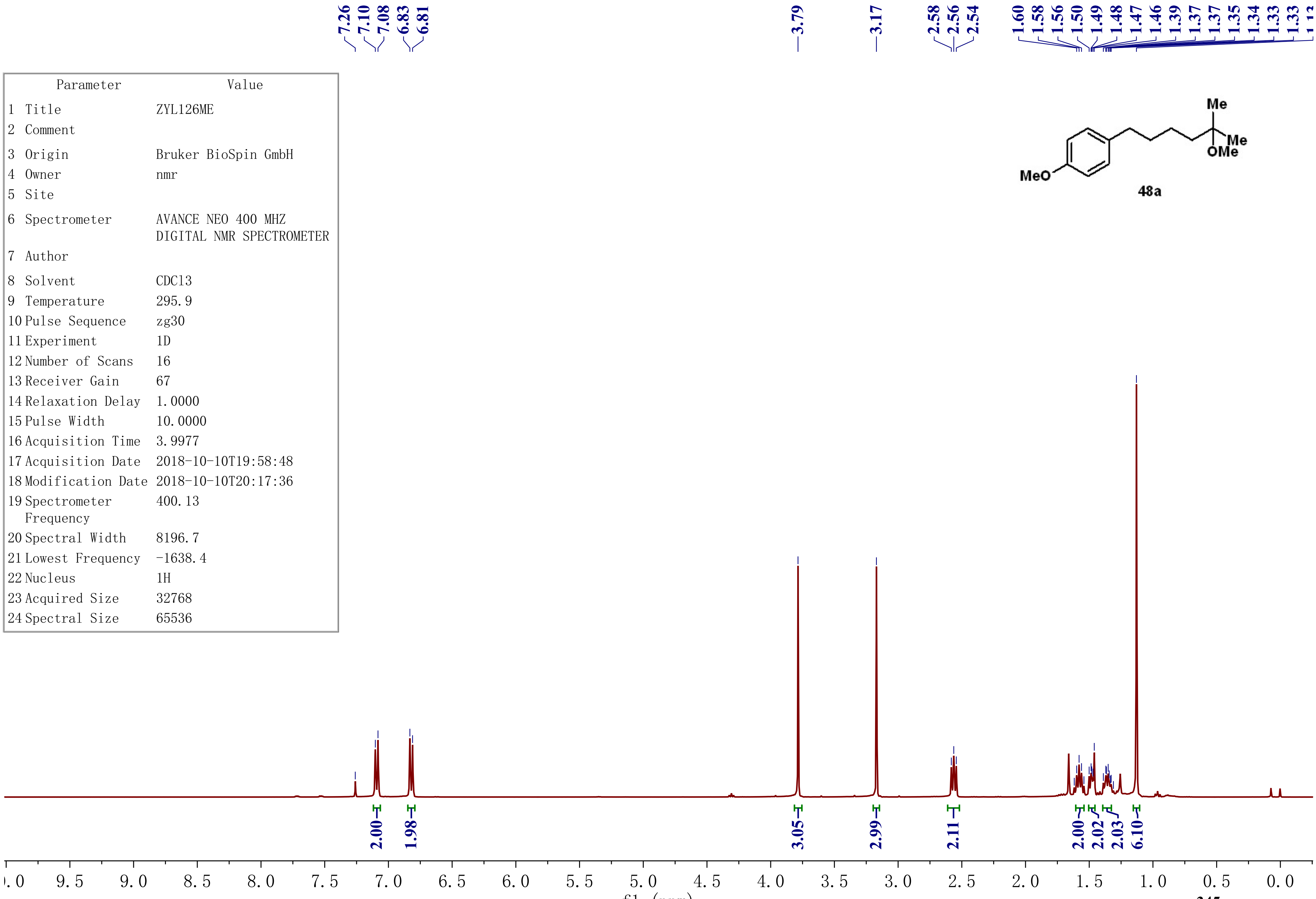

	Parameter	Value
1	Title	ZYL126ME
2	Comment	
3	Origin	Bruker BioSpin GmbH
4	Owner	nmr
5	Site	
6	Spectrometer	AVANCE NEO 400 MHZ DIGITAL NMR SPECTROMETER
7	Author	
8	Solvent	CDCl3
9	Temperature	295.9
10	Pulse Sequence	zg30
11	Experiment	1D
12	Number of Scans	16
13	Receiver Gain	67
14	Relaxation Delay	1.0000
15	Pulse Width	10.0000
16	Acquisition Time	3.9977
17	Acquisition Date	2018-10-10T19:58:48
18	Modification Date	2018-10-10T20:17:36
19	Spectrometer Frequency	400.13
20	Spectral Width	8196.7
21	Lowest Frequency	-1638.4
22	Nucleus	1H
23	Acquired Size	32768
24	Spectral Size	65536

48a

Supplementary Figure 273. ¹H NMR spectrum of compound 48a

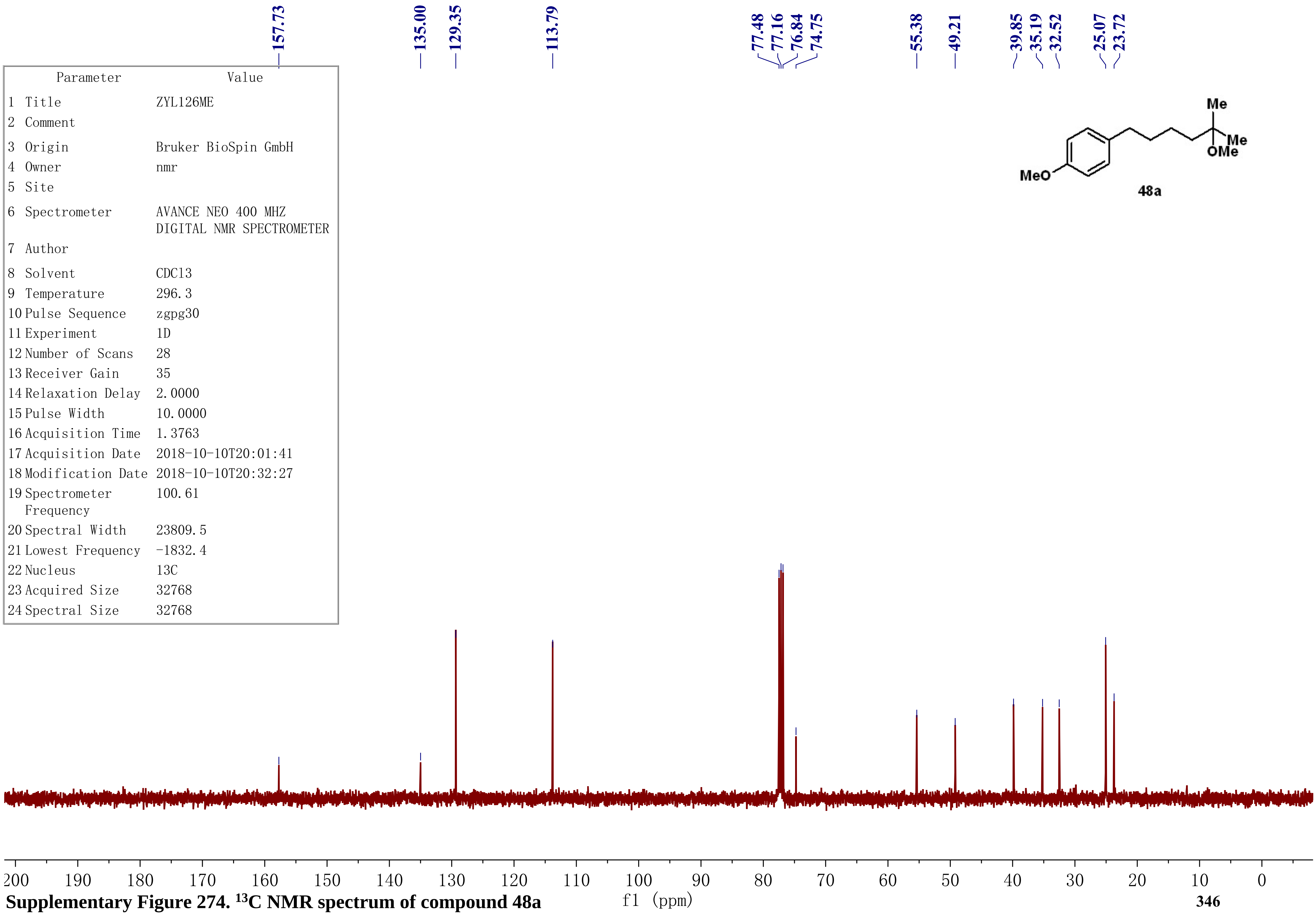

	Parameter	Value
1	Title	ZYL126ME
2	Comment	
3	Origin	Bruker BioSpin GmbH
4	Owner	nmr
5	Site	
6	Spectrometer	AVANCE NEO 400 MHZ DIGITAL NMR SPECTROMETER
7	Author	
8	Solvent	CDCl3
9	Temperature	296.3
10	Pulse Sequence	zgpg30
11	Experiment	1D
12	Number of Scans	28
13	Receiver Gain	35
14	Relaxation Delay	2.0000
15	Pulse Width	10.0000
16	Acquisition Time	1.3763
17	Acquisition Date	2018-10-10T20:01:41
18	Modification Date	2018-10-10T20:32:27
19	Spectrometer Frequency	100.61
20	Spectral Width	23809.5
21	Lowest Frequency	-1832.4
22	Nucleus	13C
23	Acquired Size	32768
24	Spectral Size	32768

48a

Supplementary Figure 274. ^{13}C NMR spectrum of compound 48a
